# Comparing modern identification methods for wild bees: Metabarcoding and image-based morphological taxonomic assignment

**DOI:** 10.1371/journal.pone.0301474

**Published:** 2024-04-02

**Authors:** Cassandra D. Smith, Robert S. Cornman, Jennifer A. Fike, Johanna M. Kraus, Sara J. Oyler-McCance, Carrie E. Givens, Michelle L. Hladik, Mark W. Vandever, Dana W. Kolpin, Kelly L. Smalling

**Affiliations:** 1 Oregon Water Science Center, U.S. Geological Survey, Bend, Oregon, United States of America; 2 Fort Collins Science Center, U.S. Geological Survey, Fort Collins, Colorado, United States of America; 3 Columbia Environmental Research Center, U.S. Geological Survey, Columbia, Missouri, United States of America; 4 Upper Midwest Water Science Center, U.S. Geological Survey, Lansing, Michigan, United States of America; 5 California Water Science Center, U.S. Geological Survey, Sacramento, California, United States of America; 6 Central Midwest Water Science Center, U.S. Geological Survey, Iowa City, Iowa, United States of America; 7 New Jersey Water Science Center, U.S. Geological Survey, Lawrenceville, New Jersey, United States of America; CNRS, University Paul Sabatier, FRANCE

## Abstract

With the decline of bee populations worldwide, studies determining current wild bee distributions and diversity are increasingly important. Wild bee identification is often completed by experienced taxonomists or by genetic analysis. The current study was designed to compare two methods of identification including: (1) morphological identification by experienced taxonomists using images of field-collected wild bees and (2) genetic analysis of composite bee legs (multiple taxa) using metabarcoding. Bees were collected from conservation grasslands in eastern Iowa in summer 2019 and identified to the lowest taxonomic unit using both methods. Sanger sequencing of individual wild bee legs was used as a positive control for metabarcoding. Morphological identification of bees using images resulted in 36 unique taxa among 22 genera, and >80% of *Bombus* specimens were identified to species. Metabarcoding was limited to genus-level assignments among 18 genera but resolved some morphologically similar genera. Metabarcoding did not consistently detect all genera in the composite samples, including kleptoparasitic bees. Sanger sequencing showed similar presence or absence detection results as metabarcoding but provided species-level identifications for cryptic species (i.e., *Lasioglossum*). Genus-specific detections were more frequent with morphological identification than metabarcoding, but certain genera such as *Ceratina* and *Halictus* were identified equally well with metabarcoding and morphology. Genera with proportionately less tissue in a composite sample were less likely to be detected using metabarcoding. Image-based methods were limited by image quality and visible morphological features, while genetic methods were limited by databases, primers, and amplification at target loci. This study shows how an image-based identification method compares with genetic techniques, and how in combination, the methods provide valuable genus- and species-level information for wild bees while preserving tissue for other analyses. These methods could be improved and transferred to a field setting to advance our understanding of wild bee distributions and to expedite conservation research.

## Introduction

Pollination of wild plants and agricultural crops is a highly valuable ecosystem service worldwide [1]. Managed pollinators (e.g., honey bees) efficiently pollinate many crop types and are adaptable to various habitats [2]. Additionally, pollination by wild bees increases some crop production [2,3], and wild bee diversity may provide resilience as threats to managed pollinators continue to increase [1,4]. Habitat loss and degradation, in addition to intensive agriculture practices such as pesticide use, stress wild bees and can cause mortality [5,6]. Conserving wild bee habitats in agricultural landscapes, however, can increase wild bee diversity and abundance [7,8].

Considering that many bee populations are in decline, monitoring their ranges and the effects of conservation practices on abundance and richness are crucial for managing their recovery [9]. Basic information about wild bee distribution is limited in many regions of the world [10,11] in part because of the taxonomic expertise and funding necessary for identification [10]. The traditional method of identifying wild bees requires expert taxonomists to study pinned specimens under a microscope while referencing detailed dichotomous keys [12–14]. For many wild bee species, identification may be based on morphological features that are difficult to discern, and the time-intensive method creates backlogs that can delay pollinator research [14–16]. Bee dissection may be required for identification through the traditional method [17,18], and the pinning and identification process results in dry specimens which are unsuitable for other purposes, such as the analysis of pesticides or microorganisms of interest.

Alternative methods for identification could expedite studies, reduce costs, maintain sample integrity, and obtain multiple types of information from a single bee specimen. Both photographic and molecular metabarcoding methods can provide low-level taxonomic information regarding wild bees in a potentially less time-intensive and less destructive manner compared to the often-preferred pinning-based approach. Photographs can be used to identify many wild bees to genera and sometimes to species level [14,19,20]. Image-based identifications are limited by factors such as image quality (e.g., color saturation), condition of bee, and visible morphological features. Additionally, the photographer must know the unique features to photograph, requiring some taxonomic expertise [19]. While collecting wild bees for scientific studies should be done thoughtfully [21], some study questions require physical samples of bee tissue, and image-based identifications can preserve specimens for those analyses.

Sequencing DNA “barcodes” (i.e., taxonomically informative genetic loci that can be amplified with a single primer set) has the potential to accurately identify bees, including cryptic species (e.g., *Lasioglossum* and *Andrena*), quickly and inexpensively [15,22,23]. Molecular methods have been used reliably to identify honey bees since the 1990s [24]. The utility of molecular techniques for taxonomic identification, however, depends on the existence of DNA sequences from vouchered specimens to provide a reference library for comparison. While such sequences do exist in reference libraries for numerous bee species, databases remain incomplete for many North American wild bees [23,25]. This limitation is likely to be reduced in the future as regional studies are actively adding to and refining the Barcode of Life Data Systems (BOLD) [26] and other repositories (e.g., GenBank) for wild bee DNA sequences [15,25,27]. Metabarcoding, which involves sequencing many independent copies of a barcode locus to identify species in a composite sample, has the potential for even faster and cheaper taxonomic characterizations. Metabarcoding will become more useful as databases are expanded [12] and primer specificity is refined. Studies with limited funding could analyze composite bee samples via DNA metabarcoding instead of genetically analyzing individual bees, depending on the bee species collected and study objectives.

Here we compared image-based bee identification (hereafter referred to as morphological identification) and metabarcoding approaches to identifying wild bee taxa while preserving the tissues for other analyses (e.g., Hladik et al. [28]), summarizing the relative strengths and weaknesses of both methods. Wild bees were collected from Iowa as part of a study designed to understand the effects of land use and pesticide exposure on bee health and diversity (see [28,29]). The hypotheses addressed in this study included: (1) there would be general agreement among the two identification methods, (2) the morphological identification would provide definitive information on the presence of specific taxa, and (3) the molecular data would resolve some cryptic species that were indiscernible from the images using the photographic approach. This study assessed non-traditional identification methods that make efficient use of collected bee specimens and discusses the most applicable identification method for future field studies of wild bees [13].

## Materials and methods

### Specimen collection

Hectare (100 × 100 m) plots were established in 24 conservation grassland fields in eastern Iowa. Pollinators were collected from the 24 study fields over 8 consecutive days in July 2019 and again over 7 consecutive days in August 2019, resulting in 48 site visits. Sweep nets were used to collect pollinators from each quadrant (50 × 50 m) of the hectare within each field for 15 minutes (min), not including handling time, for a total of 1 hour per hectare. Floral resources and bees were targeted during the sweep-net activity. Pollinators collected with sweep nets were placed in clean plastic bags and immediately frozen. Sampling details are further described in Kraus et al. [29].

### Morphological identification

**Fig 1:**
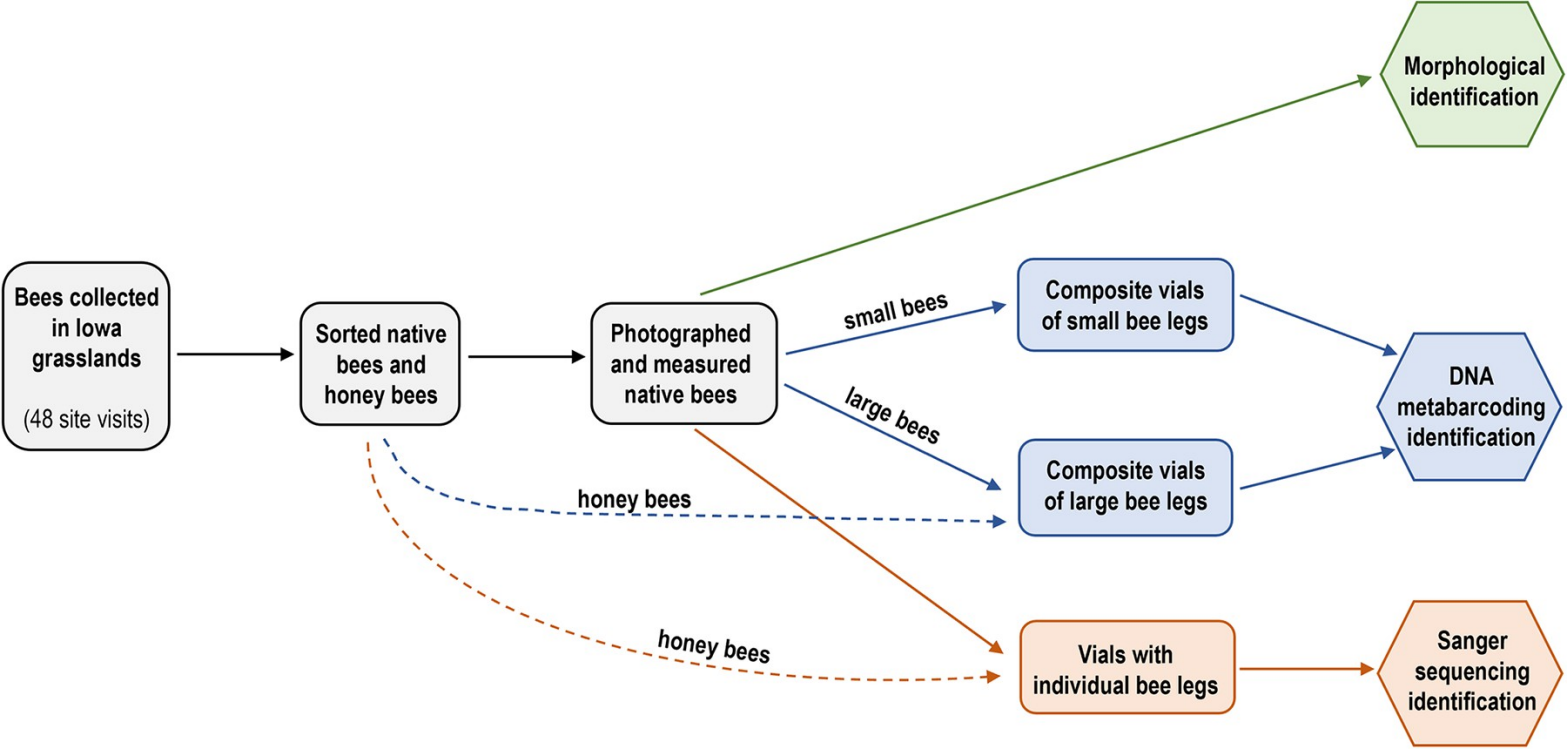
Flowchart of the wild bee identification processing steps for the morphological (nondestructive to specimen) and molecular (partially destructive to specimen) approaches.

Following collection, specimens remained frozen in a conventional freezer (-20°C) during processing to maintain sample integrity for companion pesticide and microbial analysis of bee tissue [28]. The bee carapace, however, was allowed to thaw slightly for 1–2 min before images were taken. Each bee was photographed from several angles using an AmScope microscope fitted with an MU1400 digital camera (20X magnification; Fig 1). Bee images were used for morphological identification to the lowest taxonomic unit based on keys including DiscoverLife, and an expert (S. Droege) validated each identification [30,31]. The intertegular distance (ITD) [32] of each bee was measured using the AmScope microscope and calibrated ruler. Bees were then categorized as large (>3 mm) or small (≤3 mm) based on measured ITDs to standardize the tissue mass of individual bees represented in a sample. Size thresholds were based on honey bees, which are generally considered medium-sized bees; three honey bee ITDs were measured in this study (average = 3.03 mm).

### Molecular approaches

For DNA metabarcoding, a leg from each individual bee collected at a site visit was removed. Legs of large bees and small bees from each site visit were composited separately (Fig 1; n = 86 composite vials: 42 large and 44 small). Composite samples ranged from 1 to 73 bee legs (mean = 14 bee legs per composite vial), and the number of bee legs in a composite vial was determined by the number of small or large bees collected at that site visit. Honey bees (*Apis mellifera*) were easily identified morphologically and were not photographed, but *Apis* bees were included in analyses. *Apis* bees were counted and recorded from each site, and a leg was included from each *Apis* bee in the large vial for DNA analysis since the average of 3 honey bee ITDs was >3 mm.

To test the efficacy of the metabarcoding approach, and to act as a positive control for both methods, we also sequenced a subset of bee legs individually using standard Sanger sequencing approaches (i.e., DNA barcoding [33]). This subset of samples had been identified by image-based morphology and was used to confirm that the trained assignment algorithm used for metabarcoding had high success on local populations. These samples were also used to identify instances of morphological-genetic discordance that require further effort to resolve. For Sanger sequencing, a total of 69 bees collected from five site visits with relatively high diversity were selected. A second leg was removed from each bee and placed in an individual vial (i.e., tissue from one bee per vial) for sequencing (Fig 1).

DNA was isolated from composite samples and individual bee legs using a DNeasy Blood and Tissue Kit (Qiagen), following the protocol for the purification of DNA from nails, hair, or feathers. All vials were checked to ensure the bee legs were completely submerged in the lysis buffer; more lysis buffer was added to cover the legs, if needed. Samples were then homogenized by vortexing. DNA was eluted in 120 μL Buffer AE; all samples were eluted in the same volume of Buffer AE regardless of the amount of starting tissue. Negative controls with no tissue added were included during DNA extraction to monitor for sample contamination. Samples were quantified using a Qubit Broad Range DNA Assay (Life Technologies). Using this method, DNA quantifications ranged from about 0.05 to 49.6 ng/μL.

#### Sanger sequencing of individual bees

For the individual bee legs, two mtDNA regions were amplified and sequenced each using two different sets of primers. The cytochrome *c* oxidase I (CO1) region was assessed using the primer pairs: (1) LCO1490 and HCO2198 [34] (hereafter called “Folmer primers”), which amplify a ~658 bp region, and (2) LepF and mlCO1intBeeR [11] (hereafter called “Tang primers”), which amplify a ~319 bp region (S1 Table). The 16S gene was also examined using two primer pairs: (1) Ins16S_F1short and Ins16S_R1short [35] (hereafter called “Clarke primers”), which amplify a ~156 bp region, and (2) LR13943F and LR13392R [36] (hereafter called “Costa primers”), which amplify a ~550 bp region. All amplifications were performed in 25 μL reactions consisting of 2 μL of template DNA, 0.2 mM of each dNTP, 0.5 μM forward primer, 0.5 μM reverse primer, 1.25 U GoTaq Flexi DNA polymerase (Promega), 1.5 mM MgCl_2_, and 1X GoTaq Flexi Buffer (Promega). Amplification reactions began with an initial 2-min dissociation step at 94°C, followed by 35 cycles of 94°C for 1 min, 90 seconds (sec) at the locus-specific annealing temperature (42°C for Costa primers; 46°C for Folmer and Tang primers; 58°C for Clarke primers), and a 2-min extension step at 72°C. A final extension step of 72°C was performed for 10 min. To verify PCR amplification success, 5 μL of PCR product was electrophoresed through a 2% agarose gel stained with ethidium bromide and visualized under UV light. The PCR products were purified for sequencing by the addition of 5 U exonuclease I and 0.5 U shrimp alkaline phosphatase and a subsequent 37°C incubation for 30–45 min. These two enzymes were denatured by a 15 min incubation at 80°C. Sequencing was performed for both directions in 10 μL reactions consisting of 2 μL prepared template, 0.5 μM of either forward or reverse primer, and 0.5 μL BigDye v3.1 (Applied Biosystems) in 1X sequencing buffer. Sequencing reactions were cleaned following the manufacturer’s protocol for ethanol/EDTA/sodium acetate precipitation. Purified sequenced products were run on an AB3500 Genetic Analyzer (Applied Biosystems). The resulting chromatograms were manually edited and assembled using Sequencher 5.0 (Gene Codes).

### Metabarcoding composite bee samples

The four primer pairs used for Sanger sequencing were also tested for metabarcoding of the composite bee samples. Each locus was PCR-amplified in triplicate. This PCR amplified the region of interest and added the Illumina sequencing adaptors using 3 μL of composite DNA diluted to 2 ng/μL as template. This was done in 25 μL reactions with 0.5 μM forward primer+adaptor, 0.5 μM reverse primer+adaptor, 12.5 μL KAPA2G Fast HotStart ReadyMix (KAPA Biosystems), and 0.25 μL BSA (New England Biolabs). Amplification conditions for this PCR were: 95°C for 2 min, then 25 cycles of 95°C for 45 sec, 45 sec at the locus-specific annealing temperatures given above, and 72°C for 90 sec for 25 cycles, with a final 72°C extension phase for 2 min. Triplicate PCRs were pooled per primer pair. To verify amplification, 5 μL of reaction products were visualized on 2% agarose gels stained with ethidium bromide.

The four primer pairs were sequenced in two runs on a MiSeq (Illumina). The first run consisted of the Folmer and Costa primers, and the second run included the Tang and Clarke primers. For each run, equal amounts of the two loci were combined per sample, cleaned with an UltraClean 96 PCR Clean-up kit (Qiagen), and eluted in 30 μL water. Each sample was individually barcoded with Nextera XT dual-index primers (Illumina). The 50 μL dual indexing PCR consisted of 5 μL of cleaned adaptor PCR, 5 μL of Index 1, 5 μL of Index 2, 25 μL of 2xKAPA HiFi HotStart ReadyMix (KAPA Biosystems), and 10 μL PCR-grade water. Amplification conditions were as follows: 95°C for 3 min, then 8 cycles of 95°C for 30 sec, 55°C for 30 sec, and 72°C for 30 sec, with a final extension at 72°C for 5 min. The indexed PCR was cleaned with an UltraClean 96 PCR Clean-up kit (Qiagen) and eluted in 50 μL water. Samples were quantified using a Qubit Broad Range DNA Assay (Life Technologies) and pooled based on their quantification. The pooled sample was quantified using a Qubit Broad Range DNA Assay (Life Technologies) and diluted to a concentration of 2 nM. The final library preps were denatured and diluted to 4 pM, spiked with 15% PhiX control (Illumina), and sequenced on an Illumina MiSeq with either a 300-cycle paired end run (Run 1) or a 250-cycle paired end run (Run 2).

#### Initial evaluation of metabarcode loci

Using bbduk (https://jgi.doe.gov/data-and-tools/software-tools/bbtools/), read pairs were binned by locus by matching forward primer sequences, then trimmed of low-quality bases (Phred-scaled score less than 10) as well as sequences external to the primers (kmer size set to 11). Loci were then processed differently according to expected product size. Read pairs from the Tang primer set were merged with vsearch using a minimum overlap of 80 and a maximum difference of 7.5% in the overlap. Folmer read pairs were not merged as the expected product size was greater than the combined read length, and Costa read pairs were also left unmerged as the expected overlap was too small to reliably merge. Only the forward read was analyzed for the Clarke primer set due to short length of the expected product. Forward reads or merged sequences from each locus were then clustered at 98% using vsearch with divergence measured by edit distance (“iddef” set to 1). The size of each cluster was propagated through each vsearch step, and small clusters (less than 10 sequences) were subsequently dropped within samples to mitigate sample ‘crosstalk’ (i.e., the misassignment of reads to the wrong sample due to index read errors; see [37]). For each locus, the resulting clusters were searched against NCBI’s nucleotide (nt) database, and an initial assessment of taxonomic resolution was performed using a lowest common ancestor (LCA) approach: each cluster was assigned to the LCA in the NCBI taxonomy scheme of all taxa with alignment bit scores within 3% of the top score. A single threshold bit score of 100 was used to filter matches across all loci. For loci analyzed as read pairs, bit scores were summed across matches for each read in the pair as described in Cornman et al. [38].

### Taxonomic assignment of Folmer metabarcode sequences

After the initial evaluation of metabarcoding primer sets, only the Folmer primers were selected for further analysis as the read counts and assessed taxonomic diversity at the other loci were substantially lower. This initial evaluation also revealed some potential *Wolbachia* contamination with the Folmer primers; therefore, the most frequently matched *Wolbachia* accession (JN625842.1) was used as a reference to remove clusters that aligned at 95% identity or greater. As an additional contaminant screen, any Folmer sequence that was not assigned within phylum Arthropoda by Ribosomal Database Project Classifier (RDP) [39] using the CO1v4 training set [40] at a minimum threshold of 0.8 (see below) was also excluded.

At this stage, Folmer read clustering was refined using the amplicon variant method implemented in swarm [41] in which exact low-abundance clusters are grafted to nearby exact high-abundance clusters. Chimeras were flagged using the default settings of the uchime_ref command of vsearch, with the inclusive database (described below) specified as the reference. To allow kmer-based taxonomic assignment methods such as RDP [39] and the Simple Non-Bayesian Taxonomy classifier (SINTAX) [42] to be evaluated for this locus, the representative sequence of each forward-read cluster generated by swarm was scaffolded with its reverse read using vsearch and a linker sequence of 15 N’s [43]. Raw Folmer read pairs are available from NCBI under BioProject PRJNA892648.

A custom reference database for taxonomic analysis was generated based on a checklist of regional bee species: the list of 376 bee species in Iowa was compiled using the DiscoverLife database and information from local experts with vouchered collections (S2 Table). Linnaean species names from the checklist were matched to the corresponding taxonomic IDs in NCBI’s taxonomy database, which can subsequently be used to select or exclude any taxonomically linked sequences in GenBank. Candidate accessions for the database were identified by initially searching the nt database with BLASTN, using default parameters but with low-complexity filtering disabled and a maximum of 25 matches reported. Matched sequences within order Hymenoptera were extracted from the nt database and subset to the coordinates reported by BLAST.

Sequences were also obtained from the Barcode of Life Database (BOLD) [26] on February 10, 2022, using Hymenoptera and United States as initial search criteria. Only CO1 sequences with taxonomies matching the expected species list were retained. Reference sequences were aligned with mafft [44] and then visually oriented and reviewed in BioEdit [45] to remove sequences external to the primers as well as references with excessive numbers of ambiguous characters; the maximum proportion of ambiguous characters after this step was 4%.

Two variations of the custom database were investigated: (1) an inclusive version that used 1,352 GenBank sequences of 115 taxa with a minimum length of 250 bases and (2) a more curated version that used 578 sequences of 81 taxa drawn from both GenBank and BOLD databases, requiring a higher length threshold of 400 bases as well as dereplication by species (S3 Table). Groups of reference sequences with the same taxonomic ID were dereplicated with the “derep_fulllength” command of vsearch. Three taxonomic assignment methodologies were investigated: (1) RDP [39] with the CO1v4 training of Porter and Hajibabei [40], (2) SINTAX [42] implemented in vsearch, and (3) the LCA method using the non-overlapping read method [38]. RDP and SINTAX assignment thresholds were set to 0.8. Both the inclusive and curated databases were formatted for SINTAX by extracting the NCBI taxonomic hierarchy of each accession for standard ranks (phylum, class, order, family, genus, and species). The LCA method was implemented using either the curated database or NCBI’s nt database (download date January 11, 2021), and for the latter database, with or without filtering based on the taxonomic identifiers of the regional bee species. The assigned taxon was the lowest common ancestor of accessions scoring within 3% of the best score (total number of matched bases). To be conservative, species-level assignments with an average percent identity among matching accessions less than 96% were demoted to genus, whereas genus-level assignments with an average percent identity less than 92% were demoted to family.

In total, six distinct workflows were used to generate an aggregate counts table at the genus level, which were then compared to identify the most consistent methods and taxa (S4 Table). The LCA method was performed using BLAST match scores derived three different ways: 1) from searching the full nt database, 2) from searching the full nt database but then subsequently filtering matches to include only accessions of taxa that were on the regional bee list described above, and 3) using only the curated database of NCBI and BOLD sequences. Three kmer-based assignment methods were used: the RDP Classifier [39] with the COIv4 training set available at https://github.com/terrimporter/CO1Classifier/releases, the SINTAX classifier [42] with the NCBI-derived 1,352-sequence database, and the SINTAX classifier with the curated 578-sequence database. For the kmer-based methods, the highest-resolution assignment scoring above 0.8 (RDP) or 0.9 (SINTAX) was chosen. Based on this comparison, we conservatively chose to generate a sample-level counts table for analysis using the LCA method with the custom regional database (S3 Table), as this method did not make any assignments unsupported by the majority of methods. As high-quality genetic sequences are available for most bumblebee species found in Iowa, we used explicitly phylogenetic assessments to determine if metabarcode sequences assigned to *Bombus* could be further binned to species post hoc.

#### Statistical analyses

To determine how frequently metabarcoding and morphological identification detected specific genera, a paired sample t-test was used to compare the number of times each genus was detected in samples. A Wilcoxon rank sum test was used to test if the proportion of bee legs in a composite vial of a given genus affected whether that genus was detected with metabarcoding. Results from the two identification methods were compared to the subset of Sanger sequencing validation results. Comparisons assessed: (1) the frequency that genera were detected through the two methods and validation and (2) the number of genus-level assignments of individuals at the site visit for morphological identification and Sanger sequencing validation results. Five bees could not be morphologically separated between *Heriades*, *Hoplitis*, and *Osmia*, and three bees could not be separated between *Epeolus* and *Triepeolus*; those eight bees were not included in genus-level comparisons. Bee DNA was not detected through metabarcoding in two composite vials of small legs [46] (National Center for Biotechnology Information [NCBI] BioProject: PRJNA892648); these two vials were not included in genus-level comparisons. All statistical tests were completed in Microsoft Excel and R [47].

## Results

Of the four primer pairs tested in this study, only the Folmer primers were selected for analysis because they achieved a high proportion of species assignments (80.7% compared with 28.7% with Clarke primers) and substantially higher taxonomic diversity than the other loci tested during preliminary analyses. Furthermore, *Apis mellifera* constituted 44.1% of all species-level reads with the Clarke primers but only 16.7% with the Folmer primers, suggesting that Folmer primers led to higher amplification of non-honey bee DNA compared with Clarke primers. Additional important considerations were the greater availability of reference sequences for cytochrome oxidase sequences than 16S sequences [48] and the availability of a pre-trained, arthropod-rich assignment method for CO1 to benchmark assignment methods [40]. All results and interpretations reported in this study are for the CO1 region using the Folmer primers.

Bees were captured during 47 of 48 site visits: 1,072 wild bees and 119 honey bees were morphologically identified and submitted for genetic analyses. Morphological identification of wild bees resulted in 22 genera and 36 unique taxa [31]. More than 80% of *Bombus* bees (n = 425) were identified to species by the images; the remaining 20% were either too damaged or could not be distinguished between *B*. *pensylvanicus* and *B*. *auricomus*. Eight bees were not identified to genus using morphology. Metabarcoding identification resulted in genus-level assignments for 18 genera (S5 Table). Amplification did not occur in two vials (1 small vial with 9 legs and 1 small vial with 8 legs); therefore, taxa were not detected using metabarcoding for those vials. Genera not identified with metabarcoding but were morphologically identified included *Coelioxys*, *Epeolus*, *Hylaeus*, *Nomia*, *Peponapis*, and *Triepeolus*. *Sphecodes* (n = 1) was not identified by morphology but was identified through metabarcoding. Some genera (*Hoplitis*, *Heriades*, and *Osmia*) were indistinguishable from the images alone for five bees; however, metabarcoding was able to differentiate those five bees and assign a genus, increasing genera richness at one site visit.

Numerous reference sequences were present in the reference database for Iowa *Bombus* species, but most taxonomic assignments were at the genus level. This indicates that the *Bombus* sequences detected by metabarcoding typically aligned with similar scores to multiple species. In other words, the barcode gap [49] between plausible *Bombus* taxa was not high enough at this locus using this method (3% LCA) to differentiate *Bombus* species routinely.

Morphological identifications and metabarcoding detections agreed in the number of genera and the specific genera present in 44% of the composite vials analyzed. Within the composite vials, a maximum of 6 and 9 genera were identified using DNA metabarcoding and morphological identification, respectively. Among the composite vials comprised of small legs, there were 105 instances when the morphological identification agreed with the metabarcoding identification and 55 instances when identifications misaligned (i.e., the genus was detected in only one of the two methods). There were fewer misalignments (26%) in composite vials with large legs.

**Fig 2:**
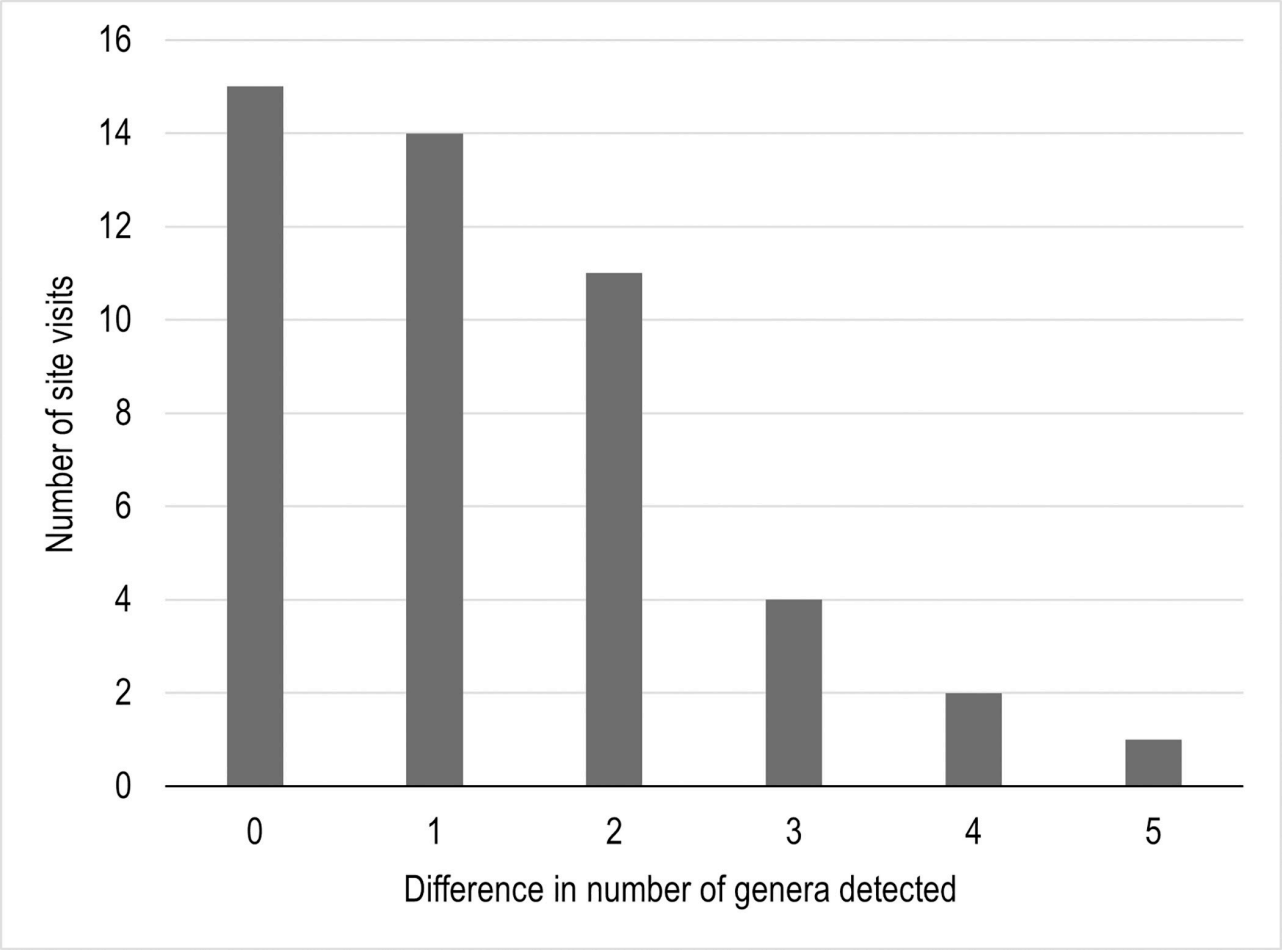
Difference in the number of bee genera detected per site visit with two identification methods (morphological minus metabarcoding). Comparisons were made for 47 site visits where bees were captured. Most site visits included both a large and a small composite vial. Positive values along the x-axis indicated how many additional genera were identified using morphological identification compared to metabarcoding.

Morphological identification yielded higher genera richness at 32 site visits, the same richness at 15 site visits, and never yielded fewer genera compared to the metabarcoding data (Fig 2 and S1 Fig). For site visit comparisons, the number of bee genera detected with the morphological method was always greater than or equal to the number of bee genera detected with metabarcoding. On average, the morphological identification method identified 1.3 more genera than metabarcoding per site visit.

**Fig 3:**
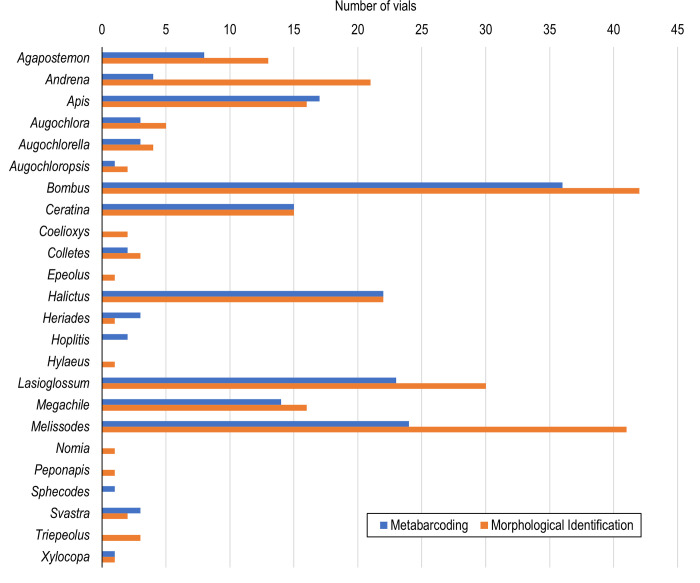
Number of vials in which a bee genus was detected using DNA metabarcoding (blue) and morphological identification (orange). Bees were morphologically identified using images, and a leg from each bee was composited. Vials contained composite bee legs from one or more genera, and genera richness was assessed in each sample that had a genetic detection (n = 84).

As expected based on these results, genera had differing detection rates using morphological identification and metabarcoding methods (Fig 3). Genera-specific detections were more frequent with morphological identification than metabarcoding (t = -2.497; *p* = 0.02). *Bombus* bees were morphologically identified in 42 samples, and the genus was detected with metabarcoding in 86% of the corresponding composite vials. *Lasioglossum* was detected in 72% of the composite vials where *Lasioglossum* was morphologically identified. *Andrena* had the lowest percent detection with metabarcoding compared to morphological identification (19%). Other genera (including *Ceratina* and *Halictus*) were identified with metabarcoding 100% of the time they were identified morphologically.

**Fig 4:**
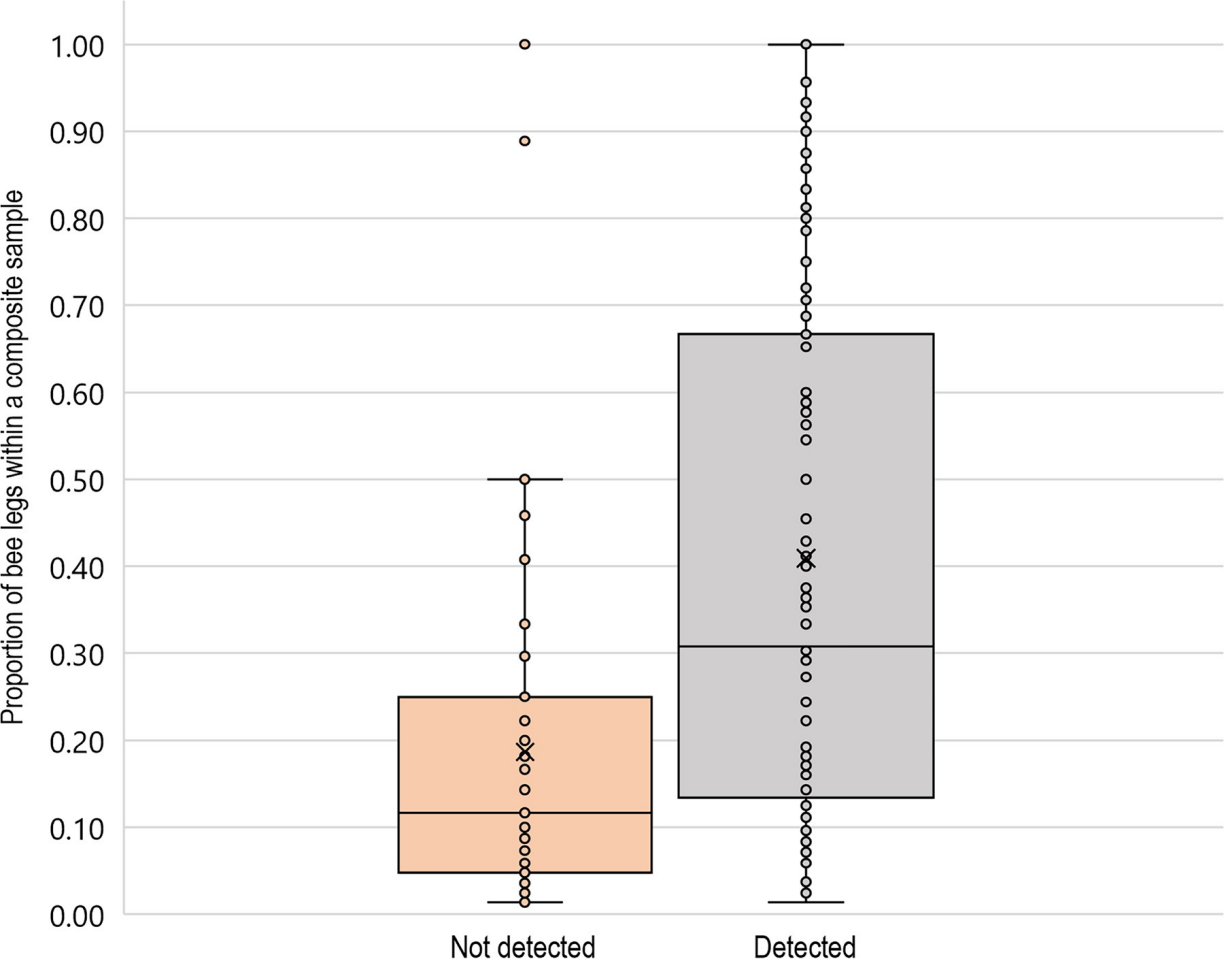
Proportion of bee legs within a composite sample for genera that were detected and not-detected using DNA metabarcoding. Box and whisker plots depict the median **(**horizontal line within the box), mean (x within the box), first quartile (bottom line of box), third quartile (top line of box), minimum value (bottom whisker), and maximum value (top whisker) unless the values are outliers (greater than 1.5 times the interquartile range above the third quartile, shown as points above top whisker).

As the proportion of bee legs per genus was known for each composite vial with genetic detections (n = 84 composite vials), the relationship between this measure of relative abundance in the original sample and frequency of genetic detection could be assessed. The mean proportion of legs per vial for genera that were not detected through metabarcoding was lower than the mean proportion of legs per vial for genera that were detected through metabarcoding (W = 3277; *p* <0.001; Fig 4). This indicated that genera with proportionately less tissue in a given vial were less likely to be detected through metabarcoding. On average, the genera that were detected with metabarcoding represented 41% of the bee legs in a vial. However, proportions ranged from 0.01 to 1.00 for both detections and non-detections. *Apis mellifera* were collected at seven site visits in July and nine site visits in August. The metabarcoding method and primer appeared to be highly sensitive to *Apis* DNA. The proportion of *Apis* legs in large vials ranged from 0.03–0.87, and the *Apis* genus was identified at all sites where those bees had been captured and even one additional site where *Apis* bees were not morphologically identified.

A maximum of 4 genera were not identified in a composite vial using metabarcoding (S2 Fig). One genus was not detected through metabarcoding in the large vial with 73 bee legs; this vial contained mostly *Bombus* legs and 3 morphologically identified genera. Conversely, 4 genera were not detected through metabarcoding in the small vial that contained 72 legs and 9 morphologically identified genera. The composite vials where >2 genera were not identified each had >20 bee legs in the sample.

There were some inconsistencies with both identification methods. For instance, in the composite vial of small legs from site visit A_582, 7 *Melissodes* (*M*. *bimaculatus* [2]; *M*. *desponsus* [2]; and *M*. *trinodis* [3]) were morphologically identified, and the genus was detected through metabarcoding. However, five *Melissodes* (*M*. *bimaculatus* [1]; *M*. *desponsus* [4]) were morphologically identified in the composite vial of large legs, but the genus was not detected through metabarcoding. Conversely, there were eight composite vials where metabarcoding detected a genus that was not identified using morphology.

In this study, 69 individual bee legs were analyzed with Sanger sequencing as a positive control for both morphological analysis and metabarcoding, but 3 individual legs were removed from analyses because both the morphological identification and sequence were unavailable. Of the 66 remaining legs, 46 were assigned to genus or species using the same method as for metabarcode sequences, and 4 were assigned at the family level. Amplification with the Folmer primers was not successful for the other 16 samples; therefore, a sequence could not be generated and thus no taxon assigned. Sanger sequence assignments and morphological identification had species-level agreement for 29 bees, and another 15 bees agreed at the genus level. Sanger sequences gave species-level assignments for 11 bees when morphological identification only assigned genus-level, especially for *Lasioglossum* (Table 1). Examining results from the two genetic techniques showed that metabarcoding was generally validated by the Sanger sequencing.

**Table 1:** Taxonomic assignments for cytochrome *c* oxidase I (CO1) barcode sequences obtained from single specimens compared to their image-based morphological identifications.

Bee ID	Taxid	Sanger sequence taxon	Morphological taxon	Metabarcoding
A-391-1	ND	ND	*Bombus griseocollis*	Y
A-391-2	207634	*Bombus griseocollis*	*Bombus griseocollis*	Y
A-391-3	ND	ND	*Bombus griseocollis*	Y
A-391-4	28643	*Bombus pensylvanicus*	*Bombus pensylvanicus*	Y
A-391-5	ND	ND	*Bombus auricomus*	Y
A-391-6	ND	ND	*Bombus griseocollis*	Y
A-391-7	207634	*Bombus griseocollis*	*Bombus griseocollis*	Y
A-391-8	207634	*Bombus griseocollis*	*Bombus griseocollis*	Y
A-391-9	28643	*Bombus pensylvanicus*	*Bombus pensylvanicus*	Y
A-391-10	309941	*Bombus affinis*	*Bombus affinis*	Y
A-391-11	132113	*Bombus impatiens*	*Bombus impatiens*	Y
A-391-12	7460	*Apis mellifera*	*Apis mellifera*	Y
A-391-13	156364	*Svastra obliqua*	*Svastra obliqua*	Y
A-391-14	156364	*Svastra obliqua*	*Svastra obliqua*	Y
A-391-15	81082	*Melissodes*	*Melissodes*	N
A-391-16	77576	*Halictus ligatus*	*Halictus ligatus*	Y
A-391-17	77576	*Halictus ligatus*	*Halictus ligatus*	Y
A-391-18	1452476	*Megachile brevis*	*Megachile*	Y
A-391-19	1452476	*Megachile brevis*	*Megachile*	Y
A-391-20	77576	*Halictus ligatus*	*Halictus ligatus*	Y
A-466-1	7460	*Apis mellifera*	*Apis mellifera*	Y
A-466-2	ND	ND	*Bombus vagans*	Y
A-466-3	132113	*Bombus impatiens*	*Bombus impatiens*	Y
A-466-4	7458	*Apidae*	*Melissodes bimaculatus*	Y
A-466-5	81082	*Melissodes*	*Melissodes*	Y
A-466-6	ND	ND	*Melissodes trinodis*	Y
A-466-7	77576	*Halictus ligatus*	*Halictus ligatus*	Y
A-466-8	ND	ND	*Lasioglossum*	N
A-825-1	28643	*Bombus pensylvanicus*	*Bombus pensylvanicus*	Y
A-825-2	28643	*Bombus pensylvanicus*	*Bombus pensylvanicus*	Y
A-825-3	207634	*Bombus griseocollis*	*Bombus griseocollis*	Y
A-825-4	586910	*Megachile latimanus*	*Megachile latimanus*	Y
A-825-5	156364	*Svastra obliqua*	*Melissodes*	Y
A-825-6	7458	*Apidae*	*Melissodes bimaculatus*	Y
A-825-7	7458	*Apidae*	*Melissodes bimaculatus*	Y
A-825-8	ND	ND	*Triepeolus*	N
A-825-9	ND	ND	*Triepeolus*	N
A-825-10	ND	ND	*Triepeolus*	N
A-825-11	77572	*Halictidae*	*Augochloropsis metallica* *fulgida*	N
A-825-12	1479665	*Agapostemon splendens*	*Agapostemon*	Y
J-825-1	239130	*Bombus citrinus*	*Bombus citrinus*	Y
J-825-2	ND	ND	*Bombus bimaculatus*	Y
J-825-3	7460	*Apis mellifera*	*Apis mellifera*	Y
J-825-4	7460	*Apis mellifera*	*Apis mellifera*	Y
J-825-5	ND	ND	*Andrena rudbeckiae*	N
J-825-6	599269	*Melissodes communis*	*Melissodes*	Y
J-825-7	ND	ND	*Andrena rudbeckiae*	N
J-825-8	81082	*Melissodes*	*Melissodes trinodis*	Y
J-825-9	81082	*Melissodes*	*Melissodes trinodis*	Y
J-825-11	77576	*Halictus ligatus*	*Halictus ligatus*	Y
J-825-12	77576	*Halictus ligatus*	*Halictus ligatus*	Y
J-825-14	88517	*Lasioglossum pectorale*	*Lasioglossum*	Y
J-825-15	88495	*Lasioglossum pilosum*	*Lasioglossum*	Y
J-825-16	88517	*Lasioglossum pectorale*	*Lasioglossum*	Y
J-880-1	ND	ND	*Andrena rudbeckiae*	Y
J-880-2	ND	ND	*Andrena rudbeckiae*	Y
J-880-3	205204	*Andrena wilkella*	*Andrena*	Y
J-880-5	205204	*Andrena wilkella*	*Andrena rudbeckiae*	Y
J-880-6	77576	*Halictus ligatus*	*Halictus ligatus*	Y
J-880-7	77576	*Halictus ligatus*	*Halictus ligatus*	Y
J-880-8	77576	*Halictus ligatus*	*Halictus ligatus*	Y
J-880-9	77576	*Halictus ligatus*	*Halictus ligatus*	Y
J-880-10	88495	*Lasioglossum pilosum*	*Lasioglossum*	Y
J-880-11	1038993	*Lasioglossum albipenne*	*Lasioglossum*	Y
J-880-12	ND	ND	*Lasioglossum*	Y
J-880-13	1040006	*Lasioglossum paradmirandum*	*Lasioglossum*	Y

Sanger sequence taxonomic assignments were based on the lowest common ancestor method using alignment bit score as the scoring metric. The "Taxid" is the unique taxonomic identifier used in the taxonomy database of the National Center for Biotechnology Information. Gray cells indicate species-level agreement, yellow cells indicate genus-level agreement, and red cells indicate disagreement. ND indicates that amplification at the target locus failed. Letters in the Metabarcoding column indicate whether the genus was detected (Y) or not detected (N) through metabarcoding of the corresponding composite sample.

## Discussion

In this study, image-based morphological identification and metabarcoding approaches were successfully used to identify wild bees collected from the field. However, most bee studies will likely rely on only one identification method (morphological or genetic sequencing), and results from this study show the strengths and weaknesses of each method for wild bees. Specifically, we found that DNA metabarcoding is quicker and often less expensive (when considering the time needed for morphological identification) than morphological identification, but additional data, such as abundance, gender, lower taxonomic resolution (in some cases), and positive identification of all taxa (i.e., even those that might not amplify), can be gained from the morphological method (Table 2).

**Table 2:** Advantages and disadvantages of image-based morphological identification and DNA metabarcoding for wild bee identification.

Potential advantages	Potential disadvantages
Image-based morphological identification	
•Nondestructive to specimen	•Requires taxonomic expertise
•Preserves specimen	•Time intensive (4 months in this study)
•Can determine richness and abundance for diversity indices	•Difficulty separating morphologically similar genera
•Possible to determine gender with some genera	•Damaged specimens may make ID more difficult
•Could identify to species in some cases •Photographs can be archived and revisited	•Equipment needed (minimum caliber microscope and camera)
	•No ability to quantify uncertainty in species identification
DNA metabarcoding	
•Partially destructive to specimen	•Requires genetic expertise
•Can resolve morphologically cryptic genera	•Can miss taxa that do not amplify in a sample
•Fast results (2–4 weeks)	•Inability to confidently assign to species level
•Specimens damaged from capture can be used	•Cannot determine gender
•Provides presence data	•Subject to primer specificity issues
•High confidence in detections •Low cost (~$6 per sample)	•Requires access to lab equipment, library preparation capability, and bioinformatics
	•Relies on publicly sourced GenBank and BOLD which is lacking sequences for some species

Due to the objectives of the larger study of which this work was a part (i.e., the need to preserve the bee tissues for additional analyses; [28,29]), the specimens had to be frozen immediately after field collection and could not be pinned or examined under a microscope. Photographing and assigning the lowest taxonomic unit using dichotomous keys and expert advice was moderately time intensive (~4 months for this study); however, the processing steps (e.g., photographing and sorting by potential taxa) completed by an amateur taxonomist greatly reduced the amount of time needed from an expert taxonomist compared to the traditional pinning identification method. Factors including ice crystals, pollen, color saturation, magnification, and specimen damage during freezing obscured some diagnostic features, preventing identification to species in many cases. Morphological identification resulted in species-level assignments for 65% of all bees and >80% of *Bombus* bees. Additional information, including the intertegular distance (which is correlated to mass and foraging distance; [50,51]) and bee gender for sexually dimorphic species, could be obtained from the images, and the images provided a publicly available reference database [31]. Richness and abundance data from the morphological identification method can also be used to calculate diversity indices (see [29]), which would not be possible with the richness-only results from metabarcoding.

Identification through DNA metabarcoding relies on comparison to sequences in currently accessible databases [12,13,23,52], which is lacking for some taxa. Most sequences can currently be attributed to genus level; however, assignments will improve as additional sequences are added to public databases (S2 Table). The six genera of wild bees not identified with metabarcoding were uncommon and only accounted for 17 bees photographed in the study. However, *Coelioxys*, *Epeolus*, and *Triepeolus* genera may be disproportionately important to identify in ecological studies because they are kleptoparasitic (or brood parasitic) bees that indicate parasite-host interactions are occurring [53]. Few *Epeolus* (n = 3) and *Coelioxys* (n = 1) sequences were available in GenBank for the regional bee species of Iowa, and no sequences were available for *Triepeolus*, showing a bias in GenBank for more common bees. Even if high-quality sequences are available for a group of interest (e.g., *Bombus*), species separation may only be attainable by targeting multiple loci [12]. Targeting sequences in bees and other hymenopterans through metabarcoding can be challenging because of their lower affinity to universal CO1 primers [54]. Future metabarcoding analyses could be improved by *in silico* design of primers for various subclades of bees from full mitogenomes and tested on mixtures and through the optimization of primers used to amplify the CO1 gene [22].

Of the four common primer sets tested in this study, the Folmer primers worked best for metabarcoding, resulting in many long reads that could be robustly assigned to genus. Well-performing primers, however, are only useful if amplification bias is tolerable and adequate reference sequences are available, emphasizing the value of reference collections and whole mitogenome sequencing (e.g., The Beenome100 Project) to establish primer-agnostic databases (in contrast to BOLD, for instance). Recent genomic bee studies have shown that metabarcoding composite samples can produce similar results as morphological identification [11,12]. Both studies used expert identification of bees using traditional methods to test the utility of metabarcoding using either 28S [12] or the entire mitochondrial genome [11]. Similar to the current study, results from composite samples provided general agreement with expert taxonomy, and both were able to assign species level identification in many cases.

An important consideration for this and future studies is that the identification methods were conservative in terms of tissue destruction and preservation, ensuring the integrity of the bee tissue for use in companion analyses (e.g., pesticide and/or microbial analyses). While these identification methods would allow multiple types of information to be collected from each bee, the bees were still sacrificed for the study. Future identification methods that do not require sacrificing the subjects may include photographing in the field [14,19,20] or bee eDNA collection in air [55], soil [56], or on flowers [57]. Our study finds that using image-based morphological identification and metabarcoding identification techniques together provide more overall information on wild bee diversity with higher degrees of confidence. These techniques can be transferred to the field by pairing camera traps and eDNA metabarcoding [57] to provide diversity data without having to capture and sacrifice wild bees. Thus, the results from this study can help inform and guide best practices for bee identification for future research.

Compared to Sanger sequencing of individual bees, using metabarcoding in composite vials of bee legs vastly reduced the number of samples to be analyzed. Some genera were not detected possibly due to primers not working well on certain species, competition with other DNA templates in the PCR, or DNA extraction issues (i.e., not enough DNA from a certain bee leg in the composite sample, insufficient homogenization of composite samples, too many bee legs in a composite sample). Results from this study indicate that the proportion of bee legs from a genus in the vial likely affected the probability of that genus being detected. DNA metabarcoding was most effective at identifying more common species that comprised 41% or more of the sample. Refinements such as lowering the number of legs in a composite sample, better composite homogenization, and obtaining vouchered reference sequences for rare species may need to be implemented to identify less common species. DNA metabarcoding was able to refine morphological identifications in several instances, especially for morphologically cryptic taxa, but metabarcoding did not always detect all the genera that were obtained through the morphological method.

Analyses of individual bee legs using Sanger sequencing further illustrate challenges of genetic taxon assignments. Amplification failed in approximately 25% of the samples, suggesting that PCR inhibition may have been important in some samples. The rigorous methodology for assigning a genetic taxon, however, confers a high degree of confidence to a final identification. *Melissodes* voucher sequences were sometimes downgraded to taxonomic family with Sanger sequencing (Table 1), most likely because *Melissodes* sequences in the database were shorter than reference sequences for other taxa and, therefore, did not generate higher alignment scores. If the Sanger sequence is not assigned to a known source, it is likely that similar taxonomic errors will occur in the metabarcode data.

Future efforts could create voucher specimens by identifying the bees with the traditional morphological approach and then adding their reference sequences to genetic databases [25,27]. This is especially important for rare and underrepresented wild bee species. Our results have shown that analyses of individual bee legs can provide species-level information and may be the best approach when studying morphologically indiscernible genera such as *Lasioglossum*. Analyses of individual bees provided some species-level assignments but, similar to the metabarcoding, Sanger sequencing was limited by the sequences available in the database and general primer and amplification variability. An upper threshold for the number of bee legs (either large or small) that should be included in a composite sample may be around 20 (S2 Fig), but it is currently unclear if there is a threshold regarding the number of genera that can consistently be identified in a composite sample. It can be surmised from the results in this study that a lower proportion of material in a vial may result in reduced genetic detections.

Processing steps before, during, and after genetic analyses may be refined to yield greater genus-level detections using molecular techniques. Because of the sensitivity of the metabarcoding method to *Apis mellifera* and the potential effects of interference, removing *Apis* bees from composite vials may increase the detections of wild bee genera. Similarly, removing other easily identifiable genera (such as *Bombus*) may yield greater detection of morphologically cryptic species using composite genetic analyses. During analyses, there may be specific primers [22,58], amplification methodology, and loci [12] that can be used to target genera of interest. In this study, small clusters of less than 10 sequences were censored as a conservative threshold for removing potential crosstalk contamination. There were five instances when sequence counts were 10 or slightly greater for genera such as *Lasioglossum* and *Agapostemon*. However, sequence counts were generally high for genera including *Augochlora*, *Ceratina*, and *Bombus*. Censoring steps may need to be adjusted to allow low counts for rare bee genera, although it is unclear how to best model crosstalk rates empirically [37].

The methods that were tested in this study show great promise in identification of wild bees without relying on substantial time investments from taxonomic experts, which can delay study results. The choice of identification method will likely be related to study objectives, budget, and focal general. Morphological identification with images is particularly helpful in situations where the specimen must be preserved for other analyses (e.g., pesticides; [28,59]) and when accurate abundance or gender information are required. The morphological method was able to identify most bees to species level, yet the uncertainty surrounding these taxonomic assessments could not be quantified. The metabarcoding approach is faster and inexpensive with high confidence in detections, yet metabarcoding lacked the ability to make species-level determinations with confidence and sometimes missed species that were likely present. Additional refinement of metabarcoding methods (e.g., optimized methods for DNA extraction of composite samples and library preparation) would likely overcome some of these obstacles, as will the continual addition of new sequences from voucher specimens to public DNA sequence repositories. Importantly, these methods complimented each other, showing general agreement of identification to genus for most samples, further highlighting the utility of both approaches.

## Supporting information

S1 Fig The difference in the number of bee genera detected with two identification methods (image-based morphological identification and DNA metabarcoding) in composite vials from site visits in July ("J") and August ("A"). Site visits without bars show that the number of bee genera detected with the two identification methods was the same. Positive values along the x-axis indicate more genera were detected by morphological identification than metabarcoding. Data were used to create Fig 2.
(TIF)

S2 Fig Comparing the number of wild bee genera not detected through metabarcoding to the number of bee legs in the composite sample. Composite samples included tissue from multiple taxa. Composite samples either contained legs from large bees (intertegular distance >3 mm) or small bees (intertegular distance ≤3 mm).
(TIF)

S1 Table Primer pairs tested on samples of wild bee DNA. (PDF)

S2 Table List of regional bee species for eastern Iowa, USA. The comprehensive list of possible species was developed by combining species included in the geographic region in DiscoverLife (DL) with species identified by local experts (LE: Steve Hendrix [University of Iowa] and Mary Harris [Iowa State University]); some species were listed by both DL and LE. The list likely excludes species in eastern Iowa and includes species not found in eastern Iowa. A curated database of sequences was used for this study, and the "Counts" column shows the number of sequences from each species present in the curated database.
(PDF)

S3 Table Curated database used for taxonomic assignment of cytochrome *c* oxidase I (CO1) metabarcode sequences, in FASTA format. Reference headers include the source accession from which the sequence was extracted and the taxonomy of the sequence according to the NCBI Taxonomy resource. The headers are formatted for compatibility with the program Sintax. The curated database was generated from both GenBank and Barcodes of Life databases, with a threshold of 400 bases.
(PDF)

S4 Table Aggregate sequence counts for bee genera from six workflows. Some workflows were restricted by the regional bee list while others were unrestricted. The full nucleotide (nt) database was searched, and then matches were "filtered" to only include accessions of taxa included on the regional bee list. Two custom databases were assessed: 1) an inclusive version generated from GenBank with a minimum length of 250 bases and 2) a curated version that was generated from both GenBank and Barcodes of Life databases, with a threshold of 400 bases and dereplicated within species. The final counts table used in this study was derived from the lowest common ancestor (LCA) method with the curated database. The RDP Classifier (Wang et al., 2007) is a Bayesian kmer-based classifier that requires prior training, for which the CO1 v.5 database of Porter and Hajibabaei (2018) was used. SINTAX (Edgar, 2016) is an alternative kmer-based classifier that does not require an external training set.
(PDF)

S5 Table Final counts table of individual metabarcode sequences that are attributed to each detected bee genus within each vial. All clusters with the same taxonomic assignment were summed within samples to generate the counts table. Vial codes include the month the bees were collected (July [J] or August [A]), a three-digit identifier for the collection site, and whether the vial contained legs from small (S) or large (L) bees.
(PDF)
