# Supplementary figures and images for: Comparing modern identification methods for wild bees: Metabarcoding and image-based morphological taxonomic assignment
Source: PLoS One. 2024 Apr 2;19(4):e0301474. doi: 10.1371/journal.pone.0301474 (PMC10986983; doi:10.1371/journal.pone.0301474)

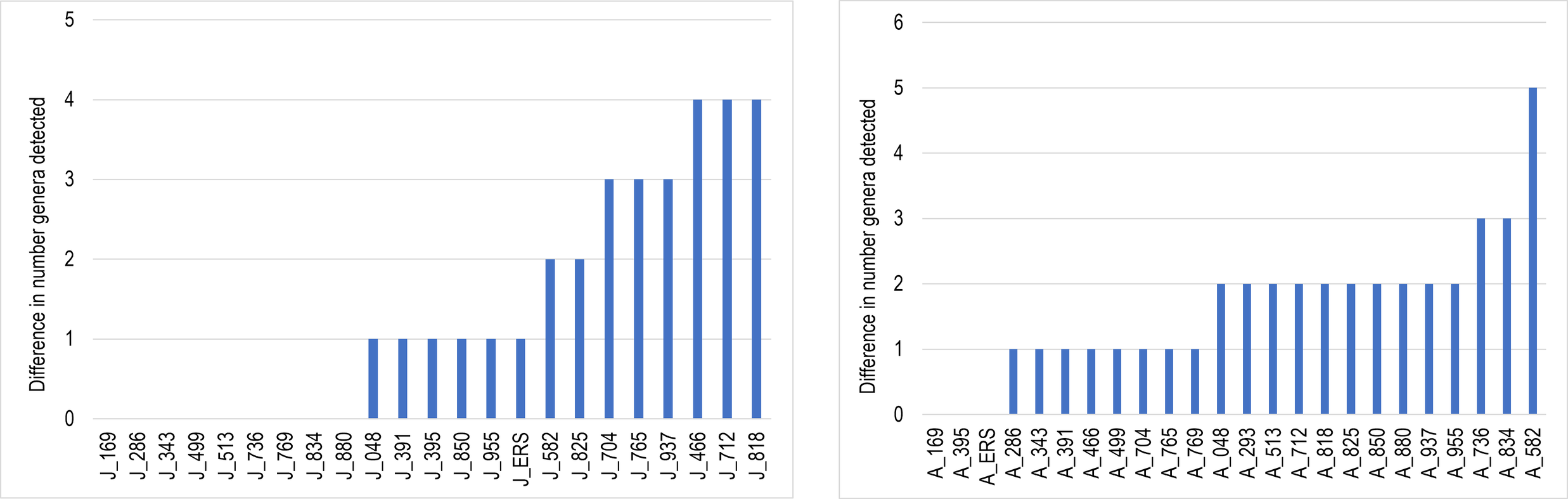

Supplement: S1 Fig — Site visits without bars show that the number of bee genera detected with the two identification methods was the same. Positive values along the x-axis indicate more genera were detected by morphological identification than metabarcoding. Data were used to create Fig 2. (TIF) [file pone.0301474.s001.tif]

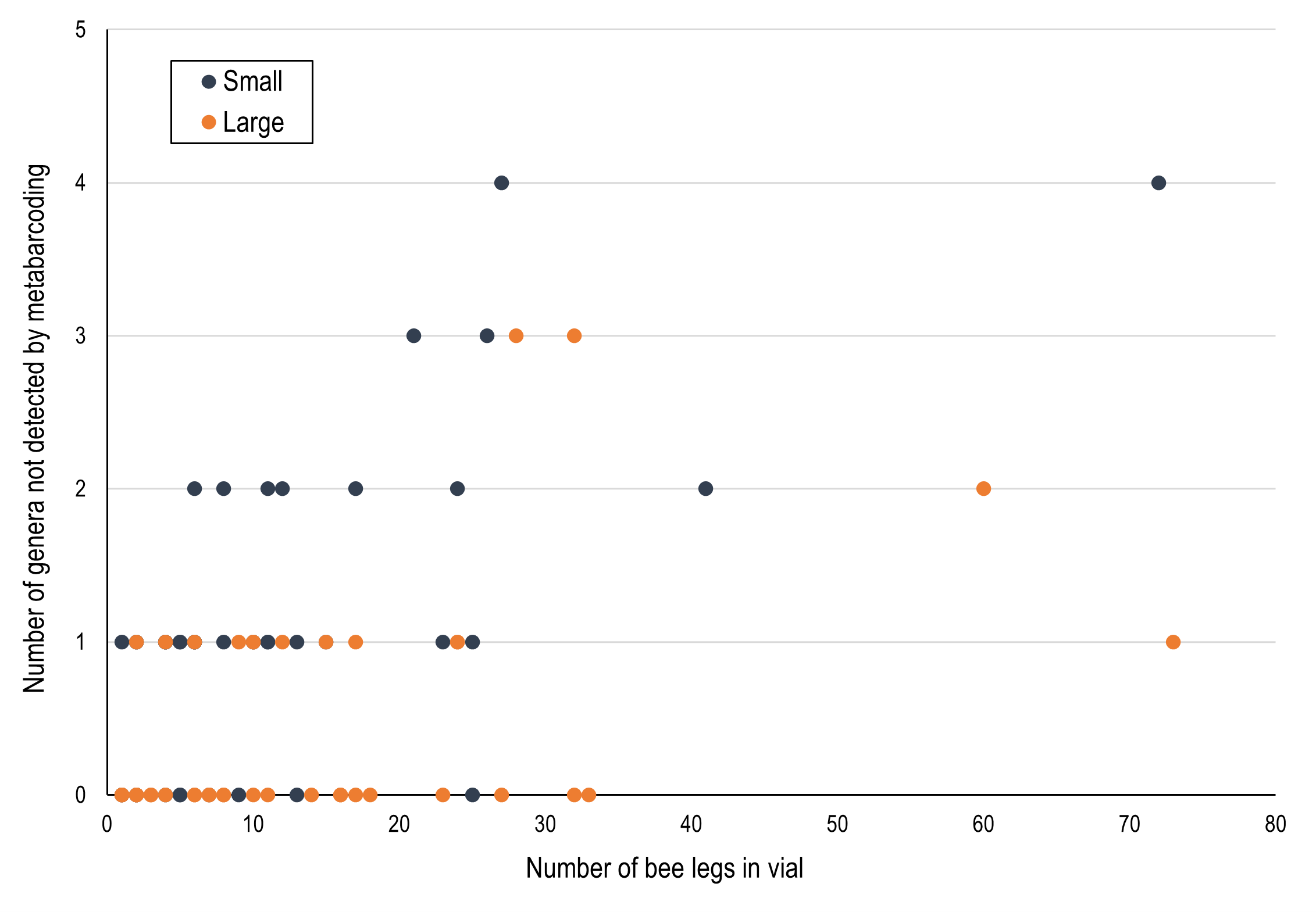

Supplement: S2 Fig — Composite samples included tissue from multiple taxa. Composite samples either contained legs from large bees (intertegular distance >3 mm) or small bees (intertegular distance ≤3 mm). (TIF) [file pone.0301474.s002.tif]
