# Supplementary material for: Comparing modern identification methods for wild bees: Metabarcoding and image-based morphological taxonomic assignment
Source: PLoS One. 2024 Apr 2;19(4):e0301474. doi: 10.1371/journal.pone.0301474 (PMC10986983; doi:10.1371/journal.pone.0301474)
Supplement: S1 Table — (PDF) [file pone.0301474.s003.pdf]

S1 Table. Primer pairs tested on samples of wild bee DNA.

Primer name and citation	Primer pair	Sequence targeted
Folmer primers (Folmer et al., 1994)	LCO1490	5'-GGTCAACAAATCATAAAGATATTGG-3'
	HCO2198	5'-TAAACTTCAGGGTGACCAAAAAATCA-3'
Tang primers (Tang et al., 2015)	LepF	5'-ATTCAACCAATCATAAAGATATTGG-3'
	mlCO1intBeeR	5'-GGDGGRTAWANDGTTCANCCHGTHCC-3'
Clarke primers (Clarke et al., 2014)	Ins16S_F1short	5'-TRRGACGAGAAGACCCTATA-3'
	Ins16S_R1short	5'-ACGCTGTTATCCCTAAGGTA-3'
Costa primers (Costa et al., 2003)	LR13943F	5'-CACCTGTTTATCAAAAACAT-3'
	LR13392R	5'-CGTCGATTTGAACTCAAATC-3'
