# Supplementary material for: Comparing modern identification methods for wild bees: Metabarcoding and image-based morphological taxonomic assignment
Source: PLoS One. 2024 Apr 2;19(4):e0301474. doi: 10.1371/journal.pone.0301474 (PMC10986983; doi:10.1371/journal.pone.0301474)
Supplement: S2 Table — The comprehensive list of possible species was developed by combining species included in the geographic region in DiscoverLife (DL) with species identified by local experts (LE: Steve Hendrix [University of Iowa] and Mary Harris [Iowa State University]); some species were listed by both DL and LE. The list likely excludes species in eastern Iowa and includes species not found in eastern Iowa. A curated database of sequences was used for this study, and the "Counts" column shows the number of sequences from each species present in the curated database. (PDF) [file pone.0301474.s004.pdf]

S2 Table. List of regional bee species for eastern Iowa, USA. The comprehensive list of possible species was developed by combining species included in the geographic region in DiscoverLife (DL) with species identified by local experts (LE: Steve Hendrix [University of Iowa] and Mary Harris [Iowa State University]); some species were listed by both DL and LE. The list likely excludes species in eastern Iowa and includes species not found in eastern Iowa. A curated database of sequences was used for this study, and the "Counts" column shows the number of sequences from each species present in the curated database.

Taxa	Source	Counts
Agapostemon angelicus	Both	0
Agapostemon sericeus	Both	0
Agapostemon splendens	Both	0
Agapostemon texanus	Both	5
Agapostemon virescens	Both	0
Andrena accepta	Both	0
Andrena aliciae	Both	0
Andrena alleghaniensis	LE	0
Andrena andrenoides	Both	0
Andrena asteris	Both	0
Andrena barbara	LE	0
Andrena barbilabris	Both	0
Andrena brevipalpis	Both	0
Andrena canadensis	DL	0
Andrena carlini	Both	0
Andrena ceanothi	LE	0
Andrena chromotricha	DL	0
Andrena commoda	Both	0
Andrena crataegi	Both	0
Andrena cressonii	Both	0
Andrena erigeniae	Both	0
Andrena erythrogaster	Both	0
Andrena erythronii	Both	0
Andrena forbesii	Both	0
Andrena fragilis	Both	0
Andrena frigida	Both	0
Andrena geranii	Both	0
Andrena helianthi	Both	0
Andrena helianthiformis	LE	0
Andrena hippotes	Both	0
Andrena hirticincta	Both	0
Andrena illini	Both	0
Andrena illinoiensis	Both	0
Andrena imitatrix	Both	0
Andrena integra	LE	0
Andrena irrasus	LE	0
Andrena lupinorum	DL	0
Andrena mandibularis	Both	0
Andrena mariae	Both	0

Andrena melanochroa	LE	0
Andrena milwaukeensis	Both	0
Andrena miranda	LE	0
Andrena miserabilis	Both	0
Andrena nasonii	Both	0
Andrena nigrae	Both	0
Andrena nivalis	Both	0
Andrena nubecula	DL	0
Andrena nuda	Both	0
Andrena perplexa	Both	0
Andrena persimulata	LE	0
Andrena personata	LE	0
Andrena placata	LE	0
Andrena platyparia	Both	0
Andrena polemonii	Both	0
Andrena quintilis	Both	0
Andrena robertsonii	LE	0
Andrena rudbeckiae	Both	0
Andrena rufosignata	LE	0
Andrena rugosa	Both	0
Andrena salictaria	Both	0
Andrena sayi	LE	0
Andrena sigmundi	DL	0
Andrena simplex	Both	0
Andrena spiraeana	Both	0
Andrena tridens	Both	1
Andrena vicina	DL	0
Andrena vicina	LE	0
Andrena violae	Both	0
Andrena wilkella	Both	0
Andrena wilmattae	Both	0
Andrena w-scripta	Both	0
Andrena ziziae	Both	0
Anthidiellum notatum	Both	0
Anthidium manicatum	Both	1
Anthidium oblongatum	DL	0
Anthophora abrupta	DL	0
Anthophora bomboides	LE	0
Anthophora occidentalis	DL	0
Anthophora terminalis	Both	0
Anthophora walshii	Both	0
Apis mellifera	DL	19
Ashmeadiella bucconis	LE	0
Augochlora pura	Both	0
Augochlorella aurata	Both	0
Augochlorella persimilis	Both	0

Augochloropsis metallica	Both	0
Bombus affinis	Both	2
Bombus auricomus	Both	1
Bombus bimaculatus	Both	11
Bombus borealis	LE	0
Bombus citrinus	Both	13
Bombus fervidus	Both	16
Bombus fraternus	Both	0
Bombus frigidus	LE	0
Bombus griseocollis	Both	0
Bombus impatiens	Both	13
Bombus morrisoni	LE	0
Bombus pensylvanicus	Both	2
Bombus rufocinctus	LE	33
Bombus ternarius	LE	1
Bombus vagans	Both	19
Bombus variabilis	Both	0
Calliopsis andreniformis	Both	0
Calliopsis coloradensis	DL	0
Calliopsis nebraskensis	Both	0
Ceratina calcarata	Both	40
Ceratina dupla	LE	39
Ceratina mikmaqi	LE	0
Ceratina strenua	Both	10
Coelioxys alternatus	DL	0
Coelioxys germana	LE	0
Coelioxys germanus	DL	0
Coelioxys modesta	LE	0
Coelioxys octodentata	LE	1
Coelioxys octodentatus	DL	0
Coelioxys porterae	LE	0
Coelioxys rufitarsis	LE	0
Coelioxys sayi	Both	0
Colletes aberrans	DL	0
Colletes americanus	DL	0
Colletes brevicornis	LE	0
Colletes ciliatus	DL	0
Colletes compactus	DL	0
Colletes eulophi	DL	1
Colletes howardi	LE	0
Colletes hyalinus	LE	2
Colletes inaequalis	Both	0
Colletes kincaidii	DL	2
Colletes latitarsis	Both	1
Colletes phaceliae	DL	1
Colletes robertsonii	Both	0

Colletes simulans	Both	0
Colletes solidaginis	DL	0
Colletes susannae	Both	0
Colletes wilmattae	DL	0
Diadasia enavata	LE	0
Dieunomia heteropoda	DL	0
Dieunomia nevadensis	LE	0
Dieunomia triangulifera	DL	0
Dufourea marginata	Both	0
Dufourea monardae	LE	0
Dufourea novaeangliae	LE	0
Epeolus ainsliei	DL	2
Epeolus bifasciatus	Both	0
Epeolus interruptus	DL	0
Epeolus lectoides	LE	1
Epeolus minimus	DL	0
Eucera atriventris	Both	0
Eucera dubitata	DL	0
Eucera hamata	Both	1
Florilegus condignus	LE	1
Halictus confusus	Both	0
Halictus ligatus	Both	0
Halictus parallelus	Both	0
Halictus rubicundus	Both	24
Halictus tripartitus	LE	0
Heriades carinata	DL	0
Heriades carinatus	LE	2
Heriades variolosa	DL	0
Heriades variolosus	LE	3
Holcopasites calliopsidis	Both	1
Holcopasites haematurus	DL	0
Holcopasites heliopsis	Both	0
Holcopasites stevensi	LE	0
Hoplitis pilosifrons	LE	3
Hoplitis producta	LE	1
Hoplitis spoliata	LE	1
Hoplitis truncata	LE	0
Hylaeus affinis	Both	0
Hylaeus annulatus	LE	17
Hylaeus fedorica	LE	0
Hylaeus illinoensis	LE	0
Hylaeus leptocephalus	Both	0
Hylaeus mesillae	Both	0
Hylaeus modestus	Both	0
Hylaeus nelumbonis	LE	0
Lasioglossum abanci	LE	0

Lasioglossum acuminatum	LE	0
Lasioglossum admirandum	LE	1
Lasioglossum albipenne	Both	2
Lasioglossum anomalum	Both	7
Lasioglossum athabascense	LE	0
Lasioglossum birkmanni	DL	0
Lasioglossum bruneri	Both	5
Lasioglossum cattellae	Both	0
Lasioglossum cephalotes	DL	0
Lasioglossum cinctipes	Both	0
Lasioglossum coeruleum	LE	0
Lasioglossum coreopsis	LE	3
Lasioglossum coriaceum	Both	0
Lasioglossum cressonii	Both	19
Lasioglossum divergens	LE	0
Lasioglossum dreisbachi	LE	2
Lasioglossum ephialtum	DL	1
Lasioglossum fedorense	LE	0
Lasioglossum forbesii	LE	0
Lasioglossum fuscipenne	LE	0
Lasioglossum hartii	LE	2
Lasioglossum hitchensi	Both	2
Lasioglossum illinoense	Both	1
Lasioglossum imitatum	Both	18
Lasioglossum insolitum	DL	0
Lasioglossum katherineae	DL	1
Lasioglossum laevissimum	LE	10
Lasioglossum leucozonium	Both	1
Lasioglossum lineatulum	Both	15
Lasioglossum lionotus	DL	0
Lasioglossum macoupinense	Both	0
Lasioglossum mitchelli	LE	1
Lasioglossum nigroviride	LE	7
Lasioglossum nulumbonis	LE	0
Lasioglossum nymphaearum	LE	1
Lasioglossum oblongum	LE	2
Lasioglossum obscurum	Both	1
Lasioglossum oceanicum	DL	0
Lasioglossum oenotherae	LE	0
Lasioglossum paradmirandum	Both	3
Lasioglossum paraforbesii	Both	0
Lasioglossum pectinatum	LE	0
Lasioglossum pectorale	Both	14
Lasioglossum pictum	Both	9
Lasioglossum pilosum	LE	2
Lasioglossum platyparium	Both	2

Lasioglossum pruinosum	Both	3
Lasioglossum rohweri	LE	0
Lasioglossum semicaeruleum	Both	12
Lasioglossum simplex	DL	1
Lasioglossum tegulare	LE	1
Lasioglossum tenax	LE	3
Lasioglossum texanum	DL	0
Lasioglossum trigeminum	LE	6
Lasioglossum truncatum	Both	2
Lasioglossum versans	DL	0
Lasioglossum versatum	Both	10
Lasioglossum vierecki	Both	3
Lasioglossum viridatum	LE	0
Lasioglossum weemsi	LE	24
Lasioglossum zephyrum	LE	1
Lasioglossum zephyrus	DL	0
Lasioglossum zonulum	Both	0
Macropis nuda	LE	0
Macropis patellata	DL	0
Macropis steironematis	DL	0
Megachile addenda	LE	0
Megachile albitarsis	LE	0
Megachile brevis	Both	0
Megachile campanulae	Both	0
Megachile centuncularis	DL	0
Megachile dakotensis	DL	0
Megachile exilis	LE	0
Megachile fortis	DL	0
Megachile frigida	LE	0
Megachile frugalis	LE	0
Megachile gemula	Both	4
Megachile georgica	LE	0
Megachile inermis	LE	0
Megachile inimica	Both	0
Megachile latimanus	Both	0
Megachile mendica	Both	0
Megachile montivaga	LE	0
Megachile parallela	DL	0
Megachile perihirta	DL	0
Megachile petulans	Both	0
Megachile pugnata	Both	3
Megachile relativa	DL	0
Megachile rotundata	LE	15
Megachile rugifrons	DL	0
Megachile sculpturalis	DL	0
Megachile texana	Both	0

Melissodes agilis	Both	1
Melissodes bidentis	DL	0
Melissodes bimaculatus	Both	0
Melissodes boltoniae	DL	0
Melissodes coloradensis	LE	0
Melissodes communis	Both	1
Melissodes comptoides	Both	0
Melissodes coreopsis	Both	0
Melissodes denticulata	LE	0
Melissodes denticulatus	DL	0
Melissodes dentiventris	LE	0
Melissodes desponsus	DL	0
Melissodes druriella	LE	9
Melissodes druriellus	DL	0
Melissodes menuachus	Both	0
Melissodes nivea	LE	0
Melissodes perlusus	DL	0
Melissodes subagilis	DL	0
Melissodes subillata	LE	0
Melissodes tepaneca	LE	0
Melissodes trinodis	Both	0
Melissodes vernoniae	Both	0
Melissodes wheeleri	LE	0
Nomada articulata	Both	0
Nomada australis	LE	0
Nomada banksi	DL	0
Nomada bella bella	LE	0
Nomada bethunei	DL	0
Nomada cressonii	LE	0
Nomada cuneata	LE	0
Nomada dentariae	DL	0
Nomada denticulata	LE	0
Nomada depressa	Both	0
Nomada erigeronis	LE	0
Nomada hemphilli	LE	0
Nomada hydrophylli	LE	0
Nomada illinoiensis	LE	0
Nomada imbricata	LE	0
Nomada integerrima	LE	0
Nomada lepida	LE	0
Nomada louisianae	LE	0
Nomada maculata	DL	0
Nomada obliterata	Both	0
Nomada parva	Both	0
Nomada perplexa	LE	0
Nomada pygmaea	DL	1

Nomada rubicunda	LE	0
Nomada sayi	LE	0
Nomada superba	Both	0
Nomada vegana	LE	0
Nomada vincta	Both	0
Nomia maneei	LE	0
Nomia universitatis	Both	0
Osmia atriventris	Both	0
Osmia bucephala	Both	0
Osmia collinsiae	LE	0
Osmia conjuncta	DL	0
Osmia cordata	LE	0
Osmia cornifrons	DL	0
Osmia distincta	LE	0
Osmia inermis	LE	0
Osmia lignaria	Both	0
Osmia michiganensis	LE	0
Osmia pumila	LE	0
Osmia simillima	Both	0
Paranthidium jugatorium	DL	0
Peponapis pruinosa	Both	0
Perdita ainsliei	DL	0
Perdita albipennis	LE	0
Perdita halictoides	Both	0
Perdita maculigera	DL	0
Perdita octomaculata	Both	0
Protandrena bancrofti	Both	0
Protandrena cockerelli	DL	0
Protandrena illinoiensis	LE	0
Pseudopanurgus aestivalis	LE	0
Pseudopanurgus albitarsis	LE	0
Pseudopanurgus andrenoides	LE	0
Pseudopanurgus compositarum	DL	0
Pseudopanurgus labrosus	LE	0
Pseudopanurgus rudbeckiae	DL	0
Sphecodes banksii	LE	0
Sphecodes confertus	LE	0
Sphecodes davisii	DL	0
Sphecodes dichrous	LE	0
Sphecodes heraclei	LE	0
Sphecodes persimilis	LE	0
Sphecodes pycnanthemi	LE	0
Stelis coarctatus	DL	0
Stelis lateralis	Both	0
Stelis subemarginata	LE	0
Stelis vernalis	LE	0

Svastra compta	LE	0
Svastra cressonii	DL	0
Svastra obliqua	Both	1
Tetraloniella albata	Both	0
Triepeolus concavus	LE	0
Triepeolus cressonii	LE	0
Triepeolus distinctus	DL	0
Triepeolus donatus	Both	0
Triepeolus helianthi	LE	0
Triepeolus lunatus	Both	0
Triepeolus remigatus	DL	0
Triepeolus simplex	Both	0
Xenoglossa kansensis	DL	0
Xenoglossa strenua	DL	0
Xylocopa virginica	Both	54
