# Supplementary material for: Comparing modern identification methods for wild bees: Metabarcoding and image-based morphological taxonomic assignment
Source: PLoS One. 2024 Apr 2;19(4):e0301474. doi: 10.1371/journal.pone.0301474 (PMC10986983; doi:10.1371/journal.pone.0301474)
Supplement: S3 Table — Reference headers include the source accession from which the sequence was extracted and the taxonomy of the sequence according to the NCBI Taxonomy resource. The headers are formatted for compatibility with the program Sintax. The curated database was generated from both GenBank and Barcodes of Life databases, with a threshold of 400 bases. (PDF) [file pone.0301474.s005.pdf]

S3 Table. Curated database used for taxonomic assignment of cytochrome *c* oxidase I (CO1) metabarcode sequences, in FASTA format. Reference headers include the source accession from which the sequence was extracted and the taxonomy of the sequence according to the NCBI Taxonomy resource. The headers are formatted for compatibility with the program Sintax. The curated database was generated from both GenBank and Barcodes of Life databases, with a threshold of 400 bases.

>JF903538;tax=d:Eukarya,p:Arthropoda,c:Insecta,o:Hymenoptera,f:Halictidae,g:Lasioglossum,s:Lasioglossum nigroviride
ATACTTTACTTTATTTTTGCTATATGATCTGGAATAATTGGAGCTTCATTAAGAATAATT
ATTCGAATAGAATTAAGTGCACCAGGAAAATGAATTAATAATGATCAAATTTATAACACT
ATTATTACTTCTCATGCATTTGTAATAATTTTTTTTATAGTTATACCATTTATAATTGGA
GGATTCGGTAATTGATTAGTCCCTTTAATAATTGGTGCCCCTGATATAGCATTTCCTCGA
ATAAATAATATAAGATTTTGATTACTTATTCCATCAATATTTATATTATTAATAAGAAGT
ATTATATCATCTGGATCAGGAACTGGATGAACTGTATACCCCCCTCTATCTTCAATTATA
TATCACTCATCAATTTCAGTAGATTACACTATTTTTTCATTACATATTGCAGGAATTTCA
TCTATTATAGGAGCAATCAACTTTATTGTATCTATTCTATTAATAAAAAATATTTCAATT
AATTATGACCAAATTCCTTTATTTCCATGATCAGTAAAAATTACTGCCATCTTATTATTA
TTATCTCTCCCAGTTTTAGCGGGAGCTATTACTATACTTTTAACAGATCGAAATTTAAAT
ACTTCATTTTTTGACCCTTCTGGAGGAGGAGACCCAATTCTTTATCAACATTTATTT
>KR785909;tax=d:Eukarya,p:Arthropoda,c:Insecta,o:Hymenoptera,f:Halictidae,g:Lasioglossum,s:Lasioglossum nigroviride
AATACTTTACTTTATTTTTGCTATATGATCTGGGATAATTGGAGCTTCATTAAGAATAAT
TATTCGAATAGAATTAAGTGCACCAGGAAAATGAATTAATAATGATCAAATTTATAACAC
TATTATTACTTCTCATGCATTTGTAATAATTTTTTTTATAGTTATACCATTTATAATTGG
AGGATTCGGTAATTGATTAGTCCCTTTAATAATTGGTGCCCCTGATATAGCATTTCCTCG
AATAAATAATATAAGATTTTGATTACTTATTCCATCAATATTTATATTATTAATAAGAAG
TATTATATCATCTGGATCAGGAACTGGATGAACTGTATACCCCCCTCTATCTTCAATTAT
ATATCACTCATCAATTTCAGTAGATTACACTATTTTTTCATTACATATTGCAGGAATTTC
ATCTATTATAGGAGCAATCAACTTTATTGTATCTATTCTATTAATAAAAAATATTTCAAT
TAATTATGACCAAATTCCTTTATTTCCATGATCAGTAAAAATTACTGCCATCTTATTATT
ATTATCTCTCCCAGTTTTAGCGGGAGCTATTACTATACTTTTAACAGATCGAAATTTAAA
TACTTCATTTTTTGACCCTTCTGGAGGAGGAGACCCAATTCTTTATCAACATTTATTT
>KR797586;tax=d:Eukarya,p:Arthropoda,c:Insecta,o:Hymenoptera,f:Halictidae,g:Lasioglossum,s:Lasioglossum nigroviride
TTGGGGCTTCATTAAGAATAATTATTCGAATAGAATTAAGTGCACCAGGAAAATGAATTA
ATAATGATCAAATTTATAACACTATTATTACTTCTCATGCATTTGTAATAATTTTTTTTA
TAGTTATACCATTTATAATTGGAGGATTTGGTAATTGATTAGTCCCTTTAATAATTGGTG
CCCCTGATATAGCATTTCCTCGAATAAATAATATAAGATTTTGATTACTTATTCCATCAA
TATTTATATTATTAATAAGAAGTATTATATCATCTGGATCAGGAACTGGATGAACTGTAT
ACCCCCCTCTATCTTCAATTATATATCACTCATCAATTTCAGTAGATTACACTATTTTTT
CATTACATATTGCAGGAATTTCATCTATTATAGGAGCAATCAACTTTATTGTATCTATTC
TATTAATAAAAAATATTTCAATTAATTATGACCAAATTCCTTTATTTCCATGATCAGTAA
AAATTACTGCCATCTTATTATTATTATCTCTCCCAGTTTTAGCGGGAGCTATTACTATAC
TTTTAACAGATCGAAATTTAAATACTTCATTTTTTGACCCTTCTGGAGGAGGAGACCCAA
TTCTTTATCAACATTTATTT
>KR897194;tax=d:Eukarya,p:Arthropoda,c:Insecta,o:Hymenoptera,f:Halictidae,g:Lasioglossum,s:Lasioglossum nigroviride
AATACTTTACTTTATTTTTGCTATATGATCTGGAATAATTGGAGCTTCATTAAGAATAAT
TATTCGAATAGAATTAAGTGCACCAGGAAAATGAATTAATAATGATCAAATTTATAACAC
TATTATTACTTCTCATGCATTTGTAATAATTTTTTTTATAGTTATACCATTTATAATTGG

AGGATTCGGTAATTGATTAGTCCCTTTAATAATTGGTGCCCCTGATATAGCATTTCCTCG
AATAAATAATATAAGATTTTGATTACTTATTCCATCAATATTTATATTATTAATAAGAAG
TATTATATCATCTGGATCAGGAACTGGATGAACTGTATACCCCCCTCTATCTTCAATTAT
ATATCACTCATCAATTTCAGTAGATTACACTATTTTTTCATTACATATTGCAGGAATTTC
ATCTATTATAGGAGCAATCAACTTTATTGTATCTATTCTATTAATAAAAAATATTTCAAT
TAATTATGACCAAATTCCTTTATTTCCATGATCAGTAAAAATTACTGCCATCTTATTATT
ATTATCTCTCCCAGTTTTAGCGGGAGCTATTACTATACTTTTAACAGATCGAAATTTAAA
TACTTCATTTTTTGACCCTTCTGGAGGAGGAGACCCAATTCTTTATCAACATTTATTT
>MG335953;tax=d:Eukarya,p:Arthropoda,c:Insecta,o:Hymenoptera,f:Halictidae,g:Lasioglossum,s:Lasioglossum nigroviride
TTTATTTTTTGCTATATGATCTGGAATAATTGGAGCTTCATTAAGAATAATTATTCGAAT
AGAATTAAGTGCACCAGGAAAATGAATTAATAATGATCAAATTTATAACACTATTATTAC
TTCTCATGCATTTGTAATAATTTTTTTTATAGTTATACCATTTATAATTGGAGGATTCGG
TAATTGATTAGTCCCTTTAATAATTGGTGCCCCTGATATAGCATTTCCTCGAATAAATAA
TATAAGATTTTGATTACTTATTCCATCAATATTTATATTATTAATAAGAAGTATTATATC
ATCTGGATCAGGAACTGGATGAACTGTATACCCCCCTCTATCTTCAATTATATATCACTC
ATCAATTTCAGTAGATTACACTATTTTTTCATTACATATTGCAGGAATTTCATCTATTAT
AGGAGCAATCAACTTTATTGTATCTATTCTATTAATAAAAAATATTTCAATTAATTATGA
CCAAATTCCTTTATTTCCATGATCAGTAAAAATTACTGCCATCTTATTATTATTATCTCT
CCCAGTTTTAGCGGGAGCTATTACTATACTTTTAACAGATCGAAATTTTAATACTTCATT
TTTTGACCCTTCTGGAGGAGGAGACCCAATTCTTTATCAACATTTA
>MG337439;tax=d:Eukarya,p:Arthropoda,c:Insecta,o:Hymenoptera,f:Halictidae,g:Lasioglossum,s:Lasioglossum nigroviride
ATACTTTACTTTATTTTTGCTATATGATCTGGAATAATTGGAGCTTCATTAAGAATAATT
ATTCGAATAGAATTAAGTGCACCAGGAAAATGAATTAATAATGATCAAATTTATAACACT
ATTATTACTTCTCATGCATTTGTAATAATTTTTTTTATAGTTATACCATTTATAATTGGA
GGATTCGGTAATTGATTAGTCCCTTTAATAATTGGTGCCCCTGATATAGCATTTCCTCGA
ATAAATAATATAAGATTTTGATTACTTATTCCATCAATATTTATATTATTAATAAGAAGT
ATTATATCATCTGGATCAGGAACTGGATGAACTGTATACCCCCCTCTATCTTCAATTATA
TATCACTCATCAATTTCAGTAGATTACACTATTTTTTCATTACATATTGCAGGAATTTCA
TCTATTATAGGAGCAATCAACTTTATTGTATCTATTCTATTAATAAAAAATATTTCAATT
AATTATGACCAAATTCCTTTATTTCCATGATCAGTAAAAATTACTGCCATCTTATTATTA
TTATCTCTCCCAGTTTTAGCGGGAGCTATTACTATACTTTTAACAGAT
>MG339317;tax=d:Eukarya,p:Arthropoda,c:Insecta,o:Hymenoptera,f:Halictidae,g:Lasioglossum,s:Lasioglossum nigroviride
ATACTTTACTTTATTTTTGCTATATGATCTGGAATAATTGGGGCTTCATTAAGAATAATT
ATTCGAATAGAATTAAGTGCACCAGGAAAATGAATTAATAATGATCAAATTTATAACACT
ATTATTACTTCTCATGCATTTGTAATAATTTTTTTTATAGTTATACCATTTATAATTGGA
GGATTCGGTAATTGATTAGTCCCTTTAATAATTGGTGCCCCTGATATAGCATTTCCTCGA
ATAAATAATATAAGATTTTGATTACTTATTCCATCAATATTTATATTATTAATAAGAAGT
ATTATATCATCTGGATCAGGAACTGGATGAACTGTATACCCCCCTCTATCTTCAATTATA
TATCACTCATCAATTTCAGTAGATTACACTATTTTTTCATTACATATTGCAGGAATTTCA
TCTATTATAGGAGCAATCAACTTTATTGTATCTATTCTATTAATAAAAAATATTTCAATT
AATTATGACCAAATTCCTTTATTTCCATGATCAGTAAAAATTACTGCCATCTTATTATTA
TTATCTCTCCCAGTTTTAGCGGGAGCTATTACTATACTTTTAACA
>JF903494;tax=d:Eukarya,p:Arthropoda,c:Insecta,o:Hymenoptera,f:Halictidae,g:Lasioglossum,s:Lasioglossum admirandum
TATACTTTATTTTATTTTTGCTATATGAGCTGGAATAATTGGAGCTTCATTAAGAATAAT
TATTCGAATAGAACTAAGTGCTCCTGGAAAATGAATTAATAATGATCAAATTTATAATAC
TATTATTACCTCACATGCATTTGTAATAATTTTTTTTATAGTTATACCATTTATAATTGG
GGGATTTGGTAATTGATTAGTTCCTTTAATAATTGGAGCACCTGATATAGCATTCCCCCG

AATAAATAATATAAGATTTTGATTACTTATTCCATCAATATTTATATTATTAATAAGAAG
TATTATATCAACTGGTTCAGGAACTGGATGAACTGTATATCCCCCTTTATCATCTATTAT
ATACCATTCATCAATTTCAGTAGATTACACTATTTTTTCATTACACATTGCAGGAATTTC
ATCTATTATAGGAGCTATCAACTTCATTGTATCTATTTTACTTATAAAAAATATTTCAAT
TAATTATGATCAAATTCCCTTATTCCCATGATCAGTAAAAATTACTGCTATTTTATTATT
ATTATCTCTACCAGTTTTAGCGGGAGCCATTACTATACTTTTAACAGATCGAAATTTAAA
TACTTCATTTTTTGATCCTTCAGGAGGGGGAGACCCTATTCTTTATCAACATTTATTT
>HQ937904;tax=d:Eukarya,p:Arthropoda,c:Insecta,o:Hymenoptera,f:Halictidae,g:Lasioglossum,s:Lasioglossum albipenne
GAGCAGGAATAATTGGAGCTTCATTAAGAATAATTATCCGAATAGAATTAAGTGCCCCAG
GAAAATGAATTAATAATGATCAAATTTATAATACTATCATTACCTCACATGCATTTGTAA
TAATTTTTTTCATAGTAATACCATTCATAATTGGAGGATTTGGAAATTGACTAATCCCTT
TAATAATTGGAGCCCCTGATATAGCATTCCCTCGAATAAATAATATAAGATTTTGATTAC
TTATTCCATCAATATTCATATTATTAATAAGAAGTATTATATCATCTGGATCAGGAACAG
GATGAACTGTATACCCCCCTTTATCTTCAATTATATACCACTCATCAATTTCAGTAGATT
ACACTATCTTTTCATTACATATTGCAGGAATCTCCTCTATTATAGGAGCTATTAATTTTA
TTGTATCTATTTTACTTATAAAAAATATTTCAATTAATTATGATCAAATTCCTTTATTTC
CATGATCAGTAAAAATTACTGCTATCCTATTATTATTATCTCTACCAGTCTTAGCAGGAG
CTATTACTATATTATTAACAGATCGAAATTTAAATACTTCATTCTTTGACCCTTCAGGAG
GGGGAGATCCTATTCTTTATCAACATCTATTC
>JF903495;tax=d:Eukarya,p:Arthropoda,c:Insecta,o:Hymenoptera,f:Halictidae,g:Lasioglossum,s:Lasioglossum albipenne
ATACTTTACTTTATTTTTGCCATATGAGCCGGAATAATTGGAGCTTCATTAAGAATAATT
ATCCGAATAGAATTAAGTGCCCCAGGAAAATGAATTAATAATGATCAAATTTATAATACT
ATTATTACTTCACATGCATTTGTAATAATTTTTTTCATAGTAATACCATTCATAATTGGG
GGATTTGGAAATTGATTAATCCCTTTAATAATTGGAGCCCCTGACATAGCATTCCCTCGA
ATAAATAATATAAGATTTTGATTACTTATTCCATCAATATTCATATTATTAATAAGAAGT
ATTATATCATCTGGGTCAGGAACAGGATGAACTGTATACCCCCCCTATCTTCAATTATA
TACCACTCATCAATTTCAGTAGATTACACTATCTTTTCATTACATATTGCAGGAATCTCC
TCTATTATAGGAGCTATTAATTTTATTGTATCTATTTTACTTATAAAAAATATTTCAATT
AATTATGATCAAATTCCTTTATTTCCATGATCAGTAAAAATTACTGCTATCCTATTATTA
TTATCTCTACCAGTCTTAGCAGGAGCTATTACTATACTATTAACAGATCGAAATTTAAAT
ACTTCATTCTTTGACCCTTCAGGAGGGGGAGATCCTATTCTTTATCAACATCTATTC
>GU708187;tax=d:Eukarya,p:Arthropoda,c:Insecta,o:Hymenoptera,f:Halictidae,g:Lasioglossum,s:Lasioglossum anomalum
GATACTTTATTTTATTTTTGCAATATGAGCAGGAATAATTGGAGCTTCCTTAAGAATAAT
TATTCGAATAGAATTAAGTGCCCCTGGAAAATGAATTAATAATGATCAAATTTATAATAC
TATTATTACTTCCCATGCATTTGTAATAATTTTTTTTATAGTTATACCATTCATAATTGG
AGGATTTGGTAATTGATTAGTTCCTTTAATAATTGGAGCCCCTGATATAGCATTTCCTCG
AATAAATAATATAAGATTTTGATTACTTATACCATCAATATTTATATTATTAATAAGAAG
TATTATTTCATCTGGTTCAGGAACTGGATGAACTGTTTACCCCCCTTTATCTTCTATTAT
ATACCATTCATCAATTTCAGTAGACTATACCATTTTTTCACTTCATATTGCAGGAATTTC
TTCTATTATAGGAGCTATTAATTTTATTGTATCTATTTTACTTATAAAAAATATTTCAAT
CAATTATGATCAAATTCCTTTATTCCCATGATCAGTAAAAATTACTGCTATTCTATTATT
ATTATCTTTACCAGTTTTAGCAGGAGCTATCACTATATTATTAACAGATCGAAATTTAAA
TACTTCATTTTTTGACCCTTCAGGAGGAGGAGACCCAATTCTTTAT
>JF903497;tax=d:Eukarya,p:Arthropoda,c:Insecta,o:Hymenoptera,f:Halictidae,g:Lasioglossum,s:Lasioglossum anomalum
GATACTTTATTTTATTTTTGCAATATGAGCAGGAATAATTGGAGCTTCCTTAAGAATAAT
TATTCGAATAGAATTAAGTGCCCCTGGAAAATGAATTAATAATGATCAAATTTATAATAC
TATTATTACTTCCCATGCATTTGTAATAATTTTTTTTATAGTTATACCATTCATAATTGG

AGGATTTGGCAATTGATTAGTTCCTTTAATAATTGGAGCCCCTGATATAGCATTTCCTCG
AATAAATAATATAAGATTTTGATTACTTATACCATCAATATTTATATTATTAATAAGAAG
TATTATTTCATCTGGTTCAGGAACTGGATGAACTGTTTACCCCCCTTTATCTTCTATTAT
ATACCATTCATCAATTTCAGTAGACTATACCATTTTTTCACTTCATATTGCAGGAATTTC
TTCTATTATAGGGGCTATTAATTTTATTGTATCTATTTTACTTATAAAAAATATTTCAAT
CAATTATGATCAAATTCCTTTATTCCCATGATCAGTAAAAATTACTGCTATTCTATTATT
ATTATCTTTACCAGTTTTAGCAGGAGCTATCACTATATTATTAACAGATCGAAATTTAAA
TACTTCATTTTTTGACCCTTCAGGAGGAGGAGACCCAATTCTTTATCAACATTTATTT
>KR784140;tax=d:Eukarya,p:Arthropoda,c:Insecta,o:Hymenoptera,f:Halictidae,g:Lasioglossum,s:Lasioglossum anomalum
GATACTTTATTTTATTTTTGCAATATGAGCAGGAATAATTGGAGCTTCCTTAAGAATAAT
TATTCGAATAGAATTAAGTGCCCCTGGAAAATGAATTAATAATGATCAAATTTATAATAC
TATTATTACTTCCCATGCATTTGTAATAATTTTTTTTATAGTTATACCATTCATAATTGG
AGGATTTGGCAATTGATTAGTTCCTTTAATAATTGGAGCCCCTGATATAGCATTTCCTCG
AATAAATAATATAAGATTTTGATTACTTATACCATCAATATTTATATTATTAATAAGAAG
TATTATTTCATCTGGTTCAGGAACTGGATGAACTGTTTACCCCCCTTTATCTTCTATTAT
ATACCATTCATCAATTTCAGTAGACTATACCATTTTTTCACTTCATATTGCAGGAATTTC
TTCTATTATAGGGGCTATTAATTTTATTGTATCTATTTTACTTATAAAAAATATTTCAAT
CAATTATGATCAAATTCCTTTATTCCCATGATCAGTAAAAATTACTGCTATTCTATTATT
ATTATCTTTACCAGTTTTAGCAGGAGCTATCACTATATTATTAACAGATCGAAATTTAAA
TACTTCATTTTT
>KR784910;tax=d:Eukarya,p:Arthropoda,c:Insecta,o:Hymenoptera,f:Halictidae,g:Lasioglossum,s:Lasioglossum anomalum
ATGAGCAGGAATAATTGGAGCTTCCTTAAGAATAATTATTCGAATAGAATTAAGTGCCCC
TGGAAAATGAATTAATAATGATCAAATTTATAATACTATTATTACTTCCCATGCATTTGT
AATAATTTTTTTTATAGTTATACCATTCATAATTGGAGGATTTGGTAATTGATTAGTTCC
TTTAATAATTGGAGCCCCTGATATAGCATTTCCTCGAATAAATAATATAAGATTTTGATT
ACTTATACCATCAATATTTATATTATTAATAAGAAGTATTATTTCATCTGGTTCAGGAAC
TGGATGAACTGTTTACCCCCCTTTATCTTCTATTATATACCATTCATCAATTTCAGTAGA
CTATACCATTTTTTCACTTCATATTGCAGGAATTTCTTCTATTATAGGGGCTATTAATTT
TATTGTATCTATTTTACTTATAAAAAATATTTCAATCAATTATGATCAAATTCCTTTATT
CCCATGATCAGTAAAAATTACTGCTATTCTATTATTATTATCTTTACCAGTTTTAGCAGG
AGCTATCACTATATTATTAACAGATCGAAATTTAAATACTTCATTTTTTGACCCTTCAGG
AGGAGGAGACCCAATTCTTTATCAACATTTATTT
>KR794709;tax=d:Eukarya,p:Arthropoda,c:Insecta,o:Hymenoptera,f:Halictidae,g:Lasioglossum,s:Lasioglossum anomalum
GATACTTTATTTTATTTTTGCAATATGAGCAGGAATAATTGGAGCTTCCTTAAGAATAAT
TATTCGAATAGAATTAAGTGCCCCTGGAAAATGAATTAATAATGATCAAATTTATAATAC
TATTATTACTTCCCATGCATTTGTAATAATTTTTTTTATAGTTATACCATTCATAATTGG
AGGATTTGGCAATTGATTAGTTCCTTTAATAATTGGAGCCCCTGATATAGCATTTCCTCG
AATAAATAATATAAGATTTTGATTACTTATACCATCAATATTTATATTATTAATAAGAAG
TATTATTTCATCTGGTTCAGGAACTGGATGAACTGTTTACCCCCCTTTATCTTCTATTAT
ATACCATTCATCAATTTCAGTAGACTATACCATTTTTTCACTTCATATTGCAGGAATTTC
TTCTATTATAGGGGCTATTAATTTTATTGTATCTATTTTACTTATAAAAAATATTTCAAT
CAATTATGATCAAATTCCTTTATTCCCATGATCAGTAAAAATTACTGCTATTCTATTATT
ATTATCTTTACCAGTTTTAGCAGGAGCTATCACTATATTATTAACAGATCGAAATTTAAA
TACTTCATTTTTTGACCCTTCAGGAGGAGGAGACCCAATTCTTTA
>KR799355;tax=d:Eukarya,p:Arthropoda,c:Insecta,o:Hymenoptera,f:Halictidae,g:Lasioglossum,s:Lasioglossum anomalum
GATACTTTATTTTATTTTTGCAATATGAGCAGGAATAATTGGAGCTTCCTTAAGAATAAT
TATTCGAATAGAATTAAGTGCCCCTGGAAAATGAATTAATAATGATCAAATTTATAATAC

TATTATTACTTCCCATGCATTTGTAATAATTTTTTTTATAGTTATACCATTCATAATTGG
AGGATTTGGCAATTGATTAGTTCCTTTAATAATTGGAGCCCCTGATATAGCATTTCCTCG
AATAAATAATATAAGATTTTGATTACTTATACCATCAATATTTATATTATTAATAAGAAG
TATTATTTCATCTGGTTCAGGAACTGGATGAACTGTTTACCCCCCTTTATCTTCTATTAT
ATACCATTCATCAATTTCAGTAGACTATACCATTTTTTCACTTCATATTGCAGGAATTTC
TTCTATTATAGGGGCTATTAATTTTATTGTATCTATTTTACTTATAAAAAATATTTCAAT
CAATTATGATCAAATTCCTTTATTCCCATGATCAGTAAAAATTACTGCTATTCTATTATT
ATTATCTTTACCAGTTTTAGCAGGAGCTATCACTATATTATTAACAGATCGAAATTTAAA
TACTTCATTTTTTGACCCTTCAGGAGGAGGAGACCCAATTCTTTAT
>MG342878;tax=d:Eukarya,p:Arthropoda,c:Insecta,o:Hymenoptera,f:Halictidae,g:Lasioglossum,s:Lasioglossum anomalum
GATACTTTATTTTATTTTTGCAATATGAGCAGGAATAATTGGAGCTTCCTTAAGAATAAT
TATTCGAATAGAATTAAGTGCCCCTGGAAAATGAATTAATAATGATCAAATTTATAATAC
TATTATTACTTCCCATGCATTTGTAATAATTTTTTTTATAGTTATACCATTCATAATTGG
AGGATTTGGCAATTGATTAGTTCCTTTAATAATTGGAGCCCCTGATATAGCATTTCCTCG
AATAAATAATATAAGATTTTGATTACTTATACCATCAATATTTATATTATTAATAAGAAG
TATTATTTCATCTGGTTCAGGAACTGGATGAACTGTTTACCCCCCTTTATCTTCTATTAT
ATACCATTCATCAATTTCAGTAGACTATACCATTTTTTCACTTCATATTGCAGGAATTTC
TTCTATTATAGGGGCTATTAATTTTATTGTATCTATTTTACTTATAAAAAATATTTCAAT
CAATTATGATCAAATTCCTTTATTCCCATGATCAGTAAAAATTACTGCTATTCTATTATT
ATTATCTTTACCAGTTTTAGCAGGAGCTATCACTATATTATTAACAGATCGAAATTTAAA
TACTTCATTTTTTGACCC
>HQ937903;tax=d:Eukarya,p:Arthropoda,c:Insecta,o:Hymenoptera,f:Halictidae,g:Lasioglossum,s:Lasioglossum bruneri
TTGGGGCTTCATTAAGAATAATTATTCGAATAGAATTAAGTGCCCCAGGAAAATGAATTA
ATAATGACCAAATTTATAATACTATTATTACTTCTCATGCATTTGTAATAATTTTTTTTA
TAGTTATACCATTTATAATTGGAGGATTTGGAAATTGATTAGTCCCTTTAATAATTGGAG
CCCCTGATATAGCTTTTCCTCGAATAAATAATATAAGATTTTGATTACTTATCCCATCAA
TATTTATATTATTAATAAGAAGAATTATAGCTTCAGGATCAGGAACTGGATGAACTGTAT
ACCCCCCCTTATCATCAATTATATACCATTCATCAATTTCAGTAGATTACACTATCTTTT
CATTACATATTGCAGGAATTTCATCTATTATAGGAGCCATCAATTTTATTGTATCAATTC
TTCTTATAAAAAATATTTCAATTAATTATGATCAAATTCCTTTATTTCCATGATCAGTAA
AAATTACTGCTATTTTATTATTATTATCTTTACCAGTTTTAGCAGGAGCAATTACTATAC
TTTTAACAGATCGAAACTTAAATACCTCATTTTTTGACCCTTCTGGAGGAGGAGACCCTA
TTCTTTATCAACATTTATTT
>JF903499;tax=d:Eukarya,p:Arthropoda,c:Insecta,o:Hymenoptera,f:Halictidae,g:Lasioglossum,s:Lasioglossum bruneri
ATACTTTATTTTATTTTTGCTATATGATCTGGAATAATTGGAGCTTCATTAAGAATAATT
ATTCGAATAGAATTAAGTGCCCCAGGAAAATGAATTAATAATGACCAAATTTATAATACT
ATTATTACTTCTCATGCATTTGTAATAATTTTTTTTATAGTTATACCATTTATAATTGGA
GGATTTGGAAATTGATTAGTCCCTTTAATAATTGGAGCCCCTGATATAGCTTTTCCTCGA
ATAAATAATATAAGATTTTGATTACTTATCCCATCAATATTTATATTATTAATAAGAAGA
ATTATAGCTTCAGGATCAGGAACTGGATGAACTGTATACCCCCCCTTATCATCAATTATA
TACCATTCATCAATTTCAGTAGATTACACTATCTTTTCATTACATATTGCAGGAATTTCA
TCTATTATAGGAGCCATCAATTTTATTGTATCAATTCTTCTTATAAAAAATATTTCAATT
AATTATGATCAAATTCCTTTATTTCCATGATCAGTAAAAATTACTGCTATTTTATTATTA
TTATCTTTACCAGTTTTAGCAGGAGCAATTACTATACTTTTAACAGATCGAAACTTAAAT
ACCTCATTTTTTGACCCTTCTGGAGGAGGAGACCCTATTCTTTATCAACATTTATTT
>KJ165284;tax=d:Eukarya,p:Arthropoda,c:Insecta,o:Hymenoptera,f:Halictidae,g:Lasioglossum,s:Lasioglossum bruneri
TGGAGCTTCATTAAGAATAATTATTCGAATAGAATTAAGTGCCCCAGGAAAATGAATTAA

TAATGACCAAATTTATAATACTATTATTACTTCTCATGCATTTGTAATAATTTTTTTTAT
AGTTATACCATTTATAATTGGAGGATTTGGAAATTGATTAGTCCCTTTAATAATTGGAGC
CCCTGATATAGCTTTTCCTCGAATAAATAATATAAGATTTTGATTACTTATCCCATCAAT
ATTTATATTATTAATAAGAAGAATTATAGCTTCAGGATCAGGAACTGGATGAACTGTATA
CCCCCCCTTATCATCAATTATATACCATTCATCAATTTCAGTAGATTACACTATCTTTTC
ATTACATATTGCAGGAATTTCATCTATTATAGGAGCCATCAATTTTATTGTATCAATTCT
TCTTATAAAAAATATTTCAATTAATTATGATCAAATTCCTTTATTTCCATGATCAGTAAA
AATTACTGCTATTTTATTATTATTATCTTTACCAGTTTTAGCAGGAGCAATTACTATACT
TTTAACAGATCGAAACTTAAATACCTCATTTTTTGACCCTTCTGGGGGAGGGGACCCTAT
TCTTTATCAACATTTATTT
>KJ167522;tax=d:Eukarya,p:Arthropoda,c:Insecta,o:Hymenoptera,f:Halictidae,g:Lasioglossum,s:Lasioglossum bruneri
TTAATAATGACCAAATTTATAATACTATTATTACTTCTCATGCATTTGTAATAATTTTTT
TTATAGTTATACCATTTATAATTGGAGGATTTGGAAATTGATTAGTCCCTTTAATAATTG
GAGCCCCTGATATAGCTTTTCCTCGAATAAATAATATAAGATTTTGATTACTTATCCCAT
CAATATTTATATTATTAATAAGAAGAATTATAGCTTCAGGATCAGGAACTGGATGAACTG
TATACCCCCCCTTATCATCAATTATATACCATTCATCAATTTCAGTAGATTACACTATCT
TTTCATTACATATTGCAGGAATTTCATCTATTATAGGAGCCATCAATTTTATTGTATCAA
TTCTTCTTATAAAAAATATTTCAATTAATTATGATCAAATTCCTTTATTTCCATGATCAG
TAAAAATTACTGCTATTTTATTATTATTATCTTTACCAGTTTTAGCAGGAGCAATTACTA
TACTTTTAACAGATCGAAACTTAAATACCTCATTTTTTGACCCTTCTGGGGGAGGGGACC
CTATTCTTTATCAACATTTATTT
>KR886474;tax=d:Eukarya,p:Arthropoda,c:Insecta,o:Hymenoptera,f:Halictidae,g:Lasioglossum,s:Lasioglossum bruneri
ATACTTTATTTTATTTTTGCTATATGATCTGGAATAATTGGAGCTTCATTAAGAATAATT
ATTCGAATAGAATTAAGTGCCCCAGGAAAATGAATTAATAATGACCAAATTTATAATACT
ATTATTACTTCTCATGCATTTGTAATAATTTTTTTTATAGTTATACCATTTATAATTGGA
GGATTTGGAAATTGATTAGTCCCTTTAATAATTGGAGCCCCTGATATAGCTTTTCCTCGA
ATAAATAATATAAGATTTTGATTACTTATCCCATCAATATTTATATTATTAATAAGAAGA
ATTATAGCTTCAGGATCAGGAACTGGATGAACTGTATACCCCCCCTTATCATCAATTATA
TACCATTCATCAATTTCAGTAGATTACACTATCTTTTCATTACATATTGCAGGAATTTCA
TCTATTATAGGAGCCATCAATTTTATTGTATCAATTCTTCTTATAAAAAATATTTCAATT
AATTATGATCAAATTCCTTTATTTCCATGATCAGTAAAAATTACTGCTATTTTATTATTA
TTATCTTTACCAGTTTTAGCAGGA
>HM423304;tax=d:Eukarya,p:Arthropoda,c:Insecta,o:Hymenoptera,f:Halictidae,g:Lasioglossum,s:Lasioglossum coreopsis
AATACTTTACTTTATTTTTGCAATATGAGCAGGAATAATTGGAGCTTCTTTAAGAATAAT
TATTCGAATAGAATTAAGTGCTCCTGGAAAATGAATTAATAATGACCAAATCTATAATAC
TATTATTACTTCTCATGCATTTATTATAATTTTTTTTATAGTTATACCATTTATAATTGG
AGGATTTGGTAATTGATTAGTCCCTTTAATAATTGGAGCCCCTGATATAGCATTCCCTCG
AATAAATAATATAAGATTTTGATTATTAATCCCATCAATATTTATATTATTAATAAGAAG
AATTCTATCCTCAGGATCAGGAACAGGATGAACAGTTTATCCTCCTTTATCATCTATTAT
ATATCATTCATCAATTTCAGTAGATTACACCATTTTTTCTTTACATATTGCAGGAATTTC
ATCCATTATAGGTGCTATTAATTTTATTGTATCAATTTTACTTATAAAAAATATTTCAAT
TAATTATGATCAAATTCCACTATTCCCATGATCAGTAAAAATTACTGCTATTTTATTATT
ATTATCCTTACCAGTTTTAGCAGGAGCTATTACTATATTATTAACTGATCGAAATTTAAA
TACTTCATTTTTTGACCCTTCAGGAGGAGGAGACCCTATTCTTTATCAACATTTATTT
>HM423306;tax=d:Eukarya,p:Arthropoda,c:Insecta,o:Hymenoptera,f:Halictidae,g:Lasioglossum,s:Lasioglossum coreopsis
AATACTTTACTTTATTTTTGCGATATGAGCAGGAATAATTGGAGCTTCTTTAAGAATAAT
TATTCGAATAGAATTAAGTGCTCCTGGAAAATGAATTAATAATGACCAAATCTATAATAC

TATTATTACTTCTCATGCATTTATTATAATTTTTTTTATAGTTATACCATTTATAATTGG
AGGATTTGGTAATTGATTAGTCCCTTTAATAATTGGAGCCCCTGATATAGCATTCCCTCG
AATAAATAATATAAGATTTTGATTATTAATCCCATCAATATTTATATTATTAATAAGAAG
AATTCTATCCTCAGGATCAGGAACAGGATGAACAGTTTATCCTCCTTTATCATCTATTAT
ATATCATTCATCAATTTCAGTAGATTACACCATTTTTTCTTTACATATTGCAGGAATTTC
ATCCATTATAGGTGCTATTAATTTTATTGTATCAATTTTACTTATAAAAAATATTTCAAT
TAATTATGATCAAATTCCACTATTCCCATGATCAGTAAAAATTACTGCTATTTTATTATT
ATTATCCTTACCAGTTTTAGCAGGAGCTATTACTATATTATTAACTGATCGAAATTTAAA
TACTTCATTTTTTGACCCTTCAGGAGGAGGAGACCCTATTCTTTATCAACATTTATTT
>JF903503;tax=d:Eukarya,p:Arthropoda,c:Insecta,o:Hymenoptera,f:Halictidae,g:Lasioglossum,s:Lasioglossum coreopsis
AATACTTTACTTTATTTTTGCAATATGAGCAGGAATAATTGGAGCTTCTTTAAGAATAAT
TATTCGAATAGAATTAAGTGCTCCTGGAAAATGAATTAATAATGACCAAATCTATAATAC
TATTATTACTTCTCATGCATTTATTATAATTTTTTTTATAGTTATACCATTTATAATTGG
AGGATTCGGTAATTGATTAGTCCCTTTAATAATTGGAGCCCCTGATATAGCATTCCCTCG
AATAAATAATATAAGATTTTGATTATTAATCCCATCAATATTTATATTATTAATAAGAAG
AATTCTCTCCTCAGGATCAGGAACAGGATGAACAGTTTATCCTCCTTTATCATCTATTAT
ATATCATTCATCAATTTCAGTAGATTACACTATTTTTTCTTTACATATTGCAGGAATTTC
ATCCATTATAGGTGCTATTAATTTTATTGTATCAATTTTACTTATAAAAAATATTTCAAT
TAATTATGATCAAATTCCACTATTCCCATGATCAGTAAAAATTACTGCCATTTTATTATT
ATTATCTTTACCAGTTTTAGCAGGAGCTATTACTATATTATTAACTGATCGAAATTTAAA
TACTTCATTTTTTGACCCTTCAGGAGGAGGAGACCCTATTCTTTATCAACATTTATTT
>JN262393;tax=d:Eukarya,p:Arthropoda,c:Insecta,o:Hymenoptera,f:Apidae,g:Bombus,s:Bombus ternarius
AATAATATATTTTATTTTTGCAATATGATCAGGAATAATTGGATCCTCAATAAGATTATT
AATTCGAATAGAACTTAGWCATCCTGGAATATGAATTAACAATGATCAAATTTACAATTC
ATTAGTAACCAGACATGCATTTTTAATAATTTTTTTTATAGTTATACCATTTATAATTGG
AGGATTTGGAAATTATTTAATTCCATTAATATTAGGATCACCTGATATAGCTTTTCCACG
AATAAATAATATTAGATTTTGATTATTACCACCATCTCTTTTTATATTATTATTAAGAAC
TTTATTTACACCTAATGTAGGAACAGGTTGAACTGTATATCCACCTTTATCATCWTATAT
ATTTCATTCATCACCTTCTRTWGATATTGCAATTTTTTCTTTACATATAACAGGAATTTC
TTCAATTATTGGTTCATTAAATTTTATTGTAACAATTATATTAATAAAAAATTTTTCATT
AAATTATGATCAAATTAATTTATTTTCATGATCAGTATGTATTACAGTAATATTATTAAT
TTTATCBCTTCCAGTTTTAGCAGGAGCAATTACTATACTTTTATTTGATCGAAATTTTAA
TACATCATTTTTTGATCCTATAGGAGGTGGAGATCCAATTTTATATCAACATTTATTT
>JF903510;tax=d:Eukarya,p:Arthropoda,c:Insecta,o:Hymenoptera,f:Halictidae,g:Lasioglossum,s:Lasioglossum dreisbachi
ATACTTTATTTTATTTTCGCTATATGAGCTGGGATAATTGGAGCTTCATTAAGAATAATT
ATTCGAATAGAACTAAGTGCTCCTGGAAAATGAATTAATAATGATCAAATTTATAATACT
ATTATTACCTCACATGCATTTGTAATAATTTTTTTTATAGTTATACCATTTATAATTGGA
GGTTTTGGTAATTGATTAGTTCCTTTAATAATTGGAGCACCTGATATAGCATTCCCCCGA
ATAAATAATATAAGATTTTGATTACTTATTCCATCAATATTTATATTATTAATAAGAAGT
ATTATATCATCTGGCTCAGGAACTGGATGAACTGTATACCCTCCTTTATCATCTATTATA
TATCATTCATCAATTTCAGTAGATTACACTATTTTTTCATTACATATTGCAGGAATTTCA
TCTATTATAGGAGCTATCAACTTCATTGTATCTATTTTACTTATAAAAAATATTTCAATT
AATTATGATCAAATTCCCTTATTTCCATGGTCAGTAAAAATTACTGCTATTTTATTATTA
TTATCTCTACCAGTTTTGGCAGGAGCTATTACTATACTTTTAACAGATCGAAATTTAAAT
ACTTCATTTTTTGACCCTTCAGGAGGAGGGGACCCTATTCTTTATCAACATTTATTT
>KR783124;tax=d:Eukarya,p:Arthropoda,c:Insecta,o:Hymenoptera,f:Halictidae,g:Lasioglossum,s:Lasioglossum dreisbachi
CATACTTTATTTTATTTTCGCTATATGAGCTGGGATAATTGGAGCTTCATTAAGAATAAT
TATTCGAATAGAACTAAGTGCTCCTGGAAAATGAATTAATAATGATCAAATTTATAATAC

TATTATTACCTCACATGCATTTGTAATAATTTTTTTATAGTTATACCATTTATAATTGG
AGGTTTTGGTAATTGATTAGTTCCTTTAATAATTGGAGCACCTGATATAGCATTCCCCCG
AATAAATAATATAAGATTTTGATTACTTATTCCATCAATATTTATATTATTAATAAGAAG
TATTATATCATCTGGCTCAGGAACTGGATGAACTGTATACCCTCCTTTATCATCTATTAT
ATATCATTCATCAATTTCAGTAGATTACACTATTTTTTCATTACATATTGCAGGAATTTC
ATCTATTATAGGAGCTATCAACTTCATTGTATCTATTTTACTTATAAAAAATATTTCAAT
TAATTATGATCAAATTCCCTTATTTCCATGATCAGTAAAAATTACTGCTATTTTATTATT
ATTATCTCTACCAGTTTTAGCAGGAGCTATTACTATACTTTTAACAGATCGAAATTTAAA
TACTTCATTTTTTGACCCTTCAGGAGGAGGGGACCCTATTCTTTATCAACATTTATTT
>HM905760;tax=d:Eukarya,p:Arthropoda,c:Insecta,o:Hymenoptera,f:Halictidae,g:Lasioglossum,s:Lasioglossum hartii
AATACTATATTTTATTTTCGCTATATGAGCTGGAATAATTGGAGCTTCATTAAGAATAAT
TATTCGAATAGAATTAAGTGCACCAGGAAAATGAATTAATAATGATCAAATTTATAATAC
TATTATTACCTCTCATGCATTTGTTATAATTTTTTTATAGTAATACCATTTATAATTGG
AGGATTCGGAAATTGATTAGTTCCTTTAATAATTGGGGCCCCTGATATAGCATTCCCTCG
AATAAATAATATAAGATTTTGATTACTTATCCCATCTATATTTATATTATTAATAAGAAG
AATTATTTCTTCAGGTTCAGGAACTGGATGAACTGTATACCCACCTTTATCTTCTATTAT
ATATCATTCATCAACTTCAGTAGATTTTACTATTTTCTCATTACATATTGCAGGAATTTC
ATCTATTATAGGAGCTATTAATTTCATTGTATCAATTTTATTAATAAAAAATATTTCAAT
TAATTATGATCAAATTCCTTTATTCCCATGATCAGTAAAAATTACTGCTATTTTACTTTT
ATTATCTTTACCAGTTTTAGCAGGAGCTATTACTATACTTTTAACAGATCGAAATTTAAA
CACTTCATTCTTTGACCCTTCAGGAGGAGGAGACCCTATTCTTTATCAACATCTATTT
>HM905893;tax=d:Eukarya,p:Arthropoda,c:Insecta,o:Hymenoptera,f:Halictidae,g:Lasioglossum,s:Lasioglossum hartii
AATACTATATTTTATTTTCGCTATATGAGCTGGAATAATTGGAGCTTCATTAAGAATAAT
TATTCGAATAGAATTAAGTGCACCAGGAAAATGAATTAATAATGATCAAATTTATAATAC
TATTATTACCTCTCATGCATTTGTTATAATTTTTTTATAGTAATACCATTTATAATTGG
AGGATTCGGAAATTGATTAGTTCCTTTAATAATTGGGGCCCCTGATATAGCATTCCCTCG
AATAAATAATATAAGATTTTGATTACTTATCCCATCTATATTTATATTATTAATAAGAAG
AATTATTTCTTCAGGTTCAGGAACTGGATGAACTGTATACCCACCTTTATCTTCTATTAT
ATATCATTCATCAACTTCAGTAGATTTTACTATTTTCTCATTACATATTGCAGGAATTTC
ATCTATTATAGGAGCTATTAATTTCATTGTATCAATTTTATTAATAAAAAATATTTCAAT
TAATTATGATCAAATTCCTTTATTCCCATGATCAGTAAAAA
>JF903519;tax=d:Eukarya,p:Arthropoda,c:Insecta,o:Hymenoptera,f:Halictidae,g:Lasioglossum,s:Lasioglossum illinoense
AATACTTTATTTTATCTTTGCTATATGAGCTGGAATAATTGGAGCTTCATTAAGAATAAT
TATTCGTATAGAATTAAGTGCCCCAGGAAAATGAATTAATAATGATCAAATTTATAATAC
TATTATTACTTCACATGCATTTGTAATAATTTTTTTATAGTTATACCATTTATAATTGG
AGGATTTGGTAATTGATTAGTTCCTTTAATAATTGGTGCCCCTGATATAGCATTCCCTCG
AATAAATAATATAAGATTTTGATTACTTATTCCATCAATATTTATATTATTAATAAGAAG
TATTATATCATCTGGTTCAGGAACTGGATGAACCGTATATCCTCCCTTATCTTCAATTAT
ATATCATTCATCAATTTCAGTAGATTATACTATTTTTTCATTACATATTGCAGGAATTTC
ATCTATTATAGGAGCAATTAACTTTATTGTATCTATTATACTTATAAAAAATATCTCAAT
TAATTATGACCAAATTCCTTTATTTCCATGATCAGTAAAAATTACTGCTATTCTATTATT
ATTATCACTACCAGTTTTAGCAGGAGCTATTACTATACTCTTAACAGATCGAAATTTAAA
TACTTCATTCTTCGATCCTTCTGGAGGAGGAGACCCTATTCTATATCAACATTTATT
>HM414393;tax=d:Eukarya,p:Arthropoda,c:Insecta,o:Hymenoptera,f:Halictidae,g:Lasioglossum,s:Lasioglossum laevissimum
AATACTTTATTTTATTTTTGCTATATGATCTGGAATAATTGGAGCCTCACTAAGAATAAT
TATTCGAATAGAATTAAGTGCACCAGGAAAATGAATTAATGATGATCAAATTTATAACAC
TATTATTACTTCCCATGCATTTGTAATAATTTTTTTATAGTTATACCATTTATAATTGG
AGGATTCGGTAATTGATTGGTTCCTTTAATAATTGGTGCACCTGATATAGCATTCCCTCG
AATAAATAACATAAGATTTTGATTACTTATTCCATCAATATTTATATTATTAATAAGAAG

TATTATATCATCTGGATCAGGAACTGGGTGAACTGTATACCCCCCCTTATCTTCAATTAT
ATACCACTCATCAATTTCAGTAGATTACACTATCTTTTCATTACATATTGCAGGAATTTC
ATCTATTATAGGAGCAATCAACTTTATTGTATCTATTTTACTTATAAAAAATATTTCAAT
TAATTATGATCAAATTCCTTTATTCCCATGATCAGTAAAAATTACTGCTATCTTATTACT
ATTATCTCTCCCAGTTTTAGCGGGAGCTATTACTATACTTTTAACAGATCGAAATTTAAA
TACTTCATTTTTTGACCCTTCTGGGGGAGGAGATCCAATTCTTTATCAACATTTATTT
>GU666299;tax=d:Eukarya,p:Arthropoda,c:Insecta,o:Hymenoptera,f:Halictidae,g:Lasioglossum,s:Lasioglossum laevissimum
AATACTTTATTTTATTTTTGCTATATGATCTGGAATAATTGGAGCCTCACTAAGAATAAT
TATTCGAATAGAATTAAGTGCACCAGGAAAATGAATTAATGATGATCAAATTTATAACAC
TATTATTACTTCCCATGCATTTGTAATAATTTTTTTTATAGTTATACCATTTATAATTGG
AGGATTCGGTAATTGATTGGTTCCTTTAATAATTGGTGCACCTGATATAGCATTCCCTCG
AATAAATAACATAAGATTTTGATTACTTATTCCATCAATATTTATATTATTAATAAGAAG
TATTATATCATCTGGATCAGGAACTGGGTGAACTGTATACCCCCCCTTATCTTCAATTAT
ATACCACTCATCAATTTCAGTAGATTACACTATCTTTTCATTACATATTGCAGGAATTTC
ATCTATTATAGGAGCAATCAACTTTATTGTATCTATTTTACTTATAAAAAATATTTCAAT
TAATTATGATCAAATTCCTTTATTCCCATGATCAGTAAAAATTACTGCTATCTTATTACT
ATTATCTCTCCCAGTTTTAGCAGGAGCTATTACTATACTTTTAACAGATCGAAATTTAAA
TACTTCATTTTTTGACCCTTCTGGGGGAGGAGATCCAATTCTTTATCAACATTTATTT
>GU690106;tax=d:Eukarya,p:Arthropoda,c:Insecta,o:Hymenoptera,f:Halictidae,g:Lasioglossum,s:Lasioglossum laevissimum
AATACTTTATTTTATTTTTGCTATATGATCTGGGATAATTGGAGCCTCACTAAGAATAAT
TATTCGAATAGAATTAAGTGCACCAGGAAAATGAATTAATGATGATCAAATTTATAACAC
TATTATTACTTCCCATGCATTTGTAATAATTTTTTTTATAGTTATACCATTTATAATTGG
AGGATTCGGTAATTGATTGGTTCCTTTAATAATTGGTGCACCTGATATAGCATTCCCTCG
AATAAATAACATAAGATTTTGATTACTTATTCCATCAATATTTATATTATTAATAAGAAG
TATTATATCATCTGGATCAGGAACTGGGTGAACTGTATACCCCCCCTTATCTTCAATTAT
ATACCACTCATCAATTTCAGTAGATTACACTATCTTTTCATTACATATTGCAGGAATTTC
ATCTATTATAGGAGCAATCAACTTTATTGTATCTATTTTACTTATAAAAAATATTTCAAT
TAATTATGATCAAATTCCTTTATTCCCATGATCAGTAAAAATTACTGCTATCTTATTACT
ATTATCTCTCCCAGTTTTAGCAGGAGCTATTACTATACTTTTAACAGATCGAAATTTAAA
TACTTCATTTTTTGACCCTTCTGGAGGAGGAGATCCAATTCTTTATCAACATTTATTT
>HQ929461;tax=d:Eukarya,p:Arthropoda,c:Insecta,o:Hymenoptera,f:Halictidae,g:Lasioglossum,s:Lasioglossum laevissimum
ATAATTGGAGCCTCACTAAGAATAATTATTCGAATAGAATTAAGTGCACCAGGAAAATGA
ATTAATGATGATCAAATTTATAACACTATTATTACTTCCCATGCATTTGTAATAATTTTT
TTTATAGTTATACCATTTATAATTGGAGGATTCGGTAATTGATTGGTTCCTTTAATAATT
GGTGCACCTGATATAGCATTCCCTCGAATAAATAACATAAGATTTTGATTACTTATTCCA
TCAATATTTATATTATTAATAAGAAGTATTATATCATCTGGATCAGGAACTGGGTGAACT
GTATACCCCCCCTTATCTTCAATTATATACCACTCATCAATTTCAGTAGATTACACTATC
TTTTCATTACATATTGCAGGAATTTCATCTATTATAGGAGCAATCAACTTTATTGTATCT
ATTTTACTTATAAAAAATATTTCAATTAATTATGATCAAATTCCTTTATTCCCATGATCA
GTAAAAATTACTGCTATCTTATTACTATTATCTCTCCCAGTTTTAGCAGGAGCTATTACT
ATACTTTTAACAGATCGAAATTTAAATACTTCATTTTTTGACCCTTCTGGAGGAGGAGAT
CCAATTCTTTATCAACATTTATTT
>HQ929462;tax=d:Eukarya,p:Arthropoda,c:Insecta,o:Hymenoptera,f:Halictidae,g:Lasioglossum,s:Lasioglossum laevissimum
AATACTTTATTTTATTTTTGCTATATGATCTGGGATAATTGGAGCCTCACTAAGAATAAT
TATTCGAATAGAATTAAGTGCACCAGGAAAATGAATTAATGATGATCAAATTTATAACAC
TATTATTACTTCCCATGCATTTGTAATAATTTTTTTTATAGTTATACCATTTATAATTGG
AGGATTCGGTAATTGATTGGTTCCTTTAATAATTGGTGCACCTGATATAGCATTCCCTCG

AATAAATAACATAAGATTTTGATTACTTATTCCATCAATATTTATATTATTAATAAGAAG
TATTATATCATCTGGATCAGGAACTGGGTGAACTGTATACCCCCCCTTATCTTCAATTAT
ATACCACTCATCAATTTCAGTAGATTACACTATCTTTTCATTACATATTGCAGGAATTTC
ATCTATTATAGGAGCAATCAACTTTATTGTATCTATTTTACTTATAAAAAATATTTCAAT
TAATTATGATCAAATTCCTTTATTCCCATGATCAGTAAAAATTACTGCTATCTTATTACT
ATTATCTCTCCCAGTTTTAGCAGGAGCTATTACTATACTTTTAACAGATCGAAATTTAAA
TACTTCATTTTTTGACCCTTCTGGAGGAGGAGATCCAATTCTTTATC
>KR421723;tax=d:Eukarya,p:Arthropoda,c:Insecta,o:Hymenoptera,f:Halictidae,g:Lasioglossum,s:Lasioglossum laevissimum
AATACTTTATTTTATTTTTGCTATATGATCTGGGATAATTGGAGCCTCACTAAGAATAAT
TATTCGAATAGAATTAAGTGCACCAGGAAAATGAATTAATGATGATCAAATTTATAACAC
TATTATTACTTCCCATGCATTTGTAATAATTTTTTTTATAGTTATACCATTTATAATTGG
AGGATTCGGTAATTGATTGGTTCCTTTAATAATTGGTGCACCTGATATAGCATTCCCTCG
AATAAATAACATAAGATTTTGATTACTTATTCCATCAATATTTATATTATTAATAAGAAG
TATTATATCATCTGGATCAGGAACTGGGTGAACTGTATACCCCCCCTTATCTTCAATTAT
ATACCACTCATCAATTTCAGTAGATTACACTATCTTTTCATTACATATTGCAGGAATTTC
ATCTATTATAGGAGCAATCAACTTTATTGTATCTATTTTACTTATAAAAAATATTTCAAT
TAATTATGATCAAATTCCTTTATTCCCATGATCAGTAAAAATTACTGCTATCTTATTACT
ATTATCTCTCCCAGTTTTAGCAGGAGCTATTACTATACTTTTAACAGATCGAAATTTAAA
TACTTCATTTTTT
>KR783723;tax=d:Eukarya,p:Arthropoda,c:Insecta,o:Hymenoptera,f:Halictidae,g:Lasioglossum,s:Lasioglossum laevissimum
AATACTTTATTTTATTTTTGCTATATGATCTGGAATAATTGGAGCCTCACTAAGAATAAT
TATTCGAATAGAATTAAGTGCACCAGGAAAATGAATTAATGATGATCAAATTTATAACAC
TATTATTACTTCCCATGCATTTGTAATAATTTTTTTTATAGTTATACCATTTATAATTGG
AGGATTCGGTAATTGATTGGTTCCTTTAATAATTGGTGCACCTGATATAGCATTCCCTCG
AATAAATAACATAAGATTTTGATTACTTATTCCATCAATATTTATATTATTAATAAGAAG
TATTATATCATCTGGATCAGGAACTGGGTGAACTGTATACCCCCCCTTATCTTCAATTAT
ATACCACTCATCAATTTCAGTAGATTACACTATCTTTTCATTACATATTGCAGGAATTTC
ATCTATTATAGGAGCAATCAACTTTATTGTATCTATTTTACTTATAAAAAATATTTCAAT
TAATTATGATCAAATTCCTTTATTCCCATGATCAGTAAAAATTACTGCTATCTTATTACT
ATTATCTCTCCCAGTTTTAGCGGGAGC
>KR885215;tax=d:Eukarya,p:Arthropoda,c:Insecta,o:Hymenoptera,f:Halictidae,g:Lasioglossum,s:Lasioglossum laevissimum
AATACTTTATTTTATTTTTGCTATATGATCTGGAATAATTGGAGCCTCACTAAGAATAAT
TATTCGAATAGAATTAAGTGCACCAGGAAAATGAATTAATGATGATCAAATTTATAACAC
TATTATTACTTCCCATGCATTTGTAATAATTTTTTTTATAGTTATACCATTTATAATTGG
AGGATTCGGTAATTGATTGGTTCCTTTAATAATTGGTGCACCTGATATAGCATTCCCTCG
AATAAATAACATAAGATTTTGATTACTTATTCCATCAATATTTATATTATTAATAAGAAG
TATTATATCATCTGGATCAGGAACTGGGTGAACTGTATACCCCCCCTTATCTTCAATTAT
ATACCACTCATCAATTTCAGTAGATTACACTATCTTTTCATTACATATTGCAGGAATTTC
ATCTATTATAGGAGCAATCAACTTTATTGTATCTATTTTACTTATAAAAAATATTTCAAT
TAATTATGATCAAATTCCTTTATTCCCATGATCAGTAAAAATTACTGCTATCTTATTACT
ATTATCTCTCCCAGTTTTAGCG
>KR899016;tax=d:Eukarya,p:Arthropoda,c:Insecta,o:Hymenoptera,f:Halictidae,g:Lasioglossum,s:Lasioglossum laevissimum
AATACTTTATTTTATTTTTGCTATATGATCTGGGATAATTGGAGCCTCACTAAGAATAAT
TATTCGAATAGAATTAAGTGCACCAGGAAAATGAATTAATGATGATCAAATTTATAACAC
TATTATTACTTCCCATGCATTTGTAATAATTTTTTTTATAGTTATACCATTTATAATTGG
AGGATTCGGTAATTGATTGGTTCCTTTAATAATTGGTGCACCTGATATAGCATTCCCTCG
AATAAATAACATAAGATTTTGATTACTTATTCCATCAATATTTATATTATTAATAAGAAG

TATTATATCATCTGGATCAGGAACTGGGTGAACTGTATACCCCCCCTTATCTTCAATTAT
ATACCACTCATCAATTTCAGTAGATTACACTATCTTTTCATTACATATTGCAGGAATTTC
ATCTATTATAGGAGCAATCAACTTTATTGTATCTATTTTACTTATAAAAAATATTTCAAT
TAATTATGATCAAATTCCTTTATTCCCATGATCAGTAAAAATTACTGCTATCTTATTACT
ATTATCTCTCCCAGTTTTAGCAGGA
>MG343052;tax=d:Eukarya,p:Arthropoda,c:Insecta,o:Hymenoptera,f:Halictidae,g:Lasioglossum,s:Lasioglossum laevissimum
AATACTTTATTTTATTTTTGCTATATGATCTGGGATAATTGGAGCCTCACTAAGAATAAT
TATTCGAATAGAATTAAGTGCACCAGGAAAATGAATTAATGATGATCAAATTTATAACAC
TATTATTACTTCCCATGCATTTGTAATAATTTTTTTTATAGTTATACCATTTATAATTGG
AGGATTCGGTAATTGATTGGTTCCTTTAATAATTGGTGCACCTGATATAGCATTCCCTCG
AATAAATAACATAAGATTTTGATTACTTATTCCATCAATATTTATATTATTAATAAGAAG
TATTATATCATCTGGATCAGGAACTGGGTGAACTGTATACCCCCCCTTATCTTCAATTAT
ATACCACTCATCAATTTCAGTAGATTACACTATCTTTTCATTACATATTGCAGGAATTTC
ATCTATTATAGGAGCAATCAACTTTATTGTATCTATTTTACTTATAAAAAATATTTCAAT
TAATTATGATCAAATTCCTTTATTCCCATGATCAGTAAAAATTACTGCTATCTTATTACT
ATTATCTCTCCCAGTTTTAGCAGGAGCTATTACTATACTTTTAACAGATCGAAATTTAAA
TACTTCATTTTTTGACCCTTCTGGAGGAGGAG
>GU806803;tax=d:Eukarya,p:Arthropoda,c:Insecta,o:Hymenoptera,f:Halictidae,g:Lasioglossum,s:Lasioglossum lineatulum
AATACTTTACTTTATCTTTGCTATATGAGCCGGAATAATTGGAGCCTCATTAAGAATAAT
TATTCGAATAGAACTAAGTGCCCCTGGAAAATGAATTAATAATGATCAAATCTATAATAC
TATTATTACATCACACGCATTTGTAATAATTTTTTTTATAGTTATACCATTTATAATTGG
GGGATTTGGTAATTGATTAATTCCTTTAATAATTGGAGCTCCTGATATAGCATTCCCTCG
AATAAATAATATAAGATTTTGATTACTTATCCCATCAATATTTATATTATTAATAAGAAG
TATTATATCATCTGGTTCAGGAACTGGATGAACCGTATATCCTCCTTTATCATCTATTAT
ATACCACTCATCAATCTCTGTAGATTACACAATCTTTTCACTACATATTGCAGGAATTTC
ATCTATTATAGGAGCTATTAATTTCATTGTATCTATTTTACTAATAAAAAATATTTCAAT
TAATTATGATCAAATTCCTTTATTTCCATGATCAGTAAAAATTACTGCTATTTTATTATT
ATTATCTCTACCAGTTTTAGCAGGAGCTATTACTATACTTTTAACAGATCGAAACTTAAA
CACTTCATTTTTTGATCCATCAGGAGGAGGAGA
>HQ937902;tax=d:Eukarya,p:Arthropoda,c:Insecta,o:Hymenoptera,f:Halictidae,g:Lasioglossum,s:Lasioglossum lineatulum
ATGAGCCGGAATAATTGGAGCCTCATTAAGAATAATTATTCGAATAGAACTAAGTGCCCC
TGGAAAATGAATTAATAATGATCAAATCTATAATACTATTATTACATCACACGCATTTGT
AATAATTTTTTTTATAGTTATACCATTTATAATTGGGGGATTTGGTAATTGATTAATTCC
TTTAATAATTGGAGCTCCTGATATAGCATTCCCTCGAATAAATAATATAAGATTTTGATT
ACTTATCCCATCAATATTTATATTATTAATAAGAAGTATTATATCATCTGGTTCAGGAAC
TGGATGAACCGTATATCCTCCTTTATCATCTATTATATACCACTCATCAATCTCTGTAGA
TTACACAATCTTTTCACTACACATTGCAGGAATTTCATCTATTATAGGAGCTATCAATTT
CATTGTATCTATTTTACTAATAAAAAATATTTCAATTAATTATGATCAAATTCCTTTATT
TCCATGATCAGTAAAAATTACTGCTATTTTATTATTATTATCTCTACCAGTTTTAGCAGG
AGCTATTACTATACTTTTAACAGATCGAAACTTAAACACTTCATTTTTTGATCCATCAGG
AGGAGGAGATCCTATTCTTTATCAACATTTATTT
>KJ087917;tax=d:Eukarya,p:Arthropoda,c:Insecta,o:Hymenoptera,f:Halictidae,g:Lasioglossum,s:Lasioglossum lineatulum
TGGAAAATGAATTAATAATGATCAAATCTATAATACTATTATTACATCACACGCATTTGT
AATAATTTTTTTTATAGTTATACCATTTATAATTGGGGGATTTGGTAATTGATTAATTCC
TTTAATAATTGGAGCTCCTGATATAGCATTCCCTCGAATAAATAATATAAGATTTTGATT
ACTTATCCCATCAATATTTATATTATTAATAAGAAGTATTATATCATCTGGTTCAGGAAC
TGGATGAACCGTATATCCTCCTTTATCATCTATTATATACCACTCATCAATCTCTGTAGA

TTACACAATCTTTTCACTACACATTGCAGGAATTTCATCTATTATAGGAGCTATCAATTT
CATTGTATCTATTTTACTAATAAAAAATATTTCAATTAATTATGATCAAATTCCTTTATT
TCCATGATCAGTAAAAATTACTGCTATTTTATTATTATTATCTCTACCAGTTTTAGCAGG
AGCTATTACTATACTTTTAACAGATCGAAACTTAAACACTTCATTTTTTGATCCATCAGG
AGGAGGAGATCCTATTCTTTATCAACATTTATTT
>KJ092408;tax=d:Eukarya,p:Arthropoda,c:Insecta,o:Hymenoptera,f:Halictidae,g:Lasioglossum,s:Lasioglossum lineatulum
AATACTTTACTTTATCTTTGCTATATGAGCCGGAATAATTGGAGCCTCATTAAGAATAAT
TATTCGAATAGAACTAAGTGCCCCTGGAAAATGAATTAATAATGATCAAATCTATAATAC
TATTATTACATCACACGCATTTGTAATAATTTTTTTTATAGTTATACCATTTATAATTGG
GGGATTTGGTAATTGATTAATTCCTTTAATAATTGGAGCTCCTGATATAGCATTCCCTCG
AATAAATAATATAAGATTTTGATTACTTATCCCATCAATATTTATATTATTAATAAGAAG
TATTATATCATCTGGTTCAGGAACTGGATGAACCGTATATCCTCCTTTATCATCTATTAT
ATACCACTCATCAATCTCTGTAGATTACACAATCTTTTCACTACACATTGCAGGAATTTC
ATCTATTATAGGAGCTATCAATTTCATTGTATCTATTTTACTAATAAAAAATATTTCAAT
TAATTATGATCAAATTCCTTTATTTCCATGATCAGTAAAAATTACTGCTATTTTATTATT
ATTATCTCTACCAGTTTTAGCAGGAGCTATTACTATACTTTTAACAGATCGAAACTTAAA
CACTTCATTTTTTGATCCATCAGGAGGAGGAGATCCTATTCTTTATCAACATTTATTT
>KR783782;tax=d:Eukarya,p:Arthropoda,c:Insecta,o:Hymenoptera,f:Halictidae,g:Lasioglossum,s:Lasioglossum lineatulum
AATACTTTACTTTATCTTTGCTATATGAGCCGGAATAATTGGAGCCTCATTAAGAATAAT
TATTCGAATAGAACTAAGTGCCCCTGGAAAATGAATTAATAATGATCAAATCTATAATAC
TATTATTACATCACACGCATTTGTAATAATTTTTTTTATAGTTATACCATTTATAATTGG
AGGATTTGGTAATTGATTAATTCCTTTAATAATTGGAGCTCCTGATATAGCATTCCCTCG
AATAAATAATATAAGATTTTGATTACTTATCCCATCAATATTTATATTATTAATAAGAAG
TATTATATCATCTGGTTCAGGAACTGGATGAACCGTATATCCTCCTTTATCATCTATTAT
ATACCACTCATCAATCTCTGTAGATTACACAATCTTTTCACTACACATTGCAGGAATTTC
ATCTATTATAGGAGCTATTAATTTCATTGTATCTATTTTACTAATAAAAAATATTTCAAT
TAATTATGATCAAATTCCTTTATTTCCATGATCAGTAAAAATTACTGCTATTTTATTATT
ATTATCTCTACCAGTTTTAGCAGGAGCTATTACTATACTTTTAACAGATCGAAACTTAAA
CACTTCATTTTTTGATCCATCAGGAGGAGGAGATCCTATTCTTTATCAACATTTATTT
>KR787407;tax=d:Eukarya,p:Arthropoda,c:Insecta,o:Hymenoptera,f:Halictidae,g:Lasioglossum,s:Lasioglossum lineatulum
ATCTTTGCTATATGAGCCGGAATAATTGGAGCCTCATTAAGAATAATTATTCGAATAGAA
CTAAGTGCCCCTGGAAAATGAATTAATAATGATCAAATCTATAATACTATTATTACATCA
CACGCATTTGTAATAATTTTTTTTATAGTTATACCATTTATAATTGGAGGATTTGGTAAT
TGATTAATTCCTTTAATAATTGGAGCTCCTGATATAGCATTCCCTCGAATAAATAATATA
AGATTTTGATTACTTATCCCATCAATATTTATATTATTAATAAGAAGTATTATATCATCT
GGTTCAGGAACTGGATGAACCGTATATCCTCCTTTATCATCTATTATATACCACTCATCA
ATCTCTGTAGATTACACAATCTTTTCACTACACATTGCAGGAATTTCATCTATTATAGGA
GCTATTAATTTCATTGTATCTATTTTACTAATAAAAAATATTTCAATTAATTATGATCAA
ATTCCTTTATTTCCATGATCAGTAAAAATTACTGCTATTTTATTATTATTATCTCTACCA
GTTTTAGCAGGAGCTATTACTATACTTTTAACAGATCGAAACTTAAACACTTCATTTTTT
GATCCATCAGGAGGAGGAGATCCTATTCTTTATCAACATTTATTT
>KR792208;tax=d:Eukarya,p:Arthropoda,c:Insecta,o:Hymenoptera,f:Halictidae,g:Lasioglossum,s:Lasioglossum lineatulum
TCTTTGCTATATGAGCCGGAATAATTGGACCCTCATTAAGAATAATTATTCGAATAGAAC
TAAGTGCCCCTGGAAAATGAATTAATAATGATCAAATCTATAATACTATTATTACATCAC
ACGCATTTGTAATAATTTTTTTTATAGTTATACCATTTATAATTGGGGGATTTGGTAATT
GATTAATTCCTTTAATAATTGGAGCTCCTGATATAGCATTCCCTCGAATAAATAATATAA
GATTTTGATTACTTATCCCATCAATATTTATATTATTAATAAGAAGTATTATATCATCTG

GTTCAGGAACTGGATGAACCGTATATCCTCCTTTATCATCTATTATATACCACTCATCAA
TCTCTGTAGATTACACAATCTTTTCACTACATATTGCAGGAATTTCATCTATTATAGGAG
CTATTAATTTCATTGTATCTATTTTACTAATAAAAAATATTTCAATTAATTATGATCAAA
TTCCTTTATTTCCATGATCAGTAAAAATTACTGCTATTTTATTATTATTATCTCTACCAG
TTTTAGCAGGAGCTATTACTATACTTTTAACAGATCGAAACTTAAACACTTCATTTTTTG
ATCCATCAGGAGGAGGAGATCCTATTCTTTATCAACATTTATTT
>KR796677;tax=d:Eukarya,p:Arthropoda,c:Insecta,o:Hymenoptera,f:Halictidae,g:Lasioglossum,s:Lasioglossum lineatulum
AATTAATAATGATCAAATCTATAATACTATTATTACATCACACGCATTTGTAATAATTTT
TTTTATAGTTATACCATTTATAATTGGGGGATTTGGTAATTGATTAATTCCTTTAATAAT
TGGAGCTCCTGATATAGCATTCCCTCGAATAAATAATATAAGATTTTGATTACTTATCCC
ATCAATATTTATATTATTAATAAGAAGTATTATATCATCTGGTTCAGGAACTGGATGAAC
CGTATATCCTCCTTTATCATCTATTATATACCACTCATCAATCTCTGTAGATTACACAAT
CTTTTCACTACACATTGCAGGAATTTCATCTATTATAGGAGCTATCAATTTCATTGTATC
TATTTTACTAATAAAAAATATTTCAATTAATTATGATCAAATTCCTTTATTTCCATGATC
AGTAAAAATTACTGCTATTTTATTATTATTATCTCTACCAGTTTTAGCAGGAGCTATTAC
TATACTTTTAACAGATCGAAACTTAAACACTTCATTTTTTGATCCATCAGGAGGAGGAGA
TCCTATTCTTTATCAACATTTATTT
>KR875657;tax=d:Eukarya,p:Arthropoda,c:Insecta,o:Hymenoptera,f:Halictidae,g:Lasioglossum,s:Lasioglossum lineatulum
ATACTTTACTTTATCTTTGCTATATGAGCCGGAATAATTGGAGCCTCATTAAGAATAATT
ATTCGAATAGAACTAAGTGCCCCTGGAAAATGAATTAATAATGATCAAATCTATAATACT
ATTATTACATCACACGCATTTGTAATAATTTTTTTTATAGTTATACCATTTATAATTGGG
GGATTTGGTAATTGATTAATTCCTTTAATAATTGGAGCTCCTGATATAGCATTCCCTCGA
ATAAATAATATAAGATTTTGATTACTTATCCCATCAATATTTATATTATTAATAAGAAGT
ATTATATCATCTGGTTCAGGAACTGGATGAACCGTATATCCTCCTTTATCATCTATTATA
TACCACTCATCAATCTCTGTAGATTACACAATCTTTTCACTACATATTGCAGGAATTTCA
TCTATTATAGGAGCTATTAATTTCATTGTATCTATTTTACTAATAAAAAATATTTCAATT
AATTATGATCAAATTCCTTTATTTCCATGATCAGTAAAAATTACTGCTATTTTATTATTA
TTATCTCTACCAGTTTTAGCAGGA
>KR886254;tax=d:Eukarya,p:Arthropoda,c:Insecta,o:Hymenoptera,f:Halictidae,g:Lasioglossum,s:Lasioglossum lineatulum
ATACTTTACTTTATCTTTGCTATATGAGCCGGAATAATTGGAGCCTCATTAAGAATAATT
ATTCGAATAGAACTAAGTGCCCCTGGAAAATGAATTAATAATGATCAAATCTATAATACT
ATTATTACATCACACGCATTTGTAATAATTTTTTTTATAGTTATACCATTTATAATTGGG
GGATTTGGTAATTGATTAATTCCTTTAATAATTGGAGCTCCTGATATAGCATTCCCTCGA
ATAAATAATATAAGATTTTGATTACTTATCCCATCAATATTTATATTATTAATAAGAAGT
ATTATATCATCTGGTTCAGGAACTGGATGAACCGTATATCCTCCTTTATCATCTATTATA
TACCACTCATCAATCTCTGTAGATTACACAATCTTTTCACTACATATTGCAGGAATTTCA
TCTATTATAGGAGCTATTAATTTCATTGTATCTATTTTACTAATAAAAAATATTTCAATT
AATTATGATCAAATTCCTTTATTTCCATGATCAGTAAAAATTACTGCTATTTTATTATTA
TTATCTCTACCAGTTTTAGCAGGAGCTATTACTATACTTTTAACAGAT
>KR929783;tax=d:Eukarya,p:Arthropoda,c:Insecta,o:Hymenoptera,f:Halictidae,g:Lasioglossum,s:Lasioglossum lineatulum
TATGAGCCGGAATAATTGGAGCCTCATTAAGAATAATTATTCGAATAGAACTAAGTGCCC
CTGGAAAATGAATTAATAATGATCAAATCTATAATACTATTATTACATCACACGCATTTG
TAATAATTTTTTTTATAGTTATACCATTTATAATTGGAGGATTTGGTAATTGATTAATTC
CTTTAATAATTGGAGCTCCTGATATAGCATTCCCTCGAATAAATAATATAAGATTTTGAT
TACTTATCCCATCAATATTTATATTATTAATAAGAAGTATTATATCATCTGGTTCAGGAA
CTGGATGAACCGTATATCCTCCTTTATCATCTATTATATACCACTCATCAATCTCTGTAG
ATTACACAATCTTTTCACTACACATTGCAGGAATTTCATCTATTATAGGAGCTATTAATT

TCATTGTATCTATTTTACTAATAAAAAATATTTCAATTAATTATGATCAAATTCCTTTAT
TTCCATGATCAGTAAAAATTACTGCTATTTTATTATTATTATCTCTACCAGTTTTAGCAG
GAGCTATTACTATACTTTTAACAGATCGAAACTTAAACACTTCATTTTTTGATCCATCAG
GAGGAGGAGATCCTATTCTTTATCAACATTTATTT
>KR935126;tax=d:Eukarya,p:Arthropoda,c:Insecta,o:Hymenoptera,f:Halictidae,g:Lasioglossum,s:Lasioglossum lineatulum
AATTGGAGCCTCATTAAGAATAATTATTCGAATAGAACTAAGTGCCCCTGGAAAATGAAT
TAATAATGATCAAATCTATAATACTATTATTACATCACACGCATTTGTAATAATTTTTTT
TATAGTTATACCATTTATAATTGGGGGATTTGGTAATTGATTAATTCCTTTAATAATTGG
AGCTCCTGATATAGCATTCCCTCGAATAAATAATATAAGATTTTGATTACTTATCCCATC
AATATTTATATTATTAATAAGAAGTATTATATCATCTGGTTCAGGAACTGGATGAACCGT
ATATCCTCCTTTATCATCTATTATATACCACTCATCAATCTCTGTAGATTACACAATCTT
TTCACTACACATTGCAGGAATTTCATCTATTATAGGAGCTATCAATTTCATTGTATCTAT
TTTACTAATAAAAAATATTTCAATTAATTATGATCAAATTCCTTTATTTCCATGATCAGT
AAAAATTACTGCTATTTTATTATTATTATCTCTACCAGTTTTAGCAGGAGCTATTACTAT
ACTTTTAACAGATCGAAACTTAAACACTTCATTTTTTGATCCATCAGGAGGAGGAGATCC
TATTCTTTATCAACATTTATTT
>MF904285;tax=d:Eukarya,p:Arthropoda,c:Insecta,o:Hymenoptera,f:Halictidae,g:Lasioglossum,s:Lasioglossum lineatulum
ATACTTTACTTTATCTTTGCTATATGAGCCGGAATAATTGGAGCCTCATTAAGAATAATT
ATTCGAATAGAACTAAGTGCCCCTGGAAAATGAATTAATAATGATCAAATCTATAATACT
ATTATTACATCACACGCATTTGTAATAATTTTTTTATAGTTATACCATTTATAATTGGG
GGATTTGGTAATTGATTAATTCCTTTAATAATTGGAGCTCCTGATATAGCATTCCCTCGA
ATAAATAATATAAGATTTTGATTACTTATCCCATCAATATTTATATTATTAATAAGAAGT
ATTATATCATCTGGTTCAGGAACTGGATGAACCGTATATCCTCCTTTATCATCTATTATA
TACCACTCATCAATCTCTGTAGATTACACAATCTTTTCACTACACATTGCAGGAATTTCA
TCTATTATAGGAGCTATTAATTTCATTGTATCTATTTTACTAATAAAAAATATTTCAATT
AATTATGATCAAATTCCTTTATTTCCATGATCAGTAAAAATTACTGCTATTTTATTATTA
TTATCTCTACCAGTTTTAGCAGGAGCTATTACTATACTTTTAACAGAT
>MF907345;tax=d:Eukarya,p:Arthropoda,c:Insecta,o:Hymenoptera,f:Halictidae,g:Lasioglossum,s:Lasioglossum lineatulum
ATACTTTACTTTATCTTTGCTATATGAGCCGGAATAATTGGAGCCTCATTAAGAATAATT
ATTCGAATAGAACTAAGTGCCCCTGGAAAATGAATTAATAATGATCAAATCTATAATACT
ATTATTACATCACACGCATTTGTAATAATTTTTTTATAGTTATACCATTTATAATTGGA
GGATTTGGTAATTGATTAATTCCTTTAATAATTGGAGCTCCTGATATAGCATTCCCTCGA
ATAAATAATATAAGATTTTGATTACTTATCCCATCAATATTTATATTATTAATAAGAAGT
ATTATATCATCTGGTTCAGGAACTGGATGAACCGTATATCCTCCTTTATCATCTATTATA
TACCACTCATCAATCTCTGTAGATTACACAATCTTTTCACTACACATTGCAGGAATTTCA
TCTATTATAGGAGCTATTAATTTCATTGTATCTATTTTACTAATAAAAAATATTTCAATT
AATTATGATCAAATTCCTTTATTTCCATGATCAGTAAAAATTACTGCTATTTTATTATTA
TTATCTCTACCAGTTTTAGCAGGAGCTATTACTATA
>MG337014;tax=d:Eukarya,p:Arthropoda,c:Insecta,o:Hymenoptera,f:Halictidae,g:Lasioglossum,s:Lasioglossum lineatulum
ATACTTTACTTTATCTTTGCTATATGAGCCGGAATAATTGGAGCCTCATTAAGAATAATT
ATTCGAATAGAACTAAGTGCCCCTGGAAAATGAATTAATAATGATCAAATCTATAATACT
ATTATTACATCACACGCATTTGTAATAATTTTTTTATAGTTATACCATTTATAATTGGA
GGATTTGGTAATTGATTAATTCCTTTAATAATTGGAGCTCCTGATATAGCATTCCCTCGA
ATAAATAATATAAGATTTTGATTACTTATCCCATCAATATTTATATTATTAATAAGAAGT
ATTATATCATCTGGCTCAGGAACTGGATGAACCGTATATCCTCCTTTATCATCTATTATA
TACCACTCATCAATCTCTGTAGATTACACAATCTTTTCACTACACATTGCAGGAATTTCA
TCTATTATAGGAGCTATTAATTTCATTGTATCTATTTTACTAATAAAAAATATTTCAATT

AATTATGATCAAATTCCTTTATTTCCATGATCAGTAAAAATTACTGCTATTTTATTATTA
TTATCTCTACCAGTTTTAGCAGGAGCTATTACTATACTTTTAACA
>JF903543;tax=d:Eukarya,p:Arthropoda,c:Insecta,o:Hymenoptera,f:Halictidae,g:Lasioglossum,s:Lasioglossum nymphaearum
ATTTTCGCTATATGAGCCGGAATAATTGGAGCTTCATTAAGAATAATTATTCGAATAGAA
TTAAGTGCACCAGGAAAATGAATTAATAATGATCAAATTTATAATACTATTATTACTTCA
CATGCATTTGTAATAATTTTTTTTATAGTAATACCATTTATAATTGGTGGATTTGGAAAT
TGATTAATCCCCTTAATAATTGGGGCCCCTGATATAGCATTCCCTCGAATAAATAATATA
AGATTTTGATTACTTATTCCATCAATATTTATATTATTAATAAGAAGTATTATATCATCT
GGATCAGGAACTGGATGAACTGTATACCCCCCTCTATCTTCAATTATATACCACTCATCA
ATTTCAGTAGATTACACTATCTTTTCATTACATATCGCAGGAATTTCATCTATTATAGGA
GCTATTAATTTTATTGTATCTGTATTACTTATAAAAAATATTTCAATCAATTATGATCAA
ATTCCTTTATTTCCATGATCAGTAAAAATTACTGCTATTTTATTATTATTATCCCTGCCA
GTCCTAGCAGGAGCTATTACTATACTTTTAACAGATCGAAATTTAAATACTTCATTTTTC
GATCCTTCTGGAGGAGGAGACCCTATTCTTTATCAACACTTATTT
>JF903540;tax=d:Eukarya,p:Arthropoda,c:Insecta,o:Hymenoptera,f:Halictidae,g:Lasioglossum,s:Lasioglossum oblongum
TATACTTTATTTTATTTTCGCTATATGAGCTGGAATAATTGGAGCTTCATTAAGAATAAT
TATCCGAATAGAACTAAGTGCTCCTGGAAAATGAATTAATAATGATCAAATTTATAATAC
TATTATTACTTCACACGCATTTGTAATAATCTTTTTCATAGTTATACCATTTATAATTGG
AGGTTTTGGTAATTGATTGGTTCCTTTAATAATTGGAGCACCTGATATAGCATTCCCCCG
AATAAATAATATAAGATTTTGATTACTTATTCCATCAATATTTATATTATTAATAAGAAG
TATTATATCATCTGGCTCAGGAACTGGATGAACTGTATACCCACCTTTATCATCTATTAT
ATATCATTCCTCAATTTCAGTAGATTACACTATCTTTTCATTACACATTGCAGGAATTTC
ATCTATTATAGGAGCTATTAACTTCATTGTATCAATTTTACTTATAAAAAATATTTCAAT
TAATTATGATCAAATTCCTTTATTTCCATGATCAGTAAAAATTACTGCTATTTTATTATT
ATTATCTCTACCAGTTTTAGCAGGAGCTATTACTATACTTTTAACAGATCGAAATTTAAA
CACTTCATTTTTTGACCCTTCAGGAGGAGGAGACCCTATTCTTTATCAACATTTATTT
>KR790533;tax=d:Eukarya,p:Arthropoda,c:Insecta,o:Hymenoptera,f:Halictidae,g:Lasioglossum,s:Lasioglossum oblongum
TATACTTTATTTTATTTTCGCTATATGAGCTGGAATAATTGGAGCTTCATTAAGAATAAT
TATCCGAATAGAACTAAGTGCTCCTGGAAAATGAATTAATAATGATCAAATTTATAATAC
TATTATTACTTCACACGCATTTGTAATAATCTTTTTCATAGTTATACCATTTATAATTGG
AGGTTTTGGTAATTGATTGGTTCCTTTAATAATTGGAGCACCTGATATAGCATTCCCCCG
AATAAATAATATAAGATTTTGATTACTTATTCCATCAATATTTATATTATTAATAAGAAG
TATTATATCATCTGGCTCAGGAACTGGATGAACTGTATACCCACCTTTATCCTCTATTAT
ATATCATTCCTCAATTTCAGTAGATTACACTATCTTTTCATTACACATTGCAGGAATTTC
ATCTATTATAGGAGCTATTAACTTCATTGTATCAATTTTACTTATAAAAAATATTTCAAT
TAATTATGATCAAATTCCTTTATTTCCATGATCAGTAAAAATTACTGCTATTTTATTATT
ATTATCTCTACCAGTTTTAGCAGGAGCTATTACTATACTTTTAACAGATCGAAATTTAAA
TACTTCATTTTTTGACCCTTCGGGAGGAGGAGACCCTATTCTTTATCAACATTTATTT
>JF903541;tax=d:Eukarya,p:Arthropoda,c:Insecta,o:Hymenoptera,f:Halictidae,g:Lasioglossum,s:Lasioglossum obscurum
TATACTTTATTTTATTTTTGCTATATGAGCTGGAATAATTGGAGCTTCATTAAGAATAAT
TATTCGAATAGAACTAAGTGCTCCTGGAAAATGAATTAATAATGATCAAATTTATAACAC
TATTATTACCTCACACGCATTTGTAATAATTTTTTTTATAGTTATACCATTTATAATTGG
AGGTTTTGGTAATTGATTAGTTCCTTTAATAATTGGAGCACCTGATATAGCATTCCCCCG
AATAAATAATATAAGATTTTGATTACTTATTCCATCAATATTTATATTATTAATAAGAAG
TATTATATCATCTGGATCAGGAACTGGATGAACTGTATATCCTCCTTTATCATCTATTAT
ATATCATTCATCAATTTCAGTAGATTACACTATTTTTTCATTACACATTGCAGGAATTTC
ATCTATTATAGGAGCTATCAACTTCATTGTATCTATTTTACTTATAAAAAATATTTCAAT

TAATTATGATCAAATTCCATTATTTCCATGATCAGTAAAAATCACTGCTATTTTATTATT
ATTATCTCTTCCAGTTCTAGCAGGAGCTATTACTATACTTTTAACAGATCGAAATTTAAA
TACTTCATTTTTTGACCCTTCAGGAGGAGGGGATCCTATCCTTTATCAACATTT
>GU707990;tax=d:Eukarya,p:Arthropoda,c:Insecta,o:Hymenoptera,f:Halictidae,g:Lasioglossum,s:Lasioglossum paradmirandum
TATACTTTATTTTATTTTTGCTATATGAGCTGGGATAATTGGAGCCTCATTAAGAATAAT
TATTCGAATAGAACTAAGTGCTCCTGGAAAATGAATTAATAATGATCAAATTTATAATAC
TATTATTACCTCACATGCATTTGTAATAATTTTTTTTATAGTTATACCATTTATAATTGG
AGGATTTGGTAATTGATTAGTTCCTTTAATAATTGGAGCACCTGATATAGCTTTCCCCCG
AATAAATAATATAAGATTTTGATTACTTATTCCATCAATATTTATATTATTAATAAGAAG
TATTATATCATCTGGCTCAGGAACTGGATGAACTGTATACCCCCCTTTATCATCTATTAT
ATACCATTCATCAATTTCAGTAGATTACACTATTTTTTCATTGCACATTGCAGGAATTTC
ATCTATTATAGGAGCTATCAACTTCATTGTATCTATTTTACTTATAAAAAATATTTCAAT
TAATTACGATCAAATTCCCTTATTCCCATGATCAGTAAAAATTACTGCTATTTTATTATT
ATTATCTCTACCAGTTTTAGCAGGAGCCATTACTATACTTTTAACAGATCGAAACTTAAA
TACTTCATTTTTTGACCCTTCAGGAGGAGGAGACCCTATTCTTTATCAACATTTATTT
>JF903545;tax=d:Eukarya,p:Arthropoda,c:Insecta,o:Hymenoptera,f:Halictidae,g:Lasioglossum,s:Lasioglossum paradmirandum
ATACTTTATTTTATTTTTGCTATATGAGCTGGAATAATTGGAGCCTCATTAAGAATAATT
ATTCGAATAGAACTAAGTGCTCCTGGAAAATGAATTAATAATGATCAAATTTATAATACT
ATTATTACCTCACATGCATTTGTAATAATTTTTTTTATAGTTATACCATTTATAATTGGA
GGATTTGGTAATTGATTAGTTCCTTTAATAATTGGAGCACCTGATATAGCATTCCCCCGA
ATAAATAATATAAGATTTTGATTACTTATTCCATCAATATTTATATTATTAATAAGAAGT
ATTATATCATCTGGCTCAGGAACTGGATGAACTGTATACCCCCCTTTATCATCTATTATA
TACCATTCATCAATTTCAGTAGATTACACTATTTTTTCATTACACATTGCAGGAATTTCA
TCTATTATAGGAGCTATTAACTTCATTGTATCTATTTTACTTATAAAAAATATCTCAATT
AATTATGATCAAATTCCCTTATTCCCATGATCAGTAAAAATTACTGCTATTTTATTATTA
TTATCTCTACCAGTTTTAGCAGGAGCCATTACTATACTTTTAACAGATCGAAATTTAAAT
ACTTCATTTTTTGACCCTTCAGGAGGAGGAGACCCTATTCTTTATCAACATTTATTT
>KT123229;tax=d:Eukarya,p:Arthropoda,c:Insecta,o:Hymenoptera,f:Halictidae,g:Lasioglossum,s:Lasioglossum paradmirandum
GAACTAAGTGCTCCTGGAAAATGAATTAATAATGATCAAATTTATAATACTATTATTACC
TCACACGCATTTGTAATAATTTTTTTTATAGTTATACCATTTATAATTGGGGGGTTTGGT
AATTGATTAGTTCCTTTAATAATTGGAGCGCCTGATATAGCATTCCCCCGAATAAATAAT
ATAAGATTTTGATTACTTATTCCATCAATATTTATATTATTAATAAGAAGTATTATATCA
TCTGGCTCAGGAACTGGATGAACTGTATACCCCCCTTTATCATCTATTATATACCATTCA
TCAATTTCAGTAGATTACACTATTTTTTCATTACACATTGCAGGAATTTCATCTATTATA
GGAGCTATCAACTTCATTGGATCTATTTTACTTATAAAAAATATTTCAATTAATTATGAT
CAAATTCCCTTATTCCCATGATCAGTAAAAATTACTGCTATTTTATTATTATTATCTCTA
CCAGTTTTAGCAGGAGCCATTACTATACTTTTAACAGATCGAAATTTAAATACTTCATTT
TTTGACCCTTCAGGGGGAGGAGACCCTATTCTTTATCAACATTTATTT
>KJ084591;tax=d:Eukarya,p:Arthropoda,c:Insecta,o:Hymenoptera,f:Halictidae,g:Lasioglossum,s:Lasioglossum pictum
AATACTTTATTTTATATTTGCAATATGATCGGGAATAATTGGTGCTTCATTAAGAATAAT
TATTCGTATAGAATTAAGAGCTCCAGGAAAATGAATTAATAATGATCAAATTTATAATAC
TATTATTACTTCTCATGCATTTGTAATAATTTTTTTTATAGTTATACCTTTTATAATTGG
AGGATTTGGAAATTGATTAGTTCCTTTAATAATTGGAGCCCCTGATATAGCATTTCCTCG
AATAAATAATATAAGATTTTGATTACTTATCCCATCAATATTTATATTATTAATAAGAAG
AATTATATCATCAGGTTCAGGAACTGGATGAACTGTATACCCCCCTTTATCTTCTATTAT
ATATCATTCATCAATTTCTGTAGATTATACTATTTTTTCACTTCATATTGCAGGAATTTC
ATCAATTATAGGAGCTATTAATTTTATTGTATCTATCCTTCTAATAAAAAATATTTCAAT

TAATTTTGATCAAATTCCTTTATTTCCATGATCAGTAAAAATTACTGCTATTTTACTATT
ATTATCTCTTCCAGTATTAGCAGGAGCTATCACTATACTTTTAACTGATCGAAATTTAAA
TACTTCCTTTTTTGACCCTTCTGGTGGAGGAG
>KJ084813;tax=d:Eukarya,p:Arthropoda,c:Insecta,o:Hymenoptera,f:Halictidae,g:Lasioglossum,s:Lasioglossum pictum
AATACTTTATTTTATATTTGCAATATGATCGGGAATAATTGGTGCTTCATTAAGAATAAT
TATTCGTATAGAATTAAGAGCTCCAGGAAAATGAATTAATAATGATCAAATTTATAATAC
TATTATTACTTCTCATGCATTTGTAATAATTTTTTTTATAGTTATACCTTTTATAATTGG
AGGATTTGGAAATTGATTAGTTCCTTTAATAATTGGAGCCCCTGATATAGCATTTCCTCG
AATAAATAATATAAGATTTTGATTACTTATCCCATCAATATTTATATTATTAATAAGAAG
AATTATATCATCAGGTTCAGGAACTGGATGAACTGTATACCCCCCTTTATCTTCTATTAT
ATATCATTCATCAATTTCTGTAGATTATACTATTTTTTCACTTCATATTGCAGGAATTTC
ATCAATTATAGGAGCTATTAATTTTATTGTATCTATCCTTCTAATAAAAAATATTTCAAT
TAATTTTGATCAAATTCCTTTATTTCCATGATCAGTAAAAATTACTGCTATTTTACTATT
ATTATCTCTTCCAGTATTAGCAGGAGCTATCACTATACTTTTAACTGATC
>KJ086799;tax=d:Eukarya,p:Arthropoda,c:Insecta,o:Hymenoptera,f:Halictidae,g:Lasioglossum,s:Lasioglossum pictum
AATACTTTATTTTATATTTGCAATATGATCGGGAATAATTGGTGCTTCATTAAGAATAAT
TATTCGTATAGAATTAAGAGCTCCAGGAAAATGAATTAATAATGATCAAATTTATAATAC
TATTATTACTTCTCATGCATTTGTAATAATTTTTTTTATAGTTATACCTTTTATAATTGG
AGGATTTGGAAATTGATTAGTTCCTTTAATAATTGGAGCCCCTGATATAGCATTTCCTCG
AATAAATAATATAAGATTTTGATTACTTATCCCATCAATATTTATATTATTAATAAGAAG
AATTATATCATCAGGTTCAGGAACTGGATGAACTGTATACCCCCCTTTATCTTCTATTAT
ATATCATTCATCAATTTCTGTAGATTATACTATTTTTTCACTTCATATTGCAGGAATTTC
ATCAATTATAGGAGCTATTAATTTTATTGTATCTATCCTTCTAATAAAAAATATTTCAAT
TAATTTTGATCAAATTCCTTTATTTCCATGATCAGTAAAAATTACTGCTATTTTACTATT
ATTATCTCTTCCAGTATTAGCAGGAGCTATCACTATACTTTTAACTGATCGAAATTTAAA
TACTTCCTTTTTTGACCCTTCT
>KJ089105;tax=d:Eukarya,p:Arthropoda,c:Insecta,o:Hymenoptera,f:Halictidae,g:Lasioglossum,s:Lasioglossum pictum
AATACTTTATTTTATATTTGCAATATGATCGGGAATAATTGGTGCTTCATTAAGAATAAT
TATTCGTATAGAATTAAGAGCTCCAGGAAAATGAATTAATAATGATCAAATTTATAATAC
TATTATTACTTCTCATGCATTTGTAATAATTTTTTTTATAGTTATACCTTTTATAATTGG
AGGATTTGGAAATTGATTAGTTCCTTTAATAATTGGAGCCCCTGATATAGCATTTCCTCG
AATAAATAATATAAGATTTTGATTACTTATCCCATCAATATTTATATTATTAATAAGAAG
AATTATATCATCAGGTTCAGGAACTGGATGAACTGTATACCCCCCTTTATCTTCTATTAT
ATATCATTCATCAATTTCTGTAGATTATACTATTTTTTCACTTCATATTGCAGGAATTTC
ATCAATTATAGGAGCTATTAATTTTATTGTATCTATCCTTCTAATAAAAAATATTTCAAT
TAATTTTGATCAAATTCCTTTATTTCCATGATCAGTAAAAATTACTGCTATTTTACTATT
ATTATCTCTTCCAGTATTAGCAGGAGCTATCACTATACTTTTAACTGATCGAAATTTAAA
TACTTCCTTTTTTGA
>KJ090045;tax=d:Eukarya,p:Arthropoda,c:Insecta,o:Hymenoptera,f:Halictidae,g:Lasioglossum,s:Lasioglossum pictum
AATACTTTATTTTATATTTGCAATATGATCGGGAATAATTGGTGCTTCATTAAGAATAAT
TATTCGTATAGAATTAAGAGCTCCAGGAAAATGAATTAATAATGATCAAATTTATAATAC
TATTATTACTTCTCATGCATTTGTAATAATTTTTTTTATAGTTATACCTTTTATAATTGG
AGGATTTGGAAATTGATTAGTTCCTTTAATAATTGGAGCCCCTGATATAGCATTTCCTCG
AATAAATAATATAAGATTTTGATTACTTATCCCATCAATATTTATATTATTAATAAGAAG
AATTATATCATCAGGTTCAGGAACTGGATGAACTGTATACCCCCCTTTATCTTCTATTAT
ATATCATTCATCAATTTCTGTAGATTATACTATTTTTTCACTTCATATTGCAGGAATTTC
ATCAATTATAGGAGCTATTAATTTTATTGTATCTATCCTTCTAATAAAAAATATTTCAAT

TAATTTTGATCAAATTCCTTTATTTCCATGATCAGTAAAAATTACTGCTATTTTACTATT
ATTATCTCTTCCAGTATTAGCAGGAGCTATCACTATACTTTTAACTGATCGAAATTTAAA
TACTTCCTTTTTTGACCC
>KJ091646;tax=d:Eukarya,p:Arthropoda,c:Insecta,o:Hymenoptera,f:Halictidae,g:Lasioglossum,s:Lasioglossum
pictum
AATACTTTATTTTATATTTGCAATATGATCGGGAATAATTGGTGCTTCATTAAGAATAAT
TATTCGTATAGAATTAAGAGCTCCAGGAAAATGAATTAATAATGATCAAATTTATAATAC
TATTATTACTTCTCATGCATTTGTAATAATTTTTTTTATAGTTATACCTTTTATAATTGG
AGGATTTGGAAATTGATTAGTTCCTTTAATAATTGGAGCCCCTGATATAGCATTTCCTCG
AATAAATAATATAAGATTTTGATTACTTATCCCATCAATATTTATATTATTAATAAGAAG
AATTATATCATCAGGTTCAGGAACTGGATGAACTGTATACCCCCCTTTATCTTCTATTAT
ATATCATTCATCAATTTCTGTAGATTATACTATTTTTTCACTTCATATTGCAGGAATTTC
ATCAATTATAGGAGCTATTAATTTTATTGTATCTATCCTTCTAATAAAAAATATTTCAAT
TAATTTTGATCAAATTCCTTTATTTCCATGATCAGTAAAAATTACTGCTATTTTACTATT
ATTATCTCTTCCAGTATTAGCAGGAGCTATCACTATACTTTTAACTGATCGAAATTTAAA
TACTTCCTTTTTTGACCCTTC
>KJ092667;tax=d:Eukarya,p:Arthropoda,c:Insecta,o:Hymenoptera,f:Halictidae,g:Lasioglossum,s:Lasioglossum
pictum
AATACTTTATTTTATATTTGCAATATGATCGGGAATAATTGGTGCTTCATTAAGAATAAT
TATTCGTATAGAATTAAGAGCTCCAGGAAAATGAATTAATAATGATCAAATTTATAATAC
TATTATTACTTCTCATGCATTTGTAATAATTTTTTTTATAGTTATACCTTTTATAATTGG
AGGATTTGGAAATTGATTAGTTCCTTTAATAATTGGAGCCCCTGATATAGCATTTCCTCG
AATAAATAATATAAGATTTTGATTACTTATCCCATCAATATTTATATTATTAATAAGAAG
AATTATATCATCAGGTTCAGGAACTGGATGAACTGTATACCCCCCTTTATCTTCTATTAT
ATATCATTCATCAATTTCTGTAGATTATACTATTTTTTCACTTCATATTGCAGGAATTTC
ATCAATTATAGGAGCTATTAATTTTATTGTATCTATCCTTCTAATAAAAAATATTTCAAT
TAATTTTGATCAAATTCCTTTATTTCCATGATCAGTAAAAATTACTGCTATTTTACTATT
ATTATCTCTTCCAGTATTAGCAGGAGCTATCACTATACTTTTAACTGATCGAAATTTAAA
TACTTCCTTTTTTGACCCTTCTGGTGGAGGAGATCC
>KJ163110;tax=d:Eukarya,p:Arthropoda,c:Insecta,o:Hymenoptera,f:Halictidae,g:Lasioglossum,s:Lasioglossum
pictum
AATACTTTATTTTATATTTGCAATATGATCGGGAATAATTGGTGCTTCATTAAGAATAAT
TATTCGTATAGAATTAAGAGCTCCAGGAAAATGAATTAATAATGATCAAATTTATAATAC
TATTATTACTTCTCATGCATTTGTAATAATTTTTTTTATAGTTATACCTTTTATAATTGG
AGGATTTGGAAATTGATTAGTTCCTTTAATAATTGGAGCCCCTGATATAGCATTTCCTCG
AATAAATAATATAAGATTTTGATTACTTATCCCATCAATATTTATATTATTAATAAGAAG
AATTATATCATCAGGTTCAGGAACTGGATGAACTGTATACCCCCCTTTATCTTCTATTAT
ATATCATTCATCAATTTCTGTAGATTATACTATTTTTTCACTTCATATTGCAGGAATTTC
ATCAATTATAGGAGCTATTAATTTTATTGTATCTATCCTTCTAATAAAAAATATTTCAAT
TAATTTTGATCAAATTCCTTTATTTCCATGATCAGTAAAAATTACTGCTATTTTACTATT
ATTATCTCTTCCAGTATTAGCAGGAGCTATCACTATACTTTTAACTGATCGAAATTTAAA
TACTTCCTTTTTTGACCCTTCTGGTGGAGGAGATCCTATTCTTTATCAACATTTATTT
>KJ164586;tax=d:Eukarya,p:Arthropoda,c:Insecta,o:Hymenoptera,f:Halictidae,g:Lasioglossum,s:Lasioglossum
pictum
AATACTTTATTTTATATTTGCAATATGATCGGGAATAATTGGTGCTTCATTAAGAATAAT
TATTCGTATAGAATTAAGAGCTCCAGGAAAATGAATTAATAATGATCAAATTTATAATAC
TATTATTACTTCTCATGCATTTGTAATAATTTTTTTTATAGTTATACCTTTTATAATTGG
AGGATTTGGAAATTGATTAGTTCCTTTAATAATTGGAGCCCCTGATATAGCATTTCCTCG
AATAAATAATATAAGATTTTGATTACTTATCCCATCAATATTTATATTATTAATAAGAAG
AATTATATCATCAGGTTCAGGAACTGGATGAACTGTATACCCCCCTTTATCTTCTATTAT
ATATCATTCATCAATTTCTGTAGATTATACTATTTTTTCACTTCATATTGCAGGAATTTC

ATCAATTATAGGAGCTATTAATTTTATTGTATCTATCCTTCTAATAAAAAATATTTCAAT
TAATTTTGATCAAATTCCTTTATTTCCATGATCAGTAAAAATTACTGCTATTTTACTATT
ATTATCTCTTCCAGTATTAGCAGGAGCTATCACTATACTTTTAACTGATCGAAATTTAAA
TACTTCCTTTTTTGACCCTTCTGGTGGA
>JF903550;tax=d:Eukarya,p:Arthropoda,c:Insecta,o:Hymenoptera,f:Halictidae,g:Lasioglossum,s:Lasioglossum platyparium
ATACTTTATTTTATTTTTGCTATATGAGCTGGAATAATTGGAGCTTCATTAAGAATAATT
ATTCGAATAGAATTAAGTGCTCCCGGAAAATGAATTAATAATGATCAAATTTATAATACT
ATTATTACATCTCATGCATTTGTAATAATTTTTTTTATAGTTATACCATTTATAATTGGT
GGTTTTGGTAATTGATTAATTCCTTTAATAATTGGAGCACCTGATATAGCATTCCCCCGA
ATAAATAATATAAGATTTTGATTACTTATCCCATCAATATTTATATTATTAATAAGAAGA
ATTATATCATCTGGTTCAGGAACTGGATGAACTGTATATCCTCCTTTATCATCTATTATA
TATCATTCATCAATTTCAGTAGATTACACTATCTTTTCATTACATATTGCAGGAATTTCA
TCTATTATAGGAGCTATTAACTTTATTGTATCTATTTTACTTATAAAAAATATTTCAATT
AATTATGATCAAATTCCTTTATTTCCATGATCAGTAAAAATTACTGCTATTTTATTATTA
TTATCTCTACCAGTTTTAGCAGGAGCTATTACTATACTTCTAACAGATCGAAATTTAAAC
ACTTCATTTTTTGACCCCTCAGGAGGAGGAGATCCTATTCTTTATCAACATTTATTT
>KR785337;tax=d:Eukarya,p:Arthropoda,c:Insecta,o:Hymenoptera,f:Halictidae,g:Lasioglossum,s:Lasioglossum platyparium
TAATTGGAGCTTCATTAAGAATAATTATTCGAATAGAATTAAGTGCTCCCGGAAAATGAA
TTAATAATGATCAAATTTATAATACTATTATTACATCTCATGCATTTGTAATAATTTTTT
TTATAGTTATACCATTTATAATTGGTGGTTTTGGTAATTGATTAATTCCTTTAATAATTG
GAGCACCTGATATAGCATTCCCCCGAATAAATAATATAAGATTTTGATTACTTATCCCAT
CAATATTTATATTATTAATAAGAAGAATTATATCATCTGGTTCAGGAACTGGATGAACTG
TATATCCTCCTTTATCATCTATTATATATCATTCATCAATTTCAGTAGATTACACTATCT
TTTCATTACATATTGCAGGAATTTCATCTATTATAGGAGCTATTAACTTTATTGTATCTA
TTTTACTTATAAAAAATATTTCAATTAATTATGATCAAATTCCTTTATTTCCATGGTCAG
TAAAAATTACTGCTATTTTATTATTATTATCTCTACCAGTTTTAGCAGGAGCTATTACTA
TACTTCTAACAGATCGAAATTTAAATACTTCATTTTTTGATCCCTCAGGAGGAGGAGATC
CTATTCTTTATCAACATTTATTT
>JF903552;tax=d:Eukarya,p:Arthropoda,c:Insecta,o:Hymenoptera,f:Halictidae,g:Lasioglossum,s:Lasioglossum pruinosum
AATACTTTATTTTATTTTTGCTATATGAGCTGGAATAATTGGAGCTTCATTAAGAATAAT
TATTCGAATAGAATTAAGTGCCCCAGGAAAATGAATTAATAATGATCAAATTTATAACAC
TATTATTACTTCACATGCATTTGTAATAATTTTTTTTATAGTTATACCATTTATAATTGG
AGGATTTGGTAATTGATTAATTCCTTTAATAATTGGTGCCCCCGATATAGCATTCCCTCG
AATAAATAATATAAGATTTTGACTATTAATTCCATCAATATTTATATTATTAATAAGAAG
TATTATATCATCTGGATCAGGTACTGGATGAACTGTTTACCCACCTTTATCTTCAATTAT
ATACCACTCATCAATTTCAGTAGATTATACTATTTTTTCATTACATATTGCAGGAATTTC
ATCAATTATAGGAGCTATTAATTTTATTGTATCTATCCTTCTTATAAAAAATATTTCAAT
TAATTATGATCAAATCCCCTTATTTCCATGATCAGTAAAAATTACTGCTATTTTATTATT
ATTATCTTTACCAGTTTTAGCAGGAGCTATTACTATACTTTTAACAGATCGAAATTTAAA
CACTTCATTTTTTGACCCTTCTGGAGGAGGAGACCCTATTCTTTATCAACATTTATTT
>KR881012;tax=d:Eukarya,p:Arthropoda,c:Insecta,o:Hymenoptera,f:Halictidae,g:Lasioglossum,s:Lasioglossum pruinosum
AATACTTTATTTTATTTTTGCTATATGAGCTGGAATAATTGGAGCTTCATTAAGAATAAT
TATTCGAATAGAATTAAGTGCCCCAGGAAAATGAATTAATAATGATCAAATTTATAACAC
TATTATTACTTCACATGCATTTGTAATAATTTTTTTTATAGTTATACCATTTATAATTGG
AGGATTTGGTAATTGATTAATTCCTTTAATAATTGGTGCCCCCGATATAGCATTCCCTCG
AATAAATAATATAAGATTTTGACTATTAATTCCATCAATATTTATATTATTAATAAGAAG
TATTATATCATCTGGATCAGGTACTGGATGAACTGTTTACCCACCTTTATCTTCAATTAT

ATACCACTCATCAATTTCAGTAGATTACACTATTTTTTCATTACATATTGCAGGAATTTC
ATCAATTATAGGAGCTATTAATTTTATTGTATCTATCCTTCTTATAAAAAATATTTCAAT
TAATTATGATCAAATCCCCTTATTTCCATGATCAGTAAAAATTACTGCTATTTTATTATT
ATTATCTTTA
>KT123231;tax=d:Eukarya,p:Arthropoda,c:Insecta,o:Hymenoptera,f:Halictidae,g:Lasioglossum,s:Lasioglossum pruinosum
ATAGAATTAAGTGCCCCAGGAAAATGAATTAATAATGATCAAATTTATAACACTATTATT
ACTTCACATGCATTTGTAATAATTTTTTTATAGTTATACCATTTATAATTGGAGGATTT
GGTAATTGATTAATTCCTTTAATAATTGGTGCCCCCGATATAGCATTCCCTCGAATAAAT
AATATAAGATTTTGATTATTAATTCCATCAATATTTATATTATTAATAAGAAGTATTATA
TCATCTGGATCAGGTACTGGATGAACTATTTACCCACCTTTATCTTCAATTATATACCAC
TCATCAATTTCAGTAGATTACACTATTTTTTCATTACATATTGCAGGAATTTCATCAATT
ATAGGAGCTATTAATTTTATTGTATCTATCCTTCTTATAAAAAATATTTCAATTAATTAT
GATCAAATCCCCTTATTTCCATGATCAGTAAAAATTACTGCTATTTTATTATTATTATCT
TTACCAGTTTTAGCAGGAGCTATTACTATACTTTTAACAGATCGAAATTTAAACACTTCA
TTTTTTGACCCTTCTGGAGGAGGAGACCCTATTCTTTATCAACATTTATTT
>HQ558150;tax=d:Eukarya,p:Arthropoda,c:Insecta,o:Hymenoptera,f:Halictidae,g:Lasioglossum,s:Lasioglossum semicaeruleum
AATACTTTACTTTATTTTTGCAATATGAGCAGGAATAATTGGAGCTTCCTTAAGAATAAT
TATTCGAATAGAATTAAGTGCACCTGGAAAATGAATTAATAATGATCAAATTTATAATAC
TATTATTACCTCTCATGCATTTGTAATAATTTTTTTATAGTTATACCATTTATAATTGG
AGGATTTGGAAATTGATTAGTTCCCTTAATAATTGGTGCTCCTGATATAGCATTTCCTCG
AATAAATAATATAAGATTCTGATTACTTATCCCATCAATATTTATATTATTAATAAGAAG
ATTTATTTCATCAGGTTCAGGGACTGGGTGAACTGTTTATCCTCCCTTATCTTCAATCAT
ATATCATTCATCAATTTCTGTAGATTACACTATTTTTTCTTTACATATTGCGGGAATTTC
ATCCATTATAGGAGCTATTAATTTTATTGTATCTATTATACTTATAAAAAATATTTCAAT
TAATTATGATCAAATTCCTTTATTTCCATGATCAGTAAAAATTACTGCTATTCTATTATT
ATTATCTCTTCCAGTATTAGCAGGAGCTATTACTATACTTTTAACAGACCGAAACTTAAA
TACTTCATTTTTCGATCCTTCTGGAGGAGGAGACCCAATTCTTTATCAACATTTATTT
>HQ929841;tax=d:Eukarya,p:Arthropoda,c:Insecta,o:Hymenoptera,f:Halictidae,g:Lasioglossum,s:Lasioglossum semicaeruleum
AATACTTTCTTTTATTTTTGCAATATGAGCAGGAATAATTGGAGCTTCCTTAAGAATAAT
TATTCGAATAGAATTAAGTGCACCTGGAAAATGAATTAATAATGATCAAATTTATAATAC
TATTATTACTTCTCATGCATTTGTAATAATTTTTTTATAGTTATACCATTTATAATTGG
AGGATTTGGAAATTGATTAGTTCCCTTAATAATTGGTGCCCCCGATATAGCATTTCCTCG
AATAAATAATATAAGATTCTGATTACTTATTCCATCAATATTTATATTATTAATAAGAAG
ATTTATTTCATCAGGTTCAGGAACTGGATGAACTGTTTACCCTCCCTTATCTTCAATCAT
ATACCATTCATCAATTTCTGTAGATTACACTATTTTTTCTCTACATATTGCAGGAATTTC
ATCCATTATAGGAGCTATTAATTTTATTGTATCTATTATACTTATAAAAAATATTTCAAT
TAATTATGATCAAATTCCTTTATTTCCATGATCAGTAAAAATTACTGCTATTCTATTATT
ATTATCTCTTCCAGTATTAGCAGGAGCTATTACTATACTTTTAACAGACCGAAATTTAAA
TACTTCATTTTTTGATCCTTCAGGAGGAGGAGA
>JF903557;tax=d:Eukarya,p:Arthropoda,c:Insecta,o:Hymenoptera,f:Halictidae,g:Lasioglossum,s:Lasioglossum semicaeruleum
AATACTTTACTTTATTTTTGCAATATGAGCAGGAATAATTGGAGCTTCCTTAAGAATAAT
TATTCGAATAGAATTAAGTGCACCTGGAAAATGAATTAATAATGATCAAATTTATAATAC
TATTATTACCTCTCATGCATTTGTAATAATTTTTTTATAGTTATACCATTTATAATTGG
AGGATTTGGAAATTGATTAGTTCCCTTAATAATTGGTGCTCCTGATATAGCATTTCCTCG
AATAAATAATATAAGATTCTGATTACTTATCCCATCAATATTTATATTATTAATAAGAAG
ATTTATTTCATCAGGTTCAGGGACTGGGTGAACTGTTTATCCTCCCTTATCTTCAATCAT
ATATCATTCATCAATTTCTGTAGATTACACTATTTTTTCTTTACATATTGCGGGAATTTC

ATCCATTATAGGAGCTATTAATTTTATTGTATCTATTATACTTATAAAAAATATTTCAAT
TAATTATGATCAAATTCCTTTATTTCCATGATCAGTAAAAATTACTGCTATTCTATTATT
ATTATCTCTTCCAGTATTAGCAGGAGCTATTACTATACTTTTAACAGACCGAAACTTAAA
TACTTCATTTTTCGATCCTTCTGGAGGAGGAGACCCAATTCTTTATCAACATTTATT
>KR783894;tax=d:Eukarya,p:Arthropoda,c:Insecta,o:Hymenoptera,f:Halictidae,g:Lasioglossum,s:Lasioglossum semicaeruleum
AATACTTTACTTTATTTTTGCAATATGAGCAGGAATAATTGGAGCTTCCTTAAGAATAAT
TATTCGAATAGAATTAAGTGCACCTGGAAAATGAATTAATAATGATCAAATTTATAATAC
TATTATTACCTCTCATGCATTTGTAATAATTTTTTTTATAGTTATACCATTTATAATTGG
AGGATTTGGAAATTGATTAGTTCCCTTAATAATTGGTGCTCCTGATATAGCATTTCCTCG
AATAAATAATATAAGATTCTGATTACTTATCCCATCAATATTTATATTATTAATAAGAAG
ATTTATTTCATCAGGTTCAGGGACTGGGTGAACTGTTTATCCTCCCTTATCTTCAATCAT
ATATCATTCATCAATTTCTGTAGATTACACTATTTTTTCTTTACATATTGCGGGAATTTC
ATCCATTATAGGAGCTATTAATTTTATTGTATCTATTATACTTATAAAAAATATTTCAAT
TAATTATGATCAAATTCCTTTATTTCCATGATCAGTAAAAATTACTGCTATTC
>KR790919;tax=d:Eukarya,p:Arthropoda,c:Insecta,o:Hymenoptera,f:Halictidae,g:Lasioglossum,s:Lasioglossum semicaeruleum
AATACTTTACTTTATTTTTGCAATATGAGCAGGAATAATTGGAGCTTCCTTAAGAATAAT
TATTCGAATAGAATTAAGTGCACCTGGAAAATGAATTAATAATGATCAAATTTATAATAC
TATTATTACCTCTCATGCATTTGTAATAATTTTTTTTATAGTTATACCATTTATAATTGG
AGGATTTGGAAATTGATTAGTTCCCTTAATAATTGGTGCTCCTGATATAGCATTTCCTCG
AATAAATAATATAAGATTCTGATTACTTATCCCATCAATATTTATATTATTAATAAGAAG
ATTTATTTCATCAGGTTCAGGGACTGGGTGAACTGTTTATCCTCCCTTATCTTCAATCAT
ATATCATTCATCAATTTCTGTAGATTACACTATTTTTTCTTTACATATTGCGGGAATTTC
ATCCATTATAGGAGCTATTAATTTTATTGTATCTATTATACTTATAAAAAATATTTCAAT
TAATTATGATCAAATTCCTTTATTTCCATGATCAGTAAAAATTACTGCTATTCTATTATT
ATTATCTCTTCCAGTATTAGCAGGAGCTATTACTATACTTTTAACAGACCGAAACTTAAA
TACTTCATTTTTCGATCCTTCTGGAGGAGGAGA
>KR881556;tax=d:Eukarya,p:Arthropoda,c:Insecta,o:Hymenoptera,f:Halictidae,g:Lasioglossum,s:Lasioglossum semicaeruleum
AATACTTTACTTTATTTTTGCAATATGAGCAGGAATAATTGGAGCTTCCTTAAGAATAAT
TATTCGAATAGAATTAAGTGCACCTGGAAAATGAATTAATAATGATCAAATTTATAATAC
TATTATTACCTCTCATGCATTTGTAATAATTTTTTTTATAGTTATACCATTTATAATTGG
AGGATTTGGAAATTGATTAGTTCCCTTAATAATTGGTGCTCCTGATATAGCATTTCCTCG
AATAAATAATATAAGATTCTGATTACTTATCCCATCAATATTTATATTATTAATAAGAAG
ATTTATTTCATCAGGTTCAGGGACTGGGTGAACTGTTTATCCTCCCCTATCTTCAATCAT
ATATCATTCATCAATTTCTGTAGATTACACTATTTTTTCTTTACATATTGCGGGAATTTC
ATCCATTATAGGAGCTATTAATTTTATTGTATCTATTATACTTATAAAAAATATTTCAAT
TAATTATGATCAAATTCCTTTATTTCCATGATCAGTAAAAATTACTGCTATTCTATTATT
ATTATCTCTTCCAGTATTAGCAGGAGCTATTACTATACTTTTAACAGACCGAAACTTAAA
TACTTCATTT
>KR882052;tax=d:Eukarya,p:Arthropoda,c:Insecta,o:Hymenoptera,f:Halictidae,g:Lasioglossum,s:Lasioglossum semicaeruleum
AATACTTTACTTTATTTTTGCAATATGAGCAGGAATAATTGGAGCTTCCTTAAGAATAAT
TATTCGAATAGAATTAAGTGCACCTGGAAAATGAATTAATAATGATCAAATTTATAATAC
TATTATTACCTCTCATGCATTTGTAATAATTTTTTTTATAGTTATACCATTTATAATTGG
AGGATTTGGAAATTGATTAGTTCCCTTAATAATTGGTGCTCCTGATATAGCATTTCCTCG
AATAAATAATATAAGATTCTGATTACTTATCCCATCAATATTTATATTATTAATAAGAAG
ATTTATTTCATCAGGTTCAGGGACTGGGTGAACTGTTTATCCTCCCTTATCTTCAATCAT
ATATCATTCATCAATTTCTGTAGATTACACTATTTTTTCTTTACATATTGCGGGAATTTC
ATCCATTATAGGAGCTATTAATTTTATTGTATCTATTATACTTATAAAAAATATTTCAAT

TAATTATGATCAAATTCCTTTATTTCCATGATCAGTAAAAATTACTGCTATTCTATTATT
ATTATCTCTTCCAGTATTAGCAGGAGCTATTA
>KR884325;tax=d:Eukarya,p:Arthropoda,c:Insecta,o:Hymenoptera,f:Halictidae,g:Lasioglossum,s:Lasioglossum semicaeruleum
AATACTTTACTTTATTTTTGCAATATGAGCAGGAATAATTGGAGCTTCCTTAAGAATAAT
TATTCGAATAGAATTAAGTGCACCTGGAAAATGAATTAATAATGATCAAATTTATAATAC
TATTATTACCTCTCATGCATTTGTAATAATTTTTTTTATAGTTATACCATTTATAATTGG
AGGATTTGGAAATTGATTAGTTCCCTTAATAATTGGTGCTCCTGATATAGCATTTCCTCG
AATAAATAATATAAGATTCTGATTACTTATCCCATCAATATTTATATTATTAATAAGAAG
ATTTATTTCATCAGGTTCAGGGACTGGGTGAACTGTTTATCCTCCCTTATCTTCAATCAT
ATATCATTCATCAATTTCTGTAGATTACACTATTTTTTCTTTACATATTGCGGGAATTTC
ATCCATTATAGGAGCTATTAATTTTATTGTATCTATTATACTTATAAAAAATATTTCAAT
TAATTATGATCAAATTCCTTTATTTCCATGATCAGTAAAAATTACTGCTATTCTATTATT
ATTATCTCTTCCAGTATTAGCAGGAGCTATTACTATACTTTTA
>KR886573;tax=d:Eukarya,p:Arthropoda,c:Insecta,o:Hymenoptera,f:Halictidae,g:Lasioglossum,s:Lasioglossum semicaeruleum
AATACTTTACTTTATTTTTGCAATATGAGCAGGAATAATTGGAGCTTCCTTAAGAATAAT
TATTCGAATAGAATTAAGTGCACCTGGAAAATGAATTAATAATGATCAAATTTATAATAC
TATTATTACCTCTCATGCATTTGTAATAATTTTTTTTATAGTTATACCATTTATAATTGG
AGGATTTGGAAATTGATTAGTTCCCTTAATAATTGGTGCTCCTGATATAGCATTTCCTCG
AATAAATAATATAAGATTCTGATTACTTATCCCATCAATATTTATATTATTAATAAGAAG
ATTTATTTCATCAGGTTCAGGGACTGGGTGAACTGTTTATCCTCCCTTATCTTCAATCAT
ATATCATTCATCAATTTCTGTAGATTACACTATTTTTTCTTTACATATTGCGGGAATTTC
ATCCATTATAGGAGCTATTAATTTTATTGTATCTATTATACTTATAAAAAATATTTCAAT
TAATTATGATCAAATTCCTTTATTTCCATGATCAGTAAAAATTACTGCTATTCTATTATT
ATTATCTCTTCCAGTATTAGCAGGAGCTATTACTATACTTTTAACAGACCGAAACTTAAA
TACTTC
>KR888624;tax=d:Eukarya,p:Arthropoda,c:Insecta,o:Hymenoptera,f:Halictidae,g:Lasioglossum,s:Lasioglossum semicaeruleum
AATACTTTACTTTATTTTTGCAATATGAGCAGGAATAATTGGAGCTTCCTTAAGAATAAT
TATTCGAATAGAATTAAGTGCACCTGGAAAATGAATTAATAATGATCAAATTTATAATAC
TATTATTACCTCTCATGCATTTGTAATAATTTTTTTTATAGTTATACCATTTATAATTGG
AGGATTTGGAAATTGATTAGTTCCCTTAATAATTGGTGCTCCTGATATAGCATTTCCTCG
AATAAATAATATAAGATTCTGATTACTTATCCCATCAATATTTATATTATTAATAAGAAG
ATTTATTTCATCAGGTTCAGGGACTGGGTGAACTATTTATCCTCCCTTATCTTCAATCAT
ATATCATTCATCAATTTCTGTAGATTACACTATTTTTTCTTTACATATTGCGGGAATTTC
ATCCATTATAGGAGCTATTAATTTTATTGTATCTATTATACTTATAAAAAATATTTCAAT
TAATTATGATCAAATTCCTTTATTTCCATGATCAGTAAAAATTACTGCTATTCTATTATT
ATTATCTCTTCCAGTATTAGCAGGAGCTATTACTATA
>KR891614;tax=d:Eukarya,p:Arthropoda,c:Insecta,o:Hymenoptera,f:Halictidae,g:Lasioglossum,s:Lasioglossum semicaeruleum
ATACTTTACTTTATTTTTGCAATATGAGCAGGAATAATTGGAGCTTCCTTAAGAATAATT
ATTCGAATAGAATTAAGTGCACCTGGAAAATGAATTAATAATGATCAAATTTATAATACT
ATTATTACCTCTCATGCATTTGTAATAATTTTTTTTATAGTTATACCATTTATAATTGGA
GGATTTGGAAATTGATTAGTTCCCTTAATAATTGGTGCTCCTGATATAGCATTTCCTCGA
ATAAATAATATAAGATTCTGATTACTTATCCCATCAATATTTATATTATTAATAAGAAGA
TTTATTTCATCAGGTTCAGGGACTGGGTGAACTGTTTATCCTCCCTTATCTTCAATCATA
TATCATTCATCAATTTCTGTAGATTACACTATTTTTTCTTTACATATTGCGGGAATTTCA
TCCATTATAGGAGCTATTAATTTTATTGTATCTATTATACTTATAAAAAATATTTCAATT
AATTATGATCAAATTCCTTTATTTCCATGATCAGTAAAAATTACTGCTATTCTATTATTA
TTATCTCTTCCAGTATTAGCAGGAGCTATTACTATACTTTTA

>KR892944;tax=d:Eukarya,p:Arthropoda,c:Insecta,o:Hymenoptera,f:Halictidae,g:Lasioglossum,s:Lasioglossum semicaeruleum
AATACTTTACTTTATTTTTGCAATATGAGCAGGAATAATTGGAGCTTCCTTAAGAATAAT
TATTCGAATAGAATTAAGTGCACCTGGAAAATGAATTAATAATGATCAAATTTATAATAC
TATTATTACCTCTCATGCATTTGTAATAATTTTTTTTATAGTTATACCATTTATAATTGG
AGGATTTGGAAATTGATTAGTTCCCTTAATAATTGGTGCTCCTGATATAGCATTTCCTCG
AATAAATAATATAAGATTCTGATTACTTATCCCATCAATATTTATATTATTAATAAGAAG
ATTTATTTCATCAGGTTCAGGGACTGGGTGAACTGTTTATCCTCCCTTATCTTCAATCAT
ATATCATTCATCAATTTCTGTAGATTACACTATTTTTTCTTTACATATTGCGGGAATTTC
ATCCATTATAGGAGCTATTAATTTTATTGTATCTATTATACTTATAAAAAATATTTCAAT
TAATTATGATCAAATTCCTTTATTTCCATGATCAGTAAAAATTACTGCTATTCTATTATT
ATTATCTCTTCCAGTATTAGCAGGAG
>JF903559;tax=d:Eukarya,p:Arthropoda,c:Insecta,o:Hymenoptera,f:Halictidae,g:Lasioglossum,s:Lasioglossum simplex
TATACTTTATTTTATTTTCGCTATATGAGCTGGAATAATTGGAGCTTCATTAAGAATAAT
TATTCGAATAGAATTAAGTGCTCCTGGAAAATGAATTAATAATGATCAAATTTATAATAC
TATTATTACATCTCATGCATTTGTAATAATTTTTTTTATAGTTATACCATTTATAATTGG
TGGTTTTGGTAACTGATTAGTTCCTTTAATAATTGGGGCACCTGATATAGCATTCCCCCG
AATAAATAATATAAGATTTTGATTACTTATCCCATCAATATTTATATTATTAATAAGAAG
CATTATATCATCTGGTTCAGGAACTGGATGAACTGTATACCCTCCTTTATCATCCATTAT
ATACCATTCATCAATTTCAGTAGATTACACTATCTTTTCATTACATATTGCAGGAATTTC
ATCTATTATAGGAGCTATTAACTTTATTGTATCTATTATACTTATAAAAAATATTTCAAT
TAATTATGATCAAATCCCTTTATTCCCATGATCAGTAAAAATTACTGCTATTTTACTATT
ATTATCTCTACCAGTTTTAGCAGGAGCTATCACTATACTTCTAACAGATCGAAATTTAAA
TACTTCATTTTTTGACCCCTCAGGAGGAGGAGACCCTATTCTTTATCAACATTTATTT
>MF902092;tax=d:Eukarya,p:Arthropoda,c:Insecta,o:Hymenoptera,f:Halictidae,g:Lasioglossum,s:Lasioglossum tenax
ATACTTTATTTTATCTTTGCTATATGAGCTGGAATAATTGGAGCTTCATTAAGAATAATT
ATTCGAATAGAATTAAGTGCACCAGGAAAATGAATTAATAATGATCAAATTTATAATACT
ATTATTACTTCTCATGCATTTGTAATAATCTTTTTTATAGTTATACCATTTATAATTGGA
GGATTTGGTAATTGATTAATCCCCTTAATAATTGGTGCCCCAGATATAGCATTTCCTCGA
ATAAATAACATAAGATTTTGATTACTTATCCCATCAATATTTATATTATTAATAAGAAGT
ATTATATCATCTGGATCAGGAACTGGATGAACTGTATACCCCCCCTTATCTTCAATTATA
TACCATTCATCTATTTCAGTAGATTACACTATTTTTTCATTACATATTGCAGGAATTTCA
TCTATTATAGGAGCCATCAACTTTATTGTATCTATTATACTTATAAAAAATATTTCAATT
AATTATGATCAAATTCCTTTATTTCCATGATCAGTAAAAATTACTGCTATTTTATTATTA
TTATCTTTACCAGTTTTAGCAGGAGCTATTACTATACTCTTAACAGAT
>MF906712;tax=d:Eukarya,p:Arthropoda,c:Insecta,o:Hymenoptera,f:Halictidae,g:Lasioglossum,s:Lasioglossum tenax
ATACTTTATTTTATCTTTGCTATATGAGCTGGAATAATTGGAGCTTCATTAAGAATAATT
ATTCGAATAGAATTAAGTGCACCAGGAAAATGAATTAATAATGATCAAATTTATAATACT
ATTATTACTTCTCATGCATTTGTAATAATCTTTTTTATAGTTATACCATTTATAATTGGA
GGATTTGGTAATTGATTAATCCCCTTAATAATTGGTGCTCCAGATATAGCATTTCCTCGA
ATAAATAATATAAGATTTTGATTACTTATCCCATCAATATTTATATTATTAATAAGAAGT
ATTATATCATCTGGATCAGGAACTGGATGAACTGTATACCCCCCCTTATCTTCAATTATA
TACCATTCATCTATTTCAGTAGATTACACTATTTTTTCATTACATATTGCAGGAATTTCA
TCTATTATAGGAGCCATCAACTTTATTGTATCTATTATACTTATAAAAAATATTTCAATT
AATTATGATCAAATTCCTTTATTTCCATGATCAGTAAAAATTACTGCTATTTTATTATTA
TTATCTTTACCAGTTTTAGCAGGAGCTATTACTATACTCTTAACAGAT
>MG336176;tax=d:Eukarya,p:Arthropoda,c:Insecta,o:Hymenoptera,f:Halictidae,g:Lasioglossum,s:Lasioglossum tenax
ATACTTTATTTTATCTTTGCTATATGAGCTGGAATAATTGGAGCTTCATTAAGAATAATT
ATTCGAATAGAATTAAGTGCACCAGGAAAATGAATTAATAATGATCAAATTTATAATACT

ATTATTACTTCTCATGCATTTGTAATAATCTTTTTTATAGTTATACCATTTATAATTGGA
GGATTTGGTAATTGATTAATCCCCTTAATAATTGGTGCTCCAGATATAGCATTTCCTCGA
ATAAATAACATAAGATTTTGATTACTTATCCCATCAATATTTATATTATTAATAAGAAGT
ATTATATCATCTGGATCAGGAACTGGATGAACTGTATACCCCCCCTTATCTTCAATTATA
TACCATTCATCTATTTCAGTAGATTACACTATTTTTCATTACATATTGCAGGAATTTCA
TCTATTATAGGAGCCATCAACTTTATTGTATCTATTATACTTATAAAAAATATTTCAATT
AATTATGATCAAATTCCTTTATTTCCATGATCAGTAAAAATTACTGCTATTTTATTATTA
TTATCTTTACCAGTTTTAGCA
>GU708183;tax=d:Eukarya,p:Arthropoda,c:Insecta,o:Hymenoptera,f:Halictidae,g:Lasioglossum,s:Lasioglossum versatum
TATACTTTATTTTATTTTTGCTATATGAGCTGGAATAATTGGAGCTTCATTAAGAATAAT
TATTCGAATAGAATTAAGTGCTCCTGGAAAATGAATTAATAATGATCAAATTTATAATAC
TATTATTACTTCACATGCATTTGTAATAATTTTTTTATAGTTATACCATTTATAATCGG
AGGATTTGGTAATTGATTAGTTCCTTTAATAATTGGAGCACCTGATATAGCATTCCCCCG
AATAAATAACATAAGATTTTGATTACTTATTCCATCAATATTTATATTATTAATAAGAAG
TATTATATCATCTGGTTCAGGTACTGGATGAACTGTATATCCACCTTTATCATCTATTAT
ATACCATTCATCAATTTCAGTAGATTACACTATTTTTCATTACACATTGCAGGAATCTC
CTCTATTATAGGAGCTATCAATTTTATTGTATCTATTTTACTTATAAAAAATATTTCAAT
TAATTATGATCAAATTCCTTTATTTCCATGATCAGTAAAAATTACTGCTATTTTATTATT
ATTATCCTTACCAGTTTTAGCAGGAGCTATTACTATACTTTTAACAGATCGAAATTTAAA
TACTTCATTTTTTGATCCTTCAGGAGGAGGAGACCCTATTCTTTATCAACATCTATTT
>HQ558153;tax=d:Eukarya,p:Arthropoda,c:Insecta,o:Hymenoptera,f:Halictidae,g:Lasioglossum,s:Lasioglossum versatum
TATACTTTATTTTATTTTTGCTATATGAGCTGGAATAATTGGAGCTTCATTAAGAATAAT
TATTCGAATAGAATTAAGTGCTCCTGGAAAATGAATTAATAATGATCAAATTTATAATAC
TATTATTACTTCACATGCATTTGTAATAATTTTTTTATAGTTATACCATTTATAATCGG
AGGATTTGGTAATTGATTAGTTCCTTTAATAATTGGAGCACCTGATATAGCATTCCCCCG
AATAAATAATATAAGATTTTGATTACTTATTCCATCAATATTTATATTATTAATAAGAAG
TATTATATCATCTGGTTCAGGTACTGGATGAACTGTATATCCACCTTTATCATCTATTAT
ATACCATTCATCAATTTCAGTAGATTACACTATTTTTCATTACACATTGCAGGAATCTC
CTCTATTATAGGAGCTATCAATTTTATTGTATCTATTTTACTTATAAAAAATATTTCAAT
TAATTATGATCAAATTCCTTTATTTCCATGATCAGTAAAAATTACTGCTATTTTATTATT
ATTATCCTTACCAGTTTTAGCAGGAGCTATTACTATACTTTTAACAGATCGAAATTTAAA
TACTTCATTTTTTGATCCTTCAGGAGGAGGAGACCCTATTCTTTATCAACATCTATTT
>HQ558114;tax=d:Eukarya,p:Arthropoda,c:Insecta,o:Hymenoptera,f:Halictidae,g:Lasioglossum,s:Lasioglossum versatum
TATACTTTATTTTATTTTTGCTATATGAGCCGGAATAATTGGAGCTTCATTAAGAATAAT
TATTCGAATAGAATTAAGTGCTCCTGGAAAATGAATTAATAATGATCAAATTTATAATAC
TATTATTACTTCACATGCATTTGTAATAATTTTTTTATAGTTATACCATTTATAATCGG
AGGATTTGGTAATTGATTAGTTCCTTTAATAATTGGAGCACCTGATATAGCATTCCCCCG
AATAAATAACATAAGATTTTGATTACTTATTCCATCAATATTTATATTATTAATAAGAAG
TATTATATCATCTGGTTCAGGTACTGGATGAACTGTATATCCACCTTTATCATCTATTAT
ATACCATTCATCAATTTCAGTAGATTACACTATTTTTCATTACACATTGCAGGAATCTC
CTCTATTATAGGAGCTATCAATTTTATTGTATCTATTTTACTTATAAAAAATATTTCAAT
TAATTATGATCAAATTCCTTTATTTCCATGATCAGTAAAAATTACTGCTATTTTATTATT
ATTATCCTTACCAGTTTTAGCAGGAGCTATTACTATACTTTTAACAGATCGAAATTTAAA
TACTTCATTTTTTGATCCTTCAGGAGGAGGAGACCCTATTCTTTATCAACATCTATTT
>HQ937905;tax=d:Eukarya,p:Arthropoda,c:Insecta,o:Hymenoptera,f:Halictidae,g:Lasioglossum,s:Lasioglossum versatum
TATGAGCTGGAATAATTGGAGCTTCATTAAGAATAATTATTCGAATAGAATTAAGTGCTC
CTGGAAAATGAATTAATAATGATCAAATTTATAATACTATTATTACTTCACATGCATTTG

TAATAATTTTTTTTATAGTTATACCATTTATAATCGGAGGATTTGGTAATTGATTAGTTC
CTTTAATAATTGGAGCACCTGATATAGCATTCCCCCGAATAAATAATATAAGATTTTGAT
TACTTATTCCATCAATATTTATATTATTAATAAGAAGTATTATATCATCTGGTTCAGGTA
CTGGATGAACTGTATATCCACCTTTATCATCTATTATATACCATTCATCAATTTCAGTAG
ATTACACTATTTTTTCATTACACATTGCAGGAATCTCCTCTATTATAGGAGCTATCAATT
TTATTGTATCTATTTTACTTATAAAAAATATTTCAATTAATTATGATCAAATTCCTTTAT
TTCCATGATCAGTAAAAATTACTGCTATTTTATTATTATTATCCTTACCAGTTTTAGCAG
GAGCTATTACTATACTTTTAACAGATCGAAATTTAAATACTTCATTTTTTGATCCTTCAG
GAGGAGGAGACCCTATTCTTTATCAACATCTATTT
>KR802684;tax=d:Eukarya,p:Arthropoda,c:Insecta,o:Hymenoptera,f:Halictidae,g:Lasioglossum,s:Lasioglossum versatum
TTGGAGCTTCATTAAGAATAATTATTCGAATAGAATTAAGTGCTCCTGGAAAATGAATTA
ATAATGATCAAATTTATAATACTATTATTACTTCACATGCATTTGTAATAATTTTTTTTA
TAGTTATACCATTTATAATCGGAGGATTTGGTAATTGATTAGTTCCTTTAATAATTGGAG
CACCTGATATAGCATTCCCCCGAATAAATAATATAAGATTTTGATTACTTATTCCATCAA
TATTTATATTATTAATAAGAAGTATTATATCATCTGGTTCAGGTACTGGATGAACTGTAT
ATCCACCTTTATCATCTATTATATACCATTCATCAATTTCAGTAGATTACACTATTTTTT
CATTACACATTGCAGGAATCTCCTCTATTATAGGAGCTATCAATTTTATTGTATCTATTT
TACTTATAAAAAATATTTCAATTAATTATGATCAAATTCCTTTATTTCCATGATCAGTAA
AAATTACTGCTATTTTATTATTATTATCCTTACCAGTTTTAGCAGGAGCTATTACTATAC
TTTTAACAGATCGAAATTTAAATACTTCATTTTTTGATCCTTCAGGAGGAGGAGACCCTA
TTCTTTATCAACATCTATTT
>KR802836;tax=d:Eukarya,p:Arthropoda,c:Insecta,o:Hymenoptera,f:Halictidae,g:Lasioglossum,s:Lasioglossum versatum
GGATAATTGGAGCTTCATTAAGAATAATTATTCGAATAGAATTAAGTGCTCCTGGAAAAT
GAATTAATAATGATCAAATTTATAATACTATTATTACTTCACATGCATTTGTAATAATTT
TTTTTATAGTTATACCATTTATAATCGGAGGATTTGGTAATTGATTAGTTCCTTTAATAA
TTGGAGCACCTGATATAGCATTCCCCCGAATAAATAATATAAGATTTTGATTACTTATTC
CATCAATATTTATATTATTAATAAGAAGTATTATATCATCTGGTTCAGGTACTGGATGAA
CTGTATATCCACCTTTATCATCTATTATATACCATTCATCAATTTCAGTAGATTACACTA
TTTTTTCATTACACATTGCAGGAATCTCCTCTATTATAGGAGCTATCAATTTTATTGTAT
CTATTTTACTTATAAAAAATATTTCAATTAATTATGATCAAATTCCTTTATTTCCATGAT
CAGTAAAAATTACTGCTATTTTATTATTATTATCCTTACCAGTTTTAGCAGGAGCTATTA
CTATACTTTTAACAGATCGAAATTTAAATACTTCATTTTTTGATCCTTCAGGAGGAGGAG
ACCCTATTCTTTATCAACATCTATTT
>KR807349;tax=d:Eukarya,p:Arthropoda,c:Insecta,o:Hymenoptera,f:Halictidae,g:Lasioglossum,s:Lasioglossum versatum
GGAATAATTGGAGCTTCATTAAGAATAATTATTCGAATAGAATTAAGTGCTCCTGGAAAA
TGAATTAATAATGATCAAATTTATAATACTATTATTACTTCACATGCATTTGTAATAATT
TTTTTTATAGTTATACCATTTATAATCGGAGGATTTGGTAATTGATTAGTTCCTTTAATA
ATTGGAGCACCTGATATAGCATTCCCCCGAATAAATAATATAAGATTTTGATTACTTATT
CCATCAATATTTATATTATTAATAAGAAGTATTATATCATCTGGTTCAGGTACTGGATGA
ACTGTATATCCACCTTTATCATCTATTATATACCATTCATCAATTTCAGTAGATTACACT
ATTTTTTCATTACACATTGCAGGAATCTCCTCTATTATAGGAGCTATCAATTTTATTGTA
TCTATTTTACTTATAAAAAATATTTCAATTAATTATGATCAAATTCCTTTATTTCCATGA
TCAGTAAAAATTACTGCTATTTTATTATTATTATCCTTACCAGTTTTAGCAGGAGCTATT
ACTATACTTTTAACAGATCGAAATTTAAATACTTCATTTTTTGATCCTTCAGGAGGAGGA
GACCCTATTCTTTATCAACATCTATTT
>KR880044;tax=d:Eukarya,p:Arthropoda,c:Insecta,o:Hymenoptera,f:Halictidae,g:Lasioglossum,s:Lasioglossum versatum
ATACTTTATTTTATTTTTGCTATATGAGCTGGAATAATTGGAGCTTCATTAAGAATAATT

ATTCGAATAGAATTAAGTGCTCCTGGAAAATGAATTAATAATGATCAAATTTATAATACT
ATTATTACTTCACATGCATTTGTAATAATTTTTTTTATAGTTATACCATTTATAATCGGA
GGATTTGGTAATTGATTAGTTCCTTTAATAATTGGAGCACCTGATATAGCATTCCCCCGA
ATAAATAATATAAGATTTTGATTACTTATTCCATCAATATTTATATTATTAATAAGAAGT
ATTATATCATCTGGTTCAGGTACTGGATGAACTGTATATCCACCTTTATCATCTATTATA
TACCATTCATCAATTTCAGTAGATTACACTATTTTTTCATTACACATTGCAGGAATCTCC
TCTATTATAGGAGCTATCAATTTTATTGTATCTATTTTACTTATAAAAAATATTTCAATT
AATTATGATCAAATTCCTTTATTTCCATGATCAGTAAAAATTACTGCTATTTTATTATTA
TTATCCTTACCAGTTTTAGCAGGA
>KR891834;tax=d:Eukarya,p:Arthropoda,c:Insecta,o:Hymenoptera,f:Halictidae,g:Lasioglossum,s:Lasioglossum versatum
ATACTTTATTTTATTTTTGCTATATGAGCTGGAATAATTGGAGCTTCATTAAGAATAATT
ATTCGAATAGAATTAAGTGCTCCTGGAAAATGAATTAATAATGATCAAATTTATAATACT
ATTATTACTTCACATGCATTTGTAATAATTTTTTTTATAGTTATACCATTTATAATCGGA
GGATTTGGTAATTGATTAGTTCCTTTAATAATTGGAGCACCTGATATAGCATTCCCCCGA
ATAAATAATATAAGATTTTGATTACTTATTCCATCAATATTTATATTATTAATAAGAAGT
ATTATATCATCTGGTTCAGGTACTGGATGAACTGTATATCCACCTTTATCATCTATTATA
TACCATTCATCAATTTCAGTAGATTACACTATTTTTTCATTACACATTGCAGGAATCTCC
TCTATTATAGGAGCTATCAATTTTATTGTATCTATTTTACTTATAAAAAATATTTCAATT
AATTATGATCAAATTCCTTTATTTCCATGATCAGTAAAAATTACTGCTATT
>MF906136;tax=d:Eukarya,p:Arthropoda,c:Insecta,o:Hymenoptera,f:Halictidae,g:Lasioglossum,s:Lasioglossum versatum
ATACTTTATTTTATTTTTGCTATATGAGCTGGAATAATTGGAGCTTCATTAAGAATAATT
ATTCGAATAGAATTAAGTGCTCCTGGAAAATGAATTAATAATGATCAAATTTATAATACT
ATTATTACTTCACATGCATTTGTAATAATTTTTTTTATAGTTATACCATTTATAATCGGA
GGATTTGGTAATTGATTAGTTCCTTTAATAATTGGAGCACCTGATATAGCATTCCCCCGA
ATAAATAATATAAGATTTTGATTACTTATTCCATCAATATTTATATTATTAATAAGAAGT
ATTATATCATCTGGTTCAGGTACTGGATGAACTGTATATCCACCTTTATCATCTATTATA
TACCATTCATCAATTTCAGTAGATTACACTATTTTTTCATTACACATTGCAGGAATCTCC
TCTATTATAGGAGCTATCAATTTTATTGTATCTATTTTACTTATAAAAAATATTTCAATT
AATTATGATCAAATTCCTTTATTTCCATGATCAGTAAAAATTACTGCTATTTTATTATTA
TTATCCTTACCAGTTTTAGCAGGAGCTATTACTATACTTTTAACAGAT
>JN310547;tax=d:Eukarya,p:Arthropoda,c:Insecta,o:Hymenoptera,f:Halictidae,g:Lasioglossum,s:Lasioglossum weemsi
AATTGGAGCTTCATTAAGAATAATTATTCGAATAGAATTAAGTGCTCCTGGAAAATGAAT
TAATAATGATCAAATTTATAATACTATTATTACATCTCATGCATTTGTAATAATTTTTTT
TATAGTTATACCATTTATAATTGGTGGTTTTGGTAATTGATTAGTTCCTTTAATAATTGG
AGCCCCTGATATAGCATTCCCCCGAATAAATAACATAAGATTTTGATTACTTATCCCATC
AATATTTATATTATTAATAAGAAGCATTATATCATCTGGTTCAGGAACTGGATGAACTGT
ATACCCCCCTTTATCATCAATTATATACCATTCATCAATTTCAGTAGATTACACTATCTT
TTCATTACATATTGCAGGAATTTCATCTATTATAGGAGCTATTAACTTTATTGTATCTAT
TTTACTTATAAAAAATATTTCAATTAACTATGACCAAATTCCTTTATTCCCATGATCAGT
AAAAATTACTGCTATTTTATTATTATTATCTCTACCAGTTTTAGCAGGAGCTATTACTAT
ACTTCTAACAGATCGAAATTTAAATACTTCATTTTTTGACCCATCAGGAGGAGGAGATCC
TATTCTTTATCAACATTTATTT
>KR791473;tax=d:Eukarya,p:Arthropoda,c:Insecta,o:Hymenoptera,f:Halictidae,g:Lasioglossum,s:Lasioglossum weemsi
TACTTTATTTTATTTTCGCTATATGAGCTGGAATAATTGGAGCTTCATTAAGAATAATTA
TTCGAATAGAATTAAGTGCTCCTGGAAAATGAATTAATAATGATCAAATTTATAATACTA
TTATTACATCTCATGCATTTGTAATAATTTTTTTTATAGTTATACCATTTATAATTGGTG
GATTTGGTAATTGATTAGTTCCTTTAATAATTGGGGCCCCTGATATAGCATTTCCCCGAA

TAAATAACATAAGATTTTGATTACTTATCCCATCAATATTTATATTATTAATAAGAAGCA
TTATATCATCTGGTTCAGGAACTGGATGAACTGTATACCCCCCTTTATCATCAATTATAT
ACCATTCATCAATTTCAGTAGATTACACTATCTTTTCATTACATATTGCAGGAATTTCAT
CTATTATAGGAGCTATTAACTTTATTGTATCTATTTTACTTATAAAAAATATTTCAATTA
ATTATGACCAAATTCCTTTATTCCCATGATCAGTAAAAATTACTGCTATTTTATTATTAT
TATCTCTACCAGTTTTAGCAGGAGCTAT
>KR792888;tax=d:Eukarya,p:Arthropoda,c:Insecta,o:Hymenoptera,f:Halictidae,g:Lasioglossum,s:Lasioglossum weemsi
GATACTTTATTTTATTTTCGCTATATGAGCTGGAATAATTGGAGCTTCATTAAGAATAAT
TATTCGAATAGAATTAAGTGCTCCTGGAAAATGAATTAATAATGATCAAATTTATAATAC
TATTATTACATCTCATGCATTTGTAATAATTTTTTTTATAGTTATACCATTTATAATTGG
TGGATTTGGTAATTGATTAGTTCCTTTAATAATTGGGGCCCCTGATATAGCATTTCCCCG
AATAAATAACATAAGATTTTGATTACTTATCCCATCAATATTTATATTATTAATAAGAAG
CATTATATCATCTGGTTCAGGAACTGGATGAACTGTATACCCCCCTTTATCATCAATTAT
ATACCATTCATCAATTTCAGTAGATTACACTATCTTTTCATTACATATTGCAGGAATTTC
ATCTATTATAGGAGCTATTAACTTTATTGTATCTATTTTACTTATAAAAAATATTTCAAT
TAATTATGACCAAATTCCTTTATTCCCATGATCAGTAAAAATTACTGCTATTTTATTATT
ATTATCTCTACCAGTTTTAGCAGGAGCTATTACTATACTTCTAACAGATCGAAATTTAAA
TACTTCATTTT
>KR794471;tax=d:Eukarya,p:Arthropoda,c:Insecta,o:Hymenoptera,f:Halictidae,g:Lasioglossum,s:Lasioglossum weemsi
ATTGGAGCTTCATTAAGAATAATTATTCGAATAGAATTAAGTGCTCCTGGAAAATGAATT
AATAATGATCAAATTTATAATACTATTATTACATCTCATGCATTTGTAATAATTTTTTTT
ATAGTTATACCATTTATAATTGGTGGTTTTGGTAATTGATTAGTTCCTTTAATAATTGGA
GCCCCTGATATAGCATTCCCCCGAATAAATAACATAAGATTTTGATTACTTATCCCATCA
ATATTTATATTATTAATAAGAAGCATTATATCATCTGGTTCAGGAACTGGATGAACTGTA
TACCCCCCTTTATCATCAATTATATACCATTCATCAATTTCAGTAGATTACACTATCTTT
TCATTACATATTGCAGGAATTTCATCTATTATAGGAGCTATTAACTTTATTGTATCTATT
TTACTTATAAAAAATATTTCAATTAACTATGACCAAATTCCTTTATTCCCATGATCAGTA
AAAATTACTGCTATTTTATTATTATTATCTCTACCAGTTTTAGCAGGAGCTATTACTATA
CTTCTAACAGATCGAAATTTAAATACTTCATTTTTTGACCCATCAGGAGGAGGAGATCCT
ATTCTTTATCAACATTTATTT
>KR798800;tax=d:Eukarya,p:Arthropoda,c:Insecta,o:Hymenoptera,f:Halictidae,g:Lasioglossum,s:Lasioglossum weemsi
TTTTATTTTCGCTATATGAGCTGGAATAATTGGAGCTTCATTAAGAATAATTATTCGAAT
AGAATTAAGTGCTCCTGGAAAATGAATTAATAATGATCAAATTTATAATACTATTATTAC
ATCTCATGCATTTGTAATAATTTTTTTTATAGTTATACCATTTATAATTGGTGGTTTTGG
TAATTGATTAGTTCCTTTAATAATTGGAGCCCCTGATATAGCATTCCCCCGAATAAATAA
CATAAGATTTTGATTACTTATCCCATCAATATTTATATTATTAATAAGAAGCATTATATC
ATCTGGTTCAGGAACTGGATGAACTGTATACCCCCCTTTATCATCAATTATATACCATTC
ATCAATTTCAGTAGATTACACTATCTTTTCATTACATATTGCAGGAATTTCATCTATTAT
AGGAGCTATTAACTTTATTGTATCTATTTTACTTATAAAAAATATTTCAATTAACTATGA
CCAAATTCCTTTATTCCCATGATCAGTAAAAATTACTGCTATTTTATTATTATTATCTCT
ACCAGTTTTAGCAGGAGCTATTACTATACTTCTAACAGAT
>KR802686;tax=d:Eukarya,p:Arthropoda,c:Insecta,o:Hymenoptera,f:Halictidae,g:Lasioglossum,s:Lasioglossum weemsi
GATACTTTATTTTATTTTCGCTATATGAGCTGGAATAATTGGAGCTTCATTAAGAATAAT
TATTCGAATAGAATTAAGTGCTCCTGGAAAATGAATTAATAATGATCAAATTTATAATAC
TATTATTACATCTCATGCATTTGTAATAATTTTTTTTATAGTTATACCATTTATAATTGG
TGGTTTTGGTAATTGATTAGTTCCTTTAATAATTGGAGCCCCTGATATAGCATTCCCCCG
AATAAATAACATAAGATTTTGATTACTTATCCCATCAATATTTATATTATTAATAAGAAG

CATTATATCATCTGGTTCAGGAACTGGATGAACTGTATACCCCCCTTTATCATCAATTAT
ATACCATTCATCAATTTCAGTAGATTACACTATCTTTTCATTACATATTGCAGGAATTTC
ATCTATTATAGGAGCTATTAACTTTATTGTATCTATTTTACTTATAAAAAATATTTCAAT
TAACTATGACCAAATTCCTTTATTCCCATGATCAGTAAAAATTACTGCTATTTTATTATT
ATTATCTCTACCAGTTTTAGCAGGAGCTATTACTATACTTCTAACAGATCGAAATTTAAA
TACTTCATTTTTTGACCCATCAGGAGGAGGAG
>KR804567;tax=d:Eukarya,p:Arthropoda,c:Insecta,o:Hymenoptera,f:Halictidae,g:Lasioglossum,s:Lasioglossum weemsi
ATTTTATTTTCGCTATATGAGCTGGAATAATTGGAGCTTCATTAAGAATAATTATTCGAA
TAGAATTAAGTGCTCCTGGAAAATGAATTAATAATGATCAAATTTATAATACTATTATTA
CATCTCATGCATTTGTAATAATTTTTTTTATAGTTATACCATTTATAATTGGTGGTTTTG
GTAATTGATTAGTTCCTTTAATAATTGGAGCCCCTGATATAGCATTCCCCCGAATAAATA
ACATAAGATTTTGATTACTTATCCCATCAATATTTATATTATTAATAAGAAGCATTATAT
CATCTGGTTCAGGAACTGGATGAACTGTATACCCCCCTTTATCATCAATTATATATACCATT
CATCAATTTCAGTAGATTACACTATCTTTTCATTACATATTGCAGGAATTTCATCTATTA
TAGGAGCTATTAACTTTATTGTATCTATTTTACTTATAAAAAATATTTCAATTAACTATG
ACCAAATTCCTTTATTCCCATGATCAGTAAAAATTACTGCTATTTTATTATTATTATCTC
TACCAGTTTTAGCAGGAGCTATTACTATACTTCTAACAG
>KR805905;tax=d:Eukarya,p:Arthropoda,c:Insecta,o:Hymenoptera,f:Halictidae,g:Lasioglossum,s:Lasioglossum weemsi
GATACTTTATTTTATTTTCGCTATATGAGCTGGAATAATTGGAGCTTCATTAAGAATAAT
TATTCGAATAGAATTAAGTGCTCCTGGAAAATGAATTAATAATGATCAAATTTATAATAC
TATTATTACATCTCATGCATTTGTAATAATTTTTTTTATAGTTATACCATTTATAATTGG
TGGTTTTGGTAATTGATTAGTTCCTTTAATAATTGGAGCCCCTGATATAGCATTCCCCCG
AATAAATAACATAAGATTTTGATTACTTATCCCATCAATATTTATATTATTAATAAGAAG
CATTATATCATCTGGTTCAGGAACTGGATGAACTGTATACCCCCCTTTATCATCAATTAT
ATACCATTCATCAATTTCAGTAGATTACACTATCTTTTCATTACATATTGCAGGAATTTC
ATCTATTATAGGAGCTATTAACTTTATTGTATCTATTTTACTTATAAAAAATATTTCAAT
TAACTATGACCAAATTCCTTTATTCCCATGATCAGTAAAAATTACTGCTATTTTATTATT
ATTATCTCTACCAGTTTTAGCAGGAGCTATTACTATACTTCTAACAGATCGAAATTTAAA
TACTTCATTTTTTGACCCATCAGGAGGAGGAGACCCTATTCTTTATCAACATTTATTT
>KR805938;tax=d:Eukarya,p:Arthropoda,c:Insecta,o:Hymenoptera,f:Halictidae,g:Lasioglossum,s:Lasioglossum weemsi
GATACTTTATTTTATTTTCGCTATATGAGCTGGAATAATTGGAGCTTCATTAAGAATAAT
TATTCGAATAGAATTAAGTGCTCCTGGAAAATGAATTAATAATGATCAAATTTATAATAC
TATTATTACATCTCATGCATTTGTAATAATTTTTTTTATAGTTATACCATTTATAATTGG
TGGATTTGGTAATTGATTAGTTCCTTTAATAATTGGGGCCCCTGATATAGCATTTCCCCG
AATAAATAACATAAGATTTTGATTACTTATCCCATCAATATTTATATTATTAATAAGAAG
CATTATATCATCTGGTTCAGGAACTGGATGAACTGTATACCCCCCTTTATCATCAATTAT
ATACCATTCATCAATTTCAGTAGATTACACTATCTTTTCATTACATATTGCAGGAATTTC
ATCTATTATAGGAGCTATTAACTTTATTGTATCTATTTTACTTATAAAAAATATTTCAAT
TAATTATGACCAAATTCCTTTATTCCCATGATCAGTAAAAATTACTGCTATTTTATTATT
ATTATCTCTACCAGTTTTAGCAGGAGCTATTACTATACTTCTAACAGAT
>KR874448;tax=d:Eukarya,p:Arthropoda,c:Insecta,o:Hymenoptera,f:Halictidae,g:Lasioglossum,s:Lasioglossum weemsi
GATACTTTATTTTATTTTCGCTATATGAGCTGGAATAATTGGAGCTTCATTAAGAATAAT
TATTCGAATAGAATTAAGTGCTCCTGGAAAATGAATTAATAATGATCAAATTTATAATAC
TATTATTACATCTCATGCATTTGTAATAATTTTTTTTATAGTTATACCATTTATAATTGG
TGGATTTGGTAATTGATTAGTTCCTTTAATAATTGGGGCCCCTGATATAGCATTTCCCCG
AATAAATAACATAAGATTTTGATTACTTATCCCATCAATATTTATATTATTAATAAGAAG
CATTATATCATCTGGTTCAGGAACTGGATGAACTGTATACCCCCCTTTATCATCAATTAT

ATACCATTCATCAATTTCAGTAGATTACACTATCTTTTCATTACATATTGCAGGAATTTC
ATCTATTATAGGAGCTATTAACTTTATTGTATCTATTTTACTTATAAAAAATATTTCAAT
TAATTATGACCAAATTCCTTTATTCCCATGATCAGTAAAAATTACTGCTATTTTATTATT
ATTATCTCTACCAGTTTTAGCAGGAGCTATTACTATACTTCTAACAGATCGAAATTTAAA
TACTTCATTTTTTGACCCATCAGGAGGAGGAGATCCTATTCTTTATCAACATTTATTT
>KR875828;tax=d:Eukarya,p:Arthropoda,c:Insecta,o:Hymenoptera,f:Halictidae,g:Lasioglossum,s:Lasioglossum weemsi
GATACTTTATTTTATTTTCGCTATATGAGCTGGAATAATTGGAGCTTCATTAAGAATAAT
TATTCGAATAGAATTAAGTGCTCCTGGAAAATGAATTAATAATGATCAAATTTATAATAC
TATTATTACATCTCATGCATTTGTAATAATTTTTTTTATAGTTATACCATTTATAATTGG
TGGATTTGGTAATTGATTAGTTCCTTTAATAATTGGGGCCCCTGATATAGCATTTCCCCG
AATAAATAACATAAGATTTTGATTACTTATCCCATCAATATTTATATTATTAATAAGAAG
CATTATATCATCTGGTTCAGGAACTGGATGAACTGTATACCCCCCTTTATCATCAATTAT
ATACCATTCATCAATTTCAGTAGATTACACTATCTTTTCATTACATATTGCAGGAATTTC
ATCTATTATAGGAGCTATTAACTTTATTGTATCTATTTTACTTATAAAAAATATTTCAAT
TAATTATGACCAAATTCCTTTATTCCCATGATCAGTAAAAATTACTGCTATT
>KR878180;tax=d:Eukarya,p:Arthropoda,c:Insecta,o:Hymenoptera,f:Halictidae,g:Lasioglossum,s:Lasioglossum weemsi
TTTATTTTCGCTATATGAGCTGGAATAATTGGAGCTTCATTAAGAATAATTATTCGAATA
GAATTAAGTGCTCCTGGAAAATGAATTAATAATGATCAAATTTATAATACTATTATTACA
TCTCATGCATTTGTAATAATTTTTTTTATAGTTATACCATTTATAATTGGTGGTTTTGGT
AATTGATTAGTTCCTTTAATAATTGGAGCCCCTGATATAGCATTCCCCCGAATAAATAAC
ATAAGATTTTGATTACTTATCCCATCAATATTTATATTATTAATAAGAAGCATTATATCA
TCTGGTTCAGGAACTGGATGAACTGTATACCCCCCTTTATCATCAATTATATACCATTCA
TCAATTTCAGTAGATTACACTATCTTTTCATTACATATTGCAGGAATTTCATCTATTATA
GGAGCTATTAACTTTATTGTATCTATTTTACTTATAAAAAATATTTCAATTAACTATGAC
CAAATTCCTTTATTCCCATGATCAGTAAAAATTACTGCTATTTTATTATTATTATCTCTA
CCAGTTTTAGCAGGA
>KR878197;tax=d:Eukarya,p:Arthropoda,c:Insecta,o:Hymenoptera,f:Halictidae,g:Lasioglossum,s:Lasioglossum weemsi
ATACTTTATTTTATTTTCGCTATATGAGCTGGAATAATTGGAGCTTCATTAAGAATAATT
ATTCGAATAGAATTAAGTGCTCCTGGAAAATGAATTAATAATGATCAAATTTATAATACT
ATTATTACATCTCATGCATTTGTAATAATTTTTTTTATAGTTATACCATTTATAATTGGT
GGTTTTGGTAATTGATTAGTTCCTTTAATAATTGGAGCCCCTGATATAGCATTCCCCCGA
ATAAATAACATAAGATTTTGATTACTTATCCCATCAATATTTATATTATTAATAAGAAGC
ATTATATCATCTGGTTCAGGAACTGGATGAACTGTATACCCCCCTTTATCATCAATTATA
TACCATTCATCAATTTCAGTAGATTACACTATCTTTTCATTACATATTGCAGGAATTTCA
TCTATTATAGGAGCTATTAACTTTATTGTATCTATTTTACTTATAAAAAATATTTCAATT
AACTATGACCAAATTCCTTTATTCCCATGATCAGTAAAAATTACTGCTATTTTATTATTA
TTATCTCTACCAGTT
>KR879210;tax=d:Eukarya,p:Arthropoda,c:Insecta,o:Hymenoptera,f:Halictidae,g:Lasioglossum,s:Lasioglossum weemsi
ATACTTTATTTTATTTTCGCTATATGAGCTGGAATAATTGGAGCTTCATTAAGAATAATT
ATTCGAATAGAATTAAGTGCTCCTGGAAAATGAATTAATAATGATCAAATTTATAATACT
ATTATTACATCTCATGCATTTGTAATAATTTTTTTTATAGTTATACCATTTATAATTGGT
GGATTTGGTAATTGATTAGTTCCTTTAATAATTGGGGCCCCTGATATAGCATTTCCCCGA
ATAAATAACATAAGATTTTGATTACTTATCCCATCAATATTTATATTATTAATAAGAAGC
ATTATATCATCTGGTTCAGGAACTGGATGAACTGTATACCCCCCTTTATCATCAATTATA
TACCATTCATCAATTTCAGTAGATTACACTATCTTTTCATTACATATTGCAGGAATTTCA
TCTATTATAGGAGCTATTAACTTTATTGTATCTATTTTACTTATAAAAAATATTTCAATT
AATTATGACCAAATTCCTTTATTCCCATGATCAGTAAAAATTACTGCTATTTTATTATTA

TTATCTCTACCAGTTTTAGCAGGAGCTATTACTATACTTCTA
>KR882224;tax=d:Eukarya,p:Arthropoda,c:Insecta,o:Hymenoptera,f:Halictidae,g:Lasioglossum,s:Lasioglossum weemsi
GATACTTTATTTTATTTTCGCTATATGAGCTGGAATAATTGGAGCTTCATTAAGAATAAT
TATTCGAATAGAATTAAGTGCTCCTGGAAAATGAATTAATAATGATCAAATTTATAATAC
TATTATTACATCTCATGCATTTGTAATAATTTTTTTTATAGTTATACCATTTATAATTGG
TGGATTTGGTAATTGATTAGTTCCTTTAATAATTGGGGCCCCTGATATAGCATTCCCCCG
AATAAATAACATAAGATTTTGATTACTTATCCCATCAATATTTATATTATTAATAAGAAG
CATTATATCATCTGGTTCAGGAACTGGATGAACTGTATACCCCCCTTTATCATCAATTAT
ATACCATTCATCAATTTCAGTAGATTACACTATCTTTTCATTACATATTGCAGGAATTTC
ATCTATTATAGGAGCTATTAACTTTATTGTATCTATTTTACTTATAAAAAATATTTCAAT
TAATTATGACCAAATTCCTTTATTCCCATGATCAGTAAAAATTACTGCTATTTTATTATT
ATTATCTCTACCAGTTTTAGCAGGAGCTATTACTATACTTCTAACAGATCGAAATTTAAA
TACTTCATTTTTTGACCCATCAGGAGGAGGAGATCCTATTCTTTATCAACATTTATTT
>KR882477;tax=d:Eukarya,p:Arthropoda,c:Insecta,o:Hymenoptera,f:Halictidae,g:Lasioglossum,s:Lasioglossum weemsi
GATACTTTATTTTATTTTCGCTATATGAGCTGGAATAATTGGAGCTTCATTAAGAATAAT
TATTCGAATAGAATTAAGTGCTCCTGGAAAATGAATTAATAATGATCAAATTTATAATAC
TATTATTACATCTCATGCATTTGTAATAATTTTTTTTATAGTTATACCATTTATAATTGG
TGGATTTGGTAATTGATTAGTTCCTTTAATAATTGGGGCCCCTGATATAGCATTTCCCCG
AATAAATAACATAAGATTTTGATTACTTATCCCATCAATATTTATATTATTAATAAGAAG
CATTATATCATCTGGTTCAGGAACTGGATGAACTGTATACCCCCCTTTATCATCAATTAT
ATACCATTCATCAATTTCAGTAGATTACACTATCTTTTCATTACATATTGCAGGAATTTC
ATCTATTATAGGAGCTATTAACTTTATTGTATCTATTTTACTTATAAAAAATATTTCAAT
TAATTATGACCAAATTCCTTTATTCCCATGATCAGTAAAAATTACTGCTATTTTATTATT
ATTATCTCTACCAGTT
>KR883193;tax=d:Eukarya,p:Arthropoda,c:Insecta,o:Hymenoptera,f:Halictidae,g:Lasioglossum,s:Lasioglossum weemsi
TTTATTTTTGCTATATGAGCTGGAATAATTGGAGCTTCATTAAGAATAATTATTCGAATA
GAATTAAGTGCTCCTGGAAAATGAATTAATAATGATCAAATTTATAATACTATTATTACA
TCTCATGCATTTGTAATAATTTTTTTTATAGTTATACCATTTATAATTGGTGGATTTGGT
AATTGATTAGTTCCTTTAATAATTGGAGCCCCTGATATAGCATTCCCCCGAATAAATAAT
ATAAGATTTTGATTACTTATCCCATCAATATTTATATTATTAATAAGAAGAATTATATCA
TCTGGTTCAGGAACTGGATGAACTGTATACCCCCCCTTATCATCAATTATATACCATTCA
TCAATTTCAGTAGATTACACTATCTTTTCATTACATATTGCAGGAATTTCATCTATTATA
GGAGCTATTAACTTTATTGTATCTATTTTACTTATAAAAAATATTTCAATTAATTATGAT
CAAATTCCTTTATTCCCATGATCAGTAAAAATTACTGCTATTTTATTATTATTATCTCTA
CCAGTTTTAGCAGGAGCTATTACTATACTTTTAACAGAT
>KR884203;tax=d:Eukarya,p:Arthropoda,c:Insecta,o:Hymenoptera,f:Halictidae,g:Lasioglossum,s:Lasioglossum weemsi
ATACTTTATTTTATTTTTGCTATATGAGCTGGAATAATTGGAGCTTCATTAAGAATAATT
ATTCGAATAGAATTAAGTGCTCCTGGAAAATGAATTAATAATGATCAAATTTATAATACT
ATTATTACATCTCATGCATTTGTAATAATTTTTTTTATAGTTATACCATTTATAATTGGT
GGATTTGGTAATTGATTAGTTCCTTTAATAATTGGAGCCCCTGATATAGCATTCCCCCGA
ATAAATAATATAAGATTTTGATTACTTATCCCATCAATATTTATATTATTAATAAGAAGA
ATTATATCATCTGGTTCAGGAACTGGATGAACTGTATACCCCCCCTTATCATCGATTATA
TACCATTCATCAATTTCAGTAGATTACACTATCTTTTCATTACATATTGCAGGAATTTCA
TCTATTATAGGAGCTATTAACTTTATTGTATCTATTTTACTTATAAAAAATATTTCAATT
AATTATGATCAAATTCCTTTATTCCCATGATCAGTAAAAATTACTGCTATTTTATTATTA
TTATCTCTACCAGTTTTAGCAGGAGCTATTACTATACTTTTAACAGATCGA

>KR885331;tax=d:Eukarya,p:Arthropoda,c:Insecta,o:Hymenoptera,f:Halictidae,g:Lasioglossum,s:Lasioglossum weemsi
ATACTTTATTTTATTTTTGCTATATGAGCTGGAATAATTGGAGCTTCATTAAGAATAATT
ATTCGAATAGAATTAAGTGCTCCTGGAAAATGAATTAATAATGATCAAATTTATAATACT
ATTATTACATCTCATGCATTTGTAATAATTTTTTTTATAGTTATACCATTTATAATTGGT
GGATTTGGTAATTGATTAGTTCCTTTAATAATTGGAGCCCCTGATATAGCATTCCCCCGA
ATAAATAATATAAGATTTTGATTACTTATCCCATCAATATTTATATTATTAATAAGAAGA
ATTATATCATCTGGTTCAGGAACTGGATGAACTGTATACCCCCCCTTATCATCAATTATA
TACCATTCATCAATTTCAGTAGATTACACTATCTTTTCATTACATATTGCAGGAATTTCA
TCTATTATAGGAGCTATTAACTTTATTGTATCTATTTTACTTATAAAAAATATTTCAATT
AATTATGATCAAATTCCTTTATTCCCATGATCAGTAAAAATTACTGCTATTTTATTATTA
TTATCTCTACCAGTTTTAGCAGGAGCTATTACTATACTTTTAACAGAT
>KR885479;tax=d:Eukarya,p:Arthropoda,c:Insecta,o:Hymenoptera,f:Halictidae,g:Lasioglossum,s:Lasioglossum weemsi
GATACTTTATTTTATTTTCGCTATATGAGCTGGAATAATTGGAGCTTCATTAAGAATAAT
TATTCGAATAGAATTAAGTGCTCCTGGAAAATGAATTAATAATGATCAAATTTATAATAC
TATTATTACATCTCATGCATTTGTAATAATTTTTTTTATAGTTATACCATTTATAATTGG
TGGTTTTGGTAATTGATTAGTTCCTTTAATAATTGGAGCCCCTGATATAGCATTCCCCCG
AATAAATAACATAAGATTTTGATTACTTATCCCATCAATATTTATATTATTAATAAGAAG
CATTATATCATCTGGTTCAGGAACTGGATGAACTGTATACCCCCCTTTATCATCAATTAT
ATACCATTCATCAATTTCAGTAGATTACACTATCTTTTCATTACATATTGCAGGAATTTC
ATCTATTATAGGAGCTATTAACTTTATTGTATCTATTTTACTTATAAAAAATATTTCAAT
TAACTATGACCAAATTCCTTTATTCCCATGATCAGTAAAAATTACTGCTATTTTATTATT
ATTATCTCTACCAGTTTTAGCA
>KR887067;tax=d:Eukarya,p:Arthropoda,c:Insecta,o:Hymenoptera,f:Halictidae,g:Lasioglossum,s:Lasioglossum weemsi
GATACTTTATTTTATTTTCGCTATATGAGCTGGAATAATTGGAGCTTCATTAAGAATAAT
TATTCGAATAGAATTAAGTGCTCCTGGAAAATGAATTAATAATGATCAAATTTATAATAC
TATTATTACATCTCATGCATTTGTAATAATTTTTTTTATAGTTATACCATTTATAATTGG
TGGATTTGGTAATTGATTAGTTCCTTTAATAATTGGGGCCCCTGATATAGCATTTCCCCG
AATAAATAACATAAGATTTTGATTACTTATCCCATCAATATTTATATTATTAATAAGAAG
CATTATATCATCTGGTTCAGGAACTGGATGAACTGTATACCCCCCTTTATCATCAATTAT
ATACCATTCATCAATTTCAGTAGATTACACTATCTTTTCATTACATATTGCAGGAATTTC
ATCTATTATAGGAGCTATTAACTTTATTGTATCTATTTTACTTATAAAAAATATTTCAAT
TAATTATGACCAAATTCCTTTATTCCCATGATCAGTAAAAATTACTGCTATTTTATTATT
ATTATCTCTACCAGTTTTAGCAGGAGCTATTACTATA
>KR893006;tax=d:Eukarya,p:Arthropoda,c:Insecta,o:Hymenoptera,f:Halictidae,g:Lasioglossum,s:Lasioglossum weemsi
ATAATTGGAGCTTCATTAAGAATAATTATTCGAATAGAATTAAGTGCTCCTGGAAAATGA
ATTAATAATGATCAAATTTATAATACTATTATTACATCTCATGCATTTGTAATAATTTTT
TTTATAGTTATACCATTTATAATTGGTGGATTTGGTAATTGATTAGTTCCTTTAATAATT
GGGGCCCCTGATATAGCATTTCCCCGAATAAATAACATAAGATTTTGATTACTTATCCCA
TCAATATTTATATTATTAATAAGAAGCATTATATCATCTGGTTCAGGAACTGGATGAACT
GTATACCCCCCTTTATCATCAATTATATACCATTCATCAATTTCAGTAGATTACACTATC
TTTTCATTACATATTGCAGGAATTTCATCTATTATAGGAGCTATTAACTTTATTGTATCT
ATTTTACTTATAAAAAATATTTCAATTAATTATGACCAAATTCCTTTATTCCCATGATCA
GTAAAAATTACTGCTATTTTATTATTATTATCTCTACCAGTTTTAGCAGGAGCTATTACT
ATACTTCTAACAGATCGAAATTTAAATACTTCATTTTTTGACCCATCAGGAGGAGGAGAT
CCTATTCTTTATCAACATTTA
>KR894484;tax=d:Eukarya,p:Arthropoda,c:Insecta,o:Hymenoptera,f:Halictidae,g:Lasioglossum,s:Lasioglossum weemsi

TTTATTTTCGCTATATGAGCTGGAATAATTGGAGCTTCATTAAGAATAATTATTCGAATA
GAATTAAGTGCTCCTGGAAAATGAATTAATAATGATCAAATTTATAATACTATTATTACA
TCTCATGCATTTGTAATAATTTTTTTTATAGTTATACCATTTATAATTGGTGGATTTGGT
AATTGATTAGTTCCTTTAATAATTGGGGCCCCTGATATAGCATTTCCCCGAATAAATAAC
ATAAGATTTTGATTACTTATCCCATCAATATTTATATTATTAATAAGAAGCATTATATCA
TCTGGTTCAGGAACTGGATGAACTGTATACCCCCCTTTATCATCAATTATATACCATTCA
TCAATTTCAGTAGATTACACTATCTTTTCATTACATATTGCAGGAATTTCATCTATTATA
GGAGCTATTAACTTTATTGTATCTATTTTACTTATAAAAAATATTTCAATTAATTATGAC
CAAATTCCTTTATTCCCATGATCAGTAAAAATTACTGCTATTTTATTATTATTATCTCTA
CCAGTTTTAGCAGGAGCTATTACTATACTTCTAACA
>MG337687;tax=d:Eukarya,p:Arthropoda,c:Insecta,o:Hymenoptera,f:Halictidae,g:Lasioglossum,s:Lasioglossum weemsi
TTTATTTTCGCTATATGAGCTGGAATAATTGGAGCTTCATTAAGAATAATTATTCGAATA
GAATTAAGTGCTCCTGGAAAATGAATTAATAATGATCAAATTTATAATACTATTATTACA
TCTCATGCATTTGTAATAATTTTTTTTATAGTTATACCATTTATAATTGGTGGATTTGGT
AATTGATTAGTTCCTTTAATAATTGGGGCCCCTGATATAGCATTTCCCCGAATAAATAAC
ATAAGATTTTGATTACTTATCCCATCAATATTTATATTATTAATAAGAAGCATTATATCA
TCTGGTTCAGGAACTGGATGAACTGTATACCCCCCTTTATCATCAATTATATACCATTCA
TCAATTTCAGTAGATTACACTATCTTTTCATTACATATTGCAGGAATTTCATCTATTATA
GGAGCTATTAACTTTATTGTATCTATTTTACTTATAAAAAATATTTCAATTAATTATGAC
CAAATTCCTTTATTCCCATGATCAGTA
>JF903512;tax=d:Eukarya,p:Arthropoda,c:Insecta,o:Hymenoptera,f:Halictidae,g:Lasioglossum,s:Lasioglossum ephialtum
TATACTTTATTTTATTTTTGCTATATGAGCTGGGATAATTGGAGCTTCATTAAGAATAAT
TATTCGAATAGAATTAAGTGCTCCTGGAAAATGAATTAATAATGATCAAATTTATAACAC
TATTATTACCTCACATGCATTTGTAATAATTTTTTTTATAGTTATACCATTTATAATTGG
AGGATTTGGTAACTGATTAGTTCCTTTAATAATTGGAGCACCTGATATAGCATTCCCCCG
AATAAATAATATAAGATTTTGATTACTTATTCCATCAATATTTATATTATTAATAAGAAG
TATTATATCATCTGGTTCAGGAACTGGATGAACTGTATACCCTCCTTTATCATCTATTAT
ATATCATTCATCAATTTCAGTAGATTACACTATTTTTTCATTACACATTGCAGGAATTTC
ATCTATTATAGGAGCCATTAACTTCATTGTATCTATTTTACTTATAAAAAATATTTCAAT
TAATTATGATCAAATTCCATTATTTCCATGATCAGTAAAAATCACTGCTATTTTATTATT
ATTATCTCTTCCAGTTCTAGCAGGAGCTATTACTATACTTTTAACAGATCGAAATTTAAA
TACTTCATTTTTTGACCCTTCAGGAGGAGGGGATCCTATCCTTTATCAACATTTATTT
>JF903536;tax=d:Eukarya,p:Arthropoda,c:Insecta,o:Hymenoptera,f:Halictidae,g:Lasioglossum,s:Lasioglossum mitchelli
AATACTTTATTTTATTTTTGCTATATGAGCTGGAATAATTGGAGCTTCATTAAGAATAAT
TATTCGAATAGAATTAAGTGCTCCTGGAAAATGAATTAATAATGATCAAATTTATAATAC
TATTATTACATCTCATGCATTTGTAATAATTTTTTTTATAGTTATACCATTTATAATTGG
TGGATTTGGTAATTGATTAGTTCCTTTAATAATTGGAGCCCCTGATATAGCATTCCCCCG
AATAAATAATATAAGATTTTGATTACTTATCCCATCAATATTTATATTATTAATAAGAAG
AATTATATCATCTGGTTCAGGAACTGGATGAACTGTATACCCCCCCTTATCATCAATTAT
ATACCATTCATCAATTTCAGTAGATTACACTATCTTTTCATTACATATTGCAGGAATTTC
ATCTATTATAGGAGCTATTAACTTTATCGTATCTATTTTACTTATAAAAAATATTTCAAT
TAATTATGATCAAATTCCTTTATTCCCATGATCAGTAAAAATTACTGCTATTTTATTATT
ATTATCTCTACCAGTTTTAGCAGGAGCTATTACTATACTTTTAACAGATCGAAATTTAAA
TACTTCATTTTTTGACCCATCAGGAGGAGGGGACCCTATTCTTTATCAACATTTATTT
>GU708261;tax=d:Eukarya,p:Arthropoda,c:Insecta,o:Hymenoptera,f:Halictidae,g:Lasioglossum,s:Lasioglossum trigeminum
TATACTTTATTTTATTTTTGCTATATGAGCTGGAATAATTGGAGCTTCATTAAGAATAAT
TATTCGAATAGAATTAAGTGCCCCTGGAAAATGAATTAACAATGATCAAATTTATAATAC

CATTATTACTTCACATGCATTTGTAATAATTTTTTTTATAGTTATACCATTTATAATTGG
AGGATTTGGTAATTGACTAGTCCCTTTAATAATTGGAGCACCTGATATAGCATTCCCACG
AATAAATAATATAAGATTTTGATTACTTATCCCATCAATATTTATATTATTAATAAGAAG
TATTATATCATCTGGTTCAGGTACTGGATGAACCGTATACCCACCTTTATCATCTATTAT
ATACCATTCATCAATTTCAGTAGATTACACTATTTTTTCATTACATATTGCAGGAATCTC
ATCTATTATAGGAGCTATTAATTTTATTGTATCTATTTTACTTATAAAAAATATTTCAAT
TAATTATGACCAAATTCCTTTATTTCCATGATCAGTAAAAATTACTGCTATTTTACTATT
ATTATCTTTACCAGTTTTAGCAGGAGCTATTACTATACTTTTAACAGATCGAAACTTAAA
TACTTCATTTTTTGATCCTTCAGGTGGGGGAGACCCTATTCTTTATCAACATCTATTT
>GU708262;tax=d:Eukarya,p:Arthropoda,c:Insecta,o:Hymenoptera,f:Halictidae,g:Lasioglossum,s:Lasioglossum trigeminum
TATACTTTATTTTATTTTTGCTATATGAGCTGGAATAATTGGAGCTTCATTAAGAATAAT
TATTCGAATAGAATTAAGTGCCCCTGGAAAATGAATTAACAATGATCAAATTTATAATAC
CATTATTACTTCACATGCATTTGTAATAATTTTTTTTATAGTTATACCATTTATAATTGG
AGGATTTGGTAATTGACTAGTCCCCTTAATAATTGGAGCACCTGATATAGCATTCCCACG
AATAAATAATATAAGATTTTGATTACTTATCCCATCAATATTTATATTATTAATAAGAAG
TATTATATCATCTGGTTCAGGTACTGGATGAACCGTATACCCACCTTTATCATCTATTAT
ATACCATTCATCAATTTCAGTAGATTACACTATTTTTTCATTACATATTGCAGGAATCTC
ATCTATTATAGGAGCTATTAATTTTATTGTATCTATTTTACTTATAAAAAATATTTCAAT
TAATTATGACCAAATTCCTTTATTTCCATGATCAGTAAAAATTACTGCTATTTTACTATT
ATTATCTTTACCAGTTTTAGCAGGAGCTATTACTATACTTTTAACAGATCGAAACTTAAA
TACTTCATTTTTTGATCCTTCAGGTGGAGGAGACCCTATTCTTTATCAACATCTATTT
>GU708273;tax=d:Eukarya,p:Arthropoda,c:Insecta,o:Hymenoptera,f:Halictidae,g:Lasioglossum,s:Lasioglossum trigeminum
AATTAGGAGCTTCATTAAGAATAATTATTCGAATAGAATTAAGTGCCCCTGGAAAATGAA
TTAACAATGATCAAATTTATAATACCATTATTACTTCACATGCATTTGTAATAATTTTTT
TTATAGTTATACCATTTATAATTGGAGGATTTGGTAATTGACTAGTCCCCTTAATAATTG
GAGCACCTGATATAGCATTCCCACGAATAAATAATATAAGATTTTGATTACTTATCCCAT
CAATATTTATATTATTAATAAGAAGTATTATATCATCTGGTTCAGGTACTGGATGAACCG
TATACCCACCTTTATCATCTATTATATACCATTCATCAATTTCAGTAGATTACACTATTT
TTTCATTACATATTGCAGGAATCTCATCTATTATAGGAGCTATTAATTTTATTGTATCTA
TTTTACTTATAAAAAATATTTCAATTAATTATGACCAAATTCCTTTATTTCCATGATCAG
TAAAAATTACTGCTATTTTACTATTATTATCTTTACCAGTTTTAGCAGGAGCTATTACTA
TACTTTTAACAGATCGAAACTTAAATACTTCATTTTTTGATCCTTCAGGTGGAGGAGACC
CTATTCTTTATCAACATCTATTT
>HM405323;tax=d:Eukarya,p:Arthropoda,c:Insecta,o:Hymenoptera,f:Halictidae,g:Lasioglossum,s:Lasioglossum trigeminum
TATACTTTATTTTATTTTTGCTATATGAGCTGGAATAATTGGAGCTTCATTAAGAATAAT
TATTCGAATAGAATTAAGTGCCCCTGGAAAATGAATTAACAATGATCAAATTTATAATAC
CATTATTACTTCACATGCATTTGTAATAATTTTTTTTATAGTTATACCATTTATGATTGG
AGGATTTGGTAATTGACTAGTCCCTTTAATAATTGGAGCACCTGATATAGCATTCCCACG
AATAAATAATATAAGATTTTGATTACTTATCCCATCAATATTTATATTATTAATAAGAAG
TATTATATCATCTGGTTCAGGTACTGGATGAACCGTATACCCACCTTTATCATCTATTAT
ATACCATTCATCAATTTCAGTAGATTACACTATTTTTTCATTACATATTGCAGGAATCTC
ATCTATTATAGGAGCTATTAATTTTATTGTATCTATTTTACTTATAAAAAATATTTCAAT
TAATTATGACCAAATTCCTTTATTTCCATGATCAGTAAAAATTACTGCTATTTTACTATT
ATTATCTTTACCAGTTTTAGCAGGAGCTATTACTATACTTTTAACAGATCGAAACTTAAA
TACTTCATTTTTTGATCCTTCAGGTGGGGGAGACCCTATTCTTTATCAACATCTATTT
>HM423307;tax=d:Eukarya,p:Arthropoda,c:Insecta,o:Hymenoptera,f:Halictidae,g:Lasioglossum,s:Lasioglossum trigeminum
TATACTTTATTTTATTTTTGCTATATGAGCTGGAATAATTGGAGCTTCATTAAGAATAAT

TATTCGAATAGAATTAAGTGCCCCTGGAAAATGAATTAACAATGATCAAATTTATAATAC
CATTATTACTTCACATGCATTTGTAATAATTTTTTTTATAGTTATACCATTTATAATTGG
AGGATTTGGTAATTGACTAGTCCCTTTAATAATTGGAGCACCTGATATAGCATTCCCACG
AATAAATAATATAAGATTTTGATTACTTATCCCATCAATATTTATATTATTAATAAGAAG
TATTATATCATCTGGTTCAGGTACTGGATGAACCGTATACCCACCTTTATCATCTATTAT
ATACCATTCATCAATTTCAGTAGATTACACTATTTTTTCATTACATATTGCAGGAATCTC
ATCTATTATAGGAGCTATTAATTTTATTGTATCTATTTTACTTATAAAAAATATTTCAAT
TAATTATGACCAAATTCCTTTATTTCCATGATCAGTAAAAATTACTGCTATTTTACTATT
ATTATCTCTACCAGTTTTAGCAGGAGCTATTACTATACTTTTAACAGATCGAAACTTAAA
TACTTCATTTTTTGATCCTTCAGGTGGGGGAGACCCTATTCTTTATCAACATCTATTT
>MH687424;tax=d:Eukarya,p:Arthropoda,c:Insecta,o:Hymenoptera,f:Halictidae,g:Lasioglossum,s:Lasioglossum trigeminum
TGGAAAATGAATTAACAATGATCAAATTTATAATACCATTATTACTTCACATGCATTTGT
AATAATTTTTTTTATAGTTATACCATTTATAATTGGAGGATTTGGTAATTGACTAGTCCC
CTTAATAATTGGAGCACCTGATATAGCATTCCCACGAATAAATAATATAAGATTTTGATT
ACTTATCCCATCAATATTTATATTATTAATAAGAAGTATTATATCATCTGGTTCAGGTAC
TGGATGAACCGTATACCCACCTTTATCATCTATTATATACCATTCATCAATTTCAGTAGA
TTATACTATTTTTTCATTACATATTGCAGGAATCTCATCTATTATAGGAGCTATTAATTT
TATTGTATCTATTTTACTTATAAAAAATATTTCAATTAATTATGACCAAATTCCTTTATT
TCCATGATCAGTAAAAATTACTGCTATTTTACTATTATTATCTTTACCAGTTTTAGCAGG
AGCTATTACTATACTTTTAACAGATCGAAACTTAAATACTTCATTTTTTGACCCTTCAGG
TGGAGGAGACCCTATTCTTTATCAACAT
>GU806805;tax=d:Eukarya,p:Arthropoda,c:Insecta,o:Hymenoptera,f:Apidae,g:Bombus,s:Bombus impatiens
AATGATATATTTTATTTTTGCTATATGATCAGGAATAATCGGATCATCAATAAGATTATT
AATTCGAATAGAACTTAGACATCCAGGAATATGAATTAATAATGATCAAATTTATAATTC
ATTAGTTACAAGACATGCATTTTTAATAATTTTTTTTATAGTTATACCATTTATAATTGG
AGGATTCGGAAATTATTTAATTCCTTTAATATTAGGATCACCTGATATAGCTTTTCCACG
AATAAATAATATTAGATTTTGATTATTACCTCCATCTATTTTTATATTATTATTAAGAAC
TTTATTTACACCTAATGTAGGAACAGGTTGAACCGTATATCCCCCTTTATCATCTTATTT
ATTTCATTCATCACCTTCAGTAGATATTGCAATTTTTTCTTTACATGTAACAGGAATTTC
TTCTATTATTGGTTCATTAAATTTTATCGTTGCTATTATATTAATAAAAAATTTTTCATT
AAATTATGATCAAATTACTTTATTTTCATGATCTGTATGTATTACAGTATTATTATTAAT
TTTATCATTACCAGTTTTAGCAGGAGCAATTACTATACTTCTTTTTGATCGAAATTTTAA
TACATCATTTTTTGATCCAATAGGAGGTGGAGATCCAATTTTATATCAACATTTATTT
>AY165702;tax=d:Eukarya,p:Arthropoda,c:Insecta,o:Hymenoptera,f:Apidae,g:Bombus,s:Bombus impatiens
GCTATATGATCAGGAATAATCGGATCATCAATAAGATTATTAATTCGAATAGAACTTAGA
CATCCAGGAATATGAATTAATAATGATCAAATTTATAATTCATTAGTTACAAGACATGCA
TTTTTAATAATTTTTTTTATAGTTATACCATTTATAATTGGAGGATTCGGAAATTATTTA
ATTCCTTTAATATTAGGATCACCTGATATAGCTTTTCCACGAATAAATAATATTAGATTT
TGATTATTACCTCCATCTATTTTTATATTATTATTAAGAACTTTATTTACACCTAATGTA
GGAACAGGTTGAACCGTATATCCCCCTTTATCATCTTATTTATTTCATTCATCACCTTCA
GTAGATATTGCAATTTTTTCTTTACATGTAACAGGAATTTCTTCTATTATTGGTTCATTA
AATTTTATCGTTGCTATTATATTAATAAAAAATTTTTCATTAAATTATGATCAAATTACT
TTATTTTCATGATCTGTATGTATTACAGTATTATTATTAATTTTATCATTACCAGTTTTA
GCAGGAGCAATTACTATACTTCTTTTTGATCGAAATTTTAATACATCATTTTTTGATCCA
ATAGGAGGTGGAGATCCAATTTTA
>FJ582125;tax=d:Eukarya,p:Arthropoda,c:Insecta,o:Hymenoptera,f:Apidae,g:Bombus,s:Bombus impatiens
TCAGGAATAATCGGATCATCAATAAGATTATTAATTCGAATAGAACTTAGACATCCAGGA
ATATGAATTAATAATGATCAAATTTATAATTCATTAGTTACAAGACATGCATTTTTAATA
ATTTTTTTTATAGTTATACCATTTATAATTGGAGGATTCGGAAATTATTTAATTCCTTTA
ATATTAGGATCACCTGATATAGCTTTTCCACGAATAAATAATATTAGATTTTGATTATTA

CCTCCATCTATTTTTATATTATTATTAAGAACTTTATTTACACCTAATGTAGGAACAGGT
TGAACCGTATATCCCCCTTTATCATCTTATTTATTTCATTCATCACCTTCAGTAGATATT
GCAATTTTTTCTTTACATGTAACAGGAATTTCTTCTATTATTGGTTCATTAAATTTTATC
GTTGCTATTATATTAATAAAAAATTTTTCATTAAATTATGATCAAATTACTTTATTTTCA
TGATCTGTATGTATTACAGTATTATTATTAATTTTATCATTACCAGTTTTAGCAGGAGCA
ATTACTATACTTCTTTTTGATCGAAATTTTAATACATCATTTTTTGATCCAATAGGAGGT
GGAGATCCAATTTTATATCAACATTTATTT
>FJ582126;tax=d:Eukarya,p:Arthropoda,c:Insecta,o:Hymenoptera,f:Apidae,g:Bombus,s:Bombus impatiens
CAGGAATAATCGGATCATCAATAAGATTATTAATTCGAATAGAACTTAGACATCCAGGAA
TATGAATTAATAATGATCAAATTTATAATTCATTAGTTACAAGACATGCATTTTTAATAA
TTTTTTTTATAGTTATACCATTTATAATTGGAGGATTCGGAAATTATTTAATTCCTTTAA
TATTAGGATCACCTGATATAGCTTTTCCACGAATAAATAATATTAGATTTTGATTATTAC
CTCCATCTATTTTTATATTATTATTAAGAACTTTATTTACACCTAATGTAGGAACAGGTT
GAACCGTATATCCCCCTTTATCATCTTATTTATTTCATTCATCACCTTCAGTAGATATTG
CAATTTTTTCTTTACATGTAACAGGAATTTCTTCTATTATTGGTTCATTAAATTTTATCG
TTGCTATTATATTAATAAAAAATTTTTCATTAAATTATGATCAAATTACTTTATTTTCAT
GATCTGTATGTATTACAGTATTATTATTAATTTTATCATTACCAGTTTTAGCAGGAGCAA
TTACTATACTTCTTTTTGATCGAAATTTTAATACATCATTTTTTGATCCAATAGGAGGTG
GAGATCCAATTTTATATCAACATTTATTT
>GU806808;tax=d:Eukarya,p:Arthropoda,c:Insecta,o:Hymenoptera,f:Apidae,g:Bombus,s:Bombus impatiens
AATGATATATTTTATTTTTGCTATATGATCAGGAATAATCGGATCATCAATAAGATTATT
AATTCGAATAGAACTTAGACATCCAGGAATATGAATTAATAATGATCAAATTTATAATTC
ATTAGTTACAAGACATGCATTTTTAATAATTTTTTTTATAGTTATACCATTTATAATTGG
AGGATTCGGAAATTATTTAATTCCTTTAATATTAGGATCACCTGATATAGCTTTTCCACG
AATAAATAATATTAGATTTTGATTATTACCTCCATCTATTTTTATATTATTATTAAGAAC
TTTATTTACACCTAATGTAGGAACAGGTTGAACCGTATATCCCCCTTTATCATCTTATTT
ATTTCATTCATCACCTTCAGTAGATATTGCAATTTTTTCTTTACATGTAACAGGAATTTC
TTCTATTATTGGTTCATTAAATTTTATCGTTGCTATTATATTAATAAAAAATTTTTCATT
AAATTATGATCAAATTACTTTATTTTCATGATCTGTATGTATTACAGTATTATTATTAAT
TTTATCATTACCAGTTTTAGCAGGAGCAATTACTATACTTCTTTTTGATCGAAATTTTAA
TACATCATTTTTTGATCCAATAGGAGGTGGAGATCCAATTATATATCAACATTTATTT
>GU806825;tax=d:Eukarya,p:Arthropoda,c:Insecta,o:Hymenoptera,f:Apidae,g:Bombus,s:Bombus impatiens
AATGATATATTTTATTTTTGCTATATGATCAGGAATAATGGGATCATCAATAAGATTATT
AATTCGAATAGAACTTAGACATCCAGGAATATGAATTAATAATGATCAAATTTATAATTC
ATTAGTTACAAGACATGCATTTTTAATAATTTTTTTTATAGTTATACCATTTATAATTGG
AGGATTCGGAAATTATTTAATTCCTTTAATATTAGGATCACCTGATATAGCTTTTCCACG
AATAAATAATATTAGATTTTGATTATTACCTCCATCTATTTTTATATTATTATTAAGAAC
TTTATTTACACCTAATGTAGGAACAGGTTGAACCGTATATCCCCCTTTATCATCTTATTT
ATTTCATTCATCACCTTCAGTAGATATTGCAATTTTTTCTTTACATGTAACAGGAATTTC
TTCTATTATTGGTTCATTAAATTTTATCGTTGCTATTATATTAATAAAAAATTTTTCATT
AAATTATGATCAAATTACTTTATTTTCATGATCTGTATGTATTACAGTATTATTATTAAT
TTTATCATTACCAGTTTTAGCAGGAGCAATTACTATACTTCTTTTTGATCGAAATTTTAA
TACATCATTTTTTGATCCAATAGGAGGTGGAGATCCAATTTTATATCAACATTTATTT
>HQ978603;tax=d:Eukarya,p:Arthropoda,c:Insecta,o:Hymenoptera,f:Apidae,g:Bombus,s:Bombus impatiens
AATGATATATTTTATTTTTGCTATATGATCAGGAATAATCGGATCATCAATAAGATTATT
AATTCGAATAGAACTTAGACATCCAGGAATATGAATTAATAATGATCAAATTTATAATTC
ATTAGTTACAAGACATGCATTTTTAATAATTTTTTTTATAGTTATACCATTTATAATTGG
AGGATTCGGAAATTATTTAATTCCTTTAATATTAGGATCACCTGATATAGCTTTTCCACG
AATAAATAATATTAGATTTTGATTATTACCTCCATCTATTTTTATATTATTATTAAGAAC
TTTATTTACACCTAATGTAGGAACAGGTTGAACCGTATATCCCCCTTTATCATCTTATTT
ATTTCATTCATCACCTTCAGTAGATATTGCAATTTTTTCTTTACATGTAACAGGAATTTC

TTCTATTATTGGTTCATTAAATTTTATCGTTGCTATTATATTAATAAAAAATTTTTCATT
AAATTATGATCAAATTACTTTATTTTCATGATCTGTATGTATTACAGTATTATTATTAAT
TTTATCATTACCAGTTTTAGCAGGAGCAATTACTATACTTCTTTTTGATCGAAATTTTAA
TA
>JN289111;tax=d:Eukarya,p:Arthropoda,c:Insecta,o:Hymenoptera,f:Apidae,g:Bombus,s:Bombus impatiens
AATGATATATTTTATTTTTGCTATATGATCAGGAATAATCGGATCATCAATAAGATTATT
AATTCGAATAGAACTTAGACATCCAGGAATATGAATTAATAATGATCAAATTTATAATTC
ATTAGTTACAAGACATGCATTTTTAATAATTTTTTTTATAGTTATACCATTTATAATTGG
AGGATTCGGAAATTATTTAATTCCTTTAATATTAGGATCACCTGATATAGCTTTTCCACG
AATAAATAATATTAGATTTTGATTATTACCTCCATCTATTTTTATATTATTATTAAGAAC
TTTATTTACACCTAATGTAGGAACAGGTTGAACCGTATATCCCCCTTTATCATCTTATTT
ATTTCATTCATCACCTTCAGTAGATATTGCAATTTTTTCTTTACATGTAACAGGAATTTC
TTCTATTATTGGTTCATTAAATTTTATCGTTGCTATTATATTAATAAAAAATTTTTCATT
AAATTATGATCAAATTACTTTATTTTCATGATCTGTATGTATTACAGTATTATTATTAAT
TTTATCATTACCAGTTTTAGCAGGAGCAATTACTATACTTCTTTTTGATCGAAATTTTAA
TACATCATTTTTTGATCCAATAGGAGGTGGAGATCCAATT
>KR902312;tax=d:Eukarya,p:Arthropoda,c:Insecta,o:Hymenoptera,f:Apidae,g:Bombus,s:Bombus impatiens
AATAATATATTTTATTTTTGCCATATGATCAGGAATAATTGGATCATCAATAAGATTATT
AATTCGAATAGAACTTAGACATCCAGGAATATGAATTAATAATGATCAAATTTATAACTC
ATTAGTTACTAGACATGCATTTTTAATAATTTTTTTTATAGTTATACCATTTATAATTGG
AGGGTTTGGAAATTATTTAATTCCTTTAATATTAGGATCACCTGATATAGCTTTCCCACG
AATAAATAATATTAGATTTTGATTATTACCTCCATCTATTTTTATATTATTATTAAGAAC
TCTATTTACACCAAATGTAGGAACAGGTTGAACTGTATATCCTCCATTATCATCTTATTT
ATTTCATTCATCACCCTCAGTAGATATTGCAATTTTTTCTTTACATATAACAGGAATTTC
TTCTATTATTGGTTCATTAAATTTTATTGTTACAATTATATTAATAAAAAATTTTTCATT
AAATTATGATCAAATTACTTTATTTTCTTGATCTGTATGTATTACAGTTTTATTATTAAT
TTTATCATTACCAGTTTTAGCTGGAGCAATTACTATACTTCTTTTTGATCGAAATTTTAA
TACATCATTTTTTGATCCAATAGGTGGTGGTGATCCAATTTTATATCAACATTTATTT
>KT604100;tax=d:Eukarya,p:Arthropoda,c:Insecta,o:Hymenoptera,f:Apidae,g:Bombus,s:Bombus impatiens
AGACATCCAGGAATATGAATTAATAATGATCAAATTTATAATTCATTAGTTACAAGACAT
GCATTTTTAATAATTTTTTTTATAGTTATACCATTTATAATTGGAGGATTCGGAAATTAT
TTAATTCCTTTAATATTAGGATCACCTGATATAGCTTTTCCACGAATAAATAATATTAGA
TTTTGATTATTACCTCCATCTATTTTTATATTATTATTAAGAACTTTATTTACACCTAAT
GTAGGAACAGGTTGAACCGTATATCCCCCTTTATCATCTTATTTATTTCATTCATCACCT
TCAGTAGATATTGCAATTTTTTCTTTACATGTAACAGGAATTTCTTCTATTATTGGTTCA
TTAAATTTTATCGTTGCTATTATATTAATAAAAAATTTTTCATTAAATTATGATCAAATT
ACTTTATTTTCATGATCTGTATGTATTACAGTATTATTATTAATTTTATCATTACCAGTT
TTAGCAGGAGCAATTACTATACTTCTTTTTGATCGAAATTTTAATACATCATTTTTTGAT
CCA
>MG439333;tax=d:Eukarya,p:Arthropoda,c:Insecta,o:Hymenoptera,f:Apidae,g:Bombus,s:Bombus impatiens
TATATTTTATTTTTGCTATATGATCAGGAATAATCGGATCATCAATAAGATTATTAATTC
GAATAGAACTTAGACATCCAGGAATATGAATTAATAATGATCAAATTTATAATTCATTAG
TTACAAGACATGCATTTTTAATAATTTTTTTTATAGTTATACCATTTATAATTGGAGGAT
TCGGAAATTATTTAATTCCTTTAATATTAGGATCACCTGATATAGCTTTTCCACGAATAA
ATAATATTAGATTTTGATTATTACCTCCATCTATTTTTATATTATTATTAAGAACTTTAT
TTACACCTAATGTAGGAACAGGTTGAACCGTATATCCCCCTTTATCATCTTATTTATTTC
ATTCATCACCTTCAGTAGATATTGCAATTTTTTCTTTACATATAACAGGAATTTCTTCTA
TTATTGGTTCATTAAATTTTATCGTTACTATTATATTAATAAAAAATTTTTCATTAAATT
ATGATCAAATTACTTTATTTTCATGATCTGTATGTATTACAGTATTATTATTAATTTTAT
CATTACCAGTTTTAGCAGGAGCAATTACTATACTTCTTTTTGATCGAAATTTTAATACAT
CCTTTTTTGATCCAATAAGAGGGGGAGACCCAAATTTT

>MG441708;tax=d:Eukarya,p:Arthropoda,c:Insecta,o:Hymenoptera,f:Apidae,g:Bombus,s:Bombus impatiens
TATATTTTATTTTTGCTATATGATCAGGAATAATCGGATCATCAATAAGATTATTAATTC
GAATAGAACTTAGACATCCAGGAATATGAATTAATAATGATCAAATTTATAATTCATTAG
TTACAAGACATGCATTTTTAATAATTTTTTTTATAGTTATACCATTTATAATTGGAGGAT
TCGGAAATTATTTAATTCCTTTAATATTAGGATCACCTGATATAGCTTTTCCACGAATAA
ATAATATTAGATTTTGATTATTACCTCCATCTATTTTTATATTATTATTAAGAACTTTAT
TTACACCTAATGTAGGAACAGGTTGAACCGTATATCCCCCTTTATCATCTTATTTATTTC
ATTCATCACCTTCAGTAGATATTGCAATTTTTTCTTTACATGTAACAGGAATTTCTTCTA
TTATTGGTTCATTAAATTTTATCGTTGCTATTATATTAATAAAAAATTTTTCATTAAATT
ATGATCAAATTACTTTATTTTCATGATCTGTATGTATTACAGTATTATTATTAATTTTAT
CATTACCAGTTTTAGCAGGAGCAATTACTATACTTCTTTTTGATCGAAATTTTAATACAT
CATTTTTTGATCCAATAGGAGGTGGAGATCCAATTTTATATCAACATTTA
>MG443774;tax=d:Eukarya,p:Arthropoda,c:Insecta,o:Hymenoptera,f:Apidae,g:Bombus,s:Bombus impatiens
ATAATATATTTTATTTTTGCCATATGATCAGGAATAATTGGATCATCAATAAGATTATTA
ATTCGAATAGAACTTAGACATCCAGGAATATGAATTAATAATGATCAAATTTATAACTCA
TTAGTTACTAGACATGCATTTTTAATAATTTTTTTTATAGTTATACCATTTATAATTGGA
GGGTTTGGAAATTATTTAATTCCTTTAATATTAGGATCACCTGATATAGCTTTCCCACGA
ATAAATAATATTAGATTTTGATTATTACCTCCATCTATTTTTATACTATTATTAAGAACT
CTATTTACACCAAATGTAGGAACAGGTTGAACTGTATATCCTCCATTATCATCTTATTTA
TTTCATTCATCACCCTCAGTAGATATTGCAATTTTTTCTTTACATATAACAGGAATTTCT
TCTATTATTGGTTCATTAAATTTTATTGTTACAATTATATTAATAAAAAATTTTTCATTA
AATTATGATCAAATTACTTTATTTTCTTGATCTGTATGTATTACAGTTTTATTATTAATT
TTATCATTACCAGTTTTAGCTGGAGCAATTACTATACTTCTTTTTGATCGA
>MG350005;tax=d:Eukarya,p:Arthropoda,c:Insecta,o:Hymenoptera,f:Megachilidae,g:Megachile,s:Megachile rotundata
TATATATAATTTTTGCTTTATGAGCTGGAATAACTGGATCAAGATTATCTATAATCATTC
GTATAGAATTAAGAACCCCAGGATCTTGAATTAAAAATGATCAAATTTATAATTCCATTG
TTACAGCTCATGCATTTTTAATAATTTTCTTTTTAGTTATACCATTTATAATTGGAGGAT
TTGGTAATTGATTAATACCATTAATAGTAGGAGCACCAGATATAGCATTTCCACGAATAA
ATAATATTAGATTTTGATTATTACCACCATCATTAATTTTATTATTATTAAGAAATTTAA
TTACTCCAAGACCAGGAACTGGTTGAACAGTATATCCACCCTTATCATTATATATATATC
ACCCATCACCTTCTATTGATATAACAATTTTTTCTCTTCATTTATCAGGAATTTCTTCAA
TTATTGGTTCATTAAATTTTATAGTAACAATTTTAATAATAAAAAATTATTCATTAAATT
ATAGTCAAATAACATTATTTCCATGATCAATTTTTATTACAACTATATTACTTTTATTAT
CATTACCAGTACTAGCAGGTGCAATTACAATATTATTATTTGATCGAAATTTAAATACAT
CTTTTTTTGATCCTATAGGTGGAGGAGATCCAATTTTATATCAACATCTA
>MG348455;tax=d:Eukarya,p:Arthropoda,c:Insecta,o:Hymenoptera,f:Megachilidae,g:Megachile,s:Megachile rotundata
TATATAATTTTTGCTTTATGAGCTGGAATAACTGGATCAAGATTATCTATAATCATTCGT
ATAGAATTAAGAACCCCAGGATCTTGAATTAAAAATGATCAAATTTATAATTCCATTGTT
ACAGCTCATGCATTTTTAATAATTTTCTTTTTAGTTATACCATTTATAATTGGAGGATTT
GGTAATTGATTAATACCATTAATAGTAGGAGCACCAGATATAGCATTTCCACGAATAAAT
AATATTAGATTTTGATTATTACCACCATCATTAATTTTATTATTATTAAGAAATTTAATT
ACTCCAAGACCAGGAACTGGTTGAACAGTATATCCACCCTTATCATTATATATATATCAC
CCATCACCTTCTATTGATATAACAATTTTTTCTCTTCATTTATCAGGAATTTCTTCAATT
ATTGGTTCATTAAATTTTATAGTAACAATTTTAATAATAAAAAATTATTCATTAAATTAT
AGTCAAATAACATTATTTCCATGATCAATTTTTATTACAACTATATTACTTTTATTATCA
TTACCAGTACTAGCAGGTGCAATTACAATATTATTATTTGATCGAAATTTAAATACATCT
TTTTTTGATCCTATAGGTGGAGGAGATCCAATTTTATATCAACATCTA
>GU706000;tax=d:Eukarya,p:Arthropoda,c:Insecta,o:Hymenoptera,f:Megachilidae,g:Megachile,s:Megachile rotundata

AATATTATATATAATTTTTGCTTTATGAGCTGGAATAATTGGATCAAGATTATCTATAAT
CATTCGTATAGAATTAAGAACCCCAGGATCTTGAATTAAAAATGATCAAATTTATAATTC
CATTGTTACAGCTCATGCATTTTTAATAATTTTCTTTTTAGTTATACCATTTATAATTGG
AGGATTTGGTAATTGATTAATACCATTAATAGTAGGAGCACCAGATATAGCATTTCCACG
AATAAATAATATTAGATTTTGATTATTACCACCATCATTAATTTTATTATTATTAAGAAA
TTTAATTACTCCAAGACCAGGAACTGGTTGAACAGTATATCCACCCTTATCATTATATAT
ATATCACCCATCACCTTCTATTGATATAACAATTTTTTCTCTTCATTTATCAGGAATTTC
TTCAATTATTGGTTCATTAAATTTTATAGTAACAATTTTAATAATAAAAAATTATTCATT
AAATTATAGTCAAATAACATTATTTCCATGATCAATTTTTATTACAACTATATTACTTTT
ATTATCATTACCAGTACTAGCAGGTGCAATTACAATATTATTATTTGATCGAAATTTAAA
TACATCTTTTTTTGATCCTATAGGTGGAGGAGATCCAATTTTATATCAACATCTATTT
>GU706001;tax=d:Eukarya,p:Arthropoda,c:Insecta,o:Hymenoptera,f:Megachilidae,g:Megachile,s:Megachile
rotundata
AATATTATATATAATTTTTGCTTTATGAGCTGGAATAATTGGATCAAGATTATCTATAAT
CATTCGTATAGAATTAAGAACCCCAGGATCTTGAATTAAAAATGATCAAATTTATAATTC
CATTGTTACAGCTCATGCATTTTTAATAATTTTCTTTTTAGTTATACCATTTATAATTGG
AGGATTTGGTAATTGATTAATACCATTAATAGTAGGAGCACCAGATATAGCATTTCCACG
AATAAATAATATTAGATTTTGATTATTACCACCATCATTAATTTTATTATTATTAAGAAA
TTTAATTACTCCAAGACCAGGAACTGGTTGAACAGTATATCCACCCTTATCATTATATAT
ATATCACCCATCACCTTCTATTGATATAACAATTTTTTCTCTTCATTTATCAGGAATTTC
TTCAATTATTGGTTCATTAAATTTTATAGTAACAATTTTAATAATAAAAAATTATTCATT
AAATTATAGTCAAATAACATTATTTCCATGATCAATTTTTATTACAACTATATTACTTTT
ATTATCATTACCAGTACTAGCAGGTGCAAT
>KJ836559;tax=d:Eukarya,p:Arthropoda,c:Insecta,o:Hymenoptera,f:Megachilidae,g:Megachile,s:Megachile rotundata
AATATTATATATAATTTTTGCTTTATGAGCTGGAATAATTGGATCAAGATTATCTATAAT
CATTCGTATAGAATTAAGAACTCCAGGATCTTGAATTAAAAATGATCAAATTTATAATTC
CATTGTCACAGCTCATGCATTTTTAATAATTTTCTTTTTAGTTATACCATTTATAATTGG
AGGATTTGGTAATTGATTAATACCATTAATAGTAGGAGCACCAGATATAGCATTTCCACG
AATAAATAATATTAGATTTTGATTATTACCACCATCATTAATTTTATTATTATTAAGAAA
TTTAATTACTCCAAGACCAGGAACTGGTTGAACAGTATATCCACCTTTATCATTATATAT
ATATCATCCATCACCTTCTGTTGATATAACAATTTTTTCTCTTCATTTATCAGGAATTTC
TTCAATTATTGGTTCATTAAATTTTATAGTAACAATTTTAATAATAAAAAATTATTCATT
AAATTATAGTCAAATAACATTATTTCCATGATCAATTTTTATTACAACTATATTACTTTT
ATTATCATTACCAGTATTAGCAGGTGCAATTACAATATTATTATTTGATCGAAATTTAAA
TACATCATTTTTTGATCCTATAGGTGGAGGAGATCCAATTTTATATCAACATCTATTT
>KJ837183;tax=d:Eukarya,p:Arthropoda,c:Insecta,o:Hymenoptera,f:Megachilidae,g:Megachile,s:Megachile rotundata
AATATTATATATAATTTTTGCTTTATGAGCTGGAATAATTGGATCAAGATTATCTATAAT
CATTCGTATAGAATTAAGAACTCCAGGATCTTGAATTAAAAATGATCAAATTTATAATTC
CATTGTCACAGCTCATGCATTTTTAATAATTTTCTTTTTAGTTATACCATTTATAATTGG
AGGATTTGGTAATTGATTAATACCATTAATAGTAGGAGCACCAGATATAGCATTTCCACG
AATAAATAATATTAGATTTTGATTATTACCACCATCATTAATTTTATTATTATTAAGAAA
TTTAATTACTCCAAGACCAGGAACTGGTTGAACAGTATATCCACCTTTATCATTATATAT
ATATCATCCATCACCTTCTGTTGATATAACAATTTTTTCTCTTCATTTATCAGGAATTTC
TTCAATTATTGGTTCATTAAATTTTATAGTAACAATTTTAATAATAAAAAATTATTCATT
AAATTATAGTCAAATAACATTATTTCCATGATCAATTTTTATTACAACTATATTACTTTT
ATTATCATTACCAGTATTAGCAGGTGCAATTACAATATTATTATTTGATCGAAATTTAAA
TACATCATTTTTTGATCCTATAGGTGGAGGAGATCCAATTTTATACCAACATCTATTT
>KR877304;tax=d:Eukarya,p:Arthropoda,c:Insecta,o:Hymenoptera,f:Megachilidae,g:Megachile,s:Megachile
rotundata
AATATTATATATAATTTTTGCTTTATGAGCTGGAATAACTGGATCAAGATTATCTATAAT
CATTCGTATAGAATTAAGAACCCCAGGATCTTGAATTAAAAATGATCAAATTTATAATTC

CATTGTTACAGCTCATGCATTTTTAATAATTTTCTTTTTAGTTATACCATTTATAATTGG
AGGATTTGGTAATTGATTAATACCATTAATAGTAGGAGCACCAGATATAGCATTTCCACG
AATAAATAATATTAGATTTTGATTATTACCACCATCATTAATTTTATTATTATTAAGAAA
TTTAATTACTCCAAGACCAGGAACTGGTTGAACAGTATATCCACCCTTATCATTATATAT
ATATCACCCATCACCTTCTATTGATATAACAATTTTTTCTCTTCATTTATCAGGAATTTC
TTCAATTATTGGTTCATTAAATTTTATAGTAACAATTTTAATAATAAAAAATTATTCATT
AAATTATAGTCAAATAACATTATTTCCATGATCAATTTTTATTACAACTATATTACTTTT
ATTATCATTACCAGTA
>KR878725;tax=d:Eukarya,p:Arthropoda,c:Insecta,o:Hymenoptera,f:Megachilidae,g:Megachile,s:Megachile rotundata
ATATTATATATAATTTTTGCTTTATGAGCTGGAATAACTGGATCAAGATTATCTATAATC
ATTCGTATAGAATTAAGAACCCCAGGATCTTGAATTAAAAATGATCAAATTTATAATTCC
ATTGTTACAGCTCATGCATTTTTAATAATTTTCTTTTTAGTTATACCATTTATAATTGGA
GGATTTGGTAATTGATTAATACCATTAATAGTAGGAGCACCAGATATAGCATTTCCACGA
ATAAATAATATTAGATTTTGATTATTACCACCATCATTAATTTTATTATTATTAAGAAAT
TTAATTACTCCAAGACCAGGAACTGGTTGAACAGTATATCCACCCTTATCATTATATATA
TATCACCCATCACCTTCTATTGATATAACAATTTTTTCTCTTCATTTATCAGGAATTTCT
TCAATTATTGGTTCATTAAATTTTATAGTAACAATTTTAATAATAAAAAATTATTCATTA
AATTATAGTCAAATAACATTATTTCCATGATCAATTTTTATTACAACTATATTACTTTTA
TTATCATTACCAGTACTAGCAGGTGCAATTACAATA
>KR878842;tax=d:Eukarya,p:Arthropoda,c:Insecta,o:Hymenoptera,f:Megachilidae,g:Megachile,s:Megachile rotundata
AATATTATATATAATTTTTGCTTTATGAGCTGGAATAACTGGATCAAGATTATCTATAAT
CATTCGTATAGAATTAAGAACCCCAGGATCTTGAATTAAAAATGATCAAATTTATAATTC
CATTGTTACAGCTCATGCATTTTTAATAATTTTCTTTTTAGTTATACCATTTATAATTGG
AGGATTTGGTAATTGATTAATACCATTAATAGTAGGAGCACCAGATATAGCATTTCCACG
AATAAATAATATTAGATTTTGATTATTACCACCATCATTAATTTTATTATTATTAAGAAA
TTTAATTACTCCAAGACCAGGAACTGGTTGAACAGTATATCCACCCTTATCATTATATAT
ATATCACCCATCACCTTCTATTGATATAACAATTTTTTCTCTTCATTTATCAGGAATTTC
TTCAATTATTGGTTCATTAAATTTTATAGTAACAATTTTAATAATAAAAAATTATTCATT
AAATTATAGTCAAATAACATTATTTCCATGATCAATTTTTATTACAACTATATTACTTTT
ATTATCATTACCAGTACTAGCAGGTGCAATTACAATATTATTATTTGATCGAAATTTAAA
TACATCTTTTTTTGATCCTATAGG
>KR880830;tax=d:Eukarya,p:Arthropoda,c:Insecta,o:Hymenoptera,f:Megachilidae,g:Megachile,s:Megachile rotundata
AATATTATATATAATTTTTGCTTTATGAGCTGGAATAACTGGATCAAGATTATCTATAAT
CATTCGTATAGAATTAAGAACCCCAGGATCTTGAATTAAAAATGATCAAATTTATAATTC
CATTGTTACAGCTCATGCATTTTTAATAATTTTCTTTTTAGTTATACCATTTATAATTGG
AGGATTTGGTAATTGATTAATACCATTAATAGTAGGAGCACCAGATATAGCATTTCCACG
AATAAATAATATTAGATTTTGATTATTACCACCATCATTAATTTTATTATTATTAAGAAA
TTTAATTACTCCAAGACCAGGAACTGGTTGAACAGTATATCCACCCTTATCATTATATAT
ATATCACCCATCACCTTCTATTGATATAACAATTTTTTCTCTTCATTTATCAGGAATTTC
TTCAATTATTGGTTCATTAAATTTTATAGTAACAATTTTAATAATAAAAAATTATTCATT
AAATTATAGTCAAATAACATTATTTCCATGATCAATTTTTATTACAACTATATTACTTTT
ATTATCATTA
>KR882246;tax=d:Eukarya,p:Arthropoda,c:Insecta,o:Hymenoptera,f:Megachilidae,g:Megachile,s:Megachile rotundata
AATATTATATATAATTTTTGCTTTATGAGCTGGAATAACTGGATCAAGATTATCTATAAT
CATTCGTATAGAATTAAGAACCCCAGGATCTTGAATTAAAAATGATCAAATTTATAATTC
CATTGTTACAGCTCATGCATTTTTAATAATTTTCTTTTTAGTTATACCATTTATAATTGG
AGGATTTGGTAATTGATTAATACCATTAATAGTAGGAGCACCAGATATAGCATTTCCACG

AATAAATAATATTAGATTTTGATTATTACCACCATCATTAATTTTATTATTATTAAGAAA
TTTAATTACTCCAAGACCAGGAACTGGTTGAACAGTATATCCACCCTTATCATTATATAT
ATATCACCCATCACCTTCTATTGATATAACAATTTTTTCTCTTCATTTATCAGGAATTTC
TTCAATTATTGGTTCATTAAATTTTATAGTAACAATTTTAATAATAAAAAATTATTCATT
AAATTATAGTCAAATAACATTATTTCCATGATCAATTTTTATTACAACTATATTACTTTT
ATTATCATTACCAGTACTAGCAGGTGCAATTACAATATTATTATATTTGAT
>KR883667;tax=d:Eukarya,p:Arthropoda,c:Insecta,o:Hymenoptera,f:Megachilidae,g:Megachile,s:Megachile rotundata
AATATTATATATATAATTTTTGCTTTATGAGCTGGAATAACTGGATCAAGATTATCTATAAT
CATTCGTATAGAATTAAGAACCCCAGGATCTTGAATTAAAAATGATCAAATTTATAATTC
CATTGTTACAGCTCATGCATTTTTAATAATTTTCTTTTTAGTTATACCATTTATAATTGG
AGGATTTGGTAATTGATTAATACCATTAATAGTAGGAGCACCAGATATAGCATTTCCACG
AATAAATAATATTAGATTTTGATTATTACCACCATCATTAATTTTATTATTATTAAGAAA
TTTAATTACTCCAAGACCAGGAACTGGTTGAACAGTATATCCACCCTTATCATTATATAT
ATATCACCCATCACCTTCTATTGATATAACAATTTTTTCTCTTCATTTATCAGGAATTTC
TTCAATTATTGGTTCATTAAATTTTATAGTAACAATTTTAATAATAAAAAATTATTCATT
AAATTATAGTCAAATAACATTATTTCCATGATCAATTTTTATTACAACTATATTACTTTT
ATTATCATTACCAGTACTAGC
>KR892976;tax=d:Eukarya,p:Arthropoda,c:Insecta,o:Hymenoptera,f:Megachilidae,g:Megachile,s:Megachile rotundata
ATATTATATATATAATTTTTGCTTTATGAGCTGGAATAATTGGATCAAGATTATCTATAATC
ATTCGTATAGAATTAAGAACCCCAGGATCTTGAATTAAAAATGATCAAATTTATAATTCC
ATTGTTACAGCTCATGCATTTTTAATAATTTTCTTTTTAGTTATACCATTTATAATTGGA
GGATTTGGTAATTGATTAATACCATTAATAGTAGGAGCACCAGATATAGCATTTCCACGA
ATAAATAATATTAGATTTTGATTATTACCACCATCATTAATTTTATTATTATTAAGAAAT
TTAATTACTCCAAGACCAGGAACTGGTTGAACAGTATATCCACCCTTATCATTATATATA
TATCACCCATCACCTTCTATTGATATAACAATTTTTTCTCTTCATTTATCAGGAATTTCT
TCAATTATTGGTTCATTAAATTTTATAGTAACAATTTTAATAATAAAAAATTATTCATTA
AATTATAGTCAAATAACATTATTTCCATGATCAATTTTTATTACAACTATATTACTTTTA
TTATCATTACCAGTACTAGCAGGTGCAATTACAATATTATTATATTTGATCGA
>KR898304;tax=d:Eukarya,p:Arthropoda,c:Insecta,o:Hymenoptera,f:Megachilidae,g:Megachile,s:Megachile rotundata
AATATTATATATATAATTTTTGCTTTATGAGCTGGAATAACTGGATCAAGATTATCTATAAT
CATTCGTATAGAATTAAGAACCCCAGGATCTTGAATTAAAAATGATCAAATTTATAATTC
CATTGTTACAGCTCATGCATTTTTAATAATTTTCTTTTTAGTTATACCATTTATAATTGG
AGGATTTGGTAATTGATTAATACCATTAATAGTAGGAGCACCAGATATAGCATTTCCACG
AATAAATAATATTAGATTTTGATTATTACCACCATCATTAATTTTATTATTATTAAGAAA
TTTAATTACTCCAAGACCAGGAACTGGTTGAACAGTATATCCACCCTTATCATTATATAT
ATATCACCCATCACCTTCTATTGATATAACAATTTTTTCTCTTCATTTATCAGGAATTTC
TTCAATTATTGGTTCATTAAATTTTATAGTAACAATTTTAATAATAAAAAATTATTCATT
AAATTATAGTCAAATAACATTATTTCCATGATCAATTTTTATTACAACTATATTACTTTT
ATTATCATTACCAGTACTAGCAGGTGCAATTACAATATTATTATATTTGATCGA
>KR901310;tax=d:Eukarya,p:Arthropoda,c:Insecta,o:Hymenoptera,f:Megachilidae,g:Megachile,s:Megachile rotundata
ATAATTTTTGCTTTATGAGCTGGAATAACTGGATCAAGATTATCTATAATCATTCGTATA
GAATTAAGAACCCCAGGATCTTGAATTAAAAATGATCAAATTTATAATTCCATTGTTACA
GCTCATGCATTTTTAATAATTTTCTTTTTAGTTATACCATTTATAATTGGAGGATTTGGT
AATTGATTAATACCATTAATAGTAGGAGCACCAGATATAGCATTTCCACGAATAAATAAT
ATTAGATTTTGATTATTACCACCATCATTAATTTTATTATTATTAAGAAATTTAATTACT
CCAAGACCAGGAACTGGTTGAACAGTATATCCACCCTTATCATTATATATATATCACCCA
TCACCTTCTATTGATATAACAATTTTTTCTCTTCATTTATCAGGAATTTCTTCAATTATT

GGTTCATTAAATTTTATAGTAACAATTTTAATAATAAAAAATTATTCATTAAATTATAGT
CAAATAACATTATTTCCATGATCAATTTTTATTACAACTATATTACTTTTATTATCATTA
CCAGTACTAGCAGGT
>GU707506;tax=d:Eukarya,p:Arthropoda,c:Insecta,o:Hymenoptera,f:Apidae,g:Ceratina,s:Ceratina calcarata
AATTTTATATATTATATTTGCTATATGATCAGGTATAATCGGAGCATCAATAAGATTAAT
TATTCGAATAGAATTAAGAACCCCAGGAAATTGAATTAATAATGATCAAATTTATAACTC
ATTAGTTACTGCTCATGCCTTTTTAATAATTTTTTTTATAGTTATACCATTTATAATTGG
GGGATTTGGAAATTGATTAATCCCTCTAATATTAGGATCACCAGATATATCTTTTCCTCG
TTTAAATAATATTAGTTTTTGATTATTACCCCCATCATTACTACTATTATTATTAAGAAA
TTTATTTTCAATAAGACCAGGAACAGGATGAACTGTTTATCCACCTTTATCATCATATTT
ATTTCACCCATCCCCATCAGTAGACTTAGCTATTTTTTCCTTACATATATCAGGAATTTC
ATCAATTTTAGGAGCTATTAATTTTATAGTTACTATTATAATAATAAAAAATATTTCATT
AAATTATGACTCTATTCCATTATTTTCATGAGCAGTATTTATTACAGCAATTTTATTACT
TTTATCATTACCAGTATTAGCAGGAGCTATTACTATATTATTATTTGATCGAAATTTAAA
TACATCCTTTTTTGATCCTATAGGAGGTGGAGATCCAATTTTATATCAACATTTATTT
>GU707514;tax=d:Eukarya,p:Arthropoda,c:Insecta,o:Hymenoptera,f:Apidae,g:Ceratina,s:Ceratina calcarata
AATTTTATATATTATATTTGCTATATGATCAGGTATAATCGGAGCATCAATAAGATTAAT
TATTCGAATAGAATTAAGAACCCCAGGAAATTGAATTAATAATGATCAAATTTATAACTC
ATTAGTTACTGCTCATGCCTTTTTAATAATTTTTTTTATAGTTATACCATTTATAATTGG
GGGATTTGGAAATTGATTAATCCCTCTAATATTAGGATCACCAGATATATCTTTTCCTCG
TTTAAATAATATTAGTTTTTGATTATTACCCCCATCATTACTACTATTATTATTAAGAAA
TTTATTTTCAATAAGACCAGGAACAGGATGAACTGTTTATCCACCTTTATCATCATATTT
ATTTCACCCATCCCCATCAGTAGACTTAGCTATTTTTTCCTTACATATATCAGGAATTTC
ATCAATTTTAGGAGCTATTAATTTTATAGTTACTATTATAATAATAAAAAATATTTCATT
AAATTATGACTCTATTCCATTATTTTCATGAGCAGTATTTATTACAGCAATTTTATTACT
TTTATCATTACCAGTATTAGCAGGAGCTATTACTATATTATTATTTGATCGAAATTTAAA
TACATCCTTTTTTG
>FJ582163;tax=d:Eukarya,p:Arthropoda,c:Insecta,o:Hymenoptera,f:Apidae,g:Ceratina,s:Ceratina calcarata
AATTTTATATATTATATTTGCTATATGATCAGGTATAATCGGAGCATCAATAAGATTAAT
TATTCGAATAGAATTAAGAACCCCAGGAAATTGAATTAATAATGATCAAATTTATAACTC
ATTAGTTACTGCTCATGCCTTTTTAATAATTTTTTTTATAGTTATACCATTTATAATTGG
AGGATTTGGAAATTGATTAATCCCTCTAATATTAGGATCACCAGATATATCTTTTCCTCG
TTTAAATAATATTAGTTTTTGATTATTACCCCCATCATTACTACTATTATTATTAAGAAA
TTTATTTTCAATAAGACCAGGAACAGGATGAACTGTTTATCCACCTTTATCATCATATTT
ATTTCACCCATCTCCATCAGTAGACTTAGCTATTTTTTCCTTACATATGTCAGGAATTTC
ATCAATTTTAGGAGCTATTAATTTTATAGTTACTATTATAATAATAAAAAATATTTCATT
AAATTATGACTCTATTCCATTATTTTCATGAGCAGTATTTATTACAGCAATTTTATTACT
TTTATCATTACCAGTATTAGCAGGAGCTATTACTATATTATTATTTGATCGAAATTTAAA
TACATCCTTTTTTGATCCTATAGGAGGTGGAGATCCAATTTTATATCAACATTTATTT
>FJ582166;tax=d:Eukarya,p:Arthropoda,c:Insecta,o:Hymenoptera,f:Apidae,g:Ceratina,s:Ceratina calcarata
GCTATATGATCAGGTATAATCGGAGCATCAATAAGATTAATTATTCGAATAGAATTAAGA
ACCCCAGGAAATTGAATTAATAATGATCAAATTTATAACTCATTAGTTACTGCTCATGCC
TTTTTAATAATTTTTTTTATAGTTATACCATTTATAATTGGAGGATTTGGAAATTGATTA
ATCCCTCTAATATTAGGATCACCAGATATATCTTTTCCTCGTTTAAATAATATTAGTTTT
TGATTATTACCCCCATCATTACTACTATTATTATTAAGAAATTTATTTTCAATAAGACCA
GGAACAGGATGAACTGTTTATCCACCTTTATCATCATATTTATTTCACCCATCTCCATCA
GTAGACTTAGCTATTTTTTCCTTACATATGTCAGGAATTTCATCAATTTTAGGAGCTATT
AATTTTATAGTTACTATTATAATAATAAAAAATATTTCATTAAATTATGACTCTATTCCA
TTATTTTCATGAGCAGTATTTATTACAGCAATTTTATTACTTTTATCATTACCAGTATTA
GCAGGAGCTATTACTATATTATTATTTGATCGAAATTTAAATACATCCTTTTTTGATCCT
ATAGGAGGTGGAGATCCAATTTTATATCAACATTTATTT

>FJ582167;tax=d:Eukarya,p:Arthropoda,c:Insecta,o:Hymenoptera,f:Apidae,g:Ceratina,s:Ceratina calcarata
AATCGGAGCATCAATAAGATTAATTATTCGAATAGAATTAAGAACCCCAGGAAATTGAAT
TAATAATGATCAAATTTATAACTCATTAGTTACTGCTCATGCCTTTTTAATAATTTTTTT
TATAGTTATACCATTTATAATTGGAGGATTTGGAAATTGATTAATCCCTCTAATATTAGG
ATCACCAGATATATCTTTTCCTCGTTTAAATAATATTAGTTTTTGATTATTACCCCCATC
ATTACTACTATTATTATTAAGAAATTTATTTTCAATAAGACCAGGAACAGGATGAACTGT
TTATCCACCTTTATCATCATATTTATTTCACCCATCTCCATCAGTAGACTTAGCTATTTT
TTCCTTACATATGTCAGGAATTTCATCAATTTTAGGAGCTATTAATTTTATAGTTACTAT
TATAATAATAAAAAATATTTCATTAAATTATGACTCTATTCCATTATTTTCATGAGCAGT
ATTTATTACAGCAATTTTATTACTTTTATCATTACCAGTATTAGCAGGAGCTATTACTAT
ATTATTATTTGATCGAAATTTAAATACATCCTTTTTTGATCCTATAGGAGGTGGAGATCC
AATTTTATATCAACATTTATTT
>GU707477;tax=d:Eukarya,p:Arthropoda,c:Insecta,o:Hymenoptera,f:Apidae,g:Ceratina,s:Ceratina calcarata
AATTTTATATATTATATTTGCTATATGATCAGGTATAATCGGAGCATCAATAAGATTAAT
TATTCGAATAGAATTAAGAACCCCGGGAAATTGAATTAATAATGATCAAATTTATAACTC
ATTAGTTACTGCTCATGCCTTTTTAATAATTTTTTTTATAGTTATACCATTTATAATTGG
GGGATTTGGAAATTGATTAATCCCTCTAATATTAGGATCACCAGATATATCTTTTCCTCG
TTTAAATAATATTAGTTTTTGATTATTACCCCCATCATTACTACTATTATTATTAAGAAA
TTTATTTTCAATAAGACCAGGAACAGGATGAACTGTTTATCCACCTTTATCATCATATTT
ATTTCACCCATCCCCATCAGTAGACTTAGCTATTTTTTCCTTACATATATCAGGAATTTC
ATCAATTTTAGGAGCTATTAATTTTATAGTTACTATTATAATAATAAAAAATATTTCATT
AAATTATGACTCTATTCCATTATTTTCATGAGCAGTATTTATTACGGCAATTTTATTACT
TTTATCATTACCAGTATTAGCAGGAGCTATTACTATATTATTATTTGATCGAAATTTAAA
TACATCCTTTTTTGATCCTATAGGAGGTGGAGATCCAATTTTATATCAACATTTATTT
>GU707484;tax=d:Eukarya,p:Arthropoda,c:Insecta,o:Hymenoptera,f:Apidae,g:Ceratina,s:Ceratina calcarata
AATTTTATATATTATATTTGCTATATGATCAGGTATAATCGGAGCATCAATAAGATTAAT
TATTCGAATAGAATTAAGAACCCCGGGAAATTGAATTAATAATGATCAAATTTATAACTC
ATTAGTTACTGCTCATGCCTTTTTAATAATTTTTTTTATAGTTATACCATTTATAATTGG
GGGATTTGGAAATTGATTAATCCCTCTAATATTAGGATCACCAGATATATCTTTTCCTCG
TTTAAATAATATTAGTTTTTGATTATTACCCCCATCATTACTACTATTATTATTAAGAAA
TTTATTTTCAATAAGACCAGGAACAGGATGAACTGTTTATCCACCTTTATCATCATATTT
ATTTCACCCATCCCCATCAGTAGACTTAGCTATTTTTTCCTTACATATATCAGGAATTTC
ATCAATTTTAGGAGCTATTAATTTTATAGTTACTATTATAATAATAAAAAATATTTCATT
AAATTATGACTCTATTCCATTATTTTCATGAGCAGTATTTATTACAGCAATTTTATTACT
TTTATCATTACCAGTATTAGCAGGAGCTATTACTATATTATTATTTGATCGAAATTTAAA
TACATCCTTTTTTGATCCTATAGGAGGTGGAGATCCAATTTTATATCAACATTTATTT
>GU707486;tax=d:Eukarya,p:Arthropoda,c:Insecta,o:Hymenoptera,f:Apidae,g:Ceratina,s:Ceratina calcarata
AATTTTATATATTATATTTGCTATATGATCAGGTATAATCGGAGCATCAATAAGATTAAT
TATTCGAATAGAATTAAGAACCCCGGGAAATTGAATTAATAATGATCAAATTTATAACTC
ATTAGTTACTGCTCATGCCTTTTTAATAATTTTTTTTATAGTTATACCATTTATAATTGG
GGGATTTGGAAATTGATTAATCCCTCTAATATTAGGATCACCAGATATATCTTTTCCTCG
TTTAAATAATATTAGTTTTTGATTATTGCCCCCATCATTACTACTATTATTATTAAGAAA
TTTATTTTCAATAAGACCAGGAACAGGATGAACTGTTTATCCACCTTTATCATCATATTT
ATTTCACCCATCCCCATCAGTAGACTTAGCTATTTTTTCCTTACATATATCAGGAATTTC
ATCAATTTTAGGAGCTATTAATTTTATAGTTACTATTATAATAATAAAAAATATTTCATT
AAATTATGACTCTATTCCATTATTTTCATGAGCAGTATTTATTACAGCAATTTTATTACT
TTTATCATTACCAGTATTAGCAGGAGCTATTACTATATTATTATTTGATCGAAATTTAAA
TACATCCTTTTTTGATCCTATAGGAGGTGGAGATCCAATTTTATATCAACATTTATTT
>GU707505;tax=d:Eukarya,p:Arthropoda,c:Insecta,o:Hymenoptera,f:Apidae,g:Ceratina,s:Ceratina calcarata
AATTTTATATATTATATTTGCTATATGATCAGGTATAATCGGAGCATCAATAAGATTAAT
TATTCGAATAGAATTAAGAACCCCAGGAAATTGAATTAATAATGATCAAATTTATAACTC

ATTAGTTACTGCTCATGCCTTTTTAATAATTTTTTTTATAGTTATACCATTTATAATTGG
AGGATTTGGAAATTGATTAATCCCTCTAATATTAGGATCACCAGATATATCTTTTCCTCG
TTTAAATAATATTAGTTTTTGATTATTACCCCCATCATTACTACTATTATTATTAAGAAA
TTTATTTTCAATAAGACCGGGAACAGGATGAACTGTTTATCCACCTTTATCATCATATTT
ATTTCACCCATCTCCATCAGTAGACTTAGCTATTTTTTCCTTACATATGTCAGGAATTTC
ATCAATTTTAGGAGCTATTAATTTTATAGTTACTATTATAATAATAAAAAATATTTCATT
AAATTATGACTCTATTCCATTATTTTCATGAGCAGTATTTATTACAGCAATTTTATTACT
TTTATCATTACCAGTATTAGCAGGAGCTATTACTATATTATTATTTGATCGAAATTTAAA
TACATCCTTTTTTGATCCTATAGGAGGTGGAGATCCAATTTTATATCAACATTTATTT
>GU707513;tax=d:Eukarya,p:Arthropoda,c:Insecta,o:Hymenoptera,f:Apidae,g:Ceratina,s:Ceratina calcarata
AATTTTATATATTATATTTGCTATATGATCAGGTATAATCGGAGCATCAATAAGATTAAT
TATTCGAATAGAATTAAGAACCCCAGGAAATTGAATTAATAATGATCAAATTTATAACTC
ATTAGTTACTGCTCATGCCTTTTTAATAATTTTTTTTATAGTTATACCATTTATAATTGG
GGGATTTGGAAATTGATTAATCCCTCTAATATTAGGCTCACCAGATATATCTTTTCCTCG
TTTAAATAATATTAGTTTTTGATTATTACCCCCATCATTACTACTATTATTATTAAGAAA
TTTATTTTCAATAAGACCAGGAACAGGATGAACTGTTTATCCACCTTTATCATCATATTT
ATTTCACCCGTCTCCATCAGTAGATTTAGCTATTTTTTCCTTACATATATCAGGAATTTC
ATCAATTTTAGGAGCTATTAATTTTATAGTTACTATTATAATAATAAAAAATATTTCATT
AAATTATGACTCTATTCCATTATTTTCATGAGCAGTATTTATTACAGCAATTTTATTACT
TTTATCATTACCAGTATTAGCAGGAGCTATTACTATATTATTATTTGATCGAAATTTAAA
TACATCCTTTTTTGATCCTATAGGAGGGGGGGATCCAATTTTATATCAACATTTATTT
>GU707517;tax=d:Eukarya,p:Arthropoda,c:Insecta,o:Hymenoptera,f:Apidae,g:Ceratina,s:Ceratina calcarata
AATTTTATATATTATATTTGCTATATGATCAGGTATAATCGGAGCATCAATAAGATTAAT
TATTCGAATAGAATTAAGAACCCCAGGAAATTGAATTAATAATGACCAAATTTATAACTC
ATTAGTTACTGCTCATGCCTTTTTAATAATTTTTTTTATAGTTATACCATTTATAATTGG
AGGATTTGGAAATTGATTAATCCCTCTAATATTAGGATCACCAGATATATCTTTTCCTCG
TTTAAATAATATTAGTTTTTGATTATTACCCCCATCATTACTACTATTATTATTAAGAAA
TTTATTTTCAATAAGACCAGGAACAGGATGAACTGTTTATCCACCTTTATCATCATATTT
ATTTCACCCATCTCCATCAGTAGACTTAGCTATTTTTTCCTTACATATGTCAGGAATTTC
ATCAATTTTAGGAGCTATTAATTTTATAGTTACTATTATAATAATAAAAAATATTTCATT
AAATTATGACTCTATTCCATTATTTTCATGAGCAGTATTTATTACAGCAATTTTATTACT
TTTATCATTACCAGTATTAGCAGGAGCTATTACTATATTATTATTTGATCGAAATTTAAA
TACATCCTTTTTTGATCCTATAGGAGGTGGAGATCCAATTTTATATCAACATTTATTT
>GU707521;tax=d:Eukarya,p:Arthropoda,c:Insecta,o:Hymenoptera,f:Apidae,g:Ceratina,s:Ceratina calcarata
AATTTTATATATTATATTTGCTATATGATCAGGTATAATCGGAGCATCAATAAGATTAAT
TATTCGAATAGAATTAAGAACCCCGGGAAATTGAATTAATAATGATCAAATTTATAACTC
ATTAGTTACTGCTCATGCCTTTTTAATAATTTTTTTTATAGTTATACCATTTATAATTGG
GGGATTTGGAAATTGATTAATCCCTCTAATATTAGGATCACCAGATATATCTTTTCCTCG
TTTAAATAATATTAGTTTTTGATTATTACCCCCATCATTACTACTATTATTATTAAGAAA
TTTATTTTCAATAAGACCAGGAACAGGATGAACTGTTTATCCACCTTTATCATCATATTT
ATTTCACCCATCCCCATCAGTAGATTTAGCTATTTTTTCCTTACATATATCAGGAATTTC
ATCAATTTTAGGAGCTATTAATTTTATAGTTACTATTATAATAATAAAAAATATTTCATT
AAATTATGACTCTATTCCATTATTTTCATGAGCAGTATTTATTACAGCAATTTTATTACT
TTTATCATTACCAGTATTAGCAGGGGCTATTACTATATTATTATTTGATCGAAATTTAAA
TACATCCTTTTTTGATCCTATAGGAGGTGGAGATCCAATTTTATATCAACATTTATTT
>GU707522;tax=d:Eukarya,p:Arthropoda,c:Insecta,o:Hymenoptera,f:Apidae,g:Ceratina,s:Ceratina calcarata
AATTTTATATATTATATTTGCTATATGATCAGGTATAATCGGAGCATCAATAAGATTAAT
TATTCGAATAGAATTAAGAACCCCAGGAAATTGAATTAATAATGATCAAATTTATAACTC
ATTAGTTACTGCTCATGCCTTTTTAATAATTTTTTTTATAGTTATACCATTTATAATTGG
AGGATTTGGAAATTGATTAATCCCTCTAATATTAGGATCACCAGATATATCTTTTCCTCG
TTTAAATAATATTAGTTTTTGATTATTACCCCCATCATTACTACTATTATTATTAAGAAA

TTTATTTTCAATAAGACCAGGAACAGGGTGAACTGTTTATCCACCTTTATCATCATATTT
ATTTCACCCATCTCCATCAGTAGACTTAGCTATTTTTTCCTTACATATATCAGGAATTTC
ATCAATTTTAGGAGCTATTAATTTTATAGTTACTATTATAATAATAAAAAATATTTCATT
AAATTATGACTCTATTCCATTATTTTCATGAGCAGTATTTATTACAGCAATTTTATTACT
TTTATCATTACCAGTATTAGCAGGAGCTATTACTATATTATTATTTGATCGAAATTTAAA
TACATCCTTTTTTGATCCTATAGGAGGTGGAGATCCAATTTTATATCAACATTTATTT
>GU707524;tax=d:Eukarya,p:Arthropoda,c:Insecta,o:Hymenoptera,f:Apidae,g:Ceratina,s:Ceratina calcarata
AATTTTATATATTATATTTGCTATATGATCAGGTATAATCGGAGCATCAATAAGATTAAT
TATTCGAATAGAATTAAGAACCCCGGGAAATTGAATTAATAATGATCAAATTTATAACTC
ATTAGTTACTGCTCATGCCTTTTTAATAATTTTTTTTATAGTTATACCATTTATAATTGG
GGGATTTGGAAATTGATTAATCCCTCTAATATTAGGATCACCAGATATATCTTTTCCTCG
TTTAAATAATATTAGTTTTTGATTATTACCCCCATCATTACTACTATTATTATTAAGAAA
TTTATTTTCAATAAGACCAGGAACAGGATGAACTGTTTATCCACCTTTATCATCATATTT
ATTTCACCCATCCCCATCAGTAGACTTAGCTATTTTTTCCTTACATATATCAGGAATTTC
ATCAATTTTAGGAGCTATTAATTTTATAGTTACTATTATAATAATAAAAAATATTTCATT
AAATTATGACTCTATTCCATTATTTTCATGAGCAGTATTTATTACAGCAATTTTATTACT
TTTATCATTACCAGTATTAGCAGGGGCTATTACTATATTATTATTTGATCGAAATTTAAA
TACATCCTTTTTTGATCCTATAGGAGGTGGAGATCCAATTTTATATCAACATTTATTT
>GU707525;tax=d:Eukarya,p:Arthropoda,c:Insecta,o:Hymenoptera,f:Apidae,g:Ceratina,s:Ceratina calcarata
AATTTTATATATTATATTTGCTATATGATCAGGTATAATCGGAGCATCAATAAGATTAAT
TATTCGAATAGAATTAAGAACCCCAGGAAATTGAATTAATAATGACCAAATTTATAACTC
ATTAGTTACTGCTCATGCCTTTTTAATAATTTTTTTTATAGTTATACCATTTATAATTGG
AGGATTTGGAAATTGATTAATCCCTCTAATATTAGGATCACCAGATATATCTTTTCCTCG
TTTAAATAATATTAGTTTTTGATTATTACCCCCATCATTACTACTATTATTATTAAGAAA
TTTATTTTCAATAAGACCAGGAACAGGATGAACTGTTTATCCACCTTTATCATCATATTT
ATTTCACCCATCTCCATCAGTAGACTTAGCTATTTTTTCCTTACATATGTCAGGAATTTC
ATCAATTTTAGGAGCTATTAATTTTATAGTTACTATTATAATAATAAAAAATATTTCATT
AAATTATGACTCTATTCCATTATTTTCATGAGCAGTATTTATTACAGCAATTTTATTACT
TTTATCATTACCAGTATTAGCAGGAGCTATTACTATATTATTATTTGATCGAAATTTAAA
TACATCTTTTTTTGATCCTATAGGAGGTGGAGATCCAATTTTATATCAACATTTATTT
>GU707536;tax=d:Eukarya,p:Arthropoda,c:Insecta,o:Hymenoptera,f:Apidae,g:Ceratina,s:Ceratina calcarata
AATTTTATATATTATATTTGCTATATGATCAGGTATAATCGGAGCATCAATAAGATTAAT
TATTCGAATAGAATTAAGAACCCCGGGAAATTGAATTAATAATGATCAAATTTATAACTC
ATTAGTTACTGCTCATGCCTTTTTAATAATTTTTTTTATAGTTATACCATTTATAATTGG
GGGATTTGGAAATTGATTAATCCCTCTAATATTAGGATCACCAGATATATCTTTTCCTCG
TTTAAATAATATTAGTTTTTGATTATTACCCCCATCATTACTATTATTATTATTAAGAAA
TTTATTTTCAATAAGACCAGGAACAGGATGAACTGTTTATCCACCTTTATCATCATATTT
ATTTCACCCATCCCCATCAGTAGACTTAGCTATTTTTTCCTTACATATATCAGGAATTTC
ATCAATTTTAGGAGCTATTAATTTTATAGTTACTATCATAATAATAAAAAATATTTCATT
AAATTATGACTCTATTCCATTATTTTCATGAGCAGTATTTATTACAGCAATTTTATTACT
TTTATCATTACCAGTATTAGCAGGAGCTATTACTATATTATTATTTGATCGAAATTTAAA
TACATCCTTTTTTGATCCTATAGGAGGTGGAGATCCAATTTTATATCAACATTTATTT
>JF271016;tax=d:Eukarya,p:Arthropoda,c:Insecta,o:Hymenoptera,f:Apidae,g:Ceratina,s:Ceratina calcarata
AATTTTATATATTATATTTGCTATATGATCAGGTATAATCGGAGCATCAATAAGATTAAT
TATTCGAATAGAATTAAGAACCCCAGGAAATTGAATTAATAATGATCAAATTTATAACTC
ATTAGTTACTGCTCATGCCTTTTTAATAATTTTTTTTATAGTTATACCATTTATAATTGG
GGGATTTGGAAATTGATTAATCCCTCTAATATTAGGATCACCAGATATATCTTTCCCTCG
TTTAAATAATATTAGTTTTTGATTATTACCCCCATCATTACTACTATTATTATTAAGAAA
TTTATTTTCAATAAGACCAGGAACAGGATGAACTGTTTATCCACCTTTATCATCATATTT
ATTTCACCCATCCCCATCAGTAGACTTAGCTATTTTTTCCTTACATATATCAGGAATTTC
ATCAATTTTAGGAGCTATTAATTTTATAGTTACTATTATAATAATAAAAAATATTTCATT

AAATTATGACTCTATTCCATTATTTTCATGAGCAGTATTTATTACAGCAATTTTATTACT
TTTATCATTACCAGTATTAGCAGGAGCTATTACTATATTATTATTTGATCGAAATTTAAA
TACATCCTTTTTTGATCCTATAGGAGGTGGAGATCCAATTTTATATCAACATTTATTT
>JF271017;tax=d:Eukarya,p:Arthropoda,c:Insecta,o:Hymenoptera,f:Apidae,g:Ceratina,s:Ceratina calcarata
ATATGATCAGGTATAATCGGAGCATCAATAAGATTAATTATTCGAATAGAATTAAGAACC
CCGGGAAATTGAATTAATAATGATCAAATTTATAACTCATTAGTTACTGCTCATGCCTTT
TTAATAATTTTTTTTATAGTTATACCATTTATAATTGGGGGATTTGGAAATTGATTAATC
CCTCTAATATTAGGATCACCAGATATATCTTTTCCTCGTTTAAATAATATTAGTTTTTGA
TTATTACCCCCATCATTACTACTATTATTATTAAGAAATTTATTTTCAATAAGACCAGGA
ACAGGATGAACTGTTTATCCACCTTTATCATCATATTTATTTCACCCATCCCCATCAGTA
GACTTAGCTATTTTTTTCCTTACATATATCAGGAATTTCATCAATTTTAGGAGCTATTAAT
TTTATAGTTACTATTATAATAATAAAAAATATTTCATTAAATTATGACTCTATTCCATTA
TTTTCATGAGCAGTATTTATTACAGCAATTTTATTACTTTTATCATTACCAGTATTAGCA
GGAGCTATTACTATATTATTATTTGATCGAAATTTAAATACATCCTTTTTTGATCCTATA
GGAGGTGGAGATCCAATTTTATATCAACATTTATTT
>JF271020;tax=d:Eukarya,p:Arthropoda,c:Insecta,o:Hymenoptera,f:Apidae,g:Ceratina,s:Ceratina calcarata
AATTTTATATATTATATTTGCTATATGATCAGGTATAATCGGAGCATCAATAAGATTAAT
TATTCGAATAGAATTAAGAACCCCAGGAAATTGAATTAATAATGATCAAATTTATAACTC
ATTAGTTACTGCTCATGCCTTTTTAATAATTTTTTTTATAGTTATACCATTTATAATTGG
GGGATTTGGAAATTGATTAATCCCTCTAATATTAGGATCACCAGATATATCTTTTCCTCG
TTTAAATAATATTAGTTTTTGATTATTACCCCCATCATTACTACTATTATTATTAAGAAA
TTTATTTTCAATAAGACCAGGAACAGGATGAACTGTTTATCCACCTTTATCATCATATTT
ATTTCACCCATCCCCATCAGTAGACTTAGCTATTTTTTTCCTTACATATATCAGGAATTTC
ATCAATTTTAGGAGCTATTAATTTTATAGTTACTATTATAATAATAAAAAATATTTCATT
AAATTATGACTCTATTCCATTATTTTCATGAGCAGTATTTATTACAGCAATTTTATTACT
TTTATCATTACCAGTATTAGCAGGAGCTATTACTATATTATTATTTGATCGAAATTTAAA
TACATCTTTTTTTGATCCTATAGGAGGTGGAGATCCAATTTTATATCAACATTTATTT
>JF271021;tax=d:Eukarya,p:Arthropoda,c:Insecta,o:Hymenoptera,f:Apidae,g:Ceratina,s:Ceratina calcarata
AATTTTATATATTATATTTGCTATATGATCAGGTATAATCGGAGCATCAATAAGATTAAT
TATTCGAATAGAATTAAGAACCCCGGGAAATTGAATTAATAATGATCAAATTTATAACTC
ATTAGTTACTGCTCATGCCTTTTTAATAATTTTTTTTATAGTTATACCATTTATAATTGG
GGGATTTGGAAATTGATTAATCCCTCTAATATTAGGATCACCAGATATATCTTTCCCTCG
TTTAAATAATATTAGTTTTTGATTATTACCCCCATCATTACTACTATTATTATTAAGAAA
TTTATTTTCAATAAGACCAGGAACAGGATGAACTGTTTATCCACCTTTATCATCATATTT
ATTTCACCCATCCCCATCAGTAGACTTAGCTATTTTTTTCCTTACATATATCAGGAATTTC
ATCAATTTTAGGAGCTATTAATTTTATAGTTACTATTATAATAATAAAAAATATTTCATT
AAATTATGACTCTATTCCATTATTTTCATGAGCAGTATTTATTACAGCAATTTTATTACT
TTTATCATTACCAGTATTAGCAGGGGCTATTACTATATTATTATTTGATCGAAATTTAAA
TACATCCTTTTTTGATCCTATAGGAGGTGGAGATCCAATTTTATATCAACATTTATTT
>JF271023;tax=d:Eukarya,p:Arthropoda,c:Insecta,o:Hymenoptera,f:Apidae,g:Ceratina,s:Ceratina calcarata
AATTTTATATATTATATTTGCTATATGATCAGGTATAATCGGAGCATCAATAAGATTAAT
TATTCGAATAGAATTAAGAACCCCAGGAAATTGAATTAATAATGATCAAATTTATAACTC
ATTAGTTACTGCTCATGCCTTTTTAATAATTTTTTTTATAGTTATACCATTTATAATTGG
GGGATTTGGAAATTGATTAATCCCTCTAATATTAGGATCACCAGATATATCTTTTCCTCG
TTTAAATAATATTAGTTTTTGATTATTACCCCCATCATTACTACTATTATTATTAAGAAA
TTTATTTTCAATAAGACCAGGAACAGGATGAACTGTTTATCCACCTTTATCATCATATTT
ATTTCACCCATCCCCATCAGTAGACTTAGCTATTTTTTTCCTTACATATATCAGGAATTTC
ATCAATTTTAGGAGCTATTAATTTTATAGTTACTATTATAATAATAAAAAATATTTCATT
AAATTATGACTCTATTCCATTATTTTCATGAGCAGTATTTATTACAGCAATTTTATTACT
TTTATCATTACCAGTATTAGCAGGAGCTATCACTATATTATTATTTGATCGAAATTTAAA
TACATCCTTTTTTGATCCTATAGGAGGTGGAGATCCAATTTTATATCAACATTTATTT

>JF271027;tax=d:Eukarya,p:Arthropoda,c:Insecta,o:Hymenoptera,f:Apidae,g:Ceratina,s:Ceratina calcarata
AATTTTATATATTATATTTGCTATATGATCAGGTATAATCGGAGCATCAATAAGATTAAT
TATTCGAATAGAATTAAGAACCCCAGGAAATTGAATTAATAATGATCAAATTTATAACTC
ATTAGTTACTGCTCATGCCTTTTTAATAATTTTTTTTATAGTTATACCATTTATAATTGG
GGGATTTGGAAATTGATTAATCCCTCTAATATTAGGATCACCAGATATATCTTTTCCTCG
TTTAAATAATATTAGTTTTTGATTATTACCCCCATCATTACTACTATTATTATTAAGAAA
TTTATTTTCAATAAGACCAGGAACAGGATGAACTGTTTATCCACCTTTATCATCATATTT
ATTTCACCCATCCCCATCAGTAGATTTAGCTATTTTTTCCTTACATATATCAGGAATTTC
ATCAATTTTAGGAGCTATTAATTTTATAGTTACTATTATAATAATAAAAAATATTTCATT
AAATTATGACTCTATTCCATTATTTTCATGAGCAGTATTTATTACAGCAATTTTATTACT
TTTATCATTACCAGTATTAGCAGGAGCTATTACTATATTATTATTTGATCGAAATTTAAA
TACATCCTTTTTTGATCCTATAGGAGGTGGAGATCCAATTTTATATCAACATTTATTT
>JF271028;tax=d:Eukarya,p:Arthropoda,c:Insecta,o:Hymenoptera,f:Apidae,g:Ceratina,s:Ceratina calcarata
AATTTTATATATTATATTTGCTATATGATCAGGTATAATCGGAGCATCAATAAGATTAAT
TATTCGAATAGAATTAAGAACCCCAGGAAATTGAATTAATAATGATCAAATTTATAACTC
ATTAGTTACTGCTCATGCCTTTTTAATAATTTTTTTTATAGTTATACCATTTATAATTGG
GGGATTTGGAAATTGATTAATCCCTCTAATATTAGGATCACCAGATATATCTTTTCCTCG
TTTAAATAATATTAGTTTTTGATTATTACCCCCATCATTACTACTATTATTATTAAGAAA
TTTATTTTCAATAAGACCAGGAACAGGATGAACTGTTTATCCACCTTTATCATCATATTT
ATTTCACCCATCCCCATCAGTAGACTTAGCTATTTTTTCCTTACATATATCAGGAATTTC
ATCAATTTTAGGAGCTATTAATTTTATAGTTACTATTATAATAATAAAAAATATTTCATT
AAATTATGACTCTATTCCATTATTTTCATGAGCAGTACTTATTACAGCAATTTTATTACT
TTTATCATTACCAGTATTAGCAGGAGCTATTACTATATTATTATTTGATCGAAATTTAAA
TACATCCTTTTTTGATCCTATAGGAGGTGGAGATCCAATTTTATATCAACATTTATT
>JF271029;tax=d:Eukarya,p:Arthropoda,c:Insecta,o:Hymenoptera,f:Apidae,g:Ceratina,s:Ceratina calcarata
AATTTTATATATTATATTTGCTATATGATCAGGTATAATCGGAGCATCAATAAGATTAAT
TATTCGAATAGAATTAAGAACCCCAGGAAATTGAATTAATAATGATCAAATTTATAACTC
ATTAGTTACTGCTCATGCCTTTTTAATAATTTTTTTTATAGTTATACCATTTATAATTGG
GGGATTTGGAAATTGATTAATCCCTCTAATATTAGGATCACCAGATATATCTTTTCCTCG
TTTAAATAATATTAGTTTTTGATTATTACCCCCATCATTACTACTATTATTATTAAGAAA
TTTATTTTCAATAAGACCAGGAACAGGATGAACTGTTTATCCACCTTTATCATCATATTT
ATTTCACCCATCCCCATCAGTAGACTTAGCTATTTTTTCCTTACATATATCAGGAATTTC
ATCAATTTTAGGAGCTATTAATTTTATAGTTACTATTATAATAATAAAAAATATTTCATT
AAATTATGACTCTATTCCATTATTTTCATGAGCAGTATTTATTACAGCAATTTTATTACT
TTTATCATTACCAGTATTAGCAGGAGCTATTACTATATTATTATTTGATCGAAATTTAAA
TACATCTTTTTTTGATCCTATAGGAGGTGGAGATCCAATTTTATATCAACATTTATT
>KJ085734;tax=d:Eukarya,p:Arthropoda,c:Insecta,o:Hymenoptera,f:Apidae,g:Ceratina,s:Ceratina calcarata
AATTTTATATATTATATTTGCTATATGATCAGGTATAATCGGAGCATCAATAAGATTAAT
TATTCGAATAGAATTAAGAACCCCGGGAAATTGAATTAATAATGATCAAATTTATAACTC
ATTAGTTACTGCTCATGCCTTTTTAATAATTTTTTTCATAGTTATACCATTTATAATTGG
AGGATTTGGAAATTGATTAATCCCTCTAATATTAGGATCACCAGATATATCTTTTCCTCG
TTTAAATAATATTAGTTTTTGATTATTACCCCCATCATTACTACTATTATTATTAAGAAA
TTTATTTTCAATAAGACCAGGAACAGGATGAACTGTTTATCCACCTTTATCATCATATTT
ATTTCACCCATCCCCATCAGTAGACTTAGCTATTTTTTCCTTACATATATCAGGAATTTC
ATCAATTTTAGGAGCTATTAATTTTATAGTTACTATTATAATAATAAAAAATATTTCATT
AAATTATGATTCTATTCCATTATTTTCATGAGCAGTATTTATTACAGCAATTTTATTACT
TTTATCATTACCAGTATTAGCAGGGGCTATTACTATATTATTATTTGATCGAAATTTAAA
TACATCCTTTTTTGATCCTATAGGAGGTGGAGA
>KJ087531;tax=d:Eukarya,p:Arthropoda,c:Insecta,o:Hymenoptera,f:Apidae,g:Ceratina,s:Ceratina calcarata
AATTTTATATATTATATTTGCTATATGATCAGGTATAATCGGAGCATCAATAAGATTAAT
TATTCGAATAGAATTAAGAACCCCAGGAAATTGAATTAATAATGATCAAATTTATAACTC

ATTAGTTACTGCTCATGCCTTTTTAATAATTTTTTTTATAGTTATACCATTTATAATTGG
GGGATTTGGAAATTGATTAATCCCTCTAATATTAGGATCACCAGATATATCTTTTCCTCG
TTTAAATAATATTAGTTTTTGATTATTACCCCCATCATTACTACTATTATTATTAAGAAA
TTTATTTTCAATAAGACCAGGAACAGGATGAACTGTTTATCCACCTTTATCATCATATTT
ATTTCACCCATCCCCATCAGTAGACTTAGCTATTTTTTCCTTACATATATCAGGAATTTC
ATCAATTTTAGGAGCTATTAATTTTATAGTTACTATTATAATAATAAAAAATATTTCATT
AAATTATGACTCTATTCCATTATTTTCATGAGCAGTATTTATTACAGCAATTTTATTACT
TTTATCATTACCAGTATTAGCAGGAGCTATTACTATATTATTATTTGATCGAAATTTAAA
TACATCCTTTTTTGATCCTATAGGAGGTGGAG
>KJ091743;tax=d:Eukarya,p:Arthropoda,c:Insecta,o:Hymenoptera,f:Apidae,g:Ceratina,s:Ceratina calcarata
AATTTTATATATTATATTTGCTATATGATCAGGTATAATCGGAGCATCAATAAGATTAAT
TATTCGAATAGAATTAAGAACCCCGGGAAATTGAATTAATAATGATCAAATTTATAACTC
ATTAGTTACTGCTCATGCCTTTTTAATAATTTTTTTTATAGTTATACCATTTATAATTGG
GGGATTTGGAAATTGATTAATCCCTCTAATATTAGGATCACCAGATATATCTTTTCCTCG
TTTAAATAATATTAGTTTTTGATTATTACCCCCATCATTACTACTATTATTATTAAGAAA
TTTATTTTCAATAAGACCAGGAACAGGATGAACTGTTTATCCACCTTTATCATCATATTT
ATTTCACCCATCCCCATCAGTAGACTTAGCTATTTTTTCCTTACATATATCAGGAATTTC
ATCAATTTTAGGAGCTATTAATTTTATAGTTACTATTATAATAATAAAAAATATTTCATT
AAATTATGACTCTATTCCATTATTTTCATGAGCAGTATTTATTACAGCAATTTTATTACT
TTTATCATTACCAGTATTAGCAGGGGCTATTACTATATTATTATTTGATCGAAATTTAAA
TACATCCTTTTTTGATCCTATAGGAGG
>KJ092622;tax=d:Eukarya,p:Arthropoda,c:Insecta,o:Hymenoptera,f:Apidae,g:Ceratina,s:Ceratina calcarata
AATTTTATATATTATATTTGCTATATGATCAGGTATAATCGGAGCATCAATAAGATTAAT
TATTCGAATAGAATTAAGAACCCCAGGAAATTGAATTAATAATGATCAAATTTATAACTC
ATTAGTTACTGCTCATGCCTTTTTAATAATTTTTTTTATAGTTATACCATTTATAATTGG
GGGATTTGGAAATTGATTAATCCCTCTAATATTAGGATCACCAGATATATCTTTTCCTCG
TTTAAATAATATTAGTTTTTGATTATTACCCCCATCATTACTACTATTATTATTAAGAAA
TTTATTTTCAATAAGACCAGGAACAGGATGAACTGTTTATCCACCTTTATCATCATATTT
ATTTCACCCATCCCCATCAGTAGACTTAGCTATTTTTTCCTTACATATATCAGGAATTTC
ATCAATTTTAGGAGCTATTAATTTTATAGTTACTATTATAATAATAAAAAATATTTCATT
AAATTATGACTCTATTCCATTATTTTCATGAGCAGTATTTATTACAGCAATTTTATTACT
TTTATCATTACCAGTATTAGCAGGAGCTATTACTATATTATTATTTGATCGAAATTTAAA
TACATCCTTTTT
>KJ092990;tax=d:Eukarya,p:Arthropoda,c:Insecta,o:Hymenoptera,f:Apidae,g:Ceratina,s:Ceratina calcarata
AATTTTATATATTATATTTGCTATATGATCAGGTATAATCGGAGCATCAATAAGATTAAT
TATTCGAATAGAATTAAGAACCCCGGGAAATTGAATTAATAATGATCAAATTTATAACTC
ATTAGTTACTGCTCATGCCTTTTTAATAATTTTTTTTATAGTTATACCATTTATAATTGG
GGGATTTGGAAATTGATTAATCCCTCTAATATTAGGATCACCAGATATATCTTTTCCTCG
TTTAAATAATATTAGTTTTTGATTATTACCCCCATCATTACTACTATTATTATTAAGAAA
TTTATTTTCAATAAGACCAGGAACAGGATGAACTGTTTATCCACCTTTATCATCATATTT
ATTTCACCCATCCCCATCAGTAGACTTAGCTATTTTTTCCTTACATATATCAGGAATTTC
ATCAATTTTAGGAGCTATTAATTTTATAGTTACTATTATAATAATAAAAAATATTTCATT
AAATTATGACTCTATTCCATTATTTTCATGAGCAGTATTTATTACAGCAATTTTATTACT
TTTATCATTACCAGTATTAGCAGGGGCTATTACTATATTATTATTTGATCGAAATTTAAA
TAC
>KP747136;tax=d:Eukarya,p:Arthropoda,c:Insecta,o:Hymenoptera,f:Apidae,g:Ceratina,s:Ceratina calcarata
CCCCAGGAAATTGAATTAATAATGATCAAATTTATAACTCATTAGTTACTGCTCATGCCT
TTTTAATAATTTTTTTTATAGTTATACCATTTATAATTGGAGGATTTGGAAATTGATTAA
TCCCTCTAATATTAGGATCACCAGATATATCTTTTCCTCGTTTAAATAATATTAGTTTTT
GATTATTACCCCCATCATTACTACTATTATTATTAAGAAATTTATTTTCAATAAGACCGG
GAACAGGATGAACTGTTTATCCACCTTTATCATCATATTTATTTCACCCATCTCCATCAG

TAGACTTAGCTATTTTTTCCTTACATATGTCAGGAATTTCATCAATTTTAGGAGCTATTA
ATTTTATAGTTACTATTATAATAATAAAAAATATTTCATTAAATTATGACTCTATTCCAT
TATTTTCATGAGCAGTATTTATTACAGCAATTTTATTACTTTTATCATTACCAGTATTAG
CAGGAGCTATTACTATATTATTATTTGATCGAAATTTAAATACATCCTTTTTTGATCCTA
TAGGAGGTGGAGATCCAATTTTATATCAACATTTA
>KP747139;tax=d:Eukarya,p:Arthropoda,c:Insecta,o:Hymenoptera,f:Apidae,g:Ceratina,s:Ceratina calcarata
CCCCGGGAAATTGAATTAATAATGATCAAATTTATAACTCATTAGTTACTGCTCATGCCT
TTTTAATAATTTTTTTTATAGTTATACCATTTATAATTGGGGGATTTGGAAATTGATTAA
TCCCTCTAATATTAGGATCACCAGATATATCTTTTCCTCGTTTAAATAATATTAGTTTTT
GATTATTACCCCCATCATTACTACTATTATTATTAAGAAATTTATTTTCAATAAGACCAG
GAACAGGATGAACTGTTTATCCACCTTTATCATCATATTTATTTCACCCATCCCCATCAG
TAGACTTAGCTATTTTTTCCTTACATATATCAGGAATTTCATCAATTTTAGGAGCTATTA
ATTTTATAGTTACTATTATAATAATAAAAAATATTTCATTAAATTATGACTCTATTCCAT
TATTTTCATGAGCAGTATTTATTACAGCAATTTTATTACTTTTATCATTACCAGTATTAG
CAGGGGCTATTACTATATTATTATTTGATCGAAATTTAAATACATCCTTTTTTGATCCTA
TAGGAGGTGGAGATCCAATTTTATATCAACATTTA
>KP747143;tax=d:Eukarya,p:Arthropoda,c:Insecta,o:Hymenoptera,f:Apidae,g:Ceratina,s:Ceratina calcarata
CCCCAGGAAATTGAATCAATAATGATCAAATTTATAACTCATTAGTTACTGCTCATGCCT
TTTTAATAATTTTTTTTATAGTTATACCATTTATAATTGGAGGATTTGGAAATTGATTAA
TCCCTCTAATATTAGGATCACCAGATATATCTTTTCCTCGTTTAAATAATATTAGTTTTT
GATTATTACCCCCATCATTACTACTATTATTATTAAGAAATTTATTTTCAATAAGACCAG
GAACAGGATGAACTGTTTATCCACCTTTATCATCATATTTATTTCACCCATCTCCATCAG
TAGACTTAGCTATTTTTTCCTTACATATGTCAGGAATTTCATCAATTTTAGGAGCTATTA
ATTTTATAGTTACTATTATAATAATAAAAAATATTTCATTAAATTATGACTCTATTCCAT
TATTTTCATGAGCAGTATTTATTACAGCAATTTTATTACTTTTATCATTACCAGTATTAG
CAGGAGCTATTACTATATTATTATTTGATCGAAATTTAAATACATCCTTTTTTGATCCTA
TAGGAGGTGGAGATCCAATTTTATATCAACATTTA
>KP747148;tax=d:Eukarya,p:Arthropoda,c:Insecta,o:Hymenoptera,f:Apidae,g:Ceratina,s:Ceratina calcarata
CCCCAGGAAATTGAATTAATAATGATCAAATTTATAACTCATTAGTTACTGCTCATGCCT
TTTTAATAATTTTTTTTATAGTTATACCATTTATAATTGGAGGATTTGGAAATTGATTAA
TCCCTCTAATATTAGGATCACCAGATATATCTTTTCCTCGTTTAAATAATATTAGTTTTT
GATTATTACCCCCATCATTACTACTATTATTATTAAGAAATTTATTTTCAATAAGACCAG
GAACAGGATGAACTGTTTATCCACCTTTATCATCATATTTATTTCACCCATCTCCATCAG
TAGACTTAGCTATTTTTTCCTTACATATGTCAGGAATTTCATCAATTTTAGGAGCTATTA
ATTTTATAGTTACTATTATAATAATAAAAAATATTTCATTAAATTATGACTCTATTCCAT
TATTTTCATGAGCAGTATTTATTACAGCAATTTTATTACTTTTATCATTACCAGTATTAG
CAGGAGCTATTACTATATTATTATTTGATCGAAATTTAAATACATCCTTTTTTGATCCTA
TAGGAGGTGGAGATCCAATTTTATATCAACATTTA
>KP747158;tax=d:Eukarya,p:Arthropoda,c:Insecta,o:Hymenoptera,f:Apidae,g:Ceratina,s:Ceratina calcarata
CCCCGGGAAATTGAATTAATAATGATCAAATTTATAACTCATTAGTTACTGCTCATGCCT
TTTTAATAATTTTTTTTATAGTTATACCATTTATAATTGGGGGATTTGGAAATTGATTAA
TCCCTCTAATATTAGGATCACCAGATATATCTTTTCCTCGTTTAAATAATATTAGTTTTT
GATTATTACCCCCATCATTACTACTATTATTATTAAGAAATTTATTTTCAATAAGACCAG
GAACAGGATGAACTGTTTATCCACCTTTATCATCATATTTATTTCACCCATCCCCATCAG
TAGACTTAGCTATTTTTTCTTTACATATATCAGGAATTTCATCAATTTTAGGAGCTATTA
ATTTTATAGTTACTATTATAATAATAAAAAATATTTCATTAAATTATGACTCTATTCCAT
TATTTTCATGAGCAGTATTTATTACAGCAATTTTATTACTTTTATCATTACCAGTATTAG
CAGGAGCTATTACTATATTATTATTTGATCGAAATTTAAATACATCCTTTTTTGATCCTA
TAGGAGGTGGAGATCCAATTTTATATCAACATTTA
>KP747160;tax=d:Eukarya,p:Arthropoda,c:Insecta,o:Hymenoptera,f:Apidae,g:Ceratina,s:Ceratina calcarata
CCCCAGGAAATTGAATTAAAAATGATCAAATTTATAACTCATTAGTTACTGCTCATGCCT

TTTTAATAATTTTTTTTATAGTTATACCATTTATAATTGGGGGATTTGGAAATTGATTAA
TCCCTCTAATATTAGGATCACCAGATATATCTTTTCCTCGTTTAAATAATATTAGTTTTT
GATTATTACCCCCATCATTACTACTATTATTATTAAGAAATTTATTTTCAATAAGACCAG
GAACAGGATGAACTGTTTATCCACCTTTATCATCATATTTATTTCACCCATCCCCATCAG
TAGACTTAGCTATTTTTTCCTTACATATATCAGGAATTTCATCAATTTTAGGAGCTATTA
ATTTTATAGTTACTATTATAATAATAAAAAATATTTCATTAAATTATGACTCTATTCCAT
TATTTTCATGAGCAGTATTTATTACAGCAATTTTATTACTTTTATCATTACCAGTATTAG
CAGGAGCTATTACTATATTATTATTTGATCGAAATTTAAATACATCCTTTTTTGATCCTA
TAGGAGGTGGAGATCCAATTTTATATCAACATTTA
>KP747166;tax=d:Eukarya,p:Arthropoda,c:Insecta,o:Hymenoptera,f:Apidae,g:Ceratina,s:Ceratina calcarata
CCCCGGGAAATTGAATTAATAATGATCAAATTTATAACTCATTAGTTACTGCTCATGCCT
TTTTAATAATTTTTTTTATAGTTATACCATTTATAATTGGGGGATTTGGAAATTGATTAA
TCCCTCTAATATTAGGATCACCAGATATATCTTTTCCTCGTTTAAATAATATTAGTTTTT
GATTATTACCCCCATCATTACTACTATTATTATTAAGAAATTTATTTTCAATAAGACCAG
GAACAGGATGAACTGTTTATCCACCTTTATCATCATATTTATTTCACCCATCTCCATCAG
TAGACTTAGCTATTTTTTCCTTACATATATCAGGAATTTCATCAATTTTAGGAGCTATTA
ATTTTATAGTTACTATTATAATAATAAAAAATATTTCATTAAATTATGACTCTATTCCAT
TATTTTCATGAGCAGTATTTATTACAGCAATTTTATTACTTTTATCATTACCAGTATTAG
CAGGAGCTATTACTATATTATTATTTGATCGAAATTTAAATACATCCTTTTTTGATCCTA
TAGGAGGTGGAGATCCAATTTTATATCAACATTTA
>KT123216;tax=d:Eukarya,p:Arthropoda,c:Insecta,o:Hymenoptera,f:Apidae,g:Ceratina,s:Ceratina calcarata
GGTATAATCGGAGCATCAATAAGATTAATTATTCGAATAGAATTAAGAACCCCAGGAAAT
TGAATTAATAATGATCAAATTTATAACTCATTAGTTACTGCTCATGCCTTTTTAATAATT
TTTTTTATAGTTATACCATTTATAATTGGAGGATTTGGAAATTGATTAATTCCTCTAATA
TTAGGATCACCAGATATATCTTTTCCTCGTTTAAATAATATTAGTTTTTGATTATTACCC
CCATCATTACTACTATTATTATTAAGAAATTTATTTTCAATAAGACCAGGAACAGGATGA
ACTGTTTATCCACCTTTATCATCATATTTATTTCACCCATCTCCATCAGTAGACTTAGCT
ATTTTTTCCTTACATATATCAGGAATTTCATCAATTTTAGGAGCTATTAATTTTATAGTT
ACTATTATAATAATAAAAAATATTTCATTAAATTATGACTCTATTCCATTATTTTCATGA
GCTGTATTTATTACAGCAATTTTATTACTTTTATCATTACCAGTATTAGCAGGAGCTATT
ACTATATTATTATTTGATCGAAATTTAAATACATCCTTTTTTGATCCTATAGGAGGTGGA
GATCCAATTTTATATCAACATTTATTT
>MG438819;tax=d:Eukarya,p:Arthropoda,c:Insecta,o:Hymenoptera,f:Apidae,g:Ceratina,s:Ceratina calcarata
TATATATTATATTTGCTATATGATCAGGTATAATCGGAGCATCAATAAGATTAATTATTC
GAATAGAATTAAGAACCCCAGGAAATTGAATTAATAATGATCAAATTTATAACTCATTAG
TTACTGCTCATGCCTTTTTAATAATTTTTTTTATAGTTATACCATTTATAATTGGGGGAT
TTGGAAATTGATTAATCCCTCTAATATTAGGATCACCAGATATATCTTTTCCTCGTTTAA
ATAATATTAGTTTTTGATTATTACCCCCATCATTACTACTATTATTATTAAGAAATTTAT
TTTCAATAAGACCAGGAACAGGATGAACTGTTTATCCACCTTTATCATCATATTTATTTC
ACCCATCCCCATCAGTAGACTTAGCTATTTTTTCCTTACATATATCAGGAATTTCATCAA
TTTTAGGAGCTATTAATTTTATAGTTACTATTATGATAATAAAAAATATTTCATTAAATT
ATGACTCTATTCCATTATTTTCATGAGCAGTATTTATTACAGCAATTTTATTACTTTTAT
CATTACCAGTATTAGCAGGAGCTATTACTATATTATTATTTGATCGAAATTTAAATACAT
CTTTTTTTGATCCTATAGGAGGTGGAGATCCAATTTTATATCAACATTTA
>MG439071;tax=d:Eukarya,p:Arthropoda,c:Insecta,o:Hymenoptera,f:Apidae,g:Ceratina,s:Ceratina calcarata
TATATATTATATTTGCTATATGATCAGGTATAATCGGAGCATCAATAAGATTAATTATTC
GAATAGAATTAAGAACCCCAGGAAATTGAATTAATAATGATCAAATTTATAACTCATTAG
TTACTGCTCATGCCTTTTTAATAATTTTTTTTATAGTTATACCATTTATAATTGGGGGAT
TTGGAAATTGATTAATCCCTCTAATATTAGGATCACCAGATATATCTTTTCCTCGTTTAA
ATAATATTAGTTTTTGATTATTACCCCCATCATTACTACTATTATTATTAAGAAATTTAT
TTTCAATAAGACCAGGAACAGGATGAACTGTTTATCCACCTTTATCATCATATTTATTTC

ACCCATCCCCATCAGTAGACTTAGCTATTTTTTCCTTACATATATCAGGAATTTCATCAA
TTTTAGGAGCTATTAATTTTATAGTTACTATTATAATAATAAAAAATATTTCATTAAATT
ATGACTCTATTCCATTATTTTCATGAGCAGTATTTATTACAGCAATTTTATTACTTTTAT
CATTACCAGTATTAGCAGGAGCTATTACTATATTATTATTTGATCGAAATTTAAATACAT
CCTTTTTTGATCCTATAGGAGGTGGAGATCCAATTTTATATCAACATTTA
>MG447610;tax=d:Eukarya,p:Arthropoda,c:Insecta,o:Hymenoptera,f:Apidae,g:Ceratina,s:Ceratina calcarata
TTTTTGCTATATGATCAGGTATAATCGGAGCATCAATAAGATTAATTATTCGAATAGAAT
TAAGAACCCCAGGAAATTGAATTAATAATGATCAAATTTATAACTCATTAGTTACTGCTC
ATGCCTTTTTAATAATTTTTTTTATAGTTATACCATTTATAATTGGGGGATTTGGAAATT
GATTAATCCCTCTAATATTAGGATCACCAGATATATCTTTTCCTCGTTTAAATAATATTA
GTTTTTGATTATTACCCCCATCATTACTACTATTATTATTAAGAAATTTATTCTCAATAA
GACCAGGAACAGGATGAACTGTTTATCCACCTTTATCATCATATTTATTTCACCCATCCC
CATCAGTAGACTTAGCTATTTTTTCCTTACATATATCAGGAATTTCATCAATTTTAGGAG
CTATTAATTTTATAGTTACTATTATAATAATAAAAAATATTTCATTAAATTATGACTCTA
TTCCATTATTTTCATGAGCAGTATTTATTACAGCAATTTTATTACTTTTATCATTACCAG
TATTAGCAGGAGCTATTACTATATTATTATTTGATCGAAATTTAAATACATCCTTTTTTG
ATCCTATAGGAGGTGGAGATCCAATTTTATATCAACATTTA
>MK919598;tax=d:Eukarya,p:Arthropoda,c:Insecta,o:Hymenoptera,f:Apidae,g:Holcopasites,s:Holcopasites calliopsidis
TCGGATCTCCTGATATAAGATTCCCACGAATAAATAATATTAGATTTTGATTATTACCCC
CTTCATTATTTTTTTTATTATTTAGAAGATTATTTAATAATCCAGCTGGAACAGGATGAA
CAGTATACCCCCCCCTTTCTCTATCTATATTTCATTCAGGGTCTTCTGNAGATTTAGTAA
TTTTTTCATTACATTTATCAGGAATATCTTCAATTATAGGAGCTATAAATTTTATAGNAA
CAATTATATTAATAAAAAATTTTTCATTAAATTATGACCAAATTAATTTATTTTCATGAT
CAACTTTTATTACTGCAATTCTTTTATTATTATCATTACCAGTTTTAGCAGGAGCAATTA
CTATATTATTATTTGATCGAAATTTTAATACATCATTCTTTGATCCTATAGGAGGAGGAG
ACCCTATTTTATATCAACATTTATTT
>FJ582235;tax=d:Eukarya,p:Arthropoda,c:Insecta,o:Hymenoptera,f:Megachilidae,g:Hoplitis,s:Hoplitis pilosifrons
AATTATTTATATAATTTTTAGAATATGATCAGGAATAATTGGTTCAGCATTAAGTATTAT
TATTCGAATAGAGTTAAGAATTCCTGGATCTTGAATTAATAATGATCAAATTTATAATTC
AATTGTTACTGCTCATGCTTTTTTAATAATTTTTTTTTTAGTAATGCCTTTTATAATTGG
GGGATTTGGAAATTGATTAATTCCATTAATATTAGGAGTTCCAGATATAGCATTTCCTCG
AATAAATAATATTAGATTTTGATTACTTCCTCCTTCATTATTATTATTATTATTAAGAAA
TTTTTTTAATCCAAGGCCAGGAACTGGATGAACAGTTTATCCTCCTTTATCTTCTTATTT
ATATCATTCTTCACCATCAGTTGATATAGCTATTTTTTCATTACATATTTCAGGTTTATC
TTCAATTATAGGATCATTAAATTTTATTGTAACTATTATTATAATAAAAAATATTTCATT
AAGTTATAATCAACTTCCATTATTTCCTTGGTCTGTATTTATTACAACTATTTTATTACT
TTTATCTTTACCTGTATTAGCTGGAGCAATTACTATACTTTTATTTGATCGAAATTTTAA
TACTTCTTTTTTTGATCCAATAGGAGGAGGAGATCCAATTTTATTCCAACATTTATTT
>MG348183;tax=d:Eukarya,p:Arthropoda,c:Insecta,o:Hymenoptera,f:Megachilidae,g:Hoplitis,s:Hoplitis pilosifrons
TTTATATAATTTTTAGAATATGATCAGGAATAATTGGTTCAGCATTAAGTATTATTATTC
GAATAGAATTAAGAATTCCTGGATCTTGAATTAATAATGATCAAATTTATAATTCAATTG
TTACTGCTCATGCTTTTTTAATAATTTTTTTTTTAGTAATGCCTTTTATAATTGGGGGAT
TTGGAAATTGATTAATTCCATTAATATTAGGAGTTCCAGATATAGCATTTCCTCGAATAA
ATAATATTAGATTTTGATTACTTCCTCCTTCATTATTATTATTATTATTAAGAAATTTTT
TTAATCCAAGGCCAGGAACTGGATGAACAGTTTATCCTCCTTTATCTTCTTATTTATATC
ATTCTTCACCATCAGTTGATATAGCTATTTTTTCATTACATATTTCAGGTTTATCTTCAA
TTATAGGATCATTAAATTTTATTGTAACTATTATTATAATAAAAAATATTTCATTAAATT
ATAATCAACTTCCATTATTTCCTTGGTCTGTATTTATTACAACTATTTTATTACTTTTAT
CTTTACCTGTATTAGCTGGAGCAATTACTATACTTTTATTTGATCGAAATTTTAATACTT
CTTTTTTTGATCCAATAGGAGGAGGAGACCCAATTTTATTCCAACATTTA

>MG353231;tax=d:Eukarya,p:Arthropoda,c:Insecta,o:Hymenoptera,f:Megachilidae,g:Hoplitis,s:Hoplitis pilosifrons
TTTATATAATTTTTAGAATATGATCAGGAATAATTGGTTCAGCATTAAGTATTATTATTC
GAATAGAGTTAAGAATTCCTGGATCTTGAATTAATAATGATCAAATTTATAATTCAATTG
TTACTGCTCATGCTTTTTTAATAATTTTTTTTTTAGTAATGCCTTTTATAATTGGGGGAT
TTGGAAATTGATTAATTCCATTAATATTAGGAGTTCCAGATATAGCATTTCCTCGAATAA
ATAATATTAGATTTTGATTACTTCCTCCTTCATTATTATTATTATTATTAAGAAATTTTT
TTAATCCAAGGCCAGGAACTGGATGAACAGTTTATCCTCCTTTATCTTCTTATTTATATC
ATTCTTCACCATCAGTTGATATAGCTATTTTTTCATTACATATTTCAGGTTTATCTTCAA
TTATAGGATCATTAAATTTTATTGTAACTATTATTATAATAAAAAATATTTCATTAAATT
ATAATCAACTTCCATTATTTCCTTGGTCTGTATTTATTACAACTATTTTATTACTTTTAT
CTTTACCTGTATTAGCTGGAGCAATTACTATACTTTTATTTGATCGAAATTTTAATACTT
CTTTTTTTGATCCAATAGGAGGAGGAGATCCAATTTTATTCCAACATTTA
>FJ582323;tax=d:Eukarya,p:Arthropoda,c:Insecta,o:Hymenoptera,f:Megachilidae,g:Megachile,s:Megachile pugnata
GCATTATGATCTGGAATAATTGGATCAGCAATAAGAATAATTATTCGAATAGAATTAAGA
ACTCCAGGATCATGAATTAAAAATGATCAAATTTATAATTCAATTGTTACTGCTCATGCA
TTTTTAATAATTTTTTTTTTAGTTATACCATTTATAATTGGAGGATTTGGAAATTGATTA
ATACCTTTAATAATTGGAGCACCAGATATAGCATTCCCACGAATAAATAATGTAAGATTT
TGATTATTACCTCCCTCATTAATCTTATTATTATCAAGAAATTTAATGAATCCAAGACCA
GGAACTGGTTGAACTGTATACCCCCCTTTATCATTAAATATATTTCATCCATCACCATCT
GTAGATTTAGCAATTTTTTCATTACACCTTTCTGGAATTTCATCTATTATTGGATCATTA
AATTTTATTGTAACAATCCTTTTAATAAAAAATAATTCATTAAATTATAGACAAATATCT
TTATTTCCATGATCAATTTTTATTACAACAATTCTATTATTATTATCATTACCAGTTTTA
GCAGGAGCAATTACTATATTATTATTTGATCGAAATTTAAATACATCTTTTTTTGATCCA
ATAGGAGGTGGTGACCCTATTTTATATCAACATTTATTT
>KM559099;tax=d:Eukarya,p:Arthropoda,c:Insecta,o:Hymenoptera,f:Megachilidae,g:Megachile,s:Megachile pugnata
AATTTTATATATAATTTTTGCATTATGATCTGGAATAATTGGATCAGCAATAAGAATAAT
TATTCGAATAGAATTAAGAACTCCAGGATCATGAATTAAAAATGATCAAATTTATAATTC
AATTGTTACTGCTCATGCATTTTTAATAATTTTTTTTTTAGTTATACCATTTATAATTGG
AGGATTTGGAAATTGATTAATACCTTTAATAATTGGAGCACCAGATATAGCATTCCCACG
AATAAATAATGTAAGATTTTGATTATTACCTCCCTCATTAATCTTATTATTATCAAGAAA
TTTAATGAATCCAAGACCAGGAACTGGTTGAACTGTATACCCCCCTTTATCATTAAATAT
ATTTCATCCATCACCATCTGTAGATTTAGCAATTTTTTCATTACACCTTTCTGGAATTTC
ATCTATTATTGGATCATTAAATTTTATTGTAACAATCCTTTTAATAAAAAATAATTCATT
AAATTATAGACAAATATCTTTATTTCCATGATCAATTTTTATTACAACAATTCTATTATT
ATTATCATTACCAGTTTTAGCAGGAGCAATTACTATATTATTATTTGATCGAAATTTAAA
TACATCTTTTTTTGATCCAATAGGAGGTGGTGACCCTATTTTATATCAACATTTATTT
>KT707417;tax=d:Eukarya,p:Arthropoda,c:Insecta,o:Hymenoptera,f:Megachilidae,g:Megachile,s:Megachile pugnata
AATTTTATATATAATTTTTGCATTATGATCTGGAATAATTGGATCAGCAATAAGAATAAT
TATTCGAATAGAATTAAGAACTCCAGGATCATGAATTAAAAATGATCAAATTTATAATTC
AATTGTTACTGCTCATGCATTTTTAATAATTTTTTTTTTAGTTATACCATTTATAATTGG
AGGATTTGGAAATTGATTAATACCTTTAATAATTGGAGCACCAGATATAGCATTCCCACG
AATAAATAATGTAAGATTTTGATTATTACCTCCCTCATTAATCTTATTATTATCAAGAAA
TTTAATGAATCCAAGACCAGGAACTGGTTGAACTGTATACCCCCCTTTATCATTAAATAT
ATTTCATCCATCACCATCTGTAGATTTAGCAATTTTTTCATTACACCTTTCTGGAATTTC
ATCTATTATTGGATCATTAAATTTTATTGTAACAATCCTTTTAATAAAAAATAATTCATT
AAATTATAGACAAATATCTTTATTTCCATGATCAATTTTTATTACAACAATTCTATTATT
ATTATCATTACCAGTTTTAGCAGGAGCAATTACTATATTATTATTTGATCGAAATTTAAA
TACATCTTTTTTTGATCCAATAGGAGG
>MG251038;tax=d:Eukarya,p:Arthropoda,c:Insecta,o:Hymenoptera,f:Apidae,g:Svastra,s:Svastra obliqua
AGTATTGTATATGCTGTTAGCTATATGATCAGGAATAGTTGGTATATCTATAAGATTAAT
TATTCGAATAGAGCTAAGATGTCCGGGCAAATGAATCAATAATGATCAGTTGTATAATTC

ATTTGTTACTGCTCATGCTTTTTTAATAATTTTTTTTATAGTTATACCCTTTTTAATTGG
GGGGTTTGGTAACTGATTAATACCAATAATATTAGGGTCCCCAGATATAGCTTTCCCTCG
TATAAATAATATTAGTTTTTGATTATTGCCTTGCTCTCTATTGTTTCTTTTAATTAGAAA
TTTATTTACTATAAGTCCAGGGACAGGATGAACTGTTTACCCTCCATTATCTTCATTTAT
ATATCATTCTTCCCCCTCAGTAGATATAATGATTTTTTCTTTGCATTTATCAGGGCTATC
TTCAATTTTAGGAGCAATAAATTTTATAGTAACAATTATAATAATAAAAAATTTATCAAT
AAATTACGATCAAATTAATTTATTTTCATGGTCATTGTTTATTACAGCTATTTTGTTATT
ATTATCTTTACCTGTATTAGCTGGGGCCATTACTATATTATTGTTTGATCGTAATTTAAA
TACTTCCTTTTTTGATCCGGTAGGGGGAGGTGATCCAATTTTATATCAACATTTATTT
>HM405324;tax=d:Eukarya,p:Arthropoda,c:Insecta,o:Hymenoptera,f:Halictidae,g:Lasioglossum,s:Lasioglossum hitchensi
AATACTTTATTTTATTTTTGCTATATGGGCTGGAATAATTGGAGCTTCATTAAGAATAAT
TATTCGAATAGAATTAAGTGCTCCTGGAAAATGAATTAATAATGATCAAATTTATAATAC
TATTATTACATCTCATGCATTTGTAATAATTTTTTTTATAGTTATACCATTTATAATTGG
TGGATTTGGTAATTGATTAGTTCCTTTAATAATTGGAGCCCCTGATATAGCATTCCCCCG
AATAAATAATATAAGATTTTGATTACTTATCCCATCAATATTTATATTATTAATAAGAAG
AATTATATCATCTGGTTCAGGAACTGGATGAACTGTATACCCCCCCTTATCATCAATTAT
ATACCATTCATCAATTTCAGTAGATTACACTATCTTTTCATTACATATTGCAGGAATTTC
ATCTATTATAGGAGCTATTAACTTTATTGTATCTATTTTACTTATAAAAAATATTTCAAT
TAATTATGATCAAATTCCTTTATTCCCATGATCAGTAAAAATTACTGCTATTTTATTATT
ATTATCTCTACCAGTTTTAGCAGGAGCTATTACTATACTTTTAACAGATCGAAATTTAAA
TACTTCATTTTTTG
>KT123226;tax=d:Eukarya,p:Arthropoda,c:Insecta,o:Hymenoptera,f:Halictidae,g:Lasioglossum,s:Lasioglossum hitchensi
GAATTAAGTGCTCCTGGAAAATGAATTAATAATGATCAAATTTATAATACTATTATTACA
TCTCATGCATTTGTAATAATTTTTTTTATAGTTATACCATTTATAATTGGTGGATTTGGT
AATTGATTAGTTCCTTTAATAATTGGAGCCCCTGATATAGCATTCCCCCGAATAAATAAT
ATAAGATTTTGATTACTTATCCCATCAATATTTATATTATTAATAAGAAGAATTATATCA
TCTGGTTCAGGAACTGGATGAACTGTATACCCCCCCTTATCATCAATTATATACCATTCA
TCAATTTCAGTAGATTACACTATCTTTTCATTACATATTGCAGGAATTTCATCTATTATA
GGAGCTATTAACTTTATTGTATCTATTTTACTTATAAAAAATATTTCAATTAATTATGAT
CAAATTCCTTTATTCCCATGATCAGTAAAAATTACTGCTATTTTATTATTATTATCTCTA
CCAGTTTTAGCAGGAGCTATTACTATACTTTTAACAGATCGAAATTTAAATACTTCATTT
TTTGACCCATCAGGAGGAGGAGACCCTATTCTTTATCAACATTTATTT
>HM423169;tax=d:Eukarya,p:Arthropoda,c:Insecta,o:Hymenoptera,f:Megachilidae,g:Heriades,s:Heriades variolosus
ATATATAATTTTTGCAATATGAGCTGGAATAATTGGATCTGCTATAAGAATTATTATTCG
AATAGAATTAAGTACATCAGGATCATGATTAAAAAATGATCAAATTTATAATTCTATTGT
AACTGCTCATGCATTTTTAATAATTTTTTTTTTAGTTATACCTTTTATAATTGGTGGATT
TGGAAATTGATTAATTCCTTTATTATTAGGTATTCCTGATATAGCTTTTCCTCGAATAAA
TAATATTAGATTTTGATTATTACCCCCATCTTTAATATTATTACTTTTAAGAAATTTATT
TAATCCAAGACCTGGAACAGGATGAACAGTTTATCCTCCTTTATCAACTTATATTTATCA
TTCATCTCCTTCTGTAGATTTAGCAATTTTTTCTTTACATATTTCAGGTATTTCATCAAT
TATAGGATCATTAAATTTTATTGTAACAATTATAATAATAAAAAATTTATCAATTAATTA
TATACAAATACCATTATTTGCATGATCAGTTTTTATTACTACAATTTTACTTTTATTATC
TTTACCTGTATTAGCTGGAGCAATTACTATACTTTTATTTGATCGAAATTTAAATTCATC
ATTTTTTGATCCAATAGGTGGAGGTGATCCAATTTTATATCAACATTTATTT
>HM423172;tax=d:Eukarya,p:Arthropoda,c:Insecta,o:Hymenoptera,f:Megachilidae,g:Heriades,s:Heriades variolosus
ATATATAATTTTTGCAATATGAGCTGGAATAATTGGATCTGCTATAAGAATTATTATTCG
AATAGAATTAAGTACATCAGGATCATGATTAAAAAATGATCAAATTTATAATTCTATTGT
AACTGCTCATGCATTTTTAATAATTTTTTTTTTAGTTATACCTTTTATAATTGGTGGATT
TGGAAATTGATTAATTCCTTTATTATTAGGTATTCCTGATATAGCCTTTCCTCGAATAAA

TAATATTAGATTTTGATTATTACCTCCATCTTTAATATTATTACTTTTAAGAAATTTATT
TAATCCTAGACCTGGAACAGGATGAACAGTTTATCCTCCTTTATCAACTTATATTTATCA
TTCATCTCCTTCTGTAGATTTAGCAATTTTTTCTTTACATATTTCAGGTATTTCATCAAT
TATAGGATCATTAAATTTTATTGTAACAATTATAATAATAAAAAATTTATCAATTAATTA
TATACAAATACCATTATTTGCATGATCAGTTTTTATTACTACAATTTTACTTTTATTATC
TTTACCTGTATTAGCTGGAGCAATTACTATACTTTTATTTGATCGAAATTTAAATTCATC
ATTTTTTGATCCAATAGGTGGAGGTGATCCAATTTTATATCAACATTTATTT
>KM563609;tax=d:Eukarya,p:Arthropoda,c:Insecta,o:Hymenoptera,f:Megachilidae,g:Heriades,s:Heriades variolosus
ATATATAATTTTTGCAATATGAGCTGGAATAATTGGATCTGCTATAAGAATTATTATTCG
AATAGAATTAAGTACATCAGGATCATGATTAAAAAATGATCAAATTTATAATTCTATTGT
AACTGCTCATGCATTTTTAATAATTTTTTTTTTAGTTATACCTTTTATAATTGGTGGATT
TGGAAATTGATTAATTCCTTTATTATTAGGTATTCCTGATATAGCTTTTCCTCGAATAAA
TAATATTAGATTTTGATTATTACCCCCATCTTTAATATTATTACTTTTAAGAAATTTATT
TAATCCAAGACCTGGAACAGGATGAACAGTTTATCCTCCTTTATCAACTTATATTTATCA
TTCATCTCCTTCTGTAGATTTAGCAATTTTTTCTTTACATATTTCAGGTATTTCATCAAT
TATAGGATCATTAAATTTTATTGTAACAATTATAATAATAAAAAATTTATCAATTAATTA
TATACAAATACCATTATTTGCATGATCAGTTTTTATTACTACAATTTTACTTTTATTATC
TTTACCTGTATTAGCTGGAGCAATTACTATACTTTTATTTGATCGAAATTTAAATTCATC
ATTTTTTG
>KX580319;tax=d:Eukarya,p:Arthropoda,c:Insecta,o:Hymenoptera,f:Megachilidae,g:Coelioxys,s:Coelioxys
octodentata
TATAATATATATAATTTTTGCAATATGATCAGGAATAATTGGATCTTCTTTAAGAATAAT
TATTCGAATAGAATTAAGAATCCCAGGTTCATGAATTAATAATGATCAAATTTATAATTC
TTTTATTACAGCTCATGCATTTTTAATAATTTTTTTTTTAGTTATACCATTTTTAATTGG
AGGATTTGGAAATTGACTCACTCCATTAATATTAGGAGCTCCTGATATAGCATTTCCTCG
AATAAATAATATTAGATTTTGATTATTACCTCCTTCATTATTAATATTATTATCAAGAAA
TTTAATTAATCCAAGACCTGGAACAGGTTGAACTGTATATCCACCATTATCTTCTTATAC
TTATCATTCTTCTCCATCTGTTGATTTAGCAATTTTTTCTTTACATTTATCAGGAATTTC
ATCTATTATTGGATCAATAAATTTTATTGTAACAATTTTAATAATAAAAAATTATTCAAT
AAATTATAATCAAATACCATTATTTCCATGATCAGTTTTAATTACTACAATTTTATTATT
ATTATCATTACCCGTATTAGCAGGAGCTATTACAATATTATTATTTGATCGAAATCTAAA
TTCATCATTTTTTGATCCTATAGGAGGAGGAGATCCAATTTTATATCAACATTTATTT
>KR874988;tax=d:Eukarya,p:Arthropoda,c:Insecta,o:Hymenoptera,f:Halictidae,g:Agapostemon,s:Agapostemon
texanus
TTTATTTTTGCCATATGATCAGGTATAATTGGATCATCATTAAGAATATTAATTCGAATA
GAATTAAGAATTCCTGGAAGATGAATTTCTAATGATCAAATATTTAATACTATTATTACC
TCCCATGCATTTATTATAATTTTTTTTATAGTTATACCATTTATAATTGGGGGATTTGGA
AATTGATTAGTACCCCTAATAATTGGAGCACCTGATATAGCTTTTCCACGTATAAACAAT
ATAAGATTTTGACTTTTATCACCATCATTATTTTTATTATTATTAAGAAGAATATTAATA
TCAGGATCAGGAACAGGATGAACAATTTACCCCCCACTATCATCTTTCATTTATCATAAT
TCAATATCAGTTGATTTAACAATTTTTTCTCTACATATTGCAGGAATTTCATCTATTATA
GGAGCAATTAATTTTATTGTATCTATTATCATAATAAAAAATATTTCTTTAAAATTTGAC
CAAATCCCATTATTTCCATGATCAGTTAATATTACTGCTATTCTACTTTTATTATCCCTT
CCAGTATTAGCG
>KR875627;tax=d:Eukarya,p:Arthropoda,c:Insecta,o:Hymenoptera,f:Halictidae,g:Agapostemon,s:Agapostemon
texanus
TTTATTTTTGCCATATGATCAGGTATAATTGGATCATCATTAAGAATATTAATTCGAATA
GAATTAAGAATTCCTGGAAGATGAATTTCTAATGATCAAATATTTAATACTATTATTACC
TCCCATGCATTTATTATAATTTTTTTTATAGTTATACCATTTATAATTGGGGGATTTGGA
AATTGATTAGTACCCCTAATAATTGGAGCACCTGATATAGCTTTTCCACGTATAAACAAT
ATAAGATTTTGACTTTTATCACCATCATTATTTTTATTATTATTAAGAAGAATATTAATA

TCAGGATCAGGAACAGGATGAACAATTTACCCCCCACTATCATCTTTCATTTATCATAAT
TCAATATCAGTTGATTTAACAATTTTTTCTCTACATATTGCAGGAATTTCATCTATTATA
GGAGCAATTAATTTTATTGTATCTATTATCATAATAAAAAATATTTCTTTAAAATTTGAC
CAAATCCCATTATTTCCATGATCAGTTAATATTACTGCTATTCTACTTTTATTATCCCTT
CCAGTA
>KR883078;tax=d:Eukarya,p:Arthropoda,c:Insecta,o:Hymenoptera,f:Halictidae,g:Agapostemon,s:Agapostemon texanus
ATTTTATTTTTGCCATATGATCAGGTATAATTGGATCATCATTAAGAATATTAATTCGAA
TAGAATTAAGAATTCCTGGAAGATGAATTTCTAATGATCAAATATTTAATACTATTATTA
CCTCCCATGCATTTATTATAATTTTTTTTATAGTTATACCATTTATAATTGGGGGATTTG
GAAATTGATTAGTACCCCTAATAATTGGAGCACCTGATATAGCTTTTCCACGTATAAACA
ATATAAGATTTTGACTTTTATCACCATCATTATTTTTATTATTATTAAGAAGAATATTAA
TATCAGGATCAGGAACAGGATGAACAATTTACCCCCCACTATCATCTTTCATTTATCATA
ATTCAATATCAGTTGATTTAACAATTTTTTCTCTACATATTGCAGGAATTTCATCTATTA
TAGGAGCAATTAATTTTATTGTATCTATTATCATAATAAAAAATATTTCTTTAAAATTTG
ACCAAATCCCATTATTTCCATGATCAGTTAATATTACTGCTATTCTACTTTTATTATCCC
TTCCAGTATTAGCA
>KR888013;tax=d:Eukarya,p:Arthropoda,c:Insecta,o:Hymenoptera,f:Halictidae,g:Agapostemon,s:Agapostemon texanus
TTTATTTTTGCCATATGATCAGGTATAATTGGATCATCATTAAGAATATTAATTCGAATA
GAATTAAGAATTCCTGGAAGATGAATTTCTAATGATCAAATATTTAATACTATTATTACC
TCCCATGCATTTATTATAATTTTTTTTATAGTTATACCATTTATAATTGGGGGATTTGGA
AATTGATTAGTACCCCTAATAATTGGAGCACCTGATATAGCTTTTCCACGTATAAACAAT
ATAAGATTTTGACTTTTATCACCATCATTATTTTTATTATTATTAAGAAGAATATTAATA
TCAGGATCAGGAACAGGATGAACAATTTACCCCCCACTATCATCTTTCATTTATCATAAT
TCAATATCAGTTGATTTAACAATTTTTTCTCTACATATTGCAGGAATTTCATCTATTATA
GGAGCAATTAATTTTATTGTATCTATTATCATAATAAAAAATATTTCTTTAAAATTTGAC
CAAATCCCATTATTTCCATGATCAGTTAATATTACTGCTATTCTACTTTTATTATCCCTT
CCAGTATTAGCGGGTGCAATTACTATACTTTTAACCGAT
>MG337537;tax=d:Eukarya,p:Arthropoda,c:Insecta,o:Hymenoptera,f:Halictidae,g:Agapostemon,s:Agapostemon texanus
ATATTATATTTTATTTTTGCCATATGATCAGGTATAATTGGATCATCATTAAGAATATTA
ATTCGAATAGAATTAAGAATTCCTGGAAGATGAATTTCTAATGATCAAATATTTAATACT
ATTATTACCTCCCATGCATTTATTATAATTTTTTTTATAGTTATACCATTTATAATTGGG
GGATTTGGAAATTGATTAGTACCCCTAATAATTGGAGCACCTGATATAGCTTTTCCACGT
ATAAACAATATAAGATTTTGACTTTTATCACCATCATTATTTTTATTATTATTAAGAAGA
ATATTAATATCAGGATCAGGAACAGGATGAACAATTTACCCCCCACTATCATCTTTCATT
TATCATAATTCAATATCAGTTGATTTAACAATTTTTTCTCTACATATTGCAGGAATTTCA
TCTATTATAGGAGCAATTAATTTTATTGTATCTATTATCATAATAAAAAATATTTCTTTA
AAATTTGACCAAATCCCATTATTTCCATGATCAGTTAATATTACTGCTATTCTACTTTTA
TTATCCCTTCCAGTATTAGCGGGTGCAATTACTATACTTTTAACCGATCGA
>JN293819;tax=d:Eukarya,p:Arthropoda,c:Insecta,o:Hymenoptera,f:Colletidae,g:Colletes,s:Colletes phaceliae
AATATTATATTTTATTTTTGCTATATGAACAGGAATAGTTGGTTCTTCTTTAAGAATAAT
TATTCGTATAGAATTAAGTTCTCCAGGTATATGAATTAATAATGATCAAGTTTATAATTC
TATTGTTACTGCACATGCTTTTATTATAATTTTTTTTATAGTTATACCTTTTTTAATTGG
TGGATTTGGAAATTGATTAGTTCCATTNATAATTGGAGCACCTGATATAGCATTTCCTCG
AATAAATAATATAAGATTTTGATTATTACCTCCTTCTTTAATTCTTTTATTATTAAATAG
AATTTTATATTCAGGAAGAGGAACAGGATGAACTGTTTATCCTCCTTTATCTTCTTTAAT
ATATCATCCATCTTTATCTGTTGATTTAACAATTTTTTCTTTACATATTGCTGGAGTTTC
ATCAATTATAGGATCAATAAATTTTATTGTTACAATTTTAATAATAAAAAATNTAAATTT
AAATTATGATCAATTACCATTATTTTCATGATCTGTTTTTATTACAACAATTTTATTATT

ATTATCTTTACCTGTATTAGCTGGAGCAATTACAATATTATTAACTGATCGTAATTTAAA
TACTTCTTTTTTTGATCCATCTGGTGGAGGAGATCCAATTCTTTATCAACATTTATTT
>MH747974;tax=d:Eukarya,p:Arthropoda,c:Insecta,o:Hymenoptera,f:Apidae,g:Bombus,s:Bombus fervidus
GATATAGCATTCCCTCGAATAAATAATATTAGATTTTGATTACTTCCTCCTTCATTAATA
ATATTATTATTAAGAAATTTATTTACACCTAATGTTGGAACAGGATGAACTGTTTATCCT
CCTTTGTCATCATATTTATTTCATTCATCTCCATCTGTAGATATTGCAATTTTTTCTTTA
CATATAACAGGAATTTCTTCAATTATTGGTTCTTTAAATTTTATTGTTACTATTATAATA
ATAAAAAATTATTCTTTAAATTATGATCAAATTAATTTATTTTCATGATCAGTTTGCATT
ACAGTTATTTTATTAATTTTATCTTTACCTGTATTAGCAGGTGCAATTACTATATTACTT
TTTGATCGAAATTTTAATACATCTTTTTTTGACCCTATAGGAGGGGGAGATCCAATTCTT
TATCAACATTTATTT
>MH747982;tax=d:Eukarya,p:Arthropoda,c:Insecta,o:Hymenoptera,f:Apidae,g:Bombus,s:Bombus fervidus
GATATAGCATTCCCTCGAATAAATAATATTAGATTTTGATTACTTCCTCCTTCATTAATA
ATATTATTATTAAGAAATTTATTTACACCTAATGTTGGAACAGGATGAACTGTTTATCCT
CCTTTGTCATCATATTTATTTCATTCATCTCCATCTGTAGATATTGCAATTTTTTCTTTA
CATATAACAGGAATTTCTTCAATTATTGGTTCTTTAAATTTTATTGTTACTATTATAATA
ATAAAAAATTATTCTTTAAATTATGATCAAATTAATTTATTTTCATGATCAGTTTGCATT
ACAGTTATTTTATTAATTTTATCTTTACCTGTATTAGCAGGTGCAATTACTATATTACTT
TTTGATCGAAATTTTAATACATCTTTTTTTGATCCTATAGGAGGAGGAGATCCAATTCTT
TATCAACATTTATTT
>MH747973;tax=d:Eukarya,p:Arthropoda,c:Insecta,o:Hymenoptera,f:Apidae,g:Bombus,s:Bombus fervidus
GATATAGCATTCCCTCGAATAAATAATATTAGATTTTGATTACTTCCTCCTTCATTAATA
ATATTATTATTAAGAAATTTATTTACACCTAATGTTGGAACAGGATGAACTGTTTATCCT
CCTTTATCATCATATTTATTTCATTCATCTCCATCTGTAGATATTGCAATTTTTTCTTTA
CATATAACAGGAATTTCTTCAATTATTGGTTCTTTAAATTTTATTGTTACTATTATAATA
ATAAAAAATTATTCTTTAAATTATGATCAAATTAATTTATTTTCATGATCAGTTTGCATT
ACAGTTATTTTATTAATTTTATCTTTACCTGTATTAGCAGGTGCAATTACTATATTACTT
TTTGATCGAAATTTTAATACATCTTTTTTTGATCCTATAGGAGGGGGAGATCCAATTCTT
TATCAACATTTATTT
>KR881048;tax=d:Eukarya,p:Arthropoda,c:Insecta,o:Hymenoptera,f:Apidae,g:Bombus,s:Bombus fervidus
AATAATATATTTTATTTTTGCTATATGATCAGGAATAATTGGTTCATCAATAAGTTTATT
AATTCGAATAGAATTAAGACATCCTGGTATATGAATTAATAATGATCAAATTTATAATGC
TTTAGTTACTAGTCATGCATTTTTAATAATTTTTTTTATAGTTATACCATTTTTAATTGG
AGGATTTGGAAATTATTTAATTCCTTTAATATTAGGTTCTCCTGATATAGCATTTCCTCG
AATAAATAATATTAGATTTTGATTACTTCCTCCTTCATTAATAATATTATTATTAAGAAA
TTTATTTACACCTAATGTTGGAACAGGATGAACTATTTATCCTCCTTTATCATCATATTT
ATTCCATTCATCCCCATCTGTAGATATTGCAATTTTTTCTTTGCATATAACAGGAATTTC
TTCAATTATTGGTTCTTTAAATTTTATTGTTACTATTATAATAATAAAAAATTATTCTTT
AAATTATGATCAAATTAATTTATTTTCATGATCAGTTTGTATTACAGTTATTTTATTAAT
TTTATCTTTACCTGTATTAGCAGGTGCAATTACTATATTACTTTTTGAT
>FJ582118;tax=d:Eukarya,p:Arthropoda,c:Insecta,o:Hymenoptera,f:Apidae,g:Bombus,s:Bombus fervidus
GCTATATGATCAGGAATAATTGGTTCGTCAATAAGTTTATTAATTCGAATAGAATTAAGA
CATCCTGGTATATGAATTAATAATGATCAAGTTTATAATGCTTTAGTTACTAGTCATGCA
TTTTTAATAATTTTTTTTATAGTTATACCATTTTTAATTGGAGGATTTGGAAATTATTTA
ATTCCTTTAATATTAGGTTCTCCTGATATAGCATTCCCTCGAATAAATAATATTAGATTT
TGATTACTTCCTCCTTCATTAATAATATTATTATTAAGAAATTTATTTACACCTAATGTT
GGAACAGGATGAACTGTTTATCCTCCTTTGTCATCATATTTATTTCATTCATCTCCATCT
GTAGATATTGCAATTTTTTCTTTACATATAACAGGAATTTCTTCAATTATTGGTTCTTTA
AATTTTATTGTTACTATTATAATAATAAAAAATTATTCTTTAAATTATGATCAAATTAAT
TTATTTTCATGATCAGTTTGCATTACAGTTATTTTATTAATTTTATCTTTACCTGTATTA
GCAGGTGCAATTACTATATTACTTTTTGATCGAAATTTTAATACATCTTTTTTTGACCCT

ATAGGAGGGGGAGATCCAATTCTTTATCAACATTTATTT
>FJ582121;tax=d:Eukarya,p:Arthropoda,c:Insecta,o:Hymenoptera,f:Apidae,g:Bombus,s:Bombus fervidus
GCTATATGATCAGGAATAATTGGTTCGTCAATAAGTTTATTAATTCGAATAGAATTAAGA
CATCCTGGTATATGAATTAATAATGATCAAGTTTATAATGCTTTAGTTACTAGTCATGCA
TTTTTAATAATTTTTTTTATAGTTATACCATTTTTAATTGGAGGATTTGGAAATTATTTA
ATTCCTTTAATATTAGGTTCTCCTGATATAGCATTCCCTCGAATAAATAATATTAGATTT
TGATTACTTCCTCCTTCATTAATAATATTATTATTAAGAAATTTATTTACACCTAATGTT
GGAACAGGATGAACTGTTTATCCTCCTTTGTCATCATATTTATTTCATTCATCTCCATCT
GTAGATATTGCAATTTTTTCTTTACATATAACAGGAATTTCTTCAATTATTGGTTCTTTA
AATTTTATTGTTACTATTATAATAATAAAAAATTATTCTTTAAATTATGATCAAATTAAT
TTATTTTCATGATCAGTTTGCATTACAGTTATTTTATTAATTTTATCTTTACCTGTATTA
GCAGGTGCAATTACTATATTACTATCTGACCGAAATTTTAATACATCTT
>HM422924;tax=d:Eukarya,p:Arthropoda,c:Insecta,o:Hymenoptera,f:Apidae,g:Bombus,s:Bombus fervidus
AATAATATATTTTATTTTTGCTATATGATCAGGAATAATTGGTTCGTCAATAAGTTTATT
AATTCGAATAGAATTAAGACATCCTGGTATATGAATTAATAATGATCAAGTTTATAATGC
TTTAGTTACTAGTCATGCATTTTTAATAATTTTTTTTATAGTTATACCATTTTTAATTGG
AGGATTTGGAAATTATTTAATTCCTTTAATATTAGGTTCTCCTGATATAGCATTCCCTCG
AATAAATAATATTAGATTTTGATTACTTCCTCCTTCATTAATAATATTATTATTAAGAAA
TTTATTTACACCTAATGTTGGAACAGGATGAACTGTTTATCCTCCTTTGTCATCATATTT
ATTTCATTCATCTCCATCTGTAGATATTGCAATTTTTTCTTTACATATAACAGGAATTTC
TTCAATTATTGGTTCTTTAAATTTTATTGTTACTATTATAATAATAAAAAATTATTCTTT
AAATTATGATCAAATTAATTTATTTTCATGATCAGTTTGCATTACAGTTATTTTATTAAT
TTTATCTTTACCTGTATTAGCAGGTGCAATTACTATATTACTTTTTGATCGAAATTTTAA
TACATCTTTTTTTGACCCTATAGGAGGGGGAGATCCAATTCTTTATCAACATTTATTT
>KR874959;tax=d:Eukarya,p:Arthropoda,c:Insecta,o:Hymenoptera,f:Apidae,g:Bombus,s:Bombus fervidus
GGTTCGTCAATAAGTTTATTAATTCGAATAGAATTAAGACATCCTGGTATATGAATTAAT
AATGATCAAGTTTATAATGCTTTAGTTACTAGTCATGCATTTTTAATAATTTTTTTTATA
GTTATACCATTTTTAATTGGAGGATTTGGAAATTATTTAATTCCTTTAATATTAGGTTCT
CCTGATATAGCATTCCCTCGAATAAATAATATTAGATTTTGATTACTTCCTCCTTCATTA
ATAATATTATTATTAAGAAATTTATTTACACCTAATGTTGGAACAGGATGAACTGTTTAT
CCTCCTTTATCATCATATTTATTTCATTCATCTCCATCTGTAGATATTGCAATTTTTTCT
TTACATATAACAGGAATTTCTTCAATTATTGGTTCTTTAAATTTTATTGTTACTATTATA
ATAATAAAAAATTATTCTTTAAATTATGATCAAATTAATTTATTTTCATGATCAGTTTGC
ATTACAGTTATTTTATTAATTTTATCTTTACCTGTATTAGCAGGT
>KR879473;tax=d:Eukarya,p:Arthropoda,c:Insecta,o:Hymenoptera,f:Apidae,g:Bombus,s:Bombus fervidus
AATAATATATTTTATTTTTGCTATATGATCAGGAATAATTGGTTCGTCAATAAGTTTATT
AATTCGAATAGAATTAAGACATCCTGGTATATGAATTAATAATGATCAAGTTTATAATGC
TTTAGTTACTAGTCATGCATTTTTAATAATTTTTTTTATAGTTATACCATTTTTAATTGG
AGGATTTGGAAATTATTTAATTCCTTTAATATTAGGTTCTCCTGATATAGCATTCCCTCG
AATAAATAATATTAGATTTTGATTACTTCCTCCTTCATTAATAATATTATTATTAAGAAA
TTTATTTACACCTAATGTTGGAACAGGATGAACTGTTTATCCTCCTTTATCATCATATTT
ATTTCATTCATCTCCATCTGTAGATATTGCAATTTTTTCTTTACATATAACAGGAATTTC
TTCAATTATTGGCTCTTTAAATTTTATTGTTACTATTATAATAATAAAAAATTATTCTTT
AAATTATGATCAAATTAATTTATTTTCATGATCAGTTTGCATTACAGTTATTTTATTAAT
TTTATCTTTACCTGTATTAGCAGGTGCAATTACTATATTACTTTTTGATCG
>KR883161;tax=d:Eukarya,p:Arthropoda,c:Insecta,o:Hymenoptera,f:Apidae,g:Bombus,s:Bombus fervidus
AATAATATATTTTATTTTTGCTATATGATCAGGAATAATTGGTTCGTCAATAAGTTTATT
AATTCGAATAGAATTAAGACATCCTGGTATATGAATTAATAATGATCAAGTTTATAATGC
TTTAGTTACTAGTCATGCATTTTTAATAATTTTTTTTATAGTTATACCATTTTTAATTGG
AGGATTTGGAAATTATTTAATTCCTTTAATATTAGGTTCTCCTGATATAGCATTCCCTCG
AATAAATAATATTAGATTTTGATTACTTCCTCCTTCATTAATAATAATATTATTAAGAAA

TTTATTTACACCTAATGTTGGAACAGGATGAACTGTTTATCCTCCTTTATCATCATATTT
ATTTCATTCATCTCCATCTGTAGATATTGCAATTTTTTCTTTACATATAACAGGAATTTC
TTCAATTATTGGTTCTTTAAATTTTATTGTTACTATTATAATAATAAAAAATTATTCTTT
AAATTATGATCAAATTAATTTATTTTCATGATCAGTTTGCATTACAGTTATTTTATTAAT
TTTATCTTTACCTGTATTAGCAGGTGCAATTACTATATTACTTTTTGATCGAAATTTTAA
TACATCTTTTTTTG
>KR893758;tax=d:Eukarya,p:Arthropoda,c:Insecta,o:Hymenoptera,f:Apidae,g:Bombus,s:Bombus fervidus
AATAATATATTTTATTTTTGCTATATGATCAGGAATAATTGGTTCATCAATAAGTTTATT
AATTCGAATAGAATTAAGACATCCTGGTATATGAATTAATAATGATCAAATTTATAATGC
TTTAGTTACTAGTCATGCATTTTTAATAATTTTTTTTATAGTTATACCATTTTTAATTGG
AGGATTTGGAAATTATTTAATTCCTTTAATATTAGGTTCTCCTGATATAGCATTTCCTCG
AATAAATAATATTAGATTTTGATTACTTCCTCCTTCATTAATAATATTATTATTAAGAAA
TTTATTTACACCTAATGTTGGAACAGGATGAACTATTTATCCTCCTTTATCATCATATTT
ATTCCATTCATCTCCATCTGTAGATATTGCAATTTTTTCTTTGCATATAACAGGAATTTC
TTCAATTATTGGTTCTTTAAATTTTATTGTTACTATTATAATAATAAAAAATTATTCTTT
AAATTATGATCAAATTAATTTATTTTCATGATCAGTTTGTATTACAGTTATTTTATTAAT
TTTATCTTTACCTGTATTAGCAGGTGCAATTACTATATTACTTTTTGATCGA
>KR895885;tax=d:Eukarya,p:Arthropoda,c:Insecta,o:Hymenoptera,f:Apidae,g:Bombus,s:Bombus fervidus
AATAATATATTTTATTTTTGCTATATGATCAGGAATAATTGGTTCATCAATAAGTTTATT
AATTCGAATAGAATTAAGACATCCTGGTATATGAATTAATAATGATCAAATTTATAATGC
TTTAGTTACTAGTCATGCATTTTTAATAATTTTTTTTATAGTTATACCATTTTTAATTGG
AGGATTTGGAAATTATTTAATTCCTTTAATATTAGGTTCTCCTGATATAGCATTTCCTCG
AATAAATAATATTAGATTTTGATTACTTCCTCCTTCATTAATAATATTATTATTAAGAAA
TTTATTTACACCTAATGTTGGAACAGGATGAACTATTTATCCTCCTTTATCATCATATTT
ATTCCATTCATCCCCATCTGTAGATATTGCAATTTTTTCTTTGCATATAACAGGAATTTC
TTCAATTATTGGTTCTTTAAATTTTATTGTTACTATTATAATAATAAAAAATTATTCTTT
AAATTATGATCAAATTAATTTATTTTCATGATCAGTTTGTATTACAGTTATTTTATTAAT
TTTATCTTTACCTGTATTAGCAGGTGCAATTACTATATTACTTTTTGATCGAAATTTTAA
TACATCTTTTTTTGATCCTATAGGAGGAGGAGATCCAATTCTTTATCAACATTTATTT
>KR897017;tax=d:Eukarya,p:Arthropoda,c:Insecta,o:Hymenoptera,f:Apidae,g:Bombus,s:Bombus fervidus
ATAATATATTTTATTTTTGCTATATGATCAGGAATAATTGGTTCGTCAATAAGTTTATTA
ATTCGAATAGAATTAAGACATCCTGGTATATGAATTAATAATGATCAAGTTTATAATGCT
TTAGTTACTAGTCATGCATTTTTAATAATTTTTTTTATAGTTATACCATTTTTAATTGGA
GGATTTGGAAATTATTTAATTCCTTTAATATTAGGTTCTCCTGATATAGCATTCCCTCGA
ATAAATAATATTAGATTTTGATTACTTCCTCCTTCATTAATAATATTATTATTAAGAAAT
TTATTTACACCTAATGTTGGAACAGGATGAACTGTTTATCCTCCTTTATCATCATATTTA
TTTCATTCATCTCCATCTGTAGATATTGCAATTTTTTCTTTACATATAACAGGAATTTCT
TCAATTATTGGTTCTTTAAATTTTATTGTTACTATTATAATAATAAAAAATTATTCTTTA
AATTATGATCAAATTAATTTATTTTCATGATCAGTTTGCATTACAGTTATTTTATTAATT
TTATCTTTACCTGTATTAGCAGGT
>KR897356;tax=d:Eukarya,p:Arthropoda,c:Insecta,o:Hymenoptera,f:Apidae,g:Bombus,s:Bombus fervidus
AATAATATATTTTATTTTTGCTATATGATCAGGAATAATTGGTTCGTCAATAAGTTTATT
AATTCGAATAGAATTAAGACATCCTGGTATATGAATTAATAATGATCAAGTTTATAATGC
TTTAGTTACTAGTCATGCATTTTTAATAATTTTTTTTATAGTTATACCATTTTTAATTGG
AGGATTTGGAAATTATTTAATTCCTTTAATATTAGGTTCTCCTGATATAGCATTCCCTCG
AATAAATAATATTAGATTTTGATTACTTCCTCCTTCATTAATAATATTATTATTAAGAAA
TTTATTTACACCTAATGTTGGAACAGGATGAACTGTTTATCCTCCTTTATCATCATATTT
ATTTCATTCATCTCCATCTGTAGATATTGCAATTTTTTCTTTACATATAACAGGAATTTC
TTCAATTATTGGTTCTTTAAATTTTATTGTTACTATTATAATAATAAAAAATTATTCTTT
AAATTATGATCAAATTAATTTATTTTCATGATCAGTTTGCATTACAGTTATTTTATTAAT
TTTATCTTTACCTGTATTAGCAGGTGCAATTACTATATTACTTTTTGATCGAAATTTTAA

TAC
>KR898805;tax=d:Eukarya,p:Arthropoda,c:Insecta,o:Hymenoptera,f:Apidae,g:Bombus,s:Bombus fervidus
AATAATATATTTTATTTTTGCTATATGATCAGGAATAATTGGTTCATCAATAAGTTTATT
AATTCGAATAGAATTAAGACATCCTGGTATATGAATTAATAATGATCAAATTTATAATGC
TTTAGTTACTAGTCATGCATTTTTAATAATTTTTTTTATAGTTATACCATTTTTAATTGG
AGGATTTGGAAATTATTTAATTCCTTTAATATTAGGTTCTCCTGATATAGCATTTCCTCG
AATAAATAATATTAGATTTTGATTACTTCCTCCTTCATTAATAATATTATTATTAAGAAA
TTTATTTACACCTAATGTTGGAACAGGATGAACTATTTATCCTCCTTTATCATCATATTT
ATTCCATTCATCCCCATCTGTAGATATTGCAATTTTTTCTTTGCATATAACAGGAATTTC
TTCAATTATTGGTTCTTTAAATTTTATTGTTACTATTATAATAATAAAAAATTATTCTTT
AAATTATGATCAAATTAATTTATTTTCATGATCAGTTTGTATTACAGTTATTTTATTAAT
TTTATCTTTACCTGTATTAGCAGGTGCAATTACTATATTACTTTTTGATCGAAATTTTAA
TACATCTTTTTTTGA
>MG448456;tax=d:Eukarya,p:Arthropoda,c:Insecta,o:Hymenoptera,f:Apidae,g:Bombus,s:Bombus fervidus
TATATTTTATTTTTGCTATATGATCAGGAATAATTGGTTCGTCAATAAGTTTATTAATTC
GAATAGAATTAAGACATCCTGGTATATGAATTAATAATGATCAAGTTTATAATGCTTTAG
TTACTAGTCATGCATTTTTAATAATTTTTTTTATAGTTATACCATTTTTAATTGGAGGAT
TTGGAAATTATTTAATTCCTTTAATATTAGGTTCTCCTGATATAGCATTCCCTCGAATAA
ATAATATTAGATTTTGATTACTTCCTCCTTCATTAATAATATTATTATTAAGAAATTTAT
TTACACCTAATGTTGGAACAGGATGAACTGTTTATCCTCCTTTGTCATCATATTTATTTC
ATTCATCTCCATCTGTAGATATTGCAATTTTTTCTTTACATATAACAGGAATTTCTTCAA
TTATTGGTTCTTTAAATTTTATTGTTACTATTATAATAATAAAAAATTATTCTTTAAATT
ATGATCAAATTAATTTATTTTCATGATCAGTTTGCATTACAGTTATTTTATTAATTTTAT
CTTTACCTGTATTAGCAGGTGCAATTACTATATTACTTTTTGATCGAAATTTTAATACAT
CTTTTTTTGACCCTATAGGAGGGGGAGAACCAATTCTTTATCAACATTTA
>GU707670;tax=d:Eukarya,p:Arthropoda,c:Insecta,o:Hymenoptera,f:Apidae,g:Bombus,s:Bombus rufocinctus
AATAATATATTTTATTTTTGCTATATGATCAGGAATAATTGGTTCATCAATAAGATTATT
AATTCGAATAGAATTAAGACATCCTGGAATATGAATCAATAATGATCAAATTTATAATTC
ATTAGTAACTAGACATGCATTTTTAATAATTTTTTTTATAGTTATACCATTTATAATTGG
AGGTTTTGGAAATTATTTAATTCCTTTAATATTGGGGTCACCTGATATAGCTTTTCCTCG
TATAAATAATATTAGATTTTGATTACTCCCTCCATCATTAATAATACTACTTTTAAGAAA
TTTATTTACACCAAATGTAGGAACAGGATGAACTGTTTATCCTCCATTATCATCTTACCT
TTTTCATTCATCACCATCAGTTGATATTGCAATTTTCTCTTTACATATAACAGGAATTTC
TTCCATTATTGGATCTTTAAATTTTATTGTAACAATTATATTTATAAAAAATTTCTCTTT
AAATTATGATCAAATTAACTTATTCTCTTGATCTGTTTGTATTACAGTAATTTTATTAAT
TCTATCCTTACCAGTTTTAGCGGGAGCTATTACTATACTTCTATTTGATCGAAATTTTAA
TACATCTTTCTTTGATCCTATAGGAGGTGGTGATCCTATTCTTTATCAACACTTATTT
>KR896959;tax=d:Eukarya,p:Arthropoda,c:Insecta,o:Hymenoptera,f:Apidae,g:Bombus,s:Bombus rufocinctus
ATAATATATTTTATTTTTGCTATATGATCAGGAATAATTGGTTCATCAATAAGATTATTA
ATTCGAATAGAATTAAGACATCCTGGAATATGAATCAATAATGATCAAATTTATAATTCA
TTAGTAACTAGACATGCATTTTTAATAATTTTTTTTATAGTTATACCATTTATAATTGGA
GGTTTTGGAAATTATTTAATTCCTTTAATATTGGGATCACCTGATATAGCTTTTCCTCGT
ATAAATAATATTAGATTTTGATTACTCCCTCCATCATTAATAATACTACTATTAAGAAAT
TTATTTACACCAAATGTAGGAACAGGATGAACTGTTTATCCTCCATTATCATCTTACCTT
TTTCATTCATCACCATCAGTTGATATTGCAATTTTCTCTTTACATATAACAGGAATTTCT
TCCATTATTGGATCTTTAAATTTTATTGTAACAATTATATTTATAAAAAATTTTTCTTTA
AATTATGATCAAATTAACTTATTCTCTTGATCTGTTTGTATTACAGTAATTTTATTAATT
CTATCCTTACCAGTTTTAGCGGGAGCTATTACTATACTTCTATTTGAT
>GU707741;tax=d:Eukarya,p:Arthropoda,c:Insecta,o:Hymenoptera,f:Apidae,g:Bombus,s:Bombus rufocinctus
AATAATATATTTTATTTTTGCTATATGATCAGGAATAATTGGTTCATCAATAAGATTATT
AATTCGAATAGAATTAAGACATCCTGGAATATGAATCAATAATGATCAAATTTATAATTC

ATTAGTAACTAGACATGCATTTTTAATAATTTTTTTTATAGTTATACCATTTATAATTGG
AGGTTTTGGAAATTATTTAATTCCTTTAATATTGGGATCACCTGATATAGCTTTTCCTCG
TATAAATAATATTAGATTTTGATTACTCCCTCCATCATTAATAATACTACTATTAAGAAA
TTTATTTACACCAAATGTAGGAACAGGATGAACTGTTTATCCTCCATTATCATCTTACCT
TTTTCATTCATCACCATCAGTTGATATTGCAATTTTCTCTTTACATATAACAGGAATTTC
TTCCATTATTGGATCTTTAAATTTTATTGTAACAATTATATTTATAAAAAATTTTTCTTT
AAATTATGATCAAATTAACTTATTCTCTTGATCTGTTTGTATTACAGTAATTTTATTAAT
TCTATCCTTACCAGTTTTAGCGGGAGCTATTACTATACTTCTATTTGATCGAAATTTTAA
TACATCTTTCTTTGATCCTATAGGAGGTGGTGATCCTATTCTTTATCAACACTTATTT
>JN293686;tax=d:Eukarya,p:Arthropoda,c:Insecta,o:Hymenoptera,f:Apidae,g:Bombus,s:Bombus rufocinctus
AATAATATATTTTATTTTTGCTATATGATCAGGAATAATTGGTTCATCAATAAGATTATT
AATTCGAATAGAATTAAGACATCCTGGAATATGAATCAATAATGATCAAATTTATAATTC
ATTAGTAACTAGACATGCATTTTTAATAATTTTTTTTATAGTTATACCATTTATAATTGG
AGGTTTTGGAAATTATTTAATTCCTTTAATATTGGGATCACCTGATATAGCTTTTCCTCG
TATAAATAATATTAGATTTTGATTACTCCCTCCATCATTAATAATACTACTATTAAGAAA
TTTATTTACACCAAATGTAGGAACAGGATGAACTGTTTATCCTCCATTATCATCTTACCT
TTTTCATTCATCACCATCAGTTGATATTGCAATTTTCTCTTTACATATAACAGGAATTTC
TTCCATTATTGGATCTTTAAATTTTATTGTAACAATTATATTTATAAAAAATTTTTCTTT
AAATTATGATCAAATTAACTTATTCTCTTGATCTGTTTGTATTACAGTAATTTTATTAAT
TCTATCCTTACCAGTTTTAGCGGGAGCTATTACTATACTTCTATTTGATCGAAATTTTAA
TACATCTTTCTTTGATCCTATAGGGGGTGGTGATCCTATTCTTTATCAACACTTATTT
>KR786746;tax=d:Eukarya,p:Arthropoda,c:Insecta,o:Hymenoptera,f:Apidae,g:Bombus,s:Bombus rufocinctus
AATAATATATTTTATTTTTGCTATATGATCAGGAATAATTGGTTCATCAATAAGATTATT
AATTCGAATAGAATTAAGACATCCTGGAATATGAATCAATAATGATCAAATTTATAATTC
ATTAGTAACTAGACATGCATTTTTAATAATTTTTTTTATAGTTATACCATTTATAATTGG
AGGTTTTGGAAATTATTTAATTCCTTTAATATTGGGGTCACCTGATATAGCTTTTCCTCG
TATAAATAATATTAGATTTTGATTACTCCCTCCATCATTAATAATACTACTATTAAGAAA
TTTATTTACACCAAATGTAGGAACAGGATGAACTGTTTATCCTCCATTATCATCTTACCT
TTTTCATTCATCACCATCAGTTGATATTGCAATTTTCTCTTTACATATAACAGGAATTTC
TTCCATTATTGGATCTTTAAATTTTATTGTAACAATTATATTTATAAAAAATTTTTCTTT
AAATTATGACCAAATTAACTTATTCTCTTGATCTGTTTGTATTACAGTAATTTTATTAAT
TCTATCCTTACCAGTTTTAGCGGGAGCTATTACTATACTTCTATTTGATCGAAATTTTAA
TACATCTTTCTTTGATCCTATAGGAGGTGGTGATCCTATTCTTTATCAACACTTATTT
>KR787946;tax=d:Eukarya,p:Arthropoda,c:Insecta,o:Hymenoptera,f:Apidae,g:Bombus,s:Bombus rufocinctus
AATAATATATTTTATTTTTGCTATATGATCAGGAATAATTGGTTCATCAATAAGATTATT
AATTCGAATAGAATTAAGACATCCTGGAATATGAATCAATAATGATCAAATTTATAATTC
ATTAGTAACTAGACATGCATTTTTAATAATTTTTTTTATAGTTATACCATTTATAATTGG
AGGTTTTGGAAATTATTTAATTCCTTTAATATTAGGGTCACCTGATATAGCTTTTCCTCG
TATAAATAATATTAGATTTTGATTACTTCCTCCATCATTAATAATACTACTATTAAGAAA
TTTATTTACACCAAATGTAGGAACAGGATGAACTGTTTATCCTCCATTATCATCTTACCT
TTTTCATTCATCACCATCAGTTGATATTGCAATTTTCTCTTTACATATAACAGGAATTTC
TTCCATTATTGGATCTTTAAATTTTATTGTAACAATTATATTTATAAAAAATTTTTCTTT
AAATTATGATCAAATTAACTTATTTTCTTGATCTGTTTGTATTACAGTAATTTTATTAAT
TCTATCCTTACCAGTTTTAGCGGGAGCTATTACTATACTTCTATTTGATCGAAATTTTAA
TACATCTTTCTTTGATCCTATAGGAGGTGGTGATCCTATTCTTTATCAACACTTATTT
>KR789506;tax=d:Eukarya,p:Arthropoda,c:Insecta,o:Hymenoptera,f:Apidae,g:Bombus,s:Bombus rufocinctus
AATAATATATTTTATTTTTGCTATATGATCAGGAATAATTGGTTCATCAATAAGATTATT
AATTCGAATAGAATTAAGACATCCTGGAATATGAATCAATAATGATCAAATTTATAATTC
ATTAGTAACTAGACATGCATTTTTAATAATTTTTTTTATAGTTATACCATTTATAATTGG
AGGTTTTGGAAATTATTTAATTCCTTTAATATTGGGGTCACCTGATATAGCTTTTCCTCG
TATAAATAATATTAGATTTTGATTACTCCCTCCATCATTAATAATACTACTATTAAGAAA

TTTATTTACACCAAATGTAGGAACAGGATGAACTGTTTATCCTCCATTATCATCTTACCT
TTTTCATTCATCACCATCAGTTGATATTGCAATTTTCTCTTTACATATAACAGGAATTTC
TTCCATTATTGGATCTTTAAATTTTATTGTAACAATTATATTTATAAAAAATTTTTCTTT
AAATTATGATCAAATTAACTTATTCTCTTGATCTGTTTGTATTACAGTAATTTTATTAAT
TCTATCCTTACCAGTTTTAGCGGGAGCTATTACTATACTTCTATTTGATCGAAATTTTAA
TACATCTTTCTTTGA
>KR791971;tax=d:Eukarya,p:Arthropoda,c:Insecta,o:Hymenoptera,f:Apidae,g:Bombus,s:Bombus rufocinctus
AATAATATATTTTATTTTTGCTATATGATCAGGAATAATTGGTTCATCAATAAGATTATT
AATTCGAATAGAATTAAGACATCCTGGAATATGAATCAATAATGATCAAATTTATAATTC
ATTAGTAACTAGACATGCATTTTTAATAATTTTTTTTATAGTTATACCATTTATAATTGG
AGGTTTTGGAAATTATTTAATTCCTTTAATATTGGGGTCACCTGATATAGCTTTTCCTCG
TATAAATAATATTAGATTTTGATTACTCCCTCCATCATTAATAATACTACTATTAAGAAA
TTTATTTACACCAAATGTAGGAACAGGATGAACTGTTTATCCTCCATTATCATCTTACCT
TTTTCATTCATCACCATCAGTTGATATTGCAATTTTCTCTTTACATATAACAGGAATTTC
TTCCATTATTGGATCTTTAAATTTTATTGTAACAATTATATTTATAAAAAATTTTTCTTT
AAATTATGATCAAATTAACTTATTCTCTTGATCTGTTTGTATTACAGTAATTTTATTAAT
TCTATCCTTACCAGTTTTAGCGGGAGC
>KR794479;tax=d:Eukarya,p:Arthropoda,c:Insecta,o:Hymenoptera,f:Apidae,g:Bombus,s:Bombus rufocinctus
AATAATATATTTTATTTTTGCTATATGATCAGGAATAATTGGTTCATCAATAAGATTATT
AATTCGAATAGAATTAAGACATCCTGGAATATGAATCAATAATGATCAAATTTATAATTC
ATTAGTAACTAGACATGCATTTTTAATAATTTTTTTTATAGTTATACCATTTATAATTGG
AGGTTTTGGAAATTATTTAATTCCTTTAATATTGGGGTCACCTGATATAGCTTTTCCTCG
TATAAATAATATTAGATTTTGATTACTCCCTCCATCATTAATAATACTACTTTTAAGAAA
TTTATTTACACCAAATGTAGGAACAGGATGAACTGTTTATCCTCCATTATCATCTTACCT
TTTTCATTCATCACCATCAGTTGATATTGCAATTTTCTCTTTACATATAACAGGAATTTC
TTCCATTATTGGATCTTTAAATTTTATTGTAACAATTATATTTATAAAAAATTTCTCTTT
AAATTATGATCAAATTAACTTATTCTCTTGATCTGTTTGTATTACAGTAATTTTATTAAT
TCTATCCTTACCAGTTTTAGCGGGAGCTATTACTATACTTCTATTTGATCGAAATTTTAA
TACATCTTTCTTTGATCCTATAGGAGGTGGTGATCC
>KR796908;tax=d:Eukarya,p:Arthropoda,c:Insecta,o:Hymenoptera,f:Apidae,g:Bombus,s:Bombus rufocinctus
AATAATATACTTTATTTTTGCTATATGATCAGGAATAATTGGTTCATCAATAAGATTATT
AATTCGAATAGAATTAAGACATCCTGGAATATGAATCAATAATGATCAAATTTATAATTC
ATTAGTAACTAGACATGCATTTTTAATAATTTTTTTTATAGTTATACCATTTATAATTGG
AGGTTTTGGAAATTATTTAATTCCTTTAATATTGGGGTCACCTGATATAGCTTTTCCTCG
TATAAATAATATTAGATTTTGATTACTCCCTCCATCATTAATAATACTACTATTAAGAAA
TTTATTTACACCAAATGTAGGAACAGGATGAACTGTTTATCCTCCATTATCATCTTACCT
TTTTCATTCATCACCATCAGTTGATATTGCAATTTTCTCTTTACATATAACAGGAATTTC
TTCCATTATTGGATCTTTAAATTTTATTGTAACAATTATATTTATAAAAAATTTTTCTTT
AAATTATGATCAAATTAACTTATTCTCTTGATCTGTTTGTATTACAGTAATTTTATTAAT
TCTATCCTTACCAGTTTTAGCGGGAGCTATTACTATACT
>KR806373;tax=d:Eukarya,p:Arthropoda,c:Insecta,o:Hymenoptera,f:Apidae,g:Bombus,s:Bombus rufocinctus
AATAATATATTTTATTTTTGCTATATGATCAGGAATAATTGGTTCATCAATAAGATTATT
AATTCGAATAGAATTAAGACATCCTGGAATATGAATCAATAATGATCAAATTTATAATTC
ATTAGTAACTAGACATGCATTTTTAATAATTTTTTTTATAGTTATACCATTTATAATTGG
AGGTTTTGGAAATTATTTAATTCCTTTAATATTGGGGTCACCTGATATAGCTTTTCCTCG
TATAAATAATATTAGATTTTGATTACTCCCTCCATCATTAATAATACTACTATTAAGAAA
TTTATTTACACCAAATGTAGGAACAGGATGAACTGTTTATCCTCCATTATCATCTTACCT
TTTTCATTCATCACCATCAGTTGATATTGCAATTTTCTCTTTACATATAACAGGAATTTC
TTCCATTATTGGATCTTTAAATTTTATTGTAACAATTATATTTATAAAAAATTTTTCTTT
AAATTATGACCAAATTAACTTATTCTCTTGATCTGTTTGTATTACAGTAATTTTATTAAT
TCTATCCTTACCAGTTTTAGCGGGAGCTATTACTATACTTCTATTTGATCGAAATTTTAA

TACATCTTTCTTTGATCCTATAGGAGG
>KR808559;tax=d:Eukarya,p:Arthropoda,c:Insecta,o:Hymenoptera,f:Apidae,g:Bombus,s:Bombus rufocinctus
AATAATATATTTTATTTTTGCTATATGATCAGGAATAATTGGTTCATCAATAAGATTATT
AATTCGAATAGAATTAAGACATCCTGGAATATGAATCAATAATGATCAAATTTATAATTC
ATTAGTAACTAGACATGCATTTTTAATAATTTTTTTTATAGTTATACCATTTATAATTGG
AGGTTTTGGAAATTATTTAATTCCTTTAATATTGGGATCACCTGATATAGCTTTTCCTCG
TATAAATAATATTAGATTTTGATTACTCCCCCCATCATTAATAATACTACTATTAAGAAA
TTTATTTACACCAAATGTAGGAACAGGATGAACTGTTTATCCTCCATTATCATCTTACCT
TTTTCATTCATCACCATCAGTTGATATTGCAATTTTCTCTTTACATATAACAGGAATTTC
TTCCATTATTGGATCTTTAAATTTTATTGTAACAATTATATTTATAAAAAATTTTTCTTT
AAATTATGATCAAATTAACTTATTCTCTTGATCTGTTTGTATTACAGTAATTTTATTAAT
TCTATCCTTACCAGTTTTAGCGGGAGCTATTACTATACTTCTATTTGATCGA
>KR874159;tax=d:Eukarya,p:Arthropoda,c:Insecta,o:Hymenoptera,f:Apidae,g:Bombus,s:Bombus rufocinctus
AATAATATATTTTATTTTTGCTATATGATCAGGAATAATTGGTTCATCAATAAGATTATT
AATTCGAATAGAATTAAGACATCCTGGAATATGAATCAATAATGATCAAATTTATAATTC
ATTAGTAACTAGACATGCATTTTTAATAATTTTTTTTATAGTTATACCATTTATAATTGG
AGGTTTTGGAAATTATTTAATTCCTTTAATATTGGGATCACCTGATATAGCTTTTCCTCG
TATAAATAATATTAGATTTTGATTACTCCCTCCATCATTAATAATACTACTATTAAGAAA
TTTATTTACACCAAATGTAGGAACAGGATGAACTGTTTATCCTCCATTATCATCTTACCT
TTTTCATTCATCACCATCAGTTGATATTGCAATTTTCTCTTTACATATAACAGGAATTTC
TTCCATTATTGGATCTTTAAATTTTATTGTAACAATTATATTTATAAAAAATTTTTCTTT
AAATTATGATCAAATTAACTTATTCTCTTGATCTGTTTGTATTACAGTAATTTTATTAAT
TCTATCCTTACCAGTTTTAGCGGGAGCTATTACTATACTTCTATTTGATCGAAATTTTAA
TACATCTTTCTTTGATCCTATAGGAG
>KR874309;tax=d:Eukarya,p:Arthropoda,c:Insecta,o:Hymenoptera,f:Apidae,g:Bombus,s:Bombus rufocinctus
AATAATATATTTTATTTTTGCTATATGATCAGGAATAATTGGTTCATCAATAAGATTATT
AATTCGAATAGAATTAAGACATCCTGGAATATGAATCAATAATGATCAAATTTATAATTC
ATTAGTAACTAGACATGCATTTTTAATAATTTTTTTTATAGTTATACCATTTATAATTGG
AGGTTTTGGAAATTATTTAATTCCTTTAATATTGGGGTCACCTGATATAGCTTTTCCTCG
TATAAATAATATTAGATTTTGATTACTCCCTCCATCATTAATAATACTACTATTAAGAAA
TTTATTTACACCAAATGTAGGAACAGGATGAACTGTTTATCCTCCATTATCATCTTACCT
TTTTCATTCATCACCATCAGTTGATATTGCAATTTTCTCTTTACATATAACAGGAATTTC
TTCCATTATTGGATCTTTAAATTTTATTGTAACAATTATATTTATAAAAAATTTTTCTTT
AAATTATGATCAAATTAACTTATTCTCTTGATCTGTTTGTATTACAGTAATTTTATTAAT
TCTATCCTTACCAGTTTTAGCGGGAGCTATTACTATACTTCTATTTGATCGAAATTTTAA
TACATCTTTCTTTGATCCTATAGGAGGTGG
>KR874887;tax=d:Eukarya,p:Arthropoda,c:Insecta,o:Hymenoptera,f:Apidae,g:Bombus,s:Bombus rufocinctus
ATAATATATTTTATTTTTGCTATATGATCAGGAATAATTGGTTCATCAATAAGATTATTA
ATTCGAATAGAATTAAGACATCCTGGAATATGAATCAATAATGATCAAATTTATAATTCA
TTAGTAACTAGACATGCATTTTTAATAATTTTTTTTATAGTTATACCATTTATAATTGGA
GGTTTTGGAAATTATTTAATTCCTTTAATATTGGGGTCACCTGATATAGCTTTTCCTCGT
ATAAATAATATTAGATTTTGATTACTTCCTCCATCATTAATAATACTACTATTAAGAAAT
TTATTTACACCAAATGTAGGAACAGGATGAACTGTTTATCCTCCATTATCATCTTACCTT
TTTCATTCATCACCATCAGTTGATATTGCAATTTTCTCTTTACATATAACAGGAATTTCT
TCCATTATTGGATCTTTAAATTTTATTGTAACAATTATATTTATAAAAAATTTTTCTTTA
AATTATGATCAAATTAACTTATTTTCTTGATCTGTTTGTATTACAGTAATTTTATTAATT
CTATCCTTACCAGTTTTAGCGGGAGCTATTACTATACTTCTATTTGATCGA
>KR875836;tax=d:Eukarya,p:Arthropoda,c:Insecta,o:Hymenoptera,f:Apidae,g:Bombus,s:Bombus rufocinctus
ATAATATATTTTATTTTTGCTATATGATCAGGAATAATTGGTTCATCAATAAGATTATTA
ATTCGAATAGAATTAAGACATCCTGGAATATGAATCAATAATGATCAAATTTATAATTCA
TTAGTAACTAGACATGCATTTTTAATAATTTTTTTTATAGTTATACCATTTATAATTGGA

GGTTTTGGAAATTATTTAATTCCTTTAATATTAGGATCACCTGATATAGCTTTTCCTCGT
ATAAATAATATTAGATTTTGATTACTCCCTCCATCATTAATAATACTACTATTAAGAAAT
TTATTTACACCAAATGTAGGAACAGGATGAACTGTTTATCCTCCATTATCATCTTACCTT
TTTCATTCATCACCATCAGTTGATATTGCAATTTTCTCTTTACATATAACAGGAATTTCT
TCCATTATTGGATCTTTAAATTTTATTGTAACAATTATATTTATAAAAAATTTTTCTTTA
AATTATGATCAAATTAACTTATTCTCTTGATCTGTTTGTATTACAGTAATTTTATTAATT
CTATCC
>KR877422;tax=d:Eukarya,p:Arthropoda,c:Insecta,o:Hymenoptera,f:Apidae,g:Bombus,s:Bombus rufocinctus
AATAATATATTTTATTTTTGCTATATGATCAGGAATAATTGGTTCATCAATAAGATTATT
AATTCGAATAGAATTAAGACATCCTGGAATATGAATCAATAATGATCAAATTTATAATTC
ATTAGTAACTAGACATGCATTTTTAATAATTTTTTTTATAGTTATACCATTTATAATTGG
AGGTTTTGGAAATTATTTAATTCCTTTAATATTGGGGTCACCTGATATAGCTTTTCCTCG
TATAAATAATATTAGATTTTGATTACTCCCTCCATCATTAATAATACTACTATTAAGAAA
TTTATTTACACCAAATGTAGGAACAGGATGAACTGTTTATCCTCCATTATCATCTTACCT
TTTTCATTCATCACCATCAGTTGATATTGCAATTTTCTCTTTACATATAACAGGAATTTC
TTCCATTATTGGATCTTTAAATTTTATTGTAACAATTATATTTATAAAAAATTTTTCTTT
AAATTATGATCAAATTAACTTATTCTCTTGATCTGTTTGTATTACAGTAATTTTATTAAT
TCTATCCTTACCAGTTTTAGCGGGAGCTATTACTATACTTCTATTTGATCGA
>KR878815;tax=d:Eukarya,p:Arthropoda,c:Insecta,o:Hymenoptera,f:Apidae,g:Bombus,s:Bombus rufocinctus
AATAATATATTTTATTTTTGCTATATGATCAGGAATAATTGGTTCATCAATAAGATTATT
AATTCGAATAGAATTAAGACATCCTGGAATATGAATCAATAATGATCAAATTTATAATTC
ATTAGTAACTAGACATGCATTTTTAATAATTTTTTTTATAGTTATACCATTTATAATTGG
AGGTTTTGGAAATTATTTAATTCCTTTAATATTGGGATCACCTGATATAGCTTTTCCTCG
TATAAATAATATTAGATTTTGATTACTCCCTCCATCATTAATAATACTACTATTAAGAAA
TTTATTTACACCAAATGTAGGAACAGGATGAACTGTTTATCCTCCATTATCATCTTACCT
TTTTCATTCATCACCATCAGTTGATATTGCAATTTTCTCTTTACATATAACAGGAATTTC
TTCCATTATTGGATCTTTAAATTTTATTGTAACAATTATATTTATAAAAAATTTTTCTTT
AAATTATGATCAAATTAACTTATTCTCTTGATCTGTTTGTATTACAGTAATTTTATTAAT
TCTATCCTTACCAGTTTTAGCGGGAGCTATTACTATACT
>KR879238;tax=d:Eukarya,p:Arthropoda,c:Insecta,o:Hymenoptera,f:Apidae,g:Bombus,s:Bombus rufocinctus
AATAATATATTTTATTTTTGCTATATGATCAGGAATAATTGGTTCATCAATAAGATTATT
AATTCGAATAGAATTAAGACATCCTGGAATATGAATCAATAATGATCAAATTTATAATTC
ATTAGTAACTAGACATGCATTTTTAATAATTTTTTTTATAGTTATACCATTTATAATTGG
AGGTTTTGGAAATTATTTAATTCCTTTAATATTGGGATCACCTGATATAGCTTTTCCTCG
TATAAATAATATTAGATTTTGATTACTCCCTCCATCATTAATAATACTACTATTAAGAAA
TTTATTTACACCAAATGTAGGAACAGGATGAACTGTTTATCCTCCATTATCATCTTACCT
TTTTCATTCATCACCATCAGTTGATATTGCAATTTTCTCTTTACATATAACAGGAATTTC
TTCCATTATTGGATCTTTAAATTTTATTGTAACAATTATATTTATAAAAAATTTTTCTTT
AAATTATGATCAAATTAACTTATTCTCTTGATCTGTTTGTATTACAGTAATTTTATTAAT
TCTATCCTTACCAGTTTTAGCGGGAGCTATTACTA
>KR881702;tax=d:Eukarya,p:Arthropoda,c:Insecta,o:Hymenoptera,f:Apidae,g:Bombus,s:Bombus rufocinctus
ATAATATACTTTATTTTTGCTATATGATCAGGAATAATTGGTTCATCAATAAGATTATTA
ATTCGAATAGAATTAAGACATCCTGGAATATGAATCAATAATGATCAAATTTATAATTCA
TTAGTAACTAGACATGCATTTTTAATAATTTTTTTTATAGTTATACCATTTATAATTGGA
GGTTTTGGAAATTATTTAATTCCTTTAATATTGGGGTCACCTGATATAGCTTTTCCTCGT
ATAAATAATATTAGATTTTGATTACTCCCTCCATCATTAATAATACTACTATTAAGAAAT
TTATTTACACCAAATGTAGGAACAGGATGAACTGTTTATCCTCCATTATCATCTTACCTT
TTTCATTCATCACCATCAGTTGATATTGCAATTTTCTCTTTACATATAACAGGAATTTCT
TCCATTATTGGATCTTTAAATTTTATTGTAACAATTATATTTATAAAAAATTTTTCTTTA
AATTATGATCAAATTAACTTATTCTCTTGATCTGTTTGTATTACAGTAATTTTATTAATT
CTATCCTTACCAGTTTTA

>KR885510;tax=d:Eukarya,p:Arthropoda,c:Insecta,o:Hymenoptera,f:Apidae,g:Bombus,s:Bombus rufocinctus
AATAATATATTTTATTTTTGCTATATGATCAGGAATAATTGGTTCATCAATAAGATTATT
AATTCGAATAGAATTAAGACATCCTGGAATATGAATCAATAATGATCAAATTTATAATTC
ATTAGTAACTAGACATGCATTTTTAATAATTTTTTTTATAGTTATACCATTTATAATTGG
AGGTTTTGGAAATTATTTAATTCCTTTAATATTGGGATCACCTGATATAGCTTTTCCTCG
TATAAATAATATTAGATTTTGATTACTCCCTCCATCATTAATAATACTACTATTAAGAAA
TTTATTTACACCAAATGTAGGAACAGGATGAACTGTTTATCCTCCATTATCATCTTACCT
TTTTCATTCATCACCATCAGTTGATATTGCAATTTTCTCTTTACATATAACAGGAATTTC
TTCCATTATTGGATCTTTAAATTTTATTGTAACAATTATATTTATAAAAAATTTTTCTTT
AAATTATGATCAAATTAACTTATTCTCTTGATCTGTTTGTATTACAGTAATTTTATTAAT
TCTATCCTTACCAGTTTTAGCGGGAGCTATTACTATACTTCTATTTGATCGAAATTTTAA
TACATCTTTCTTTGATCCTATAGGAGGCGGTGATCCTATTCTTTATCAACACTTATTT
>KR885764;tax=d:Eukarya,p:Arthropoda,c:Insecta,o:Hymenoptera,f:Apidae,g:Bombus,s:Bombus rufocinctus
AATAATATATTTTATTTTTGCTATATGATCAGGAATAATTGGTTCATCAATAAGATTATT
AATTCGAATAGAATTAAGACATCCTGGAATATGAATCAATAATGATCAAATTTATAATTC
ATTAGTAACTAGACATGCATTTTTAATAATTTTTTTTATAGTTATACCATTTATAATTGG
AGGTTTTGGAAATTATTTAATTCCTTTAATATTAGGGTCACCTGATATAGCTTTTCCTCG
TATAAATAATATTAGATTTTGATTACTCCCTCCATCATTAATAATACTACTATTAAGAAA
TTTATTTACACCAAATGTAGGAACAGGATGAACTGTTTATCCTCCATTATCATCTTACCT
TTTTCATTCATCACCATCAGTTGATATTGCAATTTTCTCTTTACATATAACAGGAATTTC
TTCCATTATTGGATCTTTAAATTTTATTGTAACAATTATATTTATAAAAAATTTTTCTTT
AAATTATGATCAAATTAACTTATTCTCTTGATCTGTTTGTATTACAGTAATTTTATTAAT
TCTATCCTTACCAGTTTTAGCGGGAGCTATTACTATACTTCTATTTGATCGAAATTTTAA
TACATCTTTCTTTGATCCTATAGGAGG
>KR891128;tax=d:Eukarya,p:Arthropoda,c:Insecta,o:Hymenoptera,f:Apidae,g:Bombus,s:Bombus rufocinctus
ATAATATACTTTATTTTTGCTATATGATCAGGAATAATTGGTTCATCAATAAGATTATTA
ATTCGAATAGAATTAAGACATCCTGGAATATGAATCAATAATGATCAAATTTATAATTCA
TTAGTAACTAGACATGCATTTTTAATAATTTTTTTTATAGTTATACCATTTATAATTGGA
GGTTTTGGAAATTATTTAATTCCTTTAATATTGGGGTCACCTGATATAGCTTTTCCTCGT
ATAAATAATATTAGATTTTGATTACTCCCTCCATCATTAATAATACTACTATTAAGAAAT
TTATTTACACCAAATGTAGGAACAGGATGAACTGTTTATCCTCCATTATCATCTTACCTT
TTTCATTCATCACCATCAGTTGATATTGCAATTTTCTCTTTACATATAACAGGAATTTCT
TCCATTATTGGATCTTTAAATTTTATTGTAACAATTATATTTATAAAAAATTTTTCTTTA
AATTATGATCAAATTAACTTATTCTCTTGATCTGTTTGTATTACAGTAATTTTATTAATT
CTATCCTTACCAGTTTTAGCGGGA
>KR891913;tax=d:Eukarya,p:Arthropoda,c:Insecta,o:Hymenoptera,f:Apidae,g:Bombus,s:Bombus rufocinctus
ATAATATATTTTATTTTTGCTATATGATCAGGAATAATTGGTTCATCAATAAGATTATTA
ATTCGAATAGAATTAAGACATCCTGGAATATGAATCAATAATGATCAAATTTATAATTCA
TTAGTAACTAGACATGCATTTTTAATAATTTTTTTTATAGTTATACCATTTATAATTGGA
GGTTTTGGAAATTATTTAATTCCTTTAATATTAGGATCACCTGATATAGCTTTTCCTCGT
ATAAATAATATTAGATTTTGATTACTCCCTCCATCATTAATAATACTACTATTAAGAAAT
TTATTTACACCAAATGTAGGAACAGGATGAACTGTTTATCCTCCATTATCATCTTACCTT
TTTCATTCATCACCATCAGTTGATATTGCAATTTTCTCTTTACATATAACAGGAATTTCT
TCCATTATTGGATCTTTAAATTTTATTGTAACAATTATATTTATAAAAAATTTTTCTTTA
AATTATGATCAAATTAACTTATTCTCTTGATCTGTTTGTATTACAGTAATTTTATTAATT
CTATCCTTACCAGTTTTAGCGGGA
>KR892517;tax=d:Eukarya,p:Arthropoda,c:Insecta,o:Hymenoptera,f:Apidae,g:Bombus,s:Bombus rufocinctus
AATAATATATTTTATTTTTGCTATATGATCAGGAATAATTGGTTCATCAATAAGATTATT
AATTCGAATAGAATTAAGACATCCTGGAATATGAATCAATAATGATCAAATTTATAATTC
ATTAGTAACTAGACATGCATTTTTAATAATTTTTTTTATAGTTATACCATTTATAATTGG
AGGTTTTGGAAATTATTTAATTCCTTTAATATTGGGATCACCTGATATAGCTTTTCCTCG

TATAAATAATATTAGATTTTGATTACTCCCTCCATCATTAATAATACTACTATTAAGAAA
TTTATTTACACCAAATGTAGGAACAGGATGAACTGTTTATCCTCCATTATCATCTTACCT
TTTTCATTCATCACCATCAGTTGATATTGCAATTTTCTCTTTACATATAACAGGAATTTC
TTCCATTATTGGATCTTTAAATTTTATTGTAACAATTATATTTATAAAAAATTTTTCTTT
AAATTATGATCAAATTAACTTATTCTCTTGATCTGTTTGTATTACAGTAATTTTATTAAT
TCTATCCTTACCAGTTTTAGCGGGAGCTATTACTATACTTCTATTTGATCGAAATTTTAA
TACATCTTTCTTTGATCCTATAGGAGGTGGTG
>KR898293;tax=d:Eukarya,p:Arthropoda,c:Insecta,o:Hymenoptera,f:Apidae,g:Bombus,s:Bombus rufocinctus
AATAATATATTTTATTTTTGCTATATGATCAGGAATAATTGGTTCATCAATAAGATTATT
AATTCGAATAGAATTAAGACATCCTGGAATATGAATCAATAATGATCAAATTTATAATTC
ATTAGTAACTAGACATGCATTTTTAATAATTTTTTTTATAGTTATACCATTTATAATTGG
AGGTTTTGGAAATTATTTAATTCCTTTAATATTGGGATCACCTGATATAGCTTTTCCTCG
TATAAATAATATTAGATTTTGATTACTCCCTCCATCATTAATAATACTACTATTAAGAAA
TTTATTTACACCAAATGTAGGAACAGGATGAACTGTTTATCCTCCATTATCATCTTACCT
TTTTCATTCATCACCATCAGTTGATATTGCAATTTTCTCTTTACATATAACAGGAATTTC
TTCCATTATTGGATCTTTAAATTTTATTGTAACAATTATATTTATAAAAAATTTTTCTTT
AAATTATGATCAAATTAACTTATTCTCTTGATCTGTTTGTATTACAGTAATTTTATTAAT
TCTATCCTTACCAGTTTTAGCGGGAGCTATTACTATACTTCTATTTGATCGAAATTTTAA
TACATCTTTCTTTGATCC
>KR900201;tax=d:Eukarya,p:Arthropoda,c:Insecta,o:Hymenoptera,f:Apidae,g:Bombus,s:Bombus rufocinctus
AATAATATATTTTATTTTTGCTATATGATCAGGAATAATTGGTTCATCAATAAGATTATT
AATTCGAATAGAATTAAGACATCCTGGAATATGAATCAATAATGATCAAATTTATAATTC
ATTAGTAACTAGACATGCATTTTTAATAATTTTTTTTATAGTTATACCATTTATAATTGG
AGGTTTTGGAAATTATTTAATTCCTTTAATATTGGGATCACCTGATATAGCTTTTCCTCG
TATAAATAATATTAGATTTTGATTACTCCCTCCATCATTAATAATACTACTATTAAGAAA
TTTATTTACACCAAATGTAGGAACAGGATGAACTGTTTATCCTCCATTATCATCTTACCT
TTTTCATTCATCACCATCAGTTGATATTGCAATTTTCTCTTTACATATAACAGGAATTTC
TTCCATTATTGGATCTTTAAATTTTATTGTAACAATTATATTTATAAAAAATTTTTCTTT
AAATTATGATCAAATTAACTTATTCTCTTGATCTGTTTGTATTACAGTAATTTTATTAAT
TCTATCCTTACCAGTTTTAGCGGGAGCTATTACTATACTTCTATTTGATCGAAATTTTAA
TACATCTTTCTTTGATCCTATAGGAGG
>KR900226;tax=d:Eukarya,p:Arthropoda,c:Insecta,o:Hymenoptera,f:Apidae,g:Bombus,s:Bombus rufocinctus
AATAATATATTTTATTTTTGCTATATGATCAGGAATAATTGGTTCATCAATAAGATTATT
AATTCGAATAGAATTAAGACATCCTGGAATATGAATCAATAATGATCAAATTTATAATTC
ATTAGTAACTAGACATGCATTTTTAATAATTTTTTTTATAGTTATACCATTTATAATTGG
AGGTTTTGGAAATTATTTAATTCCTTTAATATTGGGGTCACCTGATATAGCTTTTCCTCG
TATAAATAATATTAGATTTTGATTACTCCCTCCATCATTAATAATACTACTATTAAGAAA
TTTATTTACACCAAATGTAGGAACAGGATGAACTGTTTATCCTCCATTATCATCTTACCT
TTTTCATTCATCACCATCAGTTGATATTGCAATTTTCTCTTTACATATAACAGGAATTTC
TTCCATTATTGGATCTTTAAATTTTATTGTAACAATTATATTTATAAAAAATTTTTCTTT
AAATTATGATCAAATTAACCTATTCTCTTGATCTGTTTGTATTACAGTAATTTTATTAAT
TTTATCCTTACCAGTTTTAGCGGGAGCTATTACTATACTTCTATTTGATCGAAATTTTAA
TACATCTTTCTTTGATCCTATAGGAGG
>KR900889;tax=d:Eukarya,p:Arthropoda,c:Insecta,o:Hymenoptera,f:Apidae,g:Bombus,s:Bombus rufocinctus
AATAATATATTTTATTTTTGCTATATGATCAGGAATAATTGGTTCATCAATAAGATTATT
AATTCGAATAGAATTAAGACATCCTGGAATATGAATCAATAATGATCAAATTTATAATTC
ATTAGTAACTAGACATGCATTTTTAATAATTTTTTTTATAGTTATACCATTTATAATTGG
AGGTTTTGGAAATTATTTAATTCCTTTAATATTGGGGTCACCTGATATAGCTTTTCCTCG
TATAAATAATATTAGATTTTGATTACTCCCTCCATCATTAATAATACTACTATTAAGAAA
TTTATTTACACCAAATGTAGGAACAGGATGAACTGTTTATCCCCCATTATCATCTTACCT
TTTTCATTCATCACCATCAGTTGATATTGCAATTTTCTCTTTACATATAACAGGAATTTC

TTCCATTATTGGATCTTTAAATTTTATTGTAACAATTATATTTATAAAAAATTTTTCTTT
AAATTATGATCAAATTAACTTATTCTCTTGATCTGTTTGTATTACAGTAATTTTATTAAT
TCTATCCTTACCAGTTTTAGCGGGAGCTATTACTATACTTT
>KT706869;tax=d:Eukarya,p:Arthropoda,c:Insecta,o:Hymenoptera,f:Apidae,g:Bombus,s:Bombus rufocinctus
AATAATATATTTTATTTTTGCTATATGATCAGGAATAATTGGTTCATCAATAAGATTATT
AATTCGAATAGAATTAAGACATCCTGGAATATGAATCAATAATGATCAAATTTATAATTC
ATTAGTAACTAGACATGCATTTTTAATAATTTTTTTTATAGTTATACCATTTATAATTGG
AGGTTTTGGAAATTATTTAATTCCTTTAATATTGGGGTCACCTGATATAGCTTTTCCTCG
TATAAATAATATTAGATTTTGATTACTTCCTCCATCATTAATAATACTACTATTAAGAAA
TTTATTTACACCAAATGTAGGAACAGGATGAACTGTTTATCCTCCATTATCATCTTACCT
TTTTCATTCATCACCATCAGTTGATATTGCAATTTTCTCTTTACATATAACAGGAATTTC
TTCCATTATTGGATCTTTAAATTTTATTGTAACAATTATATTTATAAAAAATTTTTCTTT
AAATTATGATCAAATTAACTTATTTTCTTGATCTGTTTGTATTACAGTAATTTTATTAAT
TCTATCCTTACCAGTTTTAGCGGGAGCTATTACTATACTTCTATTTGATCGAAATTTTAA
TACATCTTTCTTTGATCCTATAGGAGG
>MF933805;tax=d:Eukarya,p:Arthropoda,c:Insecta,o:Hymenoptera,f:Apidae,g:Bombus,s:Bombus rufocinctus
ATAATATATTTTATTTTTGCTATATGATCAGGAATAATTGGTTCATCAATAAGATTATTA
ATTCGAATAGAATTAAGACATCCTGGAATATGAATCAATAATGATCAAATTTATAATTCA
TTAGTAACTAGACATGCATTTTTAATAATTTTTTTTATAGTTATACCATTTATAATTGGA
GGTTTTGGAAATTATTTAATTCCTTTAATATTGGGATCACCTGATATAGCTTTTCCTCGT
ATAAATAATATTAGATTTTGATTACTCCCTCCATCATTAATAATACTACTATTAAGAAAT
TTATTTACACCAAATGTAGGAACAGGATGAACTGTTTATCCTCCATTATCATCTTACCTT
TTTCATTCATCACCATCAGTTGATATTGCAATTTTCTCTTTACATATAACAGGAATTTCT
TCCATTATTGGATCTTTAAATTTTATTGTAACAATTATATTTATAAAAAATTTTTCTTTA
AATTATGATCAAATTAACTTATTCTCTTGATCTGTTTGTATTACAGTAATTTTATTAATT
CTATCCTTACCAGTTTTAGCGGGAGCTATTACTATACTTCTATTTGATCGA
>MF936976;tax=d:Eukarya,p:Arthropoda,c:Insecta,o:Hymenoptera,f:Apidae,g:Bombus,s:Bombus rufocinctus
ATAATATATTTTATTTTTGCTATATGATCAGGAATAATTGGTTCATCAATAAGATTATTA
ATTCGAATAGAATTAAGACATCCTGGAATATGAATCAATAATGATCAAATTTATAATTCA
TTAGTAACTAGACATGCATTTTTAATAATTTTTTTTATAGTTATACCATTTATAATTGGA
GGTTTTGGAAATTATTTAATTCCTTTAATATTGGGGTCACCTGATATAGCTTTTCCTCGT
ATAAATAATATTAGATTTTGATTACTCCCTCCATCATTAATAATACTACTATTAAGAAAT
TTATTTACACCAAATGTAGGAACAGGATGAACTGTTTATCCTCCATTATCATCTTACCTT
TTTCATTCATCACCATCAGTTGATATTGCAATTTTCTCTTTACATATAACAGGAATTTCT
TCCATTATTGGATCTTTAAATTTTATTGTAACAATTATATTTATAAAAAATTTTTCTTTA
AATTATGATCAAATTAACTTATTCTCTTGATCTGTTTGTATTACAGTAATTTTATTAATT
CTATCCTTACCAGTTTTAGCGGGAGCTATTACTATACTTCTATTTGAT
>MF937556;tax=d:Eukarya,p:Arthropoda,c:Insecta,o:Hymenoptera,f:Apidae,g:Bombus,s:Bombus rufocinctus
ATAATATATTTTATTTTTGCTATATGATCAGGAATAATTGGTTCATCAATAAGATTATTA
ATTCGAATAGAATTAAGACATCCTGGAATATGAATCAATAATGATCAAATTTATAATTCA
TTAGTAACTAGACATGCATTTTTAATAATTTTTTTTATAGTTATACCATTTATAATTGGA
GGTTTTGGAAATTATTTAATTCCTTTAATATTGGGGTCACCTGATATAGCTTTTCCTCGT
ATAAATAATATTAGATTTTGATTACTCCCTCCATCATTAATAATACTACTATTAAGAAAT
TTATTTACACCAAATGTAGGAACAGGATGAACTGTTTATCCTCCATTATCATCTTACCTT
TTTCATTCATCACCATCAGTTGATATTGCAATTTTCTCTTTACATATAACAGGAATTTCT
TCCATTATTGGATCTTTAAATTTTATTGTAACAATTATATTTATAAAAAATTTTTCTTTA
AATTATGATCAAATTAACTTATTCTCTTGATCTGTTTGTATTACAGTAATTTTATTAATT
CTATCCTTACCAGTTTTAGCGGGAGCTATCACTATACTTCTATTTGATCGA
>JN293817;tax=d:Eukarya,p:Arthropoda,c:Insecta,o:Hymenoptera,f:Colletidae,g:Colletes,s:Colletes hyalinus
AATATTATATTTTATTTTTGCTATATGAACTGGAATAATTGGTTCTTCATTAAGAATAAT
TATTCGTATAGAATTAAGTTCACCTGGTATATGAATTAATAATGATCAAATTTATAATTC

TATTGTTACTGCTCATGCTTTTATTATAATTTTTTTTATAGTAATACCTTTTTTAATTGG
AGGTTTTGGAAATTGATTAATTCCATTAATAATTGGAGCTCCTGATATAGCATTTCCTCG
AATGAATAATATAAGATTTTGGTTATTACCACCTTCATTAATTTTATTATTAATAAGAAG
AATTTTATATTCTGGAAGAGGAACAGGATGAACTATTTATCCTCCTTTATCATCTTTAAT
ATATCATTCTTCTTTATCTGTTGATTTAACAATTTTTTCTTTACATATTGCAGGTATTTC
ATCTATTATAGGATCTATAAATTTTATTGTTACTATTTTAAGTATAAAAAATTGTAATTT
AAACTATGATCAATTATCATTATTTTCTTGATCTGTTTTTATTACAACAATTTTATTATT
ATTATCTTTACCTGTATTAGCTGGAGCTATTACTATATTATTAACTGATCGAAATTTAAA
TACTTCTTTTTTTGATCCATCTGGTGGAGGAGATCCAATTCTTTATCAACATTTATTT
>MF906152;tax=d:Eukarya,p:Arthropoda,c:Insecta,o:Hymenoptera,f:Colletidae,g:Colletes,s:Colletes hyalinus
ATATTATATTTTATTTTTGCTATATGAACTGGAATAATTGGTTCTTCATTAAGAATAATT
ATTCGTATAGAATTAAGTTCACCTGGTATATGAATTAATAATGATCAAATTTATAATTCT
ATTGTTACTGCTCATGCTTTTATTATAATTTTTTTTATAGTAATACCTTTTTTAATTGGA
GGTTTTGGAAATTGATTAATTCCATTAATAATTGGAGCTCCTGATATAGCATTTCCTCGA
ATGAATAATATAAGATTTTGGTTATTACCACCTTCATTAATTTTATTATTAATAAGAAGA
ATTTTATATTCTGGAAGAGGAACAGGATGAACTATTTATCCTCCTTTATCATCTTTAATA
TATCATTCTTCTTTATCTGTTGATTTAACAATTTTTTCTTTACATATTGCAGGTATTTCA
TCTATTATAGGATCTATAAATTTTATTGTTACTATTTTAAGTATAAAAAATTGTAATTTA
AACTATGATCAATTATCATTATTTTCTTGATCTGTTTTTATTACAACAATTTTATTATTA
TTATCTTTACCTGTATTAGCTGGAGCTATTACTATATTATTA
>JN293763;tax=d:Eukarya,p:Arthropoda,c:Insecta,o:Hymenoptera,f:Colletidae,g:Colletes,s:Colletes kincaidii
TGTATTATATTTTATTTTTGCTATATGAACCGGGATGATTGGTTCATCTTTAAGAATAAT
TATTCGTATAGAATTAAGTTCTCCCGGTATATGAATTAATAATGATCAAATTTATAATTC
TATTGTTACTGCACATGCTTTTATTATAATTTTTTTTATAGTTATACCTTTTTTAATTGG
TGGATTTGGTAATTGATTAATTCCATTAATAATTGGAGCACCTGATATAGCATTTCCTCG
AATAAATAATATGAGTTTTTGATTGTTACCTCCTTCTTTATTTCTTTTATTATTAAATAG
AATTTTATATTCAGGTAGAGGAACAGGATGAACCATTTATCCTCCTTTGTCTTCTTTAAT
ATATCATCCTTCTTTATCTGTTGATTTAACAATTTTTTCTTTACATATTGCTGGTATTTC
ATCAATTATAGGATCAATAAATTTTATTGTTACAATTTTAATAATAAAAAATTATAATTT
AAATTATGATCAATTATCATTATTTTCGTGATCTGTTTTTATTACAACAATCTTATTATT
ATTATCTTTACCTGTATTAGCTGGAGCAATTACAATATTATTAACTGATCGTAATTTAAA
TACCTCATTTTTTGATCCATCTGGTGGAGGTGATCCTGTTCTTTATCAACATTTATTT
>MN342297;tax=d:Eukarya,p:Arthropoda,c:Insecta,o:Hymenoptera,f:Colletidae,g:Colletes,s:Colletes kincaidii
TGTATTATATTTTATTTTTGCTATATGAACCGGGATGATTGGTTCATCTTTAAGAATAAT
TATTCGTATAGAATTAAGTTCTCCCGGTATATGAATTAATAATGATCAAATTTATAATTC
TATTGTTACTGCACATGCTTTTATTATAATTTTTTTTATAGTTATACCTTTTTTAATTGG
TGGATTTGGTAATTGATTAATTCCATTAATAATTGGAGCACCTGATATAGCATTTCCTCG
AATAAATAATATGAGTTTTTGATTGTTACCTCCTTCTTTATTTCTTTTATTATTAAATAG
AATTTTATATTCAGGTAGAGGAACAGGATGAACCATTTATCCTCCTTTGTCTTCTTTAAT
ATATCATCCTTCTTTATCTGTTGATTTAACAATTTTTTCTTTACATATTGCTGGTATTTC
ATCAATTATAGGATCAATAAATTTTATTGTTACAATTTTAATAATAAAAAATTATAATTT
AAATTATGATCAATTATCATTATTTTCGTGATCTGTTTTTATTACAACAATTTTATTATT
ATTATCTTTACCTGTATTAGCTGGAGCAATTACAATATTATTAACTGATCGTAATTTAAA
TACCTCATTTTTTGATCCATCTGGTGGAGGTGATCCTGTTCTTTATCAACATTTATTT
>FJ582169;tax=d:Eukarya,p:Arthropoda,c:Insecta,o:Hymenoptera,f:Apidae,g:Ceratina,s:Ceratina dupla
AATTTTATATATTATATTTGCTATATGATCAGGTATAATCGGAGCATCAATAAGATTAAT
TATTCGAATAGAATTAAGAACCCCGGGAAATTGAATTAATAATGATCAAATTTATAATTC
ATTAGTTACTGCTCATGCCTTTTTAATAATTTTTTTTATAGTTATACCATTTATAATTGG
AGGATTTGGAAATTGATTAATTCCACTAATATTAGGATCACCAGATATATCTTTTCCTCG
TTTAAATAATATTAGTTTTTGATTATTACCCCCATCATTATTACTATTATTATTAAGAAA
TTTATTTTCAATAAGACCAGGAACAGGGTGAACTGTTTATCCACCTTTATCATCATATTT

ATTTCACCCATCTCCATCAGTAGATTTAGCTATTTTTTCCTTACATATATCAGGAATTTC
ATCAATTTTAGGAGCTATTAATTTTATAGTTACTATTATAATAATAAAAAATATTTCATT
AAATTATGATTCTATCCCATTATTTTCATGAGCAGTATTTATTACAGCAATTTTATTACT
TTTATCATTACCAGTATTAGCAGGAGCTATTACTATATTATTATTTGATCGAAATTTAAA
TACATCCTTTTTTGATCCTATAGGAGGTGGAGATCCAATTTTATACCAACATTTATTT
>FJ582170;tax=d:Eukarya,p:Arthropoda,c:Insecta,o:Hymenoptera,f:Apidae,g:Ceratina,s:Ceratina dupla
AATTTTATATATTATATTTGCTATATGATCAGGTATAATCGGAGCATCAATAAGATTAAT
TATTCGAATAGAATTAAGAACCCCGGGAAATTGAATTAATAATGATCAAATTTATAATTC
ATTAGTTACTGCTCATGCCTTTTTAATAATTTTTTTTATAGTTATACCATTTATAATTGG
AGGATTTGGAAATTGATTAATTCCACTAATATTAGGATCACCAGATATATCTTTTCCTCG
TTTAAATAATATTAGTTTTTGATTATTACCCCCATCATTATTACTATTATTATTAAGAAA
TTTATTTTCAATAAGACCAGGAACAGGGTGAACTGTTTATCCGCCTTTATCATCATATTT
ATTTCACCCATCTCCATCAGTAGATTTAGCTATTTTTTCCTTACATATATCAGGAATTTC
ATCAATTTTAGGAGCTATTAATTTTATAGTTACTATTATAATAATAAAAAATATTTCATT
AAATTATGATTCTATCCCATTATTTTCATGAGCAGTATTTATTACAGCAATTTTATTACT
TTTATCATTACCAGTATTAGCAGGAGCTATTACTATATTATTATTTGATCGAAATTTAAA
TACATCCTTTTTTGATCCTATAGGAGGTGGAGATCCAATTTTATACCAACATTTATTT
>FJ582173;tax=d:Eukarya,p:Arthropoda,c:Insecta,o:Hymenoptera,f:Apidae,g:Ceratina,s:Ceratina dupla
ATCAGGTATAATCGGAGCATCAATAAGATTAATTATTCGAATAGAATTAAGAACCCCGGG
AAATTGAATTAATAATGATCAAATTTATAATTCATTAGTTACTGCTCATGCCTTTTTAAT
AATTTTTTTTATAGTTATACCATTTATAATTGGAGGATTTGGAAATTGATTAATTCCACT
AATATTAGGATCACCAGATATATCTTTTCCTCGTTTAAATAATATTAGTTTTTGATTATT
ACCCCCATCATTATTACTATTATTATTAAGAAATTTATTTTCAATAAGACCAGGAACAGG
GTGAACTGTTTATCCACCTTTATCATCATATTTATTTCACCCATCTCCATCAGTAGATTT
AGCTATTTTTTCCTTACATATATCAGGAATTTCATCAATTTTAGGAGCTATTAATTTTAT
AGTTACTATTATAATAATAAAAAATATTTCATTAAATTATGATTCTATCCCATTATTTTC
ATGAGCAGTATTTATTACAGCAATTTTATTACTTTTATCATTACCAGTATTAGCAGGAGC
TATTACTATATTATTATTTGATCGAAATTTAAATACATCCTTTTTTGATCCTATAGGAGG
AGGAGATCCAATTTTATACCAACATTTATTT
>FJ582174;tax=d:Eukarya,p:Arthropoda,c:Insecta,o:Hymenoptera,f:Apidae,g:Ceratina,s:Ceratina dupla
GCTATATGATCAGGTATAATCGGAGCATCAATAAGATTAATTATTCGAATAGAATTAAGA
ACCCCGGGAAATTGAATTAATAATGATCAAATTTATAATTCATTAGTTACTGCTCATGCC
TTTTTAATAATTTTTTTTATAGTTATACCATTTATAATTGGAGGATTTGGAAATTGATTA
ATTCCACTAATATTAGGATCACCAGATATATCTTTTCCTCGTTTAAATAATATTAGTTTT
TGATTATTACCCCCATCATTATTACTATTATTATTAAGAAATTTATTTTCAATAAGACCA
GGAACAGGGTGAACTGTTTATCCACCTTTATCATCATATTTATTTCACCCATCTCCATCA
GTAGATTTAGCTATTTTTTCCTTACATATATCAGGAATTTCATCAATTTTAGGAGCTATT
AATTTTATAGTTACTATTATAATAATAAAAAATATTTCATTAAATTATGATTCTATCCCA
TTATTTTCATGAGCAGTATTTATTACAGCAATTTTATTACTTTTATCATTACCAGTATTA
GCAGGAGCTATTACTATATTATTATTTGATCGAAATTTAAATACATCCTTTTTTGATCCT
ATAGGAGGTGGAGATCCAATTTTATACCAACATTTATTT
>GU707469;tax=d:Eukarya,p:Arthropoda,c:Insecta,o:Hymenoptera,f:Apidae,g:Ceratina,s:Ceratina dupla
AATTTTATATATTATATTTGCTATATGATCAGGTATAATCGGAGCATCAATAAGATTAAT
TATTCGAATAGAATTAAGAACCCCAGGAAATTGAATTAATAATGATCAAATTTATAATTC
ATTAGTTACGGCTCATGCCTTTTTAATAATTTTTTTTATAGTTATACCATTTATAATTGG
GGGATTTGGAAATTGATTAATTCCACTAATATTAGGGTCACCAGATATATCTTTTCCTCG
TTTAAATAATATTAGTTTTTGATTATTACCCCCATCATTGCTACTATTATTATTAAGAAA
TTTATTTTCAATAAGACCAGGAACAGGATGAACTGTTTATCCACCTTTATCATCGTATTT
ATTCCACCCATCCCCATCAGTAGATTTAGCTATTTTTTCCTTACATATATCAGGAATTTC
ATCAATTTTAGGAGCTATTAATTTTATAGTTACTATTATAATAATAAAAAATATTTCATT
AAATTATGACTCTATCCCATTATTTTCATGAGCAGTATTTATTACAGCAATTTTATTACT

TTTATCATTACCAGTATTAGCAGGAGCTATTACTATATTATTATTTGATCGAAATTTAAA
TACATCCTTTTTTGATCCTATAGGAGGTGGAGATCCAATTTTATATCAACATTTATTT
>GU707470;tax=d:Eukarya,p:Arthropoda,c:Insecta,o:Hymenoptera,f:Apidae,g:Ceratina,s:Ceratina dupla
AATTTTATATATTATATTTGCTATATGATCAGGTATAATCGGAGCATCAATAAGATTAAT
TATTCGAATAGAATTAAGAACCCCAGGAAATTGAATTAATAATGATCAAATTTATAATTC
ATTAGTTACGGCTCATGCCTTTTTAATAATTTTTTTTATAGTTATACCATTTATAATTGG
GGGATTTGGAAATTGATTAATTCCACTAATATTAGGATCACCAGATATATCTTTTCCTCG
TTTAAATAATATTAGTTTTTGATTATTACCCCCATCATTGCTACTATTATTATTAAGAAA
TTTATTTTCAATAAGACCAGGAACAGGATGAACTGTTTATCCACCTTTATCATCGTATTT
ATTCCACCCATCCCCATCAGTAGATTTAGCTATTTTTTCCTTACATATATCAGGAATTTC
ATCAATTTTAGGAGCTATTAATTTTATAGTTACTATTATAATAATAAAAAATATTTCATT
AAATTATGACTCTATCCCATTATTTTCATGAGCAGTATTTATTACAGCAATTTTATTACT
TTTATCATTACCAGTATTAGCAGGAGCTATTACTATATTATTATTTGATCGAAATTTAAA
TACATCCTTTTTTGATCCTATAGGAGGTGGAGATCCAATTTTATATCAACATTTATTT
>GU707471;tax=d:Eukarya,p:Arthropoda,c:Insecta,o:Hymenoptera,f:Apidae,g:Ceratina,s:Ceratina dupla
AATTTTATATATTATATTTGCTATATGATCAGGTATAATCGGAGCATCAATAAGATTAAT
TATTCGAATAGAATTAAGAACCCCAGGAAATTGAATTAATAATGATCAAATTTATAATTC
ATTAGTTACTGCTCATGCCTTTTTAATAATTTTTTTTATAGTTATACCATTTATAATTGG
AGGATTTGGAAATTGATTAATTCCATTAATATTGGGATCACCAGATATATCTTTTCCTCG
TTTAAATAATATTAGTTTTTGATTATTACCCCCATCATTACTACTATTATTATTAAGAAA
TTTATTTTCAATAAGACCGGGAACAGGATGAACTGTTTATCCACCTTTATCATCATATTT
ATTTCACCCATCTCCGTCAGTAGACTTAGCTATTTTTTCCTTACATATGTCAGGAATTTC
ATCAATTTTAGGAGCTATTAATTTTATAGTTACTATTATAATAATAAAAAATATTTCATT
AAATTATGATTCTATCCCATTATTTTCATGAGCAGTATTTATTACAGCAATCTTATTACT
TTTATCATTACCAGTATTAGCAGGAGCTATTACTATATTATTATTTGATCGAAATTTAAA
TACATCCTTTTTTGATCCTATAGGAGGTGGAGATCCAATTTTATATCAACATTTATTT
>GU707473;tax=d:Eukarya,p:Arthropoda,c:Insecta,o:Hymenoptera,f:Apidae,g:Ceratina,s:Ceratina dupla
AATTTTATATATTATATTTGCTATATGATCAGGTATAATCGGAGCATCAATAAGATTAAT
TATTCGAATAGAATTAAGAACCCCAGGAAATTGAATTAATAATGATCAAATTTATAATTC
ATTAGTTACTGCTCATGCCTTTTTAATAATTTTTTTTATAGTTATACCATTTATAATTGG
AGGATTTGGAAATTGATTAATTCCATTAATATTGGGATCACCAGATATATCTTTTCCTCG
TTTAAATAATATTAGTTTTTGATTATTACCCCCATCATTACTACTATTATTATTAAGAAA
TTTATTTTCAATAAGACCGGGAACAGGATGAACTGTTTATCCACCTTTATCATCATATTT
ATTTCACCCATCTCCGTCAGTAGACTTAGCTATTTTTTCCTTACATATGTCAGGAATTTC
ATCAATTTTAGGAGCTATTAATTTTATAGTTACTATTATAATAATAAAAAATATTTCATT
AAATTATGATTCTATCCCATTATTTTCATGAGCAGTATTTATTACAGCAATTTTATTACT
TTTATCATTACCAGTATTAGCAGGAGCTATTACTATATTATTATTTGATCGAAATTTAAA
TACATCCTTTTTTGATCCTATAGGAGGTGGAGATCCAATTTTATATCAACATTTATTT
>GU707474;tax=d:Eukarya,p:Arthropoda,c:Insecta,o:Hymenoptera,f:Apidae,g:Ceratina,s:Ceratina dupla
AATTTTATATATTATATTTGCTATATGATCAGGAATAATTGGGGCATCAATAAGATTAAT
TATTCGAATAGAATTAAGAACCCCAGGAAATTGAATTAATAATGATCAAATTTATAATTC
ATTAGTTACTGCTCATGCCTTTTTAATAATTTTTTTTATAGTTATACCATTTATAATTGG
AGGATTTGGAAATTGATTAATTCCACTAATATTAGGATCACCAGATATATCTTTTCCTCG
TTTAAATAATATTAGTTTTTGATTATTACCCCCATCATTACTACTATTATTATTAAGAAA
TTTATTTTCAATAAGACCAGGAACAGGATGAACTGTTTATCCACCTTTATCATCATATTT
ATTTCACCCATCTCCATCAGTAGATTTAGCTATTTTTTCCTTACATATATCAGGAATTTC
ATCAATTTTAGGAGCTATTAATTTTATAGTCACTATTATAATAATAAAAAATATTTCATT
AAATTATGACTCTATCCCATTATTTTCATGAGCAGTATTTATTACAGCAATTTTATTACT
TTTATCATTACCAGTATTAGCAGGAGCTATTACTATATTATTATTTGATCGAAATTTAAA
TACATCCTTTTTTGATCCTATAGGAGGTGGAGATCCAATTTTATACCAACATTTATTT
>GU707475;tax=d:Eukarya,p:Arthropoda,c:Insecta,o:Hymenoptera,f:Apidae,g:Ceratina,s:Ceratina dupla

AATTTTATATATTATATTTGCCATATGATCAGGAATAATTGGAGCATCAATAAGATTAAT
TATTCGAATAGAATTAAGAACCCCAGGAAATTGAATTAATAATGATCAAATTTATAATTC
ATTAGTTACTGCTCATGCCTTTTTAATAATTTTTTTTATAGTTATACCATTTATAATTGG
AGGATTTGGAAATTGATTAATTCCACTAATATTAGGATCACCAGATATATCTTTTCCTCG
TTTAAATAATATTAGTTTTTGATTATTACCCCCATCATTACTACTATTATTATTAAGAAA
TTTATTTTCAATAAGACCAGGAACAGGATGAACTGTTTATCCACCTTTATCATCATATTT
ATTTCACCCATCTCCATCAGTAGATTTAGCTATTTTTTCCTTACATATATCAGGAATTTC
ATCAATTTTAGGAGCTATTAATTTTATAGTCACTATTATAATAATAAAAAATATTTCATT
AAATTATGACTCTATCCCATTATTTTCATGAGCAGTATTTATTACAGCAATTTTATTACT
TTTATCATTACCAGTATTAGCAGGAGCTATTACTATATTATTATTTGATCGAAATTTAAA
TACATCCTTTTTTGATCCTATAGGAGGTGGAGATCCAATTTTATATCAACATTTATTT
>GU707478;tax=d:Eukarya,p:Arthropoda,c:Insecta,o:Hymenoptera,f:Apidae,g:Ceratina,s:Ceratina dupla
AATTTTATATATTATATTTGCTATATGATCAGGTATAATCGGAGCATCAATAAGATTAAT
TATTCGAATAGAATTAAGAACCCCAGGAAATTGAATTAATAATGATCAAATTTATAATTC
ATTAGTTACTGCTCATGCCTTTTTAATAATTTTTTTTATAGTTATACCATTTATAATTGG
AGGATTTGGAAATTGATTAATTCCATTAATATTGGGATCACCAGATATATCTTTTCCTCG
TTTAAATAATATTAGTTTTTGATTATTACCCCCATCATTACTACTATTATTATTAAGAAA
TTTATTTTCAATAAGACCAGGAACAGGATGAACTGTTTATCCACCTTTATCATCATATTT
ATTTCACCCATCTCCGTCAGTAGACTTAGCTATTTTTTCCTTACATATGTCAGGAATTTC
ATCAATTTTAGGAGCTATTAATTTTATAGTTACTATTATAATAATAAAAAATATTTCATT
AAATTATGATTCTATCCCATTATTTTCATGAGCAGTATTTATTACAGCAATTTTATTACT
TTTATCATTACCAGTATTAGCAGGAGCTATTACTATATTATTATTTGATCGAAATTTAAA
TACATCCTTTTTTGATCCTATAGGAGGTGGAGATCCAATTTTATATCAACATTTATTT
>GU707479;tax=d:Eukarya,p:Arthropoda,c:Insecta,o:Hymenoptera,f:Apidae,g:Ceratina,s:Ceratina dupla
AATTTTATATATTATATTTGCTATATGATCAGGTATAATCGGAGCATCAATAAGATTAAT
TATTCGAATAGAATTAAGAACCCCAGGAAATTGAATTAATAATGATCAAATTTATAATTC
ATTAGTTACTGCTCATGCCTTTTTAATAATTTTTTTTATAGTTATACCATTTATAATTGG
AGGATTTGGAAATTGATTAATTCCATTAATATTGGGATCACCAGATATATCTTTTCCTCG
TTTAAATAATATTAGTTTTTGATTATTACCCCCATCATTACTACTATTATTATTAAGAAA
TTTATTTTCAATAAGACCGGGAACAGGATGAACTGTTTATCCACCTTTATCATCATATTT
ATTTCACCCATCTCCGTCAGTAGATTTAGCTATTTTTTCCTTACATATGTCAGGAATTTC
ATCAATTTTAGGAGCTATTAATTTTATAGTTACTATTATAATAATAAAAAATATTTCATT
AAATTATGATTCTATCCCATTATTTTCATGAGCAGTATTTATTACAGCAATTTTATTACT
TTTATCATTACCAGTATTAGCAGGAGCTATTACTATATTATTATTTGATCGAAATTTAAA
TACATCCTTTTTTGATCCTATAGGAGGTGGAGATCCAATTTTATATCAACATTTATTT
>GU707480;tax=d:Eukarya,p:Arthropoda,c:Insecta,o:Hymenoptera,f:Apidae,g:Ceratina,s:Ceratina dupla
AATTTTATATATTATATTTGCTATATGATCAGGTATAATCGGAGCATCAATAAGATTAAT
TATTCGAATAGAATTAAGAACCCCAGGAAATTGAATTAATAATGATCAAATTTATAATTC
ATTAGTTACTGCTCATGCCTTTTTAATAATTTTTTTTATAGTTATACCATTTATAATTGG
AGGATTTGGAAATTGATTAATTCCATTAATATTGGGATCACCAGATATATCTTTTCCTCG
TTTAAATAATATTAGTTTTTGATTATTACCCCCATCATTACTACTATTATTATTAAGAAA
TTTATTTTCAATAAGATCGGGAACAGGATGAACTGTTTATCCACCTTTATCATCATATTT
ATTTCACCCATCTCCGTCAGTAGACTTAGCTATTTTTTCCTTACATATGTCAGGAATTTC
ATCAATTTTAGGAGCTATTAATTTTATAGTTACTATTATAATAATAAAAAATATTTCATT
AAATTATGATTCTATCCCATTATTTTCATGAGCAGTATTTATTACAGCAATTTTATTACT
TTTATCATTACCAGTATTAGCAGGAGCTATTACTATATTATTATTTGATCGAAATTTAAA
TACATCCTTTTTTGATCCTATAGGAGGTGGAGATCCAATTTTATATCAACATTTATTT
>GU707481;tax=d:Eukarya,p:Arthropoda,c:Insecta,o:Hymenoptera,f:Apidae,g:Ceratina,s:Ceratina dupla
AATTTTATATATTATATTTGCCATATGATCAGGAATAATTGGGGCATCAATAAGATTAAT
TATTCGAATAGAATTAAGAACCCCAGGAAATTGAATTAATAATGATCAAATTTATAATTC
ATTAGTTACTGCTCATGCCTTTTTAATAATTTTTTTTATAGTTATACCATTTATAATTGG

AGGATTTGGAAATTGATTAATTCCACTAATATTAGGATCACCAGATATATCTTTTCCTCG
TTTAAATAATATTAGTTTTTGATTATTACCCCCATCATTACTACTATTATTATTAAGAAA
TTTATTTTCAATAAGACCAGGAACAGGATGAACTGTTTATCCACCTTTATCATCATATTT
ATTTCACCCATCTCCATCAGTAGATTTAGCTATTTTTTCTTTACATATATCAGGAATTTC
ATCAATTTTAGGAGCTATTAATTTTATAGTCACTATTATAATAATAAAAAATATTTCATT
AAATTATGACTCTATCCCATTATTTTCATGAGCAGTATTTATTACAGCAATTTTATTACT
TTTATCATTACCAGTATTAGCAGGAGCTATTACTATATTATTATTTGATCGAAATTTAAA
TACATCCTTTTTTGATCCTATAGGAGGTGGGGATCCAATTTTATACCAACATTTATTT
>GU707483;tax=d:Eukarya,p:Arthropoda,c:Insecta,o:Hymenoptera,f:Apidae,g:Ceratina,s:Ceratina dupla
AATTTTATATATTATATTTGCTATATGATCAGGTATAATCGGAGCATCAATAAGATTAAT
TATTCGAATAGAATTAAGAACCCCAGGAAATTGAATTAATAATGATCAAATTTATAATTC
ATTAGTTACTGCTCATGCCTTTTTAATAATTTTTTTTATAGTTATACCATTTATAATTGG
AGGATTTGGAAATTGATTAATTCCATTAATATTGGGATCACCAGATATATCTTTTCCTCG
TTTAAATAATATTAGTTTTTGATTATTACCCCCATCATTACTACTATTATTATTAAGAAA
TTTATTTTCAATAAGACCGGGAACAGGATGAACTGTTTATCCACCTTTATCATCATATTT
ATTTCACCCATCTCCGTCAGTAGACTTAGCTATTTTTTCCTTACATATGTCAGGAATTTC
ATCAATTTTAGGAGCTATTAATTTTATAGTTACTATTATAATAATAAAAAATATTTCATT
AAATTATGATTCTATCCCATTATTTTCATGAGCAGTATTTATTACAGCAATTTTATTACT
TTTATCATTACCAGTATTAGCAGGAGCTATTACTATATTATTATTTGATCGAAATTTAAA
TACATCCTTTTTTGATCCTATAGGAGGTGGGGATCCAATTTTATATCAACATTTATTT
>GU707488;tax=d:Eukarya,p:Arthropoda,c:Insecta,o:Hymenoptera,f:Apidae,g:Ceratina,s:Ceratina dupla
AATTTTATATATTATATTTGCCATATGATCAGGAATAATTGGGGCATCAATAAGATTAAT
TATTCGAATAGAATTAAGAACCCCAGGAAATTGAATTAATAATGATCAAATTTATAATTC
ATTAGTTACTGCTCATGCCTTTTTAATAATTTTTTTTATAGTTATACCATTTATAATTGG
AGGATTTGGAAATTGATTAATTCCACTAATATTAGGATCACCAGATATATCTTTTCCTCG
TTTAAATAATATTAGTTTTTGATTATTACCCCCATCATTACTACTATTATTATTAAGAAA
TTTATTTTCAATAAGACCAGGAACAGGATGAACTGTTTATCCACCTTTATCATCATATTT
ATTTCACCCATCTCCATCAGTAGATTTAGCTATTTTTTCTTTACATATATCAGGAATTTC
ATCAATTTTAGGAGCTATTAATTTTATAGTCACTATTATAATAATAAAAAATATTTCATT
AAATTATGACTCTATCCCATTATTTTCATGAGCAGTATTTATTACAGCAATTTTATTACT
TTTATCATTACCAGTATTAGCAGGAGCTATTACTATATTATTATTTGATCGAAATTTAAA
TACATCCTTTTTTGATCCTATAGGAGGTGGAGATCCAATTTTATACCAACATTTATTT
>GU707489;tax=d:Eukarya,p:Arthropoda,c:Insecta,o:Hymenoptera,f:Apidae,g:Ceratina,s:Ceratina dupla
AATTTTATATATTATATTTGCCATATGATCAGGAATAATTGGGGCATCAATAAGATTAAT
TATTCGAATAGAATTAAGAACCCCAGGAAATTGAATTAATAATGATCAAATTTATAATTC
ATTAGTTACTGCTCATGCCTTTTTAATAATTTTTTTTATAGTTATACCATTTATAATTGG
AGGGTTTGGAAATTGATTAATTCCACTAATATTAGGATCACCAGATATATCTTTTCCTCG
TTTAAATAATATTAGTTTTTGATTATTACCCCCATCATTACTACTATTATTATTAAGAAA
TTTATTTTCAATAAGACCAGGAACAGGATGAACTGTTTATCCACCTTTATCATCATATTT
ATTTCACCCATCTCCATCAGTAGATTTAGCTATTTTTTCCTTACATATATCAGGAATTTC
ATCAATTTTAGGAGCTATTAATTTTATAGTCACTATTATAATAATAAAAAATATTTCATT
AAATTATGACTCTATCCCATTATTTTCATGAGCAGTATTTATTACAGCAATTTTATTACT
TTTATCATTACCAGTATTAGCAGGAGCTATTACTATATTATTATTTGATCGAAATTTAAA
TACATCCTTTTTTGATCCTATAGGAGGCGGAGACCCAATTTTATATCAACATTTATTT
>GU707490;tax=d:Eukarya,p:Arthropoda,c:Insecta,o:Hymenoptera,f:Apidae,g:Ceratina,s:Ceratina dupla
AATTTTATATATTATATTTGCTATATGATCAGGTATAATCGGAGCATCAATAAGATTAAT
TATTCGAATAGAATTAAGAACCCCAGGAAATTGAATTAATAATGATCAAATTTATAATTC
ATTAGTTACGGCTCATGCCTTTTTAATAATTTTTTTTATAGTTATACCATTTATAATTGG
GGGATTTGGAAATTGATTAATTCCATTAATATTAGGATCACCAGATATATCTTTTCCTCG
TTTAAATAATATTAGTTTTTGATTATTACCCCCATCATTGCTACTATTATTATTAAGAAA
TTTATTTTCAATAAGACCAGGAACAGGATGAACTGTTTATCCACCTTTATCATCATATTT

ATTCCACCCATCCCCATCAGTAGATTTAGCTATTTTTTCCTTACATATATCAGGAATTTC
ATCAATTTTAGGAGCTATTAATTTTATAGTTACTATTATAATAATAAAAAATATTTCATT
AAATTATGACTCTATCCCATTATTTTCATGAGCAGTATTTATTACAGCAATTTTATTACT
TTTATCATTACCAGTATTAGCAGGAGCTATTACTATATTATTATTTGATCGAAATTTAAA
TACATCCTTTTTTGATCCTATAGGAGGTGGAGATCCAATTTTATATCAACATTTATTT
>GU707512;tax=d:Eukarya,p:Arthropoda,c:Insecta,o:Hymenoptera,f:Apidae,g:Ceratina,s:Ceratina dupla
AATTTTATATATTATATTTGCTATATGATCAGGTATAATCGGAGCATCAATAAGATTAAT
TATTCGAATAGAATTAAGAACCCCAGGAAATTGAATTAATAATGATCAAATTTATAATTC
ATTAGTTACTGCTCATGCCTTTTTAATAATTTTTTTTATAGTTATACCATTTATAATTGG
AGGATTTGGAAATTGATTAATTCCATTAATATTAGGATCACCAGATATATCTTTTCCTCG
TTTAAATAATATTAGTTTTTGATTATTACCCCCATCATTACTACTATTATTATTAAGAAA
TTTATTTTCAATAAGACCGGGAACAGGATGAACTGTTTATCCACCTTTATCATCATATTT
ATTTCACCCATCTCCATCAGTAGACTTAGCTATTTTTTCCTTACATATATCAGGAATTTC
ATCAATTTTAGGAGCTATTAATTTTATAGTTACTATTATAATAATAAAAAATATTTCATT
AAATTATGATTCTATCCCATTATTTTCATGAGCAGTATTTATTACAGCAATTTTATTACT
TTTATCATTACCAGTATTAGCAGGAGCTATTACTATATTATTATTTGATCGAAATTTAAA
TACATCTTTTTTTGATCCTATAGGAGGTGGAGATCCAATTTTATATCAACATTTATTT
>JF271036;tax=d:Eukarya,p:Arthropoda,c:Insecta,o:Hymenoptera,f:Apidae,g:Ceratina,s:Ceratina dupla
AATTTTATATATCATATTTGCTATATGATCAGGTATAATCGGAGCATCAATAAGATTAAT
TATTCGAATAGAATTAAGAACCCCGGGAAATTGAATTAATAATGATCAAATTTATAATTC
ATTAGTTACTGCTCATGCCTTTTTAATAATTTTTTTTATAGTTATACCATTTATAATTGG
AGGATTTGGAAATTGATTAATTCCACTAATATTAGGATCACCAGATATATCTTTTCCTCG
TTTAAATAATATTAGTTTTTGATTATTACCCCCATCATTATTACTATTATTATTAAGAAA
TTTATTTTCAATAAGACCAGGAACAGGGTGAACTGTTTATCCACCTTTATCATCATATTT
ATTTCACCCATCTCCATCAGTAGATTTAGCTATTTTTTCCTTACATATATCAGGAATTTC
ATCAATTTTAGGAGCTATTAATTTTATAGTTACTATTATAATAATAAAAAATATTTCATT
AAATTATGATTCTATCCCATTATTTTCATGAGCAGTATTTATTACAGCAATTTTATTACT
TTTATCATTACCAGTATTAGCAGGAGCTATTACTATATTATTATTTGATCGAAATTTAAA
TACATCCTTTTTTGATCCTATAGGAGGTGGAGATCCAATTTTATACCAACATTTATT
>JF271037;tax=d:Eukarya,p:Arthropoda,c:Insecta,o:Hymenoptera,f:Apidae,g:Ceratina,s:Ceratina dupla
AATTTTATATATCATATTTGCTATATGATCAGGTATAATCGGAGCATCAATAAGATTAAT
TATTCGAATAGAATTAAGAACCCCGGGAAATTGAATTAATAATGATCAAATTTATAATTC
ATTAGTTACTGCTCATGCCTTTTTAATAATTTTTTTTATAGTTATACCATTTATAATTGG
AGGATTTGGAAATTGATTAATTCCACTAATATTAGGATCACCAGATATATCTTTTCCTCG
TTTAAATAATATTAGTTTTTGATTATTACCCCCATCATTATTACTATTATTATTAAGAAA
TTTATTTTCAATAAGACCAGGAACAGGGTGAACTGTTTATCCACCTTTATCATCATATTT
ATTTCACCCATCTCCATCAGTAGATTTAGCTATTTTTTCCTTACATATATCAGGAATTTC
ATCAATTTTAGGAGCTATTAATTTTATAGTTACTATTATAATAATAAAAAATATTTCATT
AAATTATGATTCTATCCCATTATTTTCATGAGCAGTATTTATTACAGCAATTTTATTACT
TTTATCATTACCAGTATTAGCAGGAGCTATTACTATATTATTATTTGATCGAAATTTAAA
TACA
>JF271039;tax=d:Eukarya,p:Arthropoda,c:Insecta,o:Hymenoptera,f:Apidae,g:Ceratina,s:Ceratina dupla
AATTTTATATATCATATTTGCTATATGATCAGGTATAATCGGAGCATCAATAAGATTAAT
TATTCGAATAGAATTAAGAACCCCGGGAAATTGAATTAATAATGATCAAATTTATAATTC
ATTAGTTACTGCTCATGCCTTTTTAATAATTTTTTTTATAGTTATACCATTTATAATTGG
AGGATTTGGAAATTGATTAATTCCACTAATATTAGGGTCACCAGATATATCTTTTCCTCG
TTTAAATAATATTAGTTTTTGATTATTACCCCCATCATTATTACTATTATTATTAAGAAA
TTTATTTTCAATAAGACCAGGAACAGGGTGAACTGTTTATCCACCTTTATCATCATATTT
ATTTCACCCATCTCCATCAGTAGATTTAGCTATTTTTTCCTTACATATATCAGGAATTTC
ATCAATTTTAGGAGCTATTAATTTTATAGTTACTATTATAATAATAAAAAATATTTCATT
AAATTATGATTCTATCCCATTATTTTCATGAGCAGTATTTATTACAGCAATTTTATTACT

TTTATCATTACCAGTATTAGCAGGAGCTATTACTATATTATTATTTGATCGAAATTTAAA
TACATCCTTTTTTGATCCTATAGGAGGTGGAGATCCAATTTTATACCAACATTTATTT
>JF271044;tax=d:Eukarya,p:Arthropoda,c:Insecta,o:Hymenoptera,f:Apidae,g:Ceratina,s:Ceratina dupla
AATTTTATATATTATATTTGCTATATGATCAGGTATAATCGGAGCATCAATAAGATTAAT
TATTCGAATAGAATTAAGAACCCCGGGAAATTGAATTAATAATGATCAAATTTATAATTC
ATTAGTTACTGCTCATGCCTTTTTAATAATTTTTTTTATAGTTATACCATTTATAATTGG
AGGATTCGGAAATTGATTAATTCCACTAATATTAGGATCACCAGATATATCTTTTCCTCG
TTTAAATAATATTAGTTTTTGATTATTACCCCCATCATTATTACTATTATTATTAAGAAA
TTTATTTTCAATAAGACCAGGAACAGGATGAACTGTTTATCCACCTTTATCATCATATTT
ATTTCACCCATCTCCATCAGTAGATTTAGCTATTTTTTCCTTACATATATCAGGAATTTC
ATCAATTTTAGGAGCTATTAATTTTATAGTTACTATTATAATAATAAAAAATATTTCATT
AAATTATGACTCTATCCCATTATTTTCATGAGCAGTATTTATTACAGCAATTTTATTACT
TTTATCATTACCAGTATTAGCAGGAGCTATTACTATATTATTATTTGATCGAAATTTAAA
TACATCCTTTTTTGATCCTATAGGAGGTGGAGATCCAATTTTATACCAACATTTATTT
>JF271051;tax=d:Eukarya,p:Arthropoda,c:Insecta,o:Hymenoptera,f:Apidae,g:Ceratina,s:Ceratina dupla
AATTTTATATATTATATTTGCTATATGATCAGGTATAATCGGAGCATCAATAAGATTAAT
TATTCGAATAGAATTAAGAACCCCAGGAAATTGAATTAATAATGATCAAATTTATAATTC
ATTAGTTACTGCTCATGCCTTTTTAATAATTTTTTTTATAGTTATACCATTTATAATTGG
AGGATTTGGAAATTGATTAATTCCACTAATATTAGGATCACCAGATATATCTTTTCCTCG
TTTAAATAATATTAGTTTTTGATTATTACCCCCATCATTATTACTATTATTATTAAGAAA
TTTATTTTCAATAAGACCAGGAACAGGGTGAACTGTTTATCCACCTTTATCATCATATTT
ATTTCACCCATCTCCATCAGTAGATTTAGCTATTTTTTCCTTACATATATCAGGAATTTC
ATCAATTTTAGGAGCTATTAATTTTATAGTTACTATTATAATAATAAAAAATATTTCATT
AAATTATGATTCTATCCCATTATTTTCATGAGCAGTATTTATTACAGCAATTTTATTACT
TTTATCATTACCAGTATTAGCAGGAGCTATTACTATATTATTATTTGATCGAAATTTAAA
TACATCCTTTTTTGATCCTATAGGAGGTGGAGATCCAATTTTATACCAACATTTATT
>KP747187;tax=d:Eukarya,p:Arthropoda,c:Insecta,o:Hymenoptera,f:Apidae,g:Ceratina,s:Ceratina dupla
CCCCGGGAAATTGAATTAATAATGATCAAATTTATAATTCATTAGTTACTGCTCATGCCT
TTTTAATAATTTTTTTTATAGTTATACCATTTATAATTGGAGGATTTGGAAATTGATTAA
TTCCACTAATATTAGGATCACCAGATATATCTTTTCCTCGTTTAAATAATATTAGTTTTT
GATTATTACCCCCATCATTATTACTATTATTATTAAGAAATTTATTTTCAATAAGACCAG
GGACAGGGTGAACTGTTTATCCACCTTTATCATCATATTTATTTCACCCATCTCCATCAG
TAGATTTAGCTATTTTTTCCTTACATATATCAGGAATTTCATCAATTTTAGGAGCTATTA
ATTTTATAGTTACTATTATAATAATAAAAAATATTTCATTAAATTATGACTCTATCCCAT
TATTTTCATGAGCAGTATTTATTACAGCAATTTTATTACTTTTATCATTACCAGTATTAG
CAGGAGCTATTACTATATTATTATTTGATCGAAATTTAAATACATCCTTTTTTGATCCTA
TAGGAGGTGGAGATCCAATTTTATACCAACATTTA
>KP747192;tax=d:Eukarya,p:Arthropoda,c:Insecta,o:Hymenoptera,f:Apidae,g:Ceratina,s:Ceratina dupla
CCCCGGGAAATTGAATTAATAATGATCAAATTTATAATTCATTAGTTACTGCTCATGCCT
TTTTAATAATTTTTTTTATAGTTATACCATTTATAATTGGAGGATTTGGAAATTGATTAA
TTCCACTAATATTAGGATCACCAGATATATCTTTTCCTCGTTTAAATAATATTAGTTTTT
GATTATTACCCCCATCATTATTACTATTATTATTAAGAAATTTATTTTCAATAAGACCAG
GAACAGGATGAACTGTTTATCCACCTTTATCATCATATTTATTCCACCCATCTCCATCAG
TAGATTTAGCTATTTTTTCCTTACATATATCAGGAATTTCATCAATTTTAGGAGCTATTA
ATTTTATAGTTACTATTATAATAATAAAAAATATTTCATTAAATTATGACTCTATCCCAT
TATTTTCATGAGCAGTATTTATTACAGCAATTTTATTACTTTTATCATTACCAGTATTAG
CAGGAGCTATTACTATATTATTATTTGATCGAAATTTAAATACATCCTTTTTTGATCCTA
TAGGAGGTGGAGATCCAATTTTATACCAACATTTA
>KP747197;tax=d:Eukarya,p:Arthropoda,c:Insecta,o:Hymenoptera,f:Apidae,g:Ceratina,s:Ceratina dupla
CCCCAGGAAATTGAATTAATAATGATCAAATTTATAATTCATTAGTTACTGCTCATGCCT
TTTTAATAATTTTTTTTATAGTTATACCATTTATAATTGGAGGATTTGGAAATTGATTAA

TTCCACTAATATTAGGATCACCAGATATATCTTTTCCTCGTTTAAATAATATTAGTTTTT
GATTATTACCCCCATCATTATTACTATTATTATTAAGAAATTTATTTTCAATAAGACCAG
GGACAGGGTGAACTGTTTATCCACCTTTATCATCATATTTATTTCACCCATCTCCATCAG
TAGATTTAGCTATTTTTTCCTTACATATATCAGGAATTTCATCAATTTTAGGAGCTATTA
ATTTTATAGTTACTATTATAATAATAAAAAATATTTCATTAAATTATGACTCTATCCCAT
TATTTTCATGAGCAGTATTTATTACAGCAATTTTATTACTTTTATCATTACCAGTATTAG
CAGGAGCTATTACTATATTATTATTTGATCGAAATTTAAATACATCCTTTTTTGATCCTA
TAGGAGGTGGAGATCCAATTTTATACCAACATTTA
>KP747198;tax=d:Eukarya,p:Arthropoda,c:Insecta,o:Hymenoptera,f:Apidae,g:Ceratina,s:Ceratina dupla
CCCCGGGAAATTGAATTAATAATGATCAAATTTATAATTCATTAGTTACTGCTCATGCCT
TTTTAATAATTTTTTTTATAGTTATACCATTTATAATTGGAGGATTTGGAAATTGATTAA
TTCCACTAATATTAGGATCACCAGATATATCTTTTCCTCGTTTAAATAATATTAGTTTTT
GATTATTACCCCCATCATTATTACTATTATTATTAAGAAATTTATTTTCGATAAGACCAG
GGACAGGGTGAACTGTTTATCCACCTTTATCATCATATTTATTTCACCCATCTCCATCAG
TAGATTTAGCTATTTTTTCCTTACATATATCAGGAATTTCATCAATTTTAGGAGCTATTA
ATTTTATAGTTACTATTATAATAATAAAAAATATTTCATTAAATTATGACTCTATCCCAT
TATTTTCATGAGCAGTATTTATTACAGCAATTTTATTACTTTTATCATTACCAGTATTAG
CAGGAGCTATTACTATATTATTATTTGATCGAAATTTAAATACATCCTTTTTTGATCCTA
TAGGAGGTGGAGATCCAATTTTATACCAACATTTA
>KP747199;tax=d:Eukarya,p:Arthropoda,c:Insecta,o:Hymenoptera,f:Apidae,g:Ceratina,s:Ceratina dupla
CCCCGGGAAATTGAATTAATAATGATCAAATTTATAATTCATTAGTTACTGCTCATGCCT
TTTTAATAATTTTTTTTATAGTTATACCATTTATAATTGGAGGATTTGGAAATTGATTAA
TTCCACTAATATTAGGATCACCAGATATATCTTTTCCTCGTTTAAATAATATTAGTTTTT
GATTATTACCCCCATCATTATTACTATTATTATTAAGAAATTTATTTTCAATAAGACCAG
GGACAGGGTGAACTGTTTATCCACCTTTATCATCATATTTATTTCACCCATCTCCATCAG
TAGATTTAGCTATTTTTTCCTTGCATATATCAGGAATTTCATCAATTTTAGGAGCTATTA
ATTTTATAGTTACTATTATAATAATAAAAAATATTTCATTAAATTATGACTCTATCCCAT
TATTTTCATGAGCAGTATTTATTACAGCAATTTTATTACTTTTATCATTACCAGTATTAG
CAGGAGCTATTACTATATTATTATTTGATCGAAATTTAAATACATCCTTTTTTGATCCTA
TAGGAGGTGGAGATCCAATTTTATACCAACATTTA
>KP747200;tax=d:Eukarya,p:Arthropoda,c:Insecta,o:Hymenoptera,f:Apidae,g:Ceratina,s:Ceratina dupla
CCCCGGGAAATTGAATTAAAAATGATCAAATTTATAATTCATTAGTTACTGCTCATGCCT
TTTTAATAATTTTTTTTATAGTTATACCATTTATAATTGGAGGATTTGGAAATTGATTAA
TTCCACTAATATTAGGATCACCAGATATATCTTTTCCTCGTTTAAATAATATTAGTTTTT
GATTATTACCCCCATCATTATTACTATTATTATTAAGAAATTTATTTTCAATAAGACCAG
GAACAGGGTGAACTGTTTATCCACCTTTATCATCATATTTATTTCACCCATCTCCATCAG
TAGATTTAGCTATTTTTTCCTTACATATATCAGGAATTTCATCAATTTTAGGAGCTATTA
ATTTTATAGTTACTATTATAATAATAAAAAATATTTCATTAAATTATGATTCTATCCCAT
TATTTTCATGAGCAGTATTTATTACAGCAATTTTATTACTTTTATCATTACCAGTATTAG
CAGGAGCTATTACTATATTATTATTTGATCGAAATTTAAATACATCCTTTTTTGATCCTA
TAGGAGGTGGAGATCCAATTTTATACCAACATTTA
>KP747201;tax=d:Eukarya,p:Arthropoda,c:Insecta,o:Hymenoptera,f:Apidae,g:Ceratina,s:Ceratina dupla
CCCCGGGAAATTGAATTAATAATGATCAAATTTATAATTCATTAGTTACTGCTCATGCCT
TTTTAATAATTTTTTTTATAGTTATACCATTTATAATTGGAGGATTTGGAAATTGATTAA
TTCCACTAATATTAGGATCACCAGATATATCTTTTCCTCGTTTAAATAATATTAGTTTTT
GATTATTACCCCCATCATTATTACTATTATTATTAAGAAATTTATTTTCAATAAGACCAG
GAACAGGGTGAACTGTTTATCCACCTTTATCATCATATTTATTTCACCCATCTCCATCAG
TAGATTTAGCTATTTTTTCCTTACATATATCAGGAATTTCATCAATTTTAGGAGCTATTA
ATTTTATAGTTACTATTATAATAATAAAAAATATTTCATTAAATTATGATTCTATCCCAT
TATTTTCATGAGCAGTATTTATTACAGCAATTTTATTACTTTTATCATTACCAGTATTAG
CAGGAGCTATTACTATATTATTATTTGATCGAAATTTAAATACATCCTTTTTTGATCCTA

TAGGAGGTGGAGATCCAATTTTATACCAACATTTA
>KP747203;tax=d:Eukarya,p:Arthropoda,c:Insecta,o:Hymenoptera,f:Apidae,g:Ceratina,s:Ceratina dupla
CCCCGGGAAATTGAATTAATAATGATCAAATTTATAATTCATTAGTTACTGCTCATGCCT
TTTTAATAATTTTTTTTATAGTTATACCATTTATAATTGGAGGATTTGGAAATTGATTAA
TTCCACTAATATTAGGATCACCAGATATATCTTTTCCTCGTTTAAATAATATTAGTTTTT
GATTATTACCCCCATCATTATTACTATTATTATTAAAAAATTTATTTTCAATAAAACCAG
GAACAGGGTGAACTGTTTATCCACCTTTATCATCATATTTATTTCACCCATCTCCATCAG
TAGATTTAGCTATTTTTTCCTTACATATATCAGGAATTTCATCAATTTTAGGAGCTATTA
ATTTTATAGTTACTATTATAATAATAAAAAATATTTCATTAAATTATGACTCTATCCCAT
TATTTTCATGAGCAGTATTTATTACAGCAATTTTATTACTTTTATCATTACCAGTATTAG
CAGGAGCTATTACTATATTATTATTTGATCGAAATTTAAATACATCCTTTTTTGATCCTA
TAGGAGGTGGAGATCCAATTTTATACCAACATTTA
>KP747204;tax=d:Eukarya,p:Arthropoda,c:Insecta,o:Hymenoptera,f:Apidae,g:Ceratina,s:Ceratina dupla
CCCCGGGAAATTGAATTAATAATGATCAAATTTATAATTCATTAGTTACTGCTCATGCCT
TTTTAATAATTTTTTTTATAGTTATACCATTTATAATTGGAGGATTTGGAAATTGATTAA
TTCCACTAATATTAGGATCACCAGATATATCTTTTCCTCGTTTAAATAATATTAGTTTTT
GATTATTACCCCCATCATTATTACTATTATTATTAAGAAATTTATTTTCAATAAGACCAG
GGACAGGATGAACTGTTTATCCACCTTTATCATCATATTTATTTCACCCATCTCCATCAG
TAGATTTAGCTATTTTTTCCTTACATATATCAGGAATTTCATCAATTTTAGGAGCTATTA
ATTTTATAGTTACTATTATAATAATAAAAAATATTTCATTAAATTATGACTCTATCCCAT
TATTTTCATGAGCAGTATTTATTACAGCAATTTTATTACTTTTATCATTACCAGTATTAG
CAGGAGCTATTACTATATTATTATTTGATCGAAATTTAAATACATCCTTTTTTGATCCTA
TAGGAGGTGGAGATCCAATTTTATACCAACATTTA
>KP747205;tax=d:Eukarya,p:Arthropoda,c:Insecta,o:Hymenoptera,f:Apidae,g:Ceratina,s:Ceratina dupla
CCCCGGGAAATTGAATTAATAATGATCAAATTTATAATTCATTAGTTACTGCTCATGCCT
TTTTAATAATTTTTTTTATAGTTATACCATTTATAATTGGAGGATTTGGAAATTGATTAA
TTCCACTAATATTAGGATCACCAGATATATCTTTTCCTCGTTTAAATAATATTAGTTTTT
GATTATTACCCCCATCATTATTACTATTATTATTAAGAAATTTATTTTCAATAAGACCAG
GAACAGGATGAACTGTTTATCCACCTTTATCATCATATTTATTTCACCCATCTCCATCAG
TAGATTTAGCTATTTTTTCCTTACATATATCAGGAATTTCATCAATTTTAGGAGCTATTA
ATTTTATAGTTACTATTATAATAATAAAAAATATTTCATTAAATTATGACTCTATCCCAT
TATTTTCATGAGCAGTATTTATTACAGCAATTTTATTACTTTTATCATTACCAGTATTAG
CAGGAGCTATTACTATATTATTATTTGATCGAAATTTAAATACATCCTTTTTTGATCCTA
TAGGAGGTGGAGATCCAATTTTATACCAACATTTA
>KP747206;tax=d:Eukarya,p:Arthropoda,c:Insecta,o:Hymenoptera,f:Apidae,g:Ceratina,s:Ceratina dupla
CCCCAGGAAATTGAATTAATAATGATCAAATTTATAATTCATTAGTTACTGCTCATGCCT
TTTTAATAATTTTTTTTATAGTTATACCATTTATAATTGGAGGATTTGGAAATTGATTAA
TTCCACTAATATTAGGATCACCAGATATATCTTTTCCTCGTTTAAATAATATTAGTTTTT
GATTATTACCCCCATCATTATTACTATTATTATTAAGAAATTTATTTTCAATAAGACCAG
GAACAGGGTGAACTGTTTATCCACCTTTATCATCATATTTATTTCACCCATCTCCATCAG
TAGATTTAGCTATTTTTTCCTTACATATATCAGGAATTTCATCAATTTTAGGAGCTATTA
ATTTTATAGTTACTATTATAATAATAAAAAATATTTCATTAAATTATGACTCTATCCCAT
TATTTTCATGAGCAGTATTTATTACAGCAATTTTATTACTTTTATCATTACCAGTATTAG
CAGGAGCTATTACTATATTATTATTTGATCGAAATTTAAATACATCCTTTTTTGATCCTA
TAGGAGGTGGAGATCCAACTTTATATCAACATTTA
>KP747207;tax=d:Eukarya,p:Arthropoda,c:Insecta,o:Hymenoptera,f:Apidae,g:Ceratina,s:Ceratina dupla
CCCCAGGAAATTGAATTAATAATGATCAAATTTATAATTCATTAGTTACTGCTCATGCCT
TTTTAATAATTTTTTTTATAGTTATACCATTTATAATTGGAGGATTTGGAAATTGATTAA
TTCCACTAATATTAGGATCACCAGATATATCTTTTCCTCGTTTAAATAATATTAGTTTTT
GATTATTACCCCCATCATTATTACTATTATTATTAAGAAATTTATTTTCAATAAGACCAG
GAACAGGGTGAACTGTTTATCCACCTTTATCATCATATTTATTTCACCCATCTCCATCAG

TAGATTTAGCTATTTTTTCCTTACATATATCAGGAATTTCATCAATTTTAGGAGCTATTA
ATTTTATAGTTACTATTATAATAATAAAAAATATTTCATTAAATTATGACTCTATCCCAT
TATTTTCATGAGCAGTATTTATTACAGCAATTTTATTACTTTTATCATTACCAGTATTAG
CAGGAGCTATTACTATATTATTATTTGATCGAAATTTAAATACATCCTTTTTTGATCCTA
TAGGAGGTGGAGATCCAATTTTATATCAACATTTA
>KP747208;tax=d:Eukarya,p:Arthropoda,c:Insecta,o:Hymenoptera,f:Apidae,g:Ceratina,s:Ceratina dupla
CCCCGGGAAATTGAATTAATAATGATCAAATTTATAATTCATTAGTTACTGCTCATGCCT
TTTTAATAATTTTTTTTATAGTTATACCATTTATAATTGGAGGATTTGGAAATTGATTAA
TTCCACTAATATTAGGATCACCAGATATATCTTTTCCTCGTTTAAATAATATTAGTTTTT
GATTATTACCCCCATCATTATTACTATTATTATTAAGAAATTTATTTTCAATAAGACCAG
GAACAGGGTGAACTGTTTATCCACCTTTATCATCATATTTATTTCACCCATCTCCATCAG
TAGATTTAGCTATTTTTTCCTTACATATATCAGGAATTTCATCAATTTTAGGAGCTATTA
ATTTTATAGTTACTATTATAATAATAAAAAATATTTCATTAAATTATGACTCTATCCCAT
TATTTTCATGAGCAGTATTTATTACAGCAATTTTATTACTTTTATCATTACCAGTATTAG
CAGGAGCTATTACTATATTATTATTTGATCGAAATTTAAATACATCCTTTTTTGATCCTA
TAGGAGGTGGAGATCCAATTTTATACCAACATTTA
>KP747211;tax=d:Eukarya,p:Arthropoda,c:Insecta,o:Hymenoptera,f:Apidae,g:Ceratina,s:Ceratina dupla
CCCCGGGAAATTGAATTAATAATGATCAAATTTATAATTCATTAGTTACTGCTCATGCCT
TTTTAATAATTTTTTTTATAGTTATACCATTTATAATTGGAGGATTTGGAAATTGATTAA
TTCCACTAATATTAGGATCACCAGATATATCTTTTCCTCGTTTAAATAATATTAGTTTTT
GATTATTACCCCCATCATTATTACTATTATTATTAAGAAATTTATTTTCAATAAGACCAG
GGACAGGGTGAACTGTTTACCCACCTTTATCATCATATTTATTTCACCCATCTCCATCAG
TAGATTTAGCTATTTTTTCCTTACATATATCAGGAATTTCATCAATTTTAGGAGCTATTA
ATTTTATAGTTACTATTATAATAATAAAAAATATTTCATTAAATTATGACTCTATCCCAT
TATTTTCATGAGCAGTATTTATTACAGCAATTTTATTACTTTTATCATTACCAGTATTAG
CAGGAGCTATTACTATATTATTATTTGATCGAAATTTAAATACATCCTTTTTTGATCCTA
TAGGAGGTGGAGATCCAATTTTATACCAACATTTA
>KP747214;tax=d:Eukarya,p:Arthropoda,c:Insecta,o:Hymenoptera,f:Apidae,g:Ceratina,s:Ceratina dupla
CCCCGGGAAATTGAATTAATAATGATCAAATTTATAATTCATTAGTTACTGCTCATGCCT
TTTTAATAATTTTTTTTATAGTTATACCATTTATAATTGGAGGATTTGGAAATTGATTAA
TTCCACTAATATTAGGATCACCAGATATATCTTTTCCTCGTTTAAATAATATTAGTTTTT
GATTATTACCCCCATCATTATTACTATTATTATTAAGAAATTTATTTTCAATAAGACCAG
GAACAGGGTGAACTGTTTATCCACCTTTATCATCATATTTATTTCACCCATCTCCATCAG
TAGATTTAGCTATTTTTTCCTTACATATATCAGGAATTTCATCAATTTTAGGAGCTATTA
ATTTTATAGTTACTATTATAATAATAAAAAATATTTCATTAAATTATGACTCTATCCCAT
TATTTTCATGAGCAGTATTTATTACAGCAATTTTATTACTTTTATCATTACCAGTATTAG
CAGGAGCTATTACTATATTATTATTTGATCGAAATTTAAATACATCCTTTTTTGATCCTA
TAGGAGGTGGGGATCCAATTTTATACCAACATTTA
>FJ582111;tax=d:Eukarya,p:Arthropoda,c:Insecta,o:Hymenoptera,f:Apidae,g:Bombus,s:Bombus citrinus
AATAATATATTTTATTTTTGCTATATGATCAGGAATAATTGGTTCTTCAATAAGAATAAT
AATTCGAATAGAATTAAGTCATCCCGGTTCATGAATTAATAATGACCAAATTTATAATTC
TATAGTTACTAGACATGCATTTTTAATAATTTTTTTTATAGTTATACCATTTTTAATTGG
TGGATTTGGAAATTATTTAATCCCCCTAATATTAGGATCTCCTGATATAGCTTTCCCACG
ATTAAATAATTTAAGATTCTGACTATTACCTCCATCATTAACATTATTAATCATAAGAAA
CTTATTCACTCCTAATACAGGAACCGGTTGAACAATCTATCCTCCCTTATCTTCTTATTT
ATTCCATTCATCTCCTTCAGTAGACATAGCAATTTTTTCTTTACATATAACAGGTATTTC
CTCAATTATTGGTTCATTAAATTTTATAGTTTCAATTATAATAATAAAAAATTATTCATT
AAATTTTGATCAAATTAATTTATTTTCATGATCAGTCTCTATCACAGTAATTTTATTAAC
TTTATCTTTACCAGTCCTAGCAGGAGCTATTACAATATTATTATTTGATCGAAATTTTAA
TACATCTTTTTTTGACCCAATAGGAGGAGGAGATCCAATTCTTTATCAACATCTATTT
>FJ582113;tax=d:Eukarya,p:Arthropoda,c:Insecta,o:Hymenoptera,f:Apidae,g:Bombus,s:Bombus citrinus

GCTATATGATCAGGAATAATTGGCTCTTCAATAAGAATAATAATTCGAATAGAATTAAGT
CATCCCGGTTCATGAATTAATAATGACCAAATTTATAATTCTATAGTTACTAGACATGCA
TTTTTAATAATTTTTTTTATAGTTATACCATTTTTAATTGGTGGATTTGGAAATTATTTA
ATCCCCCTAATATTAGGATCTCCTGATATAGCTTTCCCACGATTAAATAATTTAAGATTC
TGACTATTACCTCCATCATTAACATTATTAATCATAAGAAACTTATTCACTCCTAATACA
GGAACCGGTTGAACAATCTATCCCCCCTTATCTTCTTATTTATTCCATTCATCTCCTTCA
GTAGACATAGCAATTTTCTCTTTACATATAACAGGTATTTCCTCAATTATTGGTTCATTA
AATTTTATAGTTTCAATTATAATAATAAAAAATTATTCATTAAATTTTGATCAAATTAAT
TTATTTTCATGATCAGTCTCTATCACAGTAATTTTATTAACTTTATCTTTACCAGTTCTA
GCAGGAGCTATTACAATATTATTATTTGATCGAAATTTTAATACATCTTTTTTTGACCCA
ATAGGAGGAGGAGATCCAATTCTTTATCAACATCTATTT
>KR406401;tax=d:Eukarya,p:Arthropoda,c:Insecta,o:Hymenoptera,f:Apidae,g:Bombus,s:Bombus citrinus
AATAATATATTTTATTTTTGCTATATGATCAGGAATAATTGGTTCTTCAATAAGAATAAT
AATTCGAATAGAATTAAGTCATCCCGGTTCATGAATTAATAATGACCAAATTTATAATTC
TATAGTTACTAGACATGCATTTTTAATAATTTTTTTTATAGTTATACCATTTTTAATTGG
TGGATTTGGAAATTATTTAATCCCCCTAATATTAGGATCTCCTGATATAGCTTTCCCACG
ATTAAATAATTTAAGATTCTGACTATTACCTCCATCATTAACATTATTAATCATAAGAAA
CTTATTCACTCCTAATACAGGAACCGGTTGAACAATCTATCCCCCCTTATCTTCTTATTT
ATTCCATTCATCTCCTTCAGTAGACATAGCAATTTTCTCTTTACATATAACAGGTATTTC
CTCAATTATTGGTTCATTAAATTTTATAGTTTCAATTATAATAATAAAAAATTATTCATT
AAATTTTGATCAAATTAATTTATTTTCATGATCAGTCTCTATCACAGTAATTTTATTAAC
TTTATCTTTACCAGTTCTAGCAGGAGCTATTACAATATTATTATTTGATCGA
>KR407246;tax=d:Eukarya,p:Arthropoda,c:Insecta,o:Hymenoptera,f:Apidae,g:Bombus,s:Bombus citrinus
ATTTTATTTTTGCTATATGATCAGGAATAATTGGTTCTTCAATAAGAATAATAATTCGAA
TAGAATTAAGTCATCCCGGTTCATGAATTAATAATGACCAAATTTATAATTCTATAGTTA
CTAGACATGCATTTTTAATAATTTTTTTTATAGTTATACCATTTTTAATTGGTGGATTTG
GAAATTATTTAATCCCCCTAATATTAGGATCTCCTGATATAGCTTTCCCACGATTAAATA
ATTTAAGATTCTGACTATTACCTCCATCATTAACATTATTAATCATAAGAAACTTATTCA
CTCCTAATACAGGAACCGGTTGAACAATCTATCCCCCCTTATCTTCTTATTTATTCCATT
CATCTCCTTCAGTAGACATAGCAATTTTCTCTTTACATATAACAGGTATTTCCTCAATTA
TTGGTTCATTAAATTTTATAGTTTCAATTATAATAATAAAAAATTATTCATTAAATTTTG
ATCAAATTAATTTATTTTCATGATCAGTCTCTATCACAGTAATTTTATTAACTTTATC
>KR407880;tax=d:Eukarya,p:Arthropoda,c:Insecta,o:Hymenoptera,f:Apidae,g:Bombus,s:Bombus citrinus
ATAATATATTTTATTTTTGCTATATGATCAGGAATAATTGGTTCTTCAATAAGAATAATA
ATTCGAATAGAATTAAGTCATCCCGGTTCATGAATTAATAATGACCAAATTTATAATTCT
ATAGTTACTAGACATGCATTTTTAATAATTTTTTTTATAGTTATACCATTTTTAATTGGT
GGATTTGGAAATTATTTAATCCCCCTAATATTAGGATCTCCTGATATAGCTTTCCCACGA
TTAAATAATTTAAGATTCTGACTATTACCTCCATCATTAACATTATTAATCATAAGAAAC
TTATTCACTCCTAATACAGGAACCGGTTGAACAATCTATCCTCCCTTATCTTCTTATTTA
TTCCATTCATCTCCTTCAGTAGACATAGCAATTTTTTCTTTACATATAACAGGTATTTCC
TCAATTATTGGTTCATTAAATTTTATAGTTTCAATTATAATAATAAAAAATTATTCATTA
AATTTTGATCAAATTAATTTATTTTCATGATCAGTCTCTATCACAGTAATTTTATTAACT
TTATCTTTACCAGTCCTAGCAGGAGCTATTACAATATTATTATTTGAT
>KR414904;tax=d:Eukarya,p:Arthropoda,c:Insecta,o:Hymenoptera,f:Apidae,g:Bombus,s:Bombus citrinus
TATTTTTGCTATATGATCAGGAATAATTGGTTCTTCAATAAGAATAATAATTCGAATAGA
ATTAAGTCATCCCGGTTCATGAATTAATAATGACCAAATTTATAATTCTATAGTTACTAG
ACATGCATTTTTAATAATTTTTTTTATAGTTATACCATTTTTAATTGGTGGATTTGGAAA
TTATTTAATCCCCCTAATATTAGGATCTCCTGATATAGCTTTCCCACGATTAAATAATTT
AAGATTCTGACTATTACCTCCATCATTAACATTATTAATCATAAGAAACTTATTCACTCC
TAATACAGGAACCGGTTGAACAATCTATCCCCCCTTATCTTCTTATTTATTCCATTCATC
TCCTTCAGTAGACATAGCAATTTTCTCTTTACATATAACAGGTATTTCCTCAATTATTGG

TTCATTAAATTTTATAGTTTCAATTATAATAATAAAAAATTATTCATTAAATTTTGATCA
AATTAATTTATTTTCATGATCAGTCTCTATCACAGTAATTTTATTAACTTTATCTTTACC
AGTCCTAGCAGGAGCTATTACAATATTATTATTTGAT
>KR418515;tax=d:Eukarya,p:Arthropoda,c:Insecta,o:Hymenoptera,f:Apidae,g:Bombus,s:Bombus citrinus
AATAATATATTTTATTTTTGCTATATGATCAGGAATAATTGGTTCTTCAATAAGAATAAT
AATTCGAATAGAATTAAGTCATCCCGGTTCATGAATTAATAATGACCAAATTTATAATTC
TATAGTTACTAGACATGCATTTTTAATAATTTTTTTTATAGTTATACCATTTTTAATTGG
TGGATTTGGAAATTATTTAATCCCCCTAATATTAGGATCTCCTGATATAGCTTTCCCACG
ATTAAATAATTTAAGATTCTGACTATTACCTCCATCATTAACATTATTAATCATAAGAAA
CTTATTCACTCCTAATACAGGAACCGGTTGAACAATCTATCCTCCCTTATCTTCTTATTT
ATTCCATTCATCTCCTTCAGTAGACATAGCAATTTTTTCTTTACATATAACAGGTATTTC
CTCAATTATTGGTTCATTAAATTTTATAGTTTCAATTATAATAATAAAAAATTATTCATT
AAATTTTGATCAAATTAATTTATTTTCATGATCAGTCTCTATCACAGTAATTTTATTAAC
TTTATCTTTACCAGTCCTAGCAGGAGCTATTACAATATT
>KR806784;tax=d:Eukarya,p:Arthropoda,c:Insecta,o:Hymenoptera,f:Apidae,g:Bombus,s:Bombus citrinus
AATAATATATTTTATTTTTGCTATATGATCAGGAATAATTGGTTCTTCAATAAGAATAAT
AATTCGAATAGAATTAAGTCATCCCGGTTCATGAATTAATAATGACCAAATTTATAATTC
TATAGTTACTAGACATGCATTTTTAATAATTTTTTTTATAGTTATACCATTTTTAATTGG
TGGATTTGGAAATTATTTAATCCCCCTAATATTAGGATCTCCTGATATAGCTTTCCCACG
ATTAAATAATTTAAGATTCTGACTATTACCTCCATCATTAACATTATTAATCATAAGAAA
CTTATTCACTCCTAATACAGGAACCGGTTGAACAATCTATCCTCCCTTATCTTCTTATTT
ATTCCATTCATCTCCTTCAGTAGACATAGCAATTTTTTCTTTACATATAACAGGTATTTC
CTCAATTATTGGTTCATTAAATTTTATAGTTTCAATTATAATAATAAAAAATTATTCATT
AAATTTTGATCAAATTAATTTATTTTCATGATCAGTCTCTATCACAGTAATTTTATTAAC
TTTATCTTTACCAGTCCTAGCAGGAGCTATTACAATATTATTATTTGAT
>KR890826;tax=d:Eukarya,p:Arthropoda,c:Insecta,o:Hymenoptera,f:Apidae,g:Bombus,s:Bombus citrinus
ATAATATATTTTATTTTTGCTATATGATCAGGAATAATTGGTTCTTCAATAAGAATAATA
ATTCGAATAGAATTAAGTCATCCCGGTTCATGAATTAATAATGACCAAATTTATAATTCT
ATAGTTACTAGACATGCATTTTTAATAATTTTTTTTATAGTTATACCATTTTTAATTGGT
GGATTTGGAAATTATTTAATCCCCCTAATATTAGGATCTCCTGATATAGCTTTCCCACGA
TTAAATAATTTAAGATTCTGACTATTACCTCCATCATTAACATTATTAATCATAAGAAAC
TTATTCACTCCTAATACAGGAACCGGTTGAACAATCTATCCCCCCTTATCTTCTTATTTA
TTCCATTCATCTCCTTCAGTAGACATAGCAATTTTCTCTTTACATATAACAGGTATTTCC
TCAATTATTGGTTCATTAAATTTTATAGTTTCAATTATAATAATAAAAAATTATTCATTA
AATTTTGATCAAATTAATTTATTTTCATGATCAGTCTCTATCACAGTAATTTTATTAACT
TTATCTTTACCAGTTCTAGCAGGA
>KR897150;tax=d:Eukarya,p:Arthropoda,c:Insecta,o:Hymenoptera,f:Apidae,g:Bombus,s:Bombus citrinus
ATAATATATTTTATTTTTGCTATATGATCAGGAATAATTGGTTCTTCAATAAGAATAATA
ATTCGAATAGAATTAAGTCATCCCGGTTCATGAATTAATAATGACCAAATTTATAATTCT
ATAGTTACTAGACATGCATTTTTAATAATTTTTTTTATAGTTATACCATTTTTAATTGGT
GGATTTGGAAATTATTTAATCCCCCTAATATTAGGATCTCCTGATATAGCTTTCCCACGA
TTAAATAATTTAAGATTCTGACTATTACCCCCATCATTAACATTATTAATCATAAGAAAC
TTATTCACTCCTAATACAGGAACCGGTTGAACAATCTATCCCCCCTTATCTTCTTATTTA
TTCCATTCATCTCCTTCAGTAGACATAGCAATTTTCTCTTTACATATAACAGGTATTTCC
TCAATTATTGGTTCATTAAATTTTATAGTTTCAATTATAATAATAAAAAATTATTCATTA
AATTTTGATCAAATTAATTTATTTTCATGATCAGTCTCTATTACAGTAATTTTATTAACT
TTATCTTTACCAGTCCTAGCAGGAGCTATTACAATATTATTATTTGAT
>KR898362;tax=d:Eukarya,p:Arthropoda,c:Insecta,o:Hymenoptera,f:Apidae,g:Bombus,s:Bombus citrinus
TTTATTTTTGCTATATGATCAGGAATAATTGGTTCTTCAATAAGAATAATAATTCGAATA
GAATTAAGTCATCCCGGTTCATGAATTAATAATGACCAAATTTATAATTCTATAGTTACT
AGACATGCATTTTTAATAATTTTTTTTATAGTTATACCATTTTTAATTGGTGGATTTGGA

AATTATTTAATCCCCCTAATATTAGGATCTCCTGATATAGCTTTCCCACGATTAAATAAT
TTAAGATTCTGACTATTACCCCCATCATTAACATTATTAATCATAAGAAACTTATTCACT
CCTAATACAGGAACCGGTTGAACAATCTATCCCCCCTTATCTTCTTATTTATTCCATTCA
TCTCCTTCAGTAGACATAGCAATTTTCTCTTTACATATAACAGGTATTTCCTCAATTATT
GGTTCATTAAATTTTATAGTTTCAATTATAATAATAAAAAATTATTCATTAAATTTTGAT
CAAATTAATTTATTTTCATGATCAGTCTCTATTACAGTAATTTTATTAACTTTATCTTTA
CCAGTCCTAGCAGGAGCTATTACAATATTATTATTTGAT
>MG442091;tax=d:Eukarya,p:Arthropoda,c:Insecta,o:Hymenoptera,f:Apidae,g:Bombus,s:Bombus citrinus
TATATTTTATTTTTGCTATATGATCAGGAATAATTGGTTCTTCAATAAGAATAATAATTC
GAATAGAATTAAGTCATCCCGGTTCATGAATTAATAATGACCAAATTTATAATTCTATAG
TTACTAGACATGCATTTTTAATAATTTTTTTTATAGTTATACCATTTTTAATTGGTGGAT
TTGGAAATTATTTAATCCCCCTAATATTAGGATCTCCTGATATAGCTTTCCCACGATTAA
ATAATTTAAGATTCTGACTATTACCCCCATCATTAACATTATTAATCATAAGAAACTTAT
TCACTCCTAATACAGGAACCGGTTGAACAATCTATCCCCCCTTATCTTCTTATTTATTCC
ATTCATCTCCTTCAGTAGACATAGCAATTTTCTCTTTACATATAACAGGTATTTCCTCAA
TTATTGGTTCATTAAATTTTATAATTTCAATTATAATAATAAAAAATTATTCATTAAATT
TTGATCAAATTAATTTATTTTCATGATCAGTCTCTATTACAGTAATTTTATTAACTTTAT
CTTTACCAGTCCTAGCAGGAGCTATTACAATATTATTATTTGATCGAAATTTTAATACAT
CTTTTTTTGACCCAATAGGAGGAGGAGATCCAATTCTTTATCAACATCTA
>MG448518;tax=d:Eukarya,p:Arthropoda,c:Insecta,o:Hymenoptera,f:Apidae,g:Bombus,s:Bombus citrinus
ATAATATATTTTATTTTTGCTATATGATCAGGAATAATTGGTTCTTCAATAAGAATAATA
ATTCGAATAGAATTAAGTCATCCCGGTTCATGAATTAATAATGACCAAATTTATAATTCT
ATAGTTACTAGACATGCATTTTTAATAATTTTTTTTATAGTTATACCATTTTTAATTGGT
GGATTTGGAAATTATTTAATCCCCCTAATATTAGGATCTCCTGATATAGCTTTCCCACGA
TTAAATAATTTAAGATTCTGACTATTACCCCCATCATTAACATTATTAATCATAAGAAAC
TTATTCACTCCTAATACAGGAACCGGTTGAACAATCTATCCCCCCTTATCTTCTTATTTA
TTCCATTCATCTCCTTCAGTAGACATAGCAATTTTCTCTTTACATATAACAGGTATTTCC
TCAATTATTGGTTCATTAAATTTTATAGTTTCAATTATAATAATAAAAAATTATTCATTA
AATTTTGATCAAATTAATTTATTTTCATGATCAGTCTCTATTACAGTAATTTTATTAACT
TTATCTTTACCAGTCCTAGCAGGAGCTATTACAATATTATTATTTGATCGAAATTTTAAT
ACATCTTTTTTTGACCCAATAGGAGGAGGAGATCCAATTCTTTATCAACATCTATTT
>HM884069;tax=d:Eukarya,p:Arthropoda,c:Insecta,o:Hymenoptera,f:Apidae,g:Bombus,s:Bombus vagans
AATAATATATTTTATTTTTGCTATATGATCAGGAATAATTGGATCATCAATAAGATTACT
TATTCGAATAGAATTAAGACATCCTGGAATATGAATTAATAATGATCAAATTTATAATTC
ATTAGTTACTAGACATGCATTTTTAATAATTTTTTTTATAGTTATACCATTTATAATTGG
AGGATTTGGAAATTATTTAATTCCTTTAATATTAGGAGCACCTGATATAGCTTTTCCACG
AATAAATAATATTAGATTTTGATTACTTCCTCCATCTCTTTTAATATTACTTTTAAGAAA
TTTATTCACACCAAATGTTGGTACAGGATGAACTGTTTATCCTCCTTTATCATCATATAT
ATTTCATTCATCACCATCAGTTGATATTGCAATTTTTTCACTACATATAACAGGTATTTC
TTCAATTATTGGATCTTTAAATTTTATTGTAACTATTATATTAATAAAAAATTATTCATT
AAATTATGATCAAATTAATTTATTTTCTTGATCAGTATGTATTACAGTAATTTTATTAAT
TTTATCCTTACCAGTTTTAGCAGGAGCTATTACAATACTTCTTTTTGATCGAAATTTTAA
TACATCTTTTTTTGATCCAATAGGAGGAGGTGATCCTATTCTTTATCAACATTTATTT
>FJ582155;tax=d:Eukarya,p:Arthropoda,c:Insecta,o:Hymenoptera,f:Apidae,g:Bombus,s:Bombus vagans
TCAAAAAGATTACTTATTCGAATAGAATTAAGACATCCTGGAATATGAATTAATAATGAT
CAAATTTATAATTCATTAGTTACTAGACATGCATTTTTAATAATTTTTTTTATAGTTATA
CCATTTATAATTGGAGGATTTGGAAATTATTTAATTCCTTTAATATTAGGAGCACCTGAT
ATAGCTTTTCCACGAATAAATAATATTAGATTTTGATTACTTCCTCCATCTCTTTTAATA
TTACTTTTAAGAAATTTATTCACACCAAATGTTGGTACAGGATGAACTGTTTATCCTCCT
TTATCATCATATATATTTCATTCATCACCATCAGTTGATATTGCAATTTTTTCACTACAT
ATAACAGGTATTTCTTCAATTATTGGATCTTTAAATTTTATTGTAACTATTATATTAATA

AAAAATTATTCATTAAATTATGATCAAATTAATTTATTTTCTTGATCAGTATGTATTACA
GTAATTTTATTAATTTTATCCTTACCAGTTTTAGCAGGAGCTATTACAATACTTCTTTTT
GATCGAAATTTTAATACATCTTTTTTTGATCCAATAGGAGGAGGTGATCCTATTCTTTAT
CAACATTTATTT
>FJ582156;tax=d:Eukarya,p:Arthropoda,c:Insecta,o:Hymenoptera,f:Apidae,g:Bombus,s:Bombus vagans
GCTATATGATCAGGAATAATTGGATCATCAATAAGATTACTTATTCGAATAGAATTAAGA
CATCCTGGAATATGAATTAATAATGATCAAATTTATAATTCATTAGTTACTAGACATGCA
TTTTTAATAATTTTTTTTATAGTTATACCATTTATAATTGGAGGATTTGGAAATTATTTA
ATTCCTTTAATATTAGGAGCACCTGATATAGCTTTTCCACGAATAAATAATATTAGATTT
TGATTACTTCCTCCATCTCTTTTAATATTACTTTTAAGAAATTTATTCACACCAAATGTT
GGTACAGGATGAACTGTTTATCCTCCTTTATCATCATATATATTTCATTCATCACCATCA
GTTGATATTGCAATTTTTTCACTACATATAACAGGTATTTCTTCAATTATTGGATCTTTA
AATTTTATTGTAACTATTATATTAATAAAAAATTATTCATTAAATTATGATCAAATTAAT
TTATTTTCTTGATCAGTATGTATTACAGTAATTTTATTAATTTTATCCTTACCAGTTTTA
GCAGGAGCTATTACAATACTTCTTTTTGATCGAAATTTTAATACATCTTTTTTTGATCCA
ATAGGAGGAGGTGATCCTATTCTTTATCAACATTTATTT
>KR783791;tax=d:Eukarya,p:Arthropoda,c:Insecta,o:Hymenoptera,f:Apidae,g:Bombus,s:Bombus vagans
AATAATATATTTTATTTTTGCTATATGATCAGGAATAATTGGATCATCAATAAGATTACT
TATTCGAATAGAATTAAGACATCCTGGAATATGAATTAATAATGATCAAATTTATAATTC
ATTAGTTACTAGACATGCATTTTTAATAATTTTTTTTATAGTTATACCATTTATAATTGG
AGGATTTGGAAATTATTTAATTCCTTTAATATTAGGAGCACCTGATATAGCTTTTCCACG
AATAAATAATATTAGATTTTGATTACTTCCTCCATCTCTTTTAATATTACTTTTAAGAAA
TTTATTCACACCAAATGTTGGTACAGGATGAACTGTTTATCCTCCTTTATCATCATATAT
ATTTCATTCATCACCATCAGTTGATATTGCAATTTTTTCACTACATATAACAGGTATTTC
TTCAATTATTGGATCTTTAAATTTTATTGTAACTATTATATTAATAAAAAATTATTCATT
AAATTATGATCAAATTAATTTATTTTCTTGATCAGTATGTATTACAGTAATTTTATTAAT
TTTATCCTTACCAGTTTTAGC
>KR786681;tax=d:Eukarya,p:Arthropoda,c:Insecta,o:Hymenoptera,f:Apidae,g:Bombus,s:Bombus vagans
AATAATATATTTTATTTTTGCTATATGATCAGGAATAATTGGATCATCAATAAGATTACT
TATTCGAATAGAATTAAGACATCCTGGAATATGAATTAATAATGATCAAATTTATAATTC
ATTAGTTACTAGACATGCATTTTTAATAATTTTTTTTATAGTTATACCATTTATAATTGG
AGGATTTGGAAATTATTTAATTCCTTTAATATTAGGAGCACCTGATATAGCTTTTCCACG
AATAAATAATATTAGATTTTGATTACTTCCTCCATCTCTTTTAATATTACTTTTAAGAAA
TTTATTCACACCAAATGTTGGTACAGGATGAACTGTTTATCCTCCTTTATCATCATATAT
ATTTCATTCATCACCATCAGTTGATATTGCAATTTTTTCACTACATATAACAGGTATTTC
TTCAATTATTGGATCTTTAAATTTTATTGTAACTATTATATTAATAAAAAATTATTCATT
AAATTATGATCAAATTAATTTATTTTCTTGATCAGTATGTATTACAGTAATTTTATTAAT
TTTATCCTTACCAGTTTTAGCAGGAGCTATTACAATACTTCTTTTTGATCGAAATTTTAA
TACATCTTTTTTTGATCCAATAGGAGGAGGTGATCCT
>KR788832;tax=d:Eukarya,p:Arthropoda,c:Insecta,o:Hymenoptera,f:Apidae,g:Bombus,s:Bombus vagans
AATAATATATTTTATTTTTGCTATATGATCAGGAATAATTGGATCATCAATAAGATTACT
TATTCGAATAGAATTAAGACATCCTGGAATATGAATTAATAATGATCAAATTTATAATTC
ATTAGTTACTAGACATGCATTTTTAATAATTTTTTTTATAGTTATACCATTTATAATTGG
AGGATTTGGAAATTATTTAATTCCTTTAATATTAGGAGCACCTGATATAGCTTTTCCACG
AATAAATAATATTAGATTTTGATTACTTCCTCCATCTCTTTTAATATTACTTTTAAGAAA
TTTATTCACACCAAATGTTGGTACAGGATGAACTGTTTATCCTCCTTTATCATCATATAT
ATTTCATTCATCACCATCAGTTGATATTGCAATTTTTTCACTACATATAACAGGTATTTC
TTCAATTATTGGATCTTTAAATTTTATTGTAACTATTATATTAATAAAAAATTATTCATT
AAATTATGATCAAATTAATTTATTTTCTTGATCAGTATGTATTACAGTAATTTTATTAAT
TTTATCCTTACCAGTTTTAGCAGGAGCTATTACAATACTTCTTTTTGATCGAAATTTTAA
TACATCTTTTTTTGATCCAATAG

>KR790105;tax=d:Eukarya,p:Arthropoda,c:Insecta,o:Hymenoptera,f:Apidae,g:Bombus,s:Bombus vagans
AATAATATATTTTATTTTTGCTATATGATCAGGAATAATTGGATCATCAATAAGATTACT
TATTCGAATAGAATTAAGACATCCTGGAATATGAATTAATAATGATCAAATTTATAATTC
ATTAGTTACTAGACATGCATTTTTAATAATTTTTTTTATAGTTATACCATTTATAATTGG
AGGATTTGGAAATTATTTAATTCCTTTAATATTAGGAGCACCTGATATAGCTTTTCCACG
AATAAATAATATTAGATTTTGATTACTTCCTCCATCTCTTTTAATATTACTTTTAAGAAA
TTTATTCACACCAAATGTTGGTACAGGATGAACTGTTTATCCTCCTTTATCATCATATAT
ATTTCATTCATCACCATCAGTTGATATTGCAATTTTTTCACTACATATAACAGGTATTTC
TTCAATTATTGGATCTTTAAATTTTATTGTAACTATTATATTAATAAAAAATTATTCATT
AAATTATGATCAAATTAATTTATTTTCTTGATCAGTATGTATTACAGTAATTTTATTAAT
TTTATCCTTACCAGTTTTAGCAGG
>KR790583;tax=d:Eukarya,p:Arthropoda,c:Insecta,o:Hymenoptera,f:Apidae,g:Bombus,s:Bombus vagans
AATAATATATTTTATTTTTGCTATATGATCAGGAATAATTGGATCATCAATAAGATTACT
TATTCGAATAGAATTAAGACATCCTGGAATATGAATTAATAATGATCAAATTTATAATTC
ATTAGTTACTAGACATGCATTTTTAATAATTTTTTTTATAGTTATACCATTTATAATTGG
AGGATTTGGAAATTATTTAATTCCTTTAATATTAGGAGCACCTGATATAGCTTTTCCACG
AATAAATAATATTAGATTTTGATTACTTCCTCCATCTCTTTTAATATTACTTTTAAGAAA
TTTATTCACACCAAATGTTGGTACAGGATGAACTGTTTATCCTCCTTTATCATCATATAT
ATTTCATTCATCACCATCAGTTGATATTGCAATTTTTTCATTACATATAACAGGTATTTC
TTCAATTATTGGATCTTTAAATTTTATTGTAACTATTATATTAATAAAAAATTATTCATT
AAATTATGATCAAATTAATTTATTTTCTTGATCAGTATGTATTACAGTAATTTTATTAAT
TTTATCCTTACCAGTTTTAGCAGGAGCTATTACAATACTTCTTTTTGATCG
>KR790854;tax=d:Eukarya,p:Arthropoda,c:Insecta,o:Hymenoptera,f:Apidae,g:Bombus,s:Bombus vagans
AATAATATATTTTATTTTTGCTATATGATCAGGAATAATTGGATCATCAATAAGATTACT
TATTCGAATAGAATTAAGACATCCTGGAATATGAATTAATAATGATCAAATTTATAATTC
ATTAGTTACTAGACATGCATTTTTAATAATTTTTTTTATAGTTATACCATTTATAATTGG
AGGATTTGGAAATTATTTAATTCCTTTAATATTAGGAGCACCTGATATAGCTTTTCCACG
AATAAATAATATTAGATTTTGATTACTTCCTCCATCTCTTTTAATATTACTTTTAAGAAA
TTTATTCACACCAAATGTTGGTACAGGATGAACTGTTTATCCTCCTTTATCATCATATAT
ATTTCATTCATCACCATCAGTTGATATTGCAATTTTTTCACTACATATAACAGGTATTTC
TTCAATTATTGGATCTTTAAATTTTATTGTAACTATTATATTAATAAAAAATTATTCATT
AAATTATGATCAAATTAATTTATTTTCTTGATCAGTATGTATTACAGTAATTTTATTAAT
TTTATCCTTACCAGTTTTAGCAGGAGCTATTACAATACTTCTTTTTGATCG
>KR797768;tax=d:Eukarya,p:Arthropoda,c:Insecta,o:Hymenoptera,f:Apidae,g:Bombus,s:Bombus vagans
AATAATATATTTTATTTTTGCTATATGATCAGGAATAATTGGATCATCAATAAGATTACT
TATTCGAATAGAATTAAGACATCCTGGAATATGAATTAATAATGATCAAATTTATAATTC
ATTAGTTACTAGACATGCATTTTTAATAATTTTTTTTATAGTTATACCATTTATAATTGG
AGGATTTGGAAATTATTTAATTCCTTTAATATTAGGAGCACCTGATATAGCTTTTCCACG
AATAAATAATATTAGATTTTGATTACTTCCTCCATCTCTTTTAATATTACTTTTAAGAAA
TTTATTCACACCAAATGTTGGTACAGGATGAACTGTTTATCCTCCTTTATCATCATATAT
ATTTCATTCATCACCATCAGTTGATATTGCAATTTTTTCATTACATATAACAGGTATTTC
TTCAATTATTGGATCTTTAAATTTTATTGTAACTATTATATTAATAAAAAATTATTCATT
AAATTATGATCAAATTAATTTATTTTCTTGATCAGTATGTATTACAGTAATTTTATTAAT
TTTATCCTTACCAGTTTTAGCAGGAGCTATTACAATACTTCTTTTTGATCGAAATTTTAA
TACATCTTTTTTTGA
>KR798574;tax=d:Eukarya,p:Arthropoda,c:Insecta,o:Hymenoptera,f:Apidae,g:Bombus,s:Bombus vagans
AATAATATATTTTATTTTTGCTATATGATCAGGAATAATTGGATCATCAATAAGATTACT
TATTCGAATAGAATTAAGACATCCTGGAATATGAATTAATAATGATCAAATTTATAATTC
ATTAGTTACTAGACATGCATTTTTAATAATTTTTTTTATAGTTATACCATTTATAATTGG
AGGATTTGGAAATTATTTAATTCCTTTAATATTAGGAGCACCTGATATAGCTTTTCCACG
AATAAATAATATTAGATTTTGATTACTTCCTCCATCTCTTTTAATATTACTTTTAAGAAA

TTTATTCACACCAAATGTTGGTACAGGATGAACTGTTTATCCTCCTTTATCATCATATAT
ATTTCATTCATCACCATCAGTTGATATTGCAATTTTTTCACTACATATAACAGGTATTTC
TTCAATTATTGGATCTTTAAATTTTATTGTAACTATTATATTAATAAAAAATTATTCATT
AAATTATGATCAAATTAATTTATTTTCTTGATCAGTATGTATTACAGTAATTTTATTAAT
TTTATCCTTACCAGTTTTAGCAGGAGCTATTACAATACT
>KR799335;tax=d:Eukarya,p:Arthropoda,c:Insecta,o:Hymenoptera,f:Apidae,g:Bombus,s:Bombus vagans
ATAAGATTACTTATTCGAATAGAATTAAGACATCCTGGAATATGAATTAATAATGATCAA
ATTTATAATTCATTAGTTACTAGACATGCATTTTTAATAATTTTTTTTATAGTTATACCA
TTTATAATTGGAGGATTTGGAAATTATTTAATTCCTTTAATATTAGGAGCACCTGATATA
GCTTTTCCACGAATAAATAATATTAGATTTTGATTACTTCCTCCATCTCTTTTAATATTA
CTTTTAAGAAATTTATTCACACCAAATGTTGGTACAGGATGAACTGTTTATCCTCCTTTA
TCATCATATATATTTCATTCATCACCATCAGTTGATATTGCAATTTTTTCACTACATATA
ACAGGTATTTCTTCAATTATTGGATCTTTAAATTTTATTGTAACTATTATATTAATAAAA
AATTATTCATTAAATTATGATCAAATTAATTTATTTTCTTGATCAGTATGTATTACAGTA
ATTTTATTAATTTTATCCTTACCAGTTTTAGCAGGAGCTATTACAATACTTCTTTTTGAT
CGAAATTTTAATACATCTTTTTTTGATCCAATAGGAGGAGGTGATCCTATTCTTTATCAA
CATTTATTT
>KR800715;tax=d:Eukarya,p:Arthropoda,c:Insecta,o:Hymenoptera,f:Apidae,g:Bombus,s:Bombus vagans
AATAATATATTTTATTTTTGCTATATGATCAGGAATAATTGGATCATCAATAAGATTACT
TATTCGAATAGAATTAAGACATCCTGGAATATGAATTAATAATGATCAAATTTATAATTC
ATTAGTTACTAGACATGCATTTTTAATAATTTTTTTTATAGTTATACCATTTATAATTGG
AGGATTTGGAAATTATTTAATTCCTTTAATATTAGGAGCACCTGATATAGCTTTTCCACG
AATAAATAATATTAGATTTTGATTACTTCCTCCATCTCTTTTAATATTACTTTTAAGAAA
TTTATTCACACCAAATGTTGGTACAGGATGAACTGTTTATCCTCCTTTATCATCATATAT
ATTTCATTCATCACCATCAGTTGATATTGCAATTTTTTCACTACATATAACAGGTATTTC
TTCAATTATTGGATCTTTAAATTTTATTGTAACTATTATATTAATAAAAAATTATTCATT
AAATTATGATCAAATTAATTTATTTTCTTGATCAGTATGTATTACAGTAATTTTATTAAT
TTTATCCTTACCAGTTTTAGCAGGAGCTATTACAATACTTCTTTTTGATCGAAATTTTAA
TACATCTTTTTTTGATCCAATAGGAGGAGGTG
>KR803215;tax=d:Eukarya,p:Arthropoda,c:Insecta,o:Hymenoptera,f:Apidae,g:Bombus,s:Bombus vagans
AATAATATATTTTATTTTTGCTATATGATCAGGAATAATTGGATCATCAATAAGATTACT
TATTCGAATAGAATTAAGACATCCTGGAATATGAATTAATAATGATCAAATTTATAATTC
ATTAGTTACTAGACATGCATTTTTAATAATTTTTTTTATAGTTATACCATTTATAATTGG
AGGATTTGGAAATTATTTAATTCCTTTAATATTAGGAGCACCTGATATAGCTTTTCCACG
AATAAATAATATTAGATTTTGATTACTTCCTCCATCTCTTTTAATATTACTTTTAAGAAA
TTTATTCACACCAAATGTTGGTACAGGATGAACTGTTTATCCTCCTTTATCATCATATAT
ATTTCATTCATCACCATCAGTTGATATTGCAATTTTTTCACTACATATAACAGGTATTTC
TTCAATTATTGGATCTTTAAATTTTATTGTAACTATTATATTAATAAAAAATTATTCATT
AAATTATGATCAAATTAATTTATTTTCTTGATCAGTATGTATTACAGTAATTTTATTAAT
TTTATCCTTACCAGTTTTAGCAGGAGCTATTACAATACTTCTTTTTGATCGAAATTTTAA
TACATCTTTTTTTG
>KR803745;tax=d:Eukarya,p:Arthropoda,c:Insecta,o:Hymenoptera,f:Apidae,g:Bombus,s:Bombus vagans
AATAATATATTTTATTTTTGCTATATGATCAGGAATAATTGGATCATCAATAAGATTACT
TATTCGAATAGAATTAAGACATCCTGGAATATGAATTAATAATGATCAAATTTATAATTC
ATTAGTTACTAGACATGCATTTTTAATAATTTTTTTTATAGTTATACCATTTATAATTGG
AGGATTTGGAAATTATTTAATTCCTTTAATATTAGGAGCACCTGATATAGCTTTTCCACG
AATAAATAATATTAGATTTTGATTACTTCCTCCATCTCTTTTAATATTACTTTTAAGAAA
TTTATTCACACCAAATGTTGGTACAGGATGAACTGTTTATCCTCCTTTATCATCATATAT
ATTTCATTCATCACCATCAGTTGATATTGCAATTTTTTCACTACATATAACAGGTATTTC
TTCAATTATTGGATCTTTAAATTTTATTGTAACTATTATATTAATAAAAAATTATTCATT
AAATTATGATCAAATTAATTTATTTTCTTGATCAGTATGTATTACAGTAATTTTATTAAT

TTTATCCTTACCAGTTTTAGCAGGAGCTATTACAATACTTCTTTTTGAT
>KR807643;tax=d:Eukarya,p:Arthropoda,c:Insecta,o:Hymenoptera,f:Apidae,g:Bombus,s:Bombus vagans
AATAATATATTTTATTTTTGCTATATGATCAGGAATAATTGGATCATCAATAAGATTACT
TATTCGAATAGAATTAAGACATCCTGGAATATGAATTAATAATGATCAAATTTATAATTC
ATTAGTTACTAGACATGCATTTTTAATAATTTTTTTTATAGTTATACCATTTATAATTGG
AGGATTTGGAAATTATTTAATTCCTTTAATATTAGGAGCACCTGATATAGCTTTTCCACG
AATAAATAATATTAGATTTTGATTACTTCCTCCATCTCTTTTAATATTACTTTTAAGAAA
TTTATTCACACCAAATGTTGGTACAGGATGAACTGTTTATCCTCCTTTATCATCATATAT
ATTTCATTCATCACCATCAGTTGATATTGCAATTTTTTCATTACATATAACAGGTATTTC
TTCAATTATTGGATCTTTAAATTTTATTGTAACTATTATATTAATAAAAAATTATTCATT
AAATTATGATCAAATTAATTTATTTTCTTGATCAGTATGTATTACAGTAATTTTATTAAT
TTTATCCTTACCAGTTTTAGCAGGAGCTATTACAATACTTCTTTTTGATCGAAATTTTAA
TACATCTTTTTTTGATC
>KR896816;tax=d:Eukarya,p:Arthropoda,c:Insecta,o:Hymenoptera,f:Apidae,g:Bombus,s:Bombus vagans
AATAATATATTTTATTTTTGCTATATGATCAGGAATAATTGGATCATCAATAAGATTACT
TATTCGAATAGAATTAAGACATCCTGGAATATGAATTAATAATGATCAAATTTATAATTC
ATTAGTTACTAGACATGCATTTTTAATAATTTTTTTTATAGTTATACCATTTATAATTGG
AGGATTTGGAAATTATTTAATTCCTTTAATATTAGGAGCACCTGATATAGCTTTTCCACG
AATAAATAATATTAGATTTTGATTACTTCCTCCATCTCTTTTAATATTACTTTTAAGAAA
TTTATTCACACCAAATGTTGGTACAGGATGAACTGTTTATCCTCCTTTATCATCATATAT
ATTTCATTCATCACCATCAGTTGATATTGCAATTTTTTCATTACATATAACAGGTATTTC
TTCAATTATTGGATCTTTAAATTTTATTGTAACTATTATATTAATAAAAAATTATTCATT
AAATTATGATCAAATTAATTTATTTTCTTGATCAGTATGTATTACAGTAATTTTATTAAT
TTTATCCTTACCAGTTTTAGCAGGAGCTATTACAATACTTCTTTTTGATCGAAATTTTAA
TACATCTTTTTTTGATCCAATAGGAGGAGGTGATCCTT
>KU874451;tax=d:Eukarya,p:Arthropoda,c:Insecta,o:Hymenoptera,f:Apidae,g:Bombus,s:Bombus vagans
AATTGGATCATCAATAAGATTACTTATTCGAATAGAATTAAGACATCCTGGAATATGAAT
TAATAATGATCAAATTTATAATTCATTAGTTACTAGTCATGCATTTTTAATAATTTTTTT
TATAGTTATACCATTTATAATTGGAGGATTTGGAAATTATTTAATTCCTTTAATATTAGG
AGCTCCTGATATAGCTTTCCCACGAATAAATAATATTAGATTTTGATTACTTCCTCCATC
TCTTTTAATATTACTTTTAAGAAATTTATTTACACCAAATGTTGGTACAGGATGAACTGT
TTATCCTCCTTTATCATCTTATATATTTCATTCATCACCCTCAATTGATATTGCAATCTT
TTCTTTACATATAACAGGTATTTCTTCAATTATTGGATCTTTAAATTTTATTGTAACTAT
TATATTAATAAAAAATTTTTCATTAAATTATGATCAAATTAATTTATTTTCTTGATCAGT
ATGTATTACAGTAATTTTATTAATTTTATCTTTACCAGTTTTAGCAGGAGCTATTACTAT
ACTTCTTTTTGATCGAAATTTTAATACTTCTTTCTTTGATCCAATAGGAGGAGGTGATCC
AATTCTTTATCAACATTTATTT
>MF937270;tax=d:Eukarya,p:Arthropoda,c:Insecta,o:Hymenoptera,f:Apidae,g:Bombus,s:Bombus vagans
ATAATATATTTTATTTTTGCTATATGATCAGGAATAATTGGATCATCAATAAGATTACTT
ATTCGAATAGAATTAAGACATCCTGGAATATGAATTAATAATGATCAAATTTATAATTCA
TTAGTTACTAGACATGCATTTTTAATAATTTTTTTTATAGTTATACCATTTATAATTGGA
GGATTTGGAAATTATTTAATTCCTTTAATATTAGGAGCACCTGATATAGCTTTTCCACGA
ATAAATAATATTAGATTTTGATTACTTCCTCCATCTCTTTTAATATTACTTTTAAGAAAT
TTATTCACACCAAATGTTGGTACAGGATGAACTGTTTATCCTCCTTTATCATCATATATA
TTTCATTCATCACCATCAGTTGATATTGCAATTTTTTCACTACATATAACAGGTATTTCT
TCAATTATTGGATCTTTAAATTTTATTGTAACTATTATATTAATAAAAAATTATTCATTA
AATTATGATCAAATTAATTTATTTTCTTGATCAGTATGTATTACAGTAATTTTATTAATT
TTATCCTTACCAGTTTTAGCAGGAGCTATTACAATACTTCTTTTTGAT
>HM407358;tax=d:Eukarya,p:Arthropoda,c:Insecta,o:Hymenoptera,f:Halictidae,g:Lasioglossum,s:Lasioglossum katherineae
TATACTTTATTTTATTTTTGCTATATGAGCTGGAATAATTGGAGCTTCATTAAGAATAAT

TATTCGAATAGAACTAAGTGCTCCTGGAAAATGAATTAATAATGATCAAATTTATAACAC
TATTATTACCTCACATGCATTTGTAATAATTTTTTTTATAGTTATACCATTTATAATTGG
AGGATTTGGTAATTGATTAGTTCCTTTAATAATTGGAGCACCTGATATAGCATTCCCCCG
AATAAATAATATAAGATTTTGATTACTTATTCCATCAATATTTATATTATTAATAAGAAG
TATTATATCATCTGGTTCAGGGACTGGATGAACCGTATATCCCCCTTTATCATCTATTAT
ATACCATTCATCAATTTCAGTAGATTACACTATTTTTTCATTGCATATTGCAGGAATTTC
ATCTATTATAGGAGCTATCAACTTCATTGTATCTATTTTACTTATAAAAAATATTTCAAT
TAATTATGATCAAATTCCCTTATTCCCATGATCAGTAAAAATTACTGCTATTTTATTATT
ATTATCTCTACCAGTTTTAGCAGGAGCCATTACTATACTTTTAACAGATCGAAATTTAAA
TACTTCATTTTTTGATCCCTCAGGAGGAGGAGACCCTATTCTTTATCAACATTTATTT
>MN342305;tax=d:Eukarya,p:Arthropoda,c:Insecta,o:Hymenoptera,f:Colletidae,g:Colletes,s:Colletes eulophi
AATATTATATTTTATTTTTGCTATATGAACAGGTATAGTTGGTTCATCTTTAAGAATAAT
TATTCGAATAGAATTAAGTTCTCCTGGTATATGAATTAATAATGATCAAGTTTATAATTC
TATTGTTACAGCACATGCTTTTATTATAATTTTTTTTATAGTTATACCTTTTTTAATTGG
TGGATTTGGTAATTGATTAATTCCATTAATAATTGGAGCACCTGATATAGCATTTCCTCG
TATAAATAATATAAGTTTTTGATTATTACCTCCTTCTTTAATTTTATTATTATTAAATAG
AATTTTATATTCAGGAAGAGGAACAGGATGAACTGTTTATCCTCCTTTATCTTCTTTAAT
ATATCATCCTTCTTTATCTGTTGATTTAACAATTTTTTCTTTACATATTGCAGGAATTTC
ATCAATTATAGGATCAATAAATTTTATTGTTACAATTTTAATAATAAAAAATTATAATTT
AAATTATGATCAATTATCATTATTTTCATGATCTGTTTTTATTACTACAATTTTATTATT
ATTATCTTTACCTGTATTAGCAGGAGCAATTACAATATTATTAACTGATCGTAATTTAAA
TACTTCATTTTTTGATCCTTCTGGAGGAGGAGATCCTGTTCTTTATCAACATTTATTT
>MN342307;tax=d:Eukarya,p:Arthropoda,c:Insecta,o:Hymenoptera,f:Colletidae,g:Colletes,s:Colletes latitarsis
ATATTTTATTTTTGCTATATGAACTGGAATAATTGGTTCTTCATTAAGAATAATTATTCG
TATGGAATTAAGTTCACCAGGTATATGAATTAATAATGATCAAATTTATAATTCTATTGT
TACTTCTCATGCTTTTATTATAATTTTTTTTATAGTTATACCTTTTTTAATTGGGGGATT
TGGAAATTGATTAATTCCATTAATAATTGGTGCACCAGATATAGCATTTCCACGAATAAA
TAATATAAGATTTTGATTATTACCCCCTTCATTAATTTTATTATTAATAAGTAGTATTTT
ATATAGAGGAAGAGGAACAGGATGAACTATTTACCCTCCATTATCTTCATTAATATATCA
TTCTTCTTTATCTGTTGATTTAACTATTTTTTCTTTACATATTGCAGGTATTTCATCTAT
TATAGGATCAATAAATTTTATTGTAACAATTTTAATAATAAAAAATTATAATTTAAATTA
TGATCAATTATCTTTATTTTCATGATCTGTTTTTATTACAACAATTTTATTATTATTATC
TTTACCTGTATTAGCTGGTGCAATTACAATATTATTAACTGATCGTAATTTAAATACTTC
ATTTTTTGACCCTTCAGGTGGAGGAGATCCTGTACTTTATCAACATTTATTT
>HM422927;tax=d:Eukarya,p:Arthropoda,c:Insecta,o:Hymenoptera,f:Apidae,g:Xylocopa,s:Xylocopa virginica
TATATTATATATCATATTAGCTTTATGAGCAGGTATAATTGGTACAGCAATAAGATTTAT
TATTCGGATAGAATTAAGAATTCCTGGAAATTGAATTAATAATGATCAAATTTATAATTC
ATTAGTAACAGCTCATGCTTTTTTAATAATTTTTTTTATAGTTATACCTTTTATAATTGG
TGGTTTTGGAAATTGATTAATTCCTTTAATATTAGGATTACCTGATATAGCATTTCCTCG
AATAAATAATATTAGTTTTTGATTATTACCACCATCATTAATTTTATTATTAATTAGAAA
TTTATTTTATCCAAGACCAGGAACTGGTTGAACTATTTATCCTCCTTTATCATCATCTTT
ATATCATTCTTCACCATCAGTTGATTTAATAATTTTTTCATTACATATTTCAGGAATTTC
ATCAATTATAGGAGCAATAAATTTTATAGTAACAATTATATTAATAAAAAATATTTCAAT
AAATTATGATAAAATTAATTTATTTTCATGATCTGTATTTATTACAGCAATTTTATTATT
ATTATCATTACCAGTATTAGCTGGAGCTATTACTATATTATTATTTGATCGAAATTTTAA
TACTTCATTTTTTGATCCTATAGGAGGTGGTGATCCAATTTTATTTCAACATTTATTT
>HQ929860;tax=d:Eukarya,p:Arthropoda,c:Insecta,o:Hymenoptera,f:Apidae,g:Xylocopa,s:Xylocopa virginica
TATATTATATATCATATTAGCTTTATGAGCAGGTATAATTGGTACAGCAATAAGATTTAT
TATTCGGATAGAATTAAGAATTCCTGGAAATTGAATTAATAATGATCAAATTTATAATTC
ATTAGTAACAGCTCATGCTTTTTTAATAATTTTTTTTATAGTTATACCTTTTATAATTGG
TGGTTTTGGAAATTGATTAATTCCTTTAATATTAGGATTACCTGATATAGCATTTCCTCG

AATAAATAATATTAGTTTTTGATTATTACCACCATCATTGATTTTATTATTAATTAGAAA
TTTATTTTATCCAAGACCAGGAACTGGTTGAACTATTTATCCTCCTTTATCATCATCTTT
ATATCATTCTTCACCATCAGTTGATTTAATAATTTTTTCATTACATATTTCAGGAATTTC
ATCAATTATAGGAGCAATAAATTTTATAGTAACAATTATATTAATAAAAAATATTTCAAT
AAATTATGATAAAATTAATTTATTTTCATGATCTGTATTTATTACAGCAATTTTATTATT
ATTATCATTACCAGTATTAGCTGGAGCTATTACTATATTATTATTTGATCGAAATTTTAA
TACTTCATTTTTTGATCCTATAGGAGGTGGTGATCCAATTTTATTTCAACATTTATTT
>HQ929861;tax=d:Eukarya,p:Arthropoda,c:Insecta,o:Hymenoptera,f:Apidae,g:Xylocopa,s:Xylocopa virginica
TATATTATATATCATATTAGCTTTATGAGCAGGTATAATTGGTACAGCAATAAGATTTAT
TATTCGGATAGAATTAAGAATTCCTGGAAATTGAATTAATAATGATCAAATTTATAATTC
ATTAGTAACAGCTCATGCTTTTTTAATAATTTTTTTTATAGTTATACCTTTTATAATTGG
TGGTTTTGGAAATTGATTAATTCCTTTAATATTAGGATTACCTGATATAGCATTTCCTCG
AATAAATAATATTAGTTTTTGATTATTACCACCATCATTGATTTTATTATTAATTAGAAA
TTTATTTTATCCAAGACCAGGAACTGGTTGAACTATTTATCCTCCTTTATCATCATCTTT
ATATCATTCTTCACCATCAGTTGATTTAATAATTTTTTCATTACATATTTCAGGAATTTC
ATCAATTATAGGAGCAATAAATTTTATAGTAACAATTATATTAATAAAAAATATTTCAAT
AAATTATGATAAAATCAATTTATTTTCATGATCTGTATTTATTACAGCAATTTTATTATT
ATTATCATTACCAGTATTAGCTGGAGCTATTACTATATTATTATTTGATCGAAATTTTAA
TACTTCATTTTTTGATCCTATAGGAGGTGGTGATCCAATTTTATTTCAACATTTATTT
>EU271670;tax=d:Eukarya,p:Arthropoda,c:Insecta,o:Hymenoptera,f:Apidae,g:Xylocopa,s:Xylocopa virginica
TATATTATATATCATATTAGCTTTATGAGCAGGTATAATTGGTACAGCAATAAGATTTAT
TATTCGGATAGAATTAAGAATTCCTGGAAATTGAATTAATAATGATCAAATTTATAATTC
ATTAGTAACAGCTCATGCTTTTTTAATAATTTTTTTTATAGTTATACCTTTTATAATTGG
TGGTTTTGGAAATTGATTAATTCCTTTAATATTAGGATTACCTGATATAGCATTTCCTCG
AATAAATAATATTAGTTTTTGATTATTACCACCATCATTAATTTTATTATTAATTAGAAA
TTTATTTTATCCAAGACCAGGAACTGGTTGAACTATTTATCCTCCTTTATCATCATCTTT
ATATCATTCTTCACCATCAGTTGATTTAATAATTTTTTCATTACATATTTCAGGAATTTC
ATCAATTATAGGAGCAATAAATTTTATAGTAACAATTATATTAATAAAAAATATTTCAAT
AAATTATGATAAAATCAATTTATTTTCATGATCTGTATTTATTACAGCAATTTTATTATT
ATTATCATTACCAGTATTAGCTGGAGCTATTACTATATTATTATTTGATCGAAATTTTAA
TACTTCATTTTTTGATCCTATAGGAGGTGGTGATCCAATTTTATTTCAACATTTATTT
>GU802451;tax=d:Eukarya,p:Arthropoda,c:Insecta,o:Hymenoptera,f:Apidae,g:Xylocopa,s:Xylocopa virginica
GGTATAATTGGTACAGCAATAAGATTTATTATTCGGATAGAATTAAGAATTCCTGGAAAT
TGAATTAATAATGATCAAATTTATAATTCATTAGTAACAGCTCATGCTTTTTTAATAATT
TTTTTTATAGTTATACCTTTTATAATTGGTGGTTTTGGAAATTGATTAATTCCTTTAATA
TTAGGATTACCTGATATAGCATTTCCTCGAATAAATAATATTAGTTTTTGATTATTACCA
CCATCATTAATTTTATTATTAATTAGAAATTTATTTTATCCAAGACCAGGGACTGGTTGA
ACTATTTATCCTCCTTTATCATCATCTTTATATCATTCTTCACCATCAGTTGATTTAATA
ATTTTTTCATTACATATTTCAGGAATTTCATCAATTATAGGAGCAATAAATTTTATAGTA
ACAATTATATTAATAAAAAATATTTCAATAAATTATGACAAAATTAATTTATTTTCATGA
TCTGTGTTTATTACAGCAATTTTATTATTATTATCATTACCAGTATTAGCTGGAGCTATT
ACTATATTATTATTTGATCGAAATTTTAATACTTCATTTTTTGATCCTATAGGAGGTGGT
GATCCAATTTTATTTCAACATTTATTT
>GU802452;tax=d:Eukarya,p:Arthropoda,c:Insecta,o:Hymenoptera,f:Apidae,g:Xylocopa,s:Xylocopa virginica
TATATTATATATTATATTAGCTTTATGGGCAGGTATAATTGGTACAGCAATAAGATTTAT
TATTCGGATAGAATTAAGAATTCCTGGAAATTGAATTAATAATGATCAAATTTATAATTC
ATTAGTAACAGCTCATGCTTTTTTAATAATTTTTTTTATAGTTATACCTTTTATAATTGG
TGGTTTTGGAAATTGATTAATTCCTTTAATATTAGGATTACCTGATATAGCATTTCCTCG
AATAAATAATATTAGTTTTTGATTATTACCACCATCATTAATTTTATTATTAATTAGAAA
TTTATTTTATCCAAGACCAGGGACTGGTTGAACTATTTATCCTCCTTTATCATCATCTTT
ATATCATTCTTCACCATCAGTTGATTTAATAATTTTTTCATTACATATTTCAGGAATTTC

ATCAATTACAGGAGCAATAAATTTTATAGTAACAATTATATTAATAAAAAATATTTCAAT
AAATTATGACAAAATTAATTTATTTTCATGATCTGTGTTTATTACAGCAATTTTATTATT
ATTATCATTACCAGTATTAGCTGGAGCTATTACTATATTATTATTTGATCGAAATTTTAA
TACTTCATTTTTTGATCCTATAGGAGGTGGTGATCCAATTTTATTTCAACATTTATTT
>GU802453;tax=d:Eukarya,p:Arthropoda,c:Insecta,o:Hymenoptera,f:Apidae,g:Xylocopa,s:Xylocopa virginica
TATATTATATATTATATTAGCTTTATGGGCAGGTATAATTGGTACAGCAATAAGATTTAT
TATTCGGATAGAATTAAGAATTCCTGGAAATTGAATTAATAATGATCAAATTTATAATTC
ATTAGTAACAGCTCATGCTTTTTTAATAATTTTTTTTATAGTTATACCTTTTATAATTGG
TGGTTTTGGAAATTGATTAATTCCTTTAATATTAGGATTACCTGATATAGCATTTCCTCG
AATAAATAATATTAGTTTTTGATTATTACCACCATCATTAATTTTATTATTAATTAGAAA
TTTATTTTATCCAAGACCAGGGACTGGTTGAACTATTTATCCTCCTTTATCATCATCTTT
ATATCATTCTTCACCATCAGTTGATTTAATAATTTTTTCATTACATATTTCAGGAATTTC
ATCAATTATAGGAGCAATAAATTTTATAGTAACAATTATATTAATAAAAAATATTTCAAT
AAATTATGACAAAATTAATTTATTTTCATGATCTGTGTTTATTACAGCAATTTTATTATT
ATTATCATTACCAGTATTAGCTGGAGCTATTACTATATTATTATTTGATCGAAATTTTAA
TACTTCATTTTTTGATCCTATAGGAGGTGGTGATCCAATTTTATTTCAACATTTATTT
>HM422926;tax=d:Eukarya,p:Arthropoda,c:Insecta,o:Hymenoptera,f:Apidae,g:Xylocopa,s:Xylocopa virginica
TATATTATATATTATATTAGCTTTATGAGCAGGTATAATTGGTACAGCAATAAGATTTAT
TATTCGGATAGAATTAAGAATTCCTGGAAATTGAATTAATAATGATCAAATTTATAATTC
ATTAGTAACAGCTCATGCTTTTTTAATAATTTTTTTTATAGTTATACCTTTTATAATTGG
TGGTTTTGGAAATTGATTAATTCCTTTAATATTAGGATTACCTGATATAGCATTTCCTCG
AATAAATAATATTAGTTTTTGATTATTACCACCATCATTAATTTTATTATTAATTAGAAA
TTTATTTTATCCAAGACCAGGGACTGGTTGAACTATTTATCCTCCTTTATCATCATCTTT
ATATCATTCTTCACCATCAGTTGATTTAATAATTTTTTCATTACATATTTCAGGAATTTC
ATCAATTATAGGAGCAATAAATTTTATAGTAACAATTATATTAATAAAAAATATTTCAAT
AAATTATGACAAAATTAATTTATTTTCATGATCTGTGTTTATTACAGCAATTTTATTATT
ATTATCATTACCAGTATTAGCTGGAGCTATTACTATATTATTATTTGATCGAAATTTTAA
TACTTCATTTTTTGATCCTATAGGAGGTGGTGATCCAATTTTATTTCAACATTTATTT
>HQ567580;tax=d:Eukarya,p:Arthropoda,c:Insecta,o:Hymenoptera,f:Apidae,g:Xylocopa,s:Xylocopa virginica
TATATTATATATTATATTAGCTTTATGGGCAGGTATAATTGGTACAGCAATAAGATTTAT
TATTCGGATAGAATTAAGAATTCCTGGAAATTGAATTAATAATGATCAAATTTATAATTC
ATTAGTAACAGCTCATGCTTTTTTAATAATTTTTTTTATAGTTATACCTTTTATAATTGG
TGGTTTTGGAAATTGATTAATTCCTTTAATATTAGGATTACCTGATATAGCATTTCCTCG
AATAAATAATATTAGTTTTTGATTATTACCACCATCATTAATTTTATTATTAATTAGAAA
TTTATTTTATCCAAGACCAGGGACTGGTTGAACTATTTATCCTCCTTTATCATCATTTTT
ATATCATTCTTCACCATCAGTTGATTTAATAATTTTTTCATTACATATTTCAGGAATTTC
ATCAATTATAGGAGCAATAAATTTTATAGTAACAATTATATTAATAAAAAATATTTCAAT
AAATTATGACAAAATTAATTTATTTTCATGATCTGTGTTTATTACAGCAATTTTATTATT
ATTATCATTACCAGTATTAGCTGGAGCTATTACTATATTATTATTTGATCGAAATTTTAA
TACTTCATTTTTTGATCCTATAGGAGGTGGTGATCCAATTTTATTTCAACATTTATTT
>KJ165565;tax=d:Eukarya,p:Arthropoda,c:Insecta,o:Hymenoptera,f:Apidae,g:Xylocopa,s:Xylocopa virginica
TATATTATATATCATATTAGCTTTATGAGCAGGTATAATTGGTACAGCAATAAGATTTAT
TATTCGGATAGAATTAAGAATTCCTGGAAATTGAATTAATAATGATCAAATTTATAATTC
ATTAGTAACAGCTCATGCTTTTTTAATAATTTTTTTTATAGTTATACCTTTTATAATTGG
TGGTTTTGGAAATTGATTAATTCCTTTAATATTAGGATTACCTGATATAGCATTTCCTCG
AATAAATAATATTAGTTTTTGATTATTACCACCATCATTGATTTTATTATTAATTAGAAA
TTTATTTTATCCAAGACCAGGAACTGGTTGAACTATTTATCCTCCTTTATCATCATCTTT
ATATCATTCTTCACCATCAGTTGATTTAATAATTTTTTCATTACATATTTCAGGAATTTC
ATCAATTATAGGAGCAATAAATTTTATAGTAACAATTATATTAATAAAAAATATTTCAAT
AAATTATGATAAAATTAATTTATTTTCATGATCTGTATTTATTACAGCAATTTTATTATT
ATTATCATTGCCAGTATTAGCTGGAGCTATTACTATATTATTATTTGATCGAAATTTTAA

TACTTCATTTTTTGATCCTATAGGAGGT
>KM585613;tax=d:Eukarya,p:Arthropoda,c:Insecta,o:Hymenoptera,f:Apidae,g:Xylocopa,s:Xylocopa virginica
TATATTATATATTATATTAGCTTTATGAGCAGGTATAATTGGTACAGCAATAAGATTTAT
TATTCGGATAGAATTAAGAATTCCTGGAAATTGAATTAATAATGATCAAATTTATAATTC
ATTAGTAACAGCTCATGCTTTTTTAATAATTTTTTTTATAGTTATACCTTTTATAATTGG
TGGTTTTGGAAATTGATTAATTCCTTTAATATTAGGATTACCTGATATAGCATTTCCTCG
AATAAATAATATTAGTTTTTGATTATTACCACCATCATTGATTTTATTATTAATTAGAAA
TTTATTTTATCCAAGACCAGGAACTGGTTGAACTATTTATCCTCCTTTATCATCATCTTT
ATATCATTCTTCACCATCAGTTGATTTAATAATTTTTTCATTACATATTTCAGGAATTTC
ATCAATTATAGGAGCAATAAATTTTATAGTAACAATTATATTAATAAAAAATATTTCAAT
AAATTATGATAAAATTAATTTATTTTCATGATCTGTATTTATTACAGCAATTTTATTATT
ATTATCATTACCAGTATTAGCTGGAGCTATTACTATATTATTATTTGATCGAAATTTTAA
TACTTCATTTTTTGATCCTATAGGAGGTGGTGATCCAATTTTATTTCAACATTTATTT
>KM585614;tax=d:Eukarya,p:Arthropoda,c:Insecta,o:Hymenoptera,f:Apidae,g:Xylocopa,s:Xylocopa virginica
TATATTATATATCATATTAGCTTTATGAGCAGGTATAATTGGTACAGCAATAAGATTTAT
TATTCGGATAGAATTAAGAATTCCTGGAAATTGAATTAATAATGATCAAATTTATAATTC
ATTAGTAACAGCTCATGCTTTTTTAATAATTTTTTTTATAGTTATACCTTTTATAATTGG
TGGTTTTGGAAATTGATTAATTCCTTTAATATTAGGATTACCTGATATAGCATTTCCTCG
AATAAATAATATTAGTTTTTGATTATTACCACCATCATTGATTTTATTATTAATTAGAAA
TTTATTTTATCCAAGACCAGGAACTGGTTGAACTATTTATCCTCCTTTATCATCATCTTT
ATATCATTCTTCACCATCAGTTGATTTAATAATTTTTTCATTACATATTTCAGGAATTTC
ATCAATTATAGGAGCAATAAATTTTATAGTAACAATTATATTAATAAAAAATATTTCAAT
AAATTATGATAAAATTAATTTATTTTCATGATCTGTATTTATTACAGCAATTTTATTATT
ATTATCATTACCAGTATTAGCTGGGGCTATTACTATATTATTATTTGATCGAAATTTTAA
TACTTCATTTTTTGATCCTATAGGAGGTGGTGATCCAATTTTATTTCAACATTTATTT
>KM585624;tax=d:Eukarya,p:Arthropoda,c:Insecta,o:Hymenoptera,f:Apidae,g:Xylocopa,s:Xylocopa virginica
TATATTATATATCATATTAGCTTTATGAGCAGGTATAATTGGTACAGCAATAAGATTTAT
TATTCGGATAGAATTGAGAATTCCTGGAAATTGAATTAATAATGATCAAATTTATAATTC
ATTAGTAACAGCTCATGCTTTTTTAATAATTTTTTTTATAGTTATACCTTTTATAATTGG
TGGTTTTGGAAATTGATTAATTCCTTTAATATTAGGATTACCTGATATAGCATTTCCTCG
AATAAATAATATTAGTTTTTGATTATTACCACCATCATTGATTTTATTATTAATTAGAAA
TTTATTTTATCCAAGACCAGGAACTGGTTGAACTATTTATCCTCCTTTATCATCATCTTT
ATATCATTCTTCACCATCAGTTGATTTAATAATTTTTTCATTACATATTTCAGGAATTTC
ATCAATTATAGGAGCAATAAATTTTATAGTAACAATTATATTAATAAAAAATATTTCAAT
AAATTATGATAAAATCAATTTATTTTCATGATCTGTATTTATTACAGCAATTTTATTATT
ATTATCATTACCAGTATTAGCTGGAGCTATTACTATATTATTATTTGATCGAAATTTTAA
TACTTCATTTTTTGATCCTATAGGAGGTGGTGATCCAATTTTATTTCAACATTTATTT
>KM585625;tax=d:Eukarya,p:Arthropoda,c:Insecta,o:Hymenoptera,f:Apidae,g:Xylocopa,s:Xylocopa virginica
TATATTATATATCATATTAGCTTTATGAGCAGGTATAATTGGTACAGCAATAAGATTTAT
TATTCGGATAGAATTAAGAATTCCTGGAAATTGAATTAATAATGATCAAATTTATAATTC
ATTAGTAACAGCTCATGCTTTTTTAATAATTTTTTTTATAGTTATACCTTTTATAATTGG
TGGTTTTGGAAATTGATTAATTCCTTTAATGTTAGGATTACCTGATATAGCATTTCCTCG
AATAAATAATATTAGTTTTTGATTATTACCACCATCATTGATTTTATTATTAATTAGAAA
TTTATTTTATCCAAGACCAGGGACTGGTTGAACTATTTATCCTCCTTTATCATCATCTTT
ATATCATTCTTCACCATCAGTTGATTTAATAATTTTTTCATTACATATTTCAGGAATTTC
ATCAATTATAGGAGCAATAAATTTTATAGTAACAATTATATTAATAAAAAATATTTCAAT
AAATTATGATAAAATTAATTTATTTTCATGATCTGTATTTATTACAGCAATTTTATTATT
ATTATCATTACCAGTATTAGCTGGAGCTATTACTATATTATTATTTGATCGAAATTTTAA
TACTTCATTTTTTGATCCTATAGGAGGTGGTGATCCAATTTTATTTCAACATTTATTT
>KM585628;tax=d:Eukarya,p:Arthropoda,c:Insecta,o:Hymenoptera,f:Apidae,g:Xylocopa,s:Xylocopa virginica
TATATTATATATCATATTAGCTTTATGAGCAGGTATAATTGGTACAGCAATAAGATTTAT

TATTCGGATAGAATTAAGAATTCCTGGAAATTGAATTAATAATGATCAAATTTATAATTC
ATTAGTAACAGCTCATGCTTTTTTAATAATTTTTTTTATAGTTATACCTTTTATAATTGG
TGGTTTTGGAAATTGATTAATTCCTTTAATATTAGGATTACCTGATATAGCATTTCCTCG
AATAAATAATATTAGTTTTTGATTATTACCACCATCATTGATTTTATTATTAATTAGAAA
TTTATTTTATCCAAGACCAGGAACTGGTTGAACTATTTATCCTCCTTTATCATCATCTTT
ATATCATTCTTCACCATCAGTTGATTTAATAATTTTTTCATTACATATTTCAGGAATTTC
ATCAATTATAGGAGCAATAAATTTTATAGTAACAATTATATTAATAAAAAATATTTCAAT
AAATTATGATAAAATTAATTTATTTTCATGATCTGTATTTATTACAGCAATTTTATTGTT
ATTATCATTACCAGTATTAGCTGGAGCTATTACTATATTATTATTTGATCGAAATTTTAA
TACTTCATTTTTTGATCCTATAGGAGGTGGTGATCCAATTTTATTTCAACATTTATTT
>KM585630;tax=d:Eukarya,p:Arthropoda,c:Insecta,o:Hymenoptera,f:Apidae,g:Xylocopa,s:Xylocopa virginica
TATATTATATATCATATTAGCTTTATGAGCAGGTATAATTGGTACAGCAATAAGATTTAT
TATTCGGATAGAGTTAAGAATTCCTGGAAATTGAATTAATAATGATCAAATTTATAATTC
ATTAGTAACAGCTCATGCTTTTTTAATAATTTTTTTTATAGTTATACCTTTTATAATTGG
TGGTTTTGGAAATTGATTAATTCCTTTAATATTAGGATTACCTGATATAGCATTTCCTCG
AATAAATAATATTAGTTTTTGATTATTACCACCATCATTAATTTTATTATTAATTAGAAA
TTTATTTTATCCAAGACCAGGAACTGGTTGAACTATTTATCCTCCTTTATCATCATCTTT
ATATCATTCTTCACCATCAGTTGATTTAATAATTTTTTCATTACATATTTCAGGAATTTC
ATCAATTATAGGAGCAATAAATTTTATAGTAACAATTATATTAATAAAAAATATTTCAAT
AAATTATGATAAAATCAATTTATTTTCATGATCTGTATTTATTACAGCAATTTTATTATT
ATTATCATTACCAGTATTAGCTGGAGCTATTACTATATTATTATTTGATCGAAATTTTAA
TACTTCATTTTTTGATCCTATAGGAGGTGGTGATCCAATTTTATTTCAACATTTATTT
>KM585633;tax=d:Eukarya,p:Arthropoda,c:Insecta,o:Hymenoptera,f:Apidae,g:Xylocopa,s:Xylocopa virginica
TATATTATATATCATATTAGCTTTATGAGCAGGTATAATTGGTACAGCAATAAGATTTAT
TATTCGAATAGAATTAAGAATTCCTGGAAATTGAATTAATAATGATCAAATTTATAATTC
ATTAGTAACAGCTCATGCTTTTTTAATAATTTTTTTTATAGTTATACCTTTTATAATTGG
TGGTTTTGGAAATTGATTAATTCCTTTAATGTTAGGATTACCTGATATAGCATTTCCTCG
AATAAATAATATTAGTTTTTGATTATTACCACCATCATTGATTTTATTATTAATTAGAAA
TTTATTTTATCCAAGACCAGGAACTGGTTGAACTATTTATCCTCCTTTATCATCATCTTT
ATATCATTCTTCACCATCAGTTGATTTAATAATTTTTTCATTACATATTTCAGGAATTTC
ATCAATTATAGGAGCAATAAATTTTATAGTAACAATTATATTAATAAAAAATATTTCAAT
AAATTATGATAAAATTAATTTATTTTCATGATCTGTATTTATTACAGCAATTTTATTATT
ATTATCATTACCAGTATTAGCTGGAGCTATTACTATATTATTATTTGATCGAAATTTTAA
TACTTCATTTTTTGATCCTATAGGAGGTGGTGATCCAATTTTATTTCAACATTTATTT
>KM585636;tax=d:Eukarya,p:Arthropoda,c:Insecta,o:Hymenoptera,f:Apidae,g:Xylocopa,s:Xylocopa virginica
TATATTATATATCATATTAGCTTTATGAGCAGGTATAATTGGTACAGCAATAAGATTTAT
TATTCGGATAGAATTAAGAATTCCTGGAAATTGAATTAATAATGATCAAATTTATAATTC
ATTAGTAACAGCTCATGCTTTTTTAATAATTTTTTTTATAGTTATACCTTTTATAATTGG
TGGTTTTGGAAATTGATTAATTCCTTTAATATTAGGATTACCTGATATAGCATTTCCTCG
AATAAATAATATTAGTTTTTGATTATTACCACCATCATTGATTTTATTATTAATTAGAAA
TTTATTTTATCCAAGACCAGGAACTGGTTGAACTATCTATCCTCCTTTATCATCATCTTT
ATATCATTCTTCACCATCAGTTGATTTAATAATTTTTTCATTACATATTTCAGGAATTTC
ATCAATTATAGGAGCAATAAATTTTATAGTAACAATTATATTAATAAAAAATATTTCAAT
AAATTATGATAAAATTAATTTATTTTCATGATCTGTATTTATTACAGCAATTTTATTATT
ATTATCATTACCAGTATTAGCTGGAGCTATTACTATATTATTATTTGATCGAAATTTTAA
TACTTCATTTTTTGATCCTATAGGAGGTGGTGATCCAATTTTATTTCAACATTTATTT
>KM585637;tax=d:Eukarya,p:Arthropoda,c:Insecta,o:Hymenoptera,f:Apidae,g:Xylocopa,s:Xylocopa virginica
TATATTATATATCATATTAGCTTTATGAGCAGGTATAATTGGTACAGCAATAAGATTTAT
TATTCGGATAGAATTAAGAATTCCTGGAAATTGAATTAATAATGATCAAATTTATAATTC
ATTAGTAACAGCTCATGCTTTTTTAATAATTTTTTTTATAGTTATACCTTTTATAATTGG
TGGTTTTGGAAATTGATTAATTCCTTTAATATTAGGGTTACCTGATATAGCATTTCCTCG

AATAAATAATATTAGTTTTTGATTATTACCACCATCATTGATTTTATTATTAATTAGAAA
TTTATTTTATCCAAGACCAGGAACTGGTTGAACTATTTATCCTCCTTTATCATCATCTTT
ATATCATTCTTCACCATCAGTTGATTTAATAATTTTTTCATTACATATTTCAGGAATTTC
ATCAATTATAGGAGCAATAAATTTTATAGTAACAATTATATTAATAAAAAATATTTCAAT
AAATTATGATAAAATTAATTTATTTTCATGATCTGTATTTATTACAGCAATTTTATTATT
ATTATCATTACCAGTATTAGCTGGAGCTATTACTATATTATTATTTGATCGAAATTTTAA
TACTTCATTTTTTGATCCTATAGGAGGTGGTGATCCAATTTTATTTCAACATTTATTT
>KM585638;tax=d:Eukarya,p:Arthropoda,c:Insecta,o:Hymenoptera,f:Apidae,g:Xylocopa,s:Xylocopa virginica
TATATTATATATCATATTAGCTTTATGAGCAGGTATAATTGGTACAGCAATAAGATTTAT
TATTCGAATAGAATTAAGAATTCCTGGAAATTGAATTAATAATGATCAAATTTATAATTC
ATTAGTAACAGCTCATGCTTTTTTAATAATTTTTTTTATAGTTATACCTTTTATAATTGG
TGGTTTTGGAAATTGATTAATTCCTTTAATATTAGGATTACCTGATATAGCATTTCCTCG
AATAAATAATATTAGTTTTTGATTATTACCACCATCATTAATTTTATTATTAATTAGAAA
TTTATTTTATCCAAGACCAGGAACTGGTTGAACTATTTATCCTCCTTTATCATCATCTTT
ATATCATTCTTCACCATCAGTTGATTTAATAATTTTTTCATTACATATTTCAGGAATTTC
ATCAATTATAGGAGCAATAAATTTTATAGTAACAATTATATTAATAAAAAATATTTCAAT
AAATTATGATAAAATCAATTTATTTTCATGATCTGTATTTATTACAGCAATTTTATTATT
ATTATCATTACCAGTATTAGCCGGAGCTATTACTATATTATTATTTGATCGAAATTTTAA
TACTTCATTTTTTGATCCTATAGGAGGTGGTGATCCAATTTTATTTCAACATTTATTT
>KM585640;tax=d:Eukarya,p:Arthropoda,c:Insecta,o:Hymenoptera,f:Apidae,g:Xylocopa,s:Xylocopa virginica
TATATTATATATCATATTAGCTTTATGAGCAGGTATAATTGGTACAGCAATAAGATTTAT
TATTCGGATAGAATTAAGAATTCCTGGAAATTGAATTAATAATGATCAAATTTATAATTC
ATTAGTAACAGCTCATGCTTTTTTAATAATTTTTTTTATAGTTATACCTTTTATAATTGG
TGGTTTTGGAAATTGATTAATTCCTTTAATGTTAGGATTACCTGATATAGCATTTCCTCG
AATAAATAATATTAGTTTTTGATTATTACCACCATCATTGATTTTATTATTAATTAGAAA
TTTATTTTATCCAAGACCAGGAACTGGTTGAACTATTTATCCTCCTTTATCATCATCTTT
ATATCATTCTTCACCATCAGTTGATTTAATAATTTTTTCATTACATATTTCAGGAATTTC
ATCAATTATAGGAGCAATAAATTTTATAGTAACAATTATATTAATAAAAAATATTTCAAT
AAATTATGATAAAATTAATTTATTTTCATGATCTGTATTTATTACAGCAATTTTATTATT
ATTATCATTACCAGTATTAGCTGGAGCTATTACTATATTATTATTTGATCGAAATTTTAA
TACTTCATTTTTTGATCCTATAGGAGGTGGTGATCCAATTTTATTTCAACATTTATTT
>KM585643;tax=d:Eukarya,p:Arthropoda,c:Insecta,o:Hymenoptera,f:Apidae,g:Xylocopa,s:Xylocopa virginica
TATATTATATATCATATTAGCTTTATGAGCAGGTATAATTGGTACAGCAATAAGATTTAT
TATTCGGATAGAATTAAGAATTCCTGGAAATTGAATTAATAATGATCAAATTTATAATTC
ATTAGTAACAGCTCATGCTTTTTTAATAATTTTTTTTATAGTTATACCTTTTATAATTGG
TGGTTTTGGAAATTGATTAATTCCTTTAATATTAGGATTACCTGATATAGCATTTTCTCG
AATAAATAATATTAGTTTTTGATTATTACCACCATCATTGATTTTATTATTAATTAGAAA
TTTATTTTATCCAAGACCAGGAACTGGTTGAACTATTTATCCTCCTTTATCATCATCTTT
ATATCATTCTTCACCATCAGTTGATTTAATAATTTTTTCATTACATATTTCAGGAATTTC
ATCAATTATAGGAGCAATAAATTTTATAGTAACAATTATATTAATAAAAAATATTTCAAT
AAATTATGATAAAATTAATTTATTTTCATGATCTGTATTTATTACAGCAATTTTATTATT
ATTATCATTACCAGTATTAGCTGGAGCTATTACTATATTATTATTTGATCGAAATTTTAA
TACTTCATTTTTTGATCCTATAGGAGGTGGTGATCCAATTTTATTTCAACATTTATTT
>KM585644;tax=d:Eukarya,p:Arthropoda,c:Insecta,o:Hymenoptera,f:Apidae,g:Xylocopa,s:Xylocopa virginica
TATATTATATATCATATTAGCTTTATGAGCAGGTATAATTGGTACAGCAATAAGATTTAT
TATTCGGATAGAATTAAGAATTCCTGGAAATTGAATTAATAATGATCAAATTTATAATTC
ATTAGTAACAGCTCATGCTTTTTTAATAATTTTTTTTATAGTTATACCTTTTATAATTGG
TGGTTTTGGAAATTGATTAATTCCTTTAATATTAGGATTACCTGATATAGCATTCCCTCG
AATAAATAATATTAGTTTTTGATTATTACCACCATCATTAATTTTATTATTAATTAGAAA
TTTATTTTATCCAAGACCAGGAACTGGTTGAACTATTTATCCTCCTTTATCATCATCTTT
ATATCATTCTTCACCATCAGTTGATTTAATAATTTTTTCATTACATATTTCAGGAATTTC

ATCAATTATAGGAGCAATAAATTTTATAGTAACAATTATATTAATAAAAAATATTTCAAT
AAATTATGATAAAATCAATTTATTTTCATGATCTGTATTTATTACAGCAATTTTATTATT
ATTATCATTACCAGTATTAGCTGGAGCTATTACTATATTATTATTTGATCGAAATTTTAA
TACTTCATTTTTTGATCCTATAGGAGGTGGTGATCCAATTTTATTTCAACATTTATTT
>KM585646;tax=d:Eukarya,p:Arthropoda,c:Insecta,o:Hymenoptera,f:Apidae,g:Xylocopa,s:Xylocopa virginica
TATATTATATATCATATTAGCTTTATGAGCAGGTATGATTGGTACAGCAATAAGATTTAT
TATTCGGATAGAATTAAGAATTCCTGGAAATTGAATTAATAATGATCAAATTTATAATTC
ATTAGTAACAGCTCATGCTTTTTTAATAATTTTTTTTATAGTTATACCTTTTATAATTGG
TGGTTTTGGAAATTGATTAATTCCTTTAATATTAGGATTACCTGATATAGCATTTCCTCG
AATAAATAATATTAGTTTTTGATTATTACCACCATCATTGATTTTATTATTAATTAGAAA
TTTATTTTATCCAAGACCAGGAACTGGTTGAACTATTTATCCTCCTTTATCATCATCTTT
ATATCATTCTTCACCATCAGTTGATTTAATAATTTTTTCATTACATATTTCAGGAATTTC
ATCAATTATAGGAGCAATAAATTTTATAGTAACAATTATATTAATAAAAAATATTTCAAT
AAATTATGATAAAATTAATTTATTTTCATGATCTGTATTTATTACAGCAATTTTATTATT
ATTATCATTACCAGTATTAGCTGGAGCTATTACTATATTATTATTTGATCGAAATTTTAA
TACTTCATTTTTTGATCCTATAGGAGGTGGTGATCCAATTTTATTTCAACATTTATTT
>KM585648;tax=d:Eukarya,p:Arthropoda,c:Insecta,o:Hymenoptera,f:Apidae,g:Xylocopa,s:Xylocopa virginica
TATATTATATATCATATTAGCTTTATGAGCAGGTATAATTGGTACAGCAATAAGATTTAT
TATTCGGATAGAATTAAGAATTCCTGGAAATTGAATTAATAATGATCAAATTTATAATTC
ATTAGTAACAGCTCATGCTTTTTTAATAATTTTTTTTATAGTTATACCTTTTATAATTGG
TGGTTTTGGAAATTGATTAATTCCTTTAATATTAGGATTACCTGATATAGCATTTCCTCG
AATAAATAATATTAGTTTTTGATTATTACCACCATCATTGATTTTATTATTAATTAGAAA
TTTATTTTATCCAAGACCAGGAACTGGTTGAACTATTTATCCTCCTTTATCATCATCTTT
ATATCATTCTTCACCATCAGTTGATTTAATAATTTTTTCATTACATATTTCAGGAATTTC
ATCAATTATAGGAGCAATAAATTTTATAGTAACAATTATATTAATAAAAAATATTTCAAT
AAATTATGATAAAATTAATTTATTTTCATGATCTGTATTTATTACAGCAATTTTATTATT
ATTATCGTTACCAGTATTAGCTGGAGCTATTACTATATTATTATTTGATCGAAATTTTAA
TACTTCATTTTTTGATCCTATAGGAGGTGGTGATCCAATTTTATTTCAACATTTATTT
>KM585650;tax=d:Eukarya,p:Arthropoda,c:Insecta,o:Hymenoptera,f:Apidae,g:Xylocopa,s:Xylocopa virginica
TATATTATATATCATATTAGCTTTATGAGCAGGTATAATTGGTACAGCAATAAGATTTAT
TATTCGAATAGAATTAAGAATTCCTGGAAATTGAATTAATAATGATCAAATTTATAATTC
ATTAGTAACAGCTCATGCTTTTTTAATAATTTTTTTTATAGTTATACCTTTTATAATTGG
TGGTTTTGGAAATTGATTAATTCCTTTAATGTTAGGATTACCTGATATAGCATTTCCTCG
AATAAATAATATTAGTTTTTGATTATTACCACCATCATTGATTTTATTATTAATTAGAAA
TTTATTTTATCCAAGACCAGGAACTGGTTGAACTATTTATCCTCCTTTATCATCATCTTT
ATATCATTCTTCACCATCAGTTGATTTGATAATTTTTTCATTACATATTTCAGGAATTTC
ATCAATTATAGGAGCAATAAATTTTATAGTAACAATTATATTAATAAAAAATATTTCAAT
AAATTATGATAAAATTAATTTATTTTCATGATCTGTATTTATTACAGCAATTTTATTATT
ATTATCATTACCAGTATTAGCTGGAGCTATTACTATATTATTATTTGATCGAAATTTTAA
TACTTCATTTTTTGATCCTATAGGAGGTGGTGATCCAATTTTATTTCAACATTTATTT
>KM585651;tax=d:Eukarya,p:Arthropoda,c:Insecta,o:Hymenoptera,f:Apidae,g:Xylocopa,s:Xylocopa virginica
TATATTATATATCATATTAGCTTTATGAGCAGGTATAATTGGTACAGCAATAAGATTTAT
TATTCGGATAGAATTAAGAATTCCTGGAAATTGAATTAATAATGATCAAATTTATAATTC
ATTAGTAACAGCTCATGCTTTTTTAATAATTTTTTTTATAGTTATACCTTTTATAATTGG
TGGTTTTGGAAATTGATTAATTCCTTTAATATTAGGATTACCTGATATAGCATTTCCTCG
AATAAATAATATTAGTTTTTGATTATTACCACCATCATTGATTTTATTATTAATTAGAAA
TTTATTTTATCCAAGACCAGGAACTGGTTGAACTATTTATCCTCCTTTATCATCATCTTT
ATATCATTCTTCACCATCAGTTGATTTAATAATTTTTTCATTACATATTTCAGGAATTTC
ATCAATTATAGGAGCAATAAATTTTATAGTAACAATTATATTAATAAAAAATATTTCAAT
AAATTATGATAAAATCAATTTATTTTCATGATCTGTATTTATTACAGCAATTTTATTATT
ATTATCATTACCGGTATTAGCTGGAGCTATTACTATATTATTATTTGATCGAAATTTTAA

TACTTCATTTTTTGATCCTATAGGAGGTGGTGATCCAATTTTATTTCAACATTTATTT
>KM585653;tax=d:Eukarya,p:Arthropoda,c:Insecta,o:Hymenoptera,f:Apidae,g:Xylocopa,s:Xylocopa virginica
TATATTATATATCATATTAGCTTTATGAGCAGGTATAATTGGTACAGCAATAAGATTTAT
TATTCGAATAGAATTAAGAATTCCTGGAAATTGAATTAATAATGATCAAATTTATAATTC
ATTAGTAACAGCTCATGCTTTTTTAATAATTTTTTTTATAGTTATACCTTTTATAATTGG
TGGTTTTGGAAATTGATTAATTCCTTTAATGTTAGGATTACCTGATATAGCATTTCCTCG
AATAAATAATATTAGTTTTTGATTATTACCACCATCATTGATTTTATTATTAATTAGAAA
TTTATTTTATCCAAGACCAGGAACTGGTTGAACTATTTATCCTCCTTTATCATCATCTTT
ATATCATTCTTCACCATCAGTTGATTTAATAATTTTTTCATTACATATTTCAGGAATTTC
ATCAATTATAGGAGCAATAAATTTTATAGTAACAATTATATTAATAAAAAATATTTCAAT
AAATTATGATAAAATTAATTTATTTTCATGATCTGTATTTATTACAGCGATTTTATTATT
ATTATCATTACCAGTATTAGCTGGAGCTATTACTATATTATTATTTGATCGAAATTTTAA
TACTTCATTTTTTGATCCTATAGGAGGTGGTGATCCAATTTTATTTCAACATTTATTT
>KM585656;tax=d:Eukarya,p:Arthropoda,c:Insecta,o:Hymenoptera,f:Apidae,g:Xylocopa,s:Xylocopa virginica
TATACTATATATCATATTAGCTTTATGAGCAGGTATAATTGGTACAGCAATAAGATTTAT
TATTCGGATAGAATTAAGAATTCCTGGAAATTGAATTAATAATGATCAAATTTATAATTC
ATTAGTAACAGCTCATGCTTTTTTAATAATTTTTTTTATAGTTATACCTTTTATAATTGG
TGGTTTTGGAAATTGATTAATTCCTTTAATATTAGGATTACCTGATATAGCATTTCCTCG
AATAAATAATATTAGTTTTTGATTATTACCACCATCATTGATTTTATTATTAATTAGAAA
TTTATTTTATCCAAGACCAGGAACTGGTTGAACTATTTATCCTCCTTTATCATCATCTTT
ATATCATTCTTCACCATCAGTTGATTTAATAATTTTTTCATTACATATTTCAGGAATTTC
ATCAATTATAGGAGCAATAAATTTTATAGTAACAATTATATTAATAAAAAATATTTCAAT
AAATTATGATAAAATTAATTTATTTTCATGATCTGTATTTATTACAGCAATTTTATTATT
ATTATCATTACCAGTATTAGCTGGAGCTATTACTATATTATTATTTGATCGAAATTTTAA
TACTTCATTTTTTGATCCTATAGGAGGTGGTGATCCAATTTTATTTCAACATTTATTT
>KM585657;tax=d:Eukarya,p:Arthropoda,c:Insecta,o:Hymenoptera,f:Apidae,g:Xylocopa,s:Xylocopa virginica
TATATTATATATCATATTAGCTTTATGAGCAGGTATAATTGGTACAGCAATAAGATTTAT
TATTCGGATAGAATTAAGAATTCCTGGAAATTGAATTAATAATGATCAAATTTATAATTC
ATTAGTAACAGCTCATGCTTTTTTAATAATTTTTTTTATAGTTATACCTTTTATAATTGG
TGGTTTTGGAAATTGATTAATTCCTTTAATATTAGGATTACCTGATATAGCATTTCCTCG
AATAAATAATATTAGTTTTTGATTATTACCACCATCATTAATTTTATTATTAATTAGAAA
TTTATTTTATCCAAGACCAGGAACTGGTTGAACTATTTATCCTCCTTTATCATCATCTTT
ATATCATTCTTCACCATCAGTTGATTTAATAATTTTTTCATTACATATTTCAGGAATTTC
ATCAATTATAGGAGCAATAAATTTTATAGTAACAATTATATTAATAAAAAATATTTCAAT
AAATTATGATAAAATTAATTTATTTTCATGATCTGTATTTATTACGGCAATTTTATTATT
ATTATCATTACCAGTATTAGCTGGAGCTATTACTATATTATTATTTGATCGAAATTTTAA
TACTTCATTTTTTGATCCTATAGGAGGTGGTGATCCAATTTTATTTCAACATTTATTT
>KM585665;tax=d:Eukarya,p:Arthropoda,c:Insecta,o:Hymenoptera,f:Apidae,g:Xylocopa,s:Xylocopa virginica
TATATTATATATCATATTAGCTTTATGAGCAGGTATAATTGGTACAGCAATAAGATTTAT
TATTCGGATAGAATTAAGAATTCCTGGAAATTGAATTAATAATGATCAAATTTATAATTC
ATTAGTAACAGCTCATGCTTTTTTAATAATTTTTTTTATAGTTATACCTTTTATAATTGG
TGGTTTTGGAAATTGATTAATTCCTTTAATATTAGGATTACCTGATATAGCATTTCCTCG
AATAAATAATATTAGTTTTTGATTATTACCACCATCATTGATTTTATTATTAATTAGAAA
TTTATTTTATCCAAGACCAGGAACTGGTTGAACTATTTATCCTCCTTTATCATCATCTTT
ATATCATTCTTCACCATCAGTTGATTTAATAATTTTTTCATTACATATTTCAGGAATTTC
ATCAATTATAGGAGCAATAAATTTTATAGTAACAATTATATTAATAAAAAATATTTCAAT
AAATTATGATAAGATTAATTTATTTTCATGATCTGTATTTATTACAGCAATTTTATTATT
ATTATCATTACCAGTATTAGCTGGAGCTATTACTATATTATTATTTGATCGAAATTTTAA
TACTTCATTTTTTGATCCTATAGGAGGTGGTGATCCAATTTTATTTCAACATTTATTT
>KM585666;tax=d:Eukarya,p:Arthropoda,c:Insecta,o:Hymenoptera,f:Apidae,g:Xylocopa,s:Xylocopa virginica
TATATTATATATCATATTAGCTTTATGAGCAGGTATAATTGGTACAGCAATAAGATTTAT

TATTCGGATAGAATTAAGAATTCCTGGAAATTGAATTAATAATGATCAAATTTATAATTC
ATTAGTAACAGCTCATGCTTTTTTAATAATTTTTTTTATAGTTATACCTTTTATAATTGG
TGGTTTTGGAAATTGATTAATTCCTTTAATGTTAGGATTACCTGATATAGCATTTCCTCG
AATAAATAATATTAGTTTTTGATTATTACCACCATCATTAATTTTATTATTAATTAGAAA
TTTATTTTATCCAAGACCAGGAACTGGTTGAACTATTTATCCTCCTTTATCATCATCTTT
ATATCATTCTTCACCATCAGTTGATTTAATAATTTTTTCATTACATATTTCAGGAATTTC
ATCAATTATAGGAGCAATAAATTTTATAGTAACAATTATATTAATAAAAAATATTTCAAT
AAATTATGATAAAATTAATTTATTTTCATGATCTGTATTTATTACAGCAATTTTATTATT
ATTATCATTACCAGTATTAGCTGGAGCTATTACTATATTATTATTTGATCGAAATTTTAA
TACTTCATTTTTTGATCCTATAGGAGGTGGTGATCCAATTTTATTTCAACATTTATTT
>KM585669;tax=d:Eukarya,p:Arthropoda,c:Insecta,o:Hymenoptera,f:Apidae,g:Xylocopa,s:Xylocopa virginica
TATATTATATATCATATTAGCTTTATGAGCAGGTATAATTGGTACAGCAATAAGATTTAT
TATTCGAATAGAATTAAGAATTCCTGGAAATTGAATTAATAATGATCAAATTTATAATTC
ATTAGTAACAGCTCATGCTTTTTTAATAATTTTTTTTATAGTTATACCTTTTATAATTGG
TGGTTTTGGAAATTGATTAATTCCTTTAATATTAGGATTACCTGATATAGCATTTCCTCG
AATAAATAATATTAGTTTTTGATTATTACCACCATCATTGATTTTATTATTAATTAGAAA
TTTATTTTATCCAAGACCAGGAACTGGTTGAACTATTTATCCTCCTTTATCATCATCTTT
ATATCATTCTTCACCATCAGTTGATTTAATAATTTTTTCATTACATATTTCAGGAATTTC
ATCAATTATAGGAGCAATAAATTTTATAGTAACAATTATATTAATAAAAAATATTTCAAT
AAATTATGATAAAATTAATTTATTTTCATGATCTGTATTTATTACAGCAATTTTATTATT
ATTATCATTACCAGTATTAGCTGGAGCTATTACTATATTATTATTTGATCGAAATTTTAA
TACTTCATTTTTTGATCCTATAGGAGGTGGTGATCCAATTTTATTTCAACATTTATTT
>KM585670;tax=d:Eukarya,p:Arthropoda,c:Insecta,o:Hymenoptera,f:Apidae,g:Xylocopa,s:Xylocopa virginica
TATATTATATATCATATTAGCTTTATGAGCAGGTATAATTGGTACGGCAATAAGATTTAT
TATTCGGATAGAATTAAGAATTCCTGGAAATTGAATTAATAATGATCAAATTTATAATTC
ATTAGTAACAGCTCATGCTTTTTTAATAATTTTTTTTATAGTTATACCTTTTATAATTGG
TGGTTTTGGAAATTGATTAATTCCTTTAATATTAGGATTACCTGATATAGCATTTCCTCG
AATAAATAATATTAGTTTTTGATTATTACCACCATCATTGATTTTATTATTAATTAGAAA
TTTATTTTATCCAAGACCAGGAACTGGTTGAACTATTTATCCTCCTTTATCATCATCTTT
ATATCATTCTTCACCATCAGTTGATTTAATAATTTTTTCATTACATATTTCAGGAATTTC
ATCAATTATAGGAGCAATAAATTTTATAGTAACAATTATATTAATAAAAAATATTTCAAT
AAATTATGATAAAATTAACTTATTTTCATGATCTGTATTTATTACAGCAATTTTATTATT
ATTATCATTACCAGTATTAGCTGGAGCTATTACTATATTATTATTTGATCGAAATTTTAA
TACTTCATTTTTTGATCCTATAGGAGGTGGTGATCCAATTTTATTTCAACATTTATTT
>KM585671;tax=d:Eukarya,p:Arthropoda,c:Insecta,o:Hymenoptera,f:Apidae,g:Xylocopa,s:Xylocopa virginica
TATATTATATATCATATTAGCTTTATGAGCAGGTATAATTGGTACAGCAATAAGATTTAT
TATTCGGATAGAATTAAGAATTCCTGGAAATTGAATTAATAATGATCAAATTTATAATTC
GTTAGTAACAGCTCATGCTTTTTTAATAATTTTTTTTATAGTTATACCTTTTATAATTGG
TGGTTTTGGAAATTGATTAATTCCTTTAATATTAGGATTACCTGATATAGCATTTCCTCG
AATAAATAATATTAGTTTTTGATTATTACCACCATCATTGATTTTATTATTAATTAGAAA
TTTATTTTATCCAAGACCAGGAACTGGTTGAACTATTTATCCTCCTTTATCATCATCTTT
ATATCATTCTTCACCATCAGTTGATTTAATAATTTTTTCATTACATATTTCAGGAATTTC
ATCAATTATAGGAGCAATAAATTTTATAGTAACAATTATATTAATAAAAAATATTTCAAT
AAATTATGATAAAATTAATTTATTTTCATGATCTGTATTTATTACAGCAATTTTATTATT
ATTATCATTACCAGTATTAGCTGGAGCTATTACTATATTATTATTTGATCGAAATTTTAA
TACTTCATTTTTTGATCCTATAGGGGGTGGTGATCCAATTTTATTTCAACATTTATTT
>KM585672;tax=d:Eukarya,p:Arthropoda,c:Insecta,o:Hymenoptera,f:Apidae,g:Xylocopa,s:Xylocopa virginica
TATATTATATATCATATTAGCTTTATGAGCAGGTATAATTGGTACAGCAATAAGATTTAT
TATTCGGATAGAATTAAGAATTCCTGGAAATTGAATTAATAATGATCAAATTTATAATTC
ATTAGTAACAGCTCATGCTTTTTTAATAATTTTTTTTATAGTTATACCTTTTATAATTGG
TGGTTTTGGAAATTGATTAATTCCTTTAATATTAGGATTACCTGATATAGCATTTCCTCG

AATAAATAATATTAGTTTTTGATTATTACCACCATCATTAATTTTATTATTAATTAGAAA
TTTATTTTATCCAAGACCAGGAACTGGTTGAACTATTTATCCTCCTTTATCATCATCTTT
ATATCATTCTTCACCATCAGTTGATTTAATAATTTTTTCATTACATATTTCAGGAATTTC
ATCAATTATAGGAGCAATAAATTTTATAGTAACAATTATATTAATAAAAAATATTTCAAT
AAATTATGATAAAATCAATTTATTTTCATGATCTGTATTTATTACAGCAATTTTATTATT
ATTATCATTACCAGTATTAGCCGGAGCTATTACTATATTATTATTTGATCGAAATTTTAA
TACTTCATTTTTTGATCCTATAGGAGGTGGTGATCCAATTTTATTTCAACATTTATTT
>KM585674;tax=d:Eukarya,p:Arthropoda,c:Insecta,o:Hymenoptera,f:Apidae,g:Xylocopa,s:Xylocopa virginica
TATATTATATATCATATTAGCTTTATGAGCAGGTATAATTGGTACAGCAATAAGATTTAT
TATTCGGATAGAATTAAGAATTCCTGGAAATTGAATTAATAATGATCAAATTTATAATTC
ATTAGTAACAGCTCATGCTTTTTTAATAATTTTTTTTATAGTTATACCTTTTATAATTGG
TGGTTTTGGAAATTGATTAATTCCTTTAATATTAGGATTACCTGATATAGCATTTCCTCG
AATAAATAATATTAGTTTTTGATTATTACCACCATCATTGATTTTATTATTAATTAGAAA
TTTATTTTATCCAAGACCAGGAACTGGTTGAACTATTTACCCTCCTTTATCATCATCTTT
ATATCATTCTTCACCATCAGTTGATTTAATAATTTTTTCATTACATATTTCAGGAATTTC
ATCAATTATAGGAGCAATAAATTTTATAGTAACAATTATATTAATAAAAAATATTTCAAT
AAATTATGATAAAATTAATTTATTTTCATGATCTGTATTTATTACAGCAATTTTATTATT
ATTATCATTACCAGTATTAGCTGGAGCTATTACTATATTATTATTTGATCGAAATTTTAA
TACTTCATTTTTTGATCCTATAGGAGGTGGTGATCCAATTTTATTTCAACATTTATTT
>KM585675;tax=d:Eukarya,p:Arthropoda,c:Insecta,o:Hymenoptera,f:Apidae,g:Xylocopa,s:Xylocopa virginica
TATATTATATATCATATTAGCTTTATGAGCAGGTATAATTGGTACAGCAATAAGATTTAT
TATTCGAATAGAATTAAGAATTCCTGGAAATTGAATTAATAATGATCAAATTTATAATTC
ATTAGTAACAGCTCATGCTTTTTTAATAATTTTTTTTATAGTTATACCTTTTATAATTGG
TGGTTTTGGAAATTGATTAATTCCTTTAATGTTAGGATTACCTGATATAGCATTTCCTCG
AATAAATAATATTAGTTTTTGATTATTACCACCATCATTGATTTTATTATTAATTAGAAA
TTTATTTTATCCAAGACCAGGAACTGGTTGAACTATTTATCCTCCTTTATCATCATCTTT
ATATCATTCTTCACCATCAGTTGATTTAATAATTTTTTCATTACATATTTCAGGAATTTC
ATCAATTATAGGAGCAATAAATTTTATAGTAACAATTATATTAATAAAAAATATTTCAAT
AAATTATGACAAAATTAATTTATTTTCATGATCTGTATTTATTACAGCAATTTTATTATT
ATTATCATTACCAGTATTAGCTGGAGCTATTACTATATTATTATTTGATCGAAATTTTAA
TACTTCATTTTTTGATCCTATAGGAGGTGGTGATCCAATTTTATTTCAACATTTATTT
>KM585676;tax=d:Eukarya,p:Arthropoda,c:Insecta,o:Hymenoptera,f:Apidae,g:Xylocopa,s:Xylocopa virginica
TATATTATATATCATATTAGCTTTATGAGCAGGTATAATTGGTACAGCAATAAGATTTAT
TATTCGGATAGAATTAAGAATTCCTGGAAATTGAATTAATAATGATCAAATTTATAATTC
ATTAGTAACAGCTCATGCTTTTTTAATAATTTTTTTTATAGTTATACCTTTTATAATTGG
TGGTTTTGGAAATTGATTAATTCCTTTAATATTAGGATTACCTGATATAGCATTTCCTCG
AATAAATAATATTAGTTTTTGATTATTACCACCATCATTAATTTTATTATTAATTAGAAA
TTTATTTTATCCAAGACCAGGAACTGGTTGAACTATTTATCCTCCTTTATCATCATTTTT
ATATCATTCTTCACCATCAGTTGATTTAATAATTTTTTCATTACATATTTCAGGAATTTC
ATCAATTATAGGAGCAATAAATTTTATAGTAACAATTATATTAATAAAAAATATTTCAAT
AAATTATGATAAAATCAATTTATTTTCATGATCTGTATTTATTACAGCAATTTTATTATT
ATTATCATTACCAGTATTAGCTGGAGCTATTACTATATTATTATTTGATCGAAATTTTAA
TACTTCATTTTTTGATCCTATAGGAGGTGGTGATCCAATTTTATTTCAACATTTATTT
>KM585679;tax=d:Eukarya,p:Arthropoda,c:Insecta,o:Hymenoptera,f:Apidae,g:Xylocopa,s:Xylocopa virginica
TATATTATATATCATATTAGCTTTATGAGCAGGTATAATTGGTACAGCAATAAGATTTAT
TATTCGGATAGAATTAAGAATTCCTGGAAATTGAATTAATAATGATCAAATTTATAATTC
ATTAGTAACAGCTCATGCTTTTTTAATAATTTTTTTTATAGTTATACCTTTTATAATTGG
TGGTTTTGGAAATTGATTAATTCCTTTAATATTAGGATTACCTGATATAGCATTTCCTCG
AATAAATAATATTAGTTTTTGATTATTACCACCATCATTGATTTTATTATTAATTAGAAA
TTTATTTTATCCAAGACCAGGAACTGGTTGAACTATTTATCCTCCTTTATCATCATCTTT
ATATCATTCTTCACCATCAGTTGATTTAATAATTTTTTCATTACATATTTCAGGAATTTC

ATCAATTATAGGAGCAATAAATTTTATAGTAACAATTATATTAATAAAAAATATTTCAAT
AAATTATGATAAAATTAATTTATTTTCATGATCTGTATTTATTACAACAATTTTATTATT
ACTATCATTACCAGTATTAGCTGGAGCTATTACTATATTATTATTTGATCGAAATTTTAA
TACTTCATTTTTTGATCCTATAGGAGGTGGTGATCCAATTTTATTTCAACATTTATTT
>KM585680;tax=d:Eukarya,p:Arthropoda,c:Insecta,o:Hymenoptera,f:Apidae,g:Xylocopa,s:Xylocopa virginica
TATATTATATATCATATTAGCTTCATGAGCAGGTATAATTGGTACAGCAATAAGATTTAT
TATTCGGATAGAATTAAGAATTCCTGGAAATTGAATTAATAATGATCAAATTTATAATTC
ATTAGTAACAGCTCATGCTTTTTTAATAATTTTTTTTATAGTTATACCTTTTATAATTGG
TGGTTTTGGAAATTGATTAATTCCTTTAATATTAGGATTACCTGATATAGCATTTCCTCG
AATAAATAATATTAGTTTTTGATTATTACCACCATCATTGATTTTATTATTAATTAGAAA
TTTATTTTATCCAAGACCAGGAACTGGTTGAACTATTTATCCTCCTTTATCATCATCTTT
ATATCATTCTTCACCATCAGTTGATTTAATAATTTTTTCATTACATATTTCAGGAATTTC
ATCAATTATAGGAGCAATAAATTTTATAGTAACAATTATATTAATAAAAAATATTTCAAT
AAATTATGATAAAATTAATTTATTTTCATGATCTGTATTTATTACAGCAATTTTATTATT
ATTATCATTGCCAGTATTAGCTGGAGCTATTACTATATTATTATTTGATCGAAATTTTAA
TACTTCATTTTTTGATCCTATAGGAGGTGGTGATCCAATTTTATTTCAACATTTATTT
>KM585681;tax=d:Eukarya,p:Arthropoda,c:Insecta,o:Hymenoptera,f:Apidae,g:Xylocopa,s:Xylocopa virginica
TATATTATATATCATATTAGCTTTATGAGCAGGTATAATTGGTACAGCAATAAGATTTAT
TATTCGGATAGAATTAAGAATTCCTGGAAATTGAATTAATAATGATCAAATTTATAATTC
ATTAGTAACAGCTCATGCTTTTTTAATAATTTTTTTTATAGTTATACCTTTTATAATTGG
TGGTTTTGGAAATTGATTAATTCCTTTAATATTAGGATTACCTGATATAGCATTTCCTCG
AATAAATAATATTAGTTTTTGATTATTACCACCATCATTGATTTTATTATTAATTAGAAA
TTTATTTTATCCAAGACCAGGAACTGGTTGAACTATTTATCCTCCTTTGTCATCATCTTT
ATATCATTCTTCACCATCAGTTGATTTAATAATTTTTTCATTACATATTTCAGGAATTTC
ATCAATTATAGGAGCAATAAATTTTATAGTAACAATTATATTAATAAAAAATATTTCAAT
AAATTATGATAAAATTAATTTATTTTCATGATCTGTATTTATTACAGCAATTTTATTATT
ATTATCATTACCAGTATTAGCTGGAGCTATTACTATATTATTATTTGATCGAAATTTTAA
TACTTCATTTTTTGATCCTATAGGAGGTGGTGATCCAATTTTATTTCAACATTTATTT
>KM585683;tax=d:Eukarya,p:Arthropoda,c:Insecta,o:Hymenoptera,f:Apidae,g:Xylocopa,s:Xylocopa virginica
TATATTATATATCATATTAGCTTTATGAGCAGGTATAATTGGTACAGCAATAAGATTTAT
TATTCGGATAGAATTAAGAATTCCTGGAAATTGAATTAATAATGATCAAATTTATAATTC
ATTAGTAACAGCTCATGCTTTTTTAATAATTTTTTTTATAGTTATACCTTTTATAATTGG
TGGTTTTGGAAATTGATTAATTCCTTTAATATTAGGATTACCTGATATAGCATTTCCTCG
AATAAATAATATTAGTTTTTGATTATTACCACCATCATTGATTTTATTATTAATTAGAAA
TTTATTTTATCCAAGACCAGGAACTGGTTGAACTATTTATCCTCCTTTATCATCATCTTT
ATATCATTCTTCACCATCAGTTGATTTAATAATTTTTTCATTACATATTTCAGGAATTTC
ATCAATTATAGGAGCAATAAATTTTATAGTAACAATTATATTAATAAAAAATATTTCAAT
AAATTATGATAAAATTAACTTATTTTCATGATCTGTATTTATTACAGCAATTTTATTATT
ATTATCATTACCAGTATTAGCTGGAGCTATTACTATATTATTATTTGATCGAAATTTTAA
TACTTCATTTTTTGATCCTATAGGAGGTGGTGATCCAATTTTATTTCAACATTTATTT
>KM585684;tax=d:Eukarya,p:Arthropoda,c:Insecta,o:Hymenoptera,f:Apidae,g:Xylocopa,s:Xylocopa virginica
TATATTATATATCATATTAGCTTTATGAGCAGGTATAATTGGTACAGCAATAAGATTTAT
TATTCGGATAGAATTAAGAATTCCTGGAAATTGAATTAATAATGATCAAATTTATAATTC
ATTAGTAACAGCTCATGCTTTTTTAATAATTTTTTTTATAGTTATACCTTTTATAATTGG
TGGTTTTGGAAATTGATTAATTCCTTTAATATTAGGATTACCTGATATAGCATTTCCTCG
AATAAATAATATTAGTTTTTGATTATTACCACCATCATTGATTTTATTATTAATTAGAAA
TTTATTTTATCCAAGACCAGGAACTGGTTGAACTATTTATCCTCCTTTATCAACATCTTT
ATATCATTCTTCACCATCAGTTGATTTAATAATTTTTTCATTACATATTTCAGGAATTTC
ATCAATTATAGGAGCAATAAATTTTATAGTAACAATTATATTAATAAAAAATATTTCAAT
AAATTATGATAAAATCAATTTATTTTCATGATCTGTATTTATTACAGCAATTTTATTATT
ATTATCATTACCAGTATTAGCTGGAGCTATTACTATATTATTATTTGATCGAAATTTTAA

TACTTCATTTTTTGATCCTATAGGAGGTGGTGATCCAATTTTATTTCAACATTTATTT
>KR791509;tax=d:Eukarya,p:Arthropoda,c:Insecta,o:Hymenoptera,f:Apidae,g:Xylocopa,s:Xylocopa virginica
TATATTATATATCATATTAGCTTTATGAGCAGGTATAATTGGTACAGCAATAAGATTTAT
TATTCGGATAGAATTAAGAATTCCTGGAAATTGAATTAATAATGATCAAATTTATAATTC
ATTAGTAACAGCTCATGCTTTTTTAATAATTTTTTTTATAGTTATACCTTTTATAATTGG
TGGTTTTGGAAATTGATTAATTCCTTTAATATTAGGATTACCTGATATAGCATTTCCTCG
AATAAATAATATTAGTTTTTGATTATTACCACCATCATTGATTTTATTATTAATTAGAAA
TTTATTTTATCCAAGACCAGGAACTGGTTGAACTATTTATCCTCCTTTATCATCATCTTT
ATATCATTCTTCACCATCAGTTGATTTAATAATTTTTTCATTACATATTTCAGGAATTTC
ATCAATTATAGGAGCAATAAATTTTATAGTAACAATTATATTAATAAAAAATATTTCAAT
AAATTATGATAAAATTAATTTATTTTCATGATCTGTTTTTATTACAGCAATTTTATTATT
ATTATCATTACCAGTATTAGCTGGAGCTATTACTATATTATTATTTGATCGAAATTTTAA
TACTTCATTTTTTGATCCTATAGGAGGTGGTGATCCAATTTTATTTCAACATTTATTT
>KR793949;tax=d:Eukarya,p:Arthropoda,c:Insecta,o:Hymenoptera,f:Apidae,g:Xylocopa,s:Xylocopa virginica
TAATGATCAAATTTATAATTCATTAGTAACAGCTCATGCTTTTTTAATAATTTTTTTTAT
AGTTATACCTTTTATAATTGGTGGTTTTGGAAATTGATTAATTCCTTTGATAGTAGGATT
ACCTGATATAGCATTTCCTCGAATAAATAATATTAGTTTTTGATTATTACCACCATCATT
GATTTTATTATTAATTAGAAATTTATTTTATCCAAGACCAGGAACTGGTTGAACTATTTA
TCCTCCTTTATCATCATCTTTATATCATTCTTCACCATCAGTTGATTTAATAATTTTTTC
ATTACATATTTCAGGAATTTCATCAATTATAGGAGCAATAAATTTTATAGTAACAATTAT
ATTAATAAAAAATATTTCAATAAATTATGATAAAATTAATTTATTTTCATGATCTGTATT
TATTACAGCAATTTTATTATTATTATCATTGCCAGTATTAGCTGGAGCTATTACTATATT
ATTATTTGATCGAAATTTTAATACTTCATTTTTTGATCCTATAGGAGGTGGTGATCCAAT
TTTATTTCAACATTTATTT
>KR883838;tax=d:Eukarya,p:Arthropoda,c:Insecta,o:Hymenoptera,f:Apidae,g:Xylocopa,s:Xylocopa virginica
TATATTATATATCATATTAGCTTTATGAGCAGGTATAATTGGTACAGCAATAAGATTTAT
TATTCGGATAGAATTAAGAATTCCTGGAAATTGAATTAATAATGATCAAATTTATAATTC
ATTAGTAACAGCTCATGCTTTTTTAATAATTTTTTTTATAGTTATACCTTTTATAATTGG
TGGTTTTGGAAATTGATTAATTCCTTTAATATTAGGATTACCTGATATAGCATTTCCTCG
AATAAATAATATTAGTTTTTGATTATTACCACCATCATTAATTTTATTATTAATTAGAAA
TTTATTTTATCCAAGACCAGGAACTGGTTGAACTATTTATCCTCCTTTATCATCATCTTT
ATATCATTCTTCACCATCAGTTGATTTAATAATTTTTTCATTACATATTTCAGGAATTTC
ATCAATTATAGGAGCAATAAATTTTATAGTAACAATTATATTAATAAAAAATATTTCAAT
AAATTATGATAAAATCAATTTATTTTCATGATCTGTATTTATTACAGCAATTTTATTATT
ATTATCATTACCAGTATTAGCTGGAGCTATTACTATATTATTATTTGATCGAAACTTTAA
TACTTCATTTTTTGATCCTATAGGAGGTGGTGATCCAATTTTATTTCAACATTTATTT
>KR898114;tax=d:Eukarya,p:Arthropoda,c:Insecta,o:Hymenoptera,f:Apidae,g:Xylocopa,s:Xylocopa virginica
TATATTATATATCATATTAGCTTTATGAGCAGGTATAATTGGTACAGCAATAAGATTTAT
TATTCGGATAGAATTAAGAATTCCTGGAAATTGAATTAATAATGATCAAATTTATAATTC
ATTAGTAACAGCTCATGCTTTTTTAATAATTTTTTTTATAGTTATACCTTTTATAATTGG
TGGTTTTGGAAATTGATTAATTCCTTTAATATTAGGATTACCTGATATAGCATTTCCTCG
AATAAATAATATTAGTTTTTGATTATTACCACCATCATTGATTTTATTATTAATTAGAAA
TTTATTTTATCCAAGACCAGGAACTGGTTGAACTATTTATCCTCCTTTATCATCATCTTT
ATATCATTCTTCACCATCAGTTGATTTAATAATTTTTTCATTACATATTTCAGGAATTTC
ATCAATTATAGGAGCAATAAATTTTATAGTAACAATTATATTAATAAAAAATATTTCAAT
AAATTATGATAAAATTAATTTATTTTCATGATCTGTATTTATTACAGCAATTTTATTATT
ATTATCATTGCCAGTATTAGCTGGAGCTATTACTATATTATTATTTGATCGAAATTTTAA
TACTTCATTTTTTGATCCTATAGGAGGTGGTGATCCAATTTTATTTCAACATTTATTT
>MG439221;tax=d:Eukarya,p:Arthropoda,c:Insecta,o:Hymenoptera,f:Apidae,g:Xylocopa,s:Xylocopa virginica
ATATTATATATCATATTAGCTTTATGAGCAGGTATAATTGGTACAGCAATAAGATTTATT
ATTCGGATAGAATTAAGAATTCCTGGAAATTGAATTAATAATGATCAAATTTATAATTCA

TTAGTAACAGCTCATGCTTTTTTAATAATTTTTTTTATAGTTATACCTTTTATAATTGGT
GGTTTTGGAAATTGATTAATTCCTTTAATATTAGGATTACCTGATATAGCATTTCCTCGA
ATAAATAATATTAGTTTTTGATTATTACCACCATCATTGATTTTATTATTAATTAGAAAT
TTATTTTATCCAAGACCAGGAACTGGTTGAACTATTTATCCTCCTTTATCATCATCTTTA
TATCATTCTTCACCATCAGTTGATTTAATAATTTTTTCATTACATATTTCAGGAATTTCA
TCAATTATAGGAGCAATAAATTTTATAGTAACAATTATATTAATAAAAAATATTTCAATA
AATTATGATAAAATTAATTTATTTTCATGATCTGTATTTATTACAGCAATTTTATTATTA
TTATCATTGCCAGTATTAGCTGGAGCTATTACTATATTATTATTTGAT
>MG439503;tax=d:Eukarya,p:Arthropoda,c:Insecta,o:Hymenoptera,f:Apidae,g:Xylocopa,s:Xylocopa virginica
TATATTATATATCATATTAGCTTTATGAGCAGGTATAATTGGTACAGCAATAAGATTTAT
TATTCGGATAGAATTAAGAATTCCTGGAAATTGAATTAATAATGATCAAATTTATAATTC
ATTAGTAACAGCTCATGCTTTTTTAATAATTTTTTTTATAGTTATACCTTTTATAATTGG
TGGTTTTGGAAATTGATTAATTCCTTTAATATTAGGATTACCTGATATAGCATTTCCTCG
AATAAATAATATTAGTTTTTGATTATTACCACCATCATTGATTTTATTATTAATTAGAAA
TTTATTTTATCCAAGACCAGGAACTGGTTGAACTATTTATCCTCCTTTATCATCATCTTT
ATATCATTCTTCACCATCAGTTGATTTAATAATTTTTTCATTACATATTTCAGGAATTTC
ATCAATTATAGGAGCAATAAATTTTATAGTAACAATTATATTAATAAAAAATATTTCAAT
AAATTATGATAAAATTAATTTATTTTCATGATCTGTATTTATTACAGC
>MG441286;tax=d:Eukarya,p:Arthropoda,c:Insecta,o:Hymenoptera,f:Apidae,g:Xylocopa,s:Xylocopa virginica
ATATTATATATCATATTAGCTTTATGAGCAGGTATAATTGGTACAGCAATAAGATTTATT
ATTCGGATAGAATTAAGAATTCCTGGAAATTGAATTAATAATGATCAAATTTATAATTCA
TTAGTAACAGCTCATGCTTTTTTAATAATTTTTTTTATAGTTATACCTTTTATAATTGGT
GGTTTTGGAAATTGATTAATTCCTTTAATATTAGGATTACCTGATATAGCATTTCCTCGA
ATAAATAATATTAGTTTTTGATTATTACCACCATCATTAATTTTATTATTAATTAGAAAT
TTATTTTATCCAAGACCAGGAACTGGTTGAACTATTTATCCTCCTTTATCATCATCTTTA
TATCATTCTTCACCATCAGTTGATTTAATAATTTTTTCATTACATATTTCAGGAATTTCA
TCAATTATAGGAGCAATAAATTTTATAGTAACAATTATATTAATAAAAAATATTTCAATA
AATTATGATAAAATCAATTTATTTTCATGATCTGTATTTATTACAGCAATTTTATTATTA
TTATCATTA
>MG445458;tax=d:Eukarya,p:Arthropoda,c:Insecta,o:Hymenoptera,f:Apidae,g:Xylocopa,s:Xylocopa virginica
TGAGCAGGTATAATTGGTACAGCAATAAGATTTATTATTCGGATAGAATTAAGAATTCCT
GGAAATTGAATTAATAATGATCAAATTTATAATTCATTAGTAACAGCTCATGCTTTTTTA
ATAATTTTTTTTATAGTTATACCTTTTATAATTGGTGGTTTTGGAAATTGATTAATTCCT
TTAATATTAGGATTACCTGATATAGCATTTCCTCGAATAAATAATATTAGTTTTTGATTA
TTACCACCATCATTGATTTTATTATTAATTAGAAATTTATTTTATCCAAGACCAGGAACT
GGTTGAACTATTTATCCTCCTTTATCATCATCTTTATATCATTCTTCACCATCAGTTGAT
TTAATAATTTTTTCATTACATATTTCAGGAATTTCATCAATTATAGGAGCAATAAATTTT
ATAGTAACAATTATATTAATAAAAAATATTTCAATAAATTATGATAAAATTAATTTATTT
TCATGATCTGTATTTATTACAGCAATTTTATTATTATTATCATTGCCAGTATTAGCTGGA
GCTATTACTATATTATTATTTGATCGAAATTTTAATACTTCATTTTTTGATCCTATAGGA
GGTGGTGATCCAATTTTATTTCAACATTTATTT
>MG446006;tax=d:Eukarya,p:Arthropoda,c:Insecta,o:Hymenoptera,f:Apidae,g:Xylocopa,s:Xylocopa virginica
TGAGCAGGTATAATTGGTACAGCAATAAGATTTATTATTCGGATAGAATTAAGAATTCCT
GGAAATTGAATTAATAATGATCAAATTTATAATTCATTAGTAACAGCTCATGCTTTTTTA
ATAATTTTTTTTATAGTTATACCTTTTATAATTGGTGGTTTTGGAAATTGATTAATTCCT
TTAATATTAGGATTACCTGATATAGCATTTCCTCGAATAAATAATATTAGTTTTTGATTA
TTACCACCATCATTGATTTTATTATTAATTAGAAATTTATTTTATCCAAGACCAGGAACT
GGTTGAACTATTTATCCTCCTTTATCATCATCTTTATATCATTCTTCACCATCAGTTGAT
TTAATAATTTTTTCATTACATATTTCAGGAATTTCATCAATTATAGGAGCAATAAATTTT
ATAGTAACAATTATATTAATAAAAAATATTTCAATAAATTATGATAAAATTAATTTATTT
TCATGATCTGTATTTATTACAGCAATTTTATTATTATTATCATTGCCAGTATTAGCTGGA

GCTATTACTATATTATTATTTGATCGAAATTTTAATACTTCATTTTTTGATCCTATAGGA
GGTGGTGATCCAATTTTATTTCAACATTTA
>MG447911;tax=d:Eukarya,p:Arthropoda,c:Insecta,o:Hymenoptera,f:Apidae,g:Xylocopa,s:Xylocopa virginica
TTGAGCAGGTATAATTGGTACAGCAATAAGATTTATTATTCGGATAGAATTAAGAATTCC
TGGAAATTGAATTAATAATGATCAAATTTATAATTCATTAGTAACAGCTCATGCTTTTTT
AATAATTTTTTTTATAGTTATACCTTTTATAATTGGTGGTTTTGGAAATTGATTAATTCC
TTTAATATTAGGATTACCTGATATAGCATTTCCTCGAATAAATAATATTAGTTTTTGATT
ATTACCACCATCATTAATTTTATTATTAATTAGAAATTTATTTTATCCAAGACCAGGAAC
TGGTTGAACTATTTATCCTCCTTTATCATCATCTTTATATCATTCTTCACCATCAGTTGA
TTTAATAATTTTTTCATTACATATTTCAGGAATTTCATCAATTATAGGAGCAATAAATTT
TATAGTAACAATTATATTAATAAAAAATATTTCAATAAATTATGATAAAATCAATTTATT
TTCATGATCTGTATTTATTACAGCAATTTTATTATTATTATCATTACCAGTATTAGCTGG
AGCTATTACTATATTATTATTTGATCGAAATTTTAATACTTCATTTTTTGATCCTATAGG
AGGTGGTGATCCAATTTTATTTCAACATTTA
>KC853361;tax=d:Eukarya,p:Arthropoda,c:Insecta,o:Hymenoptera,f:Apidae,g:Bombus,s:Bombus pensylvanicus
TAAGTTTATTAATTCGAATAGAATTAAGACATCCTGGAATATGAATTAATAATGATCAAA
TTTATAATTCTTTAGTTACTAGTCATGCATTTTTAATAATTTTTTTTATAGTTATACCAT
TCTTAATTGGAGGATTTGGAAATTATTTAATTCCTTTAATATTAGGATCTCCTGATATAG
CTTTTCCTCGAATAAATAATATTAGATTTTGATTACTTCCTCCTTCATTAATATTATTAT
TATTAAGAAATTTATTTTCACCTAATGCTGGAACTGGATGAACTGTATATCCTCCTTTAT
CAACTTATTTATTTCATTCATCTCCATCAGTTGATATCGCAATTTTTTCTTTACATATAA
CAGGAATTTCTTCAATTATTGGTTCTTTAAATTTTATTGTTACTATTATAATAATAAAAA
ATTATTCTTTAAATTATGATCAAATTAATTTATTTTCATGATCAGTTTGTATTACAGTTA
TTTTATTAATTTTGTCTTTACCTGTATTAGCAGGAGCAATTACTATATTACTTTTTGATC
GAAATTTTAATACATCTTTTTTTGATCCAATAGGAGGAGGGGATCCAATTCTTTATCAAC
ATTTATTT
>MG279447;tax=d:Eukarya,p:Arthropoda,c:Insecta,o:Hymenoptera,f:Apidae,g:Bombus,s:Bombus pensylvanicus
ATAATATATTTTATTTTTGCTATATGATCTGGAATAATTGGTTCATCAATAAGATTATTA
ATTCGAATAGAACTAAGACATCCTGGAATATGAATCAATAATGATCAAATTTATAATTCT
TTAGTTACTAGTCATGCATTTTTAATAATTTTTTTTATAGTTATACCATTTTTAATTGGA
GGATTTGGAAATTATTTAATTCCTTTAATATTAGGATCTCCTGATATAGCATTTCCTCGA
ATAAATAATATTAGATTTTGATTACTTCCTCCTTCATTAATATTATTATTATTAAGAAAT
ATATTTACACCTAATGTTGGAACAGGTTGAACTGTTTATCCTCCTTTATCATCTTATTTA
TTTCATTCATCTCCATCTGTAGATATTGCAATTTTTTCTTTACATATAACAGGAATTTCT
TCAATTATTGGTTCTTTAAATTTTATTGTTACTATTATAATAATAAAAAATTATTCTTTA
AGTTATGATCAAATTAATCTATTTTCATGATCAGTTTGTATTACAGTTATTTTATTAATT
TTGTCTTTACCTGTATTAGCAGGAGCAATTACTATATTACTTTTTGATCGAAATTTTAAT
ACATCTTTTTTTGATCCAATAGGAGGAGGAGATCCAATTCTTTATCAACATTTA
>GU707738;tax=d:Eukarya,p:Arthropoda,c:Insecta,o:Hymenoptera,f:Apidae,g:Bombus,s:Bombus affinis
AATAATATATTTTATTTTTGCTATATGATCAGGAATAATTGGTTCATCAATAAGTTTATT
AGTTCGAATAGAATTAAGTCATCCAGGAATATGAATTAATAATGATCAAATTTATAATTC
TTTAGTAACTAGACATGCATTTTTAATAATTTTTTTTATAGTTATACCATTTATAATTGG
AGGATTTGGTAATTACTTAATTCCATTAATATTAGGATCACCAGATATAGCTTTTCCTCG
AATAAATAATATTAGATTTTGACTATTACCCCCATCATTATTTATATTATTATTAAGAAA
TTTATTTACACCTAATGTAGGAACAGGATGAACTATTTATCCCCCTTTATCTTCTTATTT
ATTTCATTCTTCACCATCAATTGATATTGCAATCTTCTCATTGCATATATCAGGAATTTC
TTCTATTATTGGATCATTAAATTTTATTGTAACTATTTTATTAATAAAAAATTTTTCATT
AAATTATGATCAAATTAATTTATTCTCATGATCAGTATGTATTACTGTAATTTTATTAAT
TTTATCTCTACCAGTATTAGCTGGAGCAATTACAATATTACTTTTTGATCGAAATTTTAA
TACCTCATTTTTTGACCCTATAGGAGGAGGAGACCCAATTTTATATCAACACCTATTT
>JQ843369;tax=d:Eukarya,p:Arthropoda,c:Insecta,o:Hymenoptera,f:Apidae,g:Bombus,s:Bombus affinis

TGGTTCATCAATAAGTTTATTAGTTCGAATAGAATTAAGTCATCCAGGAATATGAATTAA
TAATGATCAAATTTATAATTCTTTAGTAACTAGACATGCATTTTTAATAATTTTTTTTAT
AGTTATACCATTTATAATTGGAGGATTTGGTAATTACTTAATTCCATTAATATTAGGATC
ACCAGATATAGCTTTTCCTCGAATAAATAATATTAGATTTTGACTATTACCCCCATCATT
ATTTATATTATTATTAAGAAATTTATTTACACCTAATGTAGGAACAGGATGAACTATTTA
TCCCCCTTTATCTTCTTATTTATTTCATTCTTCACCATCAATTGATATTGCAATCTTCTC
ATTGCATATATCAGGAATTTCTTCTATTATTGGATCATTAAATTTTATTGTAACTATTTT
ATTAATAAAAAATTTTTCATTAAATTATGATCAAATTAATTTATTCTCATGATCAGTATG
TATTACTGTAATTTTATTAATTTTATCTCTACCAGTATTAGCTGGAGCAATTACAATATT
ACTTTTTGATCGAAATTTTAATACCTCATTTTTTGACCCTATAGGAGGAGGAGACCCAAT
TTTATATCAACACCTATTT
>KM585615;tax=d:Eukarya,p:Arthropoda,c:Insecta,o:Hymenoptera,f:Apidae,g:Bombus,s:Bombus auricomus
AATATTATATTTTATTTTTGCTATATGATCAGGAATAATTGGTTCTTCCATAAGTTTATT
AATTCGAATAGAATTAAGAACTCCTGGAATATGAATTAATAATGATCAAATTTATAATTC
TTTAGTAACAAGACATGCTTTTTTAATAATTTTTTTTATAGTTATACCTTTCATAATTGG
TGGATTTGGAAATTATTTAATTCCTTTAATATTAGGATCACCAGATATAGCTTTCCCTCG
AATAAATAATATTAGATTTTGACTTTTACCTCCTTCATTATTTATATTATTATTAAGAAC
TTTATATTATCCAAATGTAGGAACAGGATGAACAGTTTATCCACCATTATCTTCTTATAT
ATTTCATTCATCCCCTTCTGTAGATATCGCAATTTTTTCATTACATATAACAGGAATTTC
TTCAATTATTGGATCATTAAATTTTATTGTTACAATTCTATTAATAAAAAATTTTTCATT
AAATTATGATCAAATTAATTTATTTTCTTGATCAGTATGTATTACAGTAATTTTATTAAT
ATTATCACTTCCAGTATTAGCAGGTGCAATTACTATACTTCTTTTTGATCGAAATTTTAA
TACTTCATTTTTTGATCCAATAGGAGGAGGAGATCCAATTCTTTATCAACATTTATTT
>HQ929825;tax=d:Eukarya,p:Arthropoda,c:Insecta,o:Hymenoptera,f:Apidae,g:Bombus,s:Bombus bimaculatus
AATAATATATTTTATTTTTGCTATATGATCAGGAATAATTGGATCATCAATAAGACTATT
AATTCGAATAGAACTTAGTCATCCTGGAATATGAATTAATAATGATCAAATTTATAATTC
ATTAGTTACAAGTCATGCATTTTTAATAATTTTTTTTATAGTAATACCATTTATAATTGG
AGGATTTGGAAATTATTTAATTCCTTTAATATTAGGATCACCTGACATAGCTTTTCCTCG
AATAAATAATATTAGATTTTGATTACTACCTCCATCTCTTTTTATATTACTTTTAAGAAT
ATTATTTACACCAAATGCAGGAACAGGTTGAACTGTATATCCTCCTTTATCATCATATAT
ATTTCATTCATCACCATCTATTGATATTGCAATTTTTTCCTTACATATAACAGGAATTTC
CTCAATTATTGGATCTTTAAATTTTATTGTAACTATTATACTAATAAAAAATTTTTCATT
AAATTATGACCAAATTAATTTATTCTCTTGATCTGTATGTATTACAGTAATATTATTAAT
TTTATCCTTACCAGTTTTAGCAGGAGCAATTACTATACTTCTTTTTGATCGAAATTTCAA
TACATCATTTTTTGATCCAATAGGAGGAGGTGATCCAATTCTTTATCAACATTTATTT
>HM422929;tax=d:Eukarya,p:Arthropoda,c:Insecta,o:Hymenoptera,f:Apidae,g:Bombus,s:Bombus bimaculatus
AATAATATATTTTATTTTTGCTATATGATCAGGAATAATTGGATCATCAATAAGACTATT
AATTCGAATAGAACTTAGTCATCCTGGAATATGAATTAATAATGATCAAATTTATAATTC
ATTAGTTACAAGTCATGCATTTTTAATAATTTTTTTTATAGTAATACCATTTATAATTGG
AGGATTTGGAAATTATTTAATTCCTTTAATATTAGGATCACCTGACATAGCTTTTCCTCG
AATAAATAATATTAGATTTTGATTACTACCTCCATCTCTTTTTATATTACTTTTAAGAAT
ATTATTTACACCAAATGCAGGAACAGGTTGAACTGTATATCCTCCTTTATCATCATATAT
ATTTCATTCATCACCATCTATTGATATTGCAATTTTTTCCTTACATATAACAGGAATTTC
TTCAATTATTGGATCTTTAAATTTTATTGTAACTATTATATTAATAAAAAATTTTTCATT
AAATTATGACCAAATTAATTTATTCTCTTGATCTGTATGTATTACAGTAATATTATTAAT
TTTATCCTTACCAGTTTTAGCAGGAGCAATTACTATACTTCTTTTTGATCGAAATTTCAA
TACATCATTTTTTGATCCAATAGGAGGAGGTGATCCAACTCTTTATCAACATTTATTT
>KM585629;tax=d:Eukarya,p:Arthropoda,c:Insecta,o:Hymenoptera,f:Apidae,g:Bombus,s:Bombus bimaculatus
AATAATATACTTTATTTTTGCTATATGATCAGGAATAATTGGATCATCAATAAGATTATT
AATTCGAATAGAACTTAGTCATCCTGGAATATGAATTAATAATGATCAAATTTACAATTC
ATTAGTTACAAGTCATGCATTTTTAATAATTTTTTTTATAGTAATACCATTTATAATTGG

AGGATTTGGAAATTATTTAATTCCTTTAATATTAGGATCACCTGACATAGCTTTTCCTCG
AATAAATAATATTAGATTTTGATTACTACCTCCATCTCTCTTTATATTACTTTTAAGAAC
ATTATTTACACCAAATGCAGGAACAGGTTGAACTGTATATCCACCTTTATCATCATATAT
ATTTCATTCATCACCATCTATTGATATTGCAATTTTTTCATTACATATAACAGGAATTTC
TTCAATTATTGGATCTTTAAATTTTATTGTAACTATTATATTAATAAAAAATTTTTCATT
AAATTATGATCAAATTAATTTATTTTCTTGATCTGTATGTATTACAGTAATATTATTAAT
TTTATCTTTACCAGTTTTAGCAGGAGCAATTACTATACTTCTTTTTGATCGAAATTTTAA
TACATCATTTTTTGATCCAATAGGAGGAGGTGATCCAATTCTTTACCAACATTTATTT
>KR883869;tax=d:Eukarya,p:Arthropoda,c:Insecta,o:Hymenoptera,f:Apidae,g:Bombus,s:Bombus bimaculatus
ATAATATACTTTATTTTTGCTATATGATCAGGAATAATTGGATCATCAATAAGATTATTA
ATTCGAATAGAACTTAGTCATCCTGGAATATGAATTAATAATGATCAAATTTACAATTCA
TTAGTTACAAGTCATGCATTTTTAATAATTTTTTTTATAGTAATACCATTTATAATTGGA
GGATTTGGAAATTATTTAATTCCTTTAATATTAGGATCACCTGATATAGCTTTTCCTCGA
ATAAATAATATTAGATTTTGATTACTACCCCCATCTCTCTTTATATTACTTTTAAGAACA
TTATTTACACCAAATACAGGAACAGGTTGAACTGTATATCCACCTTTATCATCATATATA
TTTCATTCATCACCATCTATTGATATTGCAATTTTTTCATTACATATAACAGGAATTTCT
TCAATTATTGGATCTTTAAATTTTATTGTAACTATTATATTAATAAAAAATTTTTCATTA
AATTATGATCAAATTAACTTATTTTCCTGATCTGTATGTATTACAGTAATATTATTAATT
TTATCTTTACCAGTTTTAGCA
>KR887497;tax=d:Eukarya,p:Arthropoda,c:Insecta,o:Hymenoptera,f:Apidae,g:Bombus,s:Bombus bimaculatus
ATAATATACTTTATTTTTGCTATATGATCAGGAATAATTGGATCATCAATAAGATTATTA
ATTCGAATAGAACTTAGTCATCCTGGAATATGAATTAATAATGATCAAATTTACAATTCA
TTAGTTACAAGTCATGCATTTTTAATAATTTTTTTTATAGTAATACCATTTATAATTGGA
GGATTTGGAAATTATTTAATTCCTTTAATATTAGGATCACCTGATATAGCTTTTCCTCGA
ATAAATAATATTAGATTTTGATTACTACCCCCATCTCTCTTTATATTACTTTTAAGAACA
TTATTTACACCAAATACAGGAACAGGTTGAACTGTATATCCACCTTTATCATCATATATA
TTTCATTCATCACCATCTATTGATATTGCAATTTTTTCATTACATATAACAGGAATTTCT
TCAATTATTGGATCTTTAAATTTTATTGTAACTATTATATTAATAAAAAATTTTTCATTA
AATTATGATCAAATTAACTTATTTTCCTGATCTGTATGTATTACA
>KY317972;tax=d:Eukarya,p:Arthropoda,c:Insecta,o:Hymenoptera,f:Apidae,g:Bombus,s:Bombus bimaculatus
GATTTGGAAATTATTTAATTCCTTTAATATTAGGATCACCTGATATAGCTTTTCCTCGAA
TAAATAATATTAGATTTTGATTACTACCTCCATCTCTTTTTATATTACTTTTAAGAATAT
TATTTACACCAAATGCAGGAACAGGTTGAACTGTATATCCTCCTTTATCATCATATATAT
TTCATTCATCACCATCTATTGATATTGCAATTTTTTCCTTACATATAACAGGAATTTCTT
CAATTATTGGATCTTTAAATTTTATTGTAACTATTATATTAATAAAAAATTTTTCATTAA
ATTATGACCAAATTAATTTATTCTCTTGATCTGTATGTATTACAGTAATATTATTAATTT
TATCCTTACCAGTTTTAGCAGGAGCAATTACTATACTTCTTTTTGATCGAAATTTCAATA
CATCATTTTTTGATCCAATAGGAGGAGGTGATCCAATTCTTTATCAACATTTATTT
>MF936074;tax=d:Eukarya,p:Arthropoda,c:Insecta,o:Hymenoptera,f:Apidae,g:Bombus,s:Bombus bimaculatus
ATAATATACTTTATTTTTGCTATATGATCAGGAATAATTGGATCATCAATAAGATTATTA
ATTCGAATAGAACTTAGTCATCCTGGAATATGAATTAATAATGATCAAATTTACAATTCA
TTAGTTACAAGTCATGCATTTTTAATAATTTTTTTTATAGTAATACCATTTATAATTGGA
GGATTTGGAAATTATTTAATTCCTTTAATATTAGGATCACCTGATATAGCTTTTCCTCGA
ATAAATAATATTAGATTTTGATTACTACCCCCATCTCTCTTTATATTACTTTTAAGAACA
TTATTTACACCAAATACAGGAACAGGTTGAACTGTATATCCACCTTTATCATCATATATA
TTTCATTCATCACCATCTATTGATATTGCAATTTTTTCATTACATATAACAGGAATTTCT
TCAATTATTGGATCTTTAAATTTTATTGTAACTATTATATTAATAAAAAATTTTTCATTA
AATTATGATCAAATTAACTTATTTTCCTGATCTGTATGTATTACAGTAATATTATTAATT
TTATCTTTACCA
>MG441768;tax=d:Eukarya,p:Arthropoda,c:Insecta,o:Hymenoptera,f:Apidae,g:Bombus,s:Bombus bimaculatus
TATATTTTATTTTTGCTATATGATCAGGAATAATTGGATCATCAATAAGACTATTAATTC

GAATAGAACTTAGTCATCCCGGAATATGAATTAATAATGATCAAATTTATAATTCATTAG
TTACAAGTCATGCATTTTTAATAATTTTTTTTATAGTAATACCATTTATAATTGGAGGAT
TTGGAAATTATTTAATTCCTTTAATATTAGGATCACCTGACATAGCTTTTCCTCGAATAA
ATAATATTAGATTTTGATTACTACCTCCATCTCTTTTTATATTACTTTTAAGAATATTAT
TTACACCAAATGCAGGAACAGGTTGAACTGTATATCCTCCTTTATCATCATATATATTTC
ATTCATCACCATCTATTGATATTGCAATTTTTTCCTTACATATAACAGGAATTTCCTCAA
TTATTGGATCTTTAAATTTTATTGTAACTATTATACTAATAAAAAATTTTTCATTAAATT
ATGACCAAATTAATTTATTCTCTTGATCTGTATGTATTACAGTAATATTATTAATTTTAT
CCTTACCAGTTTTAGCAGGAGCAATTACTATACTTCTTTTTGATCGAAATTTCAATACAT
CATTTTTTGATCAAATAGGAGGAGGTGATCCAATTCTTTATCAACATTTA
>MG441810;tax=d:Eukarya,p:Arthropoda,c:Insecta,o:Hymenoptera,f:Apidae,g:Bombus,s:Bombus bimaculatus
ATATTTTATTTTTGCTATATGATCAGGAATAATTGGATCATCAATAAGACTATTAATTCG
AATAGAACTTAGTCATCCTGGAATATGAATTAATAATGATCAAATTTATAATTCATTAGT
TACAAGTCATGCATTYTTAATAATTTTTTTTATAGTAATACCATTTATAATTGGAGGATT
TGGAAATTATTTAATTCCTTTAATATTAGGATCACCTGACATAGCTTTTCCTCGAATAAA
TAATATTAGATTTTGATTACTRCCTCCATCTCTTTTTATATTACTTTTAAGAATATTATT
TACACCAAATGCAGGAACAGGTTGAACTGTATATCCTCCTTTATCATCATATATATTTCA
TTCATCACCATCTATTGATATTGCAATTTTTTCCTTACATATAACAGGAATTTCCTCAAT
TATTGGATCTTTAAATTTTATTGTAACTATTATACTAATAAAAAATTTTTCATTAAATTA
TGACCAAATTAATTTATTCTCTTGATCTGTATGTATTACAGTAATATTATTAATTTTATC
CTTACCAGTTTTAGCAGGAGCAATTACTATACTTCTTTTTGATCGAAATTTCAATACATC
ATTTTTTGATCCAATAGGAGGAGGTGATCCAATTCTTTATCAACATTTA
>MG445614;tax=d:Eukarya,p:Arthropoda,c:Insecta,o:Hymenoptera,f:Apidae,g:Bombus,s:Bombus bimaculatus
TATATTTTATTTTTGCTATATGATCAGGAATAATTGGATCATCAATAAGAATATTAATTC
GAATAGAACTTAGTCATCCCGGAATATGAATTAATAATGATCAAATTTATAATTCATTAG
TTACAAGTCATGCATTTTTAATAATTTTTTTTATAGTAATACCATTTATAATTGGAGGAT
TTGGAAATTATTTAATTCCTTTAATATTAGGATCACCTGACATAGCTTTTCCTCGAATAA
ATAATATTAGATTTTGATTACTACCTCCATCTCTTTTTATATTACTTTTAAGAATATTAT
TTACACCAAATGCAGGAACAGGTTGAACTGTATATCCTCCTTTATCATCATATATATTTC
ATTCATCACCATCTATTGATATTGCAATTTTTTCATTACATATAACAGGAATTTCCTCAA
TTATTGGATCTTTAAATTTTATTGTAACTATTATATTAATAAAAAATTTTTCATTAAATT
ATGACCAAATTAATTTATTCTCTTGATCTGTATGTATTACAGTAATATTATTAATTTTAT
CCTTACCAGTTTTAGCAGGAGCAATTACTATACTTCTTTTTGATCGAAATTTCAATACAT
CATTTTTTGATCCAATAGGAGGAGGTGATCCAATTCTTTATCAACATTTA
>MG446783;tax=d:Eukarya,p:Arthropoda,c:Insecta,o:Hymenoptera,f:Apidae,g:Bombus,s:Bombus bimaculatus
TATATTTTATTTTTGCTATATGATCAGGAATAATTGGATCATCAATAAGACTATTAATTC
GAATAGAACTTAGTCATCCTGGAATATGAATTAATAATGATCAAATTTATAATTCATTAG
TTACAAGTCATGCATTTTTAATAATTTTTTTTATAGTAATACCATTTATAATTGGAGGAT
TTGGAAATTATTTAATTCCTTTAATATTAGGATCACCTGACATAGCTTTTCCTCGAATAA
ATAATATTAGATTTTGATTACTACCTCCATCTCTTTTTATATTACTTTTAAGAATATTAT
TTACACCAAATGCAGGAACAGGTTGAACTGTATATCCTCCTTTATCATCATATATATTTC
ATTCATCACCATCTATTGATATTGCAATTTTTTCCTTACATATAACAGGAATTTCCTCAA
TTATTGGATCTTTAAATTTTATTGTAACTATTATACTAATAAAAAATTTTTCATTAAATT
ATGACCAAATTAATTTATTCTCTTGATCTGTATGTATTACAGTAATATTATTAATTTTAT
CCTTACCAGTTTTAGCAGGAGCAATTACTATACTTCTTTTTGATCGAAATTTCAATACAT
CATTTTTTGATCAAATAGGAGGAGGTGATCCAATTCTTTATCAACATTTA
>KJ838277;tax=d:Eukarya,p:Arthropoda,c:Insecta,o:Hymenoptera,f:Megachilidae,g:Anthidium,s:Anthidium manicatum
AATACTTTATATAATTTTTTCTTTATGAGCAGGAATAATTGGTTCATCAATAAGAATAAT
TATTCGAATAGAATTAAGAATTCCAGGATCTTGAATTGAAAATGATCAAATTTATAATTC
TTTAGTAACAGCTCATGCTTTTTTAATAATTTTTTTTTTAGTTATACCTTTTTTAATTGG

AGGATTTGGAAATTGATTAATTCCTTTAATATTAGGTATTCCTGATATAGCATTCCCTCG
AATAAATAATATTAGATTTTGATTATTACCCCCATCATTAATTCTATTATTATTAAGAAA
TTTATTTAATCCAAGTCCAGGAACTGGATGAACAATTTATCCCCCTTTATCTTTATATTT
ATATCATTCATCTCCTTCTGTAGATTTAATAATTTTATCTTTACATATTTCTGGTATTTC
ATCAATTTTAGGTTCATTAAATTTTATTGTAACAATTTTAATAATGAAAAATTTTTCATT
AATTATAAATAAATTACCCATATATTCATGAGCTGTTTTAATTACAACAATTTTATTATT
ATTATCTTTACCAGTTTTAGCTGGAGCTATTACAATATTATTATTTGATCGAAATTTAAA
TACTTCATTTTTTGATCCAATAGGAGGAGGTGATCCTATTTTATATCAACATTTATTT
>FJ582230;tax=d:Eukarya,p:Arthropoda,c:Insecta,o:Hymenoptera,f:Megachilidae,g:Heriades,s:Heriades carinatus
GCAATATGAGCGGGAATGATTGGTTCTGCAATAAGAATTATTATTCGAATAGAATTAAGA
TCTTCAGGATCATGAATAAAAAATGATCAAATTTATAATTCTATTGTAACTGCTCATGCA
TTTTTAATAATTTTTTTTTTAGTTATACCATTTATAATTGGGGGATTTGGAAATTGATTA
ATTCCTTTATTATTAGGAATTCCTGATATAGCTTTTCCTCGAATAAATAATATTAGATTT
TGATTATTACCTCCATCATTAATTATATTACTTGTAAGGAATTTGTATAATCCGAGACCT
GGAACAGGATGAACAGTTTATCCACCTTTATCTTCTTATATATTTCATTCTTCACCATCT
GTAGATTTTGCAATTTTTTCATTACATATTTCAGGAATTTCATCAATTATAGGATCATTA
AATTTTATTGTAACAATTTTAATAATAAAAAATATTTCAATTAATTATATACAAATACCT
TTATTTTCATGATCTGTTTTTATTACAACAATTTTACTTTTATTATCATTACCTGTTTTA
GCAGGAGCAATTACAATACTTTTATTTGATCGAAATTTAAATTCATCATTTTTGGATCTT
ATGGGTGGAGGAGATCCAATTTTATATCAACATTTATTT
>KJ163559;tax=d:Eukarya,p:Arthropoda,c:Insecta,o:Hymenoptera,f:Megachilidae,g:Heriades,s:Heriades carinatus
ATTTTTGCAATATGAGCGGGAATGATTGGTTCTGCAATAAGAATTATTATTCGAATAGAA
TTAAGATCTTCAGGATCATGAATAAAAAATGATCAAATTTATAATTCTATTGTAACTGCT
CATGCATTTTTAATAATTTTTTTTTTAGTTATACCATTTATAATTGGGGGATTTGGAAAT
TGATTAATTCCTTTATTATTAGGAATTCCTGATATAGCTTTTCCTCGAATAAATAATATT
AGATTTTGATTATTACCTCCATCATTAATTATATTACTTGTAAGGAATTTGTATAATCCG
AGACCTGGAACAGGATGAACAGTTTATCCACCTTTATCTTCTTATATATTTCATTCTTCA
CCATCTGTAGATTTTGCAATTTTTTCATTACATATTTCAGGAATTTCATCAATTATAGGA
TCATTAAATTTTATTGTAACAATTTTAATAATAAAAAATATTTCAATTAATTATATACAA
ATACCTTTATTTTCATGATCTGTTTTTATTACAACAATTTTACTTTTATTATCATTACCT
GTTTTAGCAGGAGCAATCACAATACTTTTATTTGATCGAAATTTAAATTCATCATTTTTT
GATCCTATGGGTGGAGGAGATCCAATTTTATATCAACATTTATTT
>MG505215;tax=d:Eukarya,p:Arthropoda,c:Insecta,o:Hymenoptera,f:Megachilidae,g:Hoplitis,s:Hoplitis producta
ATAATTTTCAGAATATGATCAGGAATAATTGGTTCTGCAATAAGAATTATTATTCGTATA
GAATTAAGAATTCCTGGATCTTGAATTAATAATGATCAAATTTATAATTCAATTGTAACT
GCTCATGCTTTTTTAATAATTTTTTTTTTAGTTATACCTTTTATAATTGGAGGATTTGGA
AATTGATTAGTTCCATTAATATTAGGTATTCCTGATATAGCTTTTCCTCGAATAAATAAT
ATTAGATTTTGATTACTTCCTCCTTCATTATTACTATTACTATTAAGAAATTTTTTTAAT
CCAAGACCAGGAACTGGATGAACAGTTTATCCTCCTTTATCATCATATTTATATCATTCT
TCACCATCAGTTGATATAGCTATTTTTTCATTACATATTTCAGGTTTATCTTCAATTATA
GGATCATTAAATTTTATTGTAACTATTATTTTAATAAAAAATATTTCATTACAATATAAT
CAACTTTCATTATTTCCATGGTCTGTATTTATTACAACTATTTTATTACTTTTATCATTA
CCTGTATTAGCAGGAGCAATTACTATACTTTTATTTGAT
>JN293779;tax=d:Eukarya,p:Arthropoda,c:Insecta,o:Hymenoptera,f:Megachilidae,g:Hoplitis,s:Hoplitis spoliata
TTATATAATTTTTAGTATATGATCAGGAATAATTGGTTCAGCACTTAGAATTATTATTCG
AATAGAATTAAGAACCCCTGGTTCTTGAATTTCTAATGATCAAATTTATAATTCAATTGT
TACTGCTCATGCTTTTTTAATAATTTTTTTTTTAGTTATACCATTTATAATTGGAGGTTT
TGGAAATTGATTAGTTCCATTAATATTAGGAATTCCAGATATAGCTTTTCCTCGAATAAA
TAATATTAGATTTTGATTATTACCACCCTCATTATTTATATTATTATTAAGAAATTTTCT
TAGTCCTAGACCAGGAACAGGATGAACTGTTTATCCTCCTTTATCTTCTTATATATATCA
TTCTTCACCATCAGTTGATATAGCAATTTTTTCTTTACATATTTCAGGTTTATCTTCAAT

TATAGGATCTTTAAATTTTATTGTTACTATTATTATAATAAAAAATGTTTCATTAAATCA
TATACAACTTCCTTTATTTTCTTGATCTGTTTTTATTACAACTATTTTATTATTATTATC
ATTACCAGTATTAGCAGGAGCTATTACAATACTATTATTTGATCGAAATTTAAATACTTC
ATTTTTTGATCCAATAGGTGGAGGAGATCCAATTTTATATCAACATTTATTT
>FJ582312;tax=d:Eukarya,p:Arthropoda,c:Insecta,o:Hymenoptera,f:Megachilidae,g:Megachile,s:Megachile gemula
GCATTATGAGCAGGAATAATTGGTTCAAGATTATCAATAATTATTCGAATAGAATTAAGA
ATCCCAGGTTCTTGAATTAAAAATGATCAAATTTATAATTCAATTGTTACTGCTCATGCA
TTTTTAATAATTTTTTTTTTAGTTATACCATTTATAATTGGAGGATTTGGTAATTGATTA
ATACCATTAATAATTGGAGCTCCTGATATAGCATTCCCACGAATAAATAATGTAAGATTT
TGATTATTACCTCCATCATTAATCTTATTATTATCAAGAAACTTATTAAATCCTAGACCA
GGAACTGGATGAACTGTATATCCTCCCTTATCATTATATATATTTCATCCATCACCTTCT
GTAGATTTAACAATTTTTTCTTTACATTTATCAGGAATTTCATCAATTATTGGTTCATTA
AATTTTATAGTAACTATTTTATTAATAAAAAATTTTTCATTAAATTATAGTCAAATAACA
TTATTCCCATGATCAATTTTTATTACAACAGTTTTACTTTTATTATCTCTTCCAGTTTTA
GCAGGAGCTATTACAATATTATTATTTGATCGAAATTTAAATACATCATTTTTTGATCCA
ATAGGAGGGGGTGATCCTATTTTATATCAACATTTATTT
>KM562651;tax=d:Eukarya,p:Arthropoda,c:Insecta,o:Hymenoptera,f:Megachilidae,g:Megachile,s:Megachile gemula
AATATTATATATAATTTTTGCATTATGAGCAGGAATAATTGGTTCAAGATTATCAATAAT
TATTCGAATAGAATTAAGAATCCCAGGTTCTTGAATTAAAAATGATCAAATTTATAATTC
AATTGTTACTGCTCATGCATTTTTAATAATTTTTTTTTTAGTTATACCATTTATAATTGG
AGGATTTGGTAATTGATTAATACCATTAATAATTGGAGCTCCTGATATAGCATTCCCACG
AATAAATAATGTAAGATTTTGATTATTACCTCCATCATTAATCTTATTATTATCAAGAAA
CTTATTAAATCCTAGACCAGGAACTGGATGAACTGTATATCCTCCCTTATCATTATATAT
ATTTCATCCATCACCTTCTGTAGATTTAACAATTTTTTCTTTACATTTATCAGGAATTTC
ATCAATTATTGGTTCATTAAATTTTATAGTAACTATTTTATTAATAAAAAATTTTTCATT
AAATTATAGTCAAATAACATTATTCCCATGATCAATTTTTATTACAACAGTTTTACTTTT
ATTATCTCTTCCAGTTTTAGCAGGAGCTATTACAATATTATTATTTGATCGAAATTTAAA
TACATCATTTTTTGATCCAATAGGAGGGGGT
>KR796246;tax=d:Eukarya,p:Arthropoda,c:Insecta,o:Hymenoptera,f:Megachilidae,g:Megachile,s:Megachile gemula
AATATTATATATAATTTTTGCATTATGAGCAGGAATAATTGGTTCAAGATTATCAATAAT
TATTCGAATAGAATTAAGAATCCCAGGTTCTTGAATTAAAAATGATCAAATTTATAATTC
AATTGTTACTGCTCATGCATTTTTAATAATTTTTTTTTTAGTTATACCATTTATAATTGG
AGGATTTGGTAATTGATTAATACCATTAATAATTGGAGCTCCTGATATAGCATTCCCACG
AATAAATAATGTAAGATTTTGATTATTACCTCCATCATTAATCTTATTATTATCAAGAAA
CTTATTAAATCCTAGACCAGGAACTGGATGAACTGTATATCCTCCCTTATCATTATATAT
ATTTCATCCATCACCTTCTGTAGATTTAACAATTTTTTCTTTACATTTATCAGGAATTTC
ATCAATTATTGGTTCATTAAATTTTATAGTAACTATTTTATTAATAAAAAATTTTTCATT
AAATTATAGTCAAATAACATTATTCCCATGATCAATTTTTATTACAACAGTTTTACTTTT
ATTATCTCTTCCAGTTTTAGCAGGAGCTATTACAATATTATTATTTGATCGAAATTTAAA
TACATCATTTTTTGATCCAATAGGAGGGGGTGATCCTATTTTATATCAACATTTATTT
>MG501828;tax=d:Eukarya,p:Arthropoda,c:Insecta,o:Hymenoptera,f:Megachilidae,g:Megachile,s:Megachile gemula
ATATTATATATAATTTTTGCATTATGAGCAGGAATAATTGGTTCAAGATTATCAATAATT
ATTCGAATAGAATTAAGAATCCCAGGTTCTTGAATTAAAAATGATCAAATTTATAATTCA
ATTGTTACTGCTCATGCATTTTTAATAATTTTTTTTTTAGTTATACCATTTATAATTGGA
GGATTTGGTAATTGATTAATACCATTAATAATTGGAGCTCCTGATATAGCATTCCCACGA
ATAAATAATGTAAGATTTTGATTATTACCTCCATCATTAATCTTATTATTATCAAGAAAC
TTATTAAATCCTAGACCAGGAACTGGATGAACTGTATATCCTCCCTTATCATTATATATA
TTTCATCCATCACCTTCTGTAGATTTAACAATTTTTTCTTTACATTTATCAGGAATTTCA
TCAATTATTGGTTCATTAAATTTTATAGTAACTATTTTATTAATAAAAAATTTTTCATTA
AATTATAGTCAAATAACATTATTCCCATGATCAATTTTTATTACAACAGTTTTACTTTTA
TTATCTCTTCCAGTTTTAGCAGGAGCTATTACAATATTATTATTTGAT

>GU690117;tax=d:Eukarya,p:Arthropoda,c:Insecta,o:Hymenoptera,f:Apidae,g:Melissodes,s:Melissodes druriella
TATTTTATATATAATTTTGGCTATATGATCTGGTATAATTGGTACTTCAATAAGTTTAAT
TATTCGGATAGAATTAAGATGCCCTGGTAAATGAATTGGTAATGATCAGTTGTATAATTC
ATTTGTAACGGCTCATGCTTTTTTAATAATTTTTTTTATAGTAATACCTTTTTTAATTGG
GGGTTTTGGAAATTGATTAATGCCAATGATATTAGGATCTCCTGATATGGCTTTTCCTCG
AATAAATAATATTAGATTTTGATTATTACCTTGTTCTTTAATATTCTTATTATTAAGAAA
TTTATTTACTATAACTCCTGGGACTGGATGAACTGTTTACCCTCCTTTGTCATCGTATAT
ATTCCATCCTTCACCTTCTGTTGATATTATAATTTTTTCTTTACATTTATCGGGTATATC
ATCAATTTTAGGAGCAATAAATTTTATGGTAACAATTATAATAATAAAAAATTTGTCAAT
AAATTTTGATCAAATTAATTTATTTTCTTGATCTCTTTTTATTACTGCAGTTTTATTATT
ATTATCTTTACCAGTATTGGCAGGAGCTATTACTATATTATTATTTGATCGAAATTTAAA
TACCTCTTTTTTTGATCCAATGGGAGGAGGTGATCCAATTTTATATCAACATTTATTT
>GU690113;tax=d:Eukarya,p:Arthropoda,c:Insecta,o:Hymenoptera,f:Apidae,g:Melissodes,s:Melissodes druriella
TATTTTATATATAATTTTGGCTATATGATCTGGTATAATTGGTACTTCAATAAGTTTAAT
TATTCGGATAGAATTAAGATGCCCTGGTAAATGAATTGGTAATGATCAGTTGTATAATTC
ATTTGTAACGGCTCATGCTTTTTTAATAATTTTTTTTATAGTAATACCTTTTTTAATTGG
GGGTTTTGGAAATTGATTAATGCCAATGATATTAGGATCTCCTGATATGGCTTTTCCTCG
AATAAATAATATTAGATTTTGATTATTACCTTGTTCTTTAATATTTTTATTATTAAGAAA
TTTATTTACTATAACTCCTGGGACTGGATGAACTGTTTACCCTCCTTTGTCATCGTATAT
ATTCCATCCTTCACCTTCTGTTGATATTATAATTTTTTCTTTACATTTATCGGGTATATC
ATCAATTTTAGGAGCAATAAATTTTATGGTAACAATTATAATAATAAAAAATTTGTCAAT
AAATTTTGATCAAATTAATTTATTTTCTTGATCTCTTTTTATTACTGCAGTTTTATTATT
ATTATCTTTACCAGTATTGGCAGGAGCTATTACTATATTATTATTTGATCGAAATTTAAA
TACCTCTTTTTTTGATCCAATGGGAGGAGGTGATCCAATTTTATATCAACATTTATTT
>GU690114;tax=d:Eukarya,p:Arthropoda,c:Insecta,o:Hymenoptera,f:Apidae,g:Melissodes,s:Melissodes druriella
TATTTTATATATAATTTTGGCTATATGATCTGGTATAATTGGTACTTCAATAAGTTTAAT
TATTCGGATAGAATTAAGATGCCCTGGTAAATGAATTGGTAATGATCAGTTGTATAATTC
ATTTGTAACGGCTCATGCTTTTTTAATAATTTTTTTTATAGTAATACCTTTTTTAATTGG
GGGTTTTGGAAATTGATTAATGCCAATGATATTAGGATCTCCTGATATGGCTTTTCCTCG
AATAAATAATATTAGATTTTGATTATTACCTTGTTCTTTAATATTTTTATTATTAAGAAA
TTTATTTACTATAACTCCTGGGACTGGATGAACTGTTTACCCTCCTTTGTCATCGTATTT
ATTCCATCCTTCACCTTCTGTTGATATTATAATTTTTTCTTTACATTTATCGGGTATATC
ATCAATTTTAGGAGCAATAAATTTTATGGTAACAATTATAATAATAAAAAATTTGTCAAT
AAATTTTGATCAAATTAATTTATTTTCTTGATCTCTTTTTATTACTGCAGTTTTATTATT
ATTATCTTTACCAGTATTGGCAGGAGCTATTACTATATTATTATTTGATCGAAATTTAAA
TACCTCTTTTTTTGATCCAATGGGAGGAGGTGATCCAATTTTATATCAACATTTATTT
>GU690119;tax=d:Eukarya,p:Arthropoda,c:Insecta,o:Hymenoptera,f:Apidae,g:Melissodes,s:Melissodes druriella
TATTTTATATATAATTTTGGCTATATGATCTGGTATAATTGGTACTTCAATAAGTTTAAT
TATTCGGATAGAATTAAGATGCCCTGGTAAATGAATTGGTAATGATCAGTTGTATAATTC
ATTTGTAACGGCTCATGCTTTTTTAATAATTTTTTTTATAGTAATACCTTTTTTAATTGG
GGGTTTTGGAAATTGATTAATGCCAATGATATTAGGATCTCCTGATATGGCTTTTCCTCG
AATAAATAATATTAGATTTTGATTATTACCTTGTTCTTTAATATTCTTATTATTAAGAAA
TTTATTTACTATAACTCCTGGGACTGGATGAACTGTTTACCCTCCTTTGTCATCGTATAT
ATTCCATCCTTCACCTTCTGTTGATATTATAATTTTTTCTTTACATTTATCGGGTATATC
ATCAATTTTAGGAGCAATAAATTTTATGGTAACAATTATAATAATAAAAAATTTGTCAAT
AAATTTTGATCAAATTAATTTATTTTCTTGATCTCTTTTTATTACTGCAGTTTTATTATT
ATTATCTTTACCAGTATTGGCAGGAGCTATTACTATATTATTATTTGATCGAAATTTAAA
T
>KR893980;tax=d:Eukarya,p:Arthropoda,c:Insecta,o:Hymenoptera,f:Apidae,g:Melissodes,s:Melissodes druriella
ATTTTATATATAATTTTGGCTATATGATCTGGTATAATTGGTACTTCAATAAGTTTAATT
ATTCGGATAGAATTAAGATGCCCTGGTAAATGAATTGGTAATGATCAGTTGTATAATTCA

TTTGTAACGGCTCATGCTTTTTTAATAATTTTTTTTATAGTAATACCTTTTTTAATTGGG
GGTTTTGGAAATTGATTAATGCCAATGATATTAGGATCTCCTGATATGGCTTTTCCTCGA
ATAAATAATATTAGATTTTGATTATTACCTTGTTCTTTAATATTCTTATTATTAAGAAAT
TTATTTACTATAACTCCTGGGACTGGATGAACTGTTTACCCTCCTTTGTCATCGTATATA
TTCCATCCTTCACCTTCTGTTGATATTATAATTTTTTCTTTACATTTATCGGGTATATCA
TCAATTTTAGGAGCAATAAATTTTATGGTAACAATTATAATAATAAAAAATTTGTCAATA
AATTTTGATCAAATTAATTTATTTTCTTGATCTCTTTTTATTACTGCAGTTTTA
>KR894169;tax=d:Eukarya,p:Arthropoda,c:Insecta,o:Hymenoptera,f:Apidae,g:Melissodes,s:Melissodes druriella
ATAATTTTGGCTATATGATCTGGTATAATTGGTACTTCAATAAGTTTAATTATTCGGATA
GAATTAAGATGCCCTGGTAAATGAATTGGTAATGATCAGTTGTATAATTCATTTGTAACG
GCTCATGCTTTTTTAATAATTTTTTTTATAGTAATACCTTTTTTAATTGGGGGTTTTGGA
AATTGATTAATGCCAATGATATTAGGATCTCCTGATATGGCTTTTCCTCGAATAAATAAT
ATTAGATTTTGATTATTACCTTGTTCTTTAATATTCTTATTATTAAGAAATTTATTTACT
ATAACTCCTGGGACTGGATGAACTGTTTACCCTCCTTTGTCATCGTATATATTCCATCCT
TCACCTTCTGTTGATATTATAATTTTTTCTTTACATTTATCGGGTATATCATCAATTTTA
GGAGCAATAAATTTTATGGTAACAATTATAATAATAAAAAATTTGTCAATAAATTTTGAT
CAAATTAATTTATTTTCTTGATCTCTTTTTATTACTGCAGTTTTATTATTATTATCTTTA
CCAGTATTGGCAGGAGCTATTACTATATTATTATTTGAT
>KR894261;tax=d:Eukarya,p:Arthropoda,c:Insecta,o:Hymenoptera,f:Apidae,g:Melissodes,s:Melissodes druriella
ATTTTATATATATAATTTTGGCTATATGATCTGGTATAATTGGTACTTCAATAAGTTTAATT
ATTCGGATAGAATTAAGATGCCCTGGTAAATGAATTGGTAATGATCAGTTGTATAATTCA
TTTGTAACGGCTCATGCTTTTTTAATAATTTTTTTTATAGTAATACCTTTTTTAATTGGG
GGTTTTGGAAATTGATTAATGCCAATGATATTAGGATCTCCTGATATGGCTTTTCCTCGA
ATAAATAATATTAGATTTTGATTATTACCTTGTTCTTTAATATTCTTATTATTAAGAAAT
TTATTTACTATAACTCCTGGGACTGGATGAACTGTTTACCCTCCTTTGTCATCGTATATA
TTCCATCCTTCACCTTCTGTTGATATTATAATTTTTTCTTTACATTTATCGGGTATATCA
TCAATTTTAGGAGCAATAAATTTTATGGTAACAATTATAATAATAAAAAATTTGTCAATA
AATTTTGATCAAATTAATTTATTTTCTTGATCTCTTTTTATTACTGCAGTTTTATTATTA
TTATCTTTACCAGTATTGGCAGGA
>MG251043;tax=d:Eukarya,p:Arthropoda,c:Insecta,o:Hymenoptera,f:Apidae,g:Melissodes,s:Melissodes druriella
CTGGTAAATGAATTGGTAATGATCAGTTGTATAATTCATTTGTAACGGCTCATGCTTTTT
TAATAATTTTTTTTATAGTAATACCTTTTTTAATTGGGGGTTTTGGAAATTGATTAATGC
CAATGATATTAGGATCTCCTGATATGGCTTTTCCTCGAATAAATAATATTAGATTTTGAT
TATTACCTTGTTCTTTAATATTCTTATTATTAAGAAATTTATTTACTATAACTCCTGGGA
CTGGATGAACTGTTTACCCTCCTTTGTCATCGTATATATTCCATCCTTCACCTTCTGTTG
ATATTATAATTTTTTCTTTACATTTATCGGGTATATCATCAATTTTAGGAGCAATAAATT
TTATGGTAACAATTATAATAATAAAAAATTTGTCAATAAATTTTGATCAAATTAATTTAT
TTTCTTGATCTCTTTTTATTACTGCAGTTTTATTATTATTATCTTTACCAGTATTGGCAG
GAGCTATTACTATATTATTATTTGATCGAAATTTAAATACCTCTTTTTTTGATCCAATGG
GAGGAGGTGATCCAATTTTATATCAACATTTATTT
>MG442722;tax=d:Eukarya,p:Arthropoda,c:Insecta,o:Hymenoptera,f:Apidae,g:Melissodes,s:Melissodes druriella
ATTTTATATATATAATTTTGGCTATATGATCTGGTATAATTGGTACTTCAATAAGTTTAATT
ATTCGGATAGAATTAAGATGCCCTGGTAAATGAATTGGTAATGATCAGTTGTATAATTCA
TTTGTAACGGCTCATGCTTTTTTAATAATTTTTTTTATAGTAATACCTTTTTTAATTGGG
GGTTTTGGAAATTGATTAATGCCAATGATATTAGGATCTCCTGATATGGCTTTTCCTCGA
ATAAATAATATTAGATTTTGATTATTACCTTGTTCTTTAATATTCTTATTATTAAGAAAT
TTATTTACTATAACTCCTGGGACTGGATGAACTGTTTACCCTCCTTTGTCATCGTATATA
TTCCATCCTTCACCTTCTGTTGATATTATAATTTTTTCTTTACATTTATCGGGTATATCA
TCAATTTTAGGAGCAATAAATTTTATGGTAACAATTATAATAATAAAAAATTTGTCAATA
AATTTTGATCAAATTAATTTATTTTCTTGATCTCTTTTTATTACTGCAGTTTTATTATTA
TTATCTTTACCAGTATTGGCAGGAGCTATTACTATA

>KT619962;tax=d:Eukarya,p:Arthropoda,c:Insecta,o:Hymenoptera,f:Apidae,g:Nomada,s:Nomada pygmaea
ATATTATATATTATTTTTGCTTTATGATCAGGAATAATTGGAACTTCTATAAGATTTATT
ATTCGTATAGAATTAAGTAATCCTGGTGAATGAATTATAAATGATCAAATTTATAATTCT
TTAGTTACAGCTCATGCTTTTTTAATAATTTTTTTTATAGTAATACCTTTTATAATTGGA
GGATTTGGTAATTGATTAGTTCCTTTGATATTAGGATGTCCTGATATAGCTTTTCCTCGT
ATAAATAATATTAGATTTTGATTATTACCTCCATCTTTAATTTTATTATTATTAAGAAAT
TTATTTAATATTACTCCAGGAACAGGATGAACAGTTTATCCTCCATTATCTTCATCAATA
TTTCATAGATCTCCTTCAGTTGATTTAGCAATTTTTTCTCTTCATATATCTGGTATTTCT
TCAATTATAGGAGCTATAAATTTTATAGTTACAATTATATTAATAAAGAATATTTCAATA
AATTATGATCAAATTAATTTATTTTCATGATCAATTTTTATTACAGCAATTTTATTATTA
TTATCATTACCTGTTTTAGCT
>FJ582062;tax=d:Eukarya,p:Arthropoda,c:Insecta,o:Hymenoptera,f:Andrenidae,g:Andrena,s:Andrena tridens
TGGTGCGTCACTTAGATTTATCATTCGTATAGAATTAAGAAATCCAGGCAAATGAATCAA
TAATGATCAAATTTATAACTCCATTGTAACTTCTCACGCTTTTATTATAATTTTTTTTAT
AGTTATACCATTTATAATTGGAGGATTTGGAAATTGACTTACACCATTAATACTTGGAGC
ACCAGATATAGCTTTCCCACGTATAAATAATATAAGATTTTGACTTCTACCTCCATCAAT
TTTAATTATTCTAATAAGAATAGTATTAAATTCAGGATCTGGAACAGGATGAACAGTTTA
TCCACCATTATCATCATACAATTTTCATCCATCATCATCAGTAGATTTAACAATTTTTTC
CCTACACATTGCAGGTGTTTCATCCATTATAGGAGCAATTAACTTCATTGTAACAATTCT
AAATATAAAAAATATATCATTAAATTATGATCAACTTCCATTATTCCCATGATCAGTATT
TATTACAACAATTCTTTTATTAATTTCATTGCCAGTACTAGCAGGAGCCATTACAATATT
ATTATCAGATCGAAACTTAAATTCATCATTTTTTGATCCAATAGGAGGTGGAGATCCAAT
TTTATATCAACATTTATTT
>HM374738;tax=d:Eukarya,p:Arthropoda,c:Insecta,o:Hymenoptera,f:Colletidae,g:Hylaeus,s:Hylaeus annulatus
AATATTATATTTCATTTTTGCTATATGAGCAGGAATAATTGGAACATCATTAAGAATAAT
TATTCGAATAGAATTAAGAATACCTGGAATATGAATTAATAATGATCAAATATATAATAC
TATTGTTACATCACATGCATTTATTATAATTTTCTTTATAGTTATACCATTTATAATTGG
AGGATTCGGAAATTGATTAATTCCTATTATAATTGGAGCTCCAGATATAGCTTTTCCACG
AATAAATAATATAAGATTTTGATTATTACCACCATCTTTAATATTATTATTAATAAGAAG
AAGTTTTTACTCAGGATCAGGTACTGGGTGAACAATTTATCCTCCATTATCATCTTCTAT
ATACCATCCTTCATTATCTGTAGACTTTACAATCTTTTCACTTCATATTGCTGGTATTTC
ATCTATTATAGGAGCTATTAATTTTATTGTAACAATTATAAATATAAAAAATATTAATTT
AAACTATGATCAAATAACTTTATTTTCATGATCAGTAATTATTACTGCAATTCTTTTACT
TTTATCTCTACCAGTTCTTGCTGGAGCAATTACTATATTATTAACAGATCGTAATTTAAA
TACATCATTTTTTGACCCTTCAGGTGGGGGAGATCCAATTTTATATCAACATTTATTT
>HQ569277;tax=d:Eukarya,p:Arthropoda,c:Insecta,o:Hymenoptera,f:Colletidae,g:Hylaeus,s:Hylaeus annulatus
AATATTATATTTCATTTTTGCTATATGAGCAGGAATAATTGGAACATCATTAAGAATAAT
TATTCGAATAGAATTAAGAATACCTGGAATATGAATTAATAATGATCAAATATATAATAC
TATTGTTACATCACATGCATTTATTATAATTTTCTTTATAGTTATACCATTTATAATTGG
AGGATTCGGAAATTGATTAATTCCTATTATAATTGGAGCTCCAGATATAGCTTTTCCACG
AATAAATAATATAAGATTTTGATTATTACCACCATCTTTAATATTATTATTAATAAGAAG
AAGTTTATACTCAGGATCAGGTACTGGGTGAACAATTTATCCTCCATTATCATCTTCTAT
ATACCATCCTTCATTATCTGTAGACTTTACAATCTTTTCACTTCATATTGCTGGTATTTC
ATCTATTATAGGAGCTATTAATTTTATTGTAACAATTATAAATATAAAAAATATTAATTT
AAACTATGATCAAATAACTTTATTTTCATGATCAGTAATTATTACTGCAATTCTTTTACT
TTTATCTCTACCAGTTCTTGCTGGAGCAATTACTATATTATTAACAGATCGTAATTTAAA
TACATCATTTTTTGACCCTTCAGGTGGGGGAGATCCAATTTTATATCAACATTTATTT
>JN292774;tax=d:Eukarya,p:Arthropoda,c:Insecta,o:Hymenoptera,f:Colletidae,g:Hylaeus,s:Hylaeus annulatus
AATATTATATTTCATTTTTGCTATATGAGCAGGAATAATTGGAACATCATTAAGAATAAT
TATTCGAATAGAATTAAGAATACCTGGAATATGAATTAATAATGATCAAATATATAATAC
TATTGTTACATCACATGCATTTATTATAATTTTCTTTATAGTTATACCATTTATAATTGG

AGGATTCGGAAATTGATTAATTCCTATTATAATTGGAGCTCCAGATATAGCTTTTCCACG
AATAAATAATATAAGATTTTGATTATTACCACCATCTTTAATATTATTATTAATAAGAAG
AAGTTTATACTCAGGATCAGGTACTGGGTGAACAATTTATCCTCCATTATCATCTTCTAT
ATACCATCCTTCATTATCTGTAGACTTTACAATCTTTTCACTTCATATTGCTGGTATTTC
ATCTATTATAGGAGCTATTAATTTTATTGTAACAATTATAAATATAAAAAATATTAATTT
AAACTATGATCAAATAACTTTATTTTCATGATCAGTAATTATTACTGCAATTCTTTTACT
TTTATCTCTACCAGTTCTTGCTGGAGCAATTACTATATTATTAACAGATCGTAATTTAAA
TACATCATTTTTTGACCCTTCAGGTGGAGGAGATCCAATTTTATATCAACATTTATTT
>KJ837628;tax=d:Eukarya,p:Arthropoda,c:Insecta,o:Hymenoptera,f:Colletidae,g:Hylaeus,s:Hylaeus annulatus
AATATTATATTTTATTTTTGCTATATGAGCAGGTATAATTGGAACATCACTAAGTATAAT
TATTCGAATAGAATTAAGAATTCCTGGAATATGAATTAATAATGATCAAATATATAATAC
TATTGTTACATCACATGCATTTATTATAATTTTTTTTATAGTTATACCATTTATAATTGG
AGGATTCGGAAATTGACTAATTCCTATTATAATTGGAGCTCCAGATATAGCTTTCCCACG
AATAAATAATATAAGATTTTGATTATTACCACCATCTTTAGCATTATTATTAATAAGAAG
AAGTATATATTCAGGATCAGGTACTGGATGAACAATTTATCCTCCATTGTCATCTTCTGT
ATATCATCCTTCATTATCTGTAGATTTTACAATCTTTTCGCTTCACATTGCTGGTATTTC
ATCTATTATAGGAGCTATTAATTTTATTGTAACAATTATAAATATAAAAAATATTAATTT
AAATTATGATCAAATAACTTTATTTTCATGATCAGTAATTATTACTGCAATTCTTTTACT
TTTATCTCTACCAGTTCTTGCTGGAGCAATTACTATATTATTAACTGATCGTAATTTAAA
CACATCATTTTTTGATCCTTCAGGTGGAGGAGACCCAATCTTATATCAACATTTATTT
>KJ837633;tax=d:Eukarya,p:Arthropoda,c:Insecta,o:Hymenoptera,f:Colletidae,g:Hylaeus,s:Hylaeus annulatus
AATATTATATTTTATTTTTGCTATATGAGCAGGTATAATTGGAACATCACTAAGTATAAT
TATTCGAATAGAATTAAGAATTCCTGGAATATGAATTAATAATGATCAAATATATAATAC
TATTGTTACATCACATGCATTTATTATAATTTTTTTTATAGTTATACCATTTATAATTGG
AGGATTCGGAAATTGACTAATTCCTATTATAATTGGAGCTCCAGATATAGCTTTCCCACG
AATAAATAATATAAGATTTTGATTATTACCACCATCTTTAGCATTATTATTAATAAGAAG
AAGTATATATTCAGGATCAGGTACTGGATGAACAATTTATCCTCCATTGTCATCTTCTGT
ATATCATCCTTCATTATCTGTAGATTTTACAATCTTTTCACTTCACATTGCTGGTATTTC
ATCTATTATAGGAGCTATTAATTTTATTGTAACAATTATAAATATAAAAAATATTAATTT
AAATTATGATCAAATAACTTTATTTTCATGATCAGTAATTATTACTGCAATTCTTTTACT
TTTATCTCTACCAGTTCTTGCTGGAGCAATTACTATATTATTAACTGATCGTAATTTAAA
CACATCATTTTTTGATCCTTCAGGTGGAGGAGACCCAATCTTATATCAACATTTATTT
>KR791297;tax=d:Eukarya,p:Arthropoda,c:Insecta,o:Hymenoptera,f:Colletidae,g:Hylaeus,s:Hylaeus annulatus
AATATTATATTTCATTTTTGCTATATGAGCAGGAATAATTGGAACATCATTAAGAATAAT
TATTCGAATAGAATTAAGAATACCTGGAATATGAATTAATAATGATCAAATATATAATAC
TATTGTTACATCACATGCATTTATTATAATTTTCTTTATAGTTATACCATTTATAATTGG
AGGATTCGGAAATTGATTAATTCCTATTATAATTGGAGCTCCAGATATAGCTTTCCCACG
AATAAATAATATAAGATTTTGATTATTACCACCATCTTTAATATTATTATTAATAAGAAG
AAGTTTATATTCAGGATCAGGTACTGGATGAACAATTTATCCTCCATTATCATCTTCTAT
ATATCATCCTTCCTTATCTGTAGACTTTACAATCTTTTCACTTCACATTGCTGGTATTTC
ATCTATTATAGGAGCTATTAATTTTATTGTAACAATTATAAATATAAAAAATATTAATTT
AAACTATGATCAAATAACTTTATTTTCATGATCAGTAATTATTACTGCAATTCTTTTACT
TTTATCTCTAC
>KR802369;tax=d:Eukarya,p:Arthropoda,c:Insecta,o:Hymenoptera,f:Colletidae,g:Hylaeus,s:Hylaeus annulatus
AATATTATATTTCATTTTTGCTATATGAGCAGGAATAATTGGAACATCGTTAAGAATAAT
TATTCGAATAGAATTAAGAATACCTGGAATATGAATTAATAATGATCAAATATATAATAC
TATTGTTACATCACATGCATTTATTATAATTTTCTTTATAGTTATACCATTTATAATTGG
AGGATTCGGAAATTGATTAATTCCTATTATAATTGGAGCTCCAGATATAGCTTTTCCACG
AATAAATAATATAAGATTTTGATTATTACCACCATCTTTAATATTATTATTAATAAGAAG
AAGCTTATACTCAGGATCAGGTACTGGGTGAACAATTTATCCTCCATTATCATCTTCTAT
ATACCATCCTTCATTATCTGTAGACTTTACAATCTTTTCACTTCATATTGCTGGTATTTC

ATCTATTATAGGAGCTATTAATTTTATTGTAACAATTATAAATATAAAAAATATTAATTT
AAACTATGATCAAATAACTTTATTTTCATGATCAGTAATTATTACTGCAATTCTTTTACT
TTTATCTCTACCAGTTCTTGCTGGAGCAATTACTATATTATTAACAGATCGTAATTTAAA
TACATCATTTTTTGACCCTTCAGGTGGGGGAGATCCAATTTTATATCAACATTTATTT
>KR803825;tax=d:Eukarya,p:Arthropoda,c:Insecta,o:Hymenoptera,f:Colletidae,g:Hylaeus,s:Hylaeus annulatus
AATATTATATTTCATTTTTGCTATATGAGCAGGAATAATTGGAACATCGTTAAGAATAAT
TATTCGAATAGAATTAAGAATACCTGGAATATGAATTAATAATGATCAAATATATAATAC
TATTGTTACATCACATGCATTTATTATAATTTTCTTTATAGTTATACCATTTATAATTGG
AGGATTCGGAAATTGATTAATTCCTATTATAATTGGAGCTCCAGATATAGCTTTTCCACG
AATAAATAATATAAGATTTTGATTATTACCACCATCTTTAATATTATTATTAATAAGAAG
AAGTTTATACTCAGGATCAGGTACTGGGTGAACAATTTATCCTCCATTATCATCTTCTAT
ATACCATCCTTCATTATCTGTAGACTTTACAATCTTTTCACTTCATATTGCTGGTATTTC
ATCTATTATAGGAGCTATTAATTTTATTGTAACAATTATAAATATAAAAAATATTAATTT
AAACTATGATCAAATAACTTTATTTTCATGATCAGTAATTATTACTGCAATTCTTTTACT
TTTATCTCTACCAGTTCTTGCTGGAGCAATTACTATATTATTAACAGATCGTAATTTAAA
TACATCATTTTTTGACCCTTCAGGTGGGGGAGATC
>KR806302;tax=d:Eukarya,p:Arthropoda,c:Insecta,o:Hymenoptera,f:Colletidae,g:Hylaeus,s:Hylaeus annulatus
AATATTATATTTCATTTTTGCTATATGAGCAGGAATAATTGGAACATCGTTAAGAATAAT
TATTCGAATAGAATTAAGAATACCTGGAATATGAATTAATAATGATCAAATATATAATAC
TATTGTTACATCACATGCATTTATTATAATTTTCTTTATAGTTATACCATTTATAATTGG
AGGATTCGGAAATTGATTAATTCCTATTATAATTGGAGCTCCAGATATAGCTTTTCCACG
AATAAATAATATAAGATTTTGATTATTACCACCATCTTTAATATTATTATTAATAAGAAG
AAGTTTATATTCAGGATCAGGTACTGGGTGAACAATTTATCCTCCATTATCATCTTCTAT
ATACCATCCTTCATTATCTGTAGACTTTACAATCTTTTCACTTCATATTGCTGGTATTTC
ATCTATTATAGGAGCTATTAATTTTATTGTAACAATTATAAATATAAAAAATATTAATTT
AAACTATGATCAAATAACTTTATTTTCATGATCAGTAATTATTACTGCAATTCTTTTACT
TTTATCTCTACCAGTTCTTGCTGGAGCAATTACTATATTATTAACAGATCGTAATTTAAA
TACATCATTTTTTGACCCTTCAGGTGGGG
>KR876866;tax=d:Eukarya,p:Arthropoda,c:Insecta,o:Hymenoptera,f:Colletidae,g:Hylaeus,s:Hylaeus annulatus
AATATTATATTTCATTTTTGCTATATGAGCAGGAATAATTGGAACATCGTTAAGAATAAT
TATTCGAATAGAATTAAGAATACCTGGAATATGAATTAATAATGATCAAATATATAATAC
TATTGTTACATCACATGCATTTATTATAATTTTCTTTATAGTTATACCATTTATAATTGG
AGGATTCGGAAATTGATTAATTCCTATTATAATTGGAGCTCCAGATATAGCTTTTCCACG
AATAAATAATATAAGATTTTGATTATTACCACCATCTTTAATATTATTATTAATAAGAAG
AAGTTTATACTCAGGATCAGGTACTGGGTGAACAATTTATCCTCCATTATCATCTTCTAT
ATACCATCCTTCATTATCTGTAGACTTTACAATCTTTTCACTTCATATTGCTGGTATTTC
ATCTATTATAGGAGCTATTAATTTTATTGTAACAATTATAAATATAAAAAATATTAATTT
AAACTATGATCAAATAACTTTATTTTCATGATCAGTAATTATTACTGCAATTCTTTTACT
TTTATCTCTACCAGTTCTTGCTGGAGCAATTACTATATTATTAACAGATCGTAATTTAAA
TACATCATTTTTTG
>KR878810;tax=d:Eukarya,p:Arthropoda,c:Insecta,o:Hymenoptera,f:Colletidae,g:Hylaeus,s:Hylaeus annulatus
AATATTATATTTCATTTTTGCTATATGAGCAGGAATAATTGGAACATCGTTAAGAATAAT
TATTCGAATAGAATTAAGAATACCTGGAATATGAATTAATAATGATCAAATATATAATAC
TATTGTTACATCACATGCATTTATTATAATTTTCTTTATAGTTATACCATTTATAATTGG
AGGATTCGGAAATTGATTAATTCCTATTATAATTGGAGCTCCAGATATAGCTTTTCCACG
AATAAATAATATAAGATTTTGATTATTACCACCATCTTTAATATTATTATTAATAAGAAG
AAGTTTATACTCAGGATCAGGTACTGGGTGAACAATTTATCCTCCATTATCATCTTCTAT
ATACCATCCTTCATTATCTGTAGACTTTACAATCTTTTCACTTCATATTGCTGGTATTTC
ATCTATTATAGGAGCTATTAATTTTATTGTAACAATTATAAATATAAAAAATATTAATTT
AAACTATGATCAAATAACTTTATTTTCATGATCAGTAATTATTACTGCAATTCTTTTACT
TTTATCTCTACCAGTTCTTGCTGGAGCAATTACTATATTATTAACAGATCGTAATTTAAA

TA
>KR887579;tax=d:Eukarya,p:Arthropoda,c:Insecta,o:Hymenoptera,f:Colletidae,g:Hylaeus,s:Hylaeus annulatus
AATATTATATTTCATTTTTGCTATATGAGCAGGAATAATTGGAACATCGTTAAGAATAAT
TATTCGAATAGAATTAAGAATACCTGGAATATGAATTAATAATGATCAAATATATAATAC
TATTGTTACATCACATGCATTTATTATAATTTTCTTTATAGTTATACCATTTATAATTGG
AGGATTCGGAAATTGATTAATTCCTATTATAATTGGAGCTCCAGATATAGCTTTTCCACG
AATAAATAATATAAGATTTTGATTATTACCACCATCTTTAATATTATTATTAATAAGAAG
AAGTTTATACTCAGGATCAGGTACTGGATGAACAATTTATCCTCCATTATCATCTTCTAT
ATACCATCCTTCATTATCTGTAGACTTTACAATCTTTTCACTTCATATTGCTGGTATTTC
ATCTATTATAGGAGCTATTAATTTTATTGTAACAATTATAAATATAAAAAATATTAATTT
AAACTATGATCAAATAACTTTATTTTCATGATCAGTAATTATTACTGCAATTCTTTTACT
TTTATCTCTACCAGTTCTTGCTGGAGCAATTACTATATTATTAACAGATCGTAATTTAAA
TACATG
>KR888197;tax=d:Eukarya,p:Arthropoda,c:Insecta,o:Hymenoptera,f:Colletidae,g:Hylaeus,s:Hylaeus annulatus
AATATTATATTTCATTTTTGCTATATGAGCAGGAATAATTGGAACATCATTAAGAATAAT
TATTCGAATAGAATTAAGAATACCTGGAATATGAATTAATAATGATCAAATATATAATAC
TATTGTTACATCACATGCATTTATTATAATTTTCTTTATAGTTATACCATTTATAATTGG
AGGATTCGGAAATTGATTAATTCCTATTATAATTGGAGCCCCAGATATAGCTTTTCCACG
AATAAATAATATAAGATTTTGATTATTACCACCATCTTTAATATTATTATTAATAAGAAG
AAGTTTATACTCAGGATCAGGTACTGGGTGAACAATTTATCCTCCATTATCATCTTCTAT
ATACCATCCTTCATTATCTGTAGACTTTACAATCTTTTCACTTCATATTGCTGGTATTTC
ATCTATTATAGGAGCTATTAATTTTATTGTAACAATTATAAATATAAAAAATATTAATTT
AAACTATGATCAAATAACTTTATTTTCATGATCAGTAATTATTACTGCAATTCTTTTACT
TTTATCTCTACCAGTTCTTGCTGGAGCAATTACTATATTATTAACAGATCGTAATTTAAA
TACATCATTTTTTGACCCTTCAGGTGGGGGAGATCCAATTCTATATCAACATTTATTT
>KR890572;tax=d:Eukarya,p:Arthropoda,c:Insecta,o:Hymenoptera,f:Colletidae,g:Hylaeus,s:Hylaeus annulatus
AATATTATATTTCATTTTTGCTATATGAGCAGGAATAATTGGAACATCGTTAAGAATAAT
TATTCGAATAGAATTAAGAATACCTGGAATATGAATTAATAATGATCAAATATATAATAC
TATTGTTACATCACATGCATTTATTATAATTTTCTTTATAGTTATACCATTTATAATTGG
AGGATTCGGAAATTGATTAATTCCTATTATAATTGGAGCTCCAGATATAGCTTTTCCACG
AATAAATAATATAAGATTTTGATTATTACCACCATCTTTAATATTATTATTAATAAGAAG
AAGTTTATACTCAGGATCAGGTACTGGGTGAACAATTTATCCTCCATTATCATCTTCTAT
ATACCATCCTTCATTATCTGTAGACTTTACAATCTTTTCACTTCATATTGCTGGTATTTC
ATCTATTATAGGAGCTATTAATTTTATTGTAACAATTATAAATATAAAAAATATTAATTT
AAACTATGATCAAATAACTTTATTTTCATGATCAGTAATTATTACTGCAATTCTTTTACT
TTTATCTCTACCAGTTCTTGCTGGAGCAATTACTATATTATTAACAGATCGTAATTTAAA
TACATCATTTTTTGACCCTTC
>KU496760;tax=d:Eukarya,p:Arthropoda,c:Insecta,o:Hymenoptera,f:Colletidae,g:Hylaeus,s:Hylaeus annulatus
AATATTATATTTCATTTTTGCTATATGAGCAGGAATAATTGGAACATCGTTAAGAATAAT
TATTCGAATAGAATTAAGAATACCTGGAATATGAATTAATAATGATCAAATATATAATAC
TATTGTTACATCACATGCATTTATTATAATTTTCTTTATAGTTATACCATTTATAATTGG
AGGATTCGGAAATTGATTAATTCCTATTATAATTGGAGCTCCAGATATAGCTTTTCCACG
AATAAATAATATAAGATTTTGATTATTACCACCATCTTTAATATTATTATTAATAAGAAG
AAGTTTATACTCAGGATCAGGTACTGGGTGAACAATTTATCCTCCATTATCATCTTCTAT
ATACCATCCTTCATTATCTGTAGACTTTACAATCTTTTCACTTCATATTGCTGGTATTTC
ATCTATTATAGGAGCTATTAATTTTATTGTAACAATTATAAATATAAAAAATATTAATTT
AAACTATGATCAAATAACTTTATTTTCATGATCAGTAATTATTACTGCAATTCTTTTACT
TTTATCTCTACCAGTTCTTGCTGGAGCAATTACTATATTATTAACAGATCGTAATTTAAA
TACATCATTTTTTGACCCTTCA
>MF902192;tax=d:Eukarya,p:Arthropoda,c:Insecta,o:Hymenoptera,f:Colletidae,g:Hylaeus,s:Hylaeus annulatus
AATATTATATTTCATTTTTGCTATATGAGCAGGAATAATTGGAACATCGTTAAGAATAAT

TATTCGAATAGAATTAAGAATACCTGGAATATGAATTAATAATGATCAAATATATAATAC
TATTGTTACATCACATGCATTTATTATAATTTTCTTTATAGTTATACCATTTATAATTGG
AGGATTCGGAAATTGATTAATTCCTATTATAATTGGAGCTCCAGATATAGCTTTTCCACG
AATAAATAATATAAGATTTTGATTATTACCACCATCTTTAATATTATTATTAATAAGAAG
AAGTTTATATTCAGGATCAGGTACTGGGTGAACAATTTATCCTCCATTATCATCTTCTAT
ATACCATCCTTCATTATCTGTAGACTTTACAATCTTTTCACTTCATATTGCTGGTATTTC
ATCTATTATAGGAGCTATTAATTTTATTGTAACAATTATAAATATAAAAAATATTA
>MF905413;tax=d:Eukarya,p:Arthropoda,c:Insecta,o:Hymenoptera,f:Colletidae,g:Hylaeus,s:Hylaeus annulatus
ATATTATATTTCATTTTTGCTATATGAGCAGGAATAATTGGAACATCATTAAGAATAATT
ATTCGAATAGAATTAAGAATACCTGGAATATGAATTAATAATGATCAAATATATAATACT
ATTGTTACATCACATGCATTTATTATAATTTTCTTTATAGTTATACCATTTATAATTGGA
GGATTCGGAAATTGATTAATTCCTATTATAATTGGAGCTCCAGATATAGCTTTTCCACGA
ATAAATAATATAAGATTTTGATTATTACCACCATCTTTAATATTATTATTAATAAGAAGA
AGTTTATACTCAGGATCAGGTACTGGGTGAACAATTTATCCTCCATTATCATCTTCTATA
TACCATCCTTCATTATCTGTAGACTTTACAATCTTTTCACTTCATATTGCTGGTATTTCA
TCTATTATAGGAGCTATTAATTTTATTGTAACAATTATAAATATAAAAAATATTAATTTA
AACTATGATCAAATAACTTTATTTTCATGATCAGTAATTATTACTGCAATTCTTTTACTT
TTATCTCTACCAGTTCTTGCTGGAGCAATTACTATA
>MG251092;tax=d:Eukarya,p:Arthropoda,c:Insecta,o:Hymenoptera,f:Apidae,g:Eucera,s:Eucera hamata
CAGGAAAATGGATTGGGAATGATCAATTATATAATTCATTTGTTACAGCTCATGCCTTTT
TAATGATTTTTTTTATAGTAATACCTTTTTTAATTGGAGGATTTGGAAATTGATTAATAC
CTTTAATATTAGGGTCTCCTGATATAGCATTCCCACGAATAAATAATATTAGATTTTGAT
TATTACCTTGTTCTTTATTATTTTTACTAATAAGAAATTTATTTAACATAACTCCTGGAA
CAGGATGAACTGTGTATCCTCCTTTATCTTCATATATATTTCATTCTTCTCCATCAGTAG
ATATTATAATTTTTTCTTTACATTTATCAGGTATATCATCTATTTTAGGTGCAATAAATT
TTATAGTAACTATTATAATAATGAAAAATTTATCAATAAATTATGATCAAATTAATTTAT
TTTCATGATCAGTATTTATTACAGCTATTTTGTTATTATTATCATTACCAGTTTTAGCAG
GAGCTATTACTATGTTATTATTTGATCGGAATTTAAATACTTCATTTTTTGATCCAATAG
GAGGAGGAGATCCTATTTTATATCAACATTTATTT
>MG251039;tax=d:Eukarya,p:Arthropoda,c:Insecta,o:Hymenoptera,f:Apidae,g:Florilegus,s:Florilegus condignus
CAGGACAATGATTAAATAATGATCAATTATATAATTCTTTTGTTACAGCTCATGCATTTT
TAATAATTTTTTTCATAGTCATACCTTTTTTAATTGGAGGGTTTGGAAATTGATTAGTTC
CTTTAATATTAGGCTGTCCTGATATAGCATTTCCTCGAATAAATAATATTAGTTTTTGAT
TATTACCCTGTTCCTTATTTTTATTAATTTTAAGAAATTTTTTTAATTTAACTCCAGGAA
CAGGTTGAACAGTATACCCCCCTTTATCATCTTACTTATTTCATTCATCTCCATCTGTTG
ATTTAATAATTTTTTCATTACACTTATCAGGAATTTCTTCTATTATAGGAGCTATAAATT
TTATGGTTACTATTATAATAATAAAAAATTTATCAATAAATTATGATCAAATTAATTTAT
TTTCATGATCAATTTTTATTACAGTTATTTTACTCTTATTATCATTACCAGTTTTAGCAG
GAGCAATTACTATATTATTATTTGATCGAAATTTAAATACTTCATTTTTTGACCCTATAG
GAGGAGGGGATCCAATTTTATACCAACATTTATTT
>MG251042;tax=d:Eukarya,p:Arthropoda,c:Insecta,o:Hymenoptera,f:Apidae,g:Melissodes,s:Melissodes agilis
CTGGTAAATGAATTGGTAATGATCAGTTGTATAATTCATTTGTAACAGCTCATGCTTTTT
TAATAATTTTTTTTATAGTAATACCTTTTTTAATTGGGGGTTTTGGAAATTGATTAATAC
CAATGATATTAGGATCTCCTGATATGGCTTTTCCACGAATAAATAATATTAGATTTTGAT
TATTACCTTGTTCTTTAATATTTTTATTATTAAGTAATTTATTTACTATAACTCCTGGAA
CTGGATGAACTATTTATCCTCCTTTGTCATTATATATATTTCATCCTTCACCTTCTGTTG
ATATTATAATTTTTTCTTTACATTTGTCAGGAATATCATCAATTTTAGGGGCAATAAATT
TTATAGTTACAATTATAATAATAAAAAATTTGTCAATAAATTTTGATCAGATTAATTTAT
TTTCCTGATCTCTTTTTATTACTGCAGTTTTATTATTATTATCTTTACCAGTATTAGCAG
GGGCTATTACTATATTGTTATTTGATCGAAATTTAAATACCTCTTTTTTTGATCCAATGG
GAGGAGGTGATCCAATTTTATATCAACATTTATTT

>MH687419;tax=d:Eukarya,p:Arthropoda,c:Insecta,o:Hymenoptera,f:Apidae,g:Melissodes,s:Melissodes communis
TGAATTAATAATGATCAATTATATAATTCATTTGTTACAGCTCATGCTTTTTTAATAATT
TTTTTTATGGTTATACCTTTTTTAATTGGGGGTTTTGGTAATTGATTAATACCAATAATA
TTAGGATCTCCTGATATAGCTTTCCCTCGAATAAATAATATTAGATTTTGATTACTTCCT
TGTTCTTTATTACTATTATTATTAAGGAATTTATTTAGGGTAACTCCGGGAACAGGTTGA
ACTATTTATCCTCCATTATCATCATATATTTTTCATCCTTCTCCTTCAGTTGATGTAATA
ATTTTTTCTTTACATTTATCTGGTATATCCTCAATTTTGGGGGCAATAAATTTTATGGTA
ACAATTATAATAATGAAAAATTACTCTATGAATTTTGATCAGATTAATTTATTTTCTTGA
TCATTATTTATTACAGCTGTTTTACTATTATTATCTTTACCAGTGTTGGCAGGTGCAATT
ACTATATTATTATTTGATCGTAATTTAAATACTTCATTTTTTGACCCTATAGGGGGTGGA
GATCCTATTTTGTATCAGCAT
>MH089843;tax=d:Eukarya,p:Arthropoda,c:Insecta,o:Hymenoptera,f:Apidae,g:Epeolus,s:Epeolus ainsliei
ATATATTATTTTTGCTTTATGATCTGGTTTATTGGGTTCATCTATAAGATTTTTAGTACG
TATAGAGTTATCTATTCCTGGTTCATGAATTATAAATGATCAAATTTATAATTCATTAAT
TACATCTCATGCTTTTTTAATGATTTTTTTTATAGTGATACCTTTTTTAATTGGTGGATT
TGGTAATTGATTGATTCCAATAATACTAGGAGCCCCTGATATAGCTTTTCCTCGAATAAA
TAATATTAGGTTTTGGTTATTACCTCCTTCATTATTTATATTATTATTGAGGAATTTGTT
TGTAATTATACCTGGAACAGGTTGAACTGTTTATCCTCCTTTATCAACATATTTATATCA
TAGATCTCCTTCTGTGGATTTTATAATTTTTTCTTTACATATATCAGGTATATCTTCAAT
TATAGGTGCTATAAATTTTATAGTTACAATTATAATAATGAAGAATTATTCAATAAATTA
TGATCAAATTAATTTATTTTCTTGATCAGTTTTTATTACAGCTATTTTATTACTTTTATC
TTTACCAGTATTAGCAGGAGCTATTACTATATTATTATTTGATCGAAATTTTAATACATG
TTTTTTTGATCCTATAGGTGGTGGTGATCCAGTATTGTATCAACATTTATTT
>MH089881;tax=d:Eukarya,p:Arthropoda,c:Insecta,o:Hymenoptera,f:Apidae,g:Epeolus,s:Epeolus ainsliei
ATATATTATTTTTGCTTTATGATCTGGTTTATTGGGTTCATCTATAAGATTTTTAGTACG
TATAGAGTTATCTATTCCTGGTTCATGAATTATAAATGATCAAATTTATAATTCATTAAT
TACATCTCATGCTTTTTTAATGATTTTTTTTATAGTGATACCTTTTTTAATTGGTGGATT
TGGTAATTGATTAATTCCAATAATACTAGGAGCCCCTGATATAGCTTTTCCTCGAATAAA
TAATATTAGGTTTTGGTTATTACCTCCTTCATTATTTATATTATTATTGAGGAATTTGTT
TGTAATTATACCTGGAACAGGTTGAACTGTTTATCCTCCTTTATCAACATATTTATATCA
TAGATCTCCTTCTGTGGATTTTATAATTTTTTCTTTACATATATCAGGTATATCTTCAAT
TATAGGTGCTATAAATTTTATAGTTACAATTATAATAATGAAGAATTATTCAATAAATTA
TGATCAAATTAATTTATTTTCTTGATCAGTTTTTATTACAGCTATTTTATTACTTTTATC
TTTACCAGTATTAGCAGGAGCTATTACTATATTATTATTTGATCGAAATTTTAATACATG
TTTTTTTGATCCTATGGGTGGTGGTGATCCAGTATTGTATCAACATTTATTT
>KJ164379;tax=d:Eukarya,p:Arthropoda,c:Insecta,o:Hymenoptera,f:Apidae,g:Epeolus,s:Epeolus lectoides
TTTATTATATATTATTTTTGCTTTATGATCTGGTTTATTGGGTTCATCTATAAGATTTTT
AGTACGTATAGAATTATCAGTTCCTGGTTCTTGAATTAGTAATGATCAAATTTATAATTC
ATTAATTACATCTCATGCTTTTTTAATAATTTTTTTTATAGTTATACCTTTTTTAATTGG
TGGTTTTGGTAATTGATTGATTCCTATAATATTAGGGGCTCCAGATATAGCTTTTCCTCG
AATAAATAATATTAGATTTTGGTTATTACCTCCTTCATTATTTTTATTATTATTAAGAAA
TTTATTTATAATTATACCTGGTACAGGTTGAACTGTTTATCCTCCTTTATCAACATATTT
ATATCATAATTCTCCTTCTGTTGATTTTATAATTTTTTCTTTACATATATCAGGTATATC
ATCAATTATAGGGGCTATAAATTTTATAGTTACAATTATAATAATAAAAAATTATTCGAT
AAATTATGATCAAATTAATTTATTTTCATGATCAGTTTTTATTACAGCTATTTTATTATT
ATTATCTTTACCAGTTTTAGCTGGTGCGATTACTATATTATTATTTGATCGTAATTTTAA
TACATGTTTTTTTGATCCAATAGGTGGTGGTGATCCAGTTTTATATCAACATTTATTT
>GU707508;tax=d:Eukarya,p:Arthropoda,c:Insecta,o:Hymenoptera,f:Apidae,g:Ceratina,s:Ceratina strenua
AATTTTATATATTATATTTGCTATATGATCAGGTATAATCGGAGCATCAATAAGATTAAT
TATTCGAATAGAATTAAGAACCCCGGGAAATTGAATTAATAATGATCAAATTTATAACTC
ATTAGTTACTGCTCATGCCTTTTTAATAATTTTTTTTATAGTTATACCATTTATAATTGG

GGGATTTGGAAATTGATTAATTCCATTAATATTAGGATCACCAGATATATCTTTTCCTCG
TTTAAATAATATTAGTTTTTGATTATTACCCCCATCATTACTACTATTATTATTAAGAAA
TTTATTTTCAATAAGACCAGGAACAGGATGAACTGTTTATCCACCTTTATCATCATATTT
ATTTCACCCATCTCCATCAGTAGACTTAGCTATTTTTTCCTTACATATATCAGGAATTTC
ATCAATTTTAGGAGCTATTAATTTTATAGTTACTATTATAATAATAAAAAATATTTCATT
AAATTATGACTCTATTCCATTATTTTCATGAGCAGTATTTATTACAGCAATTTTATTACT
TTTATCATTACCAGTATTAGCAGGAGCTATTACTATATTATTATTTGATCGAAATTTAAA
TACATCCTTTTTTGATCCTATAGGAGGTGGAGATCCAATTTTATACCAACATTTATTT
>KP747272;tax=d:Eukarya,p:Arthropoda,c:Insecta,o:Hymenoptera,f:Apidae,g:Ceratina,s:Ceratina strenua
CCCCGGGAAATTGAATTAATAATGATCAAATTTATAACTCATTAGTTACTGCTCATGCCT
TTTTAATAATTTTTTTTATAGTTATACCATTTATAATTGGGGGATTTGGAAATTGATTAA
TTCCATTAATATTAGGATCACCAGATATATCTTTTCCTCGTTTAAATAATATTAGTTTTT
GATTATTACCCCCATCATTACTACTATTATTATTAAGAAATTTATTTTCAATAAGACCAG
GAACAGGATGAACTGTTTATCCACCTTTATCATCATATTTATTTCACCCATCTCCATCAG
TAGATTTAGCTATTTTTTCCTTACATATATCAGGAATTTCATCAATTTTAGGAGCTATTA
ATTTTATAGTTACTATTATAATAATAAAAAATATTTCATTAAATTATGACTCTATTCCAT
TATTTTCATGAGCAGTATTTATTACAGCAATTTTATTACTTTTATCATTACCAGTATTAG
CAGGAGCTATTACTATATTATTATTTGATCGAAATTTAAATACATCCTTTTTTGATCCTA
TAGGAGGTGGAGATCCAATTTTATATCAACATTTA
>KP747273;tax=d:Eukarya,p:Arthropoda,c:Insecta,o:Hymenoptera,f:Apidae,g:Ceratina,s:Ceratina strenua
CCCCGGGAAATTGAATTAATAATGATCAAATTTATAACTCATTAGTTACTGCTCATGCCT
TTTTAATAATTTTTTTTATAGTTATACCATTTATAATTGGGGGATTTGGAAATTGATTAA
TTCCATTAATATTAGGATCACCAGATATATCTTTTCCTCGTTTAAATAATATTAGTTTTT
GATTATTACCCCCATCATTACTACTATTATTATTAAGAAATTTATTTTCAATAAGACCAG
GAACAGGATGAACTGTTTATCCACCTTTATCATCATATTTATTTCACCCATCTCCATCAG
TAGACTTAGCTATTTTTTCCTTACATATATCAGGAATTTCATCAATTTTAGGAGCTATTA
ATTTTATAGTTACTATTATAATAATAAAAAATATTTCATTAAATTATGACTCTATTCCAT
TATTTTCATGAGCAGTATTTATTACAGCAATTTTATTACTTTTATCATTACCAGTATTAG
CAGGAGCTATTACTATATTATTATTTGATCGAAATTTAAATACATCCTTTTTTGATCCTA
TAGGAGGTGGAGATCCAATTTTATACCAACATTTA
>KP747274;tax=d:Eukarya,p:Arthropoda,c:Insecta,o:Hymenoptera,f:Apidae,g:Ceratina,s:Ceratina strenua
CCCCGGGAAATTGAATTAATAATGATCAAATTTATAACTCATTAGTTACTGCTCATGCCT
TTTTAATAATTTTTTTTATAGTTATACCATTTATAATTGGGGGATTTGGAAATTGATTAA
TTCCATTAATATTAGGATCACCAGATATATCTTTTCCTCGTTTAAATAATATTAGTTTTT
GATTATTACCCCCATCATTACTACTATTATTATTAAGAAATTTATTTTCAATAAGGCCAG
GAACAGGATGAACTGTTTATCCACCTTTATCATCATATTTATTTCACCCATCTCCATCAG
TAGACTTAGCTATTTTTTCCTTACATATATCAGGAATTTCATCAATTTTAGGAGCTATTA
ATTTTATAGTTACTATTATAATAATAAAAAATATTTCATTAAATTATGACTCTATTCCAT
TATTTTCATGAGCAGTATTTATTACAGCAATTTTATTACTTTTATCATTACCAGTATTAG
CAGGAGCTATTACTATATTATTATTTGATCGAAATTTAAATACATCCTTTTTTGATCCTA
TAGGAGGTGGAGATCCAATTTTATACCAACATTTA
>KP747276;tax=d:Eukarya,p:Arthropoda,c:Insecta,o:Hymenoptera,f:Apidae,g:Ceratina,s:Ceratina strenua
CCCCAGGAAATTGAATTAATAATGATCAAATTTATAACTCATTAGTTACTGCTCATGCCT
TTTTAATAATTTTTTTTATAGTTATACCATTTATAATTGGGGGATTTGGAAATTGATTAA
TTCCATTAATATTAGGATCACCAGATATATCTTTTCCTCGTTTAAATAATATTAGTTTTT
GATTATTACCCCCATCATTACTACTATTATTATTAAGAAATTTATTTTCAATAAGACCAG
GAACAGGATGAACTGTTTATCCACCTTTATCATCATATTTATTTCACCCATCTCCATCAG
TAGACTTAGCTATTTTTTCCTTACATATATCAGGAATTTCATCAATTTTAGGAGCTATTA
ATTTTATAGTTACTATTATAATAATAAAAAATATTTCATTAAATTATGACTCTATTCCAT
TATTTTCATGAGCAGTATTTATTACAGCAATTTTATTACTTTTATCATTACCAGTATTAG
CAGGAGCTATTACTATATTATTATTTGATCGAAATTTAAATACATCCTTTTTTGATCCTA

TAGGAGGTGGAGATCCAATTTTATACCAACATTTA
>KP747278;tax=d:Eukarya,p:Arthropoda,c:Insecta,o:Hymenoptera,f:Apidae,g:Ceratina,s:Ceratina strenua
CCCCAGGAAATTGAATTAATAATGATCAAATTTATAACTCATTAGTTACTGCTCATGCCT
TTTTAATAATTTTTTTTATAGTTATACCATTTATAATTGGGGGATTTGGAAATTGATTAA
TTCCATTAATATTAGGATCACCAGATATATCTTTTCCTCGTTTAAATAATATTAGTTTTT
GATTATTACCCCCATCATTACTACTATTATTATTAAGAAATTTATTTTCAATAAGACCCG
GAACAGGATGAACTGTTTATCCACCTTTATCATCATATTTATTTCACCCATCTCCATCAG
TAGACTTAGCTATTTTTTCCTTACATATATCAGGAATTTCATCAATTTTAGGAGCTATTA
ATTTTATAGTTACTATTATAATAATAAAAAATATTTCATTAAATTATGACTCTATTCCAT
TATTTTCATGAGCAGTATTTATTACAGCAATTTTATTACTTTTATCATTACCAGTATTAG
CAGGAGCTATTACTATATTATTATTTGATCGAAATTTAAATACATCCTTTTTTGATCCTA
TAGGAGGTGGAGATCCAATTTTATACCAACATTTA
>KP747288;tax=d:Eukarya,p:Arthropoda,c:Insecta,o:Hymenoptera,f:Apidae,g:Ceratina,s:Ceratina strenua
CCCCGGGAAATTGAATTAATAATGATCAAATTTATAACTCATTAGTTACTGCTCATGCCT
TTTTAATAATTTTTTTTATAGTTATACCATTTATAATTGGGGGATTTGGAAATTGATTAA
TTCCATTAATATTAGGATCACCAGATATATCTTTTCCTCGTTTAAATAATATTAGTTTTT
GATTATTACCTCCATCATTACTACTATTATTATTAAGAAATTTATTTTCAATAAGACCAG
GAACAGGATGAACTGTTTATCCACCTTTATCATCATATTTATTTCACCCATCTCCATCAG
TAGACTTAGCTATTTTTTCCTTACATATATCAGGAATTTCATCAATTTTAGGAGCTATTA
ATTTTATAGTTACTATTATAATAATAAAAAATATTTCATTAAATTATGACTCTATTCCAT
TATTTTCATGAGCAGTATTTATTACAGCAATTTTATTACTTTTATCATTACCAGTATTAG
CAGGAGCTATTACTATATTATTATTTGATCGAAATTTAAATACATCCTTTTTTGATCCTA
TAGGAGGTGGAGATCCAATTTTATACCAACATTTA
>KP747291;tax=d:Eukarya,p:Arthropoda,c:Insecta,o:Hymenoptera,f:Apidae,g:Ceratina,s:Ceratina strenua
CCCCGGGAAATTGAATTAATAATGATCAAATTTATAACTCATTAGTTACTGCTCATGCCT
TTTTAATAATTATTTTTATAGTTATACCATTTATAATTGGGGGATTTGGAAATTGATTAA
TTCCATTAATATTAGGATCACCAGATATATCTTTTCCTCGTTTAAATAATATTAGTTTTT
GATTATTACCCCCATCATTACTACTATTATTATTAAGAAATTTATTTTCAATAAGACCAG
GAACAGGATGAACTGTTTATCCACCTTTATCATCATATTTATTTCACCCATCTCCATCAG
TAGACTTAGCTATTTTTTCCTTACATATATCAGGAATTTCATCAATTTTAGGAGCTATTA
ATTTTATAGTTACTATTATAATAATAAAAAATATTTCATTAAATTATGACTCTATTCCAT
TATTTTCATGAGCAGTATTTATTACAGCAATTTTATTACTTTTATCATTACCAGTATTAG
CAGGAGCTATTACTATATTATTATTTGATCGAAATTTAAATACATCCTTTTTTGATCCTA
TAGGAGGTGGAGATCCAATTTTATACCAACATTTA
>KP747294;tax=d:Eukarya,p:Arthropoda,c:Insecta,o:Hymenoptera,f:Apidae,g:Ceratina,s:Ceratina strenua
CCCCGGGAAATTGAATTAATAATGATCAAATTTATAACTCATTAGTTACTGCTCATGCCT
TTTTAATAATTTTTTTTATAGTTATACCATTTATAATTGGGGGATTTGGAAATTGATTAA
TTCCATTAATATTAGGATCACCAGATATATCTTTTCCTCGTTTAAATAATATTAGTTTTT
GATTATTACCCCCATCATTACTACTATTATTATTAAGAAATTTATTTTCAATAAGACCAG
GAACAGGATGAACTGTTTATCCACCTTTATCATCATATTTATTTCACCCATCTCCATCAG
TAGACTTAGCTATTTTTTCCTTACATATATCAGGAATTTCATCAATTTTAGGAGCTATTA
ATTTTATAGTTACTATTATAATAATAAAAAATATTTCATTAAATTATGACTCTATTCCAT
TATTTTCATGAGCAGTATTTATTACAGCAATTTTATTACTTTTATCATTACCAGTATTAG
CAGGAGCTATTACTATATTATTATTTGATCGAAATTTAAATACATCCTTTTTTGATCCTA
TAGGAGGTGGAGATCCAATTTTATATCAACATTTA
>KP747298;tax=d:Eukarya,p:Arthropoda,c:Insecta,o:Hymenoptera,f:Apidae,g:Ceratina,s:Ceratina strenua
CCCCAGGAAATTGAATTAATAATGATCAAATTTATAACTCATTAGTTACTGCTCATGCCT
TTTTAATAATTTTTTTTATAGTTATACCGTTTATAATTGGGGGATTTGGAAATTGATTAA
TTCCATTAATATTAGGATCACCAGATATATCTTTTCCTCGTTTAAATAATATTAGTTTTT
GATTATTACCCCCATCATTACTACTATTATTATTAAGAAATTTATTTTCAATAAGACCAG
GAACAGGATGAACTGTTTATCCACCTTTATCATCATATTTATTTCACCCATCTCCATCAG

TAGACTTAGCTATTTTTTCCTTACATATATCAGGAATTTCATCAATTTTAGGAGCTATTA
ATTTTATAGTTACTATTATAATAATAAAAAATATTTCATTAAATTATGACTCTATTCCAT
TATTTTCATGAGCAGTATTTATTACAGCAATTTTATTACTTTTATCATTACCAGTATTAG
CAGGAGCTATTACTATATTATTATTTGATCGAAATTTAAATACATCCTTTTTTGATCCTA
TAGGAGGTGGAGATCCAATTTTATACCAACATTTA
>AP018434;tax=d:Eukarya,p:Arthropoda,c:Insecta,o:Hymenoptera,f:Apidae,g:Apis,s:Apis mellifera
GATCTTGTATATTATTCTAGCTTTATGATCTGGAATACTAGGATCATCAATGAGACTTAT
TATTCGAATAGAATTAAGATCCCCAGGATCATGAATTAGCAATGATCAAATTTATAATAC
AATTGTTACTAGTCATGCATTCCTAATAATTTTTTTTATAGTTATACCATTTTTAATTGG
AGGATTTGGAAATTGGCTTATTCCTTTAATACTAGGATCACCTGATATAGCATTCCCCCG
AATAAATAATATTAGATTTTGATTACTTCCTCCCTCATTATTTATACTTTTATTAAGAAA
TTTATTTTATCCAAGACCAGGAACTGGATGAACAGTATATCCACCATTATCAGCATATTT
ATATCATTCTTCACCTTCAGTAGATTTTGCAATTTTTTCTCTTCATATATCAGGAATTTC
CTCAATTATAGGATCATTAAACTTAATAGTTACAATTATAATAATAAAAAATTTTTCTAT
AAATTATGACCAAATTTCATTATTTCCATGATCAGTTTTTATTACAGCAATTTTATTAAT
TATATCATTACCTGTATTAGCTGGAGCAATTACTATACTATTATTTGATCGAAATTTTAA
TACATCATTTTTCGATCCTATAGGAGGTGGAGATCCAATTCTTTATCAACATTTATTT
>MW428269;tax=d:Eukarya,p:Arthropoda,c:Insecta,o:Hymenoptera,f:Apidae,g:Apis,s:Apis mellifera
GATCTTATATATTATTTTAGCTTTATGATCTGGAATACTAGGATCATCAATGAGACTTAT
TATTCGAATAGAATTAAGATCCCCAGGATCATGAATTAATAATGATCAAATTTATAATAC
AATTGTTACTAGCCATGCATTTCTAATAATTTTTTTTATAGTTATACCATTTTTAATTGG
AGGATTTGGAAATTGGCTTATTCCTTTAATACTAGGATCACCTGATATAGCATTTCCTCG
AATAAATAATATTAGATTTTGATTACTTCCTCCCTCATTATTTATACTTTTATTAAGAAA
TTTATTTTACCCAAGACCAGGAACTGGATGAACAGTATATCCACCATTATCAGCATATTT
ATATCATTCTTCACCTTCAGTAGATTTTGCAATTTTTTCTCTTCATATATCAGGAATTTC
CTCAATTATAGGATCATTAAACTTAATAGTTACAATTATAATAATAAAAAATTTTTCTAT
AAATTATGATCAAATTTCATTATTTCCATGATCAGTTTTCATTACAGCAATTTTATTAAT
TATATCATTACCTGTATTAGCTGGAGCAATTACTATACTATTATTTGATCGAAATTTTAA
TACATCATTTTTCGATCCTATAGGAGGTGGAGATCCAATTTTATATCAACATTTATTT
>MT745914;tax=d:Eukarya,p:Arthropoda,c:Insecta,o:Hymenoptera,f:Apidae,g:Apis,s:Apis mellifera
GATCTTGTATATTATTCTAGCTTTATGATCTGGAATATTAGGTTCATCAATGAGACTTAT
TATTCGAATAGAATTAAGATCTCCAGGATCATGAATTAATAATGATCAAATTTATAATAC
AATTGTTACTAGCCATGCATTTCTAATAATTTTTTTTATAGTTATACCATTTTTAATTGG
AGGATTTGGAAATTGGCTTATTCCTTTAATATTAGGATCACCTGATATAGCATTTCCTCG
AATAAATAATATTAGATTTTGATTACTTCCTCCTTCATTATTTATACTTTTATTAAGAAA
TTTATTTTACCCAAGACCAGGAACTGGATGAACAGTATATCCCCCATTATCAGCATATTT
ATATCATTCTTCACCTTCAGTAGATTTTGCAATTTTTTCTCTTCATATATCAGGAATTTC
CTCAATTATAGGATCATTAAATTTAATAGTTACAATTATAATAATAAAAAATTTTTCTAT
AAATTATGACCAAATTTCATTATTTCCATGATCAGTTTTTATTACAGCAATTTTATTAAT
TATATCATTACCTGTATTAGCTGGAGCAATTACTATACTATTATTTGATCGAAATTTTAA
TACATCATTTTTTGATCCTATAGGAGGTGGAGATCCAATTTTATATCAACATTTATTT
>MW428278;tax=d:Eukarya,p:Arthropoda,c:Insecta,o:Hymenoptera,f:Apidae,g:Apis,s:Apis mellifera
GATCTTATATATTATTTTAGCTTTATGATCTGGAATACTAGGATCATCAATGAGACTTAT
TATTCGAATAGAATTAAGATCCCCAGGATCATGAATTAATAATGATCAAATTTATAATAC
AATTGTTACTAGCCATGCATTTCTAATAATTTTTTTTATAGTTATACCATTTTTAATTGG
AGGATTTGGAAATTGGCTTATTCCTTTAATATTAGGATCACCTGATATAGCATTTCCTCG
AATAAATAATATTAGATTTTGATTACTTCCTCCCTCATTATTTATACTTTTATTAAGAAA
TTTATTTTACCCAAGACCAGGAACTGGATGAACAGTATATCCACCATTATCAGCATATTT
ATATCATTCTTCACCTTCAGTAGATTTTGCAATTTTTTCTCTTCATATATCAGGAATTTC
CTCAATTATAGGATCATTAAACTTAATAGTTACAATTATAATAATAAAAAATTTTTCTAT
AAATTATGATCAAATTTCATTATTTCCATGATCAGTTTTCATTACAGCAATTTTATTAAT

TATATCATTACCTGTATTAGCTGGAGCAATTACTATACTATTATTTGATCGAAATTTTAA
TACATCATTTTTCGATCCTATAGGAGGTGGAGATCCAATTTTATATCAACATTTATTT
>AP018432;tax=d:Eukarya,p:Arthropoda,c:Insecta,o:Hymenoptera,f:Apidae,g:Apis,s:Apis mellifera
GATCTTGTATATTATTCTAGCTTTATGATCTGGAATACTAGGATCATCAATGAGACTTAT
TATTCGAATAGAATTAAGATCCCCAGGATCATGAATTAGCAATGATCAAATTTATAATAC
AATTGTTACTAGTCATGCATTCCTAATAATTTTTTTTATAGTTATACCATTTTTAATTGG
AGGATTTGGAAATTGGCTTATTCCTTTAATACTAGGATCACCTGATATAGCATTCCCCCG
AATAAATAATATTAGATTTTGATTACTTCCTCCATCATTATTTATACTTTTATTAAGAAA
TTTATTTTATCCAAGACCAGGAACTGGATGAACAGTATATCCACCATTATCAGCATATTT
ATATCATTCTTCACCTTCAGTAGATTTTGCAATTTTTTCTCTTCATATATCAGGAATTTC
CTCAATTATAGGATCATTAAACTTAATAGTTACAATTATAATAATAAAAAATTTTTCTAT
AAATTATGACCAAATTTCATTATTTCCATGATCAGTTTTTATTACAGCAATTTTATTAAT
TATATCATTACCTGTATTAGCTGGAGCAATTACTATACTATTATTTGATCGAAATTTTAA
TACATCATTTTTCGATCCTATAGGAGGTGGAGATCCAATTCTTTATCAACATTTATTT
>MK091915;tax=d:Eukarya,p:Arthropoda,c:Insecta,o:Hymenoptera,f:Apidae,g:Apis,s:Apis mellifera
GATCTTGTATATTATTCTAGCTTTATGATCTGGAATACTAGGATCATCAATGAGACTTAT
TATTCGAATAGAATTAAGATCCCCAGGATCATGAATTAGCAATGATCAAATTTATAATAC
AATTGTTACTAGTCATGCATTCCTAATAATTTTTTTTATAGTTATACCATTTTTAATTGG
AGGATTTGGAAATTGGCTTATTCCTTTAATACTAGGATCACCTGATATAGCATTCCCCCG
AATAAATAATATTAGATTTTGATTACTTCCTCCCTCATTATTTATACTTTTATTAAGAAA
TTTATTTTATCCAAGACCAGGAACTGGATGAACAGTATATCCACCATTATCAGCATATTT
ATATCATTCTTCACCTTCAGTAGATTTTGCAATTTTTTCTCTTCATATATCAGGAATTTC
CTCAATTATAGGATCATTAAACTTAATAGTTACAATTATAATAATAAAAAATTTTTCTAT
AAATTATGACCAAATTTCATTATTTCCATGATCAGTTTTTATTACAGCAATTTTATTAAT
TATATCATTACCTGTATTAGCTGGAGCAATTACTATACTATTATTTGATCGAAATTTTAA
TACATCATTTTTCGATCCTATAGG
>KP844924;tax=d:Eukarya,p:Arthropoda,c:Insecta,o:Hymenoptera,f:Apidae,g:Apis,s:Apis mellifera
TTAAGATCCCCAGGATCATGAATTAATAATGATCAAATTTATAATACAATTGTTACTAGC
CATGCATTTCTAATAATTTTTTTTATAGTTATACCATTTTTAATTGGAGGATTTGGAAAT
TGGCTTATTCCTTTAATACTAGGATCACCTGATATAGCATTTCCCCGAATAAATAATATT
AGATTTTGATTACTTCCTCCCTCATTATTTACACTTTTATTAAGAAATTTATTTTACCCA
AGACCAGGAACTGGATGAACAGTATATCCACCATTATCAGCATATTTATATCATTCTTCA
CCTTCAGTAGATTTTACAATTTTTTCTCTTCATATATCAGGAATTTCCTCAATTATAGGA
TCATTAAATTTAATAGTTACAATTATAATAATAAAAAATTTTTCTATAAATTATGACCAA
ATTTCATTATTTCCATGATCAGTTTTTATTACAGCAATTTTATTAATTATATCACTACCT
GTATTAGCTGGAGCAATTACTATACTATTATTTGATCGAAATTTTAATACATCATTTTTC
GATCCTATAGGAGGTGGAGATCCAATTCTTTATCAACATTTATTT
>KP844945;tax=d:Eukarya,p:Arthropoda,c:Insecta,o:Hymenoptera,f:Apidae,g:Apis,s:Apis mellifera
TTAAGATCCCCAGGATCATGAATTAATAATGATCAAATTTATAATACAATTGTTACTAGC
CATGCATTTCTAATAATTTTTTTTATAGTTATACCATTTTTAATTGGAGGATTTGGAAAT
TGGCTTATTCCTTTAATACTAGGATCACCTGATATAGCATTTCCTCGAATAAATAATATT
AGATTTTGATTACTTCCTCCCTCATTATTTACACTTTTATTAAGAAATTTATTTTACCCA
AGACCAGGAACTGGATGAACAGTATATCCACCATTATCAGCATATTTATATCATTCTTCA
CCTTCAGTAGATTTTACAATTTTTTCTCTTCATATATCAGGAATTTCCTCAATTATAGGA
TCATTAAATTTAATAGTTACAATTATAATAATAAAAAATTTTTCTATAAATTATGACCAA
ATTTCATTATTTCCATGATCAGTTTTTATTACAGCAATTTTATTAATTATATCACTACCT
GTATTAGCTGGAGCAATTACTATACTATTATTTGATCGAAATTTTAATACATCATTTTTC
GATCCTATAGGAGGTGGAGATCCAATTCTTTATCAACATTTATTT
>MF100915;tax=d:Eukarya,p:Arthropoda,c:Insecta,o:Hymenoptera,f:Apidae,g:Apis,s:Apis mellifera
GATCTTGTATATTATTCTAGCTTTATGATCTGGAATACTAGGATCATCAATGAGACTTAT
TATTCGAATAGAATTAAGATCCCCAGGATCATGAATTAACAATGATCAAATTTATAATAC

AATTGTTACTAGTCATGCATTCCTAATAATTTTTTTTATAGTTATACCATTTTTAATTGG
AGGATTTGGAAATTGGCTTATTCCTTTAATACTAGGATCACCTGATATAGCATTCCCCCG
AATAAATAATATTAGATTTTGATTACTTCCTCCCTCATTATTTATACTTTTATTAAGAAA
TTTATTTTATCCAAGACCAGGAACTGGATGAACAGTATATCCACCATTATCAGCATATTT
ATATCATTCTTCACCTTCAGTAGATTTTGCAATTTTTTCTCTTCATATATCAGGAATTTC
CTCAATTATAGGATCATTAAACTTAATAGTTACAATTATAATAATAAAAAATTTTTCTAT
AAATTATGACCAAATTTCATTATTTCCATGATCAGTTTTTATTACAGCAATTTTATTAAT
TATATCATTACCTGTATTAGCTGGAGCAATTACTATACTATTATTTGATCGAAATTTTAA
TACATCATTTTTCGATCCTATAGGAGGTGGAGATCCAATTCTTTATCAACATTTATTT
>MF934891;tax=d:Eukarya,p:Arthropoda,c:Insecta,o:Hymenoptera,f:Apidae,g:Apis,s:Apis mellifera
ATTATTCTAGCTTTATGATCTGGAATACTAGGATCATCAATGAGACTTATTATTCGAATA
GAATTAAGATCCCCAGGATCATGAATTAGCAATGATCAAATTTATAATACAATTGTTACT
AGTCATGCATTCCTAATAATTTTTTTTATAGTTATACCATTTTTAATTGGAGGATTTGGA
AATTGGCTTATTCCTTTAATACTAGGATCACCTGATATAGCATTCCCCCGAATAAATAAT
ATTAGATTTTGATTACTTCCTCCCTCATTATTTATACTTTTATTAAGAAATTTATTTTAT
CCAAGACCAGGAACTGGATGAACAGTATATCCACCATTATCAGCATATTTATATCATTCT
TCACCTTCAGTAGATTTTGCAATTTTTTCTCTTCATATATCAGGAATTTCCTCAATTATA
GGATCATTAAACTTAATAGTTACAATTATAATAATAAAAAATTTTTCTATAAATTATGAC
CAAATTTCATTATTTCCATGATCAGTTTTTATTACAGCAATTTTATTAATTATATCATTA
CCTGTATTAGCTGGAGCAATTACTATA
>MG438859;tax=d:Eukarya,p:Arthropoda,c:Insecta,o:Hymenoptera,f:Apidae,g:Apis,s:Apis mellifera
CTAGCTTTATGATCTGGAATACTAGGATCATCAATGAGACTTATTATTCGAATAGAATTA
AGATCCCCAGGATCATGAATTAGCAATGATCAAATTTATAATACAATTGTTACTAGTCAT
GCATTCCTAATAATTTTTTTTATAGTTATACCATTTTTAATTGGAGGATTTGGAAATTGG
CTTATTCCTTTAATACTAGGATCACCTGATATAGCATTCCCCCGAATAAATAATATTAGA
TTTTGATTACTTCCTCCCTCATTATTTATACTTTTATTAAGAAATTTATTTTATCCAAGA
CCAGGAACTGGATGAACAGTATATCCACCATTATCAGCATATTTATATCATTCTTCACCT
TCAGTAGATTTTGCAATTTTTTCTCTTCATATATCAGGAATTTCCTCAATTATAGGATCA
TTAAACTTAATAGTTACAATTATAATAATAAAAAATTTTTCTATAAATTATGACCAAATT
TCATTATTTCCATGATCAGTTTTTATTACAGCAATTTTATTAATTATATCATTACCTGTA
TTAGCTGGAGCAATTACTATACTATTATTTGATCGA
>MG439198;tax=d:Eukarya,p:Arthropoda,c:Insecta,o:Hymenoptera,f:Apidae,g:Apis,s:Apis mellifera
GGATCATCAATGAGACTTATTATTCGAATAGAATTAAGATCCCCAGGATCATGAATTAGC
AATGATCAAATTTATAATACAATTGTTACTAGTCATGCATTCCTAATAATTTTTTTTATA
GTTATACCATTTTTAATTGGAGGATTTGGAAATTGGCTTATTCCTTTAATACTAGGATCA
CCTGATATAGCATTCCCCCGAATAAATAATATTAGATTTTGATTACTTCCTCCCTCATTA
TTTATACTTTTATTAAGAAATTTATTTTATCCAAGACCAGGAACTGGATGAACAGTATAT
CCACCATTATCAGCATATTTATATCATTCTTCACCTTCAGTAGATTTTGCAATTTTTTCT
CTTCATATATCAGGAATTTCCTCAATTATAGGATCATTAAACTTAATAGTTACAATTATA
ATAATAAAAAATTTTTCTATAAATTATGACCAAATTTCATTATTTCCATGATCAGTTTTT
ATTACAGCAATTTTATTAATTATATCATTACCTGTATTAGCTGGAGCAATTACTATACTA
TTATTTGAT
>MG439357;tax=d:Eukarya,p:Arthropoda,c:Insecta,o:Hymenoptera,f:Apidae,g:Apis,s:Apis mellifera
ATCTTGTATATTATTCTAGCTTTATGATCTGGAATACTAGGATCATCAATGAGACTTATT
ATTCGAATAGAATTAAGATCCCCAGGATCATGAATTAGCAATGATCAAATTTATAATACA
ATTGTTACTAGTCATGCATTCCTAATAATTTTTTTTATAGTTATACCATTTTTAATTGGA
GGATTTGGAAATTGGCTTATTCCTTTAATACTAGGATCACCTGATATAGCATTCCCCCGA
ATAAATAATATTAGATTTTGATTACTTCCTCCCTCATTATTTATACTTTTATTAAGAAAT
TTATTTTATCCAAGACCAGGAACTGGATGAACAGTATATCCACCATTATCAGCATATTTA
TATCATTCTTCACCTTCAGTAGATTTTGCAATTTTTTCTCTTCATATATCAGGAATTTCC
TCAATTATAGGATCATTAAACTTAATAGTTACAATTATAATAATAAAAAATTTTTCTATA

AATTATGACCAAATTTCATTATTTCCATGATCAGTTTTTATTACAGCAATTTTATTAATT
ATATCATTACCTGTATTAGCTGGAGCAATTACTATACTATTATTTGAT
>MG439759;tax=d:Eukarya,p:Arthropoda,c:Insecta,o:Hymenoptera,f:Apidae,g:Apis,s:Apis mellifera
ATTATTCTAGCTTTATGATCTGGAATACTAGGATCATCAATGAGACTTATTATTCGAATA
GAATTAAGATCCCCAGGATCATGAATTAACAATGATCAAATTTATAATACAATTGTTACT
AGTCATGCATTCCTAATAATTTTTTTTATAGTTATACCATTTTTAATTGGAGGATTTGGA
AATTGGCTTATTCCTTTAATACTAGGATCACCTGATATAGCATTCCCCCGAATAAATAAT
ATTAGATTTTGATTACTTCCTCCCTCATTATTTATACTTTTATTAAGAAATTTATTTTAT
CCAAGACCAGGAACTGGATGAACAGTATATCCACCATTATCAGCATATTTATATCATTCT
TCACCTTCAGTAGATTTTGCAATTTTTTCTCTTCATATATCAGGAATTTCCTCAATTATA
GGATCATTAAACTTAATAGTTACAATTATAATAATAAAAAATTTTTCTATAAATTATGAC
CAAATTTCATTATTTCCATGATCAGTTTTTATTACAGCAATTTTATTAATTATATCATTA
CCTGTATTAGCTGGAGCAATTACTATACTATTATTTGATCGA
>MH138083;tax=d:Eukarya,p:Arthropoda,c:Insecta,o:Hymenoptera,f:Apidae,g:Apis,s:Apis mellifera
ATTTTAATTGGAGGATTTGGAAATTGACTTATTCCTTTAATACTAGGATCACCTGATATA
GCATTTCCTCGAATAAATAATATTAGATTTTGATTACTTCCTCCCTCATTATTTATACTT
TTATTAAGAAATTTATTTTATCCAAGACCAGGAACTGGGTGAACAGTATATCCACCATTA
TCAGCATATTTATATCATTCTTCACCTTCAGTAGATTTTGCAATTTTTTCTCTTCATATA
TCAGGAATTTCCTCAATTATAGGATCATTAAACTTAATAGTTACAATTATAATAATAAAA
AATTTTTCTATAAATTATGACCAAATTTCATTATTTCCATGATCAGTTTTCATTACAGCA
ATTTTATTAATTATATCATTACCTGTATTAGCTGGAGCAATTACTATACTATTATTTGAT
CGAAATTTTAATACATCATTTTTCGATCCTATAGGAGGTGGAGATCCAATTTTATATCAA
CATTTATTT
>MH388489;tax=d:Eukarya,p:Arthropoda,c:Insecta,o:Hymenoptera,f:Apidae,g:Apis,s:Apis mellifera
TTGCATATTATTCTAGCTTTATGATCTGGAATACTAGGATCATCAATGAGACTTATTATT
CGAATAGAATTAAGATCCCCAGGATCATGAATTAACAATGATCAAATTTATAATACAATT
GTTACTAGTCATGCATTCCTAATAATTTTTTTTATAGTTATACCATTTTTAATTGGAGGA
TTTGGAAATTGGCTTATTCCTTTAATACTAGGATCACCTGATATAGCATTCCCCCGAATA
AATAATATTAGATTTTGATTACTTCCTCCCTCATTATTTATACTTTTATTAAGAAATTTA
TTTTATCCAAGACCAGGAACTGGATGAACAGTATATCCACCATTATCAGCATATTTATAT
CATTCTTCACCTTCAGTAGATTTTGCAATTTTTTCTCTTCATATATCAGGAATTTCCTCA
ATTATAGGATCATTAAACTTAATAGTTACAATTATAATAATAAAAAATTTTTCTATAAAT
TATGACCAAATTTCATTATTTCCATGATCAGTTTTTATTACAGCAATTTTATTAATTATA
TCATTACCTGTATTAGCTGGAGCAATTACTATACTATTATTTGATCGAAATTTTAATACA
TCATTTTTCGATCCTATAGGAGGTGGAGATCCAATTCTTTATCAACATTTATTT
>MH687425;tax=d:Eukarya,p:Arthropoda,c:Insecta,o:Hymenoptera,f:Apidae,g:Apis,s:Apis mellifera
AGGATCATGAATTAGCAATGATCAAATTTATAATACAATTGTTACTAGTCATGCATTCCT
AATAATTTTTTTTATAGTTATACCATTTTTAATTGGAGGATTTGGAAATTGGCTTATTCC
TTTAATACTAGGATCACCTGATATAGCATTCCCCCGAATAAATAATATTAGATTTTGATT
ACTTCCTCCCTCATTATTTATACTTTTATTAAGAAATTTATTTTATCCAAGACCAGGAAC
TGGATGAACAGTATATCCACCATTATCAGCATATTTATATCATTCTTCACCTTCAGTAGA
TTTTGCAATTTTTTCTCTTCATATATCAGGAATTTCCTCAATTATAGGATCATTAAACTT
AATAGTTACAATTATAATAATAAAAAATTTTTCTATAAATTATGACCAAATTTCATTATT
TCCATGATCAGTTTTTATTACAGCAATTTTATTAATTATATCATTACCTGTATTAGCTGG
AGCAATTACTATACTATTATTTGATCGAAATTTTAATACATCATTTTTCGATCCTATAGG
AGGTGGAGATCCAATTCTTTATCAACAT
>MK030002;tax=d:Eukarya,p:Arthropoda,c:Insecta,o:Hymenoptera,f:Apidae,g:Apis,s:Apis mellifera
GATCTTGTATATTATTCTAGCTTTATGATCTGGAATACTAGGATCATCAATGAGACTTAT
TATTCGAATAGAATTAAGATCCCCAGGATCATGAATTAATAATGATCAAATTTATAATAC
AATTGTTACTAGCCATGCATTCCTAATAATTTTTTTTATAGTTATACCATTTTTAATTGG
AGGATTTGGAAATTGGCTTATTCCTTTAATACTAGGATCACCTGATATAGCATTTCCCCG

AATAAATAATATTAGATTTTGATTACTTCCTCCCTCATTATTTACACTTTTATTAAGAAA
TTTATTTTACCCAAGACCAGGAACTGGATGAACAGTATATCCACCATTATCAGCATATTT
ATATCATTCTTCACCTTCAGTAGATTTTGCAATTTTTTCTCTTCATATATCAGGAATTTC
CTCAATTATAGGATCATTAAACTTAATAGTTACAATTATAATAATAAAAAATTTTTCTAT
AAATTATGACCAAATTTCATTATTTCCATGATCAGTTTTTATTACAGCAATTTTATTAAT
TATATCACTACCTGTATTAGCTGGAGCAATTACTATACTATTATTTGATCGAAATTTTAA
TACATCATTTTTCGATCCTATAGGAGGTGGAGATCCAATTTTATATCAACATTTATTT
>MK779172;tax=d:Eukarya,p:Arthropoda,c:Insecta,o:Hymenoptera,f:Apidae,g:Apis,s:Apis mellifera
TGAGACTTATTATTCGAATAGAATTAAGATCCCCAGGATCATGAATTACCAATGATCAAA
TTTATAATACAATTGTTACTAGTCATGCATTCCTAATAATTTTTTTTATAGTTATACCAT
TTTTAATTGGAGGATTTGGAAATTGGCTTATTCCTTTAATACTAGGATCACCTGATATAG
CATTCCCCCGAATAAATAATATTAGATTTTGATTACTTCCTCCCTCATTATTTATACTTT
TATTAAGAAATTTATTTTATCCAAGACCAGGAACTGGATGAACAGTATATCCACCATTAT
CAGCATATTTATATCATTCTTCACCTTCAGTAGATTTTGCAATTTTTTCTCTTCATATAT
CAGGAATTTCCTCAATTATAGGATCATTAAACTTAATAGTTACAATTATAATAATAAAAA
ATTTTTCTATAAATTATGACCAAATTTCATTATTTCCATGATCAGTTTTTATTACAGCAA
TTTTATTAATTATATCATTACCTGTATTAGCTGGAGCAATTACTATACTATTATTTGATC
GAAATTTTAATACATCATTTTTCGATCCTATAGGAGGTGGAGATCCAATTCTTTATCAAC
ATTTATTT
>KT164668;tax=d:Eukarya,p:Arthropoda,c:Insecta,o:Hymenoptera,f:Halictidae,g:Halictus,s:Halictus rubicundus
GATACTTTACTTTATCTTTGCTATATGATCAGGAATAATCGGAGCCTCTTTAAGTATAAT
TATTCGTATAGAATTAAGAACACCTGGAAGATGAATTAATAATGATCAAATTTATAACAC
TATTGTAACTTCACATGCTTTTATTATAATTTTTTTTATAGTTATACCATTTATAATTGG
AGGATTTGGAAATTGATTAGTACCACTAATAATTGGAGCTCCCGATATAGCATTCCCACG
TATAAATAATATAAGATTTTGATTATTAATCCCCTCATTATTTATACTTATAATAAGAAG
AACTTTATCTACAGGATCGGGAACAGGTTGAACTATTTACCCCCCTCTATCTTCAATTAT
ATATCATTCATCTTTTTCTGTTGATTTTACTATCTTTTCTTTACATATTGCAGGTATTTC
CTCTATTATAGGGGCTATTAATTTTATTGTTTCAATCATATTAATAAAAAATATTTCACT
TAATATAAATCAAATCCCCTTATTTCCCTGATCAGTTAAAATTACTGCTATTTTACTTCT
TCTTTCTCTCCCTGTTTTAGCAGGTGCTATTACTATATTATTAACAGACCGAAATTTAAA
TACATCTTTTTTTGACCCTTCTGGAGGAGGAGACCCTATTCTTTATCAACATTTATTT
>MG341269;tax=d:Eukarya,p:Arthropoda,c:Insecta,o:Hymenoptera,f:Halictidae,g:Halictus,s:Halictus rubicundus
CTTTATTTTATCTTTGCTATATGATCAGGAATAATCGGAGCATCTTTAAGTATAATTATT
CGTATAGAATTAAGAACACCTGGAAGATGAATTAATAATGACCAAATTTATAATACTATT
GTAACTTCACATGCTTTTATTATAATTTTTTTTATAGTTATACCATTTATAATTGGAGGA
TTTGGAAATTGATTAGTACCACTAATAATTGGAGCTCCTGATATAGCTTTCCCACGTATA
AATAATATAAGATTTTGATTATTAATTCCTTCATTATTTATACTTATAATAAGAAGAACT
TTATCTACAGGATCAGGAACAGGTTGAACTATTTACCCTCCCCTATCTTCAATTATATAT
CACTCATCTTTTTCTGTTGATTTTACTATTTTTTCTTTACATATCGCAGGTATTTCTTCT
ATTATAGGTGCTATTAATTTTATTGTTTCAATTATATTAATAAAAAATATTTCACTTAAT
ATAAATCAAATCCCCTTATTTCCTTGATCAGTTAAAATTACTGCTATTTTACTTCTTCTT
TCTCTCCCTGTTTTAGCAGGTGCTATTACTATACTATTAACAGACCGAAATTTAAATACA
TCTTTTTTTGACCCTTCTGGAGGAGGAGACCCTATTCTTTACCAACATTTA
>GU690077;tax=d:Eukarya,p:Arthropoda,c:Insecta,o:Hymenoptera,f:Halictidae,g:Halictus,s:Halictus rubicundus
GATACTTTATTTTATCTTTGCTATATGATCAGGAATAATCGGAGCATCTTTAAGTATAAT
TATTCGTATAGAATTAAGAACACCTGGAAGATGAATTAATAATGATCAAATTTATAATAC
TATTGTAACTTCACATGCTTTTATTATAATTTTTTTTATAGTTATACCATTTATAATTGG
AGGATTTGGAAATTGATTAGTACCACTAATAATTGGAGCTCCTGATATAGCTTTCCCACG
TATAAATAATATAAGATTTTGATTATTAATTCCTTCATTATTTATACTTATAATAAGAAG
AACTTTATCTACAGGATCAGGAACAGGTTGAACTATTTACCCTCCCCTATCTTCAATTAT
ATACCACTCATCTTTTTCTGTTGATTTTACTATTTTTTCTTTACATATCGCAGGTATTTC

TTCTATTATAGGTGCTATTAATTTTATTGTTTCAATTATATTAATAAAAAATATTTCACT
TAATATAAATCAAATCCCCTTATTTCCTTGATCAGTTAAAATTACTGCTATTTTACTTCT
TCTTTCTCTCCCTGTTTTAGCAGGTGCTATTACTATACTATTAACAGACCGAAATTTAAA
TACATCTTTTTTTGACCCTTCTGGAGGAGGAGACCCTATTCTTTACCAACATTTATTC
>HQ929857;tax=d:Eukarya,p:Arthropoda,c:Insecta,o:Hymenoptera,f:Halictidae,g:Halictus,s:Halictus rubicundus
AATACTTTATTTTATCTTTGCTATATGATCAGGAATAATCGGAGCATCTTTAAGTATAAT
TATTCGTATAGAATTAAGAACACCTGGAAGATGAATTAATAACGACCAAATTTATAATAC
TATTGTAACTTCACATGCTTTTATTATAATTTTTTTTATAGTTATACCATTTATAATTGG
AGGATTTGGAAATTGATTAGTACCACTAATAATTGGGGCTCCTGATATAGCTTTCCCACG
TATAAATAATATAAGATTTTGATTATTAATTCCTTCATTATTTATACTTATAATAAGAAG
AACTTTATCTACAGGATCAGGAACAGGATGAACTATTTACCCTCCCCTATCTTCAATTAT
ATATCACTCATCCTTTTCTGTTGATTTTACTATTTTTTCTTTACATATCGCAGGTATTTC
TTCTATTATAGGTGCTATTAATTTTATTGTTTCAATTATATTAATAAAAAATATTTCACT
TAATATAAATCAAATCCCCTTATTTCCTTGATCAGTTAAAATTACTGCTATTTTACTTCT
TCTTTCTCTCCCTGTTTTAGCAGGTGCTATTACTATACTATTAACAGACCGAAATTTAAA
TACATCTTTTTTTGACCCTTCTGGAGGAGGAGACCCTATTCTTTACCAACATTTATTC
>JN293772;tax=d:Eukarya,p:Arthropoda,c:Insecta,o:Hymenoptera,f:Halictidae,g:Halictus,s:Halictus rubicundus
AATACTTTATTTTATCTTTGCTATATGATCAGGAATAATCGGAGCATCTTTAAGTATAAT
TATTCGTATAGAATTAAGAACACCTGGAAGATGAATTAATAATGATCAAATTTATAATAC
TATTGTAACTTCACATGCTTTTATTATAATTTTTTTTATAGTTATACCATTTATAATTGG
AGGATTTGGAAATTGATTAGTACCACTAATAATTGGAGCTCCTGATATAGCTTTCCCACG
TATAAATAATATAAGATTTTGATTATTAATTCCTTCATTATTTATACTTATAATAAGAAG
AACTTTATCTACAGGATCAGGAACAGGTTGAACTATTTACCCTCCCCTATCTTCAATTAT
ATATCACTCATCTTTTTCTGTTGATTTTACTATTTTTTCTTTACATATCGCAGGTATTTC
TTCTATTATAGGTGCTATTAATTTTATTGTTTCAATTATATTAATAAAAAATATTTCACT
TAATATAAATCAAATCCCCTTATTTCCTTGATCAGTTAAAATTACTGCTATTTTACTTCT
TCTTTCTCTCCCTGTTTTAGCAGGTGCTATTACTATACTATTAACAGACCGAAATTTAAA
TACATCTTTTTTTGACCCTTCTGGAGGAGGAGACCCTATTCTTTACCAACATTTATTC
>JN293775;tax=d:Eukarya,p:Arthropoda,c:Insecta,o:Hymenoptera,f:Halictidae,g:Halictus,s:Halictus rubicundus
GATACTTTATTTTATCTTTGCTATATGATCAGGAATAATCGGAGCATCTTTAAGTATAAT
TATTCGTATAGAATTAAGAACACCTGGAAGATGAATTAATAATGATCAAATTTATAATAC
TATTGTAACTTCACATGCTTTTATTATAATTTTTTTTATAGTTATACCATTTATAATTGG
AGGATTTGGAAATTGATTAGTACCACTAATAATTGGAGCTCCTGATATAGCTTTCCCACG
TATAAATAATATAAGATTTTGATTATTAATTCCTTCATTATTTATACTTATAATAAGAAG
AACTTTATCTACAGGATCAGGAACAGGTTGAACTATTTACCCTCCCCTATCTTCAATTAT
ATATCACTCATCTTTTTCTGTTGATTTTACTATTTTTTCTTTACATATCGCAGGTATTTC
TTCTATTATAGGTGCTATTAATTTTATTGTTTCAATTATATTAATAAAAAATATTTCACT
TAATATAAATCAAATCCCCTTATTTCCTTGATCAGTTAAAATTACTGCTATTTTACTTCT
TCTTTCTCTCCCTGTTTTAGCAGGTGCTATTACTATACTATTAACAGACCGAAATTTAAA
TACATCTTTTTTTGACCCTTCTGGGGGAGGAGACCCTATTCTTTACCAACATTTATTC
>JN293776;tax=d:Eukarya,p:Arthropoda,c:Insecta,o:Hymenoptera,f:Halictidae,g:Halictus,s:Halictus rubicundus
GATACTTTATTTTATCTTTGCTATATGATCAGGAATAATCGGAGCATCTTTAAGTATAAT
TATTCGTATAGAATTAAGAACACCTGGAAGATGAATTAATAATGATCAAATTTATAACAC
TATTGTAACTTCACATGCTTTTATTATAATTTTTTTTATAGTTATACCATTTATAATTGG
AGGATTTGGAAATTGATTAGTACCACTAATAATTGGAGCTCCTGATATAGCTTTCCCACG
TATAAATAATATAAGATTTTGATTATTAATTCCTTCATTATTTATACTTATAATAAGAAG
AACTTTATCTACAGGATCAGGAACAGGTTGAACTATTTACCCCCCCCTATCTTCAATTAT
ATATCACTCATCTTTTTCTGTTGATTTTACTATTTTTTCTTTACATATCGCAGGTATTTC
TTCTATTATAGGTGCTATTAATTTTATTGTTTCAATTATATTAATAAAAAATATTTCACT
TAATATAAATCAAATCCCCTTATTTCCTTGATCAGTTAAAATTACTGCTATTTTACTTCT
TCTTTCTCTCCCTGTTTTAGCAGGTGCTATTACTATACTATTAACAGACCGAAATTTAAA

TACATCTTTTTTTGACCCTTCTGGAGGAGGAGACCCTATTCTTTACCAACATTTATTC
>KJ837035;tax=d:Eukarya,p:Arthropoda,c:Insecta,o:Hymenoptera,f:Halictidae,g:Halictus,s:Halictus rubicundus
GATACTTTACTTTATCTTTGCTATATGATCAGGAATAATCGGAGCCTCTTTAAGTATAAT
TATTCGTATAGAATTAAGAACACCTGGAAGATGAATTAATAATGATCAAATTTATAACAC
TATTGTAACTTCACATGCTTTTATTATAATTTTTTTTATAGTTATACCATTTATAATTGG
AGGATTTGGAAATTGATTAGTACCACTAATAATTGGAGCTCCCGATATAGCATTCCCACG
TATAAATAATATAAGATTTTGATTATTAATCCCCTCATTATTTATACTTATAATAAGAAG
AACTTTATCTACAGGATCGGGAACAGGTTGAACTATTTACCCCCCTCTATCTTCAATTAT
ATATCATTCATCTTTTTCTGTTGATTTTACTATCTTTTCTTTACATATTGCAGGTATTTC
CTCTATTATAGGGGCTATTAATTTTATTGTTTCAATCATATTAATAAAAAATATTTCACT
TAATATAAATCAAATCCCCTTATTTCCCTGATCAGTTAAAATTACTGCTATTTTACTTCT
TCTTTCTCTCCCTGTTTTAGCAGGTGCTATTACTATATTATTAACAGACCGAAATTTAAA
TACATCTTTTTTTGACCCTTCTGGAGGAGGAGACCC
>KJ837110;tax=d:Eukarya,p:Arthropoda,c:Insecta,o:Hymenoptera,f:Halictidae,g:Halictus,s:Halictus rubicundus
AATACTTTACTTTATCTTTGCTATATGATCAGGAATAATCGGAGCCTCTTTAAGTATAAT
TATTCGTATAGAATTAAGAACACCTGGAAGATGAATTAATAATGATCAAATTTATAACAC
TATTGTAACTTCACATGCTTTTATTATAATTTTTTTTATAGTTATACCATTTATAATTGG
AGGATTTGGAAATTGATTAGTACCACTAATAATTGGAGCTCCTGATATAGCATTCCCACG
TATAAATAATATAAGATTTTGATTATTAATCCCCTCATTATTTATACTTATAATAAGAAG
AACTTTATCTACAGGATCAGGAACAGGTTGAACTATTTATCCCCCTCTATCTTCAATTAT
ATATCATTCATCTTTTTCTGTTGATTTTACTATCTTTTCTTTACATATCGCAGGTATTTC
CTCTATTATAGGAGCTATTAATTTTATTGTTTCAATTATATTAATAAAAAATATTTCACT
TAATATAAATCAAATCCCCTTATTTCCCTGATCAGTTAAAATTACTGCTATTTTACTTCT
TCTTTCTCTCCCTGTTTTAGCAGGTGCTATTACTATATTATTAACAGACCGAAATTTAAA
TACATCTTTTTTTGACCCTTCTGGAGGAGGAGACCCTATTCTTTATCAACATTTATTT
>KJ837973;tax=d:Eukarya,p:Arthropoda,c:Insecta,o:Hymenoptera,f:Halictidae,g:Halictus,s:Halictus rubicundus
GATACTTTACTTTATCTTTGCTATATGATCAGGAATAATCGGAGCCTCTTTAAGTATAAT
TATTCGTATAGAATTAAGAACACCTGGAAGATGAATTAATAATGATCAAATTTATAACAC
TATTGTAACTTCACATGCTTTTATTATAATTTTTTTTATAGTTATACCATTTATAATTGG
AGGATTTGGAAATTGATTAGTACCACTAATAATTGGAGCTCCCGATATAGCATTCCCACG
TATAAATAATATAAGATTTTGATTATTAATCCCCTCATTATTTATACTTATAATAAGAAG
AACTTTATCTACAGGATCAGGAACAGGTTGAACTATTTACCCCCCTCTATCTTCAATTAT
ATATCATTCATCTTTTTCTGTTGATTTTACTATCTTTTCTTTACATATTGCAGGTATTTC
CTCTATTATAGGGGCTATTAATTTTATTGTTTCAATCATATTAATAAAAAATATTTCACT
TAATATAAATCAAATCCCCTTATTTCCCTGATCAGTTAAAATTACTGCTATTTTACTTCT
TCTTTCTCTCCCTGTTTTAGCAGGTGCTATTACTATATTATTAACAGACCGAAATTTAAA
TACATCTTTTTTTGACCCTTCTGGAGGAGGAGACCCTATTCTTTATCAACATTTATTT
>KR787488;tax=d:Eukarya,p:Arthropoda,c:Insecta,o:Hymenoptera,f:Halictidae,g:Halictus,s:Halictus rubicundus
GATACTTTATTTTATCTTTGCTATATGATCAGGAATAATCGGAGCATCTTTAAGTATAAT
TATTCGTATAGAATTAAGAACACCTGGAAGATGAATTAATAATGATCAAATTTATAATAC
TATTGTAACTTCACATGCTTTTATTATAATTTTTTTTATAGTTATACCATTTATAATTGG
AGGATTTGGAAATTGATTAGTACCACTAATAATTGGAGCTCCTGATATAGCTTTCCCACG
TATAAATAATATAAGATTTTGATTATTAATTCCTTCATTATTTATACTTATAATAAGAAG
AACTTTATCTACAGGATCAGGAACAGGTTGAACTATTTACCCTCCCCTATCTTCAATTAT
ATATCACTCATCTTTTTCTGTTGATTTTACTATTTTTTCTTTACATATTGCAGGTATTTC
TTCTATTATAGGTGCTATTAATTTTATTGTTTCAATTATATTAATAAAAAATATTTCACT
TAATATAAATCAAATCCCCTTATTTCCTTGATCAGTTAAAATTACTGCTATTTTACTTCT
TCTTTCTCTCCCTGTTTTAGCAGGTGCTATTACTATACTATTAACAGACCGAAATTTAAA
TACATCTTTTTTTGACCCTTCTGGGGGAGGAGACCCTATTCTTTACCAACATTTATTC
>KR802136;tax=d:Eukarya,p:Arthropoda,c:Insecta,o:Hymenoptera,f:Halictidae,g:Halictus,s:Halictus rubicundus
ATACTTTATTTTATCTTTGCTATATGATCAGGAATAATCGGAGCATCTTTAAGTATAATT

ATTCGTATAGAATTAAGAACACCTGGAAGATGAATTAATAATGATCAAATTTATAATACT
ATTGTAACTTCACATGCTTTTATTATAATTTTTTTTATAGTTATACCATTTATAATTGGA
GGATTTGGAAATTGATTAGTACCACTAATAATTGGAGCTCCTGATATAGCTTTCCCACGT
ATAAATAATATAAGATTTTGATTATTAATTCCTTCATTATTTATACTTATAATAAGAAGA
ACTTTATCTACAGGATCAGGAACAGGTTGAACTATTTACCCTCCCCTATCTTCAATTATA
TACCACTCATCTTTTTCTGTTGATTTTACTATTTTTTCTTTACATATCGCAGGTATTTCT
TCTATTATAGGTGCTATTAATTTTATTGTTTCAATTATATTAATAAAAAATATTTCACTT
AATATAAATCAAATCCCCTTATTTCCTTGATCAGTTAAAATTACTGCTATTTTACTTCTT
CTTTCTCTCCCTGTTTTAGCAGGTGCTATTACTATACTATTAACAGACCGAAATTTAAAT
ACATCTTTTTTTGACCCTTCTGGAGGAGGAGACCCTATTCTTTACCAACATTTATTC
>KR873468;tax=d:Eukarya,p:Arthropoda,c:Insecta,o:Hymenoptera,f:Halictidae,g:Halictus,s:Halictus rubicundus
GATACTTTATTTTATCTTTGCTATATGATCAGGAATAATCGGAGCATCTTTAAGTATAAT
TATTCGTATAGAATTAAGAACACCTGGAAGATGAATTAATAATGATCAAATTTATAATAC
TATTGTAACTTCACATGCTTTTATTATAATTTTTTTTATAGTTATACCATTTATAATTGG
AGGATTTGGAAATTGATTAGTACCACTAATAATTGGAGCTCCTGATATAGCTTTCCCACG
TATAAATAATATAAGATTTTGATTATTAATTCCTTCATTATTTATACTTATAATAAGAAG
AACTTTATCTACAGGATCAGGAACAGGTTGAACTATTTACCCTCCCCTATCTTCAATTAT
ATATCACTCATCTTTTTCTGTTGATTTTACTATTTTTTCTTTACATATTGCAGGTATTTC
TTCTATTATAGGTGCTATTAATTTTATTGTTTCAATTATATTAATAAAAAATATTTCACT
TAATATAAATCAAATCCCCTTATTTCCTTGATCAGTTAAAATTACTGCTATTTTACTTCT
TCTTTCTCTCCCTGTTTTAGCAGGTGCTATTACTATACTATTAACAGACCGAAATTTAAA
TACATCTTTTT
>KR877730;tax=d:Eukarya,p:Arthropoda,c:Insecta,o:Hymenoptera,f:Halictidae,g:Halictus,s:Halictus rubicundus
GATACTTTATTTTATCTTTGCTATATGATCAGGAATAATCGGAGCATCTTTAAGTATAAT
TATTCGTATAGAATTAAGAACACCTGGAAGATGAATTAATAATGATCAAATTTATAATAC
TATTGTAACTTCACATGCTTTTATTATAATTTTTTTTATAGTTATACCATTTATAATTGG
AGGATTTGGAAATTGATTAGTACCACTAATAATTGGAGCTCCTGATATAGCTTTCCCACG
TATAAATAATATAAGATTTTGATTATTAATTCCTTCATTATTTATACTTATAATAAGAAG
AACTTTATCTACAGGATCAGGAACAGGTTGAACTATTTACCCTCCCCTATCTTCAATTAT
ATATCACTCATCTTTTTCTGTTGATTTTACTATTTTTTCTTTACATATCGCAGGTATTTC
TTCTATTATAGGTGCTATTAATTTTATTGTTTCAATTATATTAATAAAAAATATTTCACT
TAATATAAATCAAATCCCCTTATTTCCTTGATCAGTTAAAATTACTGCTATTTTACTTCT
TCTTTCTCTCCCTGTTTTAGCAGGTGCTATTACTATACTATTAACAGACCGAAATTTAAA
TACATCTTTTTTTGACCCTTCTGGGGGA
>KR888368;tax=d:Eukarya,p:Arthropoda,c:Insecta,o:Hymenoptera,f:Halictidae,g:Halictus,s:Halictus rubicundus
GATACTTTATTTTATCTTTGCTATATGATCAGGAATAATCGGAGCATCTTTAAGTATAAT
TATTCGTATAGAATTAAGAACACCTGGAAGATGAATTAATAATGATCAAATTTATAATAC
TATTGTAACTTCACATGCTTTTATTATAATTTTTTTTATAGTTATACCATTTATAATTGG
AGGATTTGGAAATTGATTAGTACCACTAATAATTGGAGCTCCTGATATAGCTTTCCCACG
TATAAATAATATAAGATTTTGATTATTAATTCCTTCATTATTTATACTTATAATAAGAAG
AACTTTATCTACAGGATCAGGAACAGGTTGAACTATTTACCCTCCCCTATCTTCAATTAT
ATATCACTCATCTTTTTCTGTTGATTTTACTATTTTTTCTTTACATATCGCAGGTATTTC
TTCTATTATAGGTGCTATTAATTTTATTGTTTCAATTATATTAATAAAAAATATTTCACT
TAATATAAATCAAATCCCCTTATTTCCTTGATCAGTTAAAATTACTGCTATTTTACTTCT
TCTTTCTCTCCCTGTTTTAGCAGGTGCTATTACTATACTATTAACAGACCGAAATTTAAA
TACATCTTTTTTTGACCCTTCTGGGGGAGGAGA
>KR890817;tax=d:Eukarya,p:Arthropoda,c:Insecta,o:Hymenoptera,f:Halictidae,g:Halictus,s:Halictus rubicundus
GATACTTTATTTTATCTTTGCTATATGATCAGGAATAATCGGAGCATCTTTAAGTATAAT
TATTCGTATAGAATTAAGAACACCTGGAAGATGAATTAATAATGATCAAATTTATAATAC
TATTGTAACTTCACATGCTTTTATTATAATTTTTTTTATAGTTATACCATTTATAATTGG
AGGATTTGGAAATTGATTAGTACCACTAATAATTGGAGCTCCTGATATAGCTTTCCCACG

TATAAATAATATAAGATTTTGATTATTAATTCCTTCATTATTTATACTTATAATAAGAAG
AACTTTATCTACAGGATCAGGAACAGGTTGAACTATTTACCCTCCCCTATCTTCAATTAT
ATATCACTCATCTTTTTCTGTTGATTTTACTATTTTTTCTTTACATATCGCAGGTATTTC
TTCTATTATAGGTGCTATTAATTTTATTGTTTCAATTATATTAATAAAAAATATTTCACT
TAATATAAATCAAATCCCCTTATTTCCTTGATCAGTTAAAATTACTGCTATTTTACTTCT
TCTTTCTCTCCCTGTTTTAGCAGGTGCTATTACTATACTATTAACAGACCGAAATTTAAA
TACATCTTTTTTT
>KR897852;tax=d:Eukarya,p:Arthropoda,c:Insecta,o:Hymenoptera,f:Halictidae,g:Halictus,s:Halictus rubicundus
GATACTTTATTTTATCTTTGCTATATGATCAGGAATAATCGGAGCATCTTTAAGTATAAT
TATTCGTATAGAATTAAGAACACCTGGAAGATGAATTAATAATGATCAAATTTATAATAC
TATTGTAACTTCACATGCTTTTATTATAATTTTTTTTATAGTTATACCATTTATAATTGG
AGGATTTGGAAATTGATTAGTACCACTAATAATTGGAGCTCCTGATATAGCTTTCCCACG
TATAAATAATATAAGATTTTGATTATTAATTCCTTCATTATTTATACTTATAATAAGAAG
AACTTTATCTACAGGATCAGGAACAGGTTGAACTATTTACCCTCCCCTATCTTCAATTAT
ATATCACTCATCTTTTTCTGTTGATTTTACTATTTTTTCTTTACATATCGCAGGTATTTC
TTCTATTATAGGTGCTATTAATTTTATTGTTTCAATTATATTAATAAAAAATATTTCACT
TAATATAAATCAAATCCCCTTATTTCCTTGATCAGTTAAAATTACTGCTATTTTACTTCT
TCTTTCTCTCCCTGTTTTAGCAGGTGCTATTACTATACTATTAACAGACCGAAATTTAAA
TACATCTTTTTTTGACCCTTC
>MF901805;tax=d:Eukarya,p:Arthropoda,c:Insecta,o:Hymenoptera,f:Halictidae,g:Halictus,s:Halictus rubicundus
ATACTTTATTTTATCTTTGCTATATGATCAGGAATAATCGGAGCATCTTTAAGTATAATT
ATTCGTATAGAATTAAGAACACCTGGAAGATGAATTAATAATGATCAAATTTATAATACT
ATTGTAACTTCACATGCTTTTATTATAATTTTTTTTATAGTTATACCATTTATAATTGGA
GGATTTGGAAATTGATTAGTACCACTAATAATTGGAGCTCCTGATATAGCTTTCCCACGT
ATAAATAATATAAGATTTTGATTATTAATTCCTTCATTATTTATACTTATAATAAGAAGA
ACTTTATCTACAGGATCAGGAACAGGTTGAACTATTTACCCTCCCCTATCTTCAATTATA
TATCACTCATCTTTTTCTGTTGATTTTACTATTTTTTCTTTACATATCGCAGGTATTTCT
TCTATTATAGGTGCTATTAATTTTATTGTTTCAATTATATTAATAAAAAATATTTCACTT
AATATAAATCAAATCCCCTTATTTCCTTGATCAGTTAAAATTACTGCTATTTTACTTCTT
CTTTCTCTCCCTGTTTTAGCAGGTGCTATTACTATACTATTAACAGACCGA
>MF908230;tax=d:Eukarya,p:Arthropoda,c:Insecta,o:Hymenoptera,f:Halictidae,g:Halictus,s:Halictus rubicundus
ATACTTTATTTTATCTTTGCTATATGATCGGGAATAATCGGGGCATCTTTAAGTATAATT
ATTCGTATAGAATTAAGAACACCTGGAAGATGAATTAATAACGACCAAATTTATAATACT
ATTGTAACTTCACATGCTTTTATTATAATTTTTTTTATAGTTATACCATTTATAATTGGA
GGGTTTGGAAATTGATTAGTACCACTAATAATTGGAGCTCCTGATATAGCTTTCCCACGT
ATAAATAATATAAGATTTTGATTATTAATTCCTTCATTATTTATACTTATAATAAGAAGA
ACTTTATCTACAGGATCAGGAACAGGTTGAACTATTTACCCTCCCCTATCTTCAATTATA
TATCACTCATCCTTTTCTGTTGATTTTACTATTTTTTCTTTACATATCGCAGGTATTTCT
TCTATTATAGGTGCTATTAATTTTATTGTTTCAATTATATTAATAAAAAATATTTCACTT
AATATAAATCAAATCCCCTTATTTCCTTGATCAGTTAAAATTACTGCTATTTTACTTCTT
CTTTCTCTCCCCGTTTTAGCAGGTGCTATTACTATATTATTAACAGAC
>MG337606;tax=d:Eukarya,p:Arthropoda,c:Insecta,o:Hymenoptera,f:Halictidae,g:Halictus,s:Halictus rubicundus
CTTTATTTTATCTTTGCTATATGATCAGGAATAATCGGAGCATCTTTAAGTATAATTATT
CGTATAGAATTAAGAACACCTGGAAGATGAATTAATAACGACCAAATTTATAATACTATT
GTAACTTCACATGCTTTTATTATAATTTTTTTTATAGTTATACCATTTATAATTGGAGGG
TTTGGAAATTGATTAGTACCACTAATAATTGGAGCTCCTGATATAGCTTTCCCACGTATA
AATAATATAAGATTTTGATTATTAATTCCTTCATTATTTATACTTATAATAAGAAGAACT
TTATCTACAGGATCAGGAACAGGTTGAACTATTTACCCTCCCCTATCTTCAATTATATAT
CACTCATCTTTTTCTGTTGATTTTACTATTTTTTCTTTACATATCGCAGGTATTTCTTCT
ATTATAGGTGCTATTAATTTTATTGTTTCAATTATATTAATAAAAAATATTTCACTTAAT
ATAAATCAAATCCCCTTATTTCCTTGATCAGTTAAAATTACTGCTATTTTACTTCTTCTT

TCTCTCCCCGTTTTAGCAGGTGCTATTACTATACTATTAACAGACCGAAATTTAAATACA
TCTTTTTTTGACCCTTCTGGAGGAGGAGACCCTATTCTTTACCAACATTTA
>MG338776;tax=d:Eukarya,p:Arthropoda,c:Insecta,o:Hymenoptera,f:Halictidae,g:Halictus,s:Halictus rubicundus
CTTTATTTTATCTTTGCTATATGATCAGGAATAATCGGAGCATCTTTAAGTATAATTATT
CGTATAGAATTAAGAACACCTGGAAGATGAATTAATAATGACCAAATTTATAATACTATT
GTAACTTCACATGCTTTTATTATAATTTTTTTTATAGTTATACCATTTATAATTGGAGGG
TTTGGAAATTGATTAGTACCACTAATAATTGGAGCTCCTGATATAGCTTTCCCACGTATA
AATAATATAAGATTTTGATTATTAATTCCTTCATTATTTATACTTATAATAAGAAGAACT
TTATCTACAGGATCAGGAACAGGTTGAACTATTTACCCTCCCCTATCTTCAATTATATAT
CACTCATCTTTTTCTGTTGATTTTACTATTTTTTCTTTACATATCGCAGGTATTTCTTCT
ATTATAGGTGCTATTAATTTTATTGTTTCAATTATATTAATAAAAAATATTTCACTTAAT
ATAAATCAAATCCCCTTATTTCCTTGATCAGTTAAAATTACTGCTATTTTACTTCTTCTT
TCTCTCCCTGTTTTAGCAGGTGCTATTACTATACTATTAACAGACCGAAATTTAAATACA
TCTTTTTTTGACCCTTCTGGAGGAGGAGACCCTATTCTTTACCAACATTTA
>MG340325;tax=d:Eukarya,p:Arthropoda,c:Insecta,o:Hymenoptera,f:Halictidae,g:Halictus,s:Halictus rubicundus
CTTTATTTTATCTTTGCTATATGATCAGGAATAATCGGAGCATCTTTAAGTATAATTATT
CGTATAGAATTAAGAACACCTGGAAGATGAATTAATAATGACCAAATTTATAATACTATT
GTAACTTCACATGCTTTTATTATAATTTTTTTTATAGTTATACCATTTATAATTGGAGGA
TTTGGAAATTGATTAGTACCACTAATAATTGGAGCTCCTGATATAGCTTTCCCACGTATA
AATAATATAAGATTTTGATTATTAATTCCTTCATTATTTATACTTATAATAAGAAGAACT
TTATCTACAGGATCAGGAACAGGTTGAACTATTTACCCYCCCCTATCTTCAATTATATAT
CACTCATCTTTTTCTGTTGATTTTACTATTTTTTCTTTACATATCGCAGGTATTTCTTCT
ATTATAGGTGCTATTAATTTTATTGTTTCAATTATATTAATAAAAAATATTTCACTTAAT
ATAAATCAAATCCCCTTATTTCCTTGATCAGTTAAAATTACTGCTATTTTACTTCTTCTT
TCTCTCCCTGTTTTAGCAGGTGCTATTACTATACTATTAACAGACCGAAATTTAAATACA
TCTTTTTTTGACCCTTCTGGAGGAGGAGACCCTATTCTTTATCAACATTTA
>MG343222;tax=d:Eukarya,p:Arthropoda,c:Insecta,o:Hymenoptera,f:Halictidae,g:Halictus,s:Halictus rubicundus
CTTTATTTTATCTTTGCTATATGATCCGGAATAATCGGAGCATCTTTAAGTATAATTATT
CGTATAGAATTAAGAACACCTGGAAGATGAATTAATAACGACCAAATTTATAATACTATT
GTAACTTCACATGCTTTTATTATAATTTTTTTTATAGTTATACCATTTATAATTGGAGGA
TTTGGAAATTGATTAGTACCACTAATAATTGGAGCTCCTGATATAGCTTTCCCACGTATA
AATAATATAAGATTTTGATTATTAATTCCTTCATTATTTATACTTATAATAAGAAGAACT
TTATCTACAGGATCAGGAACAGGTTGAACTATTTACCCTCCCCTATCTTCAATTATATAT
CACTCATCTTTTTCTGTTGATTTTACTATTTTTTCTTTACATATCGCAGGTATTTCTTCT
ATTATAGGTGCTATTAATTTTATTGTTTCAATTATATTAATAAAAAATATTTCACTTAAT
ATAAATCAAATCCCCTTATTTCCTTGATCAGTTAAAATTACTGCTATTTTACTTCTTCTT
TCTCTCCCTGTTTTAGCAGGTGCTATTACTATACTATTAACAGACCGAAATTTAAATACA
TCTTTTTTTGACCCTTCTGGAGGAGGAGACCCTATTCTTTACCAACATTTA
>MG344543;tax=d:Eukarya,p:Arthropoda,c:Insecta,o:Hymenoptera,f:Halictidae,g:Halictus,s:Halictus rubicundus
CTTTATTTTATCTTTGCTATATGATCGGGAATAATCGGAGCATCTTTAAGTATAATTATT
CGTATAGAATTAAGAACACCTGGAAGATGAATTAATAACGACCAAATTTATAATACTATT
GTAACTTCACATGCTTTTATTATAATTTTTTTTATAGTTATACCATTTATAATTGGAGGA
TTTGGAAATTGATTAGTACCACTAATAATTGGAGCTCCTGATATAGCTTTCCCACGTATA
AATAATATAAGATTTTGATTATTAATTCCTTCATTATTTATACTTATAATAAGAAGAACT
TTATCTACAGGATCAGGAACAGGTTGAACTATTTACCCTCCCCTATCTTCAATTATATAT
CACTCATCCTTTTCTGTTGATTTTACTATTTTTTCTTTACATATCGCAGGTATTTCTTCT
ATTATAGGTGCTATTAATTTTATTGTTTCAATTATATTAATAAAAAATATTTCACTTAAT
ATAAATCAAATCCCCTTATTTCCTTGATCAGTTAAAATTACTGCTATTTTACTTCTTCTT
TCTCTCCCCGTTTTAGCAGGTGCTATTACTATATTATTAACAGACCGAAATTTAAATACA
TCTTTTTTTGACCCTTCTGGGGGAGGAGACCC

>HQ558103;tax=d:Eukarya,p:Arthropoda,c:Insecta,o:Hymenoptera,f:Halictidae,g:Lasioglossum,s:Lasioglossum cressonii
AATACTTTATTTTATTTTTGCTATATGATCTGGAATAATTGGAGCTTCATTAAGGATAAT
TATTCGAATAGAATTAAGTGCCCCAGGAAAATGAATTAATAATGATCAAATTTATAATAC
TATTATTACTTCACATGCATTTGTAATAATTTTTTTTATAGTTATACCATTTATAATTGG
AGGATTTGGAAATTGATTAGTCCCTTTAATAATTGGAGCCCCTGATATAGCTTTCCCTCG
AATAAACAATATAAGATTTTGATTACTTATCCCATCATTATATATATTATTAATAAGAAG
AATCGTAGCCTCTGGGTCAGGGACTGGATGAACTGTGTACCCCCCCTTATCATCAATTAT
ATACCATTCATCAATTTCAGTAGATTATACTATCTTTTCATTACACATTGCAGGAATTTC
ATCTATTATAGGAGCAATCAACTTTATTGTATCTATTTTACTTATAAAAAATATTTCAAT
TAATTATGATCAAATCCCTTTATTTCCATGATCAGTGAAAATTACTGCCATTCTATTATT
ATTATCTTTACCAATTTTAGCAGGAGCTATTACTATACTTTTAACAGATCGAAATTTAAA
TACATCATTTTTTGACCCCTCGGGGGGAGGAGACCCTATTCTTTATCAACATTTATTT
>JF864829;tax=d:Eukarya,p:Arthropoda,c:Insecta,o:Hymenoptera,f:Halictidae,g:Lasioglossum,s:Lasioglossum cressonii
AATACTTTATTTTATTTTTGCTATATGATCTGGAATAATTGGAGCTTCATTAAGAATAAT
TATTCGAATAGAATTAAGTGCCCCAGGAAAATGAATTAATAACGATCAAATTTATAATAC
TATTATTACTTCACATGCATTTGTAATAATTTTTTTTATAGTTATACCATTTATAATTGG
AGGATTTGGAAATTGATTAATCCCCTTAATAATTGGAGCCCCTGATATAGCTTTCCCTCG
AATAAACAATATAAGATTTTGGTTACTTATTCCATCAATATTTATATTATTAATAAGAAG
AATTATAGCTTCCGGATCAGGAACTGGATGAACTGTATACCCCCCTTTATCATCAATTAT
ATACCATTCATCAATTTCAGTAGATTATACTATCTTTTCATTACACATTGCAGGAATTTC
ATCTATTATAGGAGCAATCAACTTTATTGTATCTATTTTACTTATAAAAAATATTTCAAT
TAATTATGATCAAATCCCTTTATTCCCATGATCAGTAAAAATTACTGCCATTCTATTATT
ATTATCTTTACCAGTTTTAGCAGGAGCTATTACTATACTTTTAACAGATCGAAACTTAAA
TACATCATTTTTTGACCCCTCAGGAGGAGGAGATCCTATTCTTTATCAACATCTA
>JF903506;tax=d:Eukarya,p:Arthropoda,c:Insecta,o:Hymenoptera,f:Halictidae,g:Lasioglossum,s:Lasioglossum cressonii
AATACTTTATTTTATTTTTGCTATATGATCTGGAATAATTGGAGCTTCATTAAGAATAAT
TATTCGAATAGAATTAAGTGCCCCAGGAAAATGAATTAATAACGATCAAATTTATAATAC
TATTATTACTTCACATGCATTTGTAATAATTTTTTTTATAGTTATACCATTTATAATTGG
AGGATTTGGAAATTGATTAATCCCCTTAATAATTGGAGCCCCTGATATAGCTTTCCCTCG
AATAAACAATATAAGATTTTGGTTACTTATTCCATCAATATTTATATTATTAATAAGAAG
AATTATAGCTTCCGGATCAGGAACTGGATGAACTGTATACCCCCCTTTATCATCAATTAT
ATACCACTCATCAATTTCAGTAGATTATACTATCTTTTCATTACACATTGCAGGAATTTC
ATCTATTATAGGAGCAATCAACTTTATTGTATCTATTTTACTTATAAAAAATATTTCAAT
TAATTATGATCAAATCCCTTTATTCCCATGATCAGTAAAAATTACTGCCATTCTATTATT
ATTATCTTTACCAGTTTTAGCAGGAGCTATTACTATACTTTTAACAGATCGAAACTTAAA
TACATCATTTTTTGACCCCTCAGGAGGAGGAGATCCTATTCTTTATCAACATCTA
>JN288460;tax=d:Eukarya,p:Arthropoda,c:Insecta,o:Hymenoptera,f:Halictidae,g:Lasioglossum,s:Lasioglossum cressonii
AATACTTTATTTTATTTTTGCTATATGATCTGGAATAATTGGAGCTTCATTAAGAATAAT
TATTCGAATAGAATTAAGTGCCCCAGGAAAATGAATTAATAACGATCAAATTTATAATAC
TATTATTACTTCACATGCATTTGTAATAATTTTTTTTATAGTTATACCATTTATAATTGG
AGGATTTGGAAATTGATTAATCCCCTTAATAATTGGAGCCCCTGATATAGCTTTCCCTCG
AATAAACAATATAAGATTTTGGTTACTTATTCCATCAATATTTATATTATTAATAAGAAG
AATTATAGCTTCCGGATCAGGAACTGGATGAACTGTATACCCCCCTTTATCATCAATTAT
ATACCATTCATCAATTTCAGTAGATTATACTATCTTTTCATTGCACATTGCAGGAATTTC
ATCTATTATAGGAGCAATCAACTTTATTGTATCTATTTTACTTATAAAAAATATTTCAAT
TAATTATGATCAAATCCCTTTATTCCCATGATCAGTAAAAATTACTGCCATTCTATTATT
ATTATCTTTACCAGTTTTAGCAGGAGCTATTACTATACTTTTAACAGATCGAAACTTAAA

TACATCATTTTTTGACCCCTCAGGAGGAGGAGATCCTATTCTTTATCAACATCTATTT
>KR421688;tax=d:Eukarya,p:Arthropoda,c:Insecta,o:Hymenoptera,f:Halictidae,g:Lasioglossum,s:Lasioglossum cressonii
AATACTTTATTTTATTTTTGCTATATGATCTGGAATAATTGGAGCTTCATTAAGAATAAT
TATTCGAATAGAATTAAGTGCCCCAGGAAAATGAATTAATAACGATCAAATTTATAATAC
TATTATTACTTCACATGCATTTGTAATAATTTTTTTTATAGTTATACCATTTATAATTGG
AGGATTTGGAAATTGATTAATCCCCTTAATAATTGGAGCCCCTGATATAGCTTTCCCTCG
AATAAACAATATAAGATTTTGGTTACTTATTCCATCAATATTTATATTATTAATAAGAAG
AATTATAGCTTCCGGATCAGGAACTGGATGAACTGTATACCCCCCTTTATCATCAATTAT
ATACCATTCATCAATTTCAGTAGATTATACTATCTTTTCATTACACATTGCAGGAATTTC
ATCTATTATAGGAGCAATCAACTTTATTGTATCTATTTTACTTATAAAAAATATTTCAAT
TAATTATGATCAAATCCCTTTATTCCCATGATCAGTAAAAATTACTGCCATTCTATTATT
ATTATCTTTACCAGTTTTAGCAGGAGCTATTACTATACTTTTAACAGATCGAAACTTAAA
TACATCATTTTT
>KR795244;tax=d:Eukarya,p:Arthropoda,c:Insecta,o:Hymenoptera,f:Halictidae,g:Lasioglossum,s:Lasioglossum cressonii
AATACTTTATTTTATTTTTGCTATATGATCTGGAATAATTGGAGCTTCATTAAGAATAAT
TATTCGAATAGAATTAAGTGCCCCAGGAAAATGAATTAATAACGATCAAATTTATAATAC
TATTATTACTTCACATGCATTTGTAATAATTTTTTTTATAGTTATACCATTTATAATTGG
AGGATTTGGAAATTGATTAATCCCCTTAATAATTGGAGCCCCTGATATAGCTTTCCCTCG
AATAAACAATATAAGATTTTGGTTACTTATTCCATCAATATTTATATTATTAATAAGAAG
AATTATAGCTTCCGGATCAGGAACTGGATGAACTGTATACCCCCCTTTATCATCAATTAT
ATACCATTCATCAATTTCAGTAGATTATACTATCTTTTCATTACACATTGCAGGAATTTC
ATCTATTATAGGAGCAATCAACTTTATTGTATCTATTTTACTTATAAAAAATATTTCAAT
TAATTATGATCAAATCCCTTTATTCCCATGATCAGTAAAAATTACTGCCATTCTATTATT
ATTATCTTTACCAGTTTTAGCAGGAGCTATTACTATACTTTTAACAGATCGAAACTTAAA
TACATCATTTTTTGACCCCTCAGGAGGAGGAGATCCTATTCTTTATCAACATCTATTT
>KR797529;tax=d:Eukarya,p:Arthropoda,c:Insecta,o:Hymenoptera,f:Halictidae,g:Lasioglossum,s:Lasioglossum cressonii
AATACTTTATTTTATTTTTGCTATATGATCTGGGATAATTGGAGCTTCATTAAGAATAAT
TATTCGAATAGAATTAAGTGCCCCAGGAAAATGAATTAATAACGATCAAATTTATAATAC
TATTATTACTTCACATGCATTTGTAATAATTTTTTTTATAGTTATACCATTTATAATTGG
AGGATTTGGAAATTGATTAATCCCCTTAATAATTGGAGCCCCTGATATAGCTTTCCCTCG
AATAAACAATATAAGATTTTGATTACTTATTCCATCAATATTTATATTATTAATAAGAAG
AATTATAGCTTCCGGATCAGGAACTGGATGAACTGTATACCCCCCTTTATCATCAATTAT
ATACCATTCATCAATTTCAGTAGATTATACTATCTTTTCATTACACATTGCAGGAATTTC
ATCTATTATAGGAGCAATCAACTTTATTGTATCTATTTTACTTATAAAAAATATTTCAAT
TAATTATGATCAAATCCCTTTATTCCCATGATCAGTAAAAATTACTGCCATTCTATTATT
ATTATCTTTACCAGTTTTAGCAGGAGCTATTACTATACT
>KR797623;tax=d:Eukarya,p:Arthropoda,c:Insecta,o:Hymenoptera,f:Halictidae,g:Lasioglossum,s:Lasioglossum cressonii
AATACTTTATTTTATTTTTGCTATATGATCTGGGATAATTGGAGCTTCATTAAGAATAAT
TATTCGAATAGAATTAAGTGCCCCAGGAAAATGAATTAATAACGATCAAATTTATAATAC
TATTATTACTTCACATGCATTTGTAATAATTTTTTTTATAGTTATACCATTTATAATTGG
AGGATTTGGAAATTGATTAATCCCCTTAATAATTGGAGCCCCTGATATAGCTTTCCCTCG
AATAAACAATATAAGATTTTGATTACTTATTCCATCAATATTTATATTATTAATAAGAAG
AATTATAGCTTCCGGATCAGGAACTGGATGAACTGTATACCCCCCTTTATCATCAATTAT
ATACCATTCATCAATTTCAGTAGATTATACTATCTTTTCATTACACATTGCAGGAATTTC
ATCTATTATAGGAGCAATCAACTTTATTGTATCTATTTTACTTATAAAAAATATTTCAAT
TAATTATGATCAAATCCCTTTATTCCCATGATCAGTAAAAATTACTGCCATTCTATTATT
ATTATCTTTACCAGTTTTAGCAGGAGCTATTACTATACTTTTAACAGATCGAAACTTAAA

TACATCATTTTTTGACCCCTCAGGAGGAGGAGATCCTATTCTTTATCAACATCTATTT
>KR804973;tax=d:Eukarya,p:Arthropoda,c:Insecta,o:Hymenoptera,f:Halictidae,g:Lasioglossum,s:Lasioglossum cressonii
AATACTTTATTTTATTTTTGCTATATGATCTGGGATAATTGGAGCTTCATTAAGAATAAT
TATTCGAATAGAATTAAGTGCCCCAGGAAAATGAATTAATAACGATCAAATTTATAATAC
TATTATTACTTCACATGCATTTGTAATAATTTTTTTTATAGTTATACCATTTATAATTGG
AGGATTTGGAAATTGATTAATCCCCTTAATAATTGGAGCCCCTGATATAGCTTTCCCTCG
AATAAACAATATAAGATTTTGATTACTTATTCCATCAATATTTATATTATTAATAAGAAG
AATTATAGCTTCCGGATCAGGAACTGGATGAACTGTATACCCCCCTTTATCATCAATTAT
ATACCATTCATCAATTTCAGTAGATTATACTATCTTTTCATTACACATTGCAGGAATTTC
ATCTATTATAGGAGCAATCAACTTTATTGTATCTATTTTACTTATAAAAAATATTTCAAT
TAATTATGATCAAATCCCTTTATTCCCATGATCAGTAAAAATTACTGCCATTCTATTATT
ATTATCTTTACCAGTTTTAGCAGGAGCTATTACTATACTTTTAACAGATCGAAACTTAAA
TACATCATTTTTTGACCCCTCAGGAGGAGGAGATCC
>KR808410;tax=d:Eukarya,p:Arthropoda,c:Insecta,o:Hymenoptera,f:Halictidae,g:Lasioglossum,s:Lasioglossum cressonii
AATACTTTATTTTATTTTTGCTATATGATCTGGAATAATTGGAGCTTCATTAAGAATAAT
TATTCGAATAGAATTAAGTGCCCCAGGAAAATGAATTAATAACGATCAAATTTATAATAC
TATTATTACTTCACATGCATTTGTAATAATTTTTTTTATAGTTATACCATTTATAATTGG
AGGATTTGGAAATTGATTAATCCCCTTAATAATTGGAGCCCCTGATATAGCTTTCCCTCG
AATAAACAATATAAGATTTTGGTTACTTATTCCATCAATATTTATATTATTAATAAGAAG
AATTATAGCTTCCGGATCAGGAACTGGATGAACTGTATACCCCCCTTTATCATCAATTAT
ATACCATTCATCAATTTCAGTAGATTATACTATCTTTTCATTACACATTGCAGGAATTTC
ATCTATTATAGGAGCAATCAACTTTATTGTATCTATTTTACTTATAAAAAATATTTCAAT
TAATTATGATCAAATCCCTTTATTCCCATGATCAGTAAAAATTACTGCCATTCTATTATT
ATTATCTTTACCAGTTTTAGCAGGAGCTATTACTATACTTTTAA
>KR875736;tax=d:Eukarya,p:Arthropoda,c:Insecta,o:Hymenoptera,f:Halictidae,g:Lasioglossum,s:Lasioglossum cressonii
AATACTTTATTTTATTTTTGCTATATGATCTGGGATAATTGGAGCTTCATTAAGAATAAT
TATTCGAATAGAATTAAGTGCCCCAGGAAAATGAATTAATAACGATCAAATTTATAATAC
TATTATTACTTCACATGCATTTGTAATAATTTTTTTTATAGTTATACCATTTATAATTGG
AGGATTTGGAAATTGATTAATCCCCTTAATAATTGGAGCCCCTGATATAGCTTTCCCTCG
AATAAACAATATAAGATTTTGATTACTTATTCCATCAATATTTATATTATTAATAAGAAG
AATTATAGCTTCCGGATCAGGAACTGGATGAACTGTATACCCCCCTTTATCATCAATTAT
ATACCATTCATCAATTTCAGTAGATTATACTATCTTTTCATTACACATTGCAGGAATTTC
ATCTATTATAGGAGCAATCAACTTTATTGTATCTATTTTACTTATAAAAAATATTTCAAT
TAATTATGATCAAATCCCTTTATTCCCATGATCAGTAAAAATTACTGCCATTCTATTATT
ATTATCTTTACCAGTTTTAGCAGGAGCTATTACTATACTTTTAACAGATCGAAACTTAAA
TACATCATTTTTTGACCCCTCAGGAG
>KR877130;tax=d:Eukarya,p:Arthropoda,c:Insecta,o:Hymenoptera,f:Halictidae,g:Lasioglossum,s:Lasioglossum cressonii
AATACTTTATTTTATTTTTGCTATATGATCTGGAATAATTGGAGCTTCATTAAGAATAAT
TATTCGAATAGAATTAAGTGCCCCAGGAAAATGAATTAATAACGATCAAATTTATAATAC
TATTATTACTTCACATGCATTTGTAATAATTTTTTTTATAGTTATACCATTTATAATTGG
AGGATTTGGAAATTGATTAATCCCCTTAATAATTGGAGCCCCTGATATAGCTTTCCCTCG
AATAAACAATATAAGATTTTGGTTACTTATTCCATCAATATTTATATTATTAATAAGAAG
AATTATAGCTTCAGGATCAGGAACTGGATGAACTGTATACCCCCCTTTATCATCAATTAT
ATACCATTCATCAATTTCAGTAGATTATACTATCTTTTCATTACACATTGCAGGAATTTC
ATCTATTATAGGAGCAATCAACTTTATTGTATCTATTTTACTTATAAAAAATATTTCAAT
TAATTATGATCAAATCCCTTTATTCCCATGATCAGTAAAAATTACTGCCATTCTATTATT
ATTATCTTTACCAGTTTTAGCAGGAGCTATTACTATA

>KR879460;tax=d:Eukarya,p:Arthropoda,c:Insecta,o:Hymenoptera,f:Halictidae,g:Lasioglossum,s:Lasioglossum cressonii
AATACTTTATTTTATTTTTGCTATATGATCTGGAATAATTGGAGCCTCATTAAGAATAAT
TATTCGAATAGAATTAAGCGCCCCAGGAAAATGAATTAATAATGATCAAATTTATAACAC
TATTATTACTTCACATGCATTTGTAATAATTTTTTTTATAGTTATACCATTTATAATTGG
GGGATTTGGGAATTGATTAGTTCCTTTAATAATTGGAGCCCCTGATATAGCTTTCCCCCG
AATAAACAATATAAGATTTTGATTACTTATCCCATCAATATTTATATTATTAATAAGAAG
AATTATAGCTTCCGGATCAGGAACTGGGTGAACTGTATACCCCCCTTTATCATCAATTAT
ATACCATTCATCAATTTCAGTAGATTACACTATCTTTTCATTACACATTGCAGGAATCTC
ATCTATTATAGGAGCAATTAATTTTATTGTATCTATTTTACTTATAAAAAATATTTCAAT
TAATTATGACCAAATCCCTTTATTCCCATGATCAGTAAAAATTACTGCCATTCTATTATT
ATTATCTTTACCAGTTTTAGCAGGAGCTATCACTATACTTTTAACAGACCGAAATTTAAA
CACATCATTTTTTGACCCCTCAGGAGGAGGAGATCCTATTCTTTATCAACATCTATTT
>KR885567;tax=d:Eukarya,p:Arthropoda,c:Insecta,o:Hymenoptera,f:Halictidae,g:Lasioglossum,s:Lasioglossum cressonii
ATACTTTATTTTATTTTTGCTATATGATCTGGAATAATTGGAGCTTCATTAAGAATAATT
ATTCGAATAGAATTAAGTGCCCCAGGAAAATGAATTAATAATGATCAAATTTATAATACT
ATTATTACTTCACATGCATTTGTAATAATTTTTTTTATAGTTATACCATTTATAATTGGA
GGATTTGGAAATTGATTAATCCCCTTAATAATTGGAGCCCCTGATATAGCTTTCCCTCGA
ATAAACAATATAAGATTTTGGTTACTTATTCCATCAATATTTATATTATTAATAAGAAGA
ATTATAGCTTCCGGATCAGGAACTGGATGAACTGTATACCCCCCTTTATCATCAATTATA
TACCATTCATCAATTTCAGTAGATTATACTATCTTTTCATTACACATTGCAGGAATTTCA
TCTATTATAGGAGCAATCAACTTTATTGTATCTATTTTACTTATAAAAAATATTTCAATT
AATTATGATCAAATCCCTTTATTCCCATGATCAGTAAAAATTACTGCCATTCTATTATTA
TTATCTTTACCAGTTTTAGCAGGAGCTATTACTATACTTTTAACAGATCGAAACTTAAAT
ACA
>MF899206;tax=d:Eukarya,p:Arthropoda,c:Insecta,o:Hymenoptera,f:Halictidae,g:Lasioglossum,s:Lasioglossum cressonii
ATACTTTATTTTATTTTTGCTATATGATCTGGAATAATTGGAGCTTCATTAAGAATAATT
ATTCGAATAGAATTAAGTGCCCCAGGAAAATGAATTAATAATGATCAAATTTATAATACT
ATTATTACTTCACATGCATTTGTAATAATTTTTTTTATAGTTATACCATTTATAATTGGA
GGATTTGGAAATTGATTAATCCCCTTAATAATTGGAGCCCCTGATATAGCTTTCCCTCGA
ATAAACAATATAAGATTTTGGTTACTTATTCCATCAATATTTATATTATTAATAAGAAGA
ATTATAGCTTCCGGATCAGGAACTGGATGAACTGTATACCCCCCTTTATCATCAATTATA
TACCATTCATCAATTTCAGTAGATTATACTATCTTTTCATTACACATTGCAGGAATTTCA
TCTATTATAGGAGCAATCAACTTTATTGTATCTATTTTACTTATAAAAAATATTTCAATT
AATTATGATCAAATCCCTTTATTCCCATGATCAGTAAAAATTACTGCCATTCTATTATTA
TTATCTTTACCAGTTTTAGCAGGAGCTATTACTATA
>MF903562;tax=d:Eukarya,p:Arthropoda,c:Insecta,o:Hymenoptera,f:Halictidae,g:Lasioglossum,s:Lasioglossum cressonii
ATACTTTATTTTATTTTTGCTATATGATCTGGAATAATTGGAGCTTCATTAAGAATAATT
ATTCGAATAGAATTAAGTGCCCCAGGAAAATGAATTAATAATGATCAAATTTATAATACT
ATTATTACTTCACATGCATTTGTAATAATTTTTTTTATAGTTATACCATTTATAATTGGA
GGATTTGGAAATTGATTAATCCCCTTAATAATTGGAGCCCCTGATATAGCTTTCCCTCGA
ATAAACAATATAAGATTTTGGTTACTTATTCCATCAATATTTATATTATTAATAAGAAGA
ATTATAGCTTCCGGATCAGGAACTGGATGAACTGTATACCCCCCTTTATCATCAATTATA
TACCATTCATCAATTTCAGTAGATTATACTATCTTTTCATTACACATTGCAGGAATTTCA
TCTATTATAGGAGCAATCAACTTTATTGTATCTATTTTACTTATAAAAAATATTTCAATT
AATTATGATCAAATCCCTTTATTCCCATGATCAGTAAAAATTACTGCCATTCTATTATTA
TTATCTTTACCAGTTTTAGCAGGA

>MF906588;tax=d:Eukarya,p:Arthropoda,c:Insecta,o:Hymenoptera,f:Halictidae,g:Lasioglossum,s:Lasioglossum cressonii
ATACTTTATTTTATTTTTGCTATATGATCTGGAATAATTGGAGCTTCATTAAGAATAATT
ATTCGAATAGAATTAAGTGCCCCAGGAAAATGAATTAATAACGATCAAATTTATAATACT
ATTATTACTTCACATGCATTTGTAATAATTTTTTTTATAGTTATACCATTTATAATTGGA
GGATTTGGAAATTGATTAATCCCCTTAATAATTGGAGCCCCTGATATAGCTTTCCCTCGA
ATAAACAATATAAGATTTTGATTACTTATTCCATCAATATTTATATTATTAATAAGAAGA
ATTATAGCTTCCGGATCAGGAACTGGATGAACTGTATACCCCCCTTTATCATCAATTATA
TACCATTCATCAATTTCAGTAGATTATACTATCTTTTCATTACACATTGCAGGAATTTCA
TCTATTATAGGAGCAATCAACTTTATTGTATCTATTTTACTTATAAAAAATATTTCAATT
AATTATGATCAAATCCCTTTATTCCCATGATCAGTAAAAATTACTGCCATTCTATTATTA
TTATCTTTACCAGTTTTAGCAGGAGCTATTACTATACTTTTAACAGAT
>MG338703;tax=d:Eukarya,p:Arthropoda,c:Insecta,o:Hymenoptera,f:Halictidae,g:Lasioglossum,s:Lasioglossum cressonii
ATACTTTATTTTATTTTTGCTATATGATCTGGAATAATTGGAGCCTCATTAAGAATAATT
ATTCGAATAGAATTAAGCGCCCCAGGAAAATGAATTAATAATGATCAAATTTATAACACT
ATTATTACTTCACATGCATTTGTAATAATTTTTTTTATAGTTATACCATTTATAATTGGG
GGATTTGGAAATTGATTAGTTCCTTTAATAATTGGAGCCCCTGATATAGCTTTCCCCCGA
ATAAATAATATAAGATTTTGATTACTTATTCCATCAATATTTATATTATTAATAAGAAGA
ATTATAGCTTCCGGATCAGGAACTGGGTGAACTGTATACCCTCCTTTATCATCAATTATA
TACCATTCATCAATTTCAGTAGATTACACTATCTTTTCATTACACATTGCAGGAATCTCA
TCTATTATAGGAGCAATTAATTTTATTGTATCTATTTTACTTATAAAAAATATTTCAATT
AATTATGATCAAATCCCTTTATTCCCATGATCAGTAAAAATTACTGCCATTCTATTATTA
TTATCTTTACCAGTTTTAGCAGGAGCTATCACTATA
>MG343397;tax=d:Eukarya,p:Arthropoda,c:Insecta,o:Hymenoptera,f:Halictidae,g:Lasioglossum,s:Lasioglossum cressonii
AATACTTTATTTTATTTTTGCTATATGATCTGGAATAATTGGAGCCTCATTAAGAATAAT
TATTCGAATAGAATTAAGCGCCCCAGGAAAATGAATTAATAATGATCAAATTTATAACAC
TATTATTACTTCACATGCATTTGTAATAATTTTTTTTATAGTTATACCATTTATAATTGG
GGGATTTGGAAATTGATTAGTTCCTTTAATAATTGGAGCCCCTGATATAGCTTTCCCCCG
AATAAATAATATAAGATTTTGATTACTTATTCCATCAATATTTATATTATTAATAAGAAG
AATTATAGCTTCCGGATCAGGAACTGGGTGAACTGTATACCCTCCTTTATCATCAATTAT
ATACCATTCATCAATTTCAGTAGATTACACTATCTTTTCATTACACATTGCAGGAATCTC
ATCTATTATAGGAGCAATTAATTTTATTGTATCTATTTTACTTATAAAAAATATTTCAAT
TAATTATGACCAAATCCCTTTATTCCCATGATCAGTAAAAATTACTGCCATTCTATTATT
ATTATCTTTACCAGTTTTAGCAGGAGCTATCACTATACTTTTAACAGACCGAAATTTAAA
CACATCATTTTTTGACCCCTCAGGAGGGGGAGATCCTATTCTTTATCAACATCTATTT
>JF903521;tax=d:Eukarya,p:Arthropoda,c:Insecta,o:Hymenoptera,f:Halictidae,g:Lasioglossum,s:Lasioglossum imitatum
AATACTTTATTTTATTTTTGCTATATGATCTGGAATAATTGGAGCTTCATTAAGAATAAT
TATTCGAATAGAACTAAGTGCACCAGGAAAATGAATTAATAACGATCAAATTTATAATAC
TATTATTACTTCTCATGCATTCGTAATAATTTTTTTTATAGTTATACCATTTATAATTGG
GGGATTCGGTAACTGATTAGTCCCTTTAATAATTGGTGCTCCCGATATAGCATTCCCTCG
AATAAATAATATAAGATTTTGATTACTTATTCCTTCAATATTTATATTATTAATAAGAAG
TATTATATCTTCTGGATCAGGGACTGGATGAACTGTATACCCCCCCTTATCTTCAATTAT
ATACCACTCATCAATTTCAGTAGATTATACTATTTTTTCATTACATATTGCAGGAATTTC
ATCTATTATAGGTGCCATCAATTTTATTGTATCTATTATACTTATAAAAAATATTTCAAT
TAATTATGATCAAATTCCTTTATTCCCATGATCAGTAAAAATCACTGCTATTTTATTATT
ACTATCTTTACCAGTTTTAGCAGGAGCTATTACCATACTTTTAACAGATCGAAATTTAAA
CACTTCATTTTTTGACCCATCTGGAGGAGGGGACCCAATTCTTTACCAACATTTATTT

>KR783858;tax=d:Eukarya,p:Arthropoda,c:Insecta,o:Hymenoptera,f:Halictidae,g:Lasioglossum,s:Lasioglossum imitatum
AATTTATAATACTATTATTACTTCTCATGCATTCGTAATAATTTTTTTTATAGTTATACC
ATTTATAATTGGAGGATTCGGTAATTGATTAGTACCTTTAATAATTGGTGCTCCCGATAT
AGCATTCCCTCGAATAAATAATATAAGATTTTGATTACTTATTCCTTCAATATTTATATT
ATTGATAAGAAGTATTATATCTTCTGGATCAGGAACTGGGTGAACTGTATACCCCCCCTT
ATCTTCAATTATATACCACTCATCAATTTCAGTAGATTATACTATTTTTTCATTACATAT
TGCAGGAATTTCATCTATTATAGGTGCCATCAATTTTATTGTATCTATTATACTTATAAA
AAATATTTCAATTAATTATGATCAAATTCCTTTATTCCCATGATCAGTAAAAATCACTGC
TATTTTATTATTACTATCTTTACCAATTTTAGCAGGAGCTATTACCATACTTTTAACAGA
TCGAAATTTAAACACTTCATTTTTTGATCCATCTGGAGGAGGAGATCCAATTCTTTACCA
ACATTTATTT
>KR787911;tax=d:Eukarya,p:Arthropoda,c:Insecta,o:Hymenoptera,f:Halictidae,g:Lasioglossum,s:Lasioglossum imitatum
AATACTTTATTTTATTTTTGCTATATGATCTGGAATAATTGGAGCTTCATTAAGAATAAT
TATTCGAATAGAACTAAGTGCACCAGGAAAATGAATTAATAACGATCAAATTTATAATAC
TATTATTACTTCTCATGCATTTGTAATAATTTTTTTTATAGTTATACCATTTATAATTGG
AGGATTCGGTAACTGATTAGTACCTTTAATAATTGGTGCTCCCGATATAGCATTCCCTCG
AATAAATAATATAAGATTTTGATTACTTATTCCTTCAATATTTATATTATTAATAAGAAG
TATTATATCTTCTGGATCAGGAACTGGATGAACTGTATACCCCCCCTTATCTTCAATTAT
ATACCACTCATCAATTTCAGTAGATTATACTATTTTTTCATTACATATTGCAGGAATTTC
ATCTATTATAGGTGCCATCAATTTTATTGTATCTATTATACTTATAAAAAATATTTCAAT
TAATTATGATCAAATTCCTTTATTCCCATGATCAGTAAAAATCACTGCTATTTTATTATT
ACTATCTTTACCAGTTTTAGCAGGAGCTATTACCATACTTTTAACAGATCGAAATTTAAA
CACTTCATTTTTTGACCCATCTGGAGGAGGAGATCCAATTCTTTACCAACATTTATTT
>KR803247;tax=d:Eukarya,p:Arthropoda,c:Insecta,o:Hymenoptera,f:Halictidae,g:Lasioglossum,s:Lasioglossum imitatum
ACTTTATTTTATTTTTGCTATATGATCTGGAATAATTGGAGCTTCATTAAGAATAATTAT
TCGAATAGAACTAAGTGCACCAGGAAAATGAATTAATAACGATCAAATTTATAATACTAT
TATTACTTCTCATGCATTCGTAATAATTTTTTTTATAGTTATACCATTTATAATTGGGGG
ATTCGGTAATTGATTAGTCCCTTTAATAATTGGTGCTCCCGATATAGCATTCCCTCGAAT
AAATAATATAAGATTTTGATTACTTATTCCTTCAATATTTATATTATTAATAAGAAGTAT
TATATCTTCTGGGTCAGGAACTGGATGAACTGTATACCCCCCCTTATCTTCAATTATATA
CCACTCATCAATTTCAGTAGATTATACTATTTTTTCATTACATATTGCAGGAATTTCATC
TATTATAGGTGCCATCAATTTTATTGTATCTATTATACTTATAAAAAATATTTCAATTAA
TTATGATCAAATTCCTTTATTCCCATGATCAGTAAAAATCACTGCTATTTTATTATTACT
ATCTTTACCAGTTTTAGCAGGAGCTATTACCATACTTTTAACAGATCGAAATTTAAACAC
TTCATTTTTTGACCCATCTGGGGGAGGAGACCCAATTCTTTACCAACATTTATTT
>KR883043;tax=d:Eukarya,p:Arthropoda,c:Insecta,o:Hymenoptera,f:Halictidae,g:Lasioglossum,s:Lasioglossum imitatum
AATACTTTATTTTATTTTTGCTATATGATCTGGAATAATTGGAGCTTCATTAAGAATAAT
TATTCGAATAGAACTAAGTGCACCAGGAAAATGAATTAATAACGATCAAATTTATAATAC
TATTATTACTTCTCATGCATTCGTAATAATTTTTTTTATAGTTATACCATTTATAATTGG
GGGATTCGGTAACTGATTAGTCCCTTTAATAATTGGTGCTCCCGATATAGCATTCCCTCG
AATAAATAATATAAGATTTTGATTACTTATTCCTTCAATATTTATATTATTAATAAGAAG
TATTATATCTTCTGGATCAGGGACTGGATGAACTGTATACCCCCCCTTATCTTCAATTAT
ATACCACTCATCAATTTCAGTAGATTATACTATTTTTTCATTACATATTGCAGGAATTTC
ATCTATTATAGGTGCCATCAATTTTATTGTATCTATTATACTTATAAAAAATATTTCAAT
TAATTATGATCAAATTCCTTTATTCCCATGATCAGTAAAAATCACTGCTATTTTATTATT
ACTATCTTTACCAGTTTTAGCAGGAGCTATTACCATACTTTTAACA

>KR884680;tax=d:Eukarya,p:Arthropoda,c:Insecta,o:Hymenoptera,f:Halictidae,g:Lasioglossum,s:Lasioglossum imitatum
TTTATTTTTGCTATATGATCTGGAATAATTGGAGCTTCATTAAGAATAATTATTCGAATA
GAACTAAGTGCACCAGGAAAATGAATTAATAACGATCAAATTTATAATACTATTATTACT
TCTCATGCATTCGTAATAATTTTTTTTATAGTTATACCATTTATAATTGGGGGATTCGGT
AACTGATTAGTCCCTTTAATAATTGGTGCTCCCGATATAGCATTCCCTCGAATAAATAAT
ATAAGATTTTGATTACTTATTCCTTCAATATTTATATTATTAATAAGAAGTATTATATCT
TCTGGATCAGGGACTGGATGAACTGTATACCCCCCCTTATCTTCAATTATATACCACTCA
TCAATTTCAGTAGATTATACTATTTTTTCATTACATATTGCAGGAATTTCATCTATTATA
GGTGCCATCAATTTTATTGTATCTATTATACTTATAAAAAATATTTCAATTAATTATGAT
CAAATTCCTTTATTCCCATGATCAGTAAAAATCACTGCTATTTTATTATTACTATCTTTA
CCAGTTTTAGCAGGAGCTATTACCATACTTTTAACAGAT
>KR891665;tax=d:Eukarya,p:Arthropoda,c:Insecta,o:Hymenoptera,f:Halictidae,g:Lasioglossum,s:Lasioglossum imitatum
ATACTTTATTTTATTTTTGCTATATGATCTGGAATAATTGGAGCTTCATTAAGAATAATT
ATTCGAATAGAACTAAGTGCACCAGGAAAATGAATTAATAACGATCAAATTTATAATACT
ATTATTACTTCTCATGCATTCGTAATAATTTTTTTTATAGTTATACCATTTATAATTGGG
GGATTCGGTAACTGATTAGTCCCTTTAATAATTGGTGCTCCCGATATAGCATTCCCTCGA
ATAAATAATATAAGATTTTGATTACTTATTCCTTCAATATTTATATTATTAATAAGAAGT
ATTATATCTTCTGGATCAGGGACTGGATGAACTGTATACCCCCCCTTATCTTCAATTATA
TACCACTCATCAATTTCAGTAGATTATACTATTTTTTCATTACATATTGCAGGAATTTCA
TCTATTATAGGTGCCATCAATTTTATTGTATCTATTATACTTATAAAAAATATTTCAATT
AATTATGATCAAATTCCTTTATTCCCATGATCAGTAAAAATCACTGCTATTTTATTATTA
CTATCTTTACCAGTTTTA
>KT123227;tax=d:Eukarya,p:Arthropoda,c:Insecta,o:Hymenoptera,f:Halictidae,g:Lasioglossum,s:Lasioglossum imitatum
ATAGAACTAAGTGCACCAGGAAAATGAATTAATAACGATCAAATTTATAATACTATTATT
ACTTCTCATGCATTTGTAATAATTTTTTTTATAGTTATACCATTTATAATTGGAGGATTC
GGTAACTGATTAGTACCTTTAATAATTGGTGCTCCCGATATAGCATTCCCTCGAATAAAT
AATATAAGATTTTGATTACTTATTCCTTCAATATTTATATTATTAATAAGAAGTATTATA
TCTTCTGGATCAGGAACTGGATGAACTGTATACCCCCCCTTATCTTCAATTATATACCAC
TCATCAATTTCAGTAGATTATACTATTTTTTCATTACATATTGCAGGAATTTCATCTATT
ATAGGTGCCATCAATTTTATTGTATCTATTATACTTATAAAAAATATTTCAATTAATTAT
GATCAAATTCCTTTATTCCCATGATCAGTAAAAATCACTGCTATTTTATTATTACTATCT
TTACCAGTTTTA
>KT123228;tax=d:Eukarya,p:Arthropoda,c:Insecta,o:Hymenoptera,f:Halictidae,g:Lasioglossum,s:Lasioglossum imitatum
GAACTAAGTGCACCAGGAAAATGAATTAATAACGATCAAATTTATAATACTATTATTACT
TCTCATGCATTCGTAATAATTTTTTTTATAGTTATACCATTTATAATTGGAGGATTTGGT
AACTGATTAGTACCTTTAATAATTGGTGCTCCCGATATAGCATTCCCTCGAATAAATAAT
ATAAGATTTTGATTACTTATTCCTTCAATATTTATATTATTAATAAGAAGTATTATATCT
TCTGGATCAGGAACTGGATGAACTGTATACCCCCCCTTATCTTCAATTATATACCACTCA
TCAATTTCAGTAGATTATACTATTTTTTCATTGCATATTGCAGGAATTTCATCTATTATA
GGTGCCATCAATTTTATTGTATCTATTATACTTATAAAAAATATTTCAATTAATTATGAT
CAAATTCCTTTATTCCCATGATCAGTAAAAATCACTGCTATTTTATTATTACTATCTTTA
CCAGTTTTAGCAGGAGCTATTACCATACTTTTAACAGATCGAAATTTAAACACTTCATTT
TTTGACCCATCTGGAGGAGGAGATCCAATTCTTTACCAACATTTATTT
>KT708217;tax=d:Eukarya,p:Arthropoda,c:Insecta,o:Hymenoptera,f:Halictidae,g:Lasioglossum,s:Lasioglossum imitatum
AATACTTTATTTTATTTTTGCTATATGATCTGGAATAATTGGAGCTTCATTAAGAATAAT
TATTCGAATAGAACTAAGTGCACCAGGAAAATGAATTAATAACGATCAAATTTATAATAC

TATTATTACTTCTCATGCATTCGTAATAATTTTTTTTATAGTTATACCATTTATAATTGG
GGGATTCGGTAATTGATTAGTCCCTTTAATAATTGGTGCTCCCGATATAGCATTCCCTCG
AATAAATAATATAAGATTTTGATTACTTATTCCTTCAATATTTATATTATTAATAAGAAG
TATTATATCTTCTGGGTCAGGAACTGGATGAACTGTATACCCCCCCTTATCTTCAATTAT
ATACCACTCATCAATTTCAGTAGATTATACTATTTTTTCATTACATATTGCAGGAATTTC
ATCTATTATAGGTGCCATCAATTTTATTGTATCTATTATACTTATAAAAAATATTTCAAT
TAATTATGATCAAATTCCTTTATTCCCATGATCAGTAAAAATCACTGCTATTTTATTATT
ACTATCTTTACCAGTTTTAGCAGGAGCTATTACCATACTTTTAACAGATCGAAATTTAAA
CACTTCATTTTTTGACCCATCTGGGGGAGGAGACCCAAT
>MG334666;tax=d:Eukarya,p:Arthropoda,c:Insecta,o:Hymenoptera,f:Halictidae,g:Lasioglossum,s:Lasioglossum imitatum
ATACTTTATTTTATTTTTGCTATATGATCTGGAATAATTGGAGCTTCATTAAGAATAATT
ATTCGAATAGAACTAAGTGCACCAGGAAAATGAATTAATAACGATCAAATTTATAATACT
ATTATTACTTCTCATGCATTCGTAATAATTTTTTTTATAGTTATACCATTTATAATTGGG
GGATTCGGTAACTGATTAGTCCCTTTAATAATTGGTGCTCCCGATATAGCATTCCCTCGA
ATAAATAATATAAGATTTTGATTACTTATTCCTTCAATATTTATATTATTAATAAGAAGT
ATTATATCTTCTGGATCAGGGACTGGATGAACTGTATACCCCCCCTTATCTTCAATTATA
TACCACTCATCAATTTCAGTAGATTATACTATTTTTTCATTACATATTGCAGGAATTTCA
TCTATTATAGGTGCCATCAATTTTATTGTATCTATTATACTTATAAAAAATATTTCAATT
AATTATGATCAAATTCCTTTATTCCCATGATCAGTAAAAATCACTGCTATTTTATTATTA
CTATCTTTACCAGTTTTAGCAGGAGCTATTACCATACTTTTAACAGAT
>MG334859;tax=d:Eukarya,p:Arthropoda,c:Insecta,o:Hymenoptera,f:Halictidae,g:Lasioglossum,s:Lasioglossum imitatum
ATACTTTATTTTATTTTTGCTATATGATCTGGAATAATTGGAGCTTCATTAAGAATAATT
ATTCGAATAGAACTAAGTGCACCAGGAAAATGAATTAATAACGATCAAATTTATAATACT
ATTATTACTTCTCATGCATTCGTAATAATTTTTTTTATAGTTATACCATTTATAATTGGG
GGATTCGGTAACTGATTAGTCCCTTTAATAATTGGTGCTCCCGATATAGCATTCCCTCGA
ATAAATAATATAAGATTTTGATTACTTATTCCTTCAATATTTATATTATTAATAAGAAGT
ATTATATCTTCTGGATCAGGGACTGGATGAACTGTATACCCCCCCTTATCTTCAATTATA
TACCACTCATCAATTTCAGTAGATTATACTATTTTTTCATTACATATTGCAGGAATTTCA
TCTATTATAGGTGCCATCAATTTTATTGTATCTATTATACTTATAAAAAATATTTCAATT
AATTATGATCAAATTCCTTTATTCCCATGATCAGTAAAAATCACTGCTATTTTATTATTA
CTATCTTTACCAGTTTTAGCAGGAGCTATTACCATACTTTTAACA
>MG335206;tax=d:Eukarya,p:Arthropoda,c:Insecta,o:Hymenoptera,f:Halictidae,g:Lasioglossum,s:Lasioglossum imitatum
ATACTTTATTTTATTTTTGCTATATGATCTGGAATAATTGGAGCTTCATTAAGAATAATT
ATTCGAATAGAATTAAGTGCACCAGGAAAATGAATTAATAACGATCAAATTTATAATACT
ATTATTACTTCTCATGCATTCGTAATAATTTTTTTTATAGTTATACCATTTATAATTGGA
GGATTCGGTAATTGATTAGTACCTTTAATAATTGGTGCTCCCGATATAGCATTCCCTCGA
ATAAATAATATAAGATTTTGATTACTTATTCCTTCAATATTTATATTATTGATAAGAAGT
ATTATATCTTCTGGATCAGGAACTGGGTGAACTGTATACCCCCCCTTATCTTCAATTATA
TACCACTCATCAATTTCAGTAGATTATACTATTTTTTCATTACATATTGCAGGAATTTCA
TCTATTATAGGTGCCATCAATTTTATTGTATCTATTATACTTATAAAAAATATTTCAATT
AATTATGATCAAATTCCTTTATTCCCATGATCAGTAAAAATCACTGCTATTTTATTATTA
CTATCTTTACCAGTTTTAGCAGGAGCTATTACCATACTTTTAACAGAT
>MG335445;tax=d:Eukarya,p:Arthropoda,c:Insecta,o:Hymenoptera,f:Halictidae,g:Lasioglossum,s:Lasioglossum imitatum
CTTTATTTTATTTTTGCTATATGATCTGGAATAATTGGAGCTTCATTAAGAATAATTATT
CGAATAGAACTAAGTGCACCAGGAAAATGAATTAATAACGATCAAATTTATAATACTATT
ATTACTTCTCATGCATTCGTAATAATTTTTTTTATAGTTATACCATTTATAATTGGGGGA
TTCGGTAACTGATTAGTCCCTTTAATAATTGGTGCTCCCGATATAGCATTCCCTCGAATA

AATAATATAAGATTTTGATTACTTATTCCTTCAATATTTATATTATTAATAAGAAGTATT
ATATCTTCTGGATCAGGGACTGGATGAACTGTATACCCCCCCTTATCTTCAATTATATAC
CACTCATCAATTTCAGTAGATTATACTATTTTTTCATTACATATTGCAGGAATTTCATCT
ATTATAGGTGCCATCAATTTTATTGTATCTATTATACTTATAAAAAATATTTCAATTAAT
TATGATCAAATTCCTTTATTCCCATGATCAGTAAAAATCACTGCTATTTTATTATTACTA
TCTTTACCAGTTTTAGCAGGAGCTATTACCATACTTTTAACAGATCGAAATTTAAAC
>MG336475;tax=d:Eukarya,p:Arthropoda,c:Insecta,o:Hymenoptera,f:Halictidae,g:Lasioglossum,s:Lasioglossum imitatum
ATTTTTGCTATATGATCTGGAATAATTGGAGCTTCATTAAGAATAATTATTCGAATAGAA
CTAAGTGCACCAGGAAAATGAATTAATAACGATCAAATTTATAATACTATTATTACTTCT
CATGCATTCGTAATAATTTTTTTTATAGTTATACCATTTATAATTGGGGGATTCGGTAAC
TGATTAGTCCCTTTAATAATTGGTGCTCCCGATATAGCATTCCCTCGAATAAATAATATA
AGATTTTGATTACTTATTCCTTCAATATTTATATTATTAATAAGAAGTATTATATCTTCT
GGATCAGGGACTGGATGAACTGTATACCCCCCCTTATCTTCAATTATATACCACTCATCA
ATTTCAGTAGATTATACTATTTTTTCATTACATATTGCAGGAATTTCATCTATTATAGGT
GCCATCAATTTTATTGTATCTATTATACTTATAAAAAATATTTCAATTAATTATGATCAA
ATTCCTTTATTCCCATGATCAGTAAAAATCACTGCTATTTTATTATTACTATCTTTACCA
GTTTTAGCAGGAGCTATTACCATACTTTTAACAGAT
>MG339213;tax=d:Eukarya,p:Arthropoda,c:Insecta,o:Hymenoptera,f:Halictidae,g:Lasioglossum,s:Lasioglossum imitatum
CTTTATTTTATTTTTGCTATATGATCTGGAATAATTGGAGCTTCATTAAGAATAATTATT
CGAATAGAACTAAGTGCACCAGGAAAATGAATTAATAACGATCAAATTTATAATACTATT
ATTACTTCTCATGCATTCGTAATAATTTTTTTTATAGTTATACCATTTATAATTGGGGGA
TTCGGTAACTGATTAGTCCCTTTAATAATTGGTGCTCCCGATATAGCATTCCCTCGAATA
AATAATATAAGATTTTGATTACTTATTCCTTCAATATTTATATTATTAATAAGAAGTATT
ATATCTTCTGGATCAGGGACTGGATGAACTGTATACCCCCCCTTATCTTCAATTATATAC
CACTCATCAATTTCAGTAGATTATACTATTTTTTCATTACATATTGCAGGAATTTCATCT
ATTATAGGTGCCATCAATTTTATTGTATCTATTATACTTATAAAAAATATTTCAATTAAT
TATGATCAAATTCCTTTATTCCCATGATCAGTAAAAATCACTGCTATTTTATTATTACTA
TCTTTACCAGTTTTAGCAGGAGCTATTACCATACTTTTAACAGATCGAAAT
>MG341821;tax=d:Eukarya,p:Arthropoda,c:Insecta,o:Hymenoptera,f:Halictidae,g:Lasioglossum,s:Lasioglossum imitatum
TTTATTTTTGCTATATGATCTGGAATAATTGGAGCTTCATTAAGAATAATTATTCGAATA
GAACTAAGTGCACCAGGAAAATGAATTAATAACGATCAAATTTATAATACTATTATTACT
TCTCATGCATTCGTAATAATTTTTTTTATAGTTATACCATTTATAATTGGGGGATTCGGT
AACTGATTAGTCCCTTTAATAATTGGTGCTCCCGATATAGCATTCCCTCGAATAAATAAT
ATAAGATTTTGATTACTTATTCCTTCAATATTTATATTATTAATAAGAAGTATTATATCT
TCTGGATCAGGGACTGGATGAACTGTATACCCCCCCTTATCTTCAATTATATACCACTCA
TCAATTTCAGTAGATTATACTATTTTTTCATTACATATTGCAGGAATTTCATCTATTATA
GGTGCCATCAATTTTATTGTATCTATTATACTTATAAAAAATATTTCAATTAATTATGAT
CAAATTCCTTTATTCCCATGATCAGTAAAAATCACTGCTATTTTATTATTACTATCTTTA
CCAGTTTTAGCAGGAGCTATTACCATA
>MG343715;tax=d:Eukarya,p:Arthropoda,c:Insecta,o:Hymenoptera,f:Halictidae,g:Lasioglossum,s:Lasioglossum imitatum
AATACTTTATTTTATTTTTGCTATATGATCTGGAATAATTGGAGCTTCATTAAGAATAAT
TATTCGAATAGAACTAAGTGCACCAGGAAAATGAATTAATAACGATCAAATTTATAATAC
TATTATTACTTCTCATGCATTCGTAATAATTTTTTTTATAGTTATACCATTTATAATTGG
GGGATTCGGTAACTGATTAGTCCCTTTAATAATTGGTGCTCCCGATATAGCATTCCCTCG
AATAAATAATATAAGATTTTGATTACTTATTCCTTCAATATTTATATTATTAATAAGAAG
TATTATATCTTCTGGATCAGGGACTGGATGAACTGTATACCCCCCCTTATCTTCAATTAT
ATACCACTCATCAATTTCAGTAGATTATACTATTTTTTCATTACATATTGCAGGAATTTC

ATCTATTATAGGTGCCATCAATTTTATTGTATCTATTATACTTATAAAAAATATTTCAAT
TAATTATGATCAAATTCCTTTATTCCCATGATCAGTAAAAATCACTGCTATTTTATTATT
ACTATCTTTACCAGTTTTAGCAGGAGCTATTACCATACTTTTAACAGAT
>KJ088124;tax=d:Eukarya,p:Arthropoda,c:Insecta,o:Hymenoptera,f:Halictidae,g:Lasioglossum,s:Lasioglossum pilosum
AATACTTTATTTTATTTTTGCAATATGAGCTGGTATAATTGGAGCTTCATTAAGAATAAT
TATTCGAATAGAATTAAGTGCCCCAGGAAAATGAATTAATAATGATCAAATTTATAACAC
CATTATTACTTCTCATGCATTTGTAATAATTTTTTTTATAGTTATACCATTTATAATTGG
AGGATTCGGTAATTGATTAATCCCTTTAATAATTGGTGCACCTGATATAGCATTTCCTCG
AATAAATAATATAAGATTTTGATTACTTATCCCATCAATATTTATATTATTAATAAGAAG
TATTATTTCATCAGGATCAGGAACTGGATGAACTGTATATCCCCCCTTATCTTCTATTAT
ATACCATTCATCAACTTCAGTAGATTACACTATTTTCTCATTACATATTGCAGGAATTTC
ATCAATTATAGGAGCCATTAATTTTATTGTATCTATTCTTCTTATAAAAAATATTTCACT
TAATTATGATCAAATCCCTTTATTCCCATGATCAGTAAAAATTACTGCTATTTTATTATT
ATTATCTTTACCAGTTTTAGCAGGAGCTATCACTATACTTTTAACAGATCGAAACTTAAA
TACTTCATTTTTTG
>KT123230;tax=d:Eukarya,p:Arthropoda,c:Insecta,o:Hymenoptera,f:Halictidae,g:Lasioglossum,s:Lasioglossum pilosum
ATAGAATTAAGTGCCCCGGGAAAATGAATTAATAATGATCAAATTTATAACACCATTATT
ACTTCTCATGCATTTGTAATAATTTTTTTTATAGTTATACCATTTATAATTGGAGGATTC
GGTAATTGATTAATCCCTTTAATAATTGGTGCACCTGATATAGCATTTCCTCGAATAAAT
AATATAAGATTTTGATTACTTATCCCATCATTATTTATATTATTAATAAGAAGTATTATT
TCATCAGGATCAGGAACTGGATGAACTGTATATCCCCCCTTATCTTCTATTATATACCAT
TCATCAACTTCAGTAGATTACACTATTTTCTCATTACATATTGCAGGAATTTCATCAATT
ATAGGAGCCATTAATTTTATTGTATCTATTCTTCTTATAAAAAATATTTCACTTAATTAT
GATCAAATCCCTTTATTCCCATGATCAGTAAAAATTACTGCTATTTTATTATTATTATCT
TTACCAGTTTTAGCAGGAGCTATCACTATACTTTTAACAGATCGAAACTTAAATACTTCA
TTTTTTGACCCCTCTGGTGGTGGAGACCCTATTCTTTACCAACATTTATTT
>KT123232;tax=d:Eukarya,p:Arthropoda,c:Insecta,o:Hymenoptera,f:Halictidae,g:Lasioglossum,s:Lasioglossum tegulare
GAATTAAGTGCTCCCGGAAAATGAATTAATAATGATCAAATTTATAACACTATTATTACT
TCTCATGCATTCGTAATAATTTTTTTTATAGTTATACCATTTATAATTGGAGGATTTGGG
AATTGATTAGTTCCTTTAATAATTGGGGCACCGGATATAGCATTTCCTCGAATAAATAAT
ATAAGATTTTGATTACTTATTCCATCAATATTTATATTATTAATAAGAAGAATTATATCT
TCTGGTTCAGGAACTGGATGAACTATTTATCCACCTTTATCTTCAATTTTATATCATTCA
TCTACTTCTGTAGATTACACCATTTTTTCATTACATATTGCAGGAATTTCATCTATTATA
GGAGCAATTAATTTTATTGTATCTATTTTATTAATAAAAAATATTTCAATTAATTATGAT
CAAATTCCTTTATTTCCATGATCAGTAAAAATTACTGCTATCTTATTATTATTATCTTTA
CCAGTACTAGCAGGAGCAATTACTATACTTTTAACAGATCGAAATTTAAATACTTCTTTC
TTTGACCCTTCAGGAGGAGGAGATCCAATTCTTTATCAACATTTATTT
>FJ663120;tax=d:Eukarya,p:Arthropoda,c:Insecta,o:Hymenoptera,f:Halictidae,g:Lasioglossum,s:Lasioglossum vierecki
TATACTTTATTTTATTTTCGCAATATGATCAGGAATAATTGGAGCTTCTTTAAGAATAAT
TATTCGAATAGAATTAAGTGCTCCTGGAAAATGAATTAATAATGATCAAATCTATAATAC
TATTATTACATCACATGCATTTGTAATAATTTTTTTCATAGTAATACCATTTATAATTGG
GGGATTTGGAAATTGATTAGTACCTTTAATAATTGGAGCTCCTGATATAGCATTTCCTCG
AATAAATAATATAAGATTTTGATTACTAATTCCATCAATATTTATATTATTAATAAGAAG
AATTATATCATCTGGATCAGGAACTGGATGAACTGTATATCCACCTTTATCTTCTATTAT
ATACCACTCATCTATCTCAGTAGATTACACTATTTTTTCACTTCATATTGCAGGAATTTC
ATCTATTATAGGAGCAATTAATTTTATTGTATCTATTTTACTTATAAAAAATATTTCAAT
AAATTATGACCAAATTCCTTTATTTCCATGATCAGTAAAAATTACTGCCATCTTATTATT

ATTATCTTTACCAGTCTTAGCAGGAGCTATTACTATACTTTTAACTGATCGAAATTTAAA
TACTTCATTTTTTGATCCTTCAGGTGGAGGAGATCCAATTCTTTATCAACATTTATTT
>FJ663126;tax=d:Eukarya,p:Arthropoda,c:Insecta,o:Hymenoptera,f:Halictidae,g:Lasioglossum,s:Lasioglossum vierecki
TATACTTTATTTTATTTTCGCAATATGATCAGGAATAATTGGAGCTTCTTTAAGAATAAT
TATTCGAATAGAATTAAGTGCTCCTGGAAAATGAATTAATAATGATCAAATCTATAATAC
TATTATTACATCACATGCATTTGTAATAATTTTTTTCATAGTAATACCATTTATAATTGG
GGGATTTGGAAATTGATTAGTACCTTTAATAATTGGAGCTCCTGATATAGCATTTCCTCG
AATAAATAATATAAGATTTTGATTACTAATTCCATCAATATTTATATTATTAATAAGAAG
AATTATATCATCTGGATCAGGAACTGGATGAACTGTATATCCACCTTTATCTTCTATTAT
ATACCACTCATCTATCTCAGTAGATTACACTATTTTTTCACTTCATATTGCAGGAATTTC
ATCTATTATAGGAGCAATTAATTTTATTGTATCTATTTTACTTATAAAAAATATTTCAAT
AAATTATGACCAAATTCCTTTATTTCCATGATCAGTAAAAATTACTGCCATCTTATTATT
ATTATCTTTACCAGTCTTAGCAGGAGCTATTACTATACTTTTAACTGATCGAAATTTAAA
TACTTCATTTTTTGATCCTTCAGGTGGAGGAGATCCAATTCTTTATCAACATTT
>KR795938;tax=d:Eukarya,p:Arthropoda,c:Insecta,o:Hymenoptera,f:Halictidae,g:Lasioglossum,s:Lasioglossum vierecki
TATACTTTATTTTATTTTCGCAATATGATCAGGAATAATTGGAGCTTCTTTAAGAATAAT
TATTCGAATAGAATTAAGTGCTCCTGGAAAATGAATTAATAATGATCAAATCTATAATAC
TATTATTACATCACATGCATTTGTAATAATTTTTTTCATAGTAATACCATTTATAATTGG
GGGATTTGGAAATTGATTAGTACCTTTAATAATTGGAGCTCCTGATATAGCATTTCCTCG
AATAAATAATATAAGATTTTGATTACTAATTCCATCAATATTTATATTATTAATAAGAAG
AATTATATCATCTGGATCAGGAACTGGATGAACTGTATATCCACCTTTATCTTCTATTAT
ATACCACTCATCTATCTCAGTAGATTACACTATTTTTTCACTTCATATTGCAGGAATTTC
ATCTATTATAGGAGCAATTAATTTTATTGTATCTATTTTACTTATAAAAAATATTTCAAT
AAATTATGACCAAATTCCTTTATTTCCATGATCAGTAAAAATTACTGCCATCTTATTATT
ATTATCTTTACCAGTCTTAGCAGGAGCTATTACTATACTTTTAACTGATCGAAATTTAAA
TACTTCATTTTTTGATCCTTCAGGTGGAGGAGATCCAATTCTTTATCAA
>JF903565;tax=d:Eukarya,p:Arthropoda,c:Insecta,o:Hymenoptera,f:Halictidae,g:Lasioglossum,s:Lasioglossum zephyrum
ATACTTTATTTTATTTTTGCTATATGATCTGGAATAATTGGAGCTTCATTAAGAATAATT
ATTCGAATAGAATTAAGTGCACCAGGAAAATGAATTAATAATGATCAAATTTATAACACT
ATTATTACTTCACATGCATTTGTAATAATTTTTTTTATAGTTATACCATTTATAATTGGA
GGATTTGGTAATTGATTAGTTCCTTTAATAATTGGTGCACCCGATATAGCATTCCCTCGA
ATAAATAATATAAGATTTTGATTACTTATTCCATCAATATTTATATTATTAATAAGAAGT
ATTATATCATCTGGATCAGGAACTGGATGAACTATTTACCCTCCCCTATCTTCAATTATA
TATCATTCATCAATTTCAGTAGACTATACTATCTTTTCATTACATATTGCAGGAATTTCA
TCTATTATAGGAGCAATCAACTTTATTGTATCTATCCTACTTATAAAAAATATTTCAATT
AATTATGATCAAATTCCTTTATTTCCATGATCAGTAAAAATTACTGCTATTTTATTATTA
TTATCCTTACCAGTTTTAGCAGGAGCTATTACTATACTTTTAACAGATCGAAATTTAAAT
ACTTCATTTTTTGACCCTTCTGGAGGAGGAGATCCAATTCTTTACCAACATTTATTT
>FJ582295;tax=d:Eukarya,p:Arthropoda,c:Insecta,o:Hymenoptera,f:Halictidae,g:Lasioglossum,s:Lasioglossum pectorale
AATATTATATTTTATCTTTGCAATATGAGCAGGTATAATCGGAGCTTCTTTAAGAATAAT
TATTCGAATAGAATTAAGAGCTCCAGGAAGATGAATTAATAATGACCAAATTTATAATAC
AATTATTACTTCACATGCATTTATTATAATTTTTTTTATAGTTATACCTTTTATAATTGG
AGGATTTGGTAATTGACTAGTACCTTTAATAATTGGAGCTCCAGATATAGCTTTCCCACG
AATAAATAATATAAGATTTTGACTTCTTATTCCATCCTTATTTATATTATTAATAAGAAG
AATTTTAGCATCCGGTTCAGGAACAGGATGAACAGTCTATCCTCCTTTATCTTCAATTAT
ATACCATTCATCTATTTCTGTAGATTGTACAATTTTTTCATTACATATTGCAGGAATTTC
TTCAATTATAGGAGCAATCAATTTTATTGTTTCAATTATATTAATAAAAAATATTTCAAT

TAATTATGATCAAATTCCATTATTTCCATGATCAGTAAAAATTACTGCTATTCTATTACT
TTTATCTTTACCTATTCTAGCAGGAGCTATTACAATACTTTTAACTGATCGAAACTTAAA
TACATCATTTTTTGACCCTTCAGGAGGAGGAGATCCAATTTTATATCAACATTTATTT
>FJ582296;tax=d:Eukarya,p:Arthropoda,c:Insecta,o:Hymenoptera,f:Halictidae,g:Lasioglossum,s:Lasioglossum pectorale
ATAATCGGAGCTTCTTTAAGAATAATTATTCGAATAGAATTAAGAGCTCCAGGAAGATGA
ATTAATAATGACCAAATTTATAATACAATTATTACTTCACATGCATTTATTATAATTTTT
TTTATAGTTATACCTTTTATAATTGGAGGATTTGGTAATTGACTAGTACCTTTAATAATT
GGAGCTCCAGATATAGCTTTCCCACGAATAAATAATATAAGATTTTGACTTCTTATTCCA
TCCTTATTTATATTATTAATAAGAAGAATTTTAGCATCCGGTTCAGGAACAGGATGAACA
GTCTATCCTCCTTTATCTTCAATTATATACCATTCATCTATTTCTGTAGATTGTACAATT
TTTTCATTACATATTGCAGGAATTTCTTCAATTATAGGAGCAATCAATTTTATTGTTTCA
ATTATATTAATAAAAAATATTTCAATTAATTATGATCAAATTCCATTATTTCCATGATCA
GTAAAAATTACTGCTATTCTATTACTTTTATCTTTACCTATTCTAGCAGGAGCTATTACA
ATACTTTTAACTGATCGAAACTTAAATACATCATTTTTTGACCCTTCAGGAGGAGGAGAT
CCAATTTTATATCAACATTTATTT
>FJ582297;tax=d:Eukarya,p:Arthropoda,c:Insecta,o:Hymenoptera,f:Halictidae,g:Lasioglossum,s:Lasioglossum pectorale
GTATAATCGGAGCTTCTTTAAGAATAATTATTCGAATAGAATTAAGAGCTCCAGGAAGAT
GAATTAATAATGACCAAATTTATAATACAATTATTACTTCACATGCATTTATTATAATTT
TTTTTATAGTTATACCTTTTATAATTGGAGGATTTGGTAATTGACTAGTACCTTTAATAA
TTGGAGCTCCAGATATAGCTTTCCCACGAATAAATAATATAAGATTTTGACTTCTTATTC
CATCCTTATTTATATTATTAATAAGAAGAATTTTAGCATCCGGTTCAGGAACAGGATGAA
CAGTCTATCCTCCTTTATCTTCAATTATATACCATTCATCTATTTCTGTAGATTGTACAA
TTTTTTCATTACATATTGCAGGAATTTCTTCAATTATAGGAGCAATCAATTTTATTGTTT
CAATTATATTAATAAAAAATATTTCAATTAATTATGATCAAATTCCATTATTTCCATGAT
CAGTAAAAATTACTGCTATTCTATTACTTTTATCTTTACCTATTCTAGCAGGAGCTATTA
CAATACTTTTAACTGATCGAAACTTAAATACATCATTTTTTGACCCTTCAGGAGGAGGAG
ATCCAATTTTATATCAACATTTATTT
>HQ959936;tax=d:Eukarya,p:Arthropoda,c:Insecta,o:Hymenoptera,f:Halictidae,g:Lasioglossum,s:Lasioglossum pectorale
GATCGGAGCTTCTTTAAGAATAATTATTCGAATAGAATTAAGAGCTCCAGGAAGATGAAT
TAATAATGACCAAATTTATAATACAATTATTACTTCACATGCATTTATTATAATTTTTTT
TATAGTTATACCTTTTATAATTGGAGGATTTGGTAATTGACTAGTACCTTTAATAATTGG
AGCTCCAGATATAGCTTTCCCACGAATAAATAATATAAGATTTTGACTTCTTATCCCATC
CTTATTTATATTATTAATAAGAAGAATTTTAGCATCCGGTTCAGGAACAGGATGAACAGT
TTATCCTCCTTTATCTTCAATTATATACCACTCATCTATTTCTGTAGATTGTACAATTTT
TTCATTACATATTGCAGGAATTTCTTCAATTATAGGAGCAATCAATTTTATTGTTTCAAT
TATATTAATAAAAAATATTTCAATTAATTATGATCAAATTCCATTATTTCCATGATCAGT
AAAAATTACTGCTATTCTATTACTTTTATCTTTACCTGTTCTAGCAGGAGCTATTACAAT
ACTTTTAACTGATCGAAACTTAAATACATCATTTTTTGACCCTTCAGGAGGAGGAGATCC
AATTTTATATCAACATCTATTT
>KF199974;tax=d:Eukarya,p:Arthropoda,c:Insecta,o:Hymenoptera,f:Halictidae,g:Lasioglossum,s:Lasioglossum pectorale
AATATTATATTTTATCTTTGCAATATGAGCAGGTATAATCGGAGCTTCTTTAAGAATAAT
TATTCGAATAGAATTAAGAGCTCCAGGAAGATGAATTAATAATGACCAAATTTATAATAC
AATTATTACTTCACATGCATTTATCATAATTTTTTTTATAGTTATACCTTTTATAATTGG
AGGATTTGGGAATTGATTAGTACCTTTAATAATTGGAGCTCCAGATATAGCTTTTCCACG
AATAAACAATATAAGATTTTGACTTCTTATCCCATCCTTATTTATATTATTAATAAGAAG
AATTTTAGCATCCGGTTCAGGAACAGGATGAACAGTTTATCCTCCTTTATCTTCAATTAT
ATACCATTCATCTATTTCTGTAGATTGTACAATTTTTTCATTACATATTGCAGGAATTTC

TTCAATTATAGGAGCAATCAATTTTATTGTTTCAATTATATTAATAAAAAATATTTCAAT
TAATTATGATCAAATTCCATTATTTCCATGATCAGTAAAAATTACTGCTATTCTATTACT
TTTATCTTTACCTGTTCTAGCAGGAGCTATTACAATACTTTTAACTGATCGAAACTTAAA
TACATCATTTTTTGACCCTTCAGGAGGAGGAGATCCAATTTTATATCAACATTTATTT
>KF199976;tax=d:Eukarya,p:Arthropoda,c:Insecta,o:Hymenoptera,f:Halictidae,g:Lasioglossum,s:Lasioglossum pectorale
AATATTATATTTTATCTTTGCAATATGAGCAGGTATAATCGGAGCTTCTTTAAGAATAAT
TATTCGAATAGAATTAAGAGCTCCAGGAAGATGAATTAATAATGACCAAATTTATAATAC
AATTATTACTTCACATGCATTTATTATAATTTTTTTTATAGTTATACCTTTTATAATTGG
GGGATTTGGTAATTGACTAGTACCTTTAATAATTGGAGCTCCAGATATAGCTTTCCCACG
AATAAATAATATAAGATTTTGACTTCTTATTCCATCCTTATTTATATTATTAATAAGAAG
AATTTTAGCATCCGGTTCAGGAACAGGGTGAACAGTTTATCCTCCTTTATCTTCAATTAT
ATACCATTCATCTATTTCTGTAGATTGTACAATTTTTTCATTACATATTGCAGGAATTTC
TTCAATTATAGGAGCAATCAATTTTATTGTTTCAATTATATTAATAAAAAATATTTCAAT
TAATTATGATCAAATTCCATTATTTCCATGATCAGTAAAAATTACTGCTATTCTATTACT
TTTATCTTTACCTATTCTAGCAGGAGCTATTACAATACTTTTAACTGATCGAAACTTAAA
TACATCATTTTT
>KF199977;tax=d:Eukarya,p:Arthropoda,c:Insecta,o:Hymenoptera,f:Halictidae,g:Lasioglossum,s:Lasioglossum pectorale
GAGCAGGTATGATCGGAGCTTCTTTAAGAATAATTATTCGAATAGAATTAAGAGCTCCAG
GAAGATGAATTAATAATGACCAAATTTATAATACAATTATTACTTCACATGCATTTATTA
TAATTTTTTTTATAGTTATACCTTTTATAATTGGAGGATTTGGAAATTGATTAGTACCTT
TAATAATTGGAGCTCCAGATATAGCTTTTCCACGAATAAACAATATAAGATTTTGACTTC
TTATCCCATCCTTATTTATATTATTAATAAGAAGAATTTTAGCATCCGGTTCAGGAACAG
GATGAACAGTTTATCCTCCTTTATCTTCAATTATATACCATTCATCTATTTCTGTAGATT
GTACAATTTTTTCATTACATATTGCAGGAATTTCTTCAATTATAGGAGCAATCAATTTTA
TTGTTTCAATTATATTAATAAAAAATATTTCAATTAATTATGATCAAATTCCATTATTTC
CATGATCAGTAAAAATTACTGCTATTCTATTACTTTTATCTTTACCTGTTCTAGCAGGAG
CTATTACAATACTTTTAACTGATCGAAACTTAAATACATCATTTTTTGACCCTTCAGGAG
GAGGGGATCCAATTTTATATCAACATTTATTT
>KF199978;tax=d:Eukarya,p:Arthropoda,c:Insecta,o:Hymenoptera,f:Halictidae,g:Lasioglossum,s:Lasioglossum pectorale
AATATTATATTTTATCTTTGCAATATGAGCAGGTATGATCGGAGCTTCTTTAAGAATAAT
TATTCGAATAGAATTAAGAGCTCCAGGAAGATGAATTAATAATGACCAAATTTATAATAC
AATTATTACTTCACATGCATTTATTATAATTTTTTTTATAGTTATACCTTTTATAATTGG
AGGATTTGGAAATTGATTAGTACCTTTAATAATTGGAGCTCCAGATATAGCTTTTCCACG
AATAAACAATATAAGATTTTGACTTCTTATCCCATCCTTATTTATATTATTAATAAGAAG
AATTTTAGCATCCGGTTCAGGAACAGGATGAACAGTTTATCCTCCTTTATCTTCAATTAT
ATACCATTCATCTATTTCTGTAGATTGTACAATTTTTTCATTACATATTGCAGGAATTTC
TTCAATTATAGGAGCAATCAATTTTATTGTTTCAATTATATTAATAAAAAATATTTCAAT
TAATTATGATCAAATTCCATTATTTCCATGATCAGTAAAAATTACTGCTATTCTATTACT
TTTATCTTTACCTGTTCTAGCAGGAGCTATTACAATACTTTTAACTGATCGAAACTTAAA
TACATCATTTTTTGACCCTTCAGGAGGAGGAGATCCAATTTTATATCAACATTTATTT
>KF199980;tax=d:Eukarya,p:Arthropoda,c:Insecta,o:Hymenoptera,f:Halictidae,g:Lasioglossum,s:Lasioglossum pectorale
AATATTATATTTTATCTTTGCAATATGAGCAGGTATAATCGGAGCTTCTTTAAGAATAAT
TATTCGAATAGAATTAAGAGCTCCAGGAAGATGAATTAATAATGACCAAATTTATAATAC
AATTATTACTTCACATGCATTTATTATAATTTTTTTTATAGTTATACCTTTTATAATTGG
AGGATTTGGGAATTGATTAGTACCTTTAATAATTGGAGCTCCAGATATAGCTTTCCCACG
AATAAACAATATAAGATTTTGACTTCTTATCCCATCCTTATTTATATTATTAATAAGAAG
AATTTTAGCATCCGGTTCAGGAACAGGATGAACAGTTTATCCTCCTTTATCTTCAATTAT

ATACCATTCATCTATTTCTGTAGATTGTACAATTTTTTCATTACATATTGCAGGAATTTC
TTCAATTATAGGAGCAATCAATTTTATTGTTTCAATTATATTAATAAAAAATATTTCAAT
TAATTATGATCAAATTCCATTATTTCCATGATCAGTAAAAATTACTGCTATTCTATTACT
TTTATCTTTACCTGTTCTAGCAGGAGCTATTACAATACTTTTAACTGATCGAAACTTAAA
TACATCATTTTTTGACCCTTCAGGAGGAGGAGATCCAATTTTATATCAACATTT
>KF199985;tax=d:Eukarya,p:Arthropoda,c:Insecta,o:Hymenoptera,f:Halictidae,g:Lasioglossum,s:Lasioglossum pectorale
TATGAGCAGGTATAATCGGAGCTTCTTTAAGAATAATTATTCGAATAGAATTAAGAGCTC
CAGGAAGATGAATTAATAATGACCAAATTTATAATACAATTATTACTTCACATGCATTTA
TCATAATTTTTTTTATAGTTATACCTTTTATAATTGGAGGATTTGGGAATTGATTAGTAC
CTTTAATAATTGGAGCTCCAGATATAGCTTTTCCACGAATAAACAATATAAGATTTTGAC
TTCTTATCCCATCCTTATTTATATTATTAATAAGAAGAATTTTAGCATCCGGTTCAGGAA
CAGGATGAACAGTTTATCCTCCTTTATCTTCAATTATATACCATTCATCTATTTCTGTAG
ATTGTACAATTTTTTCATTACATATTGCAGGAATTTCTTCAATTATAGGAGCAATCAATT
TTATTGTTTCAATTATATTAATAAAAAATATTTCAATTAATTATGATCAAATTCCATTAT
TTCCATGATCAGTAAAAATTACTGCTATTCTATTACTTTTATCTTTACCTGTTCTAGCAG
GAGCTATTACAATACTTTTAACTGATCGAAACTTAAATACATCATTTTTTGACCCTTCAG
GAGGAGGAGATCCAATTTTATATCAACATTTATTT
>KF199987;tax=d:Eukarya,p:Arthropoda,c:Insecta,o:Hymenoptera,f:Halictidae,g:Lasioglossum,s:Lasioglossum pectorale
GGTATAATCGGAGCTTCTTTAAGAATAATTATTCGAATAGAATTAAGAGCTCCAGGAAGA
TGAATTAATAATGACCAAATTTATAATACAATTATTACTTCACATGCATTTATTATAATT
TTTTTTATAGTTATACCTTTTATAATTGGAGGATTTGGGAATTGATTAGTACCTTTAATA
ATTGGAGCTCCAGATATAGCTTTCCCACGAATAAACAATATAAGATTTTGACTTCTTATC
CCATCCTTATTTATATTATTAATAAGAAGAATTTTAGCATCCGGTTCAGGAACAGGATGA
ACAGTTTATCCTCCTTTATCTTCAATTATATACCATTCATCTATTTCTGTAGATTGTACA
ATTTTTTCATTACATATTGCAGGAATTTCTTCAATTATAGGAGCAATCAATTTTATTGTT
TCAATTATATTAATAAAAAATATTTCAATTAATTATGATCAAATTCCATTATTTCCATGA
TCAGTAAAAATTACTGCTATTCTATTACTTTTATCTTTACCTGTTCTAGCAGGAGCTATT
ACAATACTTTTAACTGATCGAAACTTAAATACATCATTTTTTGACCCTTCAGGAGGAGGA
GAT
>KF199988;tax=d:Eukarya,p:Arthropoda,c:Insecta,o:Hymenoptera,f:Halictidae,g:Lasioglossum,s:Lasioglossum pectorale
AATATTATATTTTATCTTTGCAATATGAGCAGGTATAATCGGAGCTTCTTTAAGAATAAT
TATTCGAATAGAATTAAGAGCTCCAGGAAGATGAATTAATAATGACCAAATTTATAATAC
AATTATTACTTCACATGCATTTATTATAATTTTTTTTATAGTTATACCTTTTATAATTGG
AGGATTTGGGAATTGATTAGTACCTTTAATAATTGGAGCTCCAGATATAGCTTTTCCACG
AATAAACAATATAAGATTTTGACTTCTTATCCCATCCTTATTTATATTATTAATAAGAAG
AATTTTAGCATCCGGTTCAGGAACAGGATGAACAGTTTATCCTCCTTTATCTTCAATTAT
ATACCATTCATCTATTTCTGTAGATTGTACAATTTTTTCATTACATATTGCAGGAATTTC
TTCAATTATAGGAGCAATCAATTTTATTGTTTCAATTATATTAATAAAAAATATTTCAAT
TAATTATGATCAAATTCCATTATTTCCATGATCAGTAAAAATTACTGCTATTCTATTACT
TTTATCTTTACCTGTTCTAGCAGGAGCTATTACAATACTTTTAACTGATCGAAACTTAAA
TACATCATTTTTTGACCCTTCAGGAGGAGGAGATCCAATTTTATATCAACATTTATTT
>KR875248;tax=d:Eukarya,p:Arthropoda,c:Insecta,o:Hymenoptera,f:Halictidae,g:Lasioglossum,s:Lasioglossum pectorale
TGAGCAGGTATAATCGGAGCTTCTTTAAGAATAATTATTCGAATAGAATTAAGAGCTCCA
GGAAGATGAATTAATAATGACCAAATTTATAATACAATTATTACTTCACATGCATTTATT
ATAATTTTTTTTATAGTTATACCTTTTATAATTGGAGGATTTGGTAATTGACTAGTACCT
TTAATAATTGGAGCTCCAGATATAGCTTTCCCACGAATAAATAATATAAGATTTTGACTT
CTTATTCCATCCTTATTTATATTATTAATAAGAAGAATTTTAGCATCCGGTTCAGGAACA

GGATGAACAGTCTATCCTCCTTTATCTTCAATTATATACCATTCATCTATTTCTGTAGAT
TGTACAATTTTTTCATTACATATTGCAGGAATTTCTTCAATTATAGGAGCAATCAATTTT
ATTGTTTCAATTATATTAATAAAAAATATTTCAATTAATTATGATCAAATTCCATTATTT
CCATGATCAGTAAAAATTACTGCTATTCTATTACTTTTATCTTTACCTATTCTAGCAGGA
GCTATTACAATA
>KR877113;tax=d:Eukarya,p:Arthropoda,c:Insecta,o:Hymenoptera,f:Halictidae,g:Lasioglossum,s:Lasioglossum pectorale
ATATTATATTTTATCTTTGCAATATGAGCAGGTATAATCGGAGCTTCTTTAAGAATAATT
ATTCGAATAGAATTAAGAGCTCCAGGAAGATGAATTAATAATGACCAAATTTATAATACA
ATTATTACTTCACATGCATTTATTATAATTTTTTTTATAGTTATACCTTTTATAATTGGA
GGATTTGGAAATTGATTAGTACCTTTAATAATTGGAGCTCCAGATATAGCTTTTCCACGA
ATAAACAATATAAGATTTTGACTTCTTATCCCATCCTTATTTATATTATTAATAAGAAGA
ATTTTAGCATCCGGTTCAGGAACAGGATGAACAGTTTATCCTCCTTTATCTTCAATTATA
TACCATTCATCTATTTCTGTAGATTGTACAATTTTTTCATTACATATTGCAGGAATTTCT
TCAATTATAGGAGCAATCAATTTTATTGTTTCAATTATATTAATAAAAAATATTTCAATT
AATTATGATCAAATTCCATTATTTCCATGATCAGTAAAAATTACTGCTATTCTATTACTT
TTATCTTTACCTGTTCTAGCAGGAGCTATTACAATACTTTTAACTGATCGA
>FJ864318;tax=d:Eukarya,p:Arthropoda,c:Insecta,o:Hymenoptera,f:Halictidae,g:Lasioglossum,s:Lasioglossum truncatum
TATATTATATTTTATTTTTGCCATATGAGCTGGAATAATTGGATCTTCTTTAAGAATAAT
CATCCGAATAGAATTAAGTGCTCCTGGTAAATGAATCAATAATGATCAAATTTATAATAC
TATTATCACTTCTCATGCATTTATCATAATTTTTTTTATAGTTATACCATTTATAATTGG
TGGATTTGGAAACTGATTAATTCCTTTAATAATTGGGGCCCCAGATATAGCATTCCCACG
AATAAATAATATAAGATTTTGATTACTTATCCCATCCTTATTTATACTTTTAATAAGAAG
AATTATATCTTCCGGATCAGGAACTGGATGAACTATTTATCCCCCATTATCAGCTTCATC
ATTTCACCCATCTATTTCTATAGATTGTACTATTTTTTCTCTACACATTGCAGGTATTTC
ATCTATTATAGGAGCTATTAATTTTATTGTATCCATTATATTAATAAAAAATATCTCTAT
TAACTTTGATCAAATTCCACTATTTCCATGGTCAGTAAAAATCACTGCTATTCTATTACT
ATTATCATTACCTGTTTTAGCAGGAGCTATTACTATACTTCTTACAGATCGAAATTTAAA
CACCTCATTCTTTGACCCCTCAGGAGGAGGAGATCCTATTTTATATCAACATTTATTT
>JF903561;tax=d:Eukarya,p:Arthropoda,c:Insecta,o:Hymenoptera,f:Halictidae,g:Lasioglossum,s:Lasioglossum truncatum
ATTTTTGCCATATGAGCTGGAATAATTGGATCTTCTTTAAGAATAATCATCCGAATAGAA
TTAAGTGCTCCTGGTAAATGAATCAATAATGATCAAATTTATAATACTATTATCACTTCT
CATGCATTTATCATAATTTTTTTTATAGTTATACCATTTATAATTGGTGGATTTGGAAAC
TGATTAATTCCTTTAATAATTGGGGCCCCAGATATAGCATTCCCACGAATAAATAATATA
AGATTTTGATTACTTATCCCATCCTTATTTATACTTTTAATAAGAAGAATTATATCTTCC
GGATCAGGAACTGGATGAACTATTTATCCCCCATTATCAGCTTCATCATTTCACCCATCT
ATTTCTATAGATTGTACTATTTTTTCTCTACACATTGCAGGTATTTCATCTATTATAGGA
GCTATTAATTTTATTGTATCCATTATATTAATAAAAAATATCTCTATTAACTTTGATCAA
ATTCCACTATTTCCATGGTCAGTAAAAATCACTGCTATTCTATTACTATTATCATTACCT
GTTTTAGCAGGAGCTATTACTATACTTCTTACAGATCGAAATTTAAACACCTCATTCTTT
GACCCCTCAGGAGGAGGAGATCCTATTTTATATCAACATTTATTT
>KT074054;tax=d:Eukarya,p:Arthropoda,c:Insecta,o:Hymenoptera,f:Halictidae,g:Lasioglossum,s:Lasioglossum leucozonium
AATACTATATTTTATCTTCGCAATATGATCAGGAATAATTGGAGCTTCATTAAGTATAAT
CATTCGTATAGAATTAAGAGCCCCCGGAAAATGAATTAATAATGATCAAATTTATAACAC
TATTGTTACATCCCACGCTTTCATTATAATCTTTTTTATAGTTATACCATTTATAATTGG
AGGATTTGGTAACTGATTAATTCCACTAATAATTGGAGCCCCTGATATGGCATTCCCTCG
AATAAATAATATAAGATTCTGATTATTAATCCCATCACTTATTATACTTCTATTAAGATC
AACTTTAACAGATGGATCAGGGACAGGATGAACTATCTACCCTCCTCTATCATCAATTAT

ATACCATTCCTCTAATTCTGTTGACTTCACAATTTTTTCTTTACATATTGCAGGAATATC
CTCAATTATAGGAGCCATCAATTTTATTGTTTCTATTTTATTAATAAAAAATATCTCTAT
TAAATTTGATCAAATTCCTTTATTTCCATGATCAGTAAAAATTACTGCTATTCTATTATT
ACTTTCTCTACCTGTTTTAGCAGGAGCTATTACAATATTATTAACAGATCGAAATTTAAA
TACTTCATTTTTTGACCCTTCAGGTGGAGGGGACCCAATTTTATACCAACATCTTTTT
