# Supplementary material for: Comparing modern identification methods for wild bees: Metabarcoding and image-based morphological taxonomic assignment
Source: PLoS One. 2024 Apr 2;19(4):e0301474. doi: 10.1371/journal.pone.0301474 (PMC10986983; doi:10.1371/journal.pone.0301474)
Supplement: S4 Table — Some workflows were restricted by the regional bee list while others were unrestricted. The full nucleotide (nt) database was searched, and then matches were "filtered" to only include accessions of taxa included on the regional bee list. Two custom databases were assessed: 1) an inclusive version generated from GenBank with a minimum length of 250 bases and 2) a curated version that was generated from both GenBank and Barcodes of Life databases, with a threshold of 400 bases and dereplicated within species. The final counts table used in this study was derived from the lowest common ancestor (LCA) method with the curated database. The RDP Classifier (Wang et al., 2007) is a Bayesian kmer-based classifier that requires prior training, for which the CO1 v.5 database of Porter and Hajibabaei (2018) was used. SINTAX (Edgar, 2016) is an alternative kmer-based classifier that does not require an external training set. (PDF) [file pone.0301474.s006.pdf]

S4 Table. Aggregate sequence counts for bee genera from six workflows. Some workflows were restricted by the regional bee list while others were unrestricted. The full nucleotide (nt) database was searched, and then matches were "filtered" to only include accessions of taxa included on the regional bee list. Two custom databases were assessed: 1) an inclusive version generated from GenBank with a minimum length of 250 bases, and 2) a curated version that was generated from both GenBank and Barcodes of Life databases, with a threshold of 400 bases and dereplicated within species. The final counts table used in this study was derived from the lowest common ancestor (LCA) method with the curated database. The RDP Classifier (Wang et al., 2007) is a Bayesian kmer-based classifier that requires prior training, for which the CO1 v.5 database of Porter and Hajibabaei (2018) was used. SINTAX (Edgar, 2016) is an alternative kmer-based classifier that does not require an external training set.

Genera	LCA (full nt database)	LCA (filtered nt database)	LCA (curated database)	RDP Classifier with CO1 v.5	SINTAX (inclusive database)	SINTAX (curated database)
Agapostemon	24867	26074	25649	25922	1941	26024
Andrena	17852	16444	16444	16444	16444	16444
Apis	350302	350405	350434	340838	350358	350421
Augochlora	1494	1494	1494	1482	1469	1494
Augochlorella	262	221	221	187	0	221
Augochloropsis	1179	1179	1179	0	212	1193
Bombus	495014	846906	509951	872854	885458	877215
Ceratina	125174	126132	126393	109020	125580	123472
Colletes	7431	7517	2341	2241	5612	2370
Dufourea	0	100	0	25	0	0
Epeolus	0	0	0	0	0	46
Eucera	14	0	0	0	0	0
Halictus	485060	485195	483891	458103	376344	471801
Heriades	3776	4499	4463	3878	4463	0
Hoplitis	36	173	46	46	164	150
Lasioglossum	57054	57317	55712	54782	57568	57046
Megachile	109783	115383	152833	147387	115276	157522
Melissodes	78587	37961	30183	65411	76398	76356
Seladonia	159	0	0	28	0	0
Sphecodes	1691	1691	1691	1691	12	1691
Svastra	660	660	660	0	660	660
Trigonisca	0	0	0	4398	0	0
Xylocopa	18610	19007	19033	18555	19033	19037
