# Supplementary material for: Comparing modern identification methods for wild bees: Metabarcoding and image-based morphological taxonomic assignment
Source: PLoS One. 2024 Apr 2;19(4):e0301474. doi: 10.1371/journal.pone.0301474 (PMC10986983; doi:10.1371/journal.pone.0301474)
Supplement: S5 Table — All clusters with the same taxonomic assignment were summed within samples to generate the counts table. Vial codes include the month the bees were collected (July [J] or August [A]), a three-digit identifier for the collection site, and whether the vial contained legs from small (S) or large (L) bees. (PDF) [file pone.0301474.s007.pdf]

S5 Table. Final counts table of individual metabarcode sequences that are attributed to each detected bee genus within each vial. All clusters with the same taxonomic assignment were summed within samples to generate the counts table. Vial codes include the month the bees were collected (July [J] or August [A]), a three-digit identifier for the collection site, and whether the vial contained legs from small (S) or large (L) bees.

Genus	A_048_L	A_048_S	A_169_L	A_169_S	A_286_L	A_286_S	A_293_L	A_293_S	A_343_L	A_391_L	A_391_S	A_395_L	A_395_S	A_466_L	A_466_S
Agapostemon	0	0	0	0	0	0	0	0	0	0	0	0	0	0	0
Andrena	0	0	0	0	0	0	0	0	0	0	0	0	0	0	0
Apis	0	0	0	0	34988	0	0	0	0	3368	0	0	0	7805	0
Augochlora	0	0	0	0	0	0	0	0	0	0	0	0	0	0	0
Augochlorella	0	0	0	0	0	0	0	0	0	0	0	0	0	0	0
Augochloropsis	0	0	0	0	0	0	0	0	0	0	0	0	0	0	0
Bombus	1847	0	7762	0	0	0	1068	0	0	3157	0	32672	0	20233	0
Ceratina	0	8665	0	0	0	27736	0	10776	0	0	0	0	0	0	0
Colletes	0	0	0	0	0	0	0	615	0	0	0	0	0	0	0
Halictus	0	29404	0	61613	0	0	0	992	0	0	40681	0	28033	0	27872
Heriades	0	0	0	0	0	0	0	0	0	0	0	0	0	0	0
Hoplitis	0	0	0	0	0	0	0	0	0	0	0	0	0	0	0
Lasioglossum	0	0	0	0	0	0	0	138	0	0	0	0	0	0	0
Megachile	0	0	0	0	0	0	792	0	41925	0	649	0	0	0	0
Melissodes	1310	0	0	0	0	73	10	42	0	0	0	0	0	0	10
Sphecodes	0	0	0	0	0	0	0	0	0	0	0	0	0	0	0
Svastra	0	0	0	0	0	0	0	0	0	120	0	0	0	0	0
Xylocopa	0	0	0	0	0	0	0	0	0	0	0	0	0	0	0

Genus	A_499_L	A_499_S	A_513_L	A_513_S	A_582_L	A_582_S	A_704_S	A_712_L	A_712_S	A_736_L	A_736_S	A_765_L	A_765_S	A_769_L	A_769_S
Agapostemon	0	0	0	0	0	0	0	0	0	0	239	0	68	0	0
Andrena	0	0	0	0	0	0	0	0	0	0	0	0	0	0	0
Apis	0	0	0	0	0	0	0	0	0	66945	0	0	0	6036	0
Augochlora	0	0	0	0	0	0	0	0	0	0	0	0	0	0	0
Augochlorella	0	0	0	0	0	0	0	0	0	0	0	0	0	0	0
Augochloropsis	0	0	0	0	0	0	1179	0	0	0	0	0	0	0	0
Bombus	967	0	4100	0	11190	0	0	5884	0	0	0	3860	0	14749	0
Ceratina	0	542	0	0	0	238	0	0	182	0	0	0	0	0	0
Colletes	0	0	0	0	0	1528	0	0	0	0	0	0	0	0	0
Halictus	0	30836	0	0	0	7604	0	0	43302	0	0	0	0	0	0
Heriades	0	0	0	0	0	0	0	0	0	0	0	0	0	0	0
Hoplitis	0	0	0	0	0	0	0	0	0	0	0	0	0	0	0
Lasioglossum	0	101	0	0	0	476	0	0	96	0	337	0	11	0	0
Megachile	989	0	0	0	114	0	0	0	236	0	74	0	0	0	0
Melissodes	188	18	0	125	0	67	0	0	0	0	0	36	0	0	1025
Sphecodes	0	0	0	0	0	0	0	0	0	0	0	0	0	0	0
Svastra	0	0	479	0	0	0	0	0	0	0	0	0	0	0	0
Xylocopa	0	0	0	0	0	0	0	0	0	0	0	0	0	0	0

Genus	A_818_L	A_818_S	A_825_L	A_825_S	A_834_L	A_834_S	A_850_L	A_850_S	A_880_L	A_880_S	A_937_L	A_937_S	A_955_L	A_955_S	A_ERS_L
Agapostemon	0	0	0	24883	0	0	0	80	25	0	0	0	0	0	0
Andrena	0	0	0	0	0	0	0	0	0	0	0	0	0	0	0
Apis	10329	0	0	0	0	0	0	0	0	0	7175	0	13429	0	22184
Augochlora	0	0	0	0	0	0	0	0	0	0	0	0	0	0	0
Augochlorella	0	0	0	0	0	0	0	0	0	0	0	0	0	0	0
Augochloropsis	0	0	0	0	0	0	0	0	0	0	0	0	0	0	0
Bombus	245	0	4008	0	9083	0	33182	0	669	0	25761	0	0	0	123
Ceratina	0	0	0	0	0	3887	0	0	0	0	0	0	0	38387	0
Colletes	0	0	0	0	0	0	0	0	0	0	0	0	0	0	0
Halictus	0	0	0	0	0	0	0	10375	0	0	0	0	0	0	0
Heriades	0	3989	0	0	0	0	0	0	0	0	0	0	0	0	0
Hoplitis	0	0	0	0	0	0	0	0	0	0	0	0	0	0	0
Lasioglossum	13	7435	0	0	0	3540	0	10383	10	0	0	0	0	0	0
Megachile	0	0	2817	0	0	0	0	0	253	25407	0	0	0	0	0
Melissodes	0	11	0	237	0	320	0	0	3926	1314	0	168	0	0	0
Sphecodes	0	0	0	0	0	0	0	0	0	0	0	0	0	0	0
Svastra	0	0	61	0	0	0	0	0	0	0	0	0	0	0	0
Xylocopa	0	0	0	0	0	0	0	0	0	0	0	0	19033	0	0

**Genus**	**J_048_L**	**J_048_S**	**J_169_L**	**J_169_S**	**J_286_S**	**J_343_L**	**J_343_S**	**J_391_L**	**J_391_S**	**J_395_L**	**J_395_S**	**J_466_L**	**J_466_S**	**J_499_S**	**J_513_L**	**J_582_L**
Agapostemon	0	0	0	0	0	0	0	0	0	0	0	0	45	0	0	0
Andrena	0	0	0	0	0	0	38	0	14	0	0	0	0	0	0	0
Apis	0	0	0	0	0	0	0	20364	0	0	0	6960	0	0	0	0
Augochlora	0	0	0	0	0	0	0	0	0	0	0	0	0	0	0	0
Augochlorella	0	0	0	35	0	0	0	0	0	0	0	0	27	0	0	0
Augochloropsis	0	0	0	0	0	0	0	0	0	0	0	0	0	0	0	0
Bombus	1482	0	38201	0	0	2315	0	13756	0	42241	0	871	0	0	31823	0
Ceratina	0	3369	0	0	6976	0	0	0	0	0	0	0	0	0	0	0
Colletes	0	0	0	0	0	0	0	0	0	0	0	0	0	0	0	0
Halictus	0	0	0	27677	0	0	0	0	1459	0	0	0	2679	0	0	0
Heriades	0	0	0	0	0	0	0	0	0	0	0	0	0	0	0	0
Hoplitis	0	0	0	0	0	0	0	0	0	0	0	0	0	0	0	0
Lasioglossum	0	0	0	2699	0	0	0	0	0	0	0	0	0	1095	0	0
Megachile	0	0	0	0	0	0	0	0	0	0	0	0	0	0	0	61347
Melissodes	0	0	0	0	17363	0	69	0	68	0	0	0	0	0	0	0
Sphecodes	0	0	0	0	0	0	0	0	1691	0	0	0	0	0	0	0
Svastra	0	0	0	0	0	0	0	0	0	0	0	0	0	0	0	0
Xylocopa	0	0	0	0	0	0	0	0	0	0	0	0	0	0	0	0

Genus	J_582_S	J_704_L	J_704_S	J_712_L1	J_712_L2	J_712_S	J_736_L	J_736_S	J_765_L	J_765_S	J_769_L	J_769_S	J_818_L	J_818_S	J_825_L
Agapostemon	10	0	0	0	0	0	0	299	0	0	0	0	0	0	0
Andrena	0	0	0	0	0	0	0	16310	0	0	0	0	0	0	0
Apis	0	14382	0	0	0	0	0	0	0	0	0	315	47680	0	8071
Augochlora	0	0	0	0	0	0	0	0	0	0	0	0	0	0	0
Augochlorella	0	0	0	0	0	159	0	0	0	0	0	0	0	0	0
Augochloropsis	0	0	0	0	0	0	0	0	0	0	0	0	0	0	0
Bombus	0	867	0	2977	1394	0	3120	0	0	0	2017	0	0	0	17535
Ceratina	0	0	0	0	0	0	0	0	0	0	0	0	0	2137	0
Colletes	0	0	0	0	0	0	0	0	0	0	0	0	0	0	0
Halictus	24978	0	0	0	0	6907	0	0	15145	0	0	0	0	11460	0
Heriades	0	0	0	0	0	0	0	0	0	0	0	0	0	0	0
Hoplitis	0	0	0	0	0	0	0	0	0	0	0	0	0	26	0
Lasioglossum	562	0	0	0	0	17	0	0	63	0	0	0	0	119	0
Megachile	0	0	0	0	0	6860	0	0	0	0	0	0	0	26	0
Melissodes	0	0	0	0	0	0	0	0	819	2314	0	0	0	14	0
Sphecodes	0	0	0	0	0	0	0	0	0	0	0	0	0	0	0
Svastra	0	0	0	0	0	0	0	0	0	0	0	0	0	0	0
Xylocopa	0	0	0	0	0	0	0	0	0	0	0	0	0	0	0

Genus	J_825_S	J_834_S	J_850_L	J_850_S	J_880_S	J_937_L	J_937_S	J_955_L	J_955_S	J_ERS_L	J_ERS_S
Agapostemon	0	0	0	0	0	0	0	0	0	0	0
Andrena	0	0	0	0	82	0	0	0	0	0	0
Apis	0	0	0	0	0	0	0	0	0	24973	0
Augochlora	0	0	0	283	0	0	546	0	0	0	665
Augochlorella	0	0	0	0	0	0	0	0	0	0	0
Augochloropsis	0	0	0	0	0	0	0	0	0	0	0
Bombus	0	0	8134	0	0	43524	0	42359	0	3617	0
Ceratina	0	11692	0	3979	0	0	34	0	0	0	585
Colletes	0	0	0	0	0	0	0	0	0	0	0
Halictus	40852	0	0	6349	21750	0	15924	0	0	0	26657
Heriades	0	0	0	276	0	0	198	0	0	0	0
Hoplitis	0	0	0	20	0	0	0	0	0	0	0
Lasioglossum	34	8096	0	0	41	0	134	0	20242	0	40
Megachile	0	0	0	0	11344	0	0	0	0	0	0
Melissodes	602	0	0	0	0	0	0	0	0	0	0
Sphecodes	0	0	0	0	0	0	0	0	0	0	0
Svastra	0	0	0	0	0	0	0	0	0	0	0
Xylocopa	0	0	0	0	0	0	0	0	0	0	0
